# Yeast Phenomics: An Experimental Approach for Modeling Gene Interaction Networks that Buffer Disease

**DOI:** 10.3390/genes6010024

**Published:** 2015-02-06

**Authors:** John L. Hartman, Chandler Stisher, Darryl A. Outlaw, Jingyu Guo, Najaf A. Shah, Dehua Tian, Sean M. Santos, John W. Rodgers, Richard A. White

**Affiliations:** 1 Department of Genetics, University of Alabama at Birmingham, 730 Hugh Kaul Human Genetics Building, 720 20th Street South, Birmingham, AL 35294, USA; E-Mails: cstisher@uab.edu (C.S.); doutlaw@uab.edu (D.A.O.); guo.jingy@gmail.com (J.G.); najafali@gmail.com (N.A.S.); dehuat@gmail.com (D.T.); ssantos@uab.edu (S.M.S.); jwrodger@uab.edu (J.W.R.); 2 Department of Statistics and Michael Smith Laboratories, University of British Columbia, 3182 Earth Sciences Building, 2207 Main Mall, Vancouver, BC V6T-1Z4, Canada; E-Mail: rickw@stat.ubc.ca

**Keywords:** yeast phenomics, yeast models of human disease, cell proliferation phenotypes or cell proliferation parameters (CPPs), gene interaction networks, quantitative high throughput cell array phenotyping (Q-HTCP), genetic buffering, cystic fibrosis (CF), human-like (HL) yeast media, ammonium toxicity, recursive expectation-maximization clustering (REMc)

## Abstract

The genome project increased appreciation of genetic complexity underlying disease phenotypes: many genes contribute each phenotype and each gene contributes multiple phenotypes. The aspiration of predicting common disease in individuals has evolved from seeking primary loci to marginal risk assignments based on many genes. Genetic interaction, defined as contributions to a phenotype that are dependent upon particular digenic allele combinations, could improve prediction of phenotype from complex genotype, but it is difficult to study in human populations. High throughput, systematic analysis of *S. cerevisiae* gene knockouts or knockdowns in the context of disease-relevant phenotypic perturbations provides a *tractable experimental approach* to derive gene interaction networks, in order to deduce by cross-species gene homology how phenotype is buffered against disease-risk genotypes. Yeast gene interaction network analysis to date has revealed biology more complex than previously imagined. This has motivated the development of more powerful yeast cell array phenotyping methods to globally model the role of gene interaction networks in modulating phenotypes (which we call yeast phenomic analysis). The article illustrates yeast phenomic technology, which is applied here to quantify gene X media interaction at higher resolution and supports use of a human-like media for future applications of yeast phenomics for modeling human disease.

## 1. Introduction and Perspective

### 1.1. Buffering of Phenotypes: Yeast Phenomic Analysis Reveals Gene Interaction Networks Responsible for Phenotypic Variability

It is increasingly recognized that the phenotypic effects of environmental or genetic perturbation depend upon the functional/allelic status of interacting loci [1,2]. We consider genetic buffering to underlie phenotypic stability/variability within a population and to derive from interaction between sets of gene variants and environmental factors, and that sets of buffering genes represent functional networks that distinctly modulate each phenotype [3]. In *S. cerevisiae*, genome-wide analysis shows that genes interact extensively [4,5]. Thus, in yeast one can experimentally define functional gene networks in terms of their capacity to buffer, or stabilize phenotypes. Buffering networks mask functional genetic variants subject to natural selection, and thus comprise a reservoir within populations for the complex expression of phenotypes [3]. Gene interaction networks are constrained evolutionarily across species and diverse phenotypes [6,7,8,9]. Therefore yeast gene interaction networks can provide insight to buffering and variable expression of disease when yeast phenomic experiments are designed within a cellular context analogous to human biology [10].

By yeast phenomic analysis, we mean systematic, comprehensive, quantitative analysis of gene interaction using the comprehensive collection of yeast gene knockout/knockdown (YKO/KD) strains [10,11,12,13,14]. The YKO/KD library has been widely used to identify genes that affect yeast cell proliferation (also called fitness) on compounds of interest,* i.e.*, gene-drug interaction [15,16,17,18]. It has also been used for studying gene-gene interaction, where a mutation of interest is introduced to the background of the entire YKO/KD library, and the effect of interaction between loci on fitness can be assessed genome-wide [19,20]. The Boone laboratory is analyzing all pairwise interactions in this way, toward a complete interaction map for a eukaryotic cell [21], and the Weissman laboratory has examined all possible combinations for selected sets of genes, called E-MAPs (epistatic mini array profiles) [22,23,24]. These and other studies have demonstrated, on a genomic scale, networks of interdependent genes that produce phenotypes. Thus gene interaction networks will be an essential component of complete models of any cell, organism, or disease [25].

We anticipate gene interaction networks will be specific to (and can potentially define) distinct diseases. Such disease-buffering networks will also exhibit context-specificity with respect to environmental inputs. Most large-scale gene-gene interaction studies have been carried out pairwise in a single, or limited number of media. However, genes interact with environmental factors [14,26] and in more than pairs [27], and we are just beginning to learn about the dynamic nature of yeast gene interaction networks with respect to more than two genetic or environmental perturbations. These observations of complexity point to a need for greater phenotypic resolution to develop quantitative models. In this article, we discuss our effort to develop technology to resolve yeast gene interaction networks more quantitatively so that the YKO/KD collection can be used to model disease buffering networks more precisely. Considering the evolutionarily conserved nature of gene interaction [28,29], prior success in using yeast as a genetic model for human disease, and advances in technology for phenomic analysis in yeast, we build the rationale moving forward for more extensive efforts to construct yeast phenomic models of disease buffering networks.

### 1.2. The Need for Quantitative Phenotyping to Experimentally Derive Buffering Networks

To enable phenomics, we have improved methodology for quantifying yeast gene interaction on a genomic scale. It remains technologically challenging to collect and analyze genetic interaction data due to the combinatorial explosion inherent to such networks [30]. Most automated YKO/KD phenotyping has been done either by microarray hybridization of total genomic DNA harvested at different time points from liquid cultures of pooled mutants to compare relative fitness [17,31,32], or by pinning small amounts of cell paste from mutant cultures arrayed on agar media and measuring the area of outgrowth of the spherical culture at an endpoint [33,34]. A few studies have also performed large-scale phenotyping by time series analysis of liquid culture arrays [14,35,36]. The quantitative high throughput cell array phenotyping (Q-HTCP) methodology we have been developing is based on the classic method of dilution and spotting of liquid cell suspension to agar (normally analyzed qualitatively and reported as an image), with the modification that kinetic growth curves are obtained by serial imaging and image analysis [16]. We discovered that growth curves generated by kinetic analysis of cell array images, if fit to a logistic growth function, yield cell proliferation parameters (CPPs) useful for measuring gene interaction [10,37]. In this article we illustrate improved resolution for quantifying gene interaction with Q-HTCP data, which reveals gene X media interaction and suggests a human-like (HL) yeast media could reduce false positive results when validating yeast phenomic results in human cells.

### 1.3. Are Gene Interaction Networks That Buffer Human Disease Evolutionarily Conserved?

Fundamental processes shared by eukaryotic cells such as cell cycle control and protein secretion are genetically conserved across evolution [38,39]. The importance of such processes in disease is evident, but whether digenic inputs involving allelic variants that interact with respect to yeast phenotypes are predictive for expression of human disease is only beginning to be explored. A purpose of this article is to advocate for the use of *S. cerevisiae* to create experimental phenomic models of gene interaction to investigate genetic buffering of human disease.

There are multiple examples suggesting that yeast can serve as useful models of human disease. One example is neuronal degeneration, where disease-related human proteins have been expressed in yeast to discover yeast genes that modulate toxicity, with subsequent validation in animal models of neurodegeneration [40,41,42,43,44,45]. Another disease model investigates the gene interaction network influencing biogenesis of the CFTR-∆F508 gene product, the main cause of cystic fibrosis (CF). A yeast homolog of CFTR was constructed with a mutation of the conserved disease-relevant F508 residue (Yor1-∆F670) to screen the YKO/KD library for modifiers. Conservation of gene interaction was demonstrated by comparing the Yor1-∆F670 phenomic screen results to the literature reporting their effects on CFTR-∆F508 biogenesis (when knocked down by RNA interference) [10].

In addition to modifiers of Mendelian disease, such as CF, and multifactorial diseases like neurodegeneration, yeast phenomics holds promise for modeling organismal processes, including aging and mitochondrial dysfunction, which are relevant to a wide variety of human disease [46,47,48,49]. Numerous other genetic models of human disease are being developed, and these span across yeast and other model organisms [50]. A great advantage of yeast models of human disease is the relative ease of genome-wide phenotypic analysis, nevertheless translation of these models typically necessitates a reductionist approach, focusing on validation of a few individual genes. Thus, an important future direction is integrative, systems level modeling of disease buffering networks.

### 1.4. Experimental Resources and Technology for Yeast Phenomic Analysis

To quantify pair-wise gene interaction, phenotypic measures are needed for the wild-type and mutant cell, in the perturbed and unperturbed context [16]. The YKO/KD strain collection provides a genomic set of mutants for systematic analyses of gene interaction. Perturbations can take the form of additional gene mutations introduced by the synthetic genetic array method [4], small molecules, or environmental variations. A null hypothesis, predictive of phenotype, is required so that “interaction” can be quantified as departure from expectation [51]. The power and resolution to analyze gene interaction networks is a function of the precision, accuracy, and quantitative resolution of phenotypic data.

To advance quantitative analysis of yeast mutant libraries, we have developed an automated workflow with cell-array printing, time-lapse imaging, image analysis, growth-curve fitting, and quantification of gene interaction [10,16,37,52]. Cell-array imaging can be performed manually with a commercial grade scanner (with built-in transparency unit) or using a new imaging robot, which can be integrated with a robotic incubator (we use the Cytomat 6001 from Thermo Fisher Scientific, Asheville, NC, USA). The robotic Q-HTCP system has a culture capacity of 72,576 (189 × 384-cultures arrays), exceeding commercial systems for growth-curve analysis by over 500-fold [30]. While single time point analysis of colony outgrowth area is higher throughput for breadth of global interaction analysis [33,34], Q-HTCP is more quantitative for greater resolution in specific disease models [10].

### 1.5. Examples of Yeast Phenomic Modeling of Disease in Our Laboratory

In accord with this special issue, we discuss technology, current applications and speculate that the application of yeast phenomic modeling for human disease research is the tip of an iceberg, where the primary challenge is to identify phenotypes for which experimental derivation of gene interaction networks in yeast discovers gene modules relevant to variable disease expression in humans [53,54,55,56,57,58].

Cystic fibrosis is a model we are developing to investigate whether yeast phenomic analysis could reveal a gene interaction network relevant to a Mendelian human disorder [10]. There are two unique sources of information to validate the yeast phenomic model of CFTR-∆F508, one being the extensive siRNA literature involving targets that rescue the processing defect in human cell models, and another being genomic data from the CF GWAS consortium, which is studying large cohorts of CF patients [59]. Thus CF represents a promising test case for the paradigm of evolutionarily conserved yeast gene interaction networks that buffer/modulate the expression of human disease.

In contrast to CF, a genetically tractable disease due to highly penetrant loss of function mutations at a single locus, cancer and aging represent polygenic and complex disease. To model cancer we are studying the genetic buffering networks of ribonucleotide reductase (RNR) and target of rapamycin (TOR), which are evolutionarily conserved regulators of DNA replication and cell growth, respectively [16,60]. RNR and TOR are involved in tumorigenesis and progression and comprise important targets for development of chemotherapeutic agents [61,62,63,64]. With the hypothesis that the integration of these networks provides a master level of cell cycle regulation (*i.e.*, DNA and protein synthesis), an unexpected connection between TOR and RNR suggested by this model is threonine catabolic flux, which we believe could be regulated by TOR signaling and that we’ve found to be a mechanism for buffering depletion of dNTP pools due to loss of RNR function [16,60]. Threonine catabolism was also found to be important for mouse embryonic stem cell survival due to increased need for DNA replication and also a role in histone modification [65,66,67]. A third model is aging, which represents a disease-associated cellular process that can be interrogated by phenomic analysis of the YKO/KD libraries. We are using Q-HTCP to measure chronological lifespan (CLS) by growth curve analysis of stationary phase cultures that are periodically rescued to fresh media so that change in the viable percentage of cells can be estimated during the aging process [68], as a function of every gene and different nutrient conditions.

In summary, every cellular process has a disease correlate and *vice-versa*. Gene interaction, though rare on a percentage basis, is frequent in aggregate and contributes greatly to disease expression. Yeast phenomics enables experimental derivation of gene interaction networks in a comprehensive and quantitative manner that is unparalleled for modeling genotype-phenotype complexity.

### 1.6. Development of a Human-Like (HL) Media for Yeast Phenomic Studies

With success of yeast phenomic disease models, homologues of yeast genes will be increasingly tested for conserved gene interaction in their human cell context. To improve the positive predictive value of such models, we sought to reduce the potential for interaction due to uncontrolled differences in culture media for yeast and human cells. We used Q-HTCP to analyze the YKO/KD library for gene interaction in standard media* vs.* a new media we designed to more closely resemble what is used for human cell culture. This investigation was motivated in part by work from the Botstein laboratory showing high potassium is required in standard defined (“dropout”) yeast media to help buffer toxicity from ammonium sulfate [69], an ingredient omitted from human tissue culture media. We also observed ammonium toxicity, and reduced other ingredients to create a yeast media that resembles human cell culture media but supports normal growth of the YKO/KD reference strain (BY4741). We characterized the HL media by genome-wide Q-HTCP analysis of the YKO/KD collection to identify gene X media interaction. Our observations of deletion strains with differential cell proliferation across media supports the possibility that HL media could improve the translational relevance of yeast phenomic screens. We also demonstrate in some cases that the growth inhibitory effect of small molecules depends on media. Although beyond the scope of the paper to formally assess all of these factors in the context of an actual human disease model, our results thus far suggest careful consideration of media type is useful for assay optimization and interpretation.

## 2. Methods

### 2.1. Yeast Media and Strains

The YKO/KD library was obtained from Research Genetics (Huntsville, AL, USA) and Open Biosystems (Huntsville, AL, USA). The genetic background for the YKO/KD library was BY4741 (S288C *MAT*a *ura3-∆0 his3-∆1 leu2-∆0 met17-∆0*). Yeast media was YP (10 g/L Yeast Extract, 20 g/L peptone) with either 2% dextrose (YPD) or 3% glycerol/3% ethanol (YPEG) as carbon source. The Cold Spring Harbor (CSH) synthetic complete (SC) dropout media [70] was also used with either carbon source as was the “human-like” (HL) media. The recipe for HL media was derived from the CSH SC media with the following modifications: ammonium sulfate was removed (the normal 5 g/L was reduced to 0.5 g/L in HL + AS media). Potassium phosphate was reduced from 1 to 0.5 gm/L. Magnesium sulfate was reduced from 0.5 to 0.05 g/L. PABA was removed from the amino acid powder and inositol was dropped from 0.0734 to 0.025 g/L. Leucine was reduced from 0.367 to 0.1468 g/L. The potassium phosphate and magnesium sulfate modifications were introduced by ordering custom yeast nitrogen base (without ammonium sulfate) from Sunrise Science (San Diego, CA, USA). The HL media recipe was partly guided by comparison with RPMI and DMEM media components.

### 2.2. Quantitative High Throughput Cell Array Phenotyping (Q-HTCP)

A Caliper Sciclone 3000 liquid handling robot was used for cell array printing (384-culture arrays), integrated with a custom imaging robotic system and a Cytomat 6001, having capacity for 189 arrays (Thermo Fisher Scientific, Asheville, NC, USA). Images were taken every 2–3 hours and analyzed as previously described to obtain cell proliferation parameters [10], using the logistic equation, *G(t)* = K/(1 + *e ^−r(t−l)^*), assuming *G(0) < K*, where *G(t)* is the image intensity of a spotted culture* vs.* time, *K* is the final carrying capacity, *r* is the maximum specific growth rate, and *l* is the time that maximal absolute growth rate occurs, when *G(t)* = K/2 [51].

### 2.3. Quantification of Gene Interaction 

For the genome-wide screen, cell proliferation phenotypes (CPPs) were used to calculate gene X media interaction in the following way: the CPP for each deletion strain was adjusted by its difference compared to the median CPP among 384 cultures of the reference stain in YP (YPD or YPEG), and by the difference of the median CPP of the reference strain in YP with the respective test media. After the normalizations, the difference between the deletion strain on CSH or HL media* vs.* YP media was taken as the interaction value. YP-dextrose was the control for all dextrose media and YP-ethanol/glycerol for all ethanol-glycerol media. For the drug x gene x media interaction analysis, CPPs were obtained in the same way and control arrays containing the same media without drug were subtracted from the respective drug gradient arrays.

### 2.4. Recursive Expectation Maximization Clustering (REMc)

Clustering was performed as previously described [12], followed by hierarchical clustering and heat map generation applying an R script (http://www.r-project.org/) to each REMc cluster. REMc was performed with a 16-dataset matrix, including the ORF-deletion effect (“shift” value) on YP media and the interaction values for defined media, for both K and L parameters. A custom java code that utilizes Weka (www.cs.waikato.ac.nz/ml/weka) was used to generate clusters. After REMc, a python script is used to format the clusters for analysis by the command line version of the Gene Ontology (GO) Term Finder (GTF) downloaded from http://search.cpan.org/dist/GO-TermFinder/ [71]. GTF searches for enrichment of Gene Ontology terms in each cluster by comparing the ratio of genes assigned to a term within a cluster to the total number of genes tested in the experiment having that term.

## 3. Results

### 3.1. Using Growth Curve Parameters as High-Resolution, Quantitative Phenotypes

**Figure 1:**
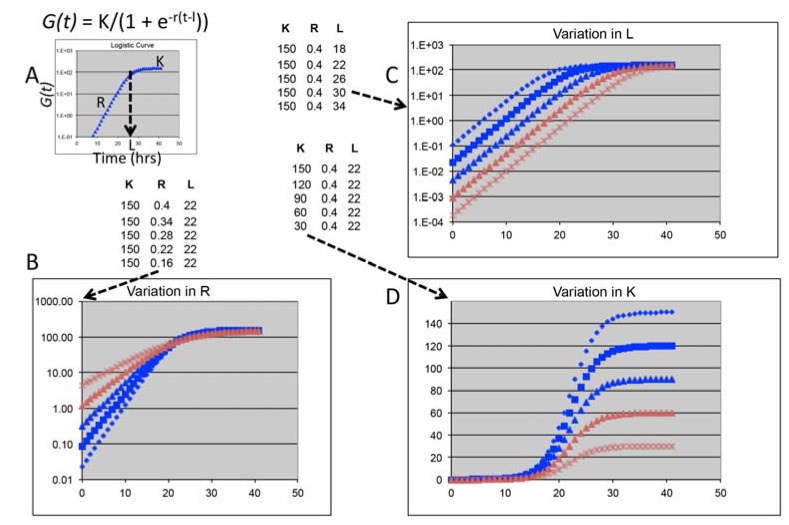
Cell proliferation parameters (CPPs) for quantifying gene interaction are obtained from fitting Q-HTCP data with a logistic growth function. The equation and an example growth curve are shown in panel A; CPPs are phenotypes for measuring gene interaction: K is the carrying capacity (final growth density, quantified as average pixel intensity), L is the time (h) it takes for a culture to reach K/2, and R is the maximum specific rate. Each parameter is independent of the other, because *G(0)* is treated as unknown. The CPPs for each graph are given in the respective table to illustrate variation in each parameter.

A fundamental challenge of phenomics for all organisms is quantifying phenotypes with respect to gene interaction on a genomic scale. Every disease has multiple phenotypes with multiple different genes contributing to each one. There is a functional spectrum among different alleles for each gene, and the influence of a given allele on the phenotype depends on combination with particular alleles at different loci [1]. Given this genetic complexity of phenotypic expression, yeast offers key advantages for mapping gene interaction as comprehensively and quantitatively as possible with respect to both environment and other genes: (1) Much of the overall fitness is encapsulated by the phenotype of cell proliferation, which lends comprehensiveness; and (2) Cell proliferation is a continuous trait that’s straightforward to quantify, where analysis with a logistic growth function resolves fitness into three components, providing additional resolution for phenomic analysis and gene interaction network construction (Figure 1).

Though phenotypes are more complex in humans than yeast, it is possible to extrapolate across species between potentially any phenotype, based upon gene interaction networks that function similarly across evolution [6,7,8,9]. Cell proliferation is under strong selection in yeast and involves evolutionarily conserved genes, which may be involved in the production of different phenotypes subject to natural selection for other reasons across evolution. For example, in the yeast model of CFTR-∆F508, gene interactions that influence Yor1-∆F670, also influence CFTR-∆F508 biogenesis in human cell lines [10]. In yeast, “∆F biogenesis” can be selected for by cell proliferation on oligomycin, whereas in humans it is assayed by chloride transport. Although the cellular and organismal phenotypes associated with Yor1-∆F670 are different from those of CFTR-∆F508, the network of gene interactions affecting biogenesis of the respective proteins is similar [10]. Hardly unique to ABC transporters, quantifying cell proliferation of yeast mutant arrays provides a powerful strategy for broadly analyzing eukaryotic gene networks that influence a variety of human disease.

Q-HTCP is an automated method for collecting over 70,000 growth curves per experiment. Time series image data is taken from miniature lawns of proliferating agar cell culture arrays each representing a different defined mutant [10,16,37,52]. These data fit closely to a logistic growth function [37], yielding independent quantitative CPPs with which to calculate gene interaction (Figure 1A). Cell proliferation parameters represent fundamentally distinct phenotypes under differential natural selection and thus regulated by different gene interaction networks buffering each parameter in a context/perturbation-specific manner.

Of the three parameters, we have found the L parameter to be especially useful (Figure 1C). Oligomycin growth inhibition is evident best by L in the Yor1-∆F508 model for CFTR-∆F508 [10]. Similarly, in the chronological lifespan (CLS) model of aging, where the yeast phenotype is duration of post-mitotic survival after entry into stationary phase [72], L best reflects longevity, which is quantified as the percentage of cells with persistent ability to regrow upon transfer to fresh media [73]. Q-HTCP can be used for phenomic analysis of CLS by collecting growth curves at weekly intervals for the entire genomic collection of YKO/KD strains. If other growth parameters,* i.e.*, rate, carrying capacity and lag remain consistent across the aging process, changes in L would estimate CLS by a rightward shift of the growth curve over time, reflecting reduction in the number of colony forming units* vs.* age. Other gene- or disease-related processes might be better modeled with a different parameter; for example, mitochondrial function could be modeled by change in K, resulting from a relative decline in growth after the diauxic shift (Figure 1D).

### 3.2. A Human-Like Yeast Media to Increase Positive Predictive Value of Yeast Phenomic Models

**Figure 2:**
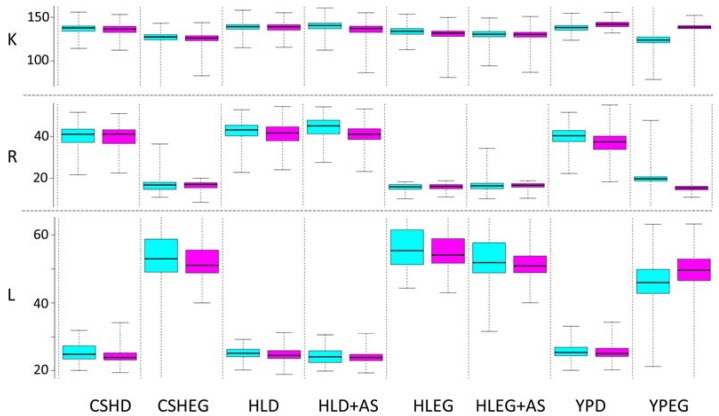
Cell proliferation phenotypes (CPPs) for BY4741 are similar in HL and standard yeast media. Box plots represent the central 75% (colored box), median (bold bar), and range (whiskers). Media types include Cold Spring Harbor (CSH), human-like (HL) with or without 0.5 gm/L ammonium sulfate (AS), or rich yeast/peptone (YP). Each media type was prepared with either dextrose (D) or ethanol glycerol (EG) as a carbon source. CPP abbreviations are described in Figure 1. Values of R were multiplied by one hundred. Data are from two different 384-culture liquid arrays (teal and lavender box plots) printed onto each media type. The main conclusion is that cell proliferation is similar for each media, controlled for dextrose or ethanol/glycerol as carbon source. This experiment demonstrates feasibility to modify synthetic defined yeast media to resemble that used for human cells. It stands to reason that similarity in the media will improve reproducibility of gene interaction across species by reducing gene interaction with media components.

Cell culture media components can modulate phenotypes, representing a form of gene x environment interaction. To increase translational relevance of yeast phenomic models to cultured human cells, we introduced changes to defined yeast media to resemble human cell culture media. By reducing differences between yeast and human cell media, discovery of gene interaction from yeast phenomic screens can be focused on the disease network by reducing media-specific gene interaction. The following changes were made to the Cold Spring Harbor synthetic complete media [70] to create HL media: (1) in the yeast nitrogen base, magnesium sulfate was reduced 90% (from 0.5 to 0.05 g/L), potassium phosphate was reduced 50% (from 1 to 0.5 g/L), and ammonium sulfate (5 g/L) was removed (0.5 g/L ammonium sulfate was added to HL + AS media); (2) in the complete dropout powder, inositol was reduced from 0.0734 to 0.025 g/L, leucine was reduced from 0.367 to 0.1468 g/L, and PABA (0.0734 g/L) was removed. The YKO/KD collection reference strain, BY4741, exhibited robust cell proliferation parameters across these media modifications (Figure 2).

### 3.3. Phenomic Analysis Reveals Clusters of Gene X Media Interaction

**Figure 3:**
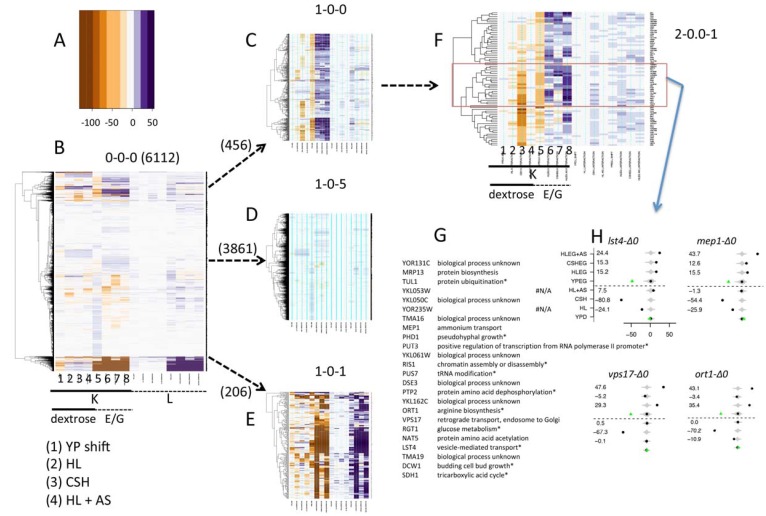
REMc reveals gene X media interaction modules. Q-HTCP was performed for the YKO/KD collection on the indicated media, and interactions were calculated for defined media using YPD or YPEG as a control (see text and methods). REMc was used to mine interaction values derived from K and L for shared patterns. (**A**) A color scale was used to visualize interaction values, which are positive/blue with respect to the K phenotype if YKO/KD strains have higher fitness in defined than YP media, relative to the reference strain. Conversely interactions are negative/brown with respect to L (less time required to reach K/2) if YKO/KD strains exhibit a relative increased in fitness on defined *vs.* YP media; (**B**) The entire data matrix (root cluster) was analyzed by hierarchical clustering (before REMc). The x-axis order (media conditions and growth parameters) is the same in all panels; (**C**–**E**) The first round of REMc yielded six clusters, three of which are shown. The YP “shift” refers to the difference between the respective YKO/KD strain and the median of the reference (see Figure 2 and panel H); (**C**) Cluster 1-0-0 contains 456 YKO/KD strains, many of which exhibit lower fitness (lower K and longer L) on YPEG media (column 5 and 13) that is partially alleviated when grown on defined media (columns 6 (CSH/EG), 7 (HL/EG) and 8 (HL + AS/EG)); (**D**) Cluster 1-0-5 contains a majority (3861) of YKO/KD strains that exhibit similar phenotypes on all media; (**E**) Cluster 1-0-1 contains genes that share reduced fitness on non-fermentable media (columns 6–8 and 14–16). Some strains reveal this phenotype on HL media (columns 6, 8, 14, and 16), but not YPEG or CSHEG defined media (columns 5, 7, 13 and 15); (**F**) As part of the REMc workflow, clusters are iteratively analyzed until terminal clusters are obtained. Cluster 2-0.0-1, obtained in the second round from cluster 1-0-0, contained strains with low fitness in CSHD (column 3), but not YPD, HLD, or HLD + AS (columns 1, 2 and 4). A sub-cluster (red box) contains YKO/KD strains whose functions are listed in the table (**G**), with the data for particular genes discussed in the text given in (**H**), where the interaction for each gene on each media type is plotted for the K parameter. Green diamonds indicate the ORF effect, or “shift” (difference between deletion strain and reference strain on YPD or YPEG, respectively) by which data are normalized/shifted [10].

To investigate the potential significance of HL media for yeast phenomic analysis, we collected growth curves for all (~6000) YKO/KD strains on eight different media, alternatively using dextrose or ethanol/glycerol as carbon sources in YP (YPD/YPEG), CSH synthetic complete (CSHD/CSHEG), or HL without (HLD/HLEG) or with 0.5 g/L ammonium sulfate (HLD + AS/HLEG + AS). Interactions were calculated by normalizing growth of hundreds of replicate cultures of the reference strain (BY4741) on each media to growth on YP media and also normalizing by the effect of the open reading frame on growth in the YP media (*i.e.*, YPD was used as the reference media for CSHD and HLD, while YPEG was used for CSHEG and HLEG). We used REMc to objectively define groups of genes with shared patterns of media interaction, and hierarchical clustering with heat maps to best visualize patterns of interaction (Figure 3, Supplemental Data Files 1 and 2).

REMc analysis revealed six first round clusters. Cluster 1-0-0 had reduced fitness on YPEG (column 5), which was alleviated in defined media (columns 6–8) (Figure 3C). As typical for most phenotypes, the majority (3816) of strains exhibited little or no interaction, as illustrated by the large cluster, 1-0-5, (Figure 3D). YKO/KD strains failing to grow on every E/G media comprised a distinct cluster (Figure 3B at bottom, extreme negative K and positive L interaction indicates no growth in columns 5–8 and 13–16), and consisted of genes enriched for GO Terms related to respiratory function as expected (see also cluster 1-0-2 in Supplemental Data Files 1 and 2). Cluster 1-0-1 highlights gene deletions that have reduced fitness on defined media with non-fermentable carbon source, but not rich media or media with glucose as the carbon source; moreover, more strains display this phenotype on HL than on CSH media (Figure 3E). Cluster 1-0-1 was enriched for genes functioning in mitochondrial and ribosomal processes, consistent with roles in buffering combined perturbations of carbon and nitrogen availability (see Supplemental Data Files 1 and 2).

Within cluster 1-0-0 (Figure 3C), several of the strains have reduced fitness on CSHD media. This set of genes was better highlighted in the second round cluster 2-0.0-1 (Figure 3F). There was no enrichment in cellular processes annotated by gene-ontology for clusters 1-0-0 or 2-0.0-1, which is often true for interactions modulating relatively unstudied phenotypes. However, we did find genes in cluster 2-0.0-1 that are implicated in amino acid metabolism, amino acid permease trafficking and ammonium efflux (Figure 3G), consistent with a report from the Botstein laboratory suggesting that up-regulation of amino acid biosynthesis and excretion is necessary to buffer ammonium toxicity [69]. In support of this model, Lst4 is involved in trafficking of Gap1, the general amino acid permease [74]. The *gap1-∆0* strain had no phenotype, possibly due to redundancy among amino acid permeases, and thus we hypothesize that Lst4 could regulate a module of permeases such that its functional loss (but not disrupting any single permease), would alter fitness in the context of ammonium toxicity introduced by the CSH media. The growth inhibitory phenotype suspected to be due to ammonium sulfate in CSH would be alleviated by its removal from HL media [69]. Similarly, deletion of *MEP1*, the high flux ammonium transporter reduced fitness in CSH media, but not HL. Deletion of *VPS17* and *VPS24*, which are involved with protein sorting to the endosome and vacuole also appear to buffer CSH media, based on their being relatively dispensable in HL media (Figure 3H), perhaps implicating their function too in the regulation of amino acid or ammonium permeases. Another gene in this cluster, *ORT1*, is implicated in ammonium toxicity in humans. *ORT1* is required for arginine biosynthesis due to its function as a mitochondrial ornithine transporter, and its human ortholog ORNT1 is causative of the recessive disease, hyperornithinemia-hyperammonemia-homocitrullinuria syndrome (http://omim.org/). In summary, cluster 2-0.0-1 exhibits a pattern indicative of genes that buffer ammonium toxicity based on the functional requirement for maintaining fitness in CSH but not HL media. While some genes in the cluster have putative relationships between their known functions, others may point to novel molecular activity or new connections between known activities [27,60] (see also Supplemental Data Files 1 and 2).

### 3.4. Resolving Drug-Media Interaction by Q-HTCP across Drug-Gradients

**Figure 4:**
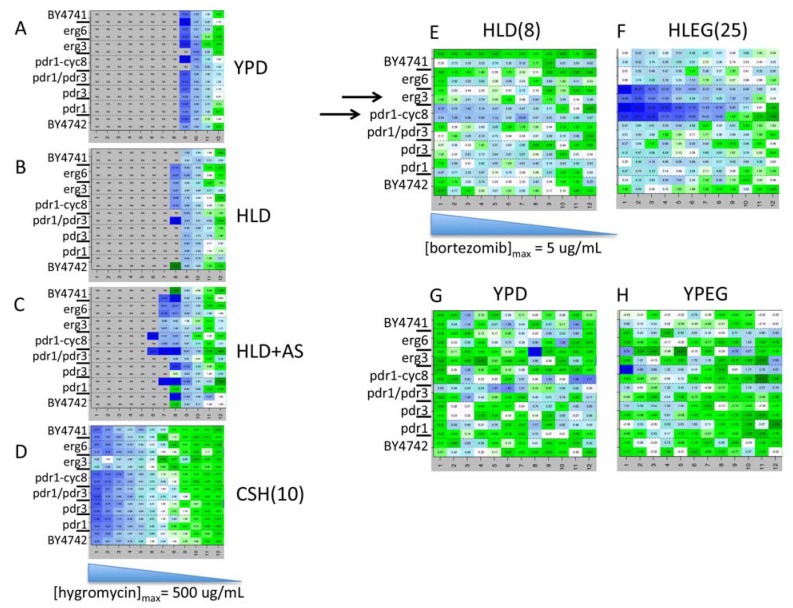
Drug sensitivity can depend upon media type, cellular efflux and permeability of drugs, or interaction between media and efflux or permeability. The experimental description is given in the text. The blue color scale indicates decreased fitness (increased L), with the range (hours) indicated in parentheses beside media type labels where relevant (panels E and F). Numerical interaction values are printed in each cell. Gray color indicates that no growth curve was observed (complete growth inhibition at the particular drug concentration). The cell array data was rearranged in the heat maps to assist visualization (rows 1/9, 2/10…8/16 were grouped). (**A**–**D**) Gradient plates were poured with 500 μg/mL hygromycin in (**A**) YPD, (**B**) HLD, (**C**) HLD+AS, and (**D**) CSHD; (**E**–**H**) Gradient plates were poured with 5 µg/mL bortezomib in (**E**) HLD, (**F**) HLEG, (**G**) YPD, and (**H**) YPEG.

The YKO/KD collection is frequently used to determine gene-drug interaction profiles. After finding an abundance of gene X media interaction (Figure 3), we investigated media-dependence of drug responses. Q-HTCP was performed on different media containing the same drug concentration gradients. Desired concentrations of drug were added to 10 mL of media and poured into a monowell plate that was tipped at an angle (by overlapping the bottom plate on its lid by about an inch) in order for the media to solidify as a wedge at one end. Next, the plate was laid flat and 30 mL of media without drug was layered over the wedge, creating a diffusion gradient. 384-cell arrays (16 × 24), consisting of 8 different strains, with 24-cultures per row, and strains arrayed in rows 1–8 repeated in rows 9–16 (to control for evenness in the gradient), were printed onto the drug gradient plates and control media without drug. Q-HTCP was performed and the growth curve parameter array of the control (no drug) was subtracted from the drug gradient plate. The change in L across the gradient was compared between different media (Figure 4). Eight strains harboring different mutants altering drug efflux (*PDR* mutants) or permeability (*ERG* mutants) were tested to investigate whether these factors influence the phenotype associated with particular compounds.

Media and genetic background dependence of growth phenotypes were observed for some compounds, including hygromycin and bortezomib **(Figure 4)**. Hygromycin selection [75] was strong in YPD (Figure 4A) and HL media (Figure 4B), but not on standard CSH media (Figure 4D). The addition of 0.5 mg/mL ammonium sulfate to HLD media (1/10 of what is added to CSH) slightly reduced growth inhibition by hygromycin (Figure 4C), suggesting it reduces hygromycin efficacy. Results for hygromycin are reminiscent of reduced efficacy of G418 with ammonium sulfate [19].

Bortezomib (Velcade, Millennium Pharmaceuticals, Cambridge, MA, USA) was approved in 2003 for myeloma treatment, and is thought to inhibit the proteasome by binding to the 26S subunit, although its anti-cancer effect is not fully understood. Bortezomib exerted little or no growth inhibitory effect on the reference strain (BY4741) in YPD. When a drug does not inhibit growth, it could be due to low concentration, lack of permeability, extrusion by the efficient yeast drug efflux (pleiotropic drug resistance, PDR) system, absence of the target, failure of the drug to sufficiently inactivate the target, the target being unrequired under the growth conditions, and/or buffering of the physiological effect of the drug. To assess the possibility of a drug efflux or permeability mechanism, the panel of strains, including mutants in ergosterol biosynthesis, pleotropic drug resistance, and a chimeric fusion of the PDR1 DNA-binding domain with the transcriptional repressor domain of CYC8 (Pdr1-Cyc8) [76], were tested with a concentration gradient of 13 µM bortezomib in YPD, YPEG, HLD, and HLEG (Figure 4E–H). Bortezomib was found to interact with media, drug efflux, and permeability. This result confirms a previous report that drug efflux function influences sensitivity to bortezomib [77], and we found further enhancement of sensitivity with HL media and ethanol/glycerol as the carbon source. Not much of a growth phenotype was observed on YPEG (Figure 4E–H), suggesting the combination of carbon and nitrogen sources influence drug responsiveness. The drug efflux mutants we tested were different from those previously examined [77], and we found that a chimeric protein constructed by Stepanov* et al.* was effective for sensitizing to bortezomib [76]. *ERG3* deletion also influenced growth inhibition by bortezomib on HLEG media, presumably by affecting membrane permeability.

In summary, gradient experiments assist optimization of drug screening conditions by surveying a wide range of concentration in multiple different media and with multiple strains. Identification of a media type that confers enhanced sensitivity can reduce the drug cost for a genomic screen. Likewise, drug efflux or permeability mutations could be introduced into the YKO/KD libraries if needed [19,20], as in the case of bortezomib. Furthermore, gradient plate Q-HTCP can be used to quantify known gene-drug interactions (e.g., target or buffering genes) to help optimize a range of concentrations for a phenomic screen [16]. The identification of drug X media interaction (e.g., differential drug sensitivity of the wild type genetic background on fermentable *vs.* non-fermentable media) may point to comparative phenomic screens to discover differential gene interaction networks informative about the impact of biological context (e.g., Warburg effect) on drug resistance networks.

## 4. Discussion

Can Yeast Phenomic Models Aid Construction of Quantitative Genetic Networks that Predict Disease?

The genomic collection of *S. cerevisiae* yeast knockout and knockdown mutants has been a boon for surveying genotype-phenotype complexity [21]. Prior to construction of the YKO/KD library, the vastness of gene interaction space was unappreciated. Q-HTCP was developed as a cell array-based growth curve technology so that the large number of mutant phenotypes can be better resolved and gene interaction more precisely quantified, which is complementary to higher throughput, less quantitative methods. The ability to explore gene interaction in greater detail, with increased sensitivity and specificity comes with the constraint of more focused sets of questions and/or models, but may lead to better understanding of complex gene networks in the context of specific disease models [10].

Most high-throughput yeast phenotyping methods measure fitness endpoints [30]. In contrast, Q-HTCP obtains kinetic growth, likened to OD of liquid culture, but with much greater throughput, so that fitness can be further analyzed in terms of distinct components of fitness including carrying capacity, maximum specific rate, and time to reach half-carrying capacity [37]. For example, carrying capacity (final growth density) of cultures on glucose is affected by the respiratory function of yeast, because inefficient respiration results in reduced biomass accumulation following the diauxic shift (*i.e.*, during growth without glycolysis), but may not affect other logistic growth parameters. By contrast, YKO/KD strains may exhibit differences in lag time if they carry mutations disrupting functions that influence efflux of the growth inhibitory compound. This phenotype would be captured by the time to half-carrying capacity (the L parameter) even if the carrying capacity or the maximum specific rate were unaffected [10]. Specific classes of mutants could be detected by considering the interaction pattern across all parameters: for example cell cycle checkpoint mutants might be identified by a short L with an accelerated maximum specific rate (due to checkpoint failure) and a reduced carrying capacity (due to cell death) [78,79]. These are just a few examples of the overall theme, which is that genetic buffering of fitness can be further resolved with Q-HTCP to increase the resolution of gene interaction networks, both quantitatively and in terms of different CPPs.

**Figure 5:**
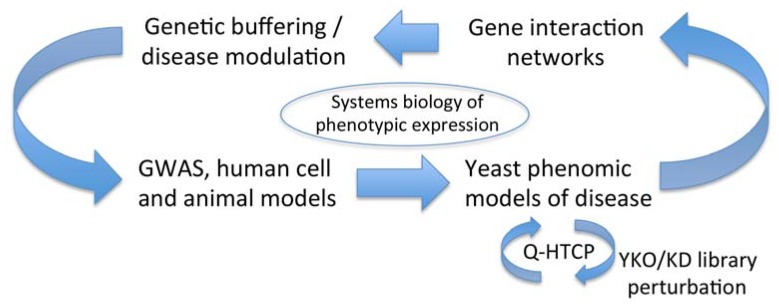
Yeast phenomic models can serve to discover gene interaction networks underlying expression of disease phenotypes. Disease-buffering gene interaction networks are derived experimentally by disease-relevant perturbation of the yeast gene knockout/knockdown mutant library followed by measurement of gene interaction with Q-HTCP. The resulting gene networks are queried for evolutionary conservation to generate hypotheses regarding their relevance to human phenotypes. Hypotheses can be tested in translational models, leading to model validation or refinement, with iterative yeast phenomic analysis for further refinement.

Aspects of Q-HTCP development in our laboratory currently include: (1) the use of commercial liquid handling robotics for cell array printing; (2) development of imaging methods and image analysis software to convert images to growth curves [16]; (3) use of a logistic growth model to fit Q-HTCP data so that CPPs can be used for quantifying gene interaction (Figure 1); (4) development of approaches to optimize screening conditions for phenomic studies, such as media modification (Figure 2 and Figure 3) and gradient array analysis (Figure 4); (5) incorporation of the synthetic genetic array method to carry out phenomic screens of gene x gene interaction [20]; and (6), development of data mining tools including REMc [12]. Future development of the technology will focus on its application to additional disease models and integration of yeast gene interaction networks with other omic data to more fully understand disease expression (Figure 5).

We suspect human genotype-phenotype complexity originates substantially from cellular processes shared in common with yeast. Thus yeast phenomic models of human disease could inform the basic biology of gene interaction networks at the same time as variable disease expression, functioning for both “basic” science [80] and “translational” insight intended for validation in higher eukaryotes [50,81]. The experimental convenience and eukaryotic relevance of the YKO/KD collections, together with the development and application of Q-HTCP techniques provide a powerful opportunity to discover gene interaction networks underlying disease biology.

## 5. Conclusions

Work with the YKO/KD library has revealed eukaryotic gene interaction networks to be extensive, revealing vast phenotypic complexity even in a single-cell organism. Here we report, even when cell proliferation of the reference strain is similar between different media, hundreds of the YKO/KD strains have growth phenotypes, thus revealing extensive gene X media interaction. The fact that animals have additional organismal complexity is perhaps the strongest argument for using *S. cerevisiae* to model gene interaction networks [82]. Cellular functions and the genes carrying them out are conserved from yeast to human [83]. Thus gene interactions that further modulate these processes may also be usefully modeled across species. Many technical and biological factors will impact the implementation and translational success of any particular yeast phenomic disease model. Cell culture media is one such factor and the use of HL yeast media may increase positive predictive value for disease translation in some human cell models. In any case, awareness of the potential for gene x media interaction is likely to be beneficial. A challenge not addressed in this article, but critical to the success of yeast phenomics for modeling disease-relevant gene interaction networks, is development of computational methods that leverage phenomic and other omic data to extract biological insight [84]. As yeast phenomics and other gene interaction techniques to study human disease advance, principles for genetic buffering in humans will emerge and give rise to iterative models employing human data and yeast experiments [3]. An innovative approach in this regard is the Resilience Project, which seeks to identify, from within the non-diseased human populations, buffering loci that harbor the capacity to reduce phenotypic manifestations of disease-associated mutations [85]. The development of a workflow to identify buffering networks in people could leverage yeast phenomic models to help mine for gene interaction networks underlying disease expression in human populations. Such understanding will advance disease diagnosis and provide new targets for personalized/precision management of disease phenotypes.

## Supplementary Files

Supplementary File 1
