# Supplementary material for: Yeast Phenomics: An Experimental Approach for Modeling Gene Interaction Networks that Buffer Disease
Source: Genes (Basel). 2015 Feb 6;6(1):24–45. doi: 10.3390/genes6010024 (PMC4377832; doi:10.3390/genes6010024)
Supplement: Supplementary File 1 [file genes-06-00024-s001.zip › genes-63849-supplementary/Supplemental Data File 1.pdf]

Supplemental Data File 1: REMc and heatmaps for gene x media interaction screen.

Q-HTCP was performed and gene x media interaction quantified as described in methods. REMc clustering was performed with the cluster naming convention as described in reference 12. There were 56 YKO/KD cultures that did not grow on YPD (control/reference media), for which the respective genes were omitted from the analysis. In cases where a particular YKO/KD strain did not yield a growth curve on a test media but did grown on YPD, K values were set to -130 and L to 90, representing extreme loss of fitness. The CPP-derived interaction and shift values are contained in Supplemental Data File 2 along with REMc results and GO Term enrichment (see reference 71 for details on enrichment analysis).

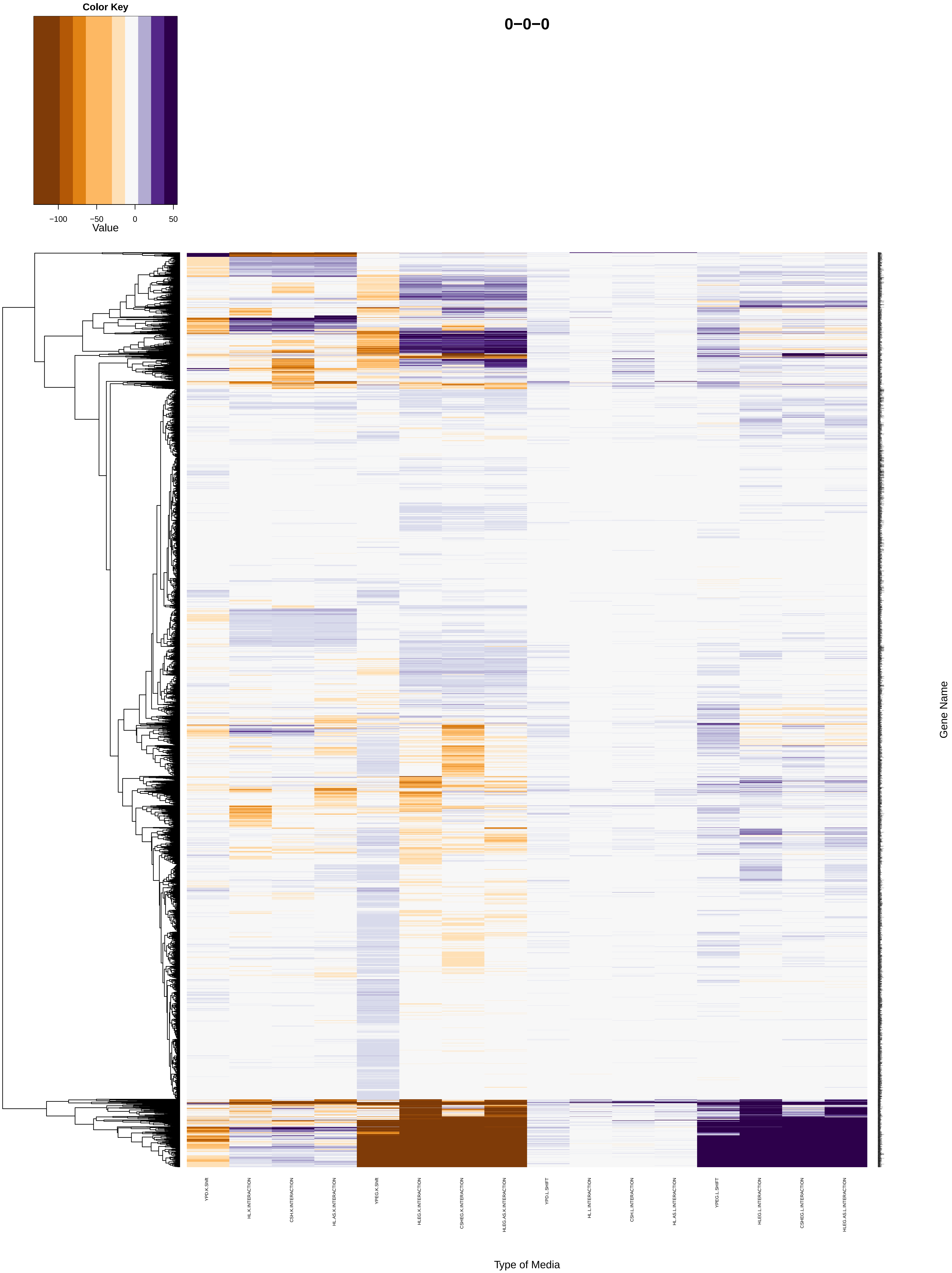

Color Key
−100
−50
0
50
Value
0−0−0
YPD.K.Shift
HL.K.INTERACTION
CSH.K.INTERACTION
HL.AS.K.INTERACTION
YPEG.K.Shift
HLEG.K.INTERACTION
CSHEG.K.INTERACTION
HLEG.AS.K.INTERACTION
YPD.L.SHIFT
HL.L.INTERACTION
CSH.L.INTERACTION
HL.AS.L.INTERACTION
YPEG.L.SHIFT
HLEG.L.INTERACTION
CSHEG.L.INTERACTION
HLEG.AS.L.INTERACTION
Type of Media
Gene Name

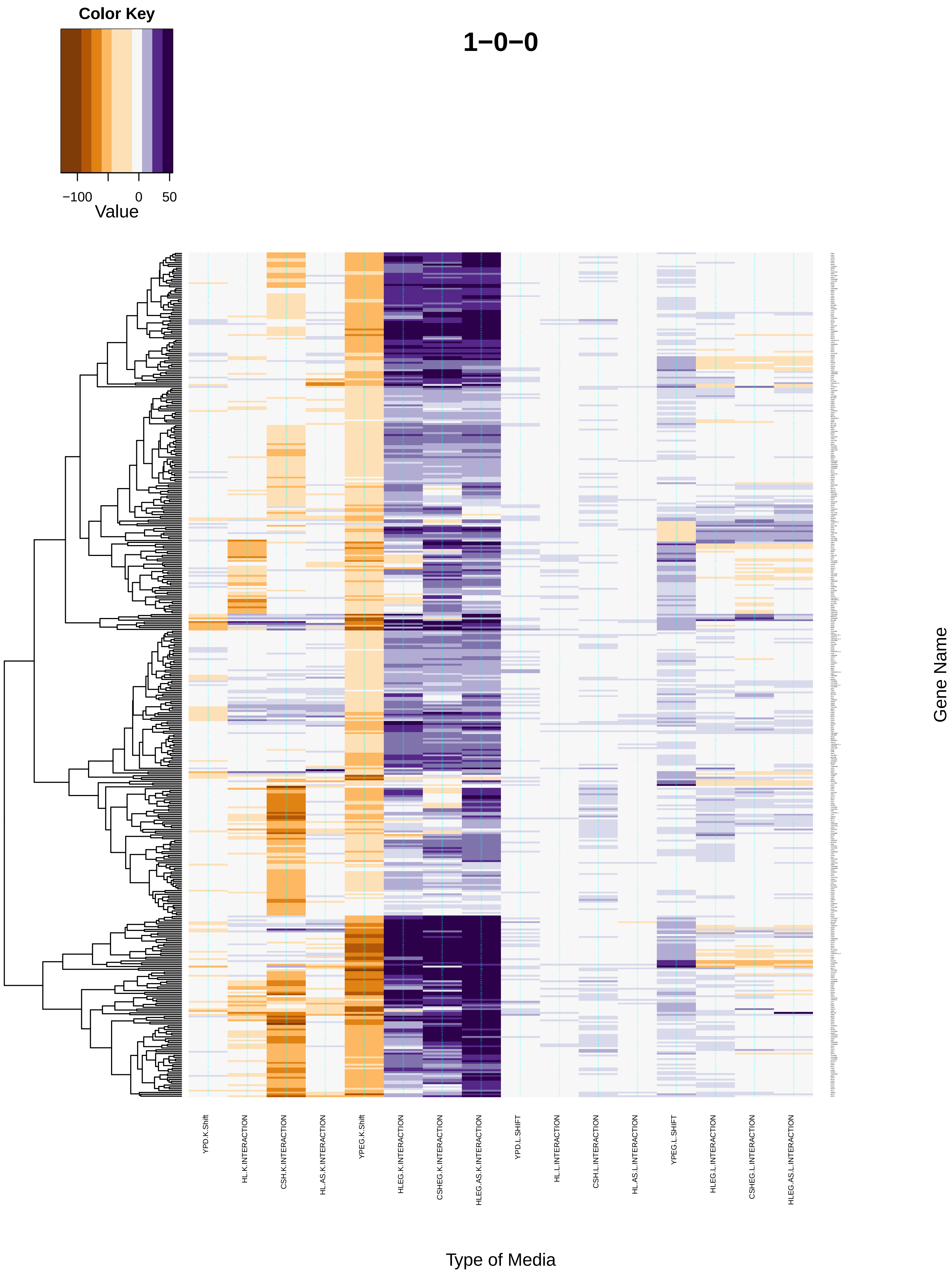

Color Key
−100
0
50
Value
1−0−0
Gene Name
YPD.K.Shift
HL.K.INTERACTION
CSH.K.INTERACTION
HL.AS.K.INTERACTION
YPEG.K.Shift
HLEG.K.INTERACTION
CSHEG.K.INTERACTION
HLEG.AS.K.INTERACTION
YPD.L.SHIFT
HL.L.INTERACTION
CSH.L.INTERACTION
HL.AS.L.INTERACTION
YPEG.L.SHIFT
HLEG.L.INTERACTION
CSHEG.L.INTERACTION
HLEG.AS.L.INTERACTION
Type of Media

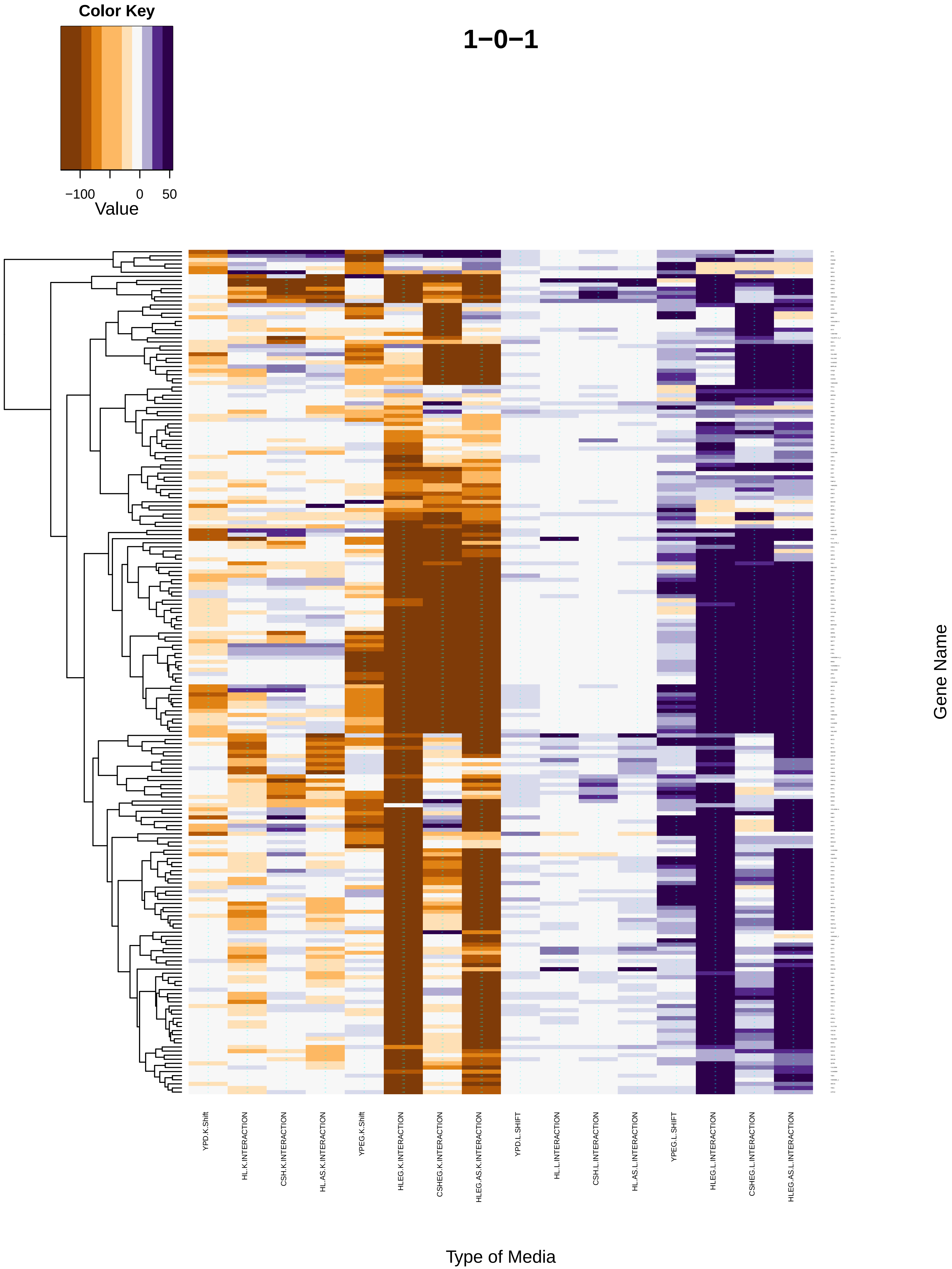
Color Key
−100
0
50
Value
1−0−1
YPD.K.Shift
HL.K.INTERACTION
CSH.K.INTERACTION
HL.AS.K.INTERACTION
YPEG.K.Shift
HLEG.K.INTERACTION
CSHEG.K.INTERACTION
HLEG.AS.K.INTERACTION
YPD.L.SHIFT
HL.L.INTERACTION
CSH.L.INTERACTION
HL.AS.L.INTERACTION
YPEG.L.SHIFT
HLEG.L.INTERACTION
CSHEG.L.INTERACTION
HLEG.AS.L.INTERACTION
Gene Name
Type of Media

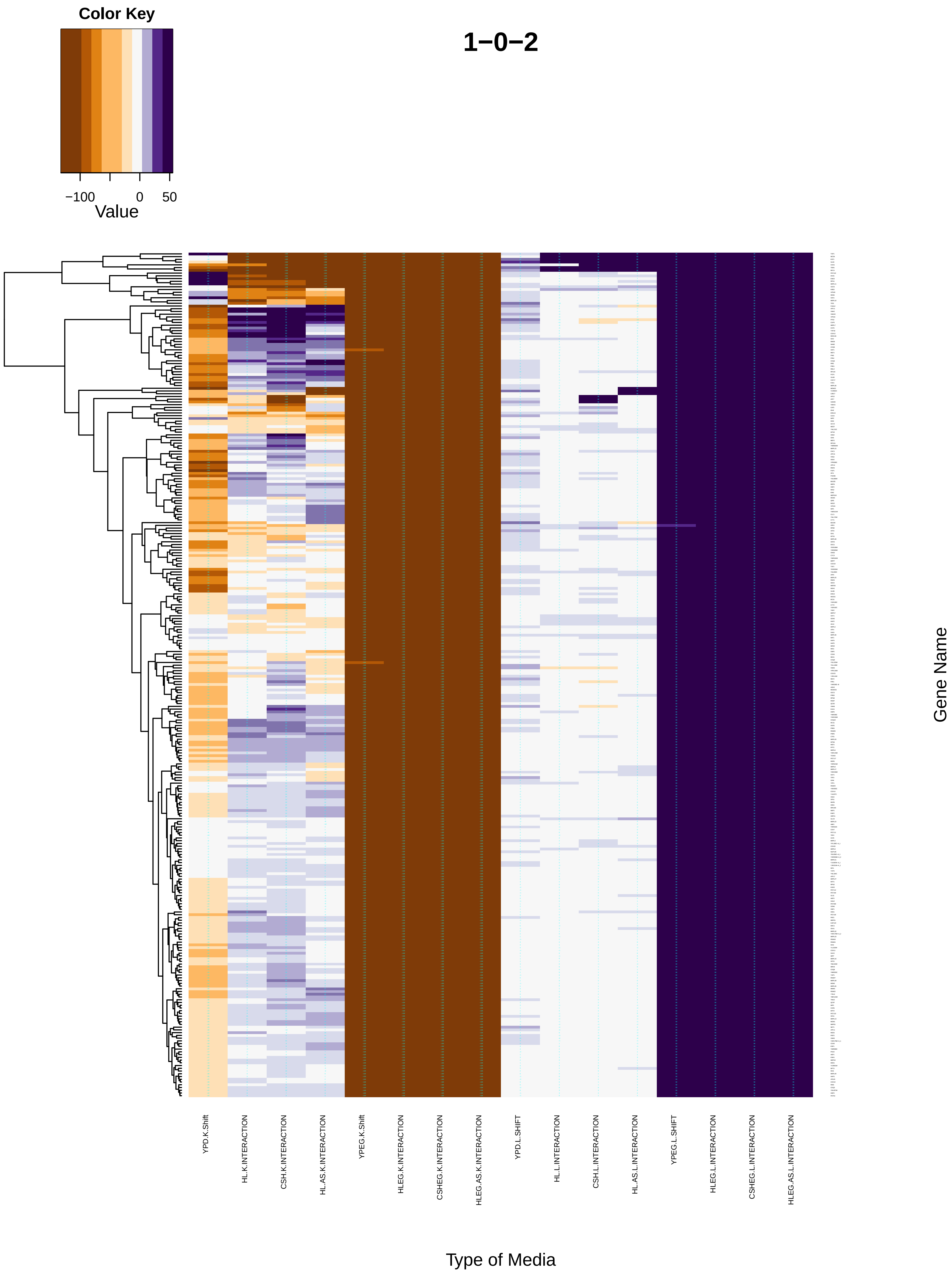
Color Key
−100
0
50
Value
1−0−2
YPD.K.Shift
HL.K.INTERACTION
CSH.K.INTERACTION
HL.AS.K.INTERACTION
YPEG.K.Shift
HLEG.K.INTERACTION
CSHEG.K.INTERACTION
HLEG.AS.K.INTERACTION
YPD.L.SHIFT
HL.L.INTERACTION
CSH.L.INTERACTION
HL.AS.L.INTERACTION
YPEG.L.SHIFT
HLEG.L.INTERACTION
CSHEG.L.INTERACTION
HLEG.AS.L.INTERACTION
Type of Media
Gene Name

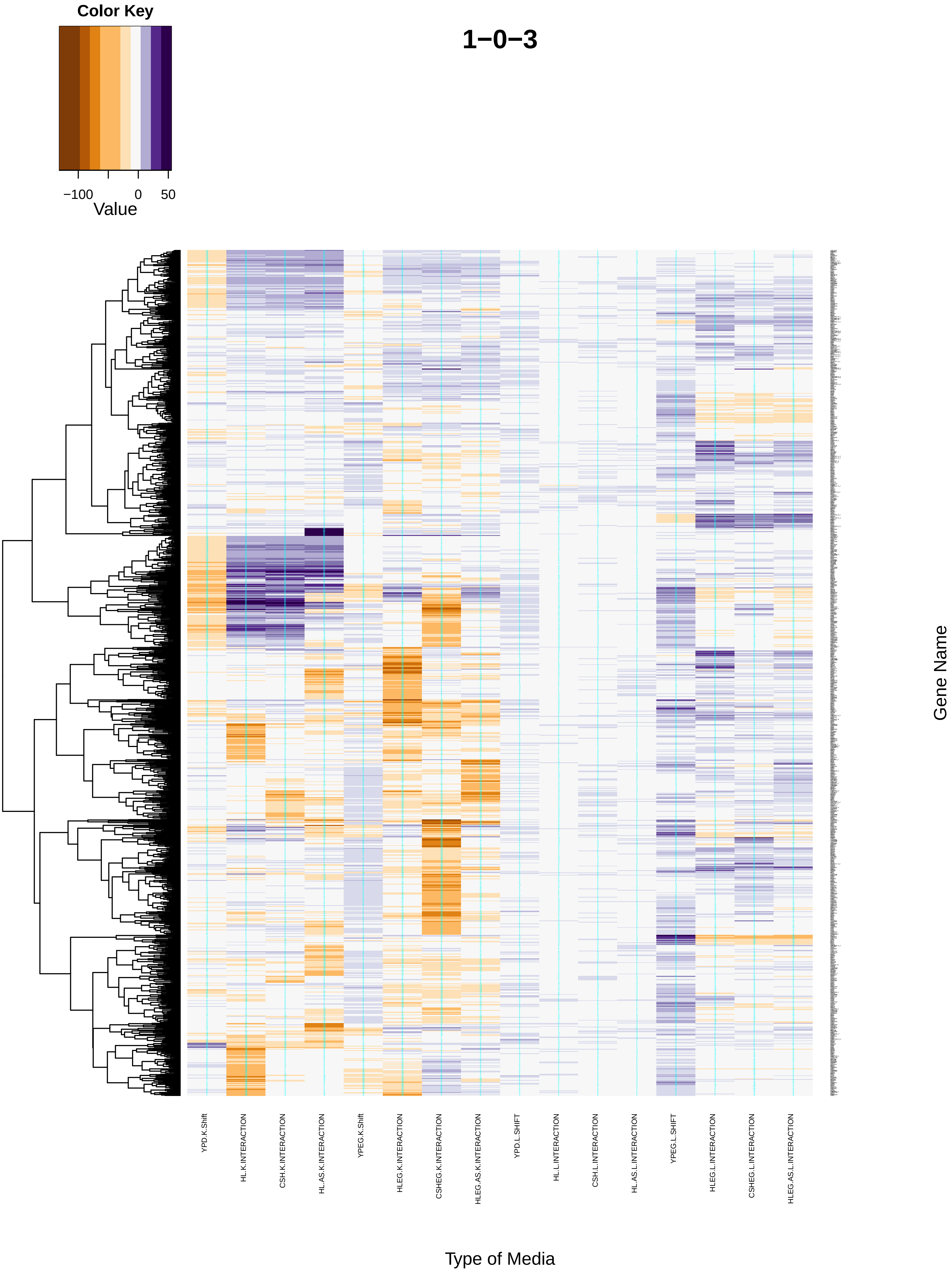

Color Key
−100
0
50
Value
1−0−3
YPD.K.Shift
HL.K.INTERACTION
CSH.K.INTERACTION
HL.AS.K.INTERACTION
YPEG.K.Shift
HLEG.K.INTERACTION
CSHEG.K.INTERACTION
HLEG.AS.K.INTERACTION
YPD.L.SHIFT
HL.L.INTERACTION
CSH.L.INTERACTION
HL.AS.L.INTERACTION
YPEG.L.SHIFT
HLEG.L.INTERACTION
CSHEG.L.INTERACTION
HLEG.AS.L.INTERACTION
Gene Name
Type of Media

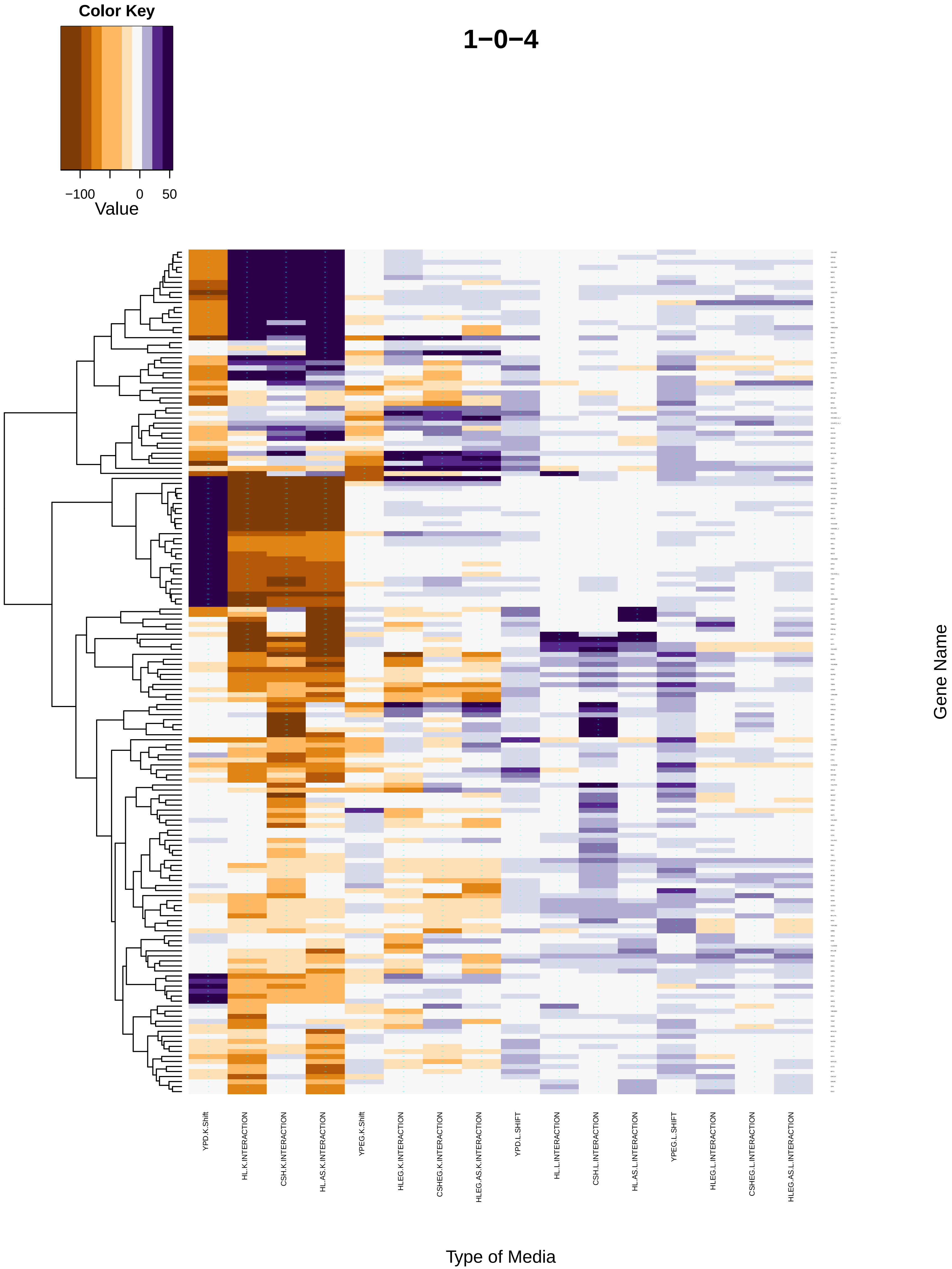
Color Key
−100
0
50
Value
1−0−4
YPD.K.Shift
HL.K.INTERACTION
CSH.K.INTERACTION
HL.AS.K.INTERACTION
YPEG.K.Shift
HLEG.K.INTERACTION
CSHEG.K.INTERACTION
HLEG.AS.K.INTERACTION
YPD.L.SHIFT
HL.L.INTERACTION
CSH.L.INTERACTION
HL.AS.L.INTERACTION
YPEG.L.SHIFT
HLEG.L.INTERACTION
CSHEG.L.INTERACTION
HLEG.AS.L.INTERACTION
Type of Media
Gene Name

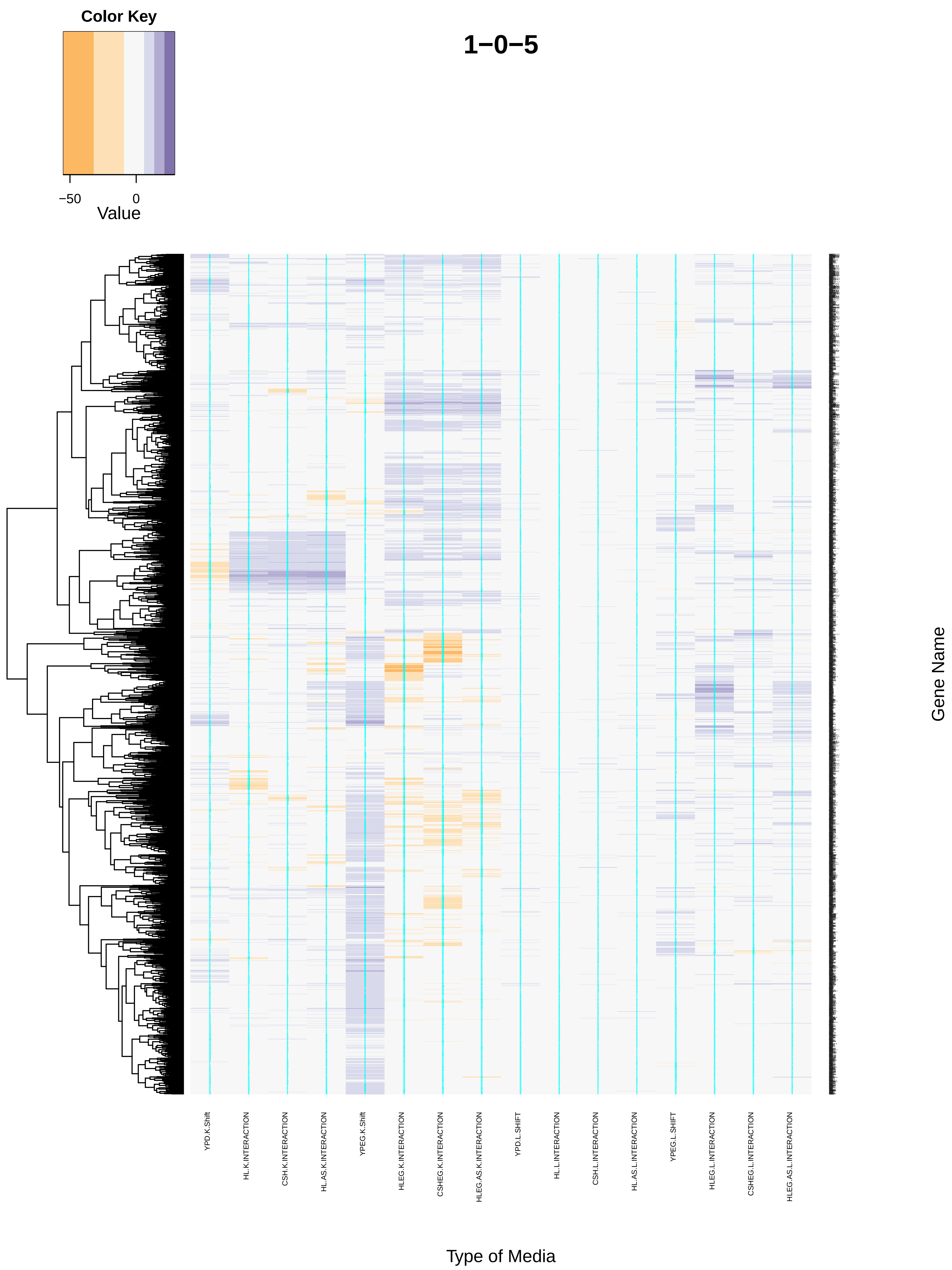
1−0−5
Color Key
−50
0
Value
YPD.K.Shift
HL.K.INTERACTION
CSH.K.INTERACTION
HL.AS.K.INTERACTION
YPEG.K.Shift
HLEG.K.INTERACTION
CSHEG.K.INTERACTION
HLEG.AS.K.INTERACTION
YPD.L.SHIFT
HL.L.INTERACTION
CSH.L.INTERACTION
HL.AS.L.INTERACTION
YPEG.L.SHIFT
HLEG.L.INTERACTION
CSHEG.L.INTERACTION
HLEG.AS.L.INTERACTION
Gene Name
Type of Media

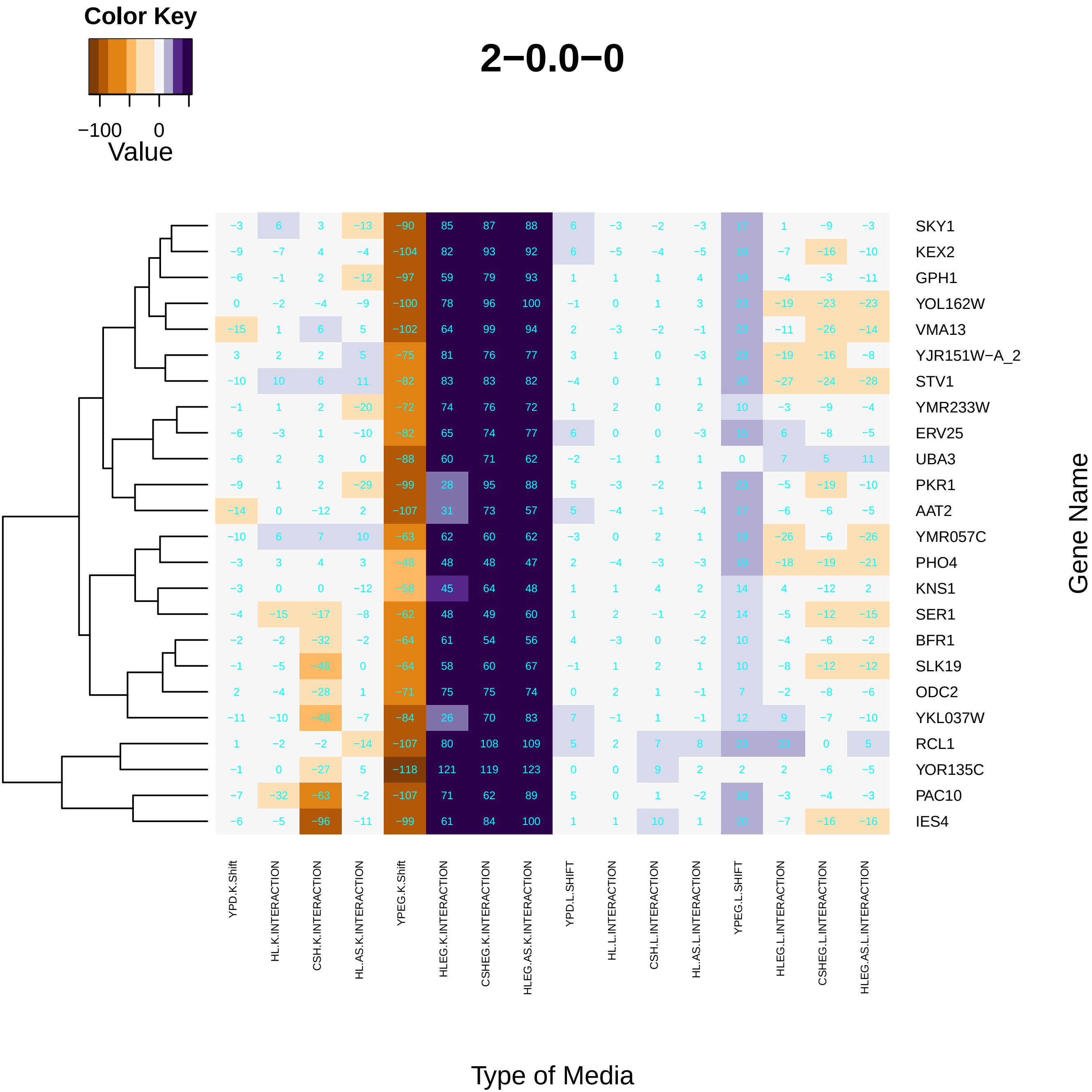

Color Key
−100 0
Value
2−0.0−0
YPD.K.Shift HL.K.INTERACTION CSH.K.INTERACTION HL.AS.K.INTERACTION YPEG.K.Shift HLEG.K.INTERACTION CSHEG.K.INTERACTION HLEG.AS.K.INTERACTION YPD.L.SHIFT HL.L.INTERACTION CSH.L.INTERACTION HL.AS.L.INTERACTION YPEG.L.SHIFT HLEG.L.INTERACTION CSHEG.L.INTERACTION HLEG.AS.L.INTERACTION
SKY1 −3 6 3 −13 −90 85 87 88 6 −3 −2 −3 17 1 −9 −3
KEX2 −9 −7 4 −4 −104 82 93 92 6 −5 −4 −5 19 −7 −16 −10
GPH1 −6 −1 2 −12 −97 59 79 93 1 1 1 4 19 −4 −3 −11
YOL162W 0 −2 −4 −9 −100 78 96 100 −1 0 1 3 23 −19 −23 −23
VMA13 −15 1 6 5 −102 64 99 94 2 −3 −2 −1 23 −11 −26 −14
YJR151W−A_2 3 2 2 5 −75 81 76 77 3 1 0 −3 23 −19 −16 −8
STV1 −10 10 6 11 −82 83 83 82 −4 0 1 1 20 −27 −24 −28
YMR233W −1 1 2 −20 −72 74 76 72 1 2 0 2 10 −3 −9 −4
ERV25 −6 −3 1 −10 −82 65 74 77 6 0 0 −3 15 6 −8 −5
UBA3 −6 2 3 0 −88 60 71 62 −2 −1 1 1 0 7 5 11
PKR1 −9 1 2 −29 −99 28 95 88 5 −3 −2 1 23 −5 −19 −10
AAT2 −14 0 −12 2 −107 31 73 57 5 −4 −1 −4 17 −6 −6 −5
YMR057C −10 6 7 10 −63 62 60 62 −3 0 2 1 19 −26 −6 −26
PHO4 −3 3 4 3 −48 48 48 47 2 −4 −3 −3 19 −18 −19 −21
KNS1 −3 0 0 −12 −58 45 64 48 1 1 4 2 14 4 −12 2
SER1 −4 −15 −17 −8 −62 48 49 60 1 2 −1 −2 14 −5 −12 −15
BFR1 −2 −2 −32 −2 −64 61 54 56 4 −3 0 −2 10 −4 −6 −2
SLK19 −1 −5 −46 0 −64 58 60 67 −1 1 2 1 10 −8 −12 −12
ODC2 2 −4 −28 1 −71 75 75 74 0 2 1 −1 7 −2 −8 −6
YKL037W −11 −10 −48 −7 −84 26 70 83 7 −1 1 −1 12 9 −7 −10
RCL1 1 −2 −2 −14 −107 80 108 109 5 2 7 8 23 23 0 5
YOR135C −1 0 −27 5 −118 121 119 123 0 0 9 2 2 2 −6 −5
PAC10 −7 −32 −63 −2 −107 71 62 89 5 0 1 −2 18 −3 −4 −3
IES4 −6 −5 −96 −11 −99 61 84 100 1 1 10 1 20 −7 −16 −16
Gene Name
Type of Media

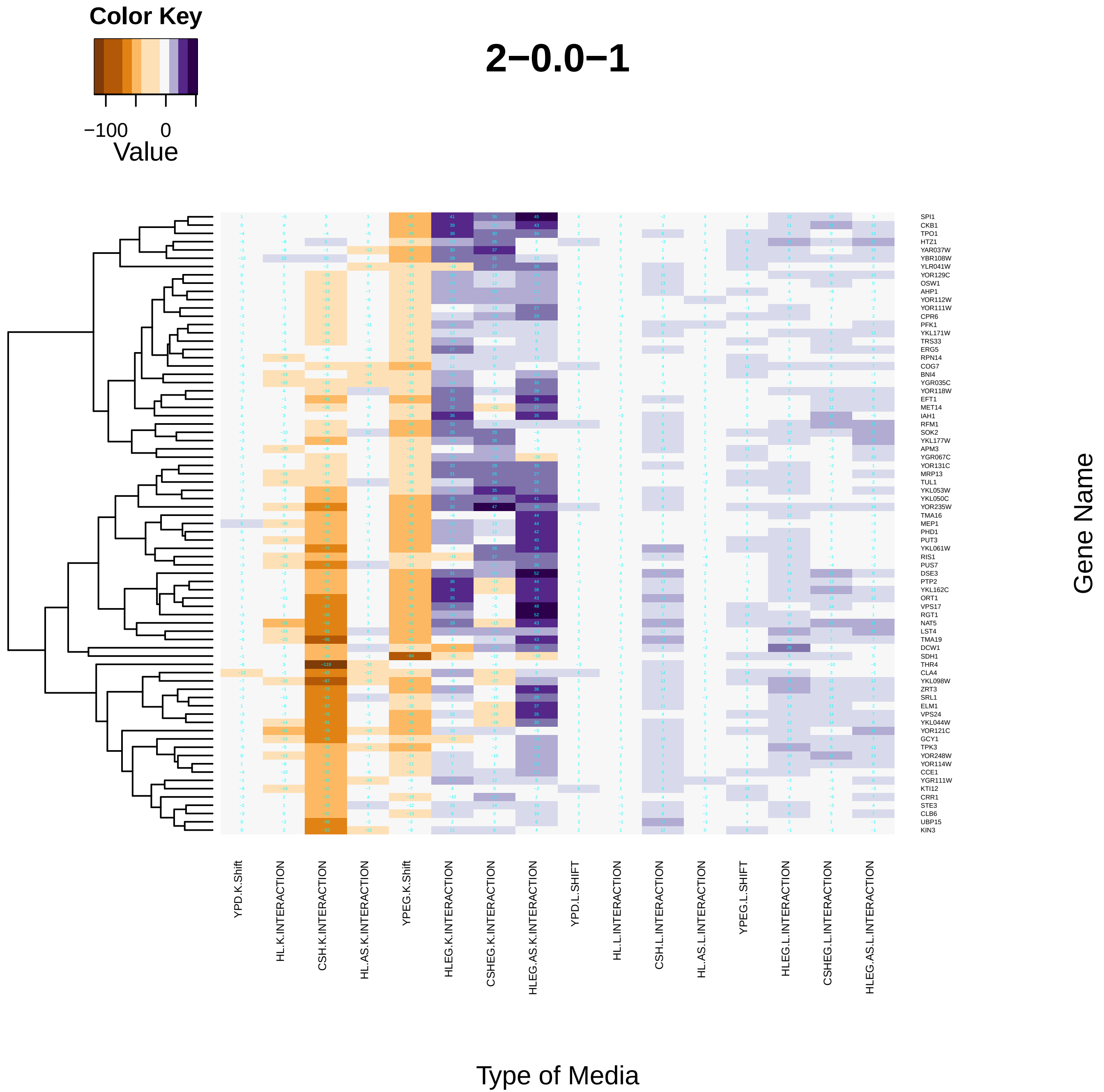

Color Key
−100
0
Value
2−0.0−1
SPI1
CKB1
TPO1
HTZ1
YAR037W
YBR108W
YLR041W
YOR129C
OSW1
AHP1
YOR112W
YOR111W
CPR6
PFK1
YKL171W
TRS33
ERG5
RPN14
COG7
BNI4
YGR035C
YOR118W
EFT1
MET14
IAH1
RFM1
SOK2
YKL177W
APM3
YGR067C
YOR131C
MRP13
TUL1
YKL053W
YKL050C
YOR235W
TMA16
MEP1
PHD1
PUT3
YKL061W
RIS1
PUS7
DSE3
PTP2
YKL162C
ORT1
VPS17
RGT1
NAT5
LST4
TMA19
DCW1
SDH1
THR4
CLA4
YKL098W
ZRT3
SRL1
ELM1
VPS24
YKL044W
YOR121C
GCY1
TPK3
YOR248W
YOR114W
CCE1
YGR111W
KTI12
CRR1
STE3
CLB6
UBP15
KIN3
Gene Name
YPD.K.Shift
HL.K.INTERACTION
CSH.K.INTERACTION
HL.AS.K.INTERACTION
YPEG.K.Shift
HLEG.K.INTERACTION
CSHEG.K.INTERACTION
HLEG.AS.K.INTERACTION
YPD.L.SHIFT
HL.L.INTERACTION
CSH.L.INTERACTION
HL.AS.L.INTERACTION
YPEG.L.SHIFT
HLEG.L.INTERACTION
CSHEG.L.INTERACTION
HLEG.AS.L.INTERACTION
Type of Media

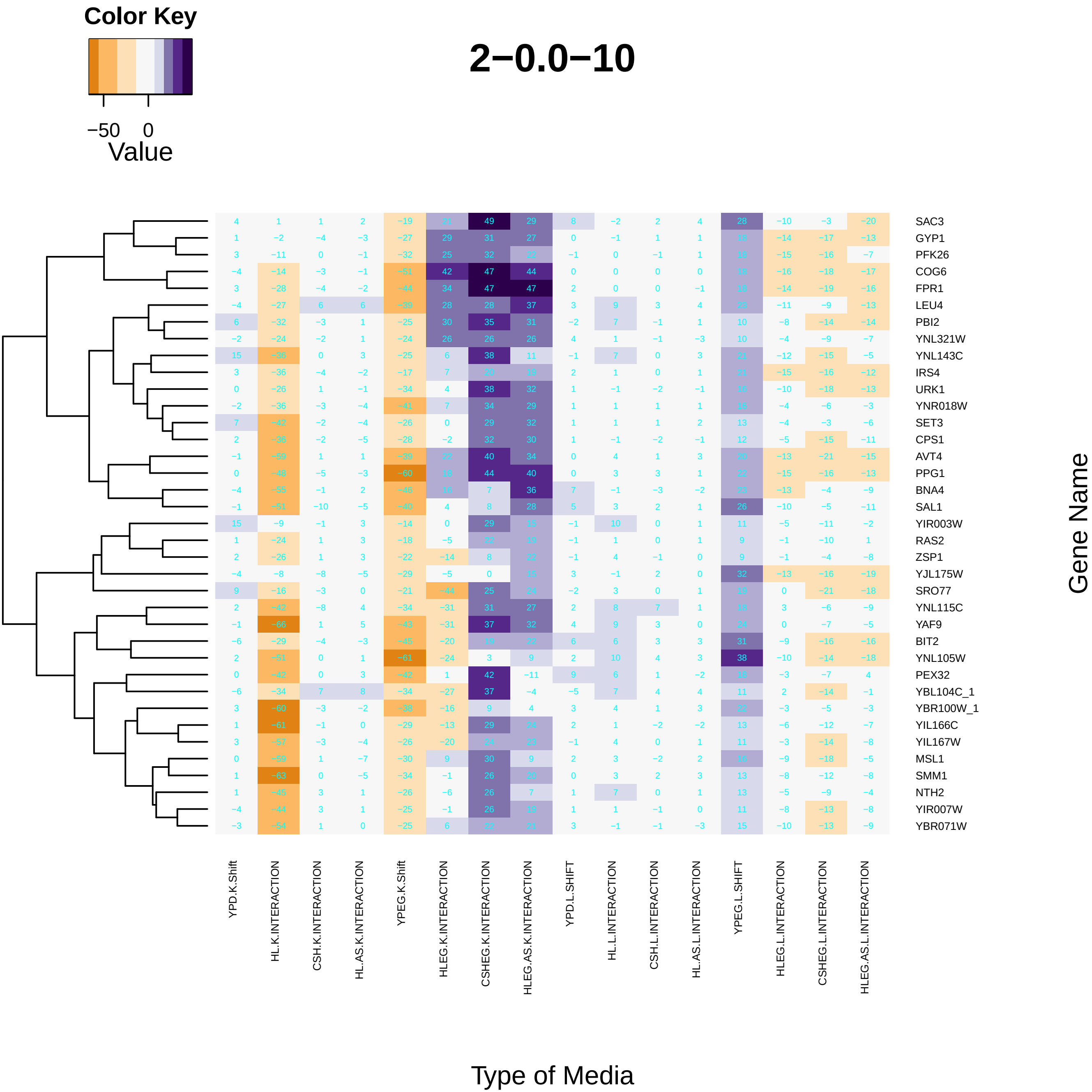
Color Key
−50 0
Value
2−0.0−10
SAC3
GYP1
PFK26
COG6
FPR1
LEU4
PBI2
YNL321W
YNL143C
IRS4
URK1
YNR018W
SET3
CPS1
AVT4
PPG1
BNA4
SAL1
YIR003W
RAS2
ZSP1
YJL175W
SRO77
YNL115C
YAF9
BIT2
YNL105W
PEX32
YBL104C_1
YBR100W_1
YIL166C
YIL167W
MSL1
SMM1
NTH2
YIR007W
YBR071W
Gene Name
YPD.K.Shift
HL.K.INTERACTION
CSH.K.INTERACTION
HL.AS.K.INTERACTION
YPEG.K.Shift
HLEG.K.INTERACTION
CSHEG.K.INTERACTION
HLEG.AS.K.INTERACTION
YPD.L.SHIFT
HL.L.INTERACTION
CSH.L.INTERACTION
HL.AS.L.INTERACTION
YPEG.L.SHIFT
HLEG.L.INTERACTION
CSHEG.L.INTERACTION
HLEG.AS.L.INTERACTION
Type of Media

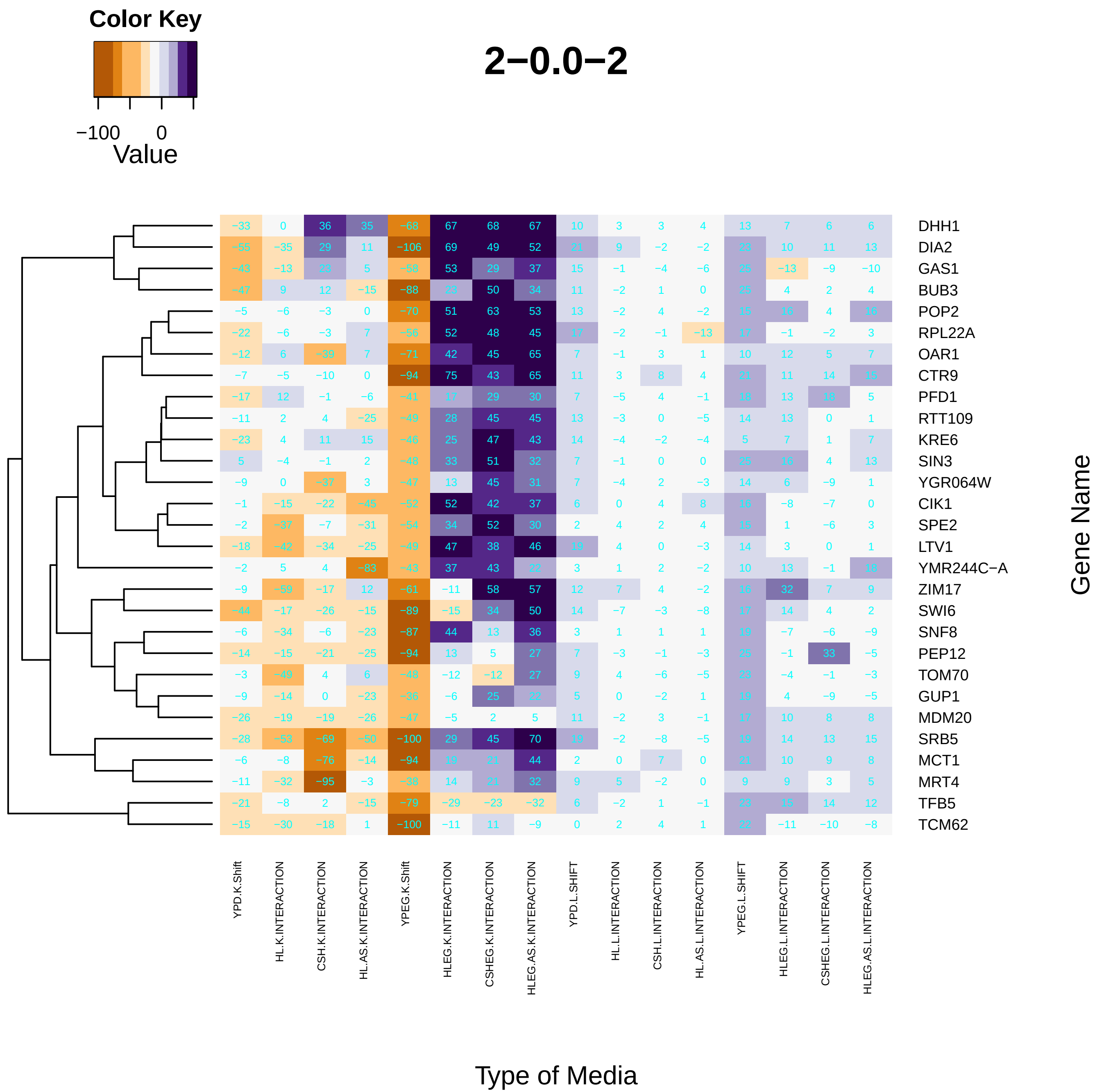
2−0.0−2
Color Key
−100
0
Value
DHH1
DIA2
GAS1
BUB3
POP2
RPL22A
OAR1
CTR9
PFD1
RTT109
KRE6
SIN3
YGR064W
CIK1
SPE2
LTV1
YMR244C−A
ZIM17
SWI6
SNF8
PEP12
TOM70
GUP1
MDM20
SRB5
MCT1
MRT4
TFB5
TCM62
Gene Name
YPD.K.Shift
HL.K.INTERACTION
CSH.K.INTERACTION
HL.AS.K.INTERACTION
YPEG.K.Shift
HLEG.K.INTERACTION
CSHEG.K.INTERACTION
HLEG.AS.K.INTERACTION
YPD.L.SHIFT
HL.L.INTERACTION
CSH.L.INTERACTION
HL.AS.L.INTERACTION
YPEG.L.SHIFT
HLEG.L.INTERACTION
CSHEG.L.INTERACTION
HLEG.AS.L.INTERACTION
Type of Media

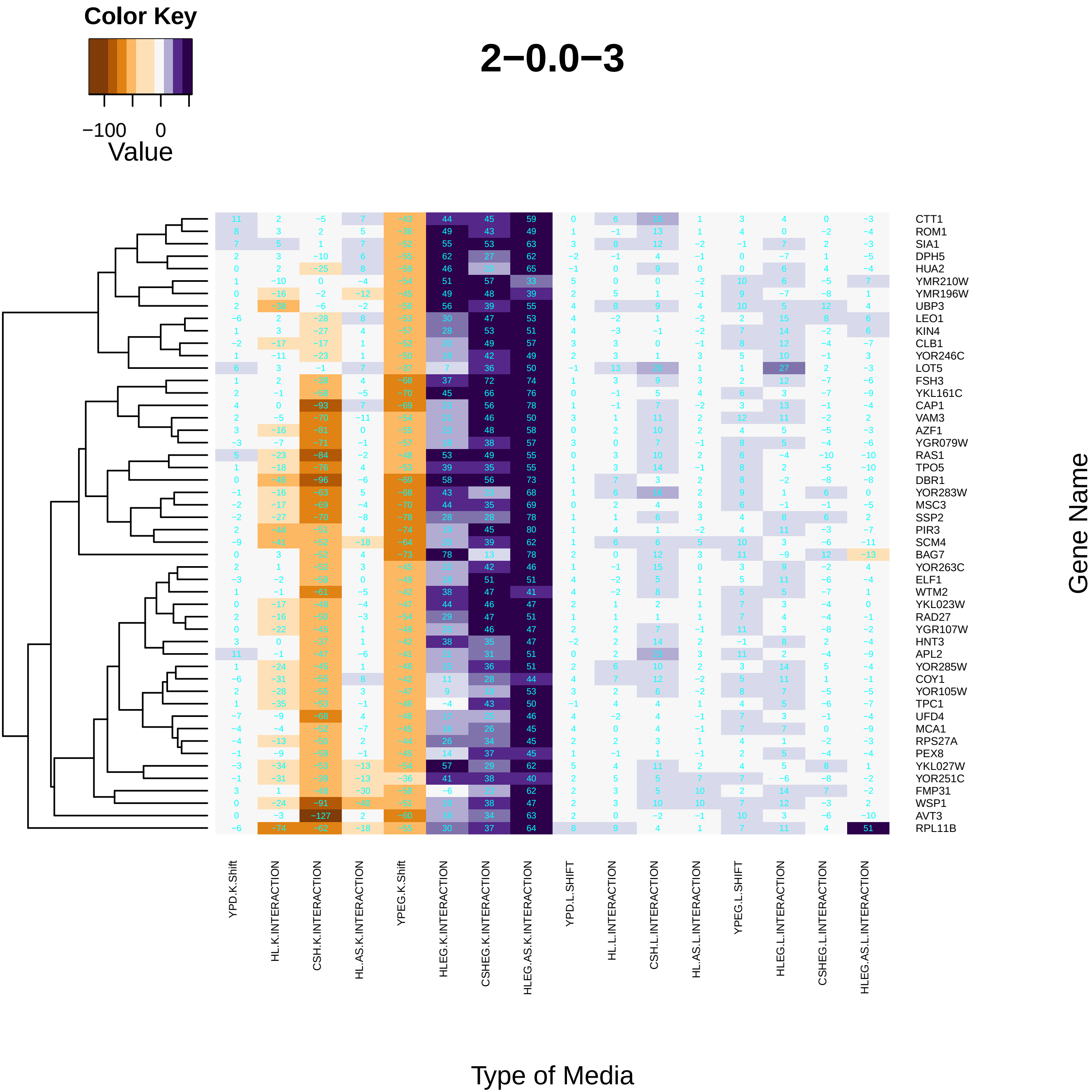

Color Key
−100
0
Value
2−0.0−3
CTT1
ROM1
SIA1
DPH5
HUA2
YMR210W
YMR196W
UBP3
LEO1
KIN4
CLB1
YOR246C
LOT5
FSH3
YKL161C
CAP1
VAM3
AZF1
YGR079W
RAS1
TPO5
DBR1
YOR283W
MSC3
SSP2
PIR3
SCM4
BAG7
YOR263C
ELF1
WTM2
YKL023W
RAD27
YGR107W
HNT3
APL2
YOR285W
COY1
YOR105W
TPC1
UFD4
MCA1
RPS27A
PEX8
YKL027W
YOR251C
FMP31
WSP1
AVT3
RPL11B
Gene Name
YPD.K.Shift
HL.K.INTERACTION
CSH.K.INTERACTION
HL.AS.K.INTERACTION
YPEG.K.Shift
HLEG.K.INTERACTION
CSHEG.K.INTERACTION
HLEG.AS.K.INTERACTION
YPD.L.SHIFT
HL.L.INTERACTION
CSH.L.INTERACTION
HL.AS.L.INTERACTION
YPEG.L.SHIFT
HLEG.L.INTERACTION
CSHEG.L.INTERACTION
HLEG.AS.L.INTERACTION
Type of Media

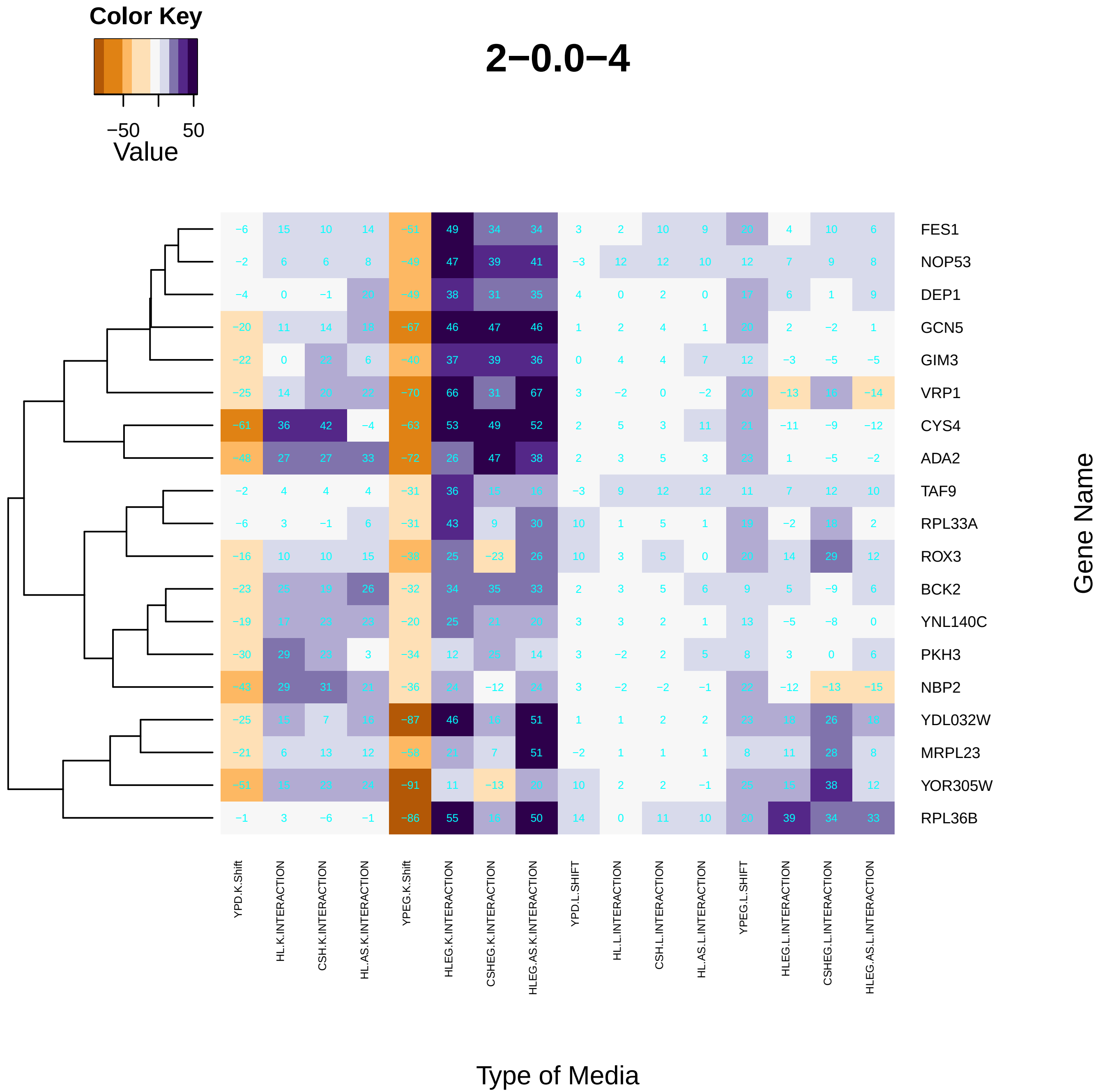
Color Key
−50
50
Value
2−0.0−4
FES1 −6 15 10 14 −51 49 34 34 3 2 10 9 20 4 10 6
NOP53 −2 6 6 8 −49 47 39 41 −3 12 12 10 12 7 9 8
DEP1 −4 0 −1 20 −49 38 31 35 4 0 2 0 17 6 1 9
GCN5 −20 11 14 18 −67 46 47 46 1 2 4 1 20 2 −2 1
GIM3 −22 0 22 6 −40 37 39 36 0 4 4 7 12 −3 −5 −5
VRP1 −25 14 20 22 −70 66 31 67 3 −2 0 −2 20 −13 16 −14
CYS4 −61 36 42 −4 −63 53 49 52 2 5 3 11 21 −11 −9 −12
ADA2 −48 27 27 33 −72 26 47 38 2 3 5 3 23 1 −5 −2
TAF9 −2 4 4 4 −31 36 15 16 −3 9 12 12 11 7 12 10
RPL33A −6 3 −1 6 −31 43 9 30 10 1 5 1 19 −2 18 2
ROX3 −16 10 10 15 −38 25 −23 26 10 3 5 0 20 14 29 12
BCK2 −23 25 19 26 −32 34 35 33 2 3 5 6 9 5 −9 6
YNL140C −19 17 23 23 −20 25 21 20 3 3 2 1 13 −5 −8 0
PKH3 −30 29 23 3 −34 12 25 14 3 −2 2 5 8 3 0 6
NBP2 −43 29 31 21 −36 24 −12 24 3 −2 −2 −1 22 −12 −13 −15
YDL032W −25 15 7 16 −87 46 16 51 1 1 2 2 23 18 26 18
MRPL23 −21 6 13 12 −58 21 7 51 −2 1 1 1 8 11 28 8
YOR305W −51 15 23 24 −91 11 −13 20 10 2 2 −1 25 15 38 12
RPL36B −1 3 −6 −1 −86 55 16 50 14 0 11 10 20 39 34 33
YPD.K.Shift
HL.K.INTERACTION
CSH.K.INTERACTION
HL.AS.K.INTERACTION
YPEG.K.Shift
HLEG.K.INTERACTION
CSHEG.K.INTERACTION
HLEG.AS.K.INTERACTION
YPD.L.SHIFT
HL.L.INTERACTION
CSH.L.INTERACTION
HL.AS.L.INTERACTION
YPEG.L.SHIFT
HLEG.L.INTERACTION
CSHEG.L.INTERACTION
HLEG.AS.L.INTERACTION
Gene Name
Type of Media

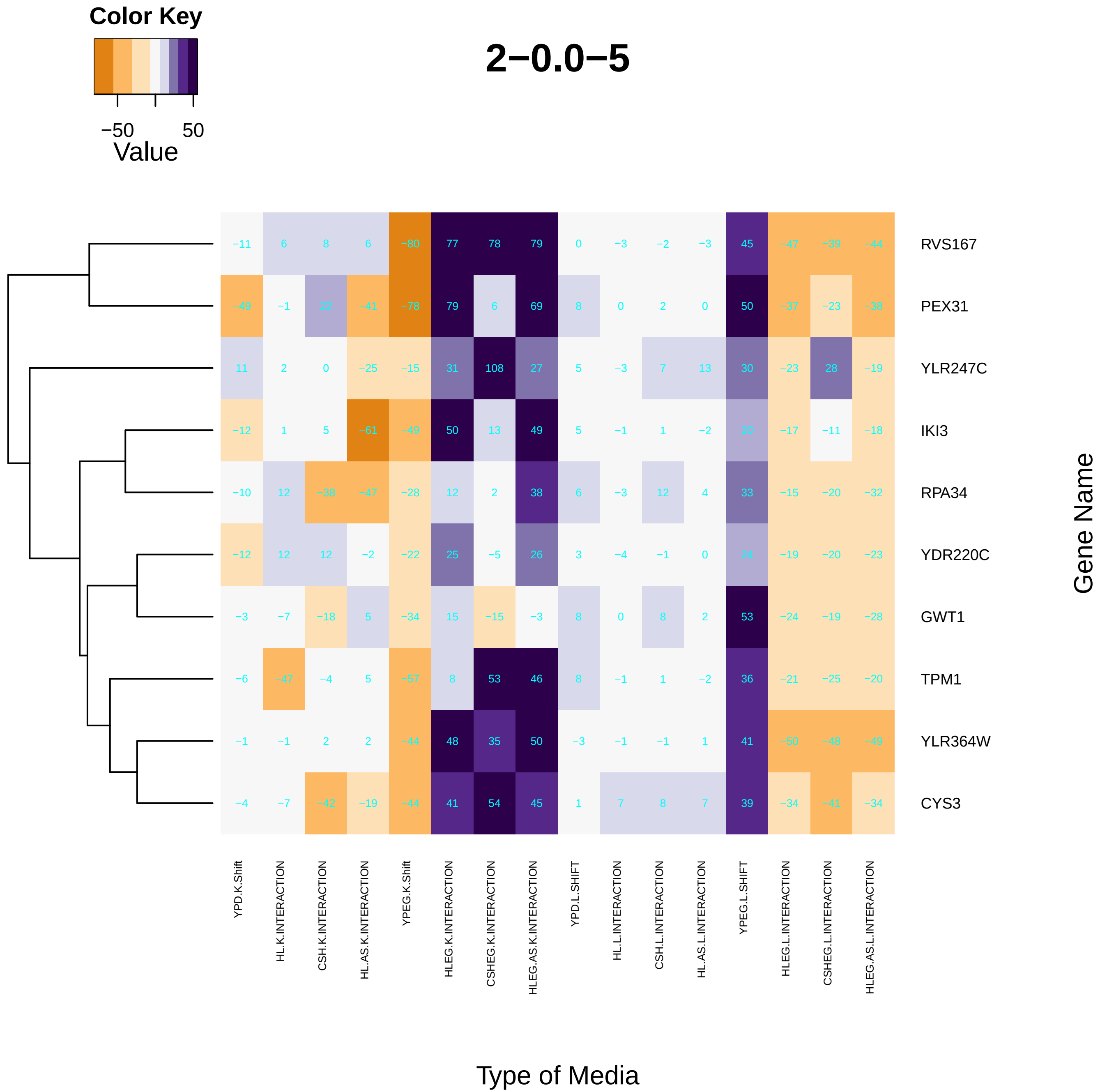
Color Key
−50
50
Value
2−0.0−5
YPD.K.Shift
HL.K.INTERACTION
CSH.K.INTERACTION
HL.AS.K.INTERACTION
YPEG.K.Shift
HLEG.K.INTERACTION
CSHEG.K.INTERACTION
HLEG.AS.K.INTERACTION
YPD.L.SHIFT
HL.L.INTERACTION
CSH.L.INTERACTION
HL.AS.L.INTERACTION
YPEG.L.SHIFT
HLEG.L.INTERACTION
CSHEG.L.INTERACTION
HLEG.AS.L.INTERACTION
RVS167 −11 6 8 6 −80 77 78 79 0 −3 −2 −3 45 −47 −39 −44
PEX31 −49 −1 22 −41 −78 79 6 69 8 0 2 0 50 −37 −23 −38
YLR247C 11 2 0 −25 −15 31 108 27 5 −3 7 13 30 −23 28 −19
IKI3 −12 1 5 −61 −49 50 13 49 5 −1 1 −2 20 −17 −11 −18
RPA34 −10 12 −38 −47 −28 12 2 38 6 −3 12 4 33 −15 −20 −32
YDR220C −12 12 12 −2 −22 25 −5 26 3 −4 −1 0 24 −19 −20 −23
GWT1 −3 −7 −18 5 −34 15 −15 −3 8 0 8 2 53 −24 −19 −28
TPM1 −6 −47 −4 5 −57 8 53 46 8 −1 1 −2 36 −21 −25 −20
YLR364W −1 −1 2 2 −44 48 35 50 −3 −1 −1 1 41 −50 −48 −49
CYS3 −4 −7 −42 −19 −44 41 54 45 1 7 8 7 39 −34 −41 −34
Gene Name
Type of Media

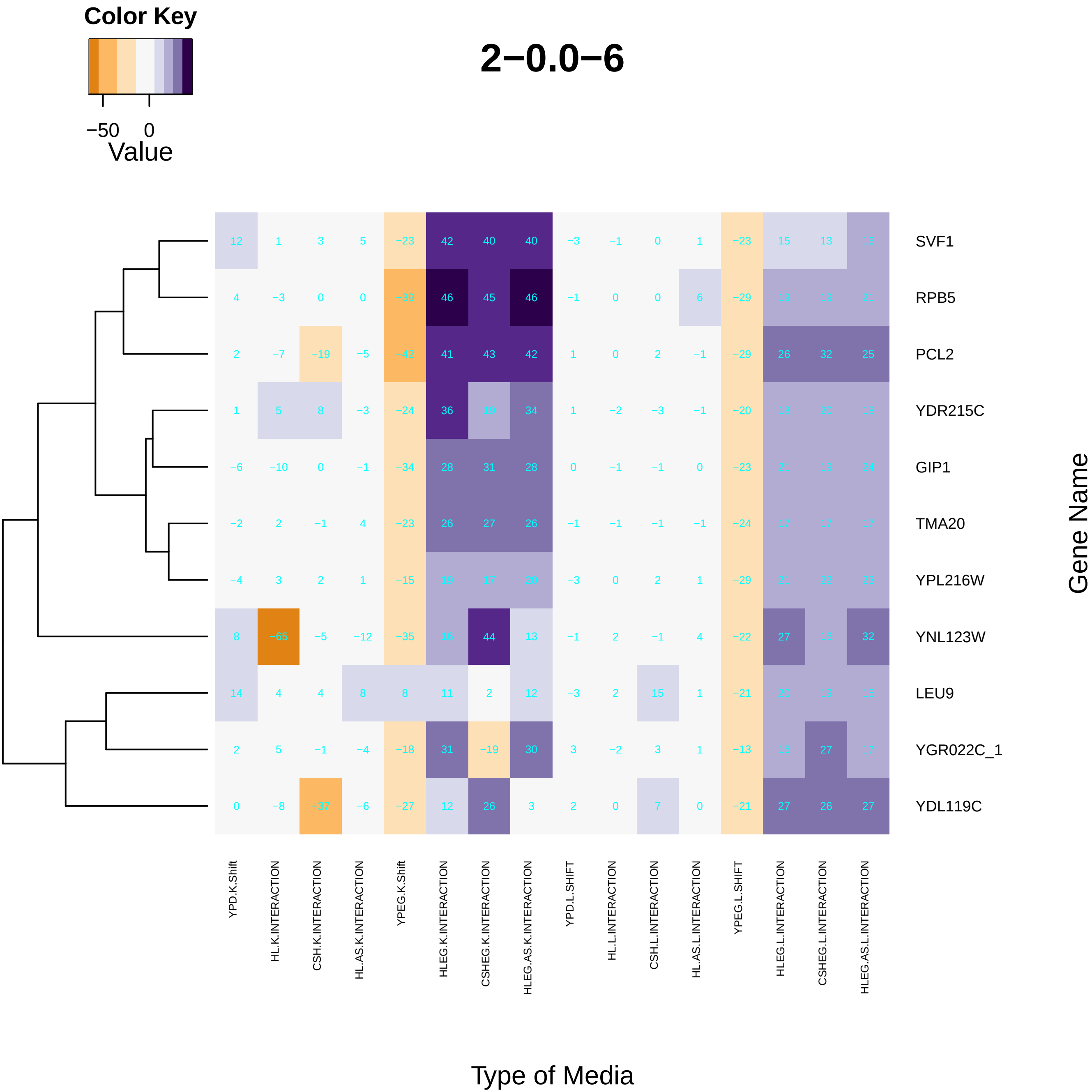
Color Key
−50 0
Value
2−0.0−6
SVF1
RPB5
PCL2
YDR215C
GIP1
TMA20
YPL216W
YNL123W
LEU9
YGR022C_1
YDL119C
Gene Name
YPD.K.Shift
HL.K.INTERACTION
CSH.K.INTERACTION
HL.AS.K.INTERACTION
YPEG.K.Shift
HLEG.K.INTERACTION
CSHEG.K.INTERACTION
HLEG.AS.K.INTERACTION
YPD.L.SHIFT
HL.L.INTERACTION
CSH.L.INTERACTION
HL.AS.L.INTERACTION
YPEG.L.SHIFT
HLEG.L.INTERACTION
CSHEG.L.INTERACTION
HLEG.AS.L.INTERACTION
Type of Media

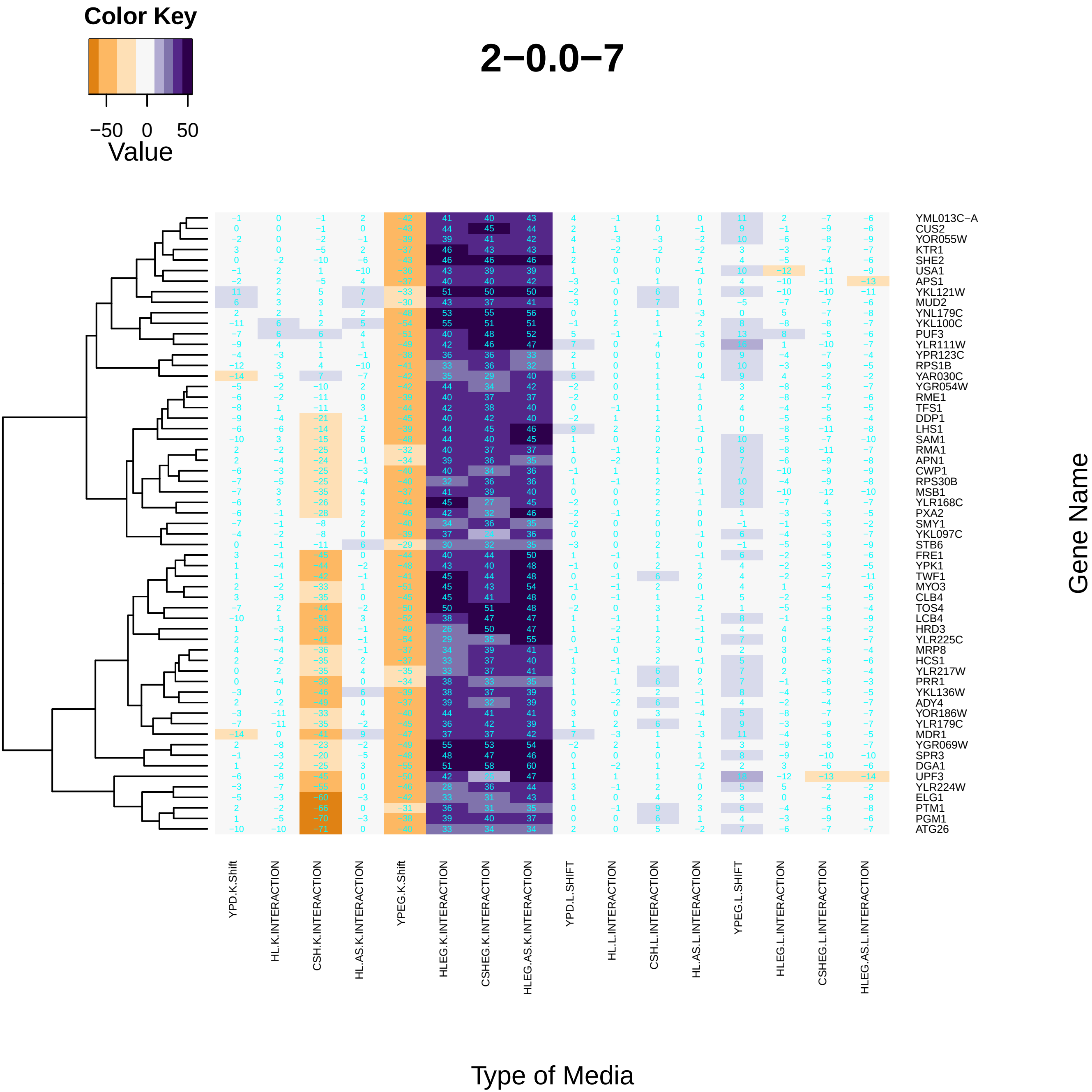

Color Key
−50 0 50
Value
2−0.0−7
YML013C−A
CUS2
YOR055W
KTR1
SHE2
USA1
APS1
YKL121W
MUD2
YNL179C
YKL100C
PUF3
YLR111W
YPR123C
RPS1B
YAR030C
YGR054W
RME1
TFS1
DDP1
LHS1
SAM1
RMA1
APN1
CWP1
RPS30B
MSB1
YLR168C
PXA2
SMY1
YKL097C
STB6
FRE1
YPK1
TWF1
MYO3
CLB4
TOS4
LCB4
HRD3
YLR225C
MRP8
HCS1
YLR217W
PRR1
YKL136W
ADY4
YOR186W
YLR179C
MDR1
YGR069W
SPR3
DGA1
UPF3
YLR224W
ELG1
PTM1
PGM1
ATG26
Gene Name
YPD.K.Shift
HL.K.INTERACTION
CSH.K.INTERACTION
HL.AS.K.INTERACTION
YPEG.K.Shift
HLEG.K.INTERACTION
CSHEG.K.INTERACTION
HLEG.AS.K.INTERACTION
YPD.L.SHIFT
HL.L.INTERACTION
CSH.L.INTERACTION
HL.AS.L.INTERACTION
YPEG.L.SHIFT
HLEG.L.INTERACTION
CSHEG.L.INTERACTION
HLEG.AS.L.INTERACTION
Type of Media

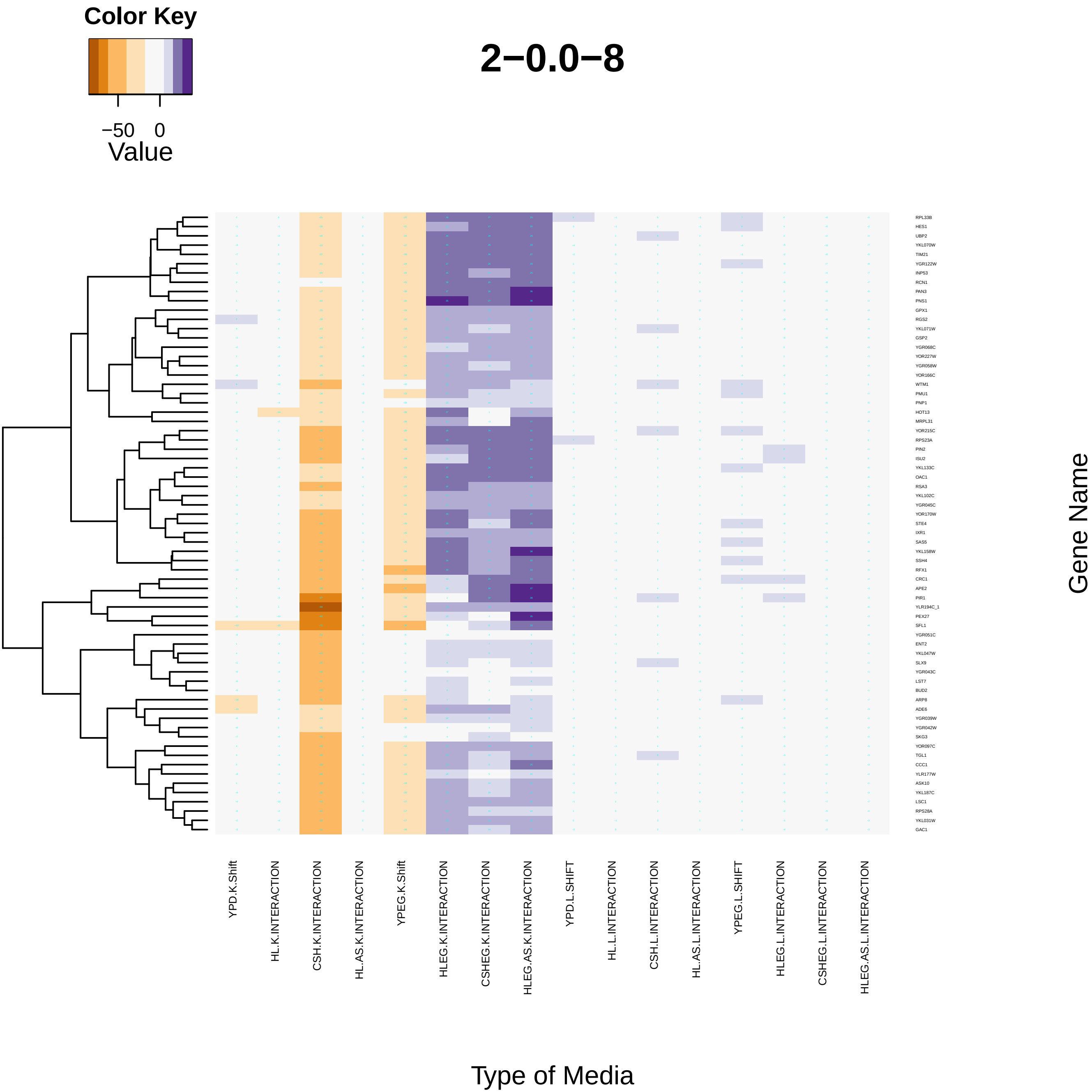
Color Key
−50
0
Value
2−0.0−8
RPL33B
HES1
UBP2
YKL070W
TIM21
YGR122W
INP53
RCN1
PAN3
PNS1
GPX1
RGS2
YKL071W
GSP2
YGR068C
YOR227W
YGR058W
YOR166C
WTM1
PMU1
PNP1
HOT13
MRPL31
YOR215C
RPS23A
PIN2
ISU2
YKL133C
OAC1
RSA3
YKL102C
YGR045C
YOR170W
STE4
IXR1
SAS5
YKL158W
SSH4
RFX1
CRC1
APE2
PIR1
YLR194C_1
PEX27
SFL1
YGR051C
ENT2
YKL047W
SLX9
YGR043C
LST7
BUD2
ARP8
ADE6
YGR039W
YGR042W
SKG3
YOR097C
TGL1
CCC1
YLR177W
ASK10
YKL187C
LSC1
RPS28A
YKL031W
GAC1
Gene Name
YPD.K.Shift
HL.K.INTERACTION
CSH.K.INTERACTION
HL.AS.K.INTERACTION
YPEG.K.Shift
HLEG.K.INTERACTION
CSHEG.K.INTERACTION
HLEG.AS.K.INTERACTION
YPD.L.SHIFT
HL.L.INTERACTION
CSH.L.INTERACTION
HL.AS.L.INTERACTION
YPEG.L.SHIFT
HLEG.L.INTERACTION
CSHEG.L.INTERACTION
HLEG.AS.L.INTERACTION
Type of Media

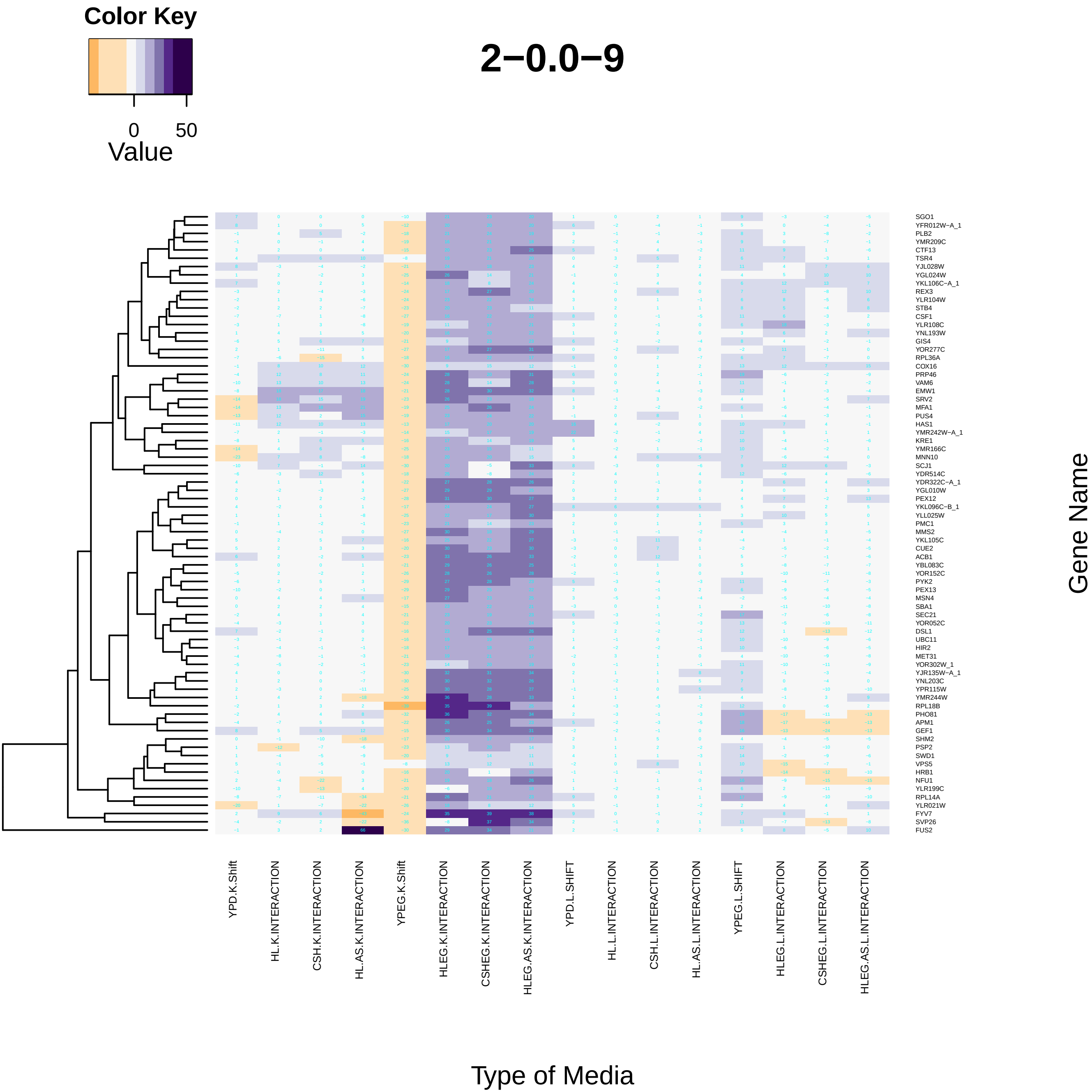

Color Key
0
50
Value
2−0.0−9
SGO1
YFR012W−A_1
PLB2
YMR209C
CTF13
TSR4
YJL028W
YGL024W
YKL106C−A_1
REX3
YLR104W
STB4
CSF1
YLR108C
YNL193W
GIS4
YOR277C
RPL36A
COX16
PRP46
VAM6
EMW1
SRV2
MFA1
PUS4
HAS1
YMR242W−A_1
KRE1
YMR166C
MNN10
SCJ1
YDR514C
YDR322C−A_1
YGL010W
PEX12
YKL096C−B_1
YLL025W
PMC1
MMS2
YKL105C
CUE2
ACB1
YBL083C
YOR152C
PYK2
PEX13
MSN4
SBA1
SEC21
YOR052C
DSL1
UBC11
HIR2
MET31
YOR302W_1
YJR135W−A_1
YNL203C
YPR115W
YMR244W
RPL18B
PHO81
APM1
GEF1
SHM2
PSP2
SWD1
VPS5
HRB1
NFU1
YLR199C
RPL14A
YLR021W
FYV7
SVP26
FUS2
Gene Name
YPD.K.Shift
HL.K.INTERACTION
CSH.K.INTERACTION
HL.AS.K.INTERACTION
YPEG.K.Shift
HLEG.K.INTERACTION
CSHEG.K.INTERACTION
HLEG.AS.K.INTERACTION
YPD.L.SHIFT
HL.L.INTERACTION
CSH.L.INTERACTION
HL.AS.L.INTERACTION
YPEG.L.SHIFT
HLEG.L.INTERACTION
CSHEG.L.INTERACTION
HLEG.AS.L.INTERACTION
Type of Media

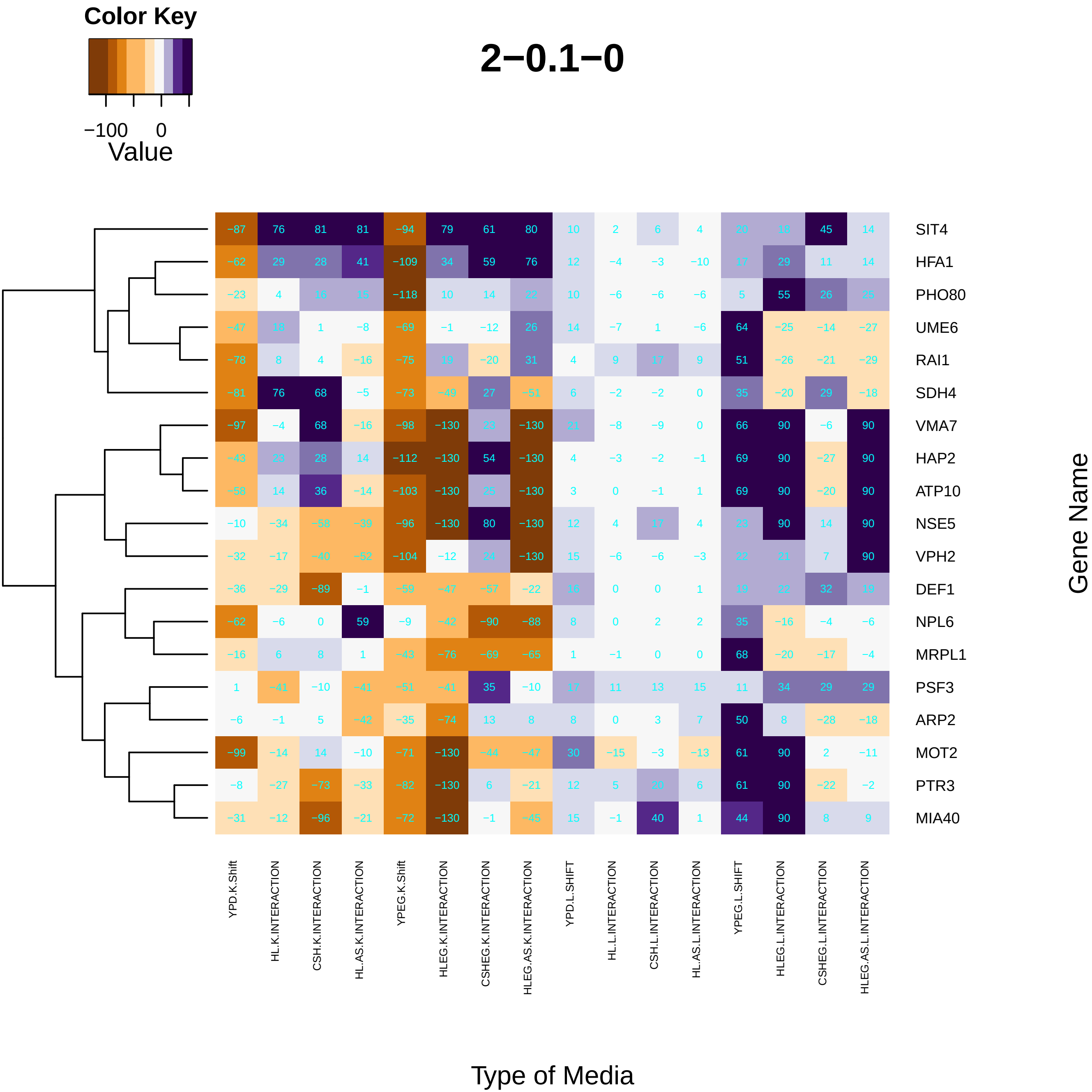
Color Key
−100
0
Value
2−0.1−0
SIT4
HFA1
PHO80
UME6
RAI1
SDH4
VMA7
HAP2
ATP10
NSE5
VPH2
DEF1
NPL6
MRPL1
PSF3
ARP2
MOT2
PTR3
MIA40
Gene Name
YPD.K.Shift
HL.K.INTERACTION
CSH.K.INTERACTION
HL.AS.K.INTERACTION
YPEG.K.Shift
HLEG.K.INTERACTION
CSHEG.K.INTERACTION
HLEG.AS.K.INTERACTION
YPD.L.SHIFT
HL.L.INTERACTION
CSH.L.INTERACTION
HL.AS.L.INTERACTION
YPEG.L.SHIFT
HLEG.L.INTERACTION
CSHEG.L.INTERACTION
HLEG.AS.L.INTERACTION
Type of Media

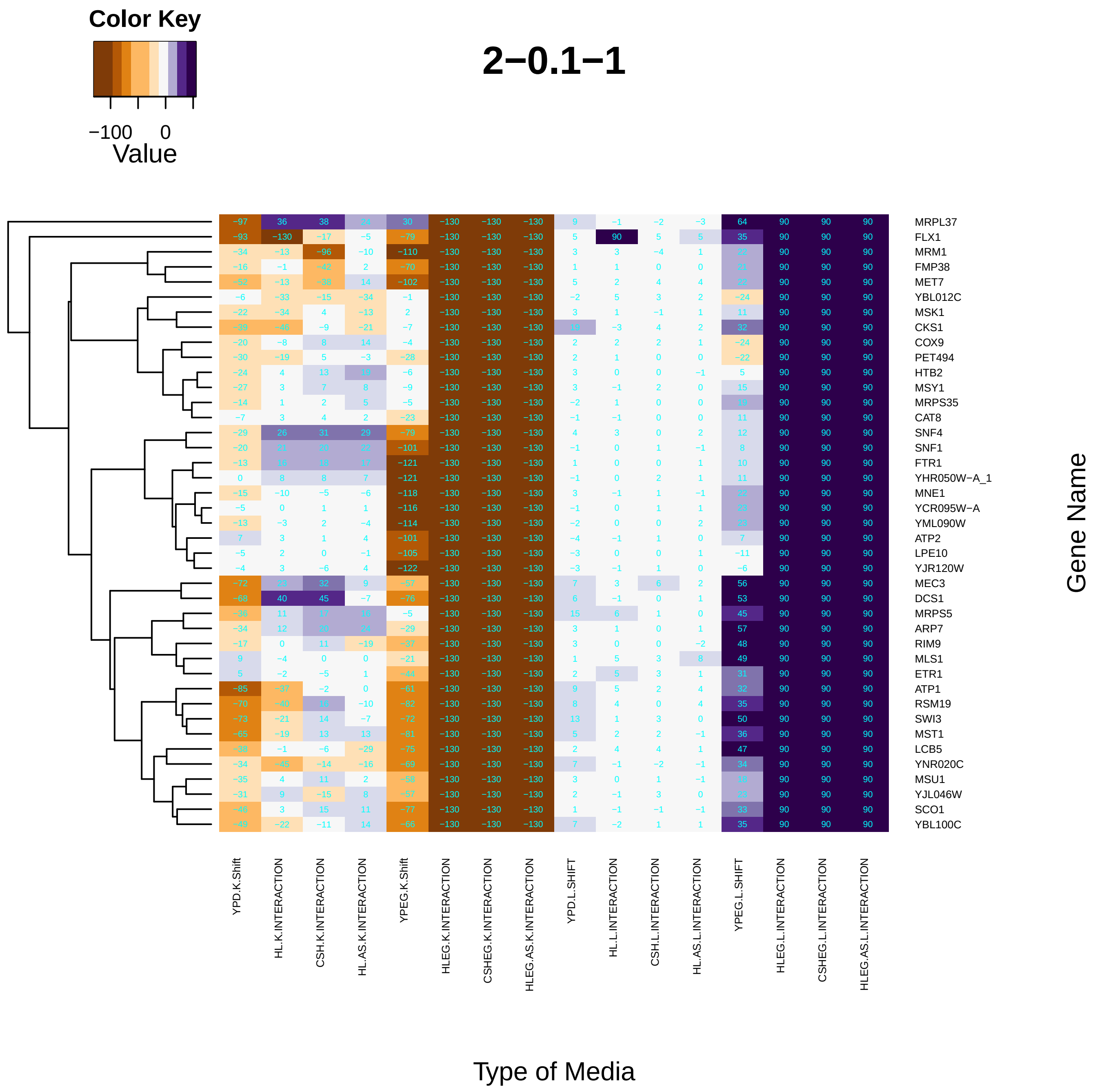
Color Key
−100
0
Value
2−0.1−1
MRPL37
FLX1
MRM1
FMP38
MET7
YBL012C
MSK1
CKS1
COX9
PET494
HTB2
MSY1
MRPS35
CAT8
SNF4
SNF1
FTR1
YHR050W−A_1
MNE1
YCR095W−A
YML090W
ATP2
LPE10
YJR120W
MEC3
DCS1
MRPS5
ARP7
RIM9
MLS1
ETR1
ATP1
RSM19
SWI3
MST1
LCB5
YNR020C
MSU1
YJL046W
SCO1
YBL100C
Gene Name
YPD.K.Shift
HL.K.INTERACTION
CSH.K.INTERACTION
HL.AS.K.INTERACTION
YPEG.K.Shift
HLEG.K.INTERACTION
CSHEG.K.INTERACTION
HLEG.AS.K.INTERACTION
YPD.L.SHIFT
HL.L.INTERACTION
CSH.L.INTERACTION
HL.AS.L.INTERACTION
YPEG.L.SHIFT
HLEG.L.INTERACTION
CSHEG.L.INTERACTION
HLEG.AS.L.INTERACTION
Type of Media

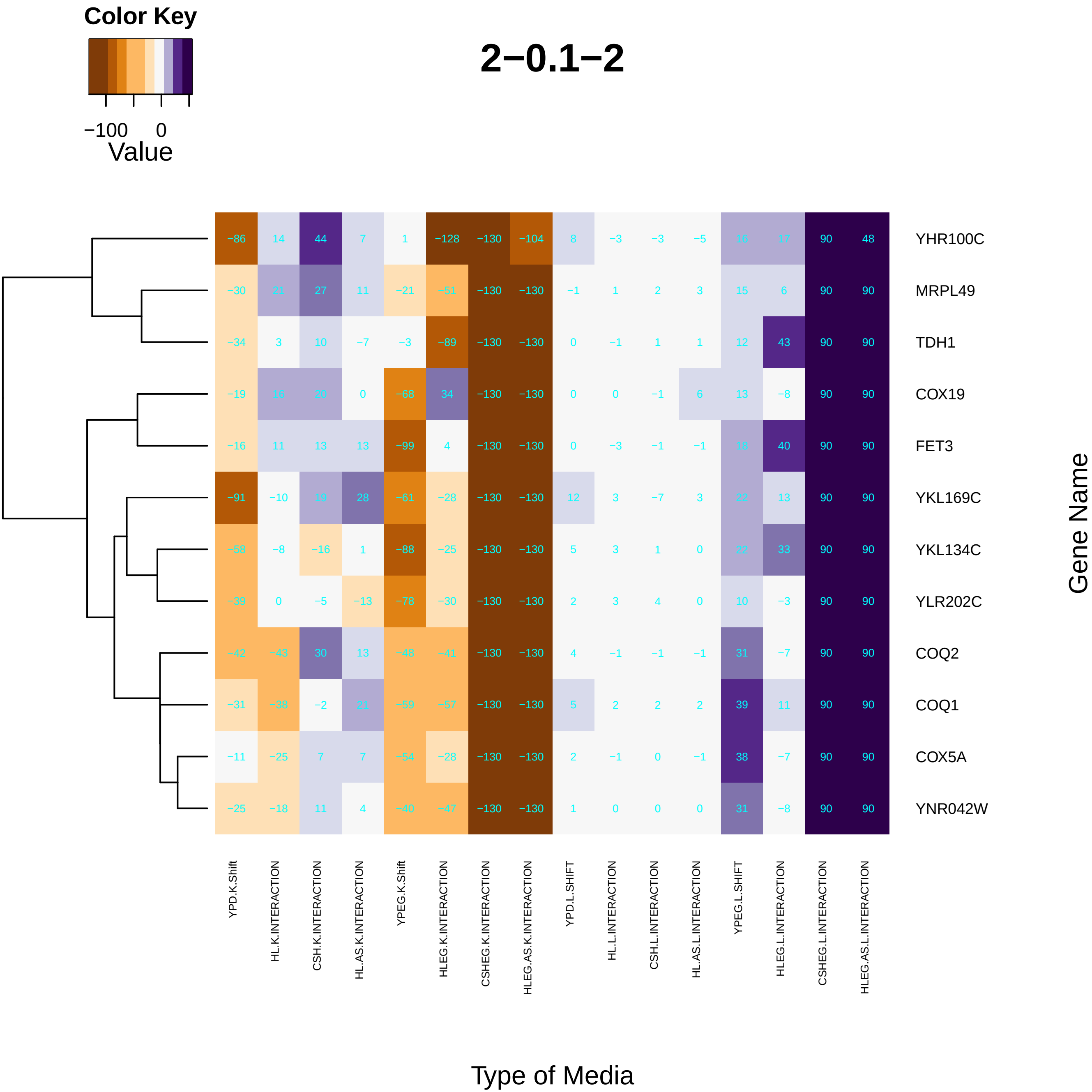

Color Key
−100
0
Value
2−0.1−2
YHR100C
MRPL49
TDH1
COX19
FET3
YKL169C
YKL134C
YLR202C
COQ2
COQ1
COX5A
YNR042W
Gene Name
YPD.K.Shift
HL.K.INTERACTION
CSH.K.INTERACTION
HL.AS.K.INTERACTION
YPEG.K.Shift
HLEG.K.INTERACTION
CSHEG.K.INTERACTION
HLEG.AS.K.INTERACTION
YPD.L.SHIFT
HL.L.INTERACTION
CSH.L.INTERACTION
HL.AS.L.INTERACTION
YPEG.L.SHIFT
HLEG.L.INTERACTION
CSHEG.L.INTERACTION
HLEG.AS.L.INTERACTION
Type of Media

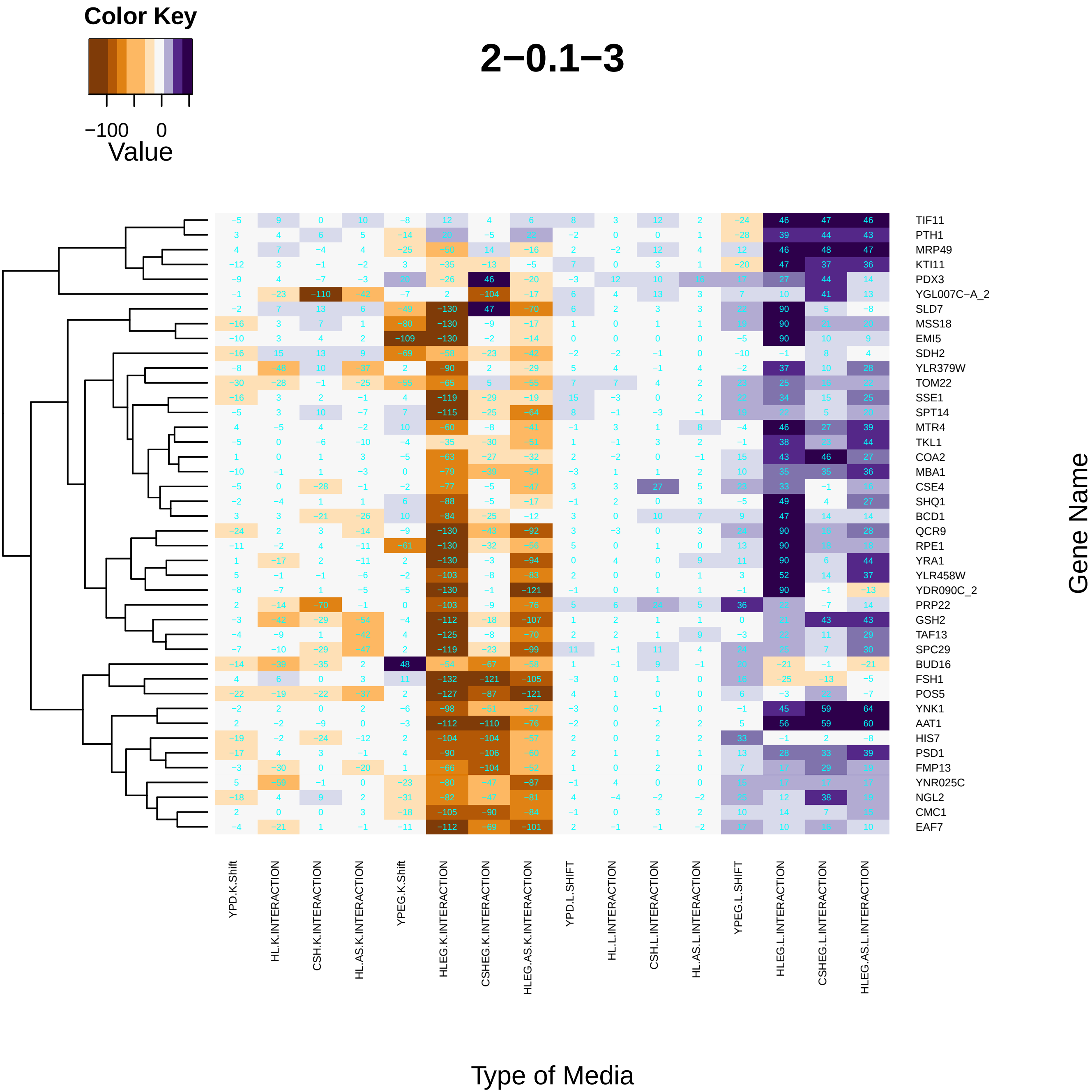
Color Key
−100
0
Value
2−0.1−3
TIF11
PTH1
MRP49
KTI11
PDX3
YGL007C−A_2
SLD7
MSS18
EMI5
SDH2
YLR379W
TOM22
SSE1
SPT14
MTR4
TKL1
COA2
MBA1
CSE4
SHQ1
BCD1
QCR9
RPE1
YRA1
YLR458W
YDR090C_2
PRP22
GSH2
TAF13
SPC29
BUD16
FSH1
POS5
YNK1
AAT1
HIS7
PSD1
FMP13
YNR025C
NGL2
CMC1
EAF7
Gene Name
YPD.K.Shift
HL.K.INTERACTION
CSH.K.INTERACTION
HL.AS.K.INTERACTION
YPEG.K.Shift
HLEG.K.INTERACTION
CSHEG.K.INTERACTION
HLEG.AS.K.INTERACTION
YPD.L.SHIFT
HL.L.INTERACTION
CSH.L.INTERACTION
HL.AS.L.INTERACTION
YPEG.L.SHIFT
HLEG.L.INTERACTION
CSHEG.L.INTERACTION
HLEG.AS.L.INTERACTION
Type of Media

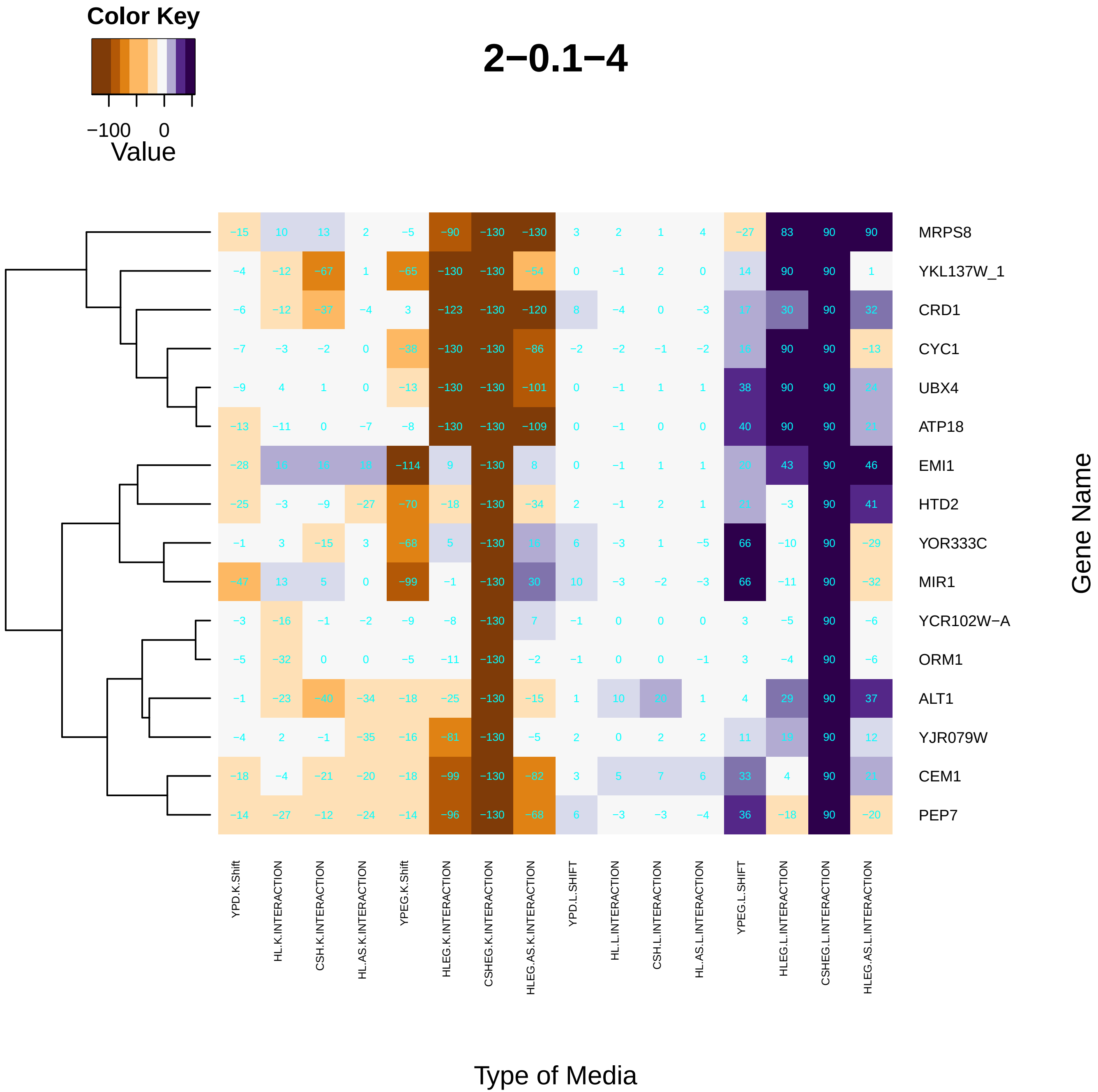
Color Key
−100 0
Value
2−0.1−4
YPD.K.Shift
HL.K.INTERACTION
CSH.K.INTERACTION
HL.AS.K.INTERACTION
YPEG.K.Shift
HLEG.K.INTERACTION
CSHEG.K.INTERACTION
HLEG.AS.K.INTERACTION
YPD.L.SHIFT
HL.L.INTERACTION
CSH.L.INTERACTION
HL.AS.L.INTERACTION
YPEG.L.SHIFT
HLEG.L.INTERACTION
CSHEG.L.INTERACTION
HLEG.AS.L.INTERACTION
MRPS8 −15 10 13 2 −5 −90 −130 −130 3 2 1 4 −27 83 90 90
YKL137W_1 −4 −12 −67 1 −65 −130 −130 −54 0 −1 2 0 14 90 90 1
CRD1 −6 −12 −37 −4 3 −123 −130 −120 8 −4 0 −3 17 30 90 32
CYC1 −7 −3 −2 0 −38 −130 −130 −86 −2 −2 −1 −2 16 90 90 −13
UBX4 −9 4 1 0 −13 −130 −130 −101 0 −1 1 1 38 90 90 24
ATP18 −13 −11 0 −7 −8 −130 −130 −109 0 −1 0 0 40 90 90 21
EMI1 −28 16 16 18 −114 9 −130 8 0 −1 1 1 20 43 90 46
HTD2 −25 −3 −9 −27 −70 −18 −130 −34 2 −1 2 1 21 −3 90 41
YOR333C −1 3 −15 3 −68 5 −130 16 6 −3 1 −5 66 −10 90 −29
MIR1 −47 13 5 0 −99 −1 −130 30 10 −3 −2 −3 66 −11 90 −32
YCR102W−A −3 −16 −1 −2 −9 −8 −130 7 −1 0 0 0 3 −5 90 −6
ORM1 −5 −32 0 0 −5 −11 −130 −2 −1 0 0 −1 3 −4 90 −6
ALT1 −1 −23 −40 −34 −18 −25 −130 −15 1 10 20 1 4 29 90 37
YJR079W −4 2 −1 −35 −16 −81 −130 −5 2 0 2 2 11 19 90 12
CEM1 −18 −4 −21 −20 −18 −99 −130 −82 3 5 7 6 33 4 90 21
PEP7 −14 −27 −12 −24 −14 −96 −130 −68 6 −3 −3 −4 36 −18 90 −20
Gene Name
Type of Media

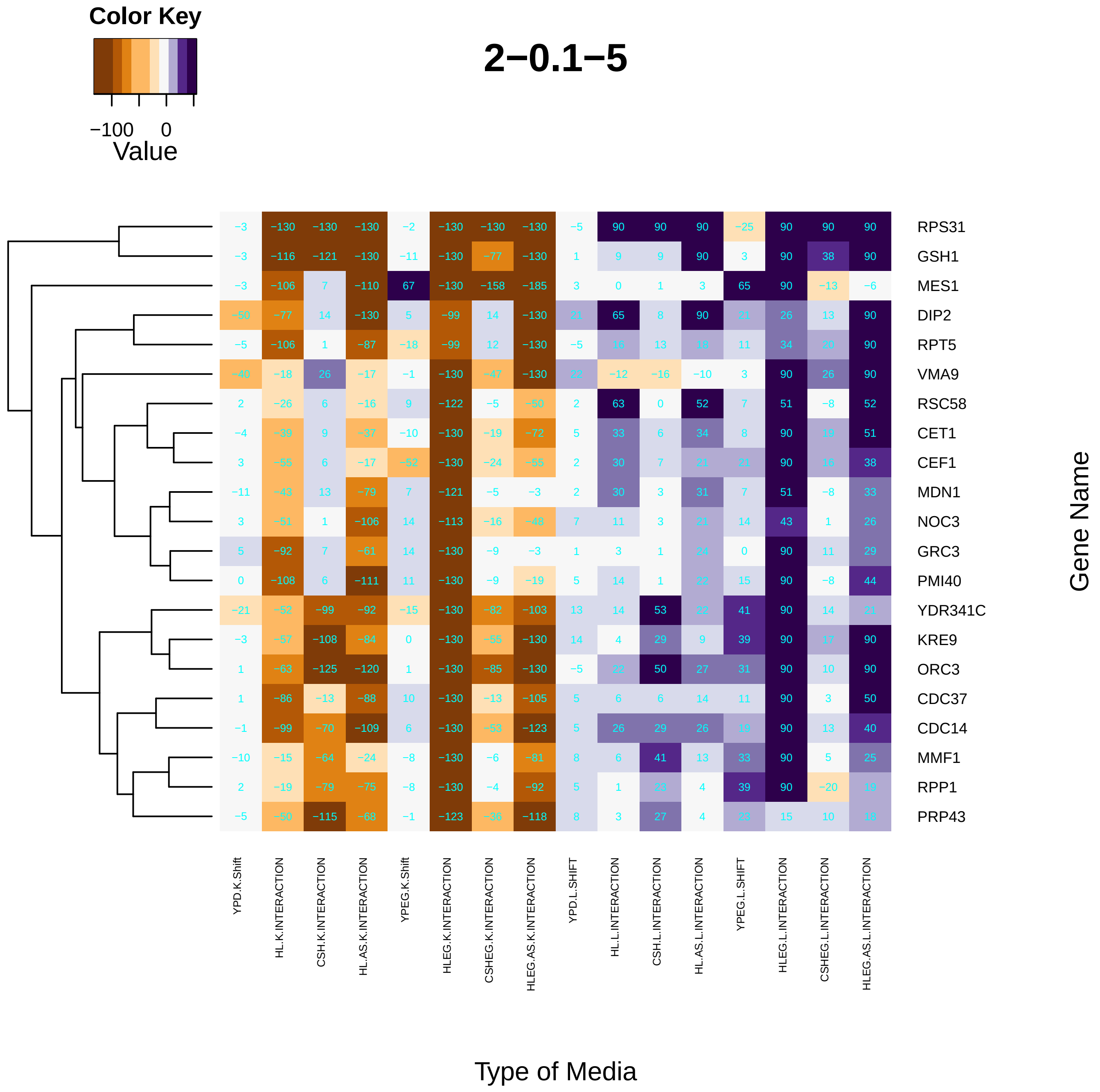
Color Key
−100 0
Value
2−0.1−5
YPD.K.Shift HL.K.INTERACTION CSH.K.INTERACTION HL.AS.K.INTERACTION YPEG.K.Shift HLEG.K.INTERACTION CSHEG.K.INTERACTION HLEG.AS.K.INTERACTION YPD.L.SHIFT HL.L.INTERACTION CSH.L.INTERACTION HL.AS.L.INTERACTION YPEG.L.SHIFT HLEG.L.INTERACTION CSHEG.L.INTERACTION HLEG.AS.L.INTERACTION
RPS31 −3 −130 −130 −130 −2 −130 −130 −130 −5 90 90 90 −25 90 90 90
GSH1 −3 −116 −121 −130 −11 −130 −77 −130 1 9 9 90 3 90 38 90
MES1 −3 −106 7 −110 67 −130 −158 −185 3 0 1 3 65 90 −13 −6
DIP2 −50 −77 14 −130 5 −99 14 −130 21 65 8 90 21 26 13 90
RPT5 −5 −106 1 −87 −18 −99 12 −130 −5 16 13 18 11 34 20 90
VMA9 −40 −18 26 −17 −1 −130 −47 −130 22 −12 −16 −10 3 90 26 90
RSC58 2 −26 6 −16 9 −122 −5 −50 2 63 0 52 7 51 −8 52
CET1 −4 −39 9 −37 −10 −130 −19 −72 5 33 6 34 8 90 19 51
CEF1 3 −55 6 −17 −52 −130 −24 −55 2 30 7 21 21 90 16 38
MDN1 −11 −43 13 −79 7 −121 −5 −3 2 30 3 31 7 51 −8 33
NOC3 3 −51 1 −106 14 −113 −16 −48 7 11 3 21 14 43 1 26
GRC3 5 −92 7 −61 14 −130 −9 −3 1 3 1 24 0 90 11 29
PMI40 0 −108 6 −111 11 −130 −9 −19 5 14 1 22 15 90 −8 44
YDR341C −21 −52 −99 −92 −15 −130 −82 −103 13 14 53 22 41 90 14 21
KRE9 −3 −57 −108 −84 0 −130 −55 −130 14 4 29 9 39 90 17 90
ORC3 1 −63 −125 −120 1 −130 −85 −130 −5 22 50 27 31 90 10 90
CDC37 1 −86 −13 −88 10 −130 −13 −105 5 6 6 14 11 90 3 50
CDC14 −1 −99 −70 −109 6 −130 −53 −123 5 26 29 26 19 90 13 40
MMF1 −10 −15 −64 −24 −8 −130 −6 −81 8 6 41 13 33 90 5 25
RPP1 2 −19 −79 −75 −8 −130 −4 −92 5 1 23 4 39 90 −20 19
PRP43 −5 −50 −115 −68 −1 −123 −36 −118 8 3 27 4 23 15 10 18
Gene Name
Type of Media

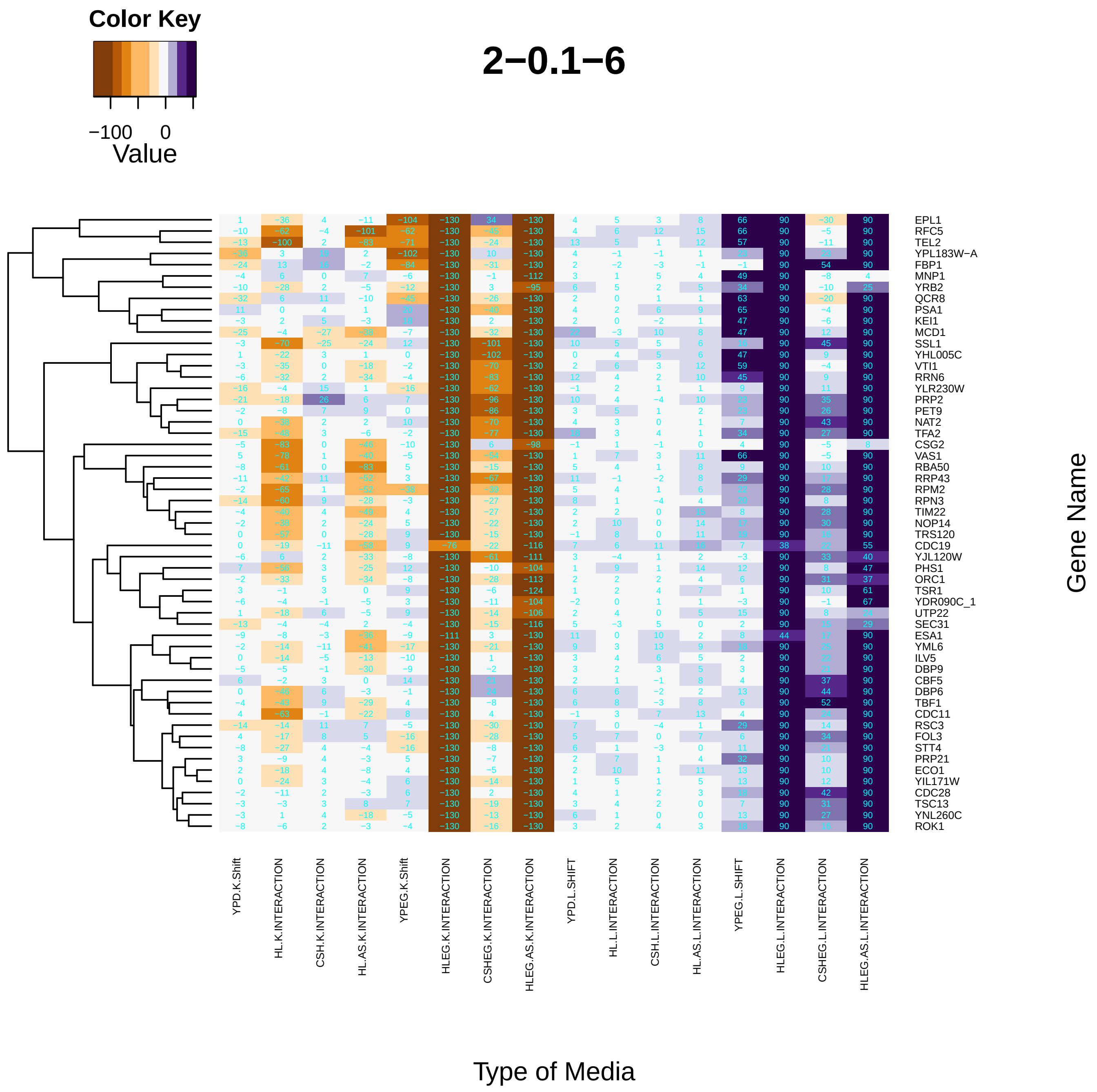
Color Key
−100
0
Value
2−0.1−6
EPL1
RFC5
TEL2
YPL183W−A
FBP1
MNP1
YRB2
QCR8
PSA1
KEI1
MCD1
SSL1
YHL005C
VTI1
RRN6
YLR230W
PRP2
PET9
NAT2
TFA2
CSG2
VAS1
RBA50
RRP43
RPM2
RPN3
TIM22
NOP14
TRS120
CDC19
YJL120W
PHS1
ORC1
TSR1
YDR090C_1
UTP22
SEC31
ESA1
YML6
ILV5
DBP9
CBF5
DBP6
TBF1
CDC11
RSC3
FOL3
STT4
PRP21
ECO1
YIL171W
CDC28
TSC13
YNL260C
ROK1
YPD.K.Shift
HL.K.INTERACTION
CSH.K.INTERACTION
HL.AS.K.INTERACTION
YPEG.K.Shift
HLEG.K.INTERACTION
CSHEG.K.INTERACTION
HLEG.AS.K.INTERACTION
YPD.L.SHIFT
HL.L.INTERACTION
CSH.L.INTERACTION
HL.AS.L.INTERACTION
YPEG.L.SHIFT
HLEG.L.INTERACTION
CSHEG.L.INTERACTION
HLEG.AS.L.INTERACTION
Gene Name
Type of Media

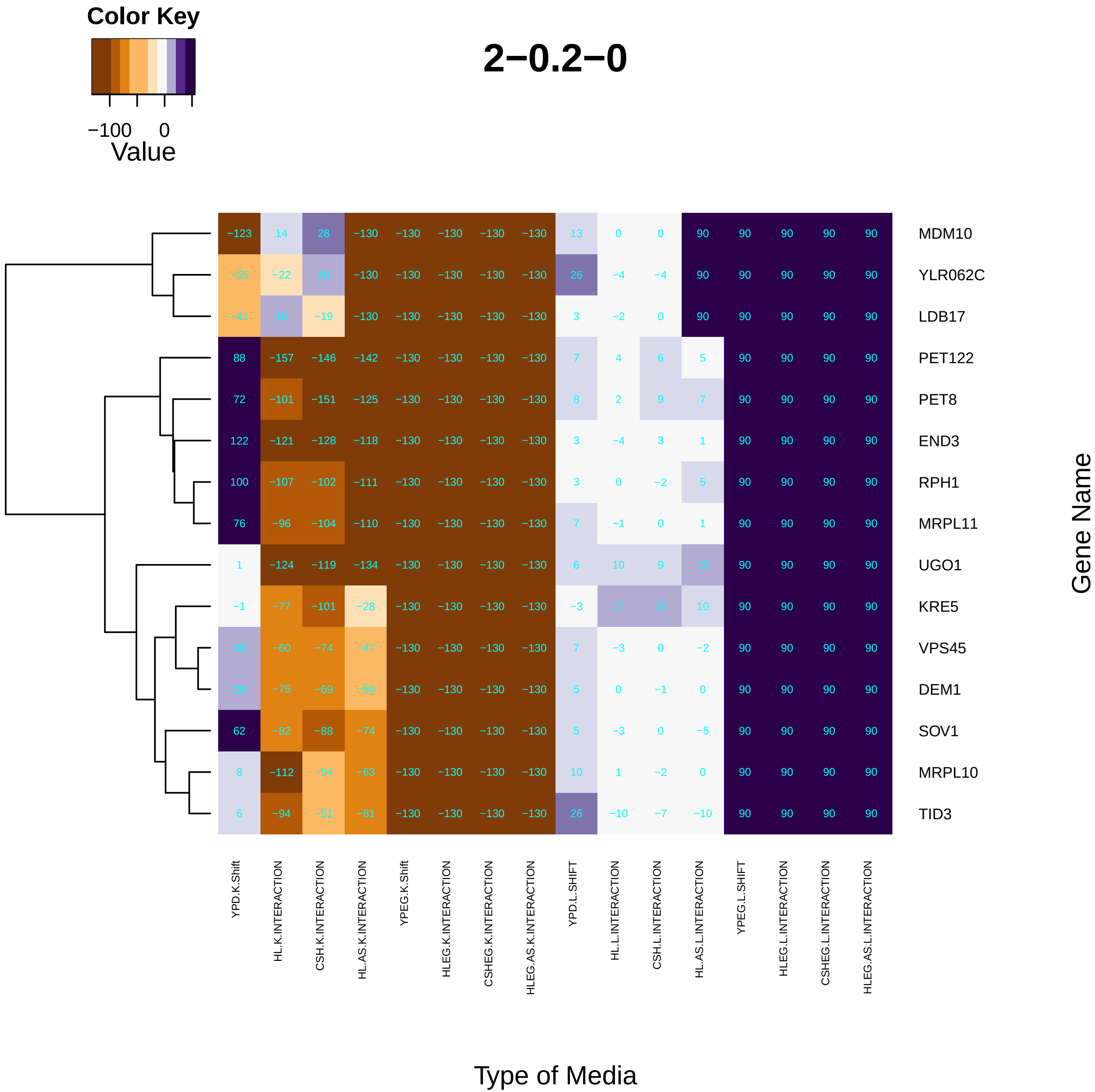
2−0.2−0
Color Key
−100 0
Value
Gene Name
Type of Media
YPD.K.Shift HL.K.INTERACTION CSH.K.INTERACTION HL.AS.K.INTERACTION YPEG.K.Shift HLEG.K.INTERACTION CSHEG.K.INTERACTION HLEG.AS.K.INTERACTION YPD.L.SHIFT HL.L.INTERACTION CSH.L.INTERACTION HL.AS.L.INTERACTION YPEG.L.SHIFT HLEG.L.INTERACTION CSHEG.L.INTERACTION HLEG.AS.L.INTERACTION
MDM10 −123 14 28 −130 −130 −130 −130 −130 13 0 0 90 90 90 90 90
YLR062C −55 −22 20 −130 −130 −130 −130 −130 26 −4 −4 90 90 90 90 90
LDB17 −41 16 −19 −130 −130 −130 −130 −130 3 −2 0 90 90 90 90 90
PET122 88 −157 −146 −142 −130 −130 −130 −130 7 4 6 5 90 90 90 90
PET8 72 −101 −151 −125 −130 −130 −130 −130 8 2 9 7 90 90 90 90
END3 122 −121 −128 −118 −130 −130 −130 −130 3 −4 3 1 90 90 90 90
RPH1 100 −107 −102 −111 −130 −130 −130 −130 3 0 −2 5 90 90 90 90
MRPL11 76 −96 −104 −110 −130 −130 −130 −130 7 −1 0 1 90 90 90 90
UGO1 1 −124 −119 −134 −130 −130 −130 −130 6 10 9 15 90 90 90 90
KRE5 −1 −77 −101 −28 −130 −130 −130 −130 −3 17 16 10 90 90 90 90
VPS45 20 −60 −74 −47 −130 −130 −130 −130 7 −3 0 −2 90 90 90 90
DEM1 20 −75 −69 −59 −130 −130 −130 −130 5 0 −1 0 90 90 90 90
SOV1 62 −82 −88 −74 −130 −130 −130 −130 5 −3 0 −5 90 90 90 90
MRPL10 8 −112 −54 −63 −130 −130 −130 −130 10 1 −2 0 90 90 90 90
TID3 6 −94 −51 −81 −130 −130 −130 −130 26 −10 −7 −10 90 90 90 90

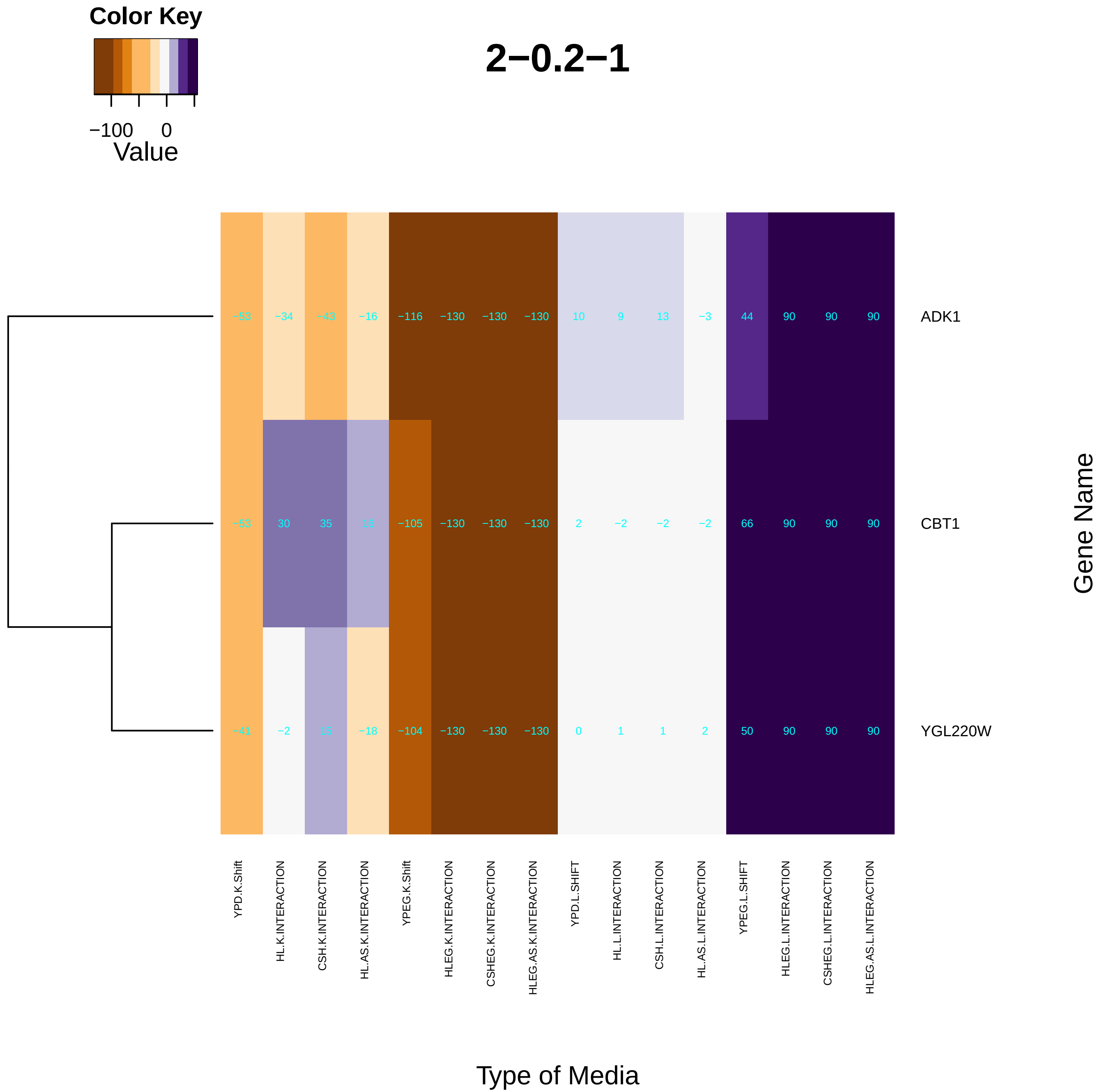

Color Key
−100
0
Value
2−0.2−1
ADK1
CBT1
YGL220W
−53 −34 −43 −16 −116 −130 −130 −130 10 9 13 −3 44 90 90 90
−53 30 35 16 −105 −130 −130 −130 2 −2 −2 −2 66 90 90 90
−41 −2 15 −18 −104 −130 −130 −130 0 1 1 2 50 90 90 90
YPD.K.Shift
HL.K.INTERACTION
CSH.K.INTERACTION
HL.AS.K.INTERACTION
YPEG.K.Shift
HLEG.K.INTERACTION
CSHEG.K.INTERACTION
HLEG.AS.K.INTERACTION
YPD.L.SHIFT
HL.L.INTERACTION
CSH.L.INTERACTION
HL.AS.L.INTERACTION
YPEG.L.SHIFT
HLEG.L.INTERACTION
CSHEG.L.INTERACTION
HLEG.AS.L.INTERACTION
Gene Name
Type of Media

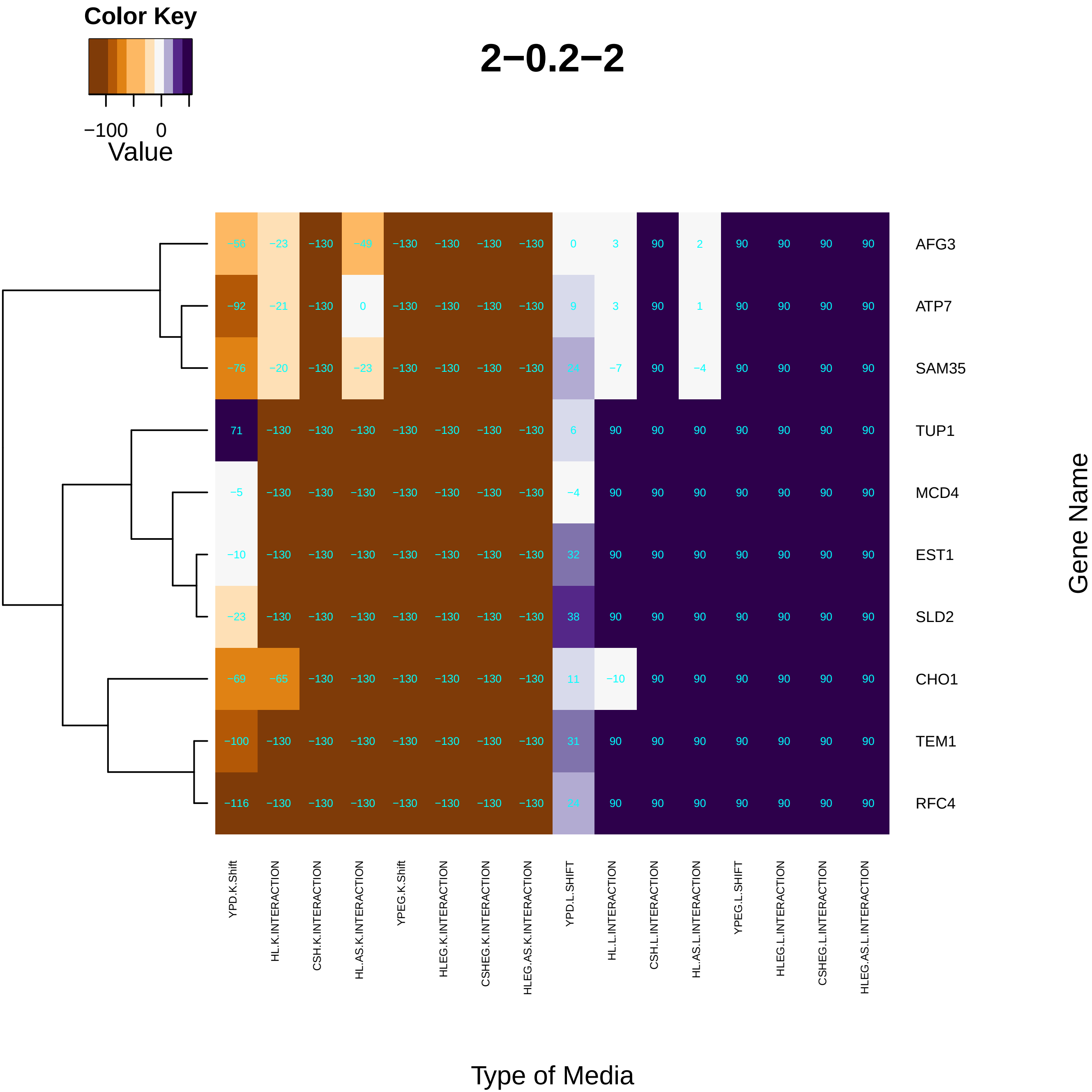
Color Key
−100 0
Value
2−0.2−2
AFG3: −56 −23 −130 −49 −130 −130 −130 −130 0 3 90 2 90 90 90 90
ATP7: −92 −21 −130 0 −130 −130 −130 −130 9 3 90 1 90 90 90 90
SAM35: −76 −20 −130 −23 −130 −130 −130 −130 24 −7 90 −4 90 90 90 90
TUP1: 71 −130 −130 −130 −130 −130 −130 −130 6 90 90 90 90 90 90 90
MCD4: −5 −130 −130 −130 −130 −130 −130 −130 −4 90 90 90 90 90 90 90
EST1: −10 −130 −130 −130 −130 −130 −130 −130 32 90 90 90 90 90 90 90
SLD2: −23 −130 −130 −130 −130 −130 −130 −130 38 90 90 90 90 90 90 90
CHO1: −69 −65 −130 −130 −130 −130 −130 −130 11 −10 90 90 90 90 90 90
TEM1: −100 −130 −130 −130 −130 −130 −130 −130 31 90 90 90 90 90 90 90
RFC4: −116 −130 −130 −130 −130 −130 −130 −130 24 90 90 90 90 90 90 90
YPD.K.Shift
HL.K.INTERACTION
CSH.K.INTERACTION
HL.AS.K.INTERACTION
YPEG.K.Shift
HLEG.K.INTERACTION
CSHEG.K.INTERACTION
HLEG.AS.K.INTERACTION
YPD.L.SHIFT
HL.L.INTERACTION
CSH.L.INTERACTION
HL.AS.L.INTERACTION
YPEG.L.SHIFT
HLEG.L.INTERACTION
CSHEG.L.INTERACTION
HLEG.AS.L.INTERACTION
Gene Name
Type of Media

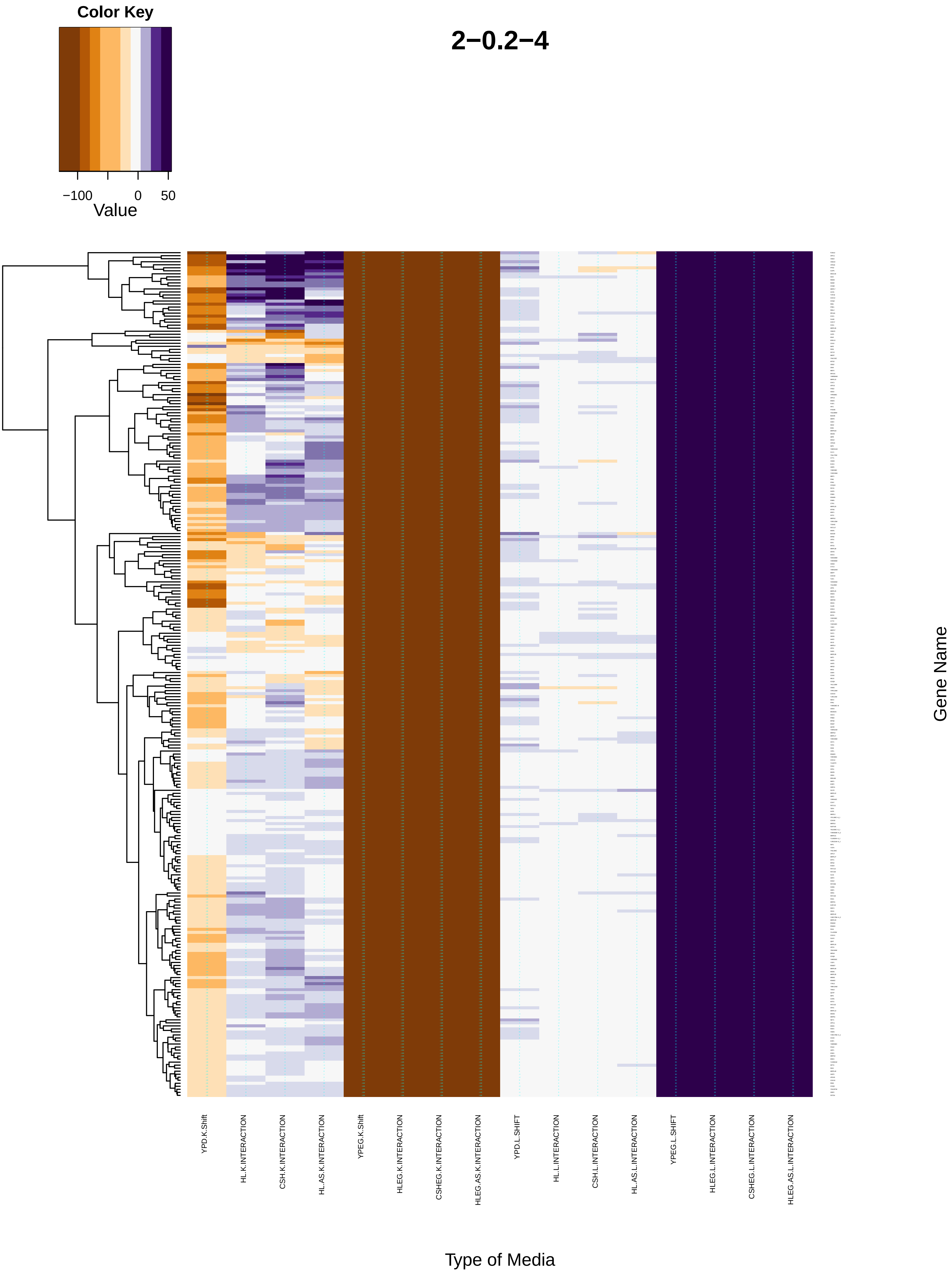
2−0.2−4
Color Key
−100
0
50
Value
YPD.K.Shift
HL.K.INTERACTION
CSH.K.INTERACTION
HL.AS.K.INTERACTION
YPEG.K.Shift
HLEG.K.INTERACTION
CSHEG.K.INTERACTION
HLEG.AS.K.INTERACTION
YPD.L.SHIFT
HL.L.INTERACTION
CSH.L.INTERACTION
HL.AS.L.INTERACTION
YPEG.L.SHIFT
HLEG.L.INTERACTION
CSHEG.L.INTERACTION
HLEG.AS.L.INTERACTION
Type of Media
Gene Name

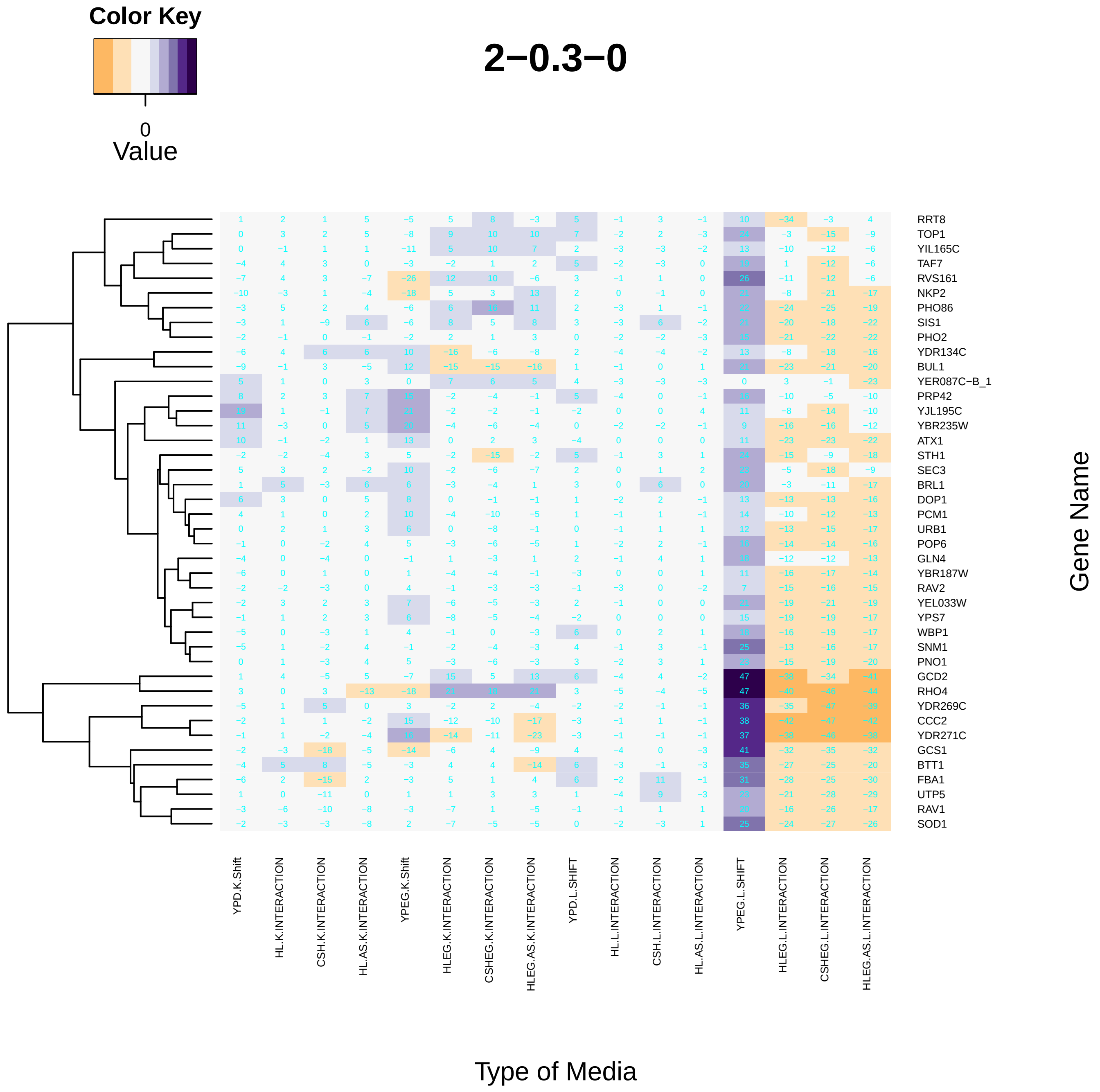

Color Key
0
Value
2−0.3−0
RRT8
TOP1
YIL165C
TAF7
RVS161
NKP2
PHO86
SIS1
PHO2
YDR134C
BUL1
YER087C−B_1
PRP42
YJL195C
YBR235W
ATX1
STH1
SEC3
BRL1
DOP1
PCM1
URB1
POP6
GLN4
YBR187W
RAV2
YEL033W
YPS7
WBP1
SNM1
PNO1
GCD2
RHO4
YDR269C
CCC2
YDR271C
GCS1
BTT1
FBA1
UTP5
RAV1
SOD1
Gene Name
YPD.K.Shift
HL.K.INTERACTION
CSH.K.INTERACTION
HL.AS.K.INTERACTION
YPEG.K.Shift
HLEG.K.INTERACTION
CSHEG.K.INTERACTION
HLEG.AS.K.INTERACTION
YPD.L.SHIFT
HL.L.INTERACTION
CSH.L.INTERACTION
HL.AS.L.INTERACTION
YPEG.L.SHIFT
HLEG.L.INTERACTION
CSHEG.L.INTERACTION
HLEG.AS.L.INTERACTION
Type of Media

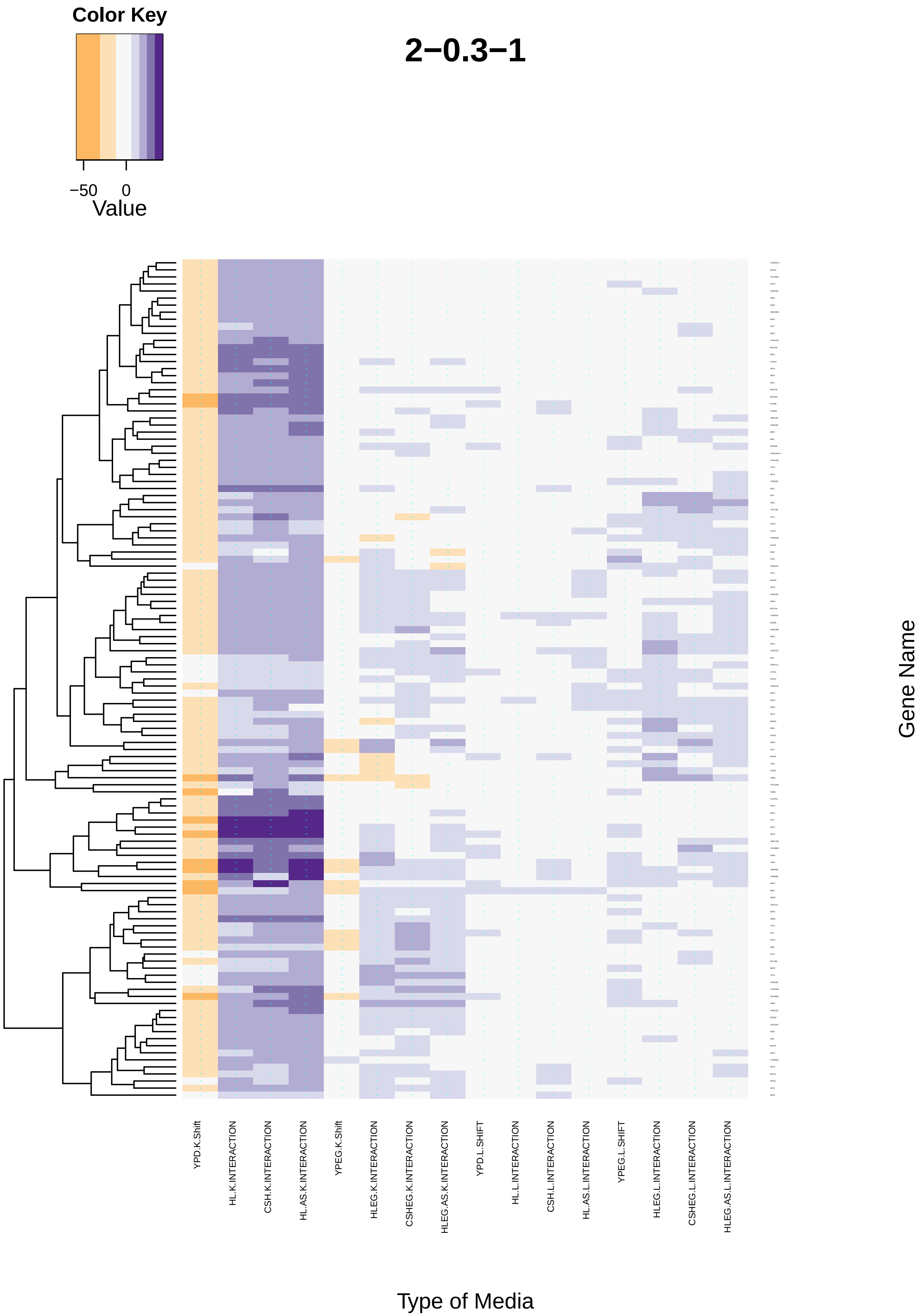
Color Key
−50
0
Value
2−0.3−1
YPD.K.Shift
HL.K.INTERACTION
CSH.K.INTERACTION
HL.AS.K.INTERACTION
YPEG.K.Shift
HLEG.K.INTERACTION
CSHEG.K.INTERACTION
HLEG.AS.K.INTERACTION
YPD.L.SHIFT
HL.L.INTERACTION
CSH.L.INTERACTION
HL.AS.L.INTERACTION
YPEG.L.SHIFT
HLEG.L.INTERACTION
CSHEG.L.INTERACTION
HLEG.AS.L.INTERACTION
Gene Name
Type of Media

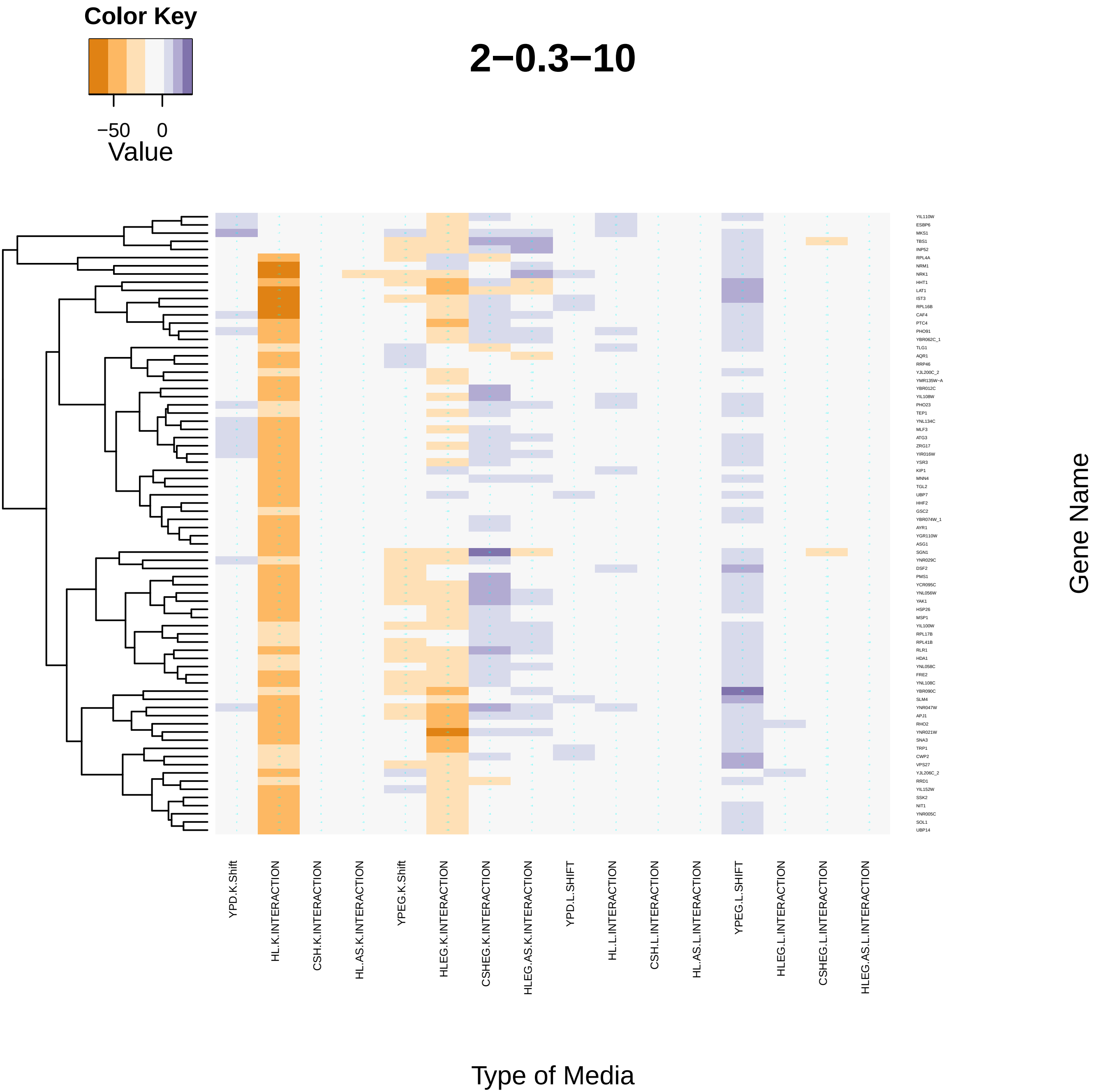

Color Key
−50
0
Value
2−0.3−10
YIL110W
ESBP6
MKS1
TBS1
INP52
RPL4A
NRM1
NRK1
HHT1
LAT1
IST3
RPL16B
CAF4
PTC4
PHO91
YBR062C_1
TLG1
AQR1
RRP46
YJL200C_2
YMR135W−A
YBR012C
YIL108W
PHO23
TEP1
YNL134C
MLF3
ATG3
ZRG17
YIR016W
YSR3
KIP1
MNN4
TGL2
UBP7
HHF2
GSC2
YBR074W_1
AYR1
YGR110W
ASG1
SGN1
YNR029C
DSF2
PMS1
YCR095C
YNL056W
YAK1
HSP26
MSP1
YIL100W
RPL17B
RPL41B
RLR1
HDA1
YNL058C
FRE2
YNL108C
YBR090C
SLM4
YNR047W
APJ1
RHO2
YNR021W
SNA3
TRP1
CWP2
VPS27
YJL206C_2
RRD1
YIL152W
SSK2
NIT1
YNR005C
SOL1
UBP14
Gene Name
YPD.K.Shift
HL.K.INTERACTION
CSH.K.INTERACTION
HL.AS.K.INTERACTION
YPEG.K.Shift
HLEG.K.INTERACTION
CSHEG.K.INTERACTION
HLEG.AS.K.INTERACTION
YPD.L.SHIFT
HL.L.INTERACTION
CSH.L.INTERACTION
HL.AS.L.INTERACTION
YPEG.L.SHIFT
HLEG.L.INTERACTION
CSHEG.L.INTERACTION
HLEG.AS.L.INTERACTION
Type of Media

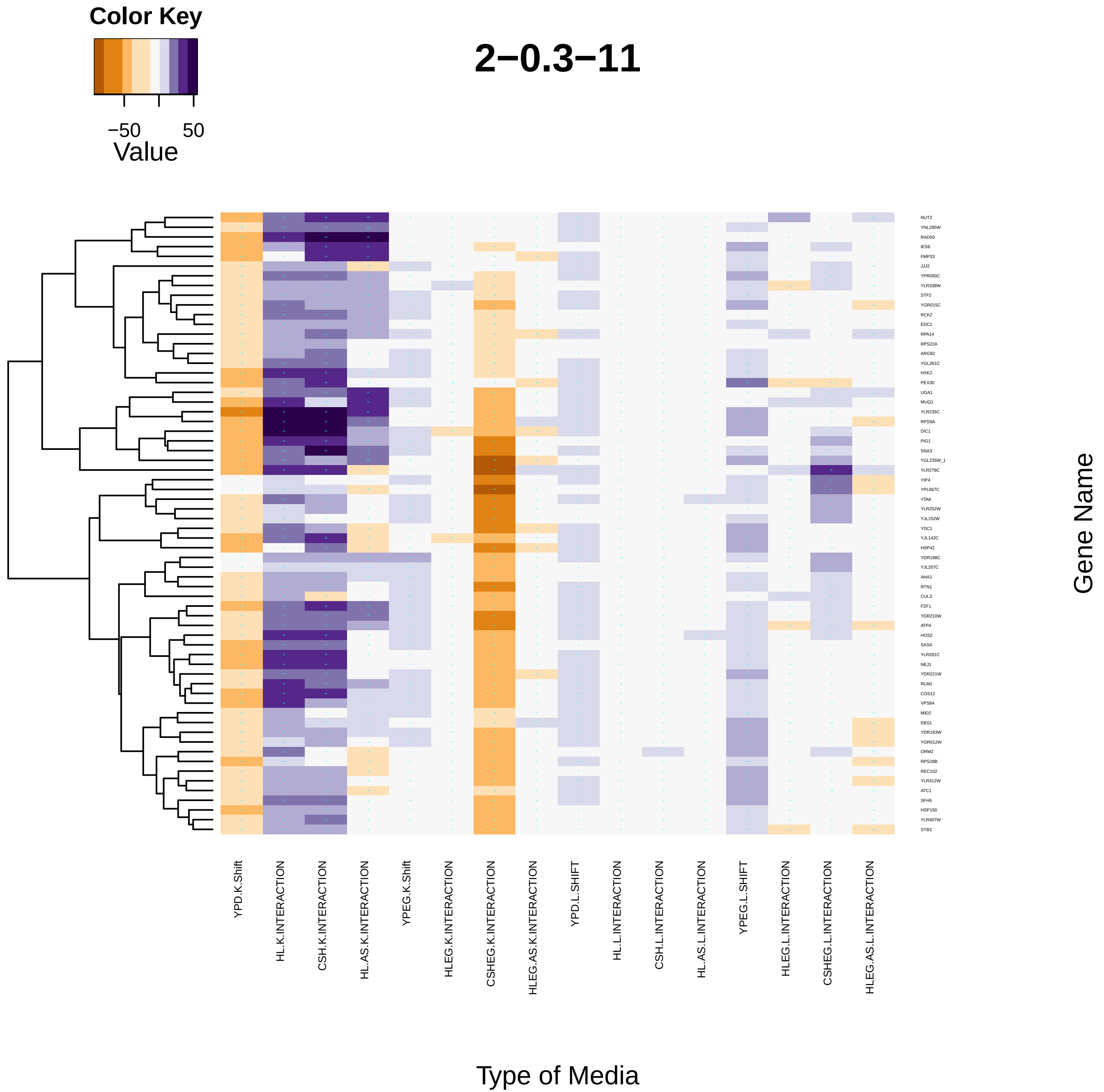
Color Key
−50
50
Value
2−0.3−11
NUT2
YNL295W
RAD50
IES6
FMP33
JJJ2
YPR050C
YLR338W
STF2
YGR015C
RCK2
EDC1
RPA14
RPS22A
ARG82
YGL261C
HXK2
PEX30
UGA1
MUQ1
YLR235C
RPS9A
DIC1
PIG1
SNA3
YGL235W_1
YLR278C
YIP4
YPL067C
YTA6
YLR252W
YJL152W
YDC1
YJL142C
HSP42
YDR198C
YJL207C
AHA1
RTN1
CUL3
FZF1
YDR210W
ATP4
HOS2
SAS4
YLR281C
NEJ1
YDR221W
RLM1
COS12
VPS64
MID2
EBS1
YDR193W
YGR012W
ORM2
RPS28B
REC102
YLR412W
ATC1
SFH5
HSP150
YLR407W
STB3
Gene Name
YPD.K.Shift
HL.K.INTERACTION
CSH.K.INTERACTION
HL.AS.K.INTERACTION
YPEG.K.Shift
HLEG.K.INTERACTION
CSHEG.K.INTERACTION
HLEG.AS.K.INTERACTION
YPD.L.SHIFT
HL.L.INTERACTION
CSH.L.INTERACTION
HL.AS.L.INTERACTION
YPEG.L.SHIFT
HLEG.L.INTERACTION
CSHEG.L.INTERACTION
HLEG.AS.L.INTERACTION
Type of Media

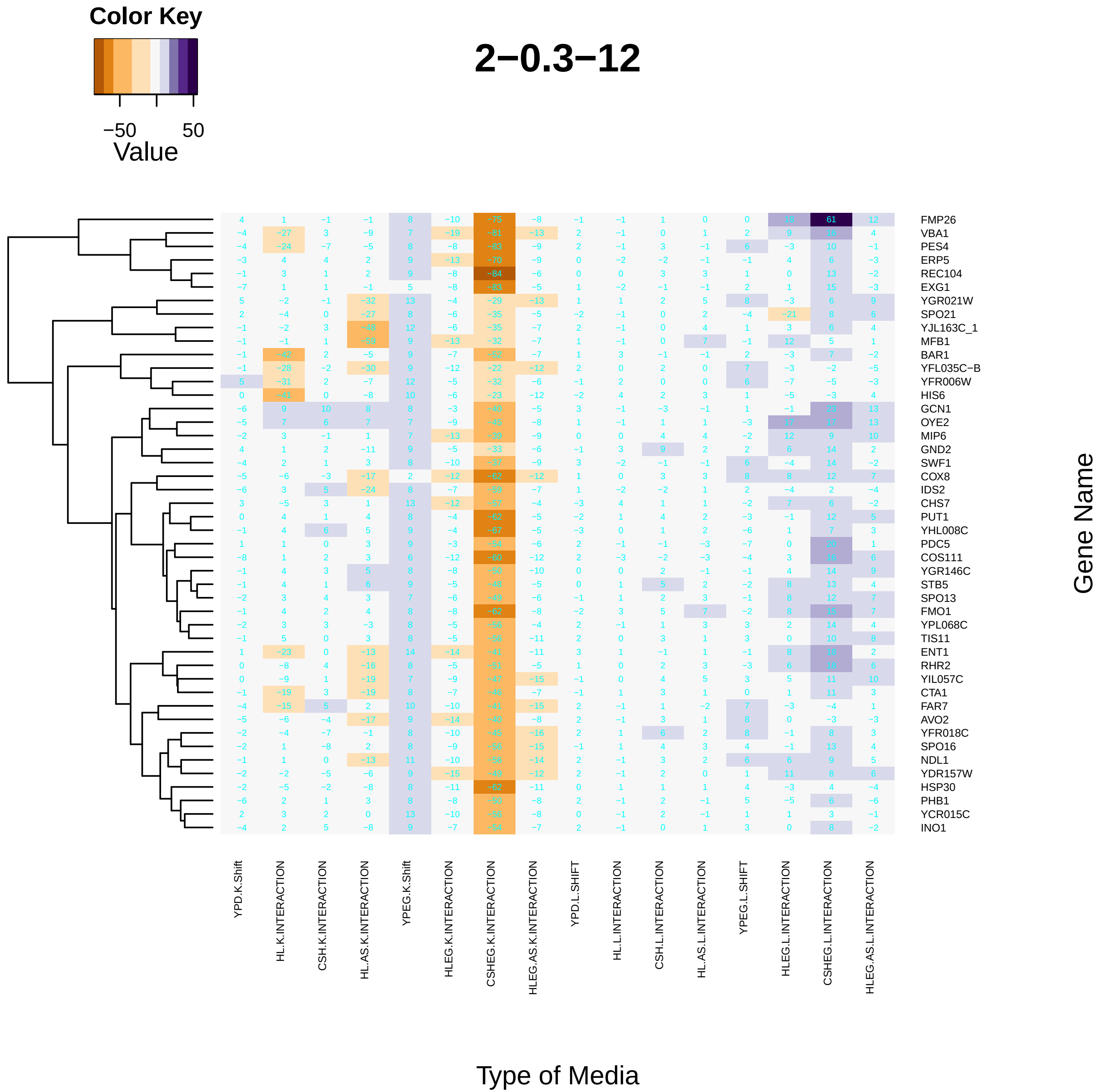
Color Key
−50
50
Value
2−0.3−12
FMP26
VBA1
PES4
ERP5
REC104
EXG1
YGR021W
SPO21
YJL163C_1
MFB1
BAR1
YFL035C−B
YFR006W
HIS6
GCN1
OYE2
MIP6
GND2
SWF1
COX8
IDS2
CHS7
PUT1
YHL008C
PDC5
COS111
YGR146C
STB5
SPO13
FMO1
YPL068C
TIS11
ENT1
RHR2
YIL057C
CTA1
FAR7
AVO2
YFR018C
SPO16
NDL1
YDR157W
HSP30
PHB1
YCR015C
INO1
Gene Name
YPD.K.Shift
HL.K.INTERACTION
CSH.K.INTERACTION
HL.AS.K.INTERACTION
YPEG.K.Shift
HLEG.K.INTERACTION
CSHEG.K.INTERACTION
HLEG.AS.K.INTERACTION
YPD.L.SHIFT
HL.L.INTERACTION
CSH.L.INTERACTION
HL.AS.L.INTERACTION
YPEG.L.SHIFT
HLEG.L.INTERACTION
CSHEG.L.INTERACTION
HLEG.AS.L.INTERACTION
Type of Media

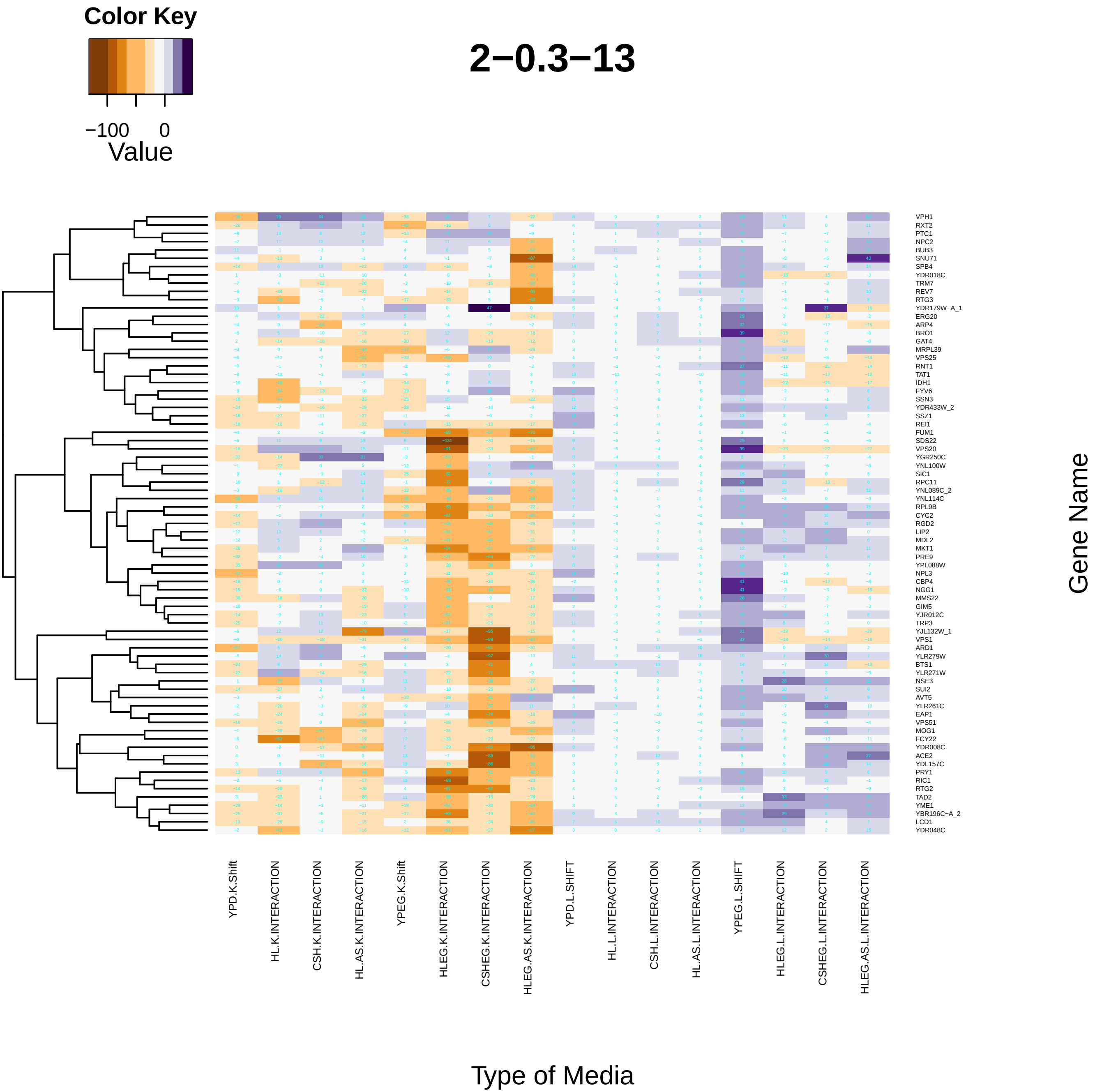
Color Key
−100
0
Value
2−0.3−13
VPH1
RXT2
PTC1
NPC2
BUB3
SNU71
SPB4
YDR018C
TRM7
REV7
RTG3
YDR179W−A_1
ERG20
ARP4
BRO1
GAT4
MRPL39
VPS25
RNT1
TAT1
IDH1
FYV6
SSN3
YDR433W_2
SSZ1
REI1
FUM1
SDS22
VPS20
YGR250C
YNL100W
SIC1
RPC11
YNL089C_2
YNL114C
RPL9B
CYC2
RGD2
LIP2
MDL2
MKT1
PRE9
YPL088W
NPL3
CBP4
NGG1
MMS22
GIM5
YJR012C
TRP3
YJL132W_1
VPS1
ARD1
YLR279W
BTS1
YLR271W
NSE3
SUI2
AVT5
YLR261C
EAP1
VPS51
MOG1
FCY22
YDR008C
ACE2
YDL157C
PRY1
RIC1
RTG2
TAD2
YME1
YBR196C−A_2
LCD1
YDR048C
YPD.K.Shift
HL.K.INTERACTION
CSH.K.INTERACTION
HL.AS.K.INTERACTION
YPEG.K.Shift
HLEG.K.INTERACTION
CSHEG.K.INTERACTION
HLEG.AS.K.INTERACTION
YPD.L.SHIFT
HL.L.INTERACTION
CSH.L.INTERACTION
HL.AS.L.INTERACTION
YPEG.L.SHIFT
HLEG.L.INTERACTION
CSHEG.L.INTERACTION
HLEG.AS.L.INTERACTION
Gene Name
Type of Media

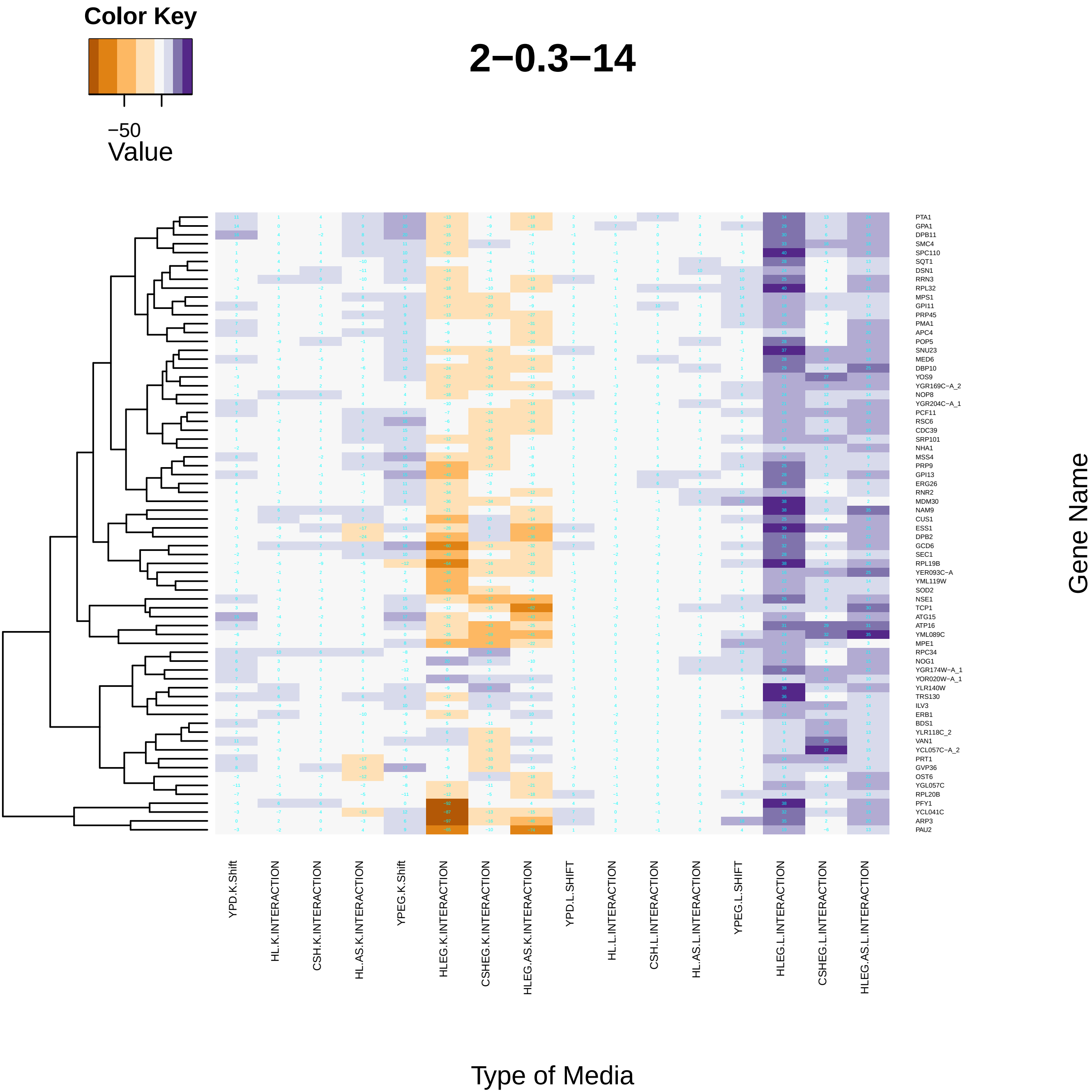

Color Key
−50
Value
2−0.3−14
PTA1
GPA1
DPB11
SMC4
SPC110
SQT1
DSN1
RRN3
RPL32
MPS1
GPI11
PRP45
PMA1
APC4
POP5
SNU23
MED6
DBP10
YOS9
YGR169C−A_2
NOP8
YGR204C−A_1
PCF11
RSC6
CDC39
SRP101
NHA1
MSS4
PRP9
GPI13
ERG26
RNR2
MDM30
NAM9
CUS1
ESS1
DPB2
GCD6
SEC1
RPL19B
YER093C−A
YML119W
SOD2
NSE1
TCP1
ATG15
ATP16
YML089C
MPE1
RPC34
NOG1
YGR174W−A_1
YOR020W−A_1
YLR140W
TRS130
ILV3
ERB1
BDS1
YLR118C_2
VAN1
YCL057C−A_2
PRT1
GVP36
OST6
YGL057C
RPL20B
PFY1
YCL041C
ARP3
PAU2
Gene Name
YPD.K.Shift
HL.K.INTERACTION
CSH.K.INTERACTION
HL.AS.K.INTERACTION
YPEG.K.Shift
HLEG.K.INTERACTION
CSHEG.K.INTERACTION
HLEG.AS.K.INTERACTION
YPD.L.SHIFT
HL.L.INTERACTION
CSH.L.INTERACTION
HL.AS.L.INTERACTION
YPEG.L.SHIFT
HLEG.L.INTERACTION
CSHEG.L.INTERACTION
HLEG.AS.L.INTERACTION
Type of Media

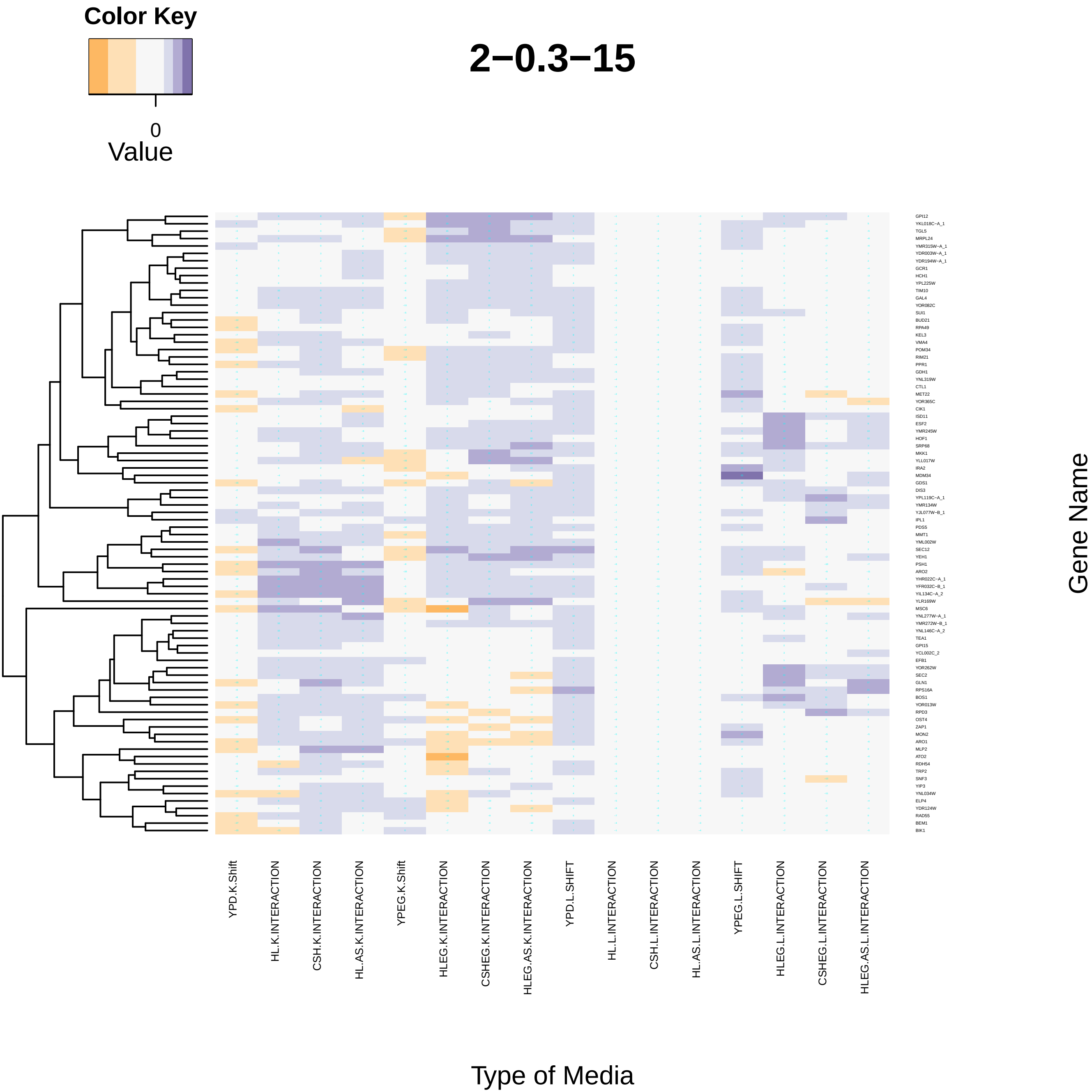

Color Key
0
Value
2−0.3−15
GPI12
YKL018C−A_1
TGL5
MRPL24
YMR315W−A_1
YDR003W−A_1
YDR194W−A_1
GCR1
HCH1
YPL225W
TIM10
GAL4
YOR082C
SUI1
BUD21
RPA49
KEL3
VMA4
POM34
RIM21
PPR1
GDH1
YNL319W
CTL1
MET22
YOR365C
CIK1
ISD11
ESF2
YMR245W
HOF1
SRP68
MKK1
YLL017W
IRA2
MDM34
GDS1
DIS3
YPL119C−A_1
YMR134W
YJL077W−B_1
IPL1
PDS5
MMT1
YML002W
SEC12
YEH1
PSH1
ARO2
YHR022C−A_1
YFR032C−B_1
YIL134C−A_2
YLR169W
MSC6
YNL277W−A_1
YMR272W−B_1
YNL146C−A_2
TEA1
GPI15
YCL002C_2
EFB1
YOR262W
SEC2
GLN1
RPS16A
BOS1
YOR013W
RPD3
OST4
ZAP1
MON2
ARO1
MLP2
ATO2
RDH54
TRP2
SNF3
YIP3
YNL034W
ELP4
YDR124W
RAD55
BEM1
BIK1
Gene Name
YPD.K.Shift
HL.K.INTERACTION
CSH.K.INTERACTION
HL.AS.K.INTERACTION
YPEG.K.Shift
HLEG.K.INTERACTION
CSHEG.K.INTERACTION
HLEG.AS.K.INTERACTION
YPD.L.SHIFT
HL.L.INTERACTION
CSH.L.INTERACTION
HL.AS.L.INTERACTION
YPEG.L.SHIFT
HLEG.L.INTERACTION
CSHEG.L.INTERACTION
HLEG.AS.L.INTERACTION
Type of Media

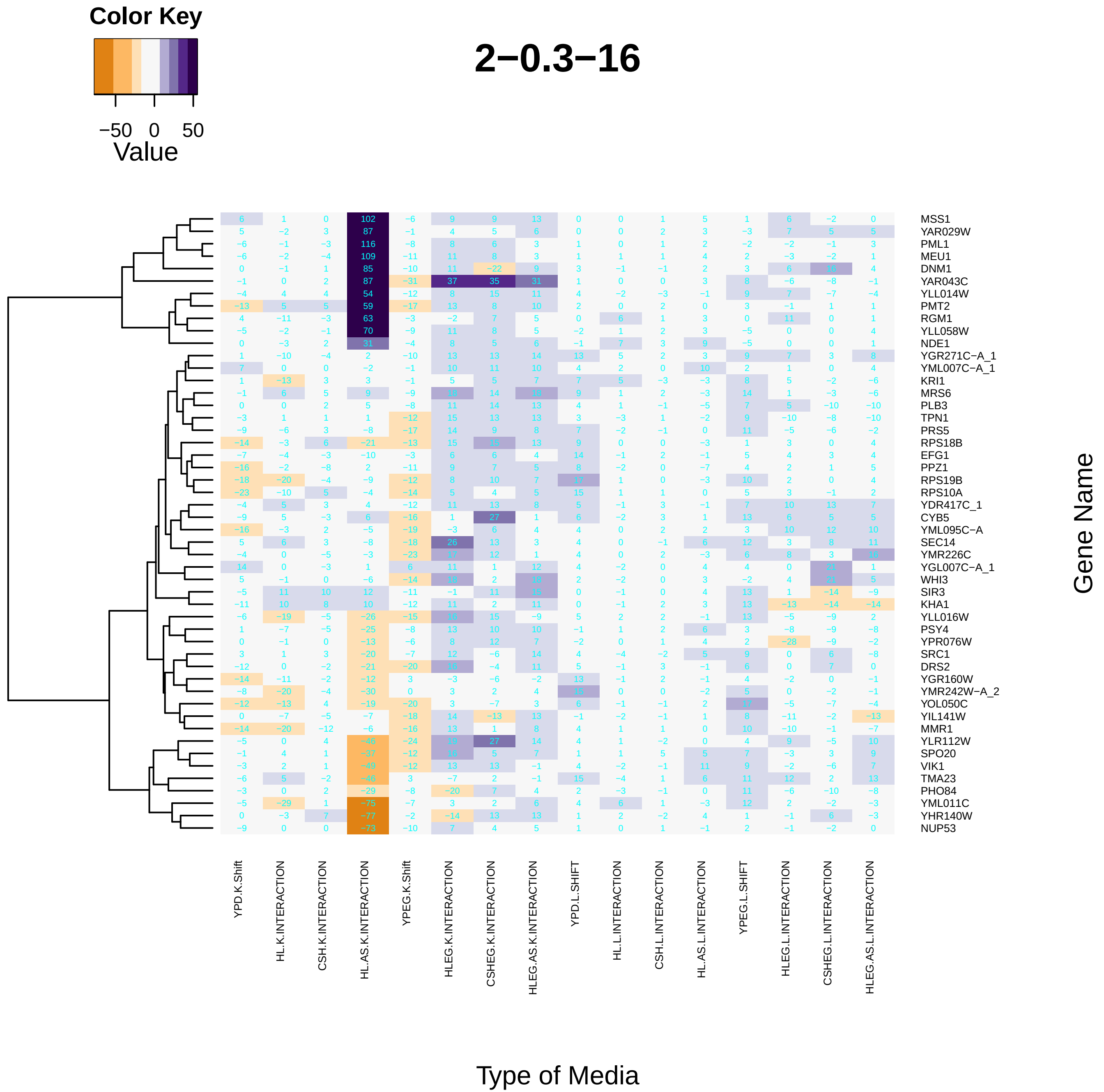

Color Key
−50 0 50
Value
2−0.3−16
MSS1
YAR029W
PML1
MEU1
DNM1
YAR043C
YLL014W
PMT2
RGM1
YLL058W
NDE1
YGR271C−A_1
YML007C−A_1
KRI1
MRS6
PLB3
TPN1
PRS5
RPS18B
EFG1
PPZ1
RPS19B
RPS10A
YDR417C_1
CYB5
YML095C−A
SEC14
YMR226C
YGL007C−A_1
WHI3
SIR3
KHA1
YLL016W
PSY4
YPR076W
SRC1
DRS2
YGR160W
YMR242W−A_2
YOL050C
YIL141W
MMR1
YLR112W
SPO20
VIK1
TMA23
PHO84
YML011C
YHR140W
NUP53
Gene Name
YPD.K.Shift
HL.K.INTERACTION
CSH.K.INTERACTION
HL.AS.K.INTERACTION
YPEG.K.Shift
HLEG.K.INTERACTION
CSHEG.K.INTERACTION
HLEG.AS.K.INTERACTION
YPD.L.SHIFT
HL.L.INTERACTION
CSH.L.INTERACTION
HL.AS.L.INTERACTION
YPEG.L.SHIFT
HLEG.L.INTERACTION
CSHEG.L.INTERACTION
HLEG.AS.L.INTERACTION
Type of Media

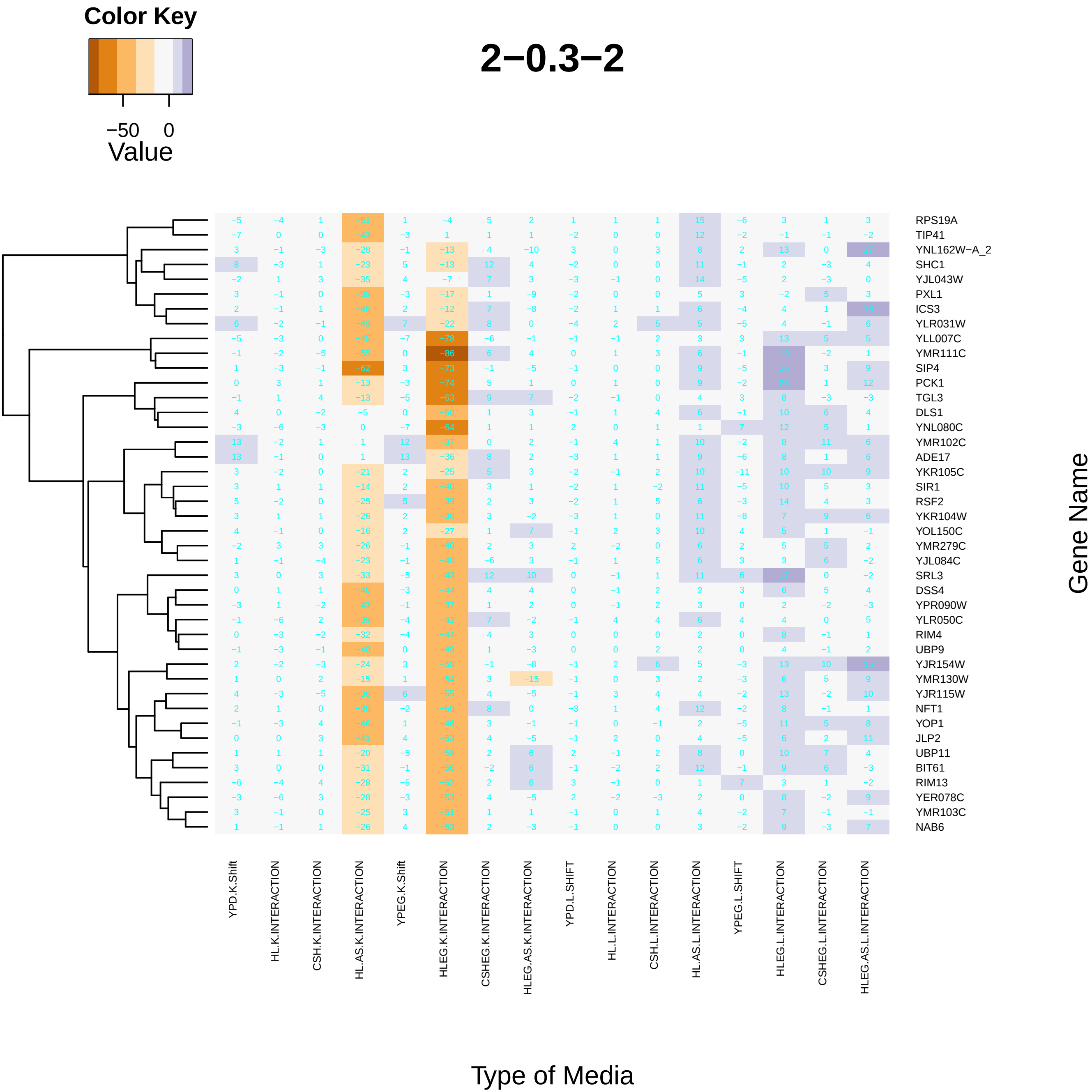

Color Key
−50
0
Value
2−0.3−2
RPS19A
TIP41
YNL162W−A_2
SHC1
YJL043W
PXL1
ICS3
YLR031W
YLL007C
YMR111C
SIP4
PCK1
TGL3
DLS1
YNL080C
YMR102C
ADE17
YKR105C
SIR1
RSF2
YKR104W
YOL150C
YMR279C
YJL084C
SRL3
DSS4
YPR090W
YLR050C
RIM4
UBP9
YJR154W
YMR130W
YJR115W
NFT1
YOP1
JLP2
UBP11
BIT61
RIM13
YER078C
YMR103C
NAB6
Gene Name
YPD.K.Shift
HL.K.INTERACTION
CSH.K.INTERACTION
HL.AS.K.INTERACTION
YPEG.K.Shift
HLEG.K.INTERACTION
CSHEG.K.INTERACTION
HLEG.AS.K.INTERACTION
YPD.L.SHIFT
HL.L.INTERACTION
CSH.L.INTERACTION
HL.AS.L.INTERACTION
YPEG.L.SHIFT
HLEG.L.INTERACTION
CSHEG.L.INTERACTION
HLEG.AS.L.INTERACTION
Type of Media

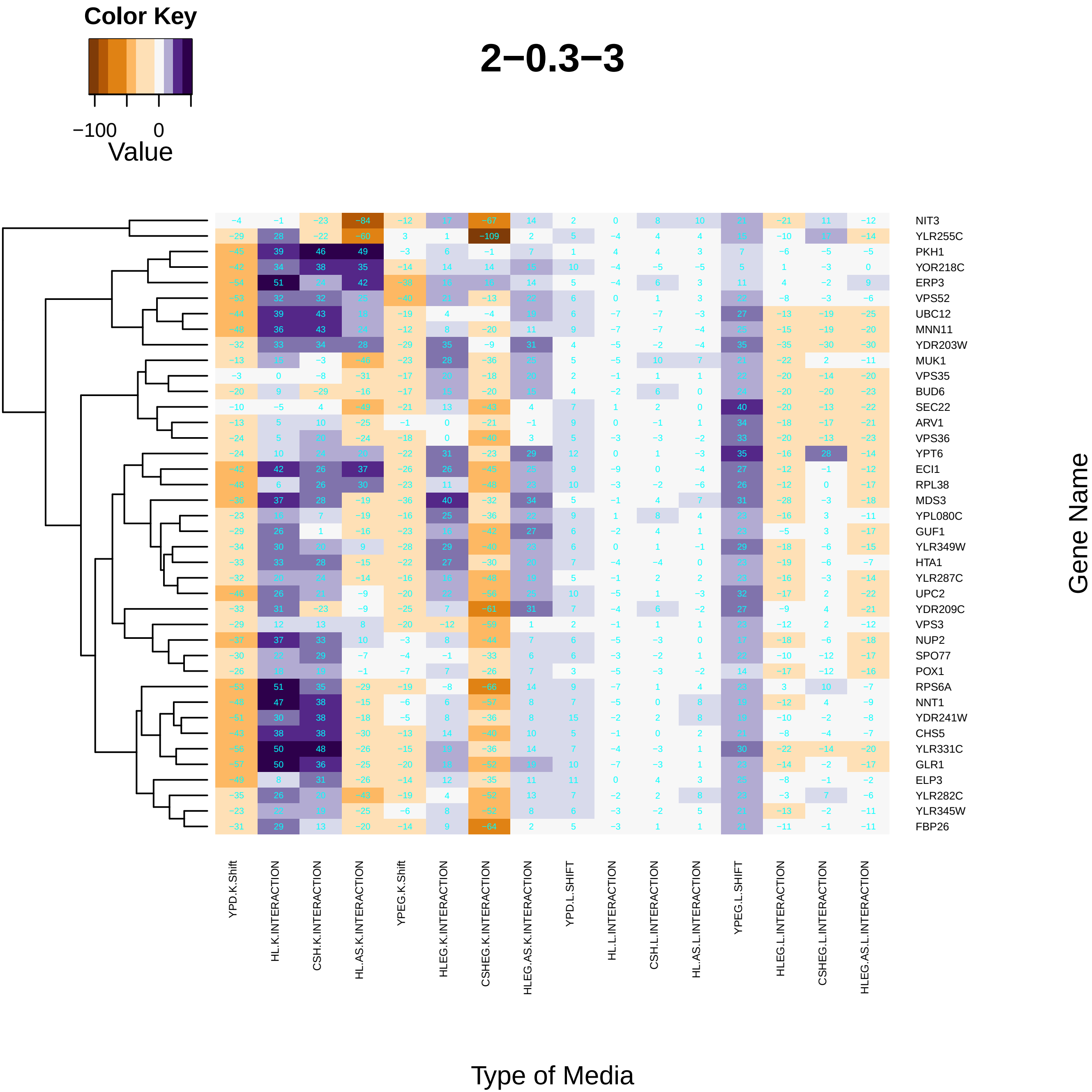
Color Key
−100
0
Value
2−0.3−3
NIT3
YLR255C
PKH1
YOR218C
ERP3
VPS52
UBC12
MNN11
YDR203W
MUK1
VPS35
BUD6
SEC22
ARV1
VPS36
YPT6
ECI1
RPL38
MDS3
YPL080C
GUF1
YLR349W
HTA1
YLR287C
UPC2
YDR209C
VPS3
NUP2
SPO77
POX1
RPS6A
NNT1
YDR241W
CHS5
YLR331C
GLR1
ELP3
YLR282C
YLR345W
FBP26
Gene Name
YPD.K.Shift
HL.K.INTERACTION
CSH.K.INTERACTION
HL.AS.K.INTERACTION
YPEG.K.Shift
HLEG.K.INTERACTION
CSHEG.K.INTERACTION
HLEG.AS.K.INTERACTION
YPD.L.SHIFT
HL.L.INTERACTION
CSH.L.INTERACTION
HL.AS.L.INTERACTION
YPEG.L.SHIFT
HLEG.L.INTERACTION
CSHEG.L.INTERACTION
HLEG.AS.L.INTERACTION
Type of Media

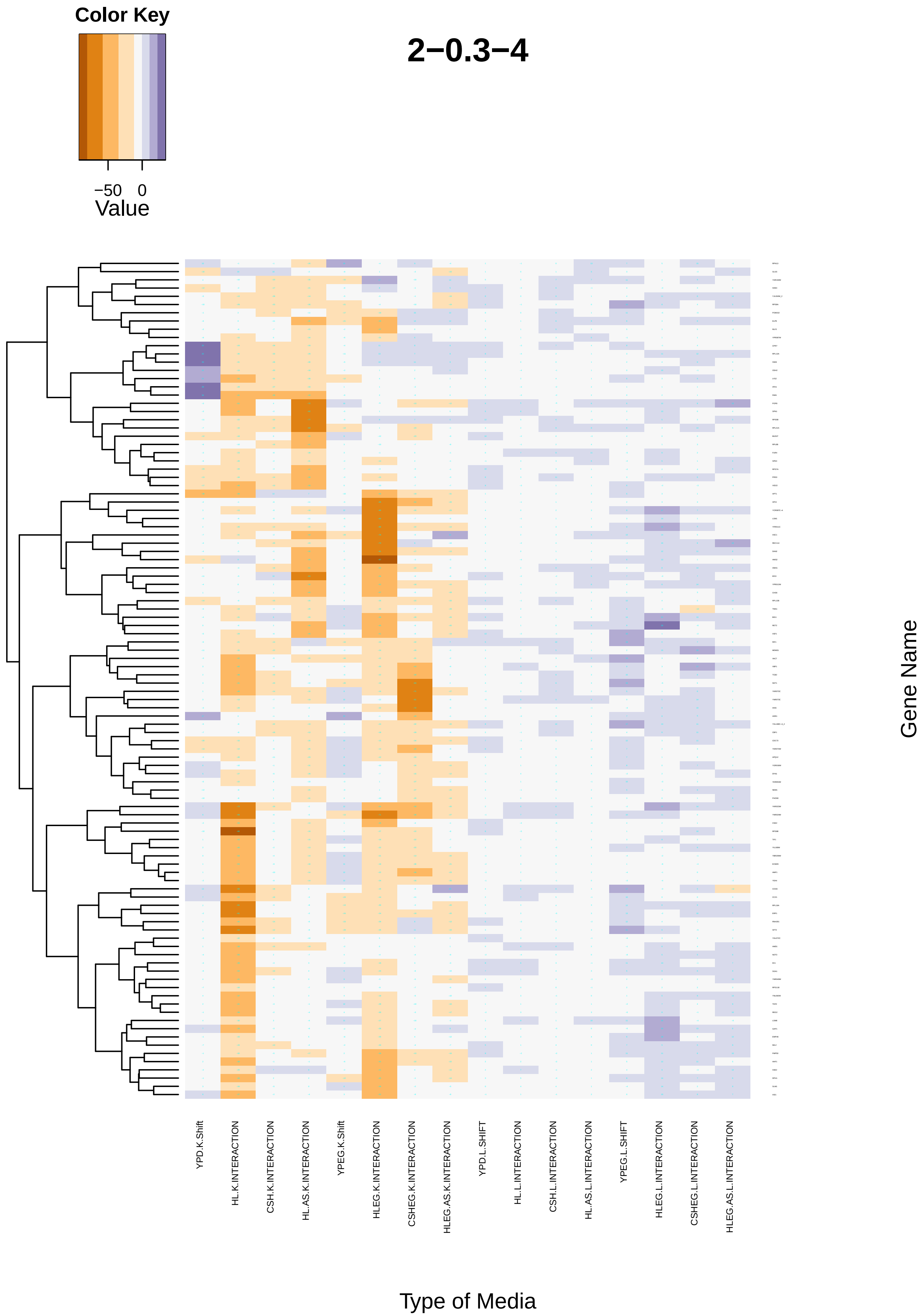
Color Key
−50
0
Value
2−0.3−4
Gene Name
YPD.K.Shift
HL.K.INTERACTION
CSH.K.INTERACTION
HL.AS.K.INTERACTION
YPEG.K.Shift
HLEG.K.INTERACTION
CSHEG.K.INTERACTION
HLEG.AS.K.INTERACTION
YPD.L.SHIFT
HL.L.INTERACTION
CSH.L.INTERACTION
HL.AS.L.INTERACTION
YPEG.L.SHIFT
HLEG.L.INTERACTION
CSHEG.L.INTERACTION
HLEG.AS.L.INTERACTION
Type of Media

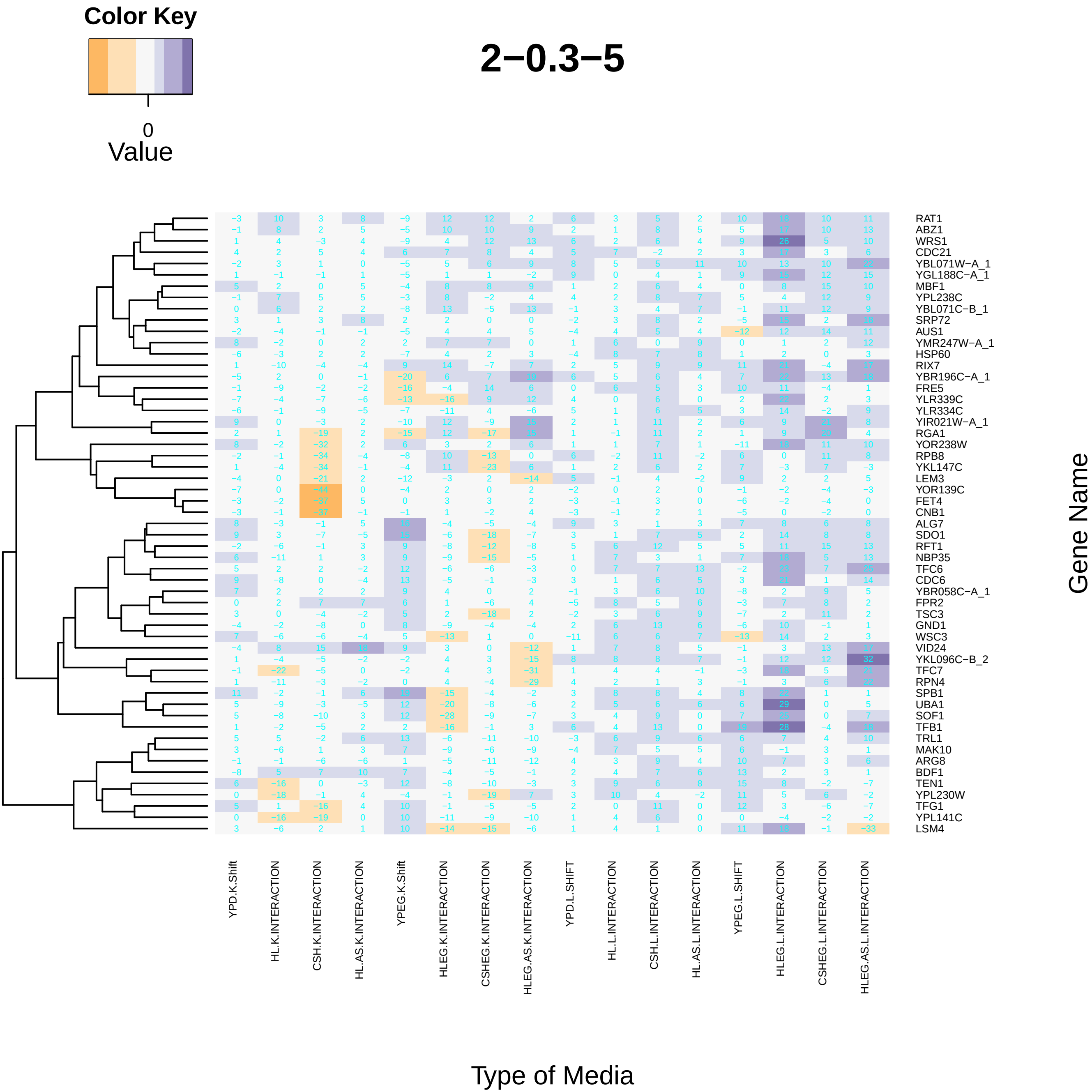
Color Key
0
Value
2−0.3−5
RAT1
ABZ1
WRS1
CDC21
YBL071W−A_1
YGL188C−A_1
MBF1
YPL238C
YBL071C−B_1
SRP72
AUS1
YMR247W−A_1
HSP60
RIX7
YBR196C−A_1
FRE5
YLR339C
YLR334C
YIR021W−A_1
RGA1
YOR238W
RPB8
YKL147C
LEM3
YOR139C
FET4
CNB1
ALG7
SDO1
RFT1
NBP35
TFC6
CDC6
YBR058C−A_1
FPR2
TSC3
GND1
WSC3
VID24
YKL096C−B_2
TFC7
RPN4
SPB1
UBA1
SOF1
TFB1
TRL1
MAK10
ARG8
BDF1
TEN1
YPL230W
TFG1
YPL141C
LSM4
Gene Name
YPD.K.Shift
HL.K.INTERACTION
CSH.K.INTERACTION
HL.AS.K.INTERACTION
YPEG.K.Shift
HLEG.K.INTERACTION
CSHEG.K.INTERACTION
HLEG.AS.K.INTERACTION
YPD.L.SHIFT
HL.L.INTERACTION
CSH.L.INTERACTION
HL.AS.L.INTERACTION
YPEG.L.SHIFT
HLEG.L.INTERACTION
CSHEG.L.INTERACTION
HLEG.AS.L.INTERACTION
Type of Media

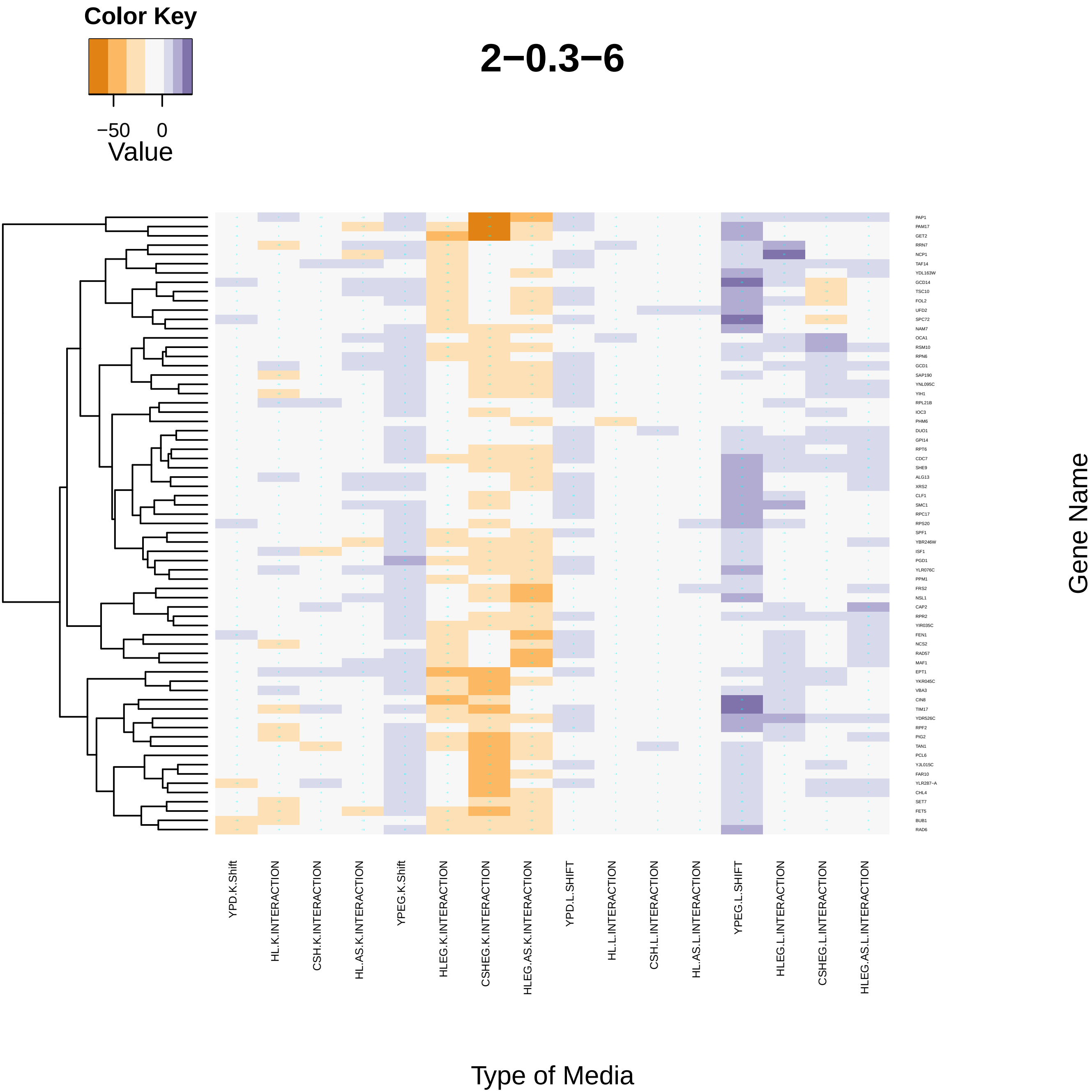

Color Key
−50
0
Value
2−0.3−6
PAP1
PAM17
GET2
RRN7
NCP1
TAF14
YDL163W
GCD14
TSC10
FOL2
UFD2
SPC72
NAM7
OCA1
RSM10
RPN6
GCD1
SAP190
YNL095C
YIH1
RPL21B
IOC3
PHM6
DUO1
GPI14
RPT6
CDC7
SHE9
ALG13
XRS2
CLF1
SMC1
RPC17
RPS20
SPF1
YBR246W
ISF1
PGD1
YLR076C
PPM1
FRS2
NSL1
CAP2
RPR2
YIR035C
FEN1
NCS2
RAD57
MAF1
EPT1
YKR045C
VBA3
CIN8
TIM17
YDR526C
RPF2
PIG2
TAN1
PCL6
YJL015C
FAR10
YLR287−A
CHL4
SET7
FET5
BUB1
RAD6
Gene Name
YPD.K.Shift
HL.K.INTERACTION
CSH.K.INTERACTION
HL.AS.K.INTERACTION
YPEG.K.Shift
HLEG.K.INTERACTION
CSHEG.K.INTERACTION
HLEG.AS.K.INTERACTION
YPD.L.SHIFT
HL.L.INTERACTION
CSH.L.INTERACTION
HL.AS.L.INTERACTION
YPEG.L.SHIFT
HLEG.L.INTERACTION
CSHEG.L.INTERACTION
HLEG.AS.L.INTERACTION
Type of Media

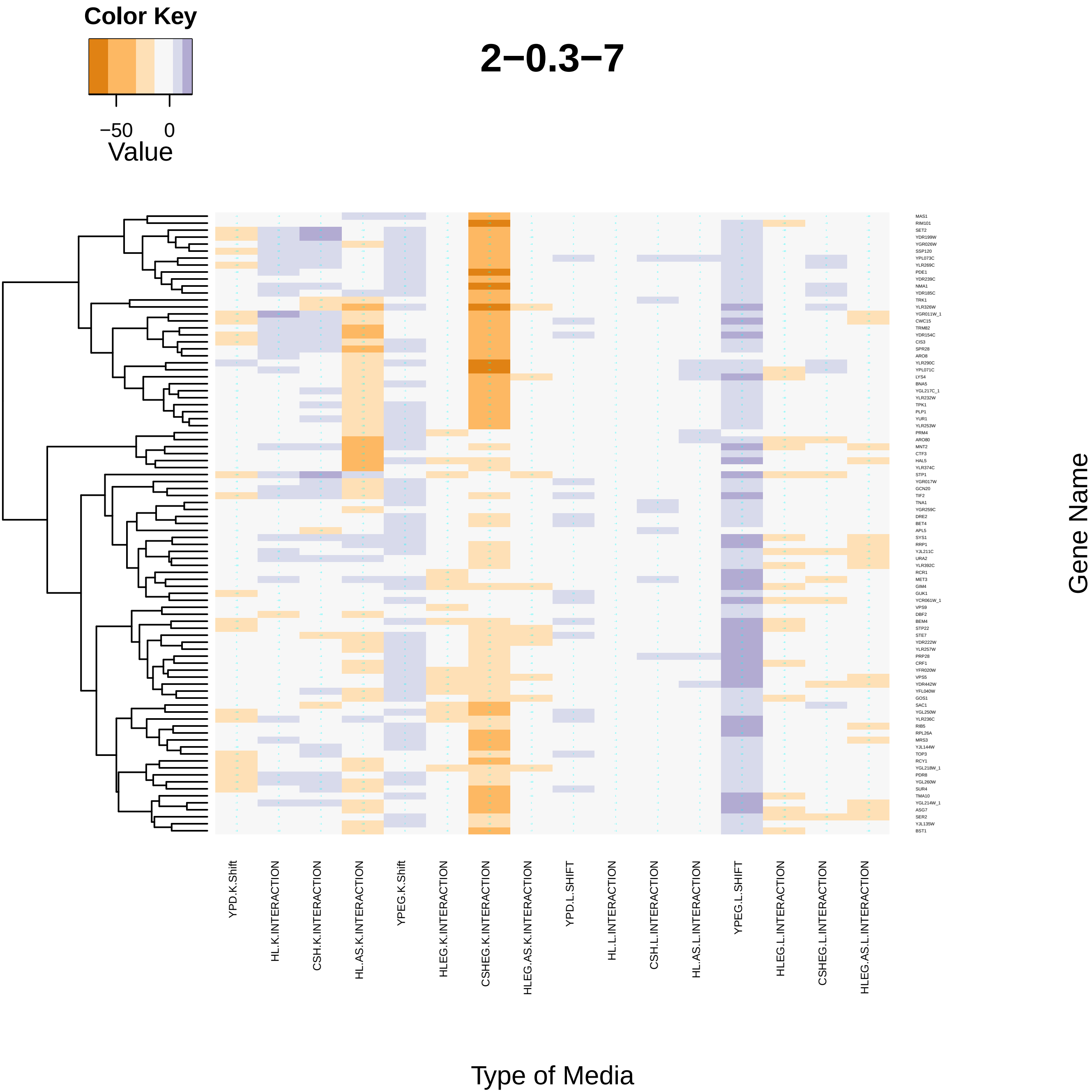
Color Key
−50
0
Value
2−0.3−7
MAS1
RIM101
SET2
YDR199W
YGR026W
SSP120
YPL073C
YLR269C
PDE1
YDR239C
NMA1
YDR185C
TRK1
YLR326W
YGR011W_1
CWC15
TRM82
YDR154C
CIS3
SPR28
ARO8
YLR290C
YPL071C
LYS4
BNA5
YGL217C_1
YLR232W
TPK1
PLP1
YUR1
YLR253W
PRM4
ARO80
MNT2
CTF3
HAL5
YLR374C
STP1
YGR017W
GCN20
TIF2
TNA1
YGR259C
DRE2
BET4
APL5
SYS1
RRP1
YJL211C
URA2
YLR392C
RCR1
MET3
GIM4
GUK1
YCR061W_1
VPS9
DBF2
BEM4
STP22
STE7
YDR222W
YLR257W
PRP28
CRF1
YFR020W
VPS5
YDR442W
YFL040W
GOS1
SAC1
YGL250W
YLR236C
RIB5
RPL26A
MRS3
YJL144W
TOP3
RCY1
YGL218W_1
PDR8
YGL260W
SUR4
TMA10
YGL214W_1
ASG7
SER2
YJL135W
BST1
Gene Name
YPD.K.Shift
HL.K.INTERACTION
CSH.K.INTERACTION
HL.AS.K.INTERACTION
YPEG.K.Shift
HLEG.K.INTERACTION
CSHEG.K.INTERACTION
HLEG.AS.K.INTERACTION
YPD.L.SHIFT
HL.L.INTERACTION
CSH.L.INTERACTION
HL.AS.L.INTERACTION
YPEG.L.SHIFT
HLEG.L.INTERACTION
CSHEG.L.INTERACTION
HLEG.AS.L.INTERACTION
Type of Media

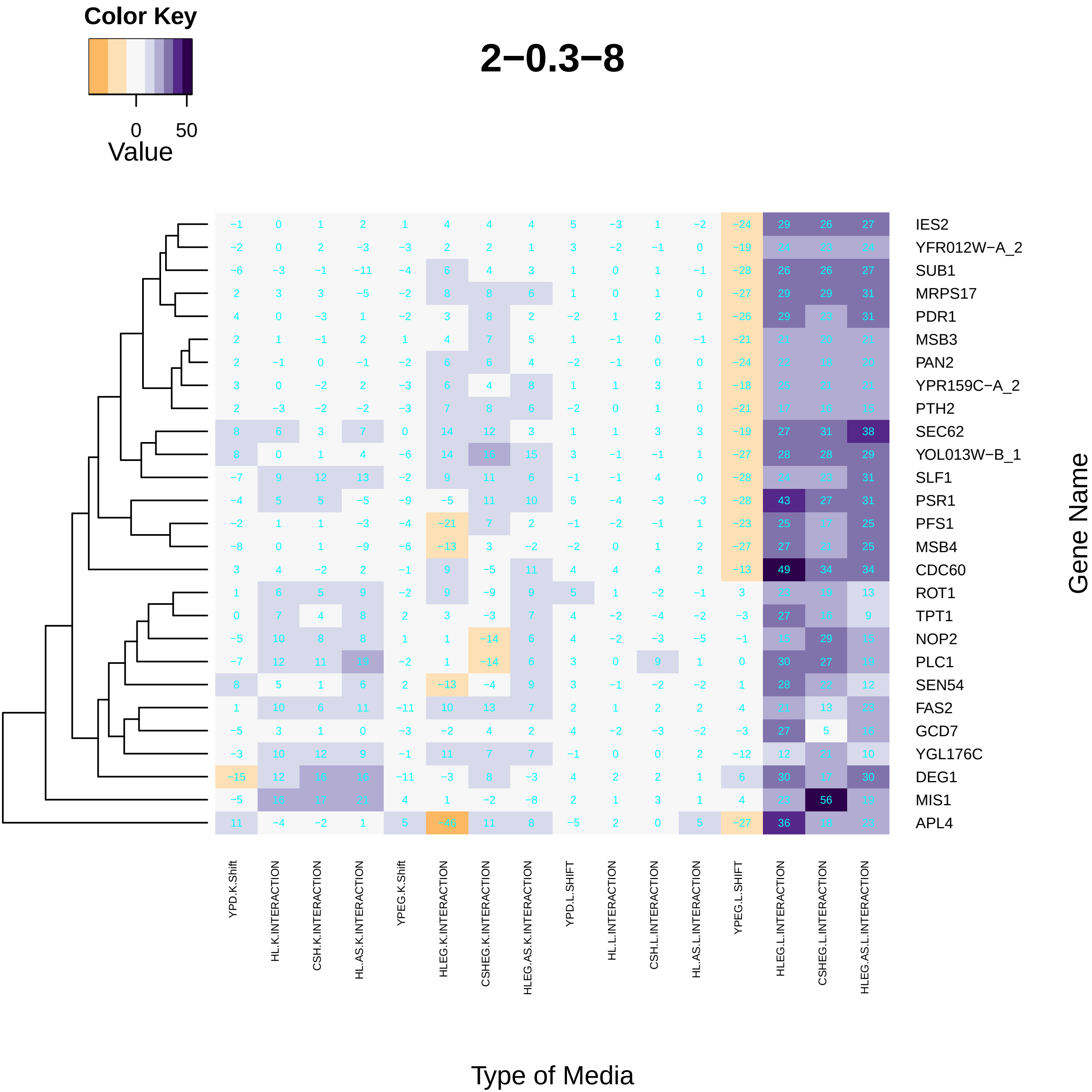
2−0.3−8
Color Key
0
50
Value
IES2
YFR012W−A_2
SUB1
MRPS17
PDR1
MSB3
PAN2
YPR159C−A_2
PTH2
SEC62
YOL013W−B_1
SLF1
PSR1
PFS1
MSB4
CDC60
ROT1
TPT1
NOP2
PLC1
SEN54
FAS2
GCD7
YGL176C
DEG1
MIS1
APL4
Gene Name
YPD.K.Shift
HL.K.INTERACTION
CSH.K.INTERACTION
HL.AS.K.INTERACTION
YPEG.K.Shift
HLEG.K.INTERACTION
CSHEG.K.INTERACTION
HLEG.AS.K.INTERACTION
YPD.L.SHIFT
HL.L.INTERACTION
CSH.L.INTERACTION
HL.AS.L.INTERACTION
YPEG.L.SHIFT
HLEG.L.INTERACTION
CSHEG.L.INTERACTION
HLEG.AS.L.INTERACTION
Type of Media

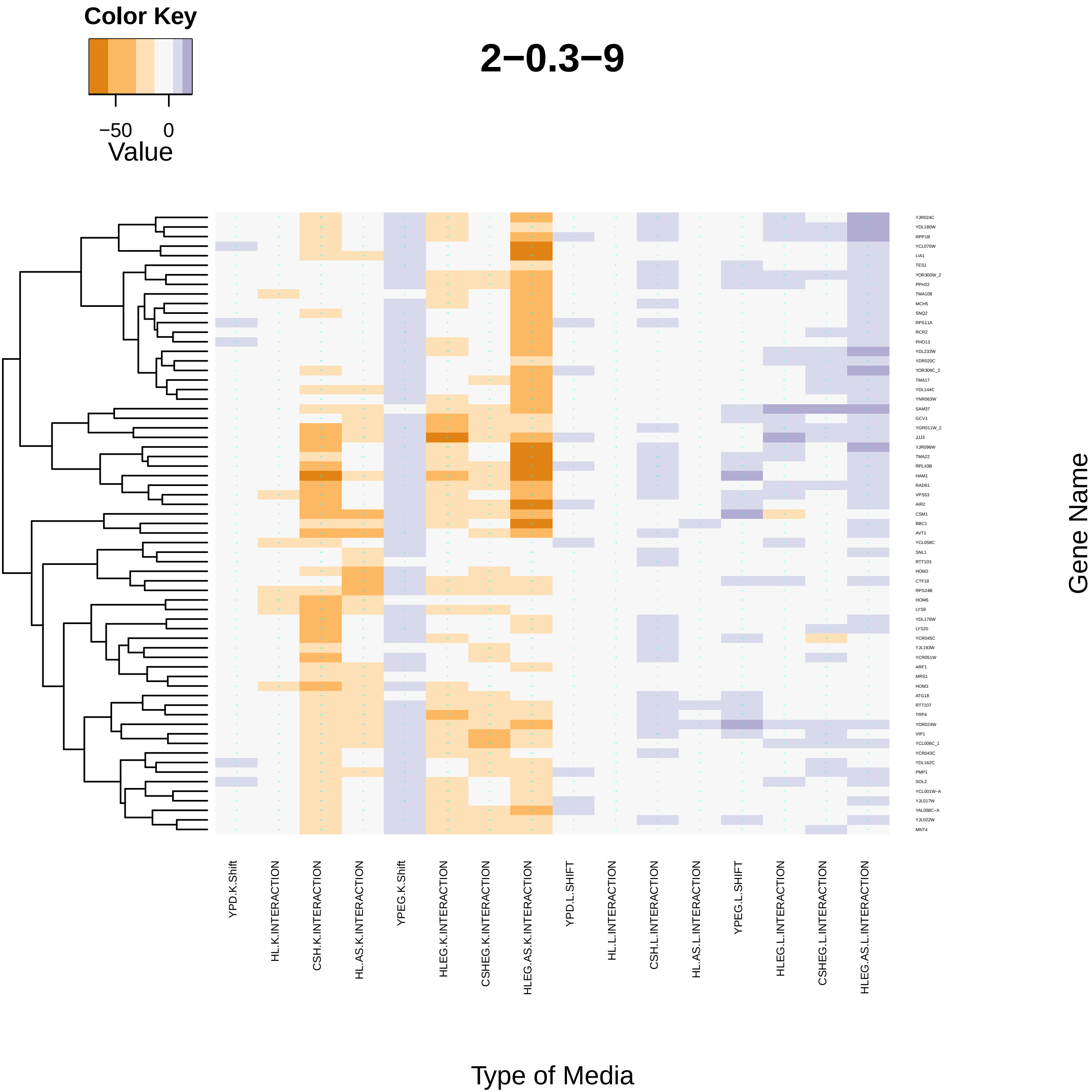

Color Key
−50
0
Value
2−0.3−9
YJR024C
YDL180W
RPP1B
YCL076W
LIA1
TES1
YOR300W_2
PPH22
TMA108
MCH5
SNQ2
RPS11A
RCR2
PHO13
YDL233W
YDR020C
YOR309C_2
TMA17
YDL144C
YNR063W
SAM37
GCV1
YGR011W_2
JJJ3
YJR096W
TMA22
RPL43B
HAM1
RAD61
VPS53
AIR2
CSM1
BBC1
AVT1
YCL058C
SNL1
RTT103
HOM2
CTF18
RPS24B
HOM6
LYS9
YDL176W
LYS20
YCR045C
YJL193W
YCR051W
ARF1
MRS1
HOM3
ATG18
RTT107
TRP4
YDR024W
VIP1
YCL006C_1
YCR043C
YDL162C
PMP1
SOL2
YCL001W−A
YJL017W
YAL058C−A
YJL022W
MNT4
Gene Name
YPD.K.Shift
HL.K.INTERACTION
CSH.K.INTERACTION
HL.AS.K.INTERACTION
YPEG.K.Shift
HLEG.K.INTERACTION
CSHEG.K.INTERACTION
HLEG.AS.K.INTERACTION
YPD.L.SHIFT
HL.L.INTERACTION
CSH.L.INTERACTION
HL.AS.L.INTERACTION
YPEG.L.SHIFT
HLEG.L.INTERACTION
CSHEG.L.INTERACTION
HLEG.AS.L.INTERACTION
Type of Media

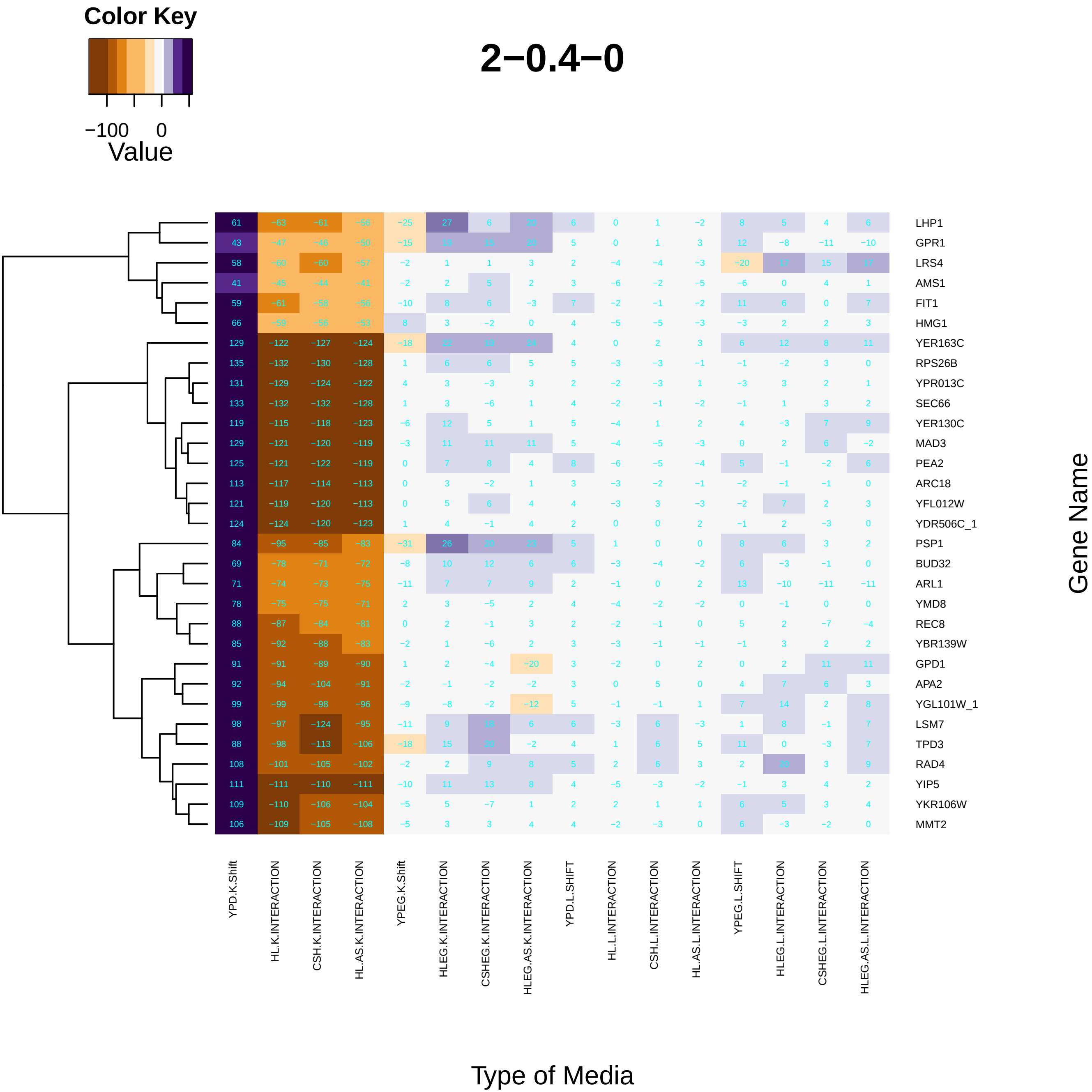

Color Key
−100
0
Value
2−0.4−0
LHP1
GPR1
LRS4
AMS1
FIT1
HMG1
YER163C
RPS26B
YPR013C
SEC66
YER130C
MAD3
PEA2
ARC18
YFL012W
YDR506C_1
PSP1
BUD32
ARL1
YMD8
REC8
YBR139W
GPD1
APA2
YGL101W_1
LSM7
TPD3
RAD4
YIP5
YKR106W
MMT2
Gene Name
YPD.K.Shift
HL.K.INTERACTION
CSH.K.INTERACTION
HL.AS.K.INTERACTION
YPEG.K.Shift
HLEG.K.INTERACTION
CSHEG.K.INTERACTION
HLEG.AS.K.INTERACTION
YPD.L.SHIFT
HL.L.INTERACTION
CSH.L.INTERACTION
HL.AS.L.INTERACTION
YPEG.L.SHIFT
HLEG.L.INTERACTION
CSHEG.L.INTERACTION
HLEG.AS.L.INTERACTION
Type of Media

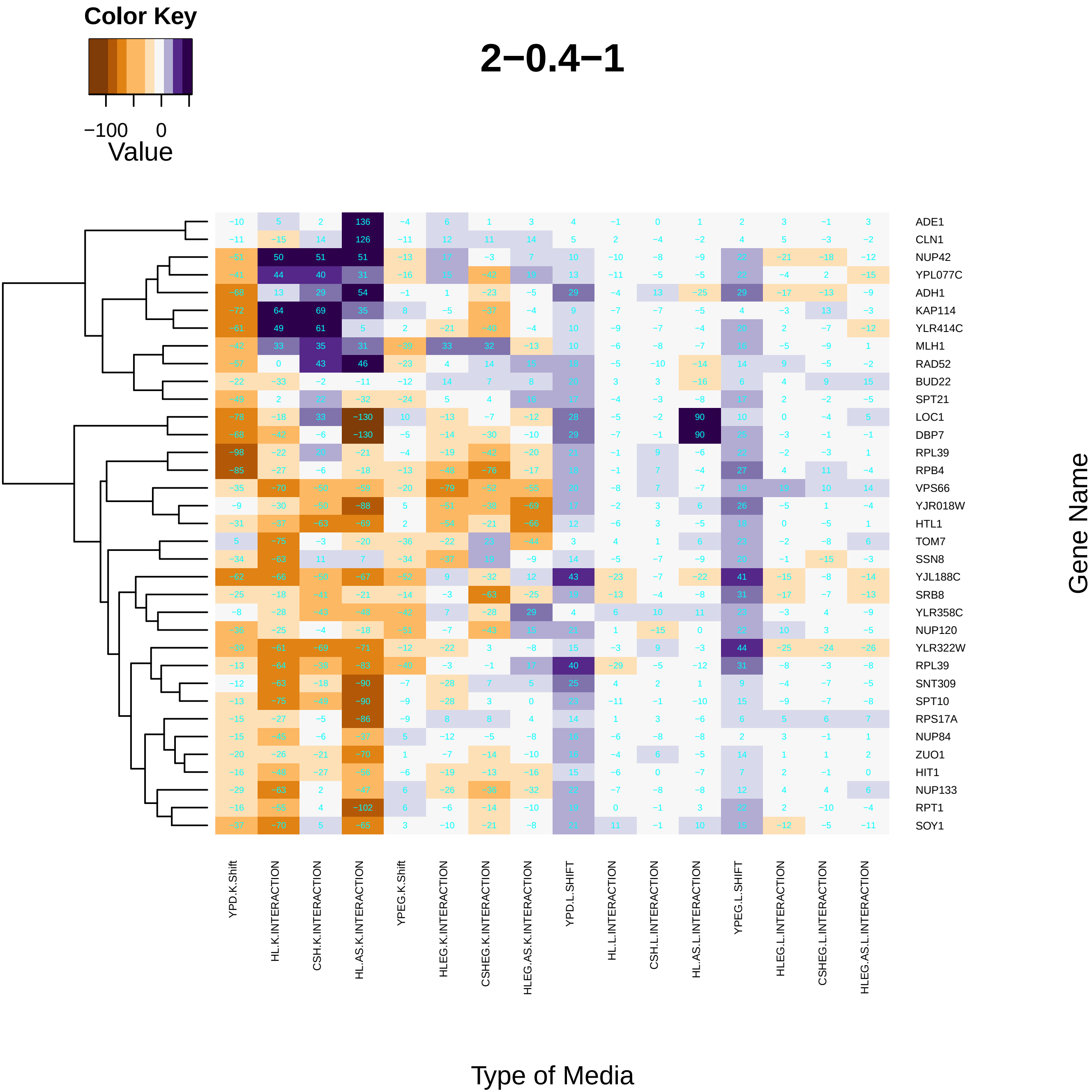

Color Key
−100
0
Value
2−0.4−1
ADE1
CLN1
NUP42
YPL077C
ADH1
KAP114
YLR414C
MLH1
RAD52
BUD22
SPT21
LOC1
DBP7
RPL39
RPB4
VPS66
YJR018W
HTL1
TOM7
SSN8
YJL188C
SRB8
YLR358C
NUP120
YLR322W
RPL39
SNT309
SPT10
RPS17A
NUP84
ZUO1
HIT1
NUP133
RPT1
SOY1
Gene Name
YPD.K.Shift
HL.K.INTERACTION
CSH.K.INTERACTION
HL.AS.K.INTERACTION
YPEG.K.Shift
HLEG.K.INTERACTION
CSHEG.K.INTERACTION
HLEG.AS.K.INTERACTION
YPD.L.SHIFT
HL.L.INTERACTION
CSH.L.INTERACTION
HL.AS.L.INTERACTION
YPEG.L.SHIFT
HLEG.L.INTERACTION
CSHEG.L.INTERACTION
HLEG.AS.L.INTERACTION
Type of Media

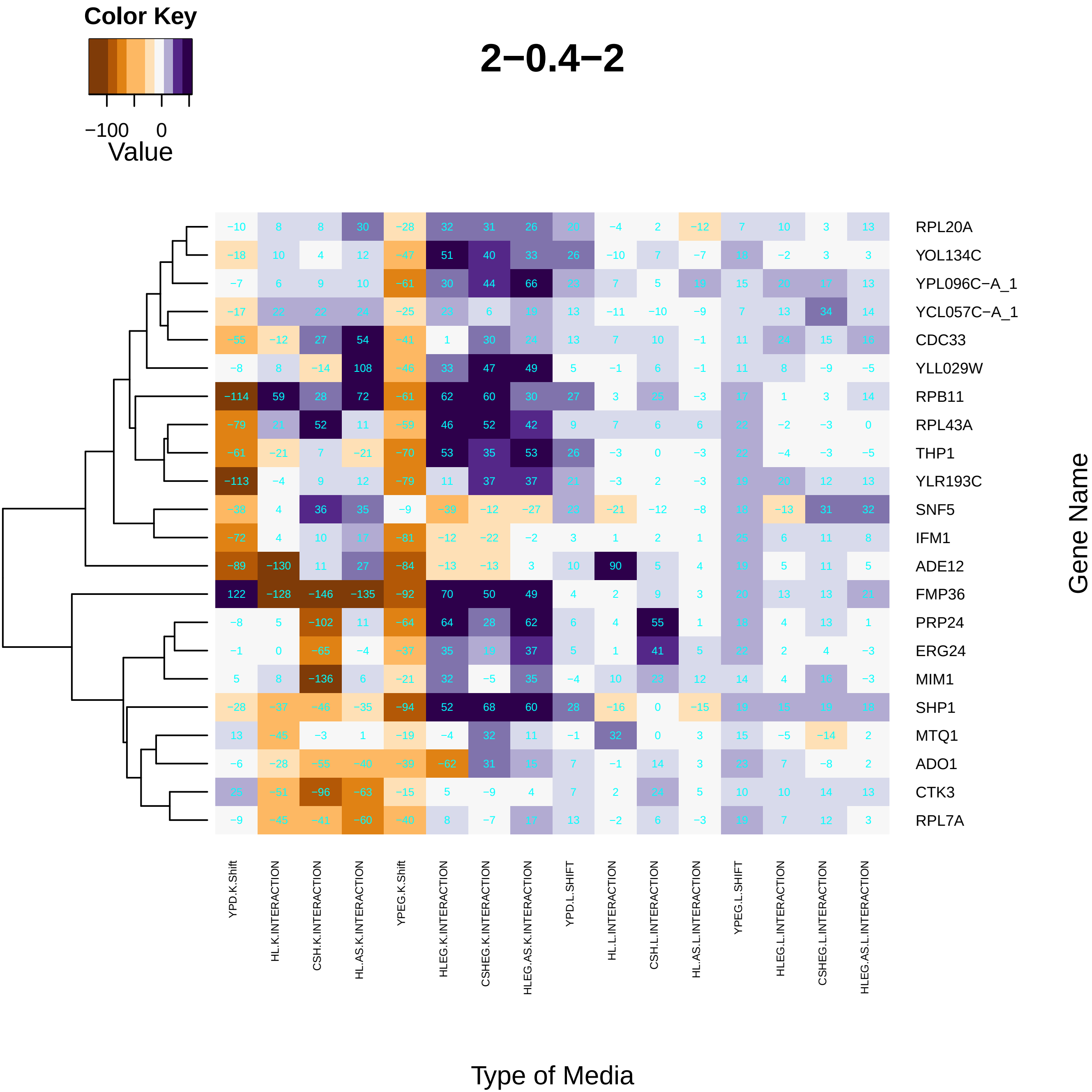
Color Key
−100
0
Value
2−0.4−2
RPL20A
YOL134C
YPL096C−A_1
YCL057C−A_1
CDC33
YLL029W
RPB11
RPL43A
THP1
YLR193C
SNF5
IFM1
ADE12
FMP36
PRP24
ERG24
MIM1
SHP1
MTQ1
ADO1
CTK3
RPL7A
Gene Name
YPD.K.Shift
HL.K.INTERACTION
CSH.K.INTERACTION
HL.AS.K.INTERACTION
YPEG.K.Shift
HLEG.K.INTERACTION
CSHEG.K.INTERACTION
HLEG.AS.K.INTERACTION
YPD.L.SHIFT
HL.L.INTERACTION
CSH.L.INTERACTION
HL.AS.L.INTERACTION
YPEG.L.SHIFT
HLEG.L.INTERACTION
CSHEG.L.INTERACTION
HLEG.AS.L.INTERACTION
Type of Media

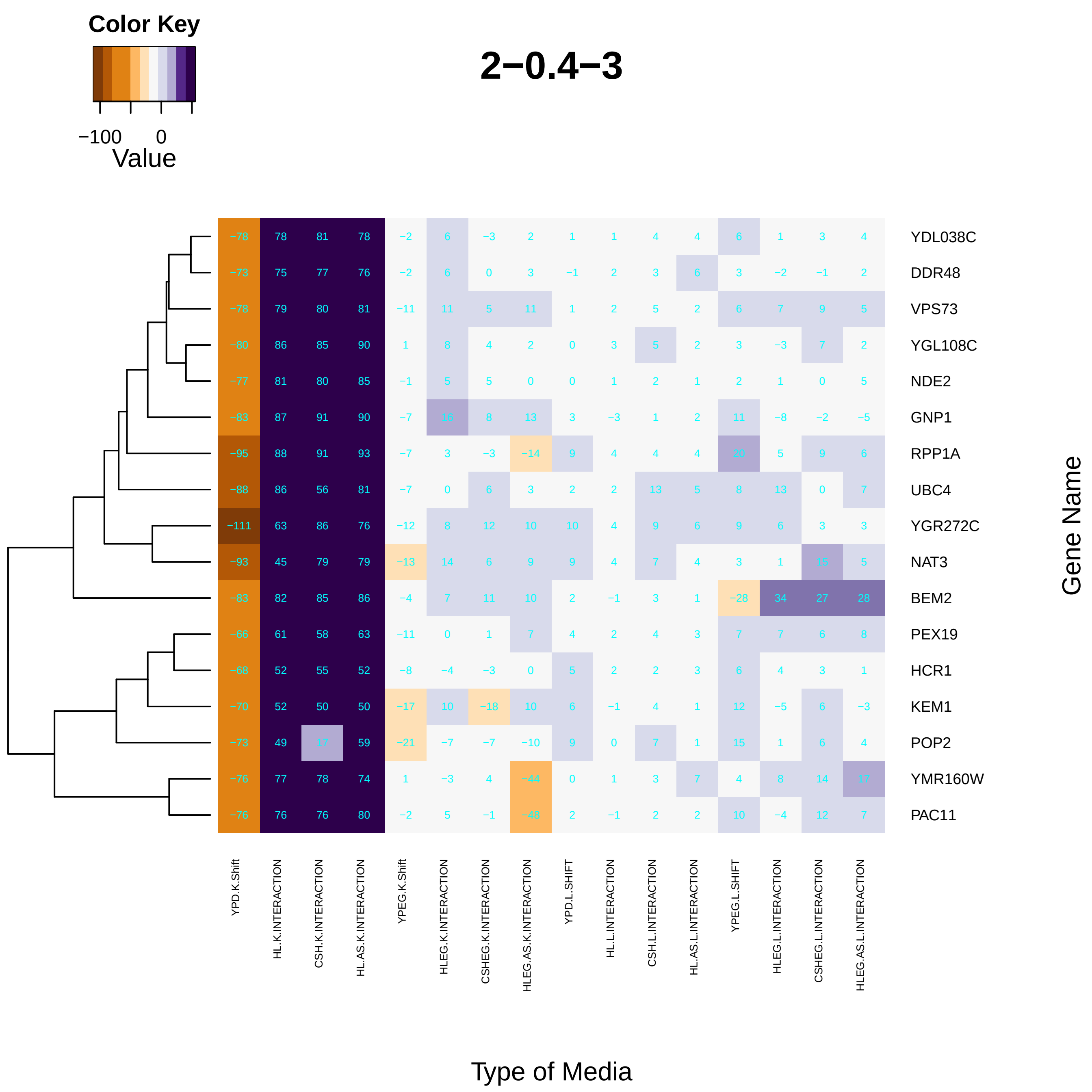
Color Key
−100
0
Value
2−0.4−3
YPD.K.Shift
HL.K.INTERACTION
CSH.K.INTERACTION
HL.AS.K.INTERACTION
YPEG.K.Shift
HLEG.K.INTERACTION
CSHEG.K.INTERACTION
HLEG.AS.K.INTERACTION
YPD.L.SHIFT
HL.L.INTERACTION
CSH.L.INTERACTION
HL.AS.L.INTERACTION
YPEG.L.SHIFT
HLEG.L.INTERACTION
CSHEG.L.INTERACTION
HLEG.AS.L.INTERACTION
YDL038C −78 78 81 78 −2 6 −3 2 1 1 4 4 6 1 3 4
DDR48 −73 75 77 76 −2 6 0 3 −1 2 3 6 3 −2 −1 2
VPS73 −78 79 80 81 −11 11 5 11 1 2 5 2 6 7 9 5
YGL108C −80 86 85 90 1 8 4 2 0 3 5 2 3 −3 7 2
NDE2 −77 81 80 85 −1 5 5 0 0 1 2 1 2 1 0 5
GNP1 −83 87 91 90 −7 16 8 13 3 −3 1 2 11 −8 −2 −5
RPP1A −95 88 91 93 −7 3 −3 −14 9 4 4 4 20 5 9 6
UBC4 −88 86 56 81 −7 0 6 3 2 2 13 5 8 13 0 7
YGR272C −111 63 86 76 −12 8 12 10 10 4 9 6 9 6 3 3
NAT3 −93 45 79 79 −13 14 6 9 9 4 7 4 3 1 15 5
BEM2 −83 82 85 86 −4 7 11 10 2 −1 3 1 −28 34 27 28
PEX19 −66 61 58 63 −11 0 1 7 4 2 4 3 7 7 6 8
HCR1 −68 52 55 52 −8 −4 −3 0 5 2 2 3 6 4 3 1
KEM1 −70 52 50 50 −17 10 −18 10 6 −1 4 1 12 −5 6 −3
POP2 −73 49 17 59 −21 −7 −7 −10 9 0 7 1 15 1 6 4
YMR160W −76 77 78 74 1 −3 4 −44 0 1 3 7 4 8 14 17
PAC11 −76 76 76 80 −2 5 −1 −48 2 −1 2 2 10 −4 12 7
Gene Name
Type of Media

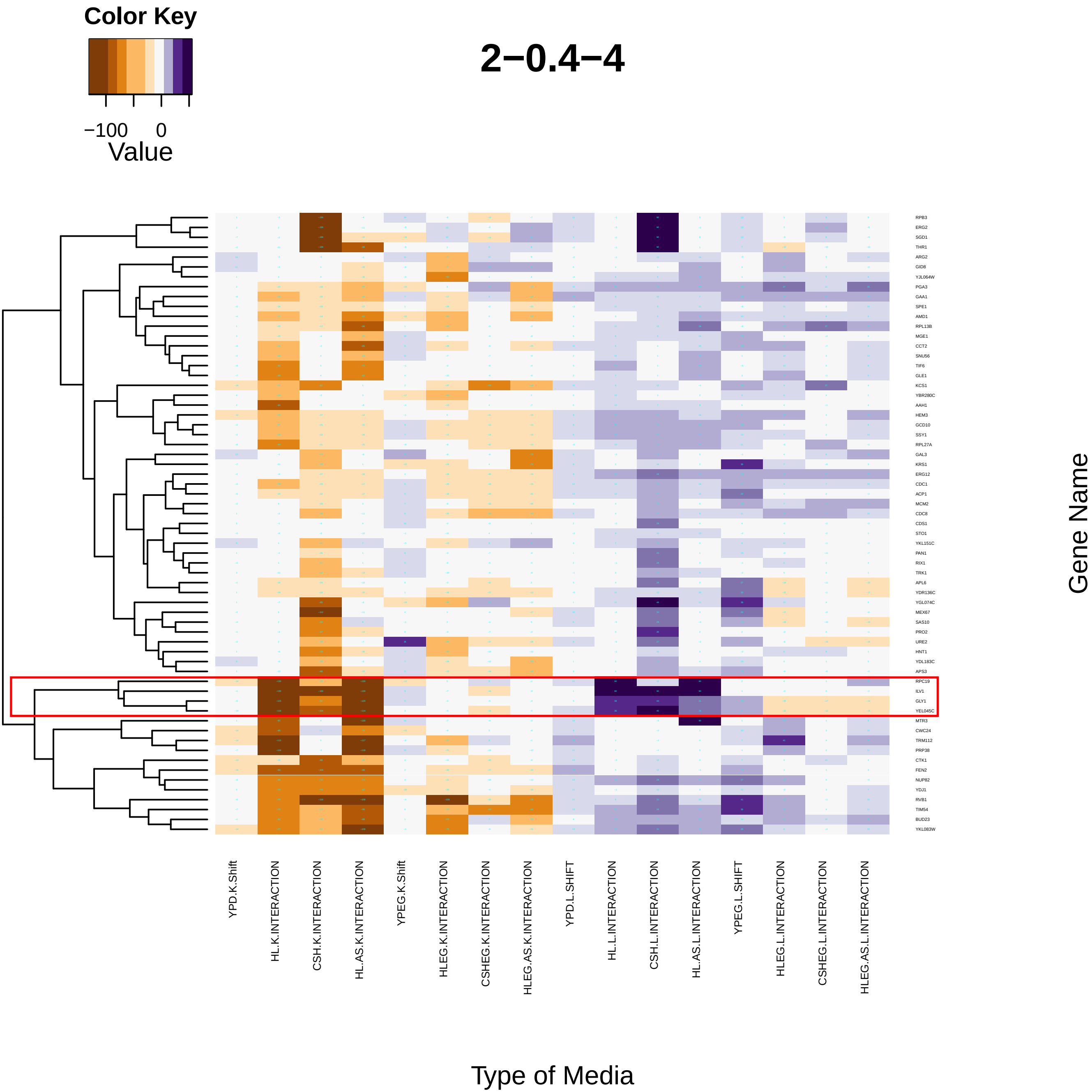

Color Key
−100
0
Value
2−0.4−4
Gene Name
Type of Media
YPD.K.Shift
HL.K.INTERACTION
CSH.K.INTERACTION
HL.AS.K.INTERACTION
YPEG.K.Shift
HLEG.K.INTERACTION
CSHEG.K.INTERACTION
HLEG.AS.K.INTERACTION
YPD.L.SHIFT
HL.L.INTERACTION
CSH.L.INTERACTION
HL.AS.L.INTERACTION
YPEG.L.SHIFT
HLEG.L.INTERACTION
CSHEG.L.INTERACTION
HLEG.AS.L.INTERACTION
RPB3
ERG2
SGD1
THR1
ARG2
GID8
YJL064W
PGA3
GAA1
SPE1
AMD1
RPL13B
MGE1
CCT2
SNU56
TIF6
GLE1
KCS1
YBR280C
AAH1
HEM3
GCD10
SSY1
RPL27A
GAL3
KRS1
ERG12
CDC1
ACP1
MCM2
CDC8
CDS1
STO1
YKL151C
PAN1
RIX1
TRK1
APL6
YDR136C
YGL074C
MEX67
SAS10
PRO2
URE2
HNT1
YDL183C
APS3
RPC19
ILV1
GLY1
YEL045C
MTR3
CWC24
TRM112
PRP38
CTK1
FEN2
NUP82
YDJ1
RVB1
TIM54
BUD23
YKL083W

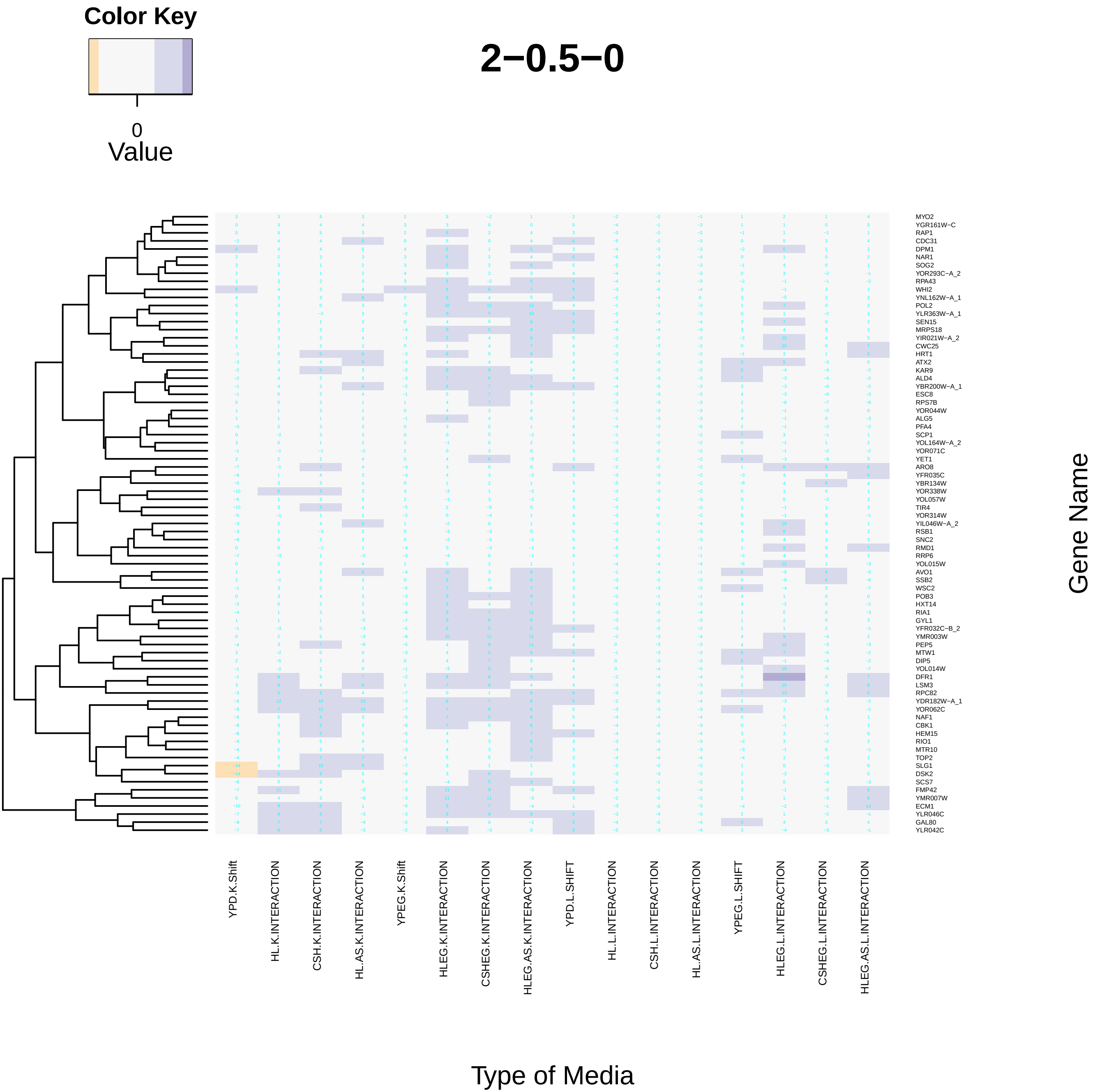
Color Key
0
Value
2−0.5−0
MYO2
YGR161W−C
RAP1
CDC31
DPM1
NAR1
SOG2
YOR293C−A_2
RPA43
WHI2
YNL162W−A_1
POL2
YLR363W−A_1
SEN15
MRPS18
YIR021W−A_2
CWC25
HRT1
ATX2
KAR9
ALD4
YBR200W−A_1
ESC8
RPS7B
YOR044W
ALG5
PFA4
SCP1
YOL164W−A_2
YOR071C
YET1
ARO8
YFR035C
YBR134W
YOR338W
YOL057W
TIR4
YOR314W
YIL046W−A_2
RSB1
SNC2
RMD1
RRP6
YOL015W
AVO1
SSB2
WSC2
POB3
HXT14
RIA1
GYL1
YFR032C−B_2
YMR003W
PEP5
MTW1
DIP5
YOL014W
DFR1
LSM3
RPC82
YDR182W−A_1
YOR062C
NAF1
CBK1
HEM15
RIO1
MTR10
TOP2
SLG1
DSK2
SCS7
FMP42
YMR007W
ECM1
YLR046C
GAL80
YLR042C
Gene Name
YPD.K.Shift
HL.K.INTERACTION
CSH.K.INTERACTION
HL.AS.K.INTERACTION
YPEG.K.Shift
HLEG.K.INTERACTION
CSHEG.K.INTERACTION
HLEG.AS.K.INTERACTION
YPD.L.SHIFT
HL.L.INTERACTION
CSH.L.INTERACTION
HL.AS.L.INTERACTION
YPEG.L.SHIFT
HLEG.L.INTERACTION
CSHEG.L.INTERACTION
HLEG.AS.L.INTERACTION
Type of Media

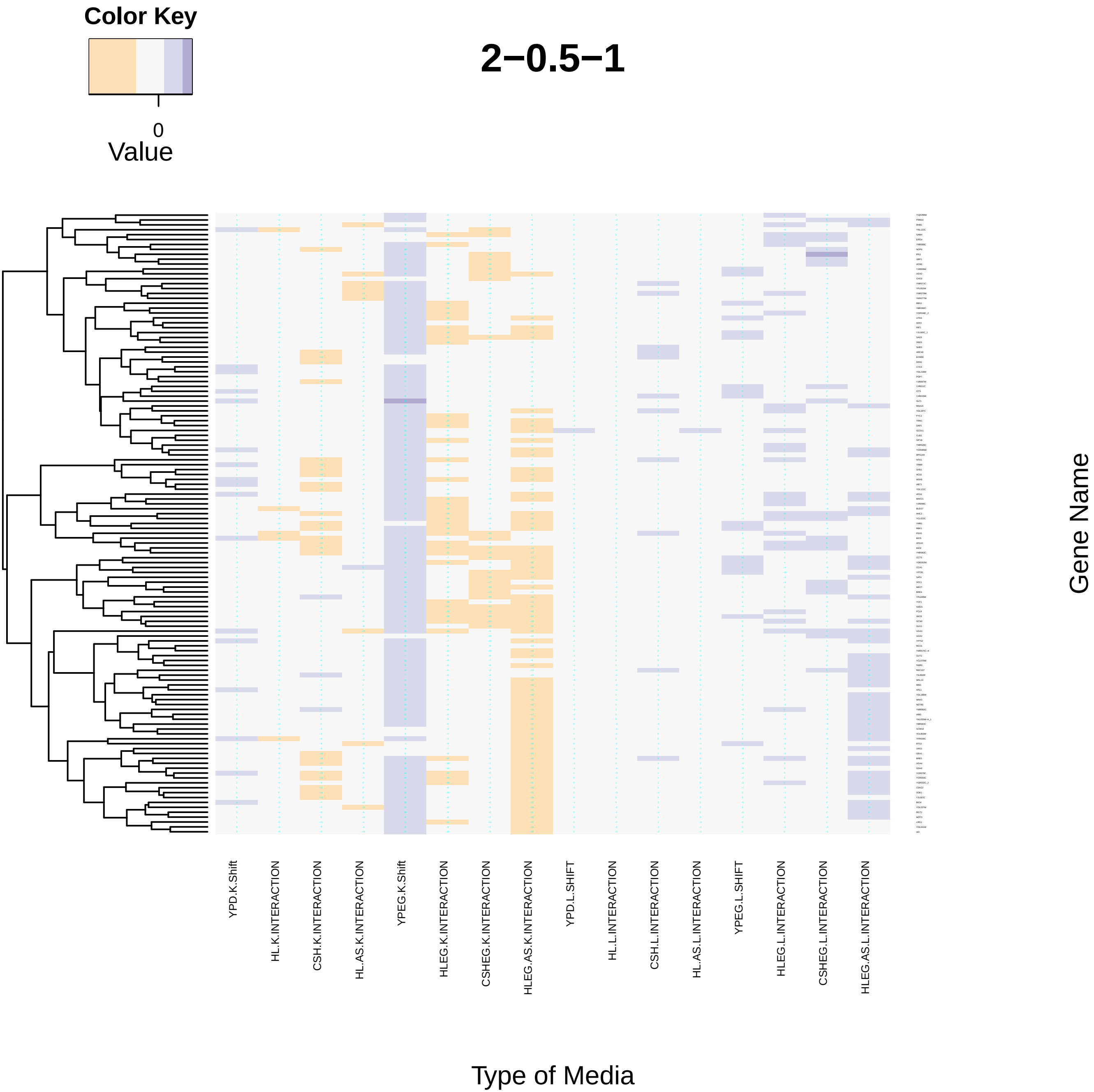
Color Key
0
Value
2−0.5−1
YPD.K.Shift
HL.K.INTERACTION
CSH.K.INTERACTION
HL.AS.K.INTERACTION
YPEG.K.Shift
HLEG.K.INTERACTION
CSHEG.K.INTERACTION
HLEG.AS.K.INTERACTION
YPD.L.SHIFT
HL.L.INTERACTION
CSH.L.INTERACTION
HL.AS.L.INTERACTION
YPEG.L.SHIFT
HLEG.L.INTERACTION
CSHEG.L.INTERACTION
HLEG.AS.L.INTERACTION
Gene Name
Type of Media

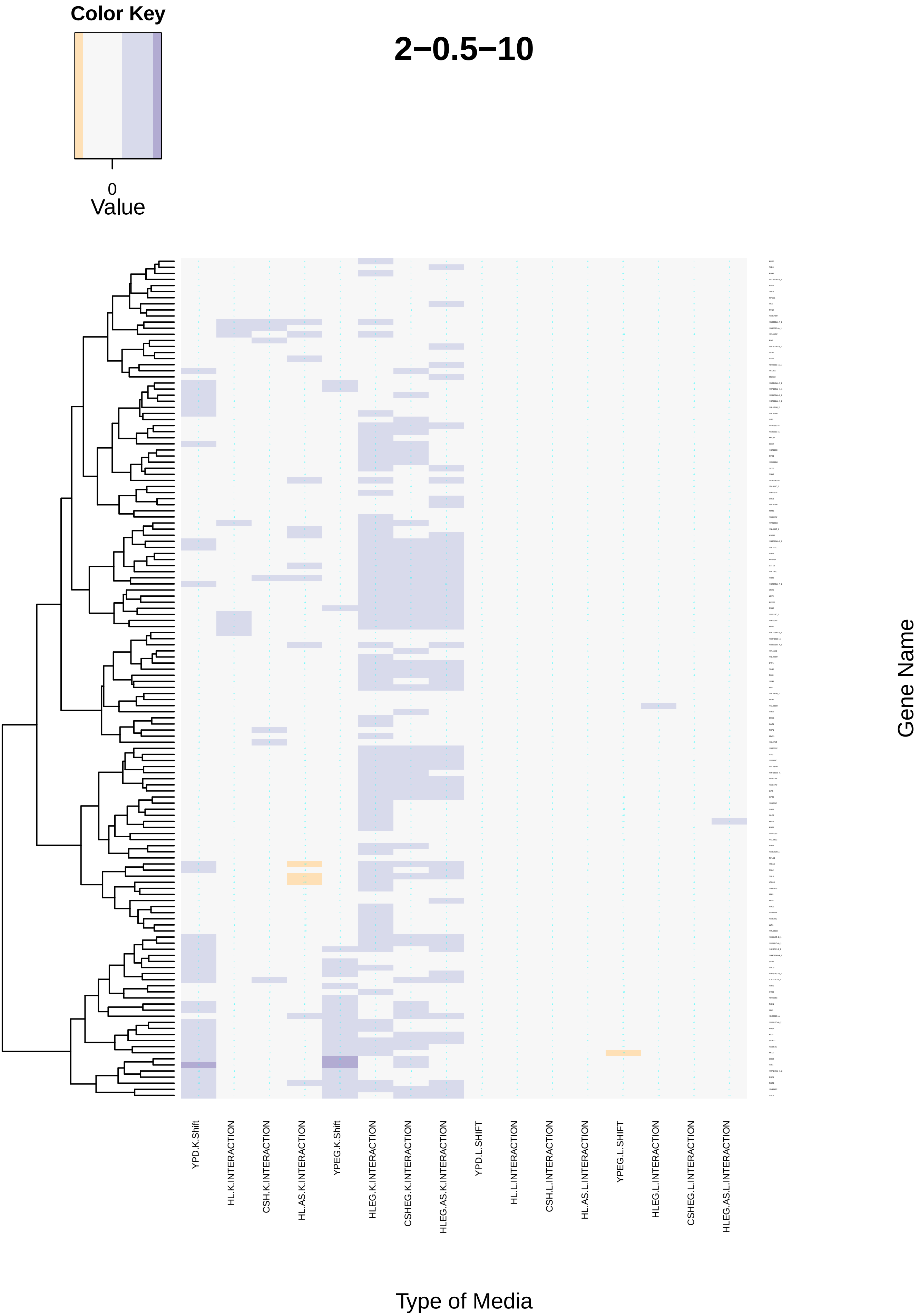

Color Key
0
Value
2−0.5−10
YPD.K.Shift
HL.K.INTERACTION
CSH.K.INTERACTION
HL.AS.K.INTERACTION
YPEG.K.Shift
HLEG.K.INTERACTION
CSHEG.K.INTERACTION
HLEG.AS.K.INTERACTION
YPD.L.SHIFT
HL.L.INTERACTION
CSH.L.INTERACTION
HL.AS.L.INTERACTION
YPEG.L.SHIFT
HLEG.L.INTERACTION
CSHEG.L.INTERACTION
HLEG.AS.L.INTERACTION
Type of Media
Gene Name

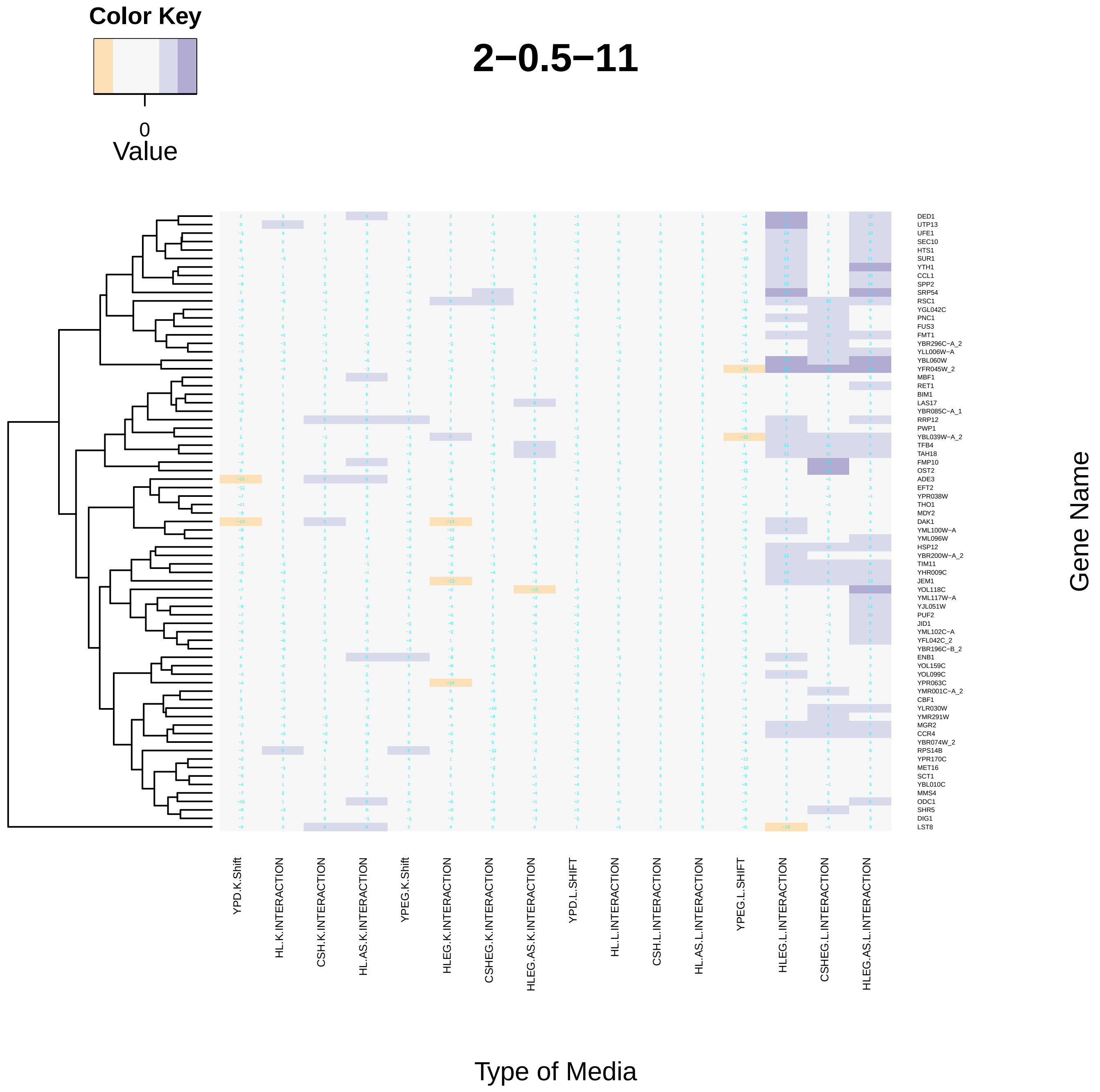

Color Key
0
Value
2−0.5−11
DED1
UTP13
UFE1
SEC10
HTS1
SUR1
YTH1
CCL1
SPP2
SRP54
RSC1
YGL042C
PNC1
FUS3
FMT1
YBR296C−A_2
YLL006W−A
YBL060W
YFR045W_2
MBF1
RET1
BIM1
LAS17
YBR085C−A_1
RRP12
PWP1
YBL039W−A_2
TFB4
TAH18
FMP10
OST2
ADE3
EFT2
YPR038W
THO1
MDY2
DAK1
YML100W−A
YML096W
HSP12
YBR200W−A_2
TIM11
YHR009C
JEM1
YOL118C
YML117W−A
YJL051W
PUF2
JID1
YML102C−A
YFL042C_2
YBR196C−B_2
ENB1
YOL159C
YOL099C
YPR063C
YMR001C−A_2
CBF1
YLR030W
YMR291W
MGR2
CCR4
YBR074W_2
RPS14B
YPR170C
MET16
SCT1
YBL010C
MMS4
ODC1
SHR5
DIG1
LST8
Gene Name
YPD.K.Shift
HL.K.INTERACTION
CSH.K.INTERACTION
HL.AS.K.INTERACTION
YPEG.K.Shift
HLEG.K.INTERACTION
CSHEG.K.INTERACTION
HLEG.AS.K.INTERACTION
YPD.L.SHIFT
HL.L.INTERACTION
CSH.L.INTERACTION
HL.AS.L.INTERACTION
YPEG.L.SHIFT
HLEG.L.INTERACTION
CSHEG.L.INTERACTION
HLEG.AS.L.INTERACTION
Type of Media

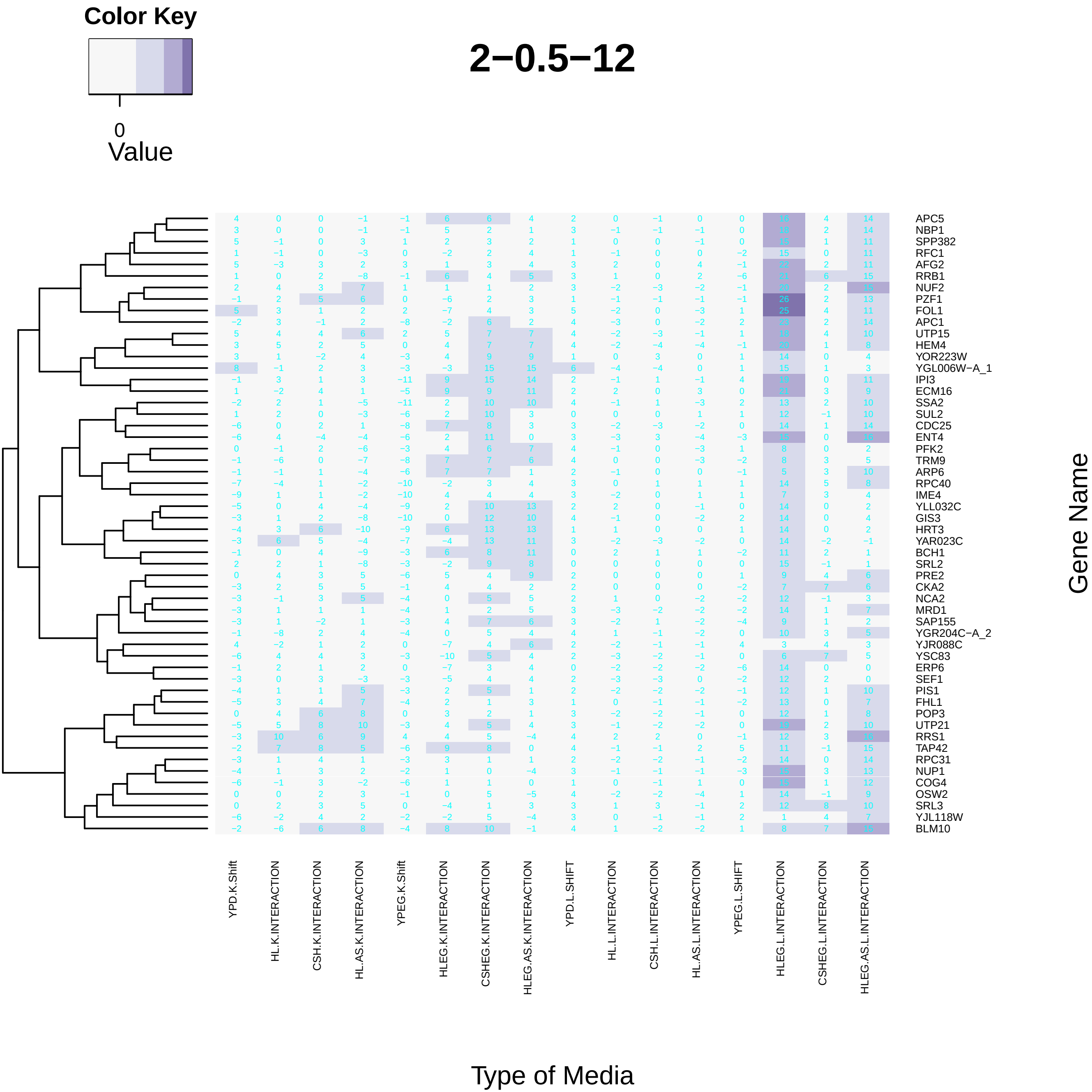

Color Key
0
Value
2−0.5−12
APC5
NBP1
SPP382
RFC1
AFG2
RRB1
NUF2
PZF1
FOL1
APC1
UTP15
HEM4
YOR223W
YGL006W−A_1
IPI3
ECM16
SSA2
SUL2
CDC25
ENT4
PFK2
TRM9
ARP6
RPC40
IME4
YLL032C
GIS3
HRT3
YAR023C
BCH1
SRL2
PRE2
CKA2
NCA2
MRD1
SAP155
YGR204C−A_2
YJR088C
YSC83
ERP6
SEF1
PIS1
FHL1
POP3
UTP21
RRS1
TAP42
RPC31
NUP1
COG4
OSW2
SRL3
YJL118W
BLM10
Gene Name
YPD.K.Shift
HL.K.INTERACTION
CSH.K.INTERACTION
HL.AS.K.INTERACTION
YPEG.K.Shift
HLEG.K.INTERACTION
CSHEG.K.INTERACTION
HLEG.AS.K.INTERACTION
YPD.L.SHIFT
HL.L.INTERACTION
CSH.L.INTERACTION
HL.AS.L.INTERACTION
YPEG.L.SHIFT
HLEG.L.INTERACTION
CSHEG.L.INTERACTION
HLEG.AS.L.INTERACTION
Type of Media

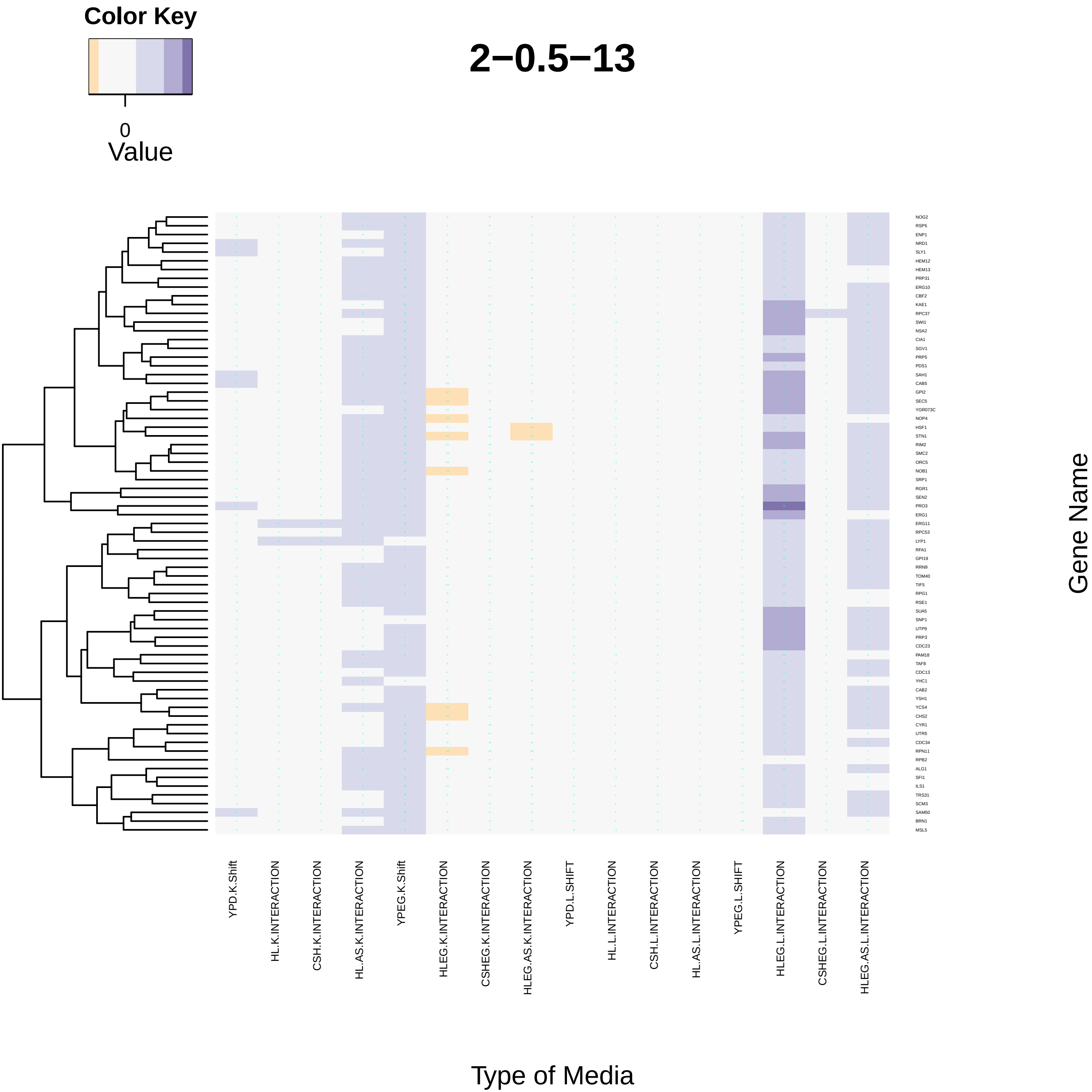
Color Key
0
Value
2−0.5−13
Gene Name
Type of Media
YPD.K.Shift
HL.K.INTERACTION
CSH.K.INTERACTION
HL.AS.K.INTERACTION
YPEG.K.Shift
HLEG.K.INTERACTION
CSHEG.K.INTERACTION
HLEG.AS.K.INTERACTION
YPD.L.SHIFT
HL.L.INTERACTION
CSH.L.INTERACTION
HL.AS.L.INTERACTION
YPEG.L.SHIFT
HLEG.L.INTERACTION
CSHEG.L.INTERACTION
HLEG.AS.L.INTERACTION
NOG2
RSP5
ENP1
NRD1
SLY1
HEM12
HEM13
PRP31
ERG10
CBF2
KAE1
RPC37
SWI1
NSA2
CIA1
SGV1
PRP5
PDS1
SAH1
CAB5
GPI2
SEC5
YGR073C
NOP4
HSF1
STN1
RIM2
SMC2
ORC5
NOB1
SRP1
RGR1
SEN2
PRO3
ERG1
ERG11
RPC53
LYP1
RFA1
GPI19
RRN9
TOM40
TIF5
RPG1
RSE1
SUA5
SNP1
UTP9
PRP3
CDC23
PAM18
TAF8
CDC13
YHC1
CAB2
YSH1
YCS4
CHS2
CYR1
UTR5
CDC34
RPN11
RPB2
ALG1
SFI1
ILS1
TRS31
SCM3
SAM50
BRN1
MSL5

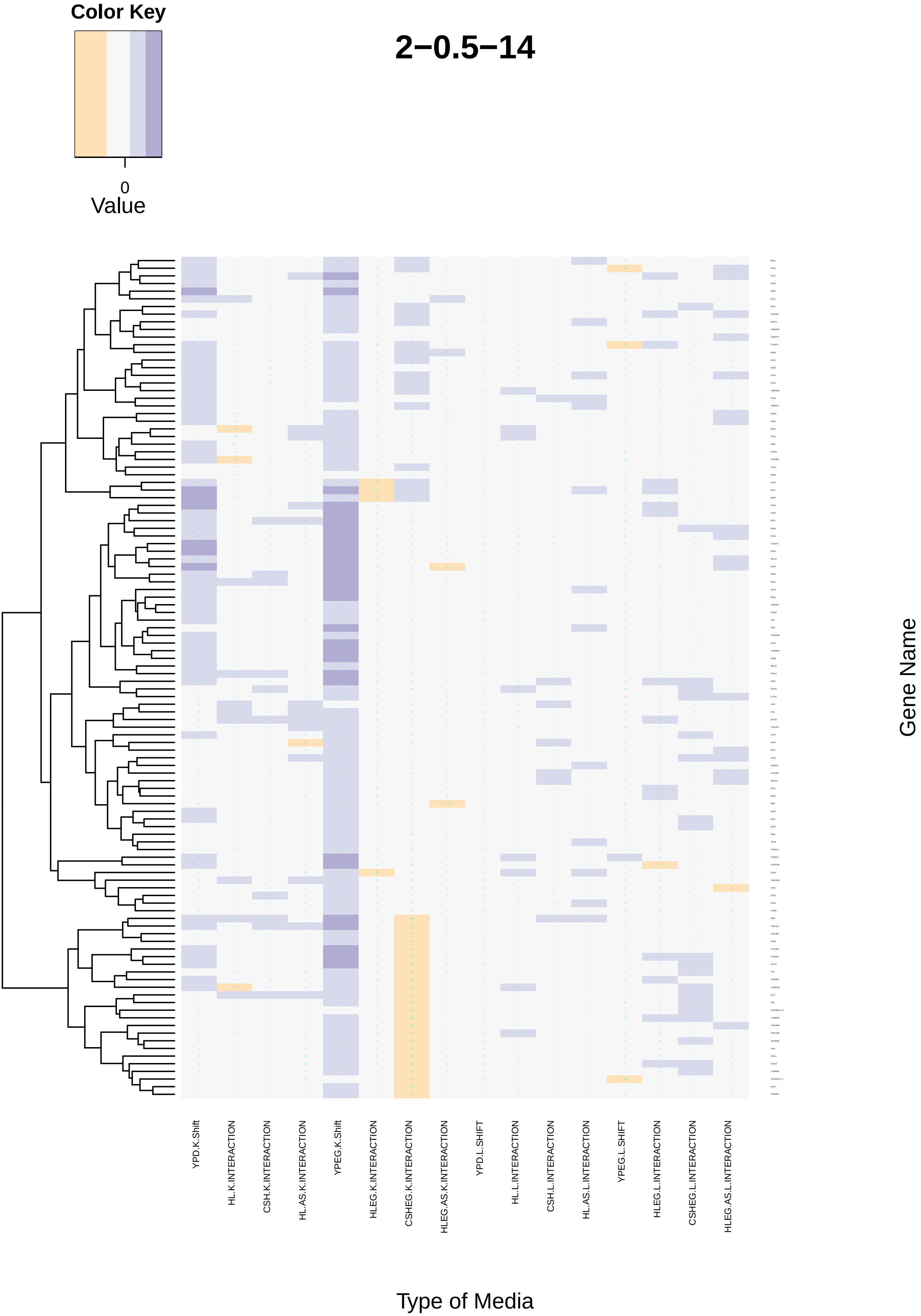
Color Key
0
Value
2−0.5−14
YPD.K.Shift
HL.K.INTERACTION
CSH.K.INTERACTION
HL.AS.K.INTERACTION
YPEG.K.Shift
HLEG.K.INTERACTION
CSHEG.K.INTERACTION
HLEG.AS.K.INTERACTION
YPD.L.SHIFT
HL.L.INTERACTION
CSH.L.INTERACTION
HL.AS.L.INTERACTION
YPEG.L.SHIFT
HLEG.L.INTERACTION
CSHEG.L.INTERACTION
HLEG.AS.L.INTERACTION
Gene Name
Type of Media

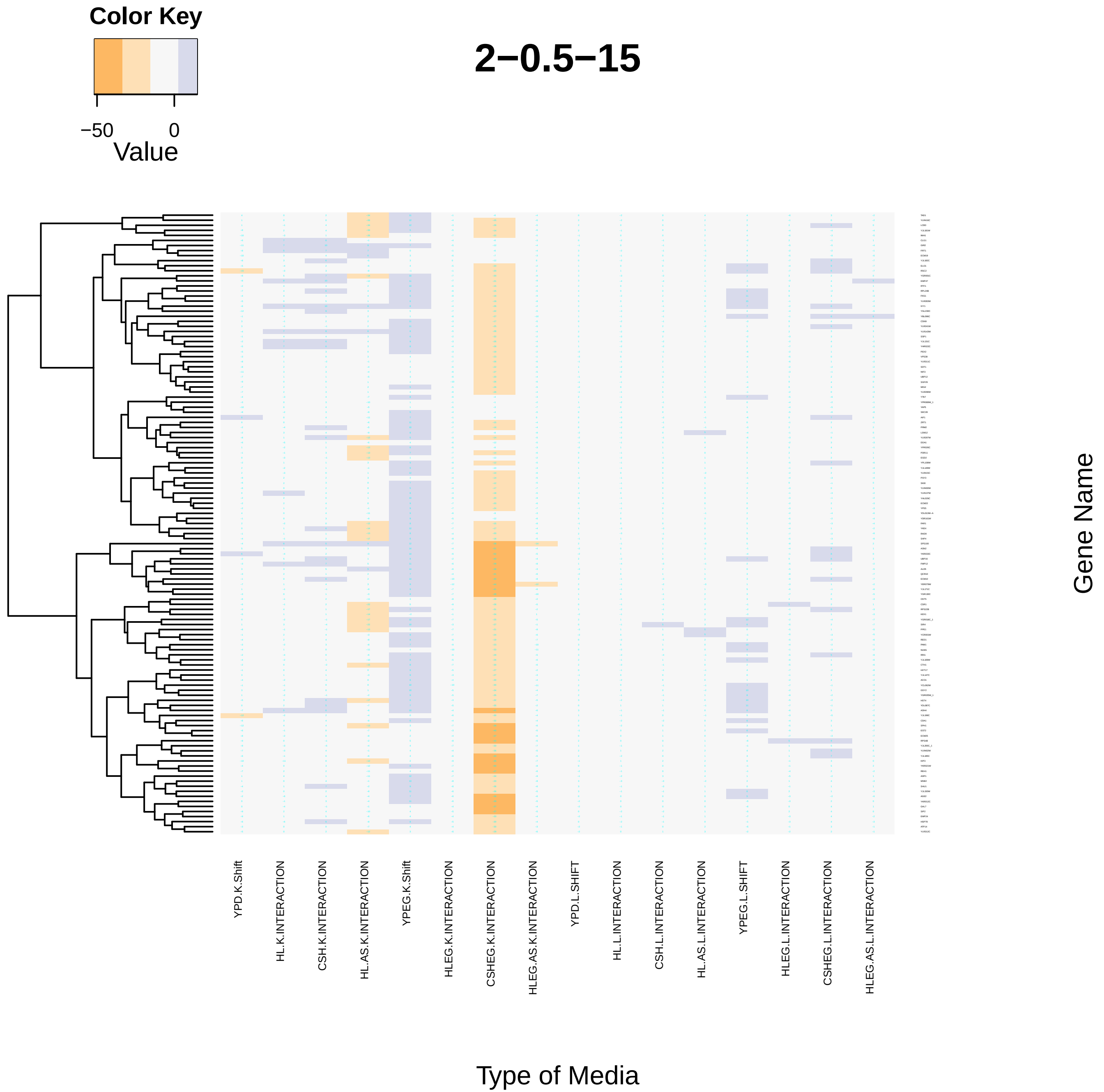
Color Key
−50
0
Value
2−0.5−15
YPD.K.Shift
HL.K.INTERACTION
CSH.K.INTERACTION
HL.AS.K.INTERACTION
YPEG.K.Shift
HLEG.K.INTERACTION
CSHEG.K.INTERACTION
HLEG.AS.K.INTERACTION
YPD.L.SHIFT
HL.L.INTERACTION
CSH.L.INTERACTION
HL.AS.L.INTERACTION
YPEG.L.SHIFT
HLEG.L.INTERACTION
CSHEG.L.INTERACTION
HLEG.AS.L.INTERACTION
Gene Name
Type of Media

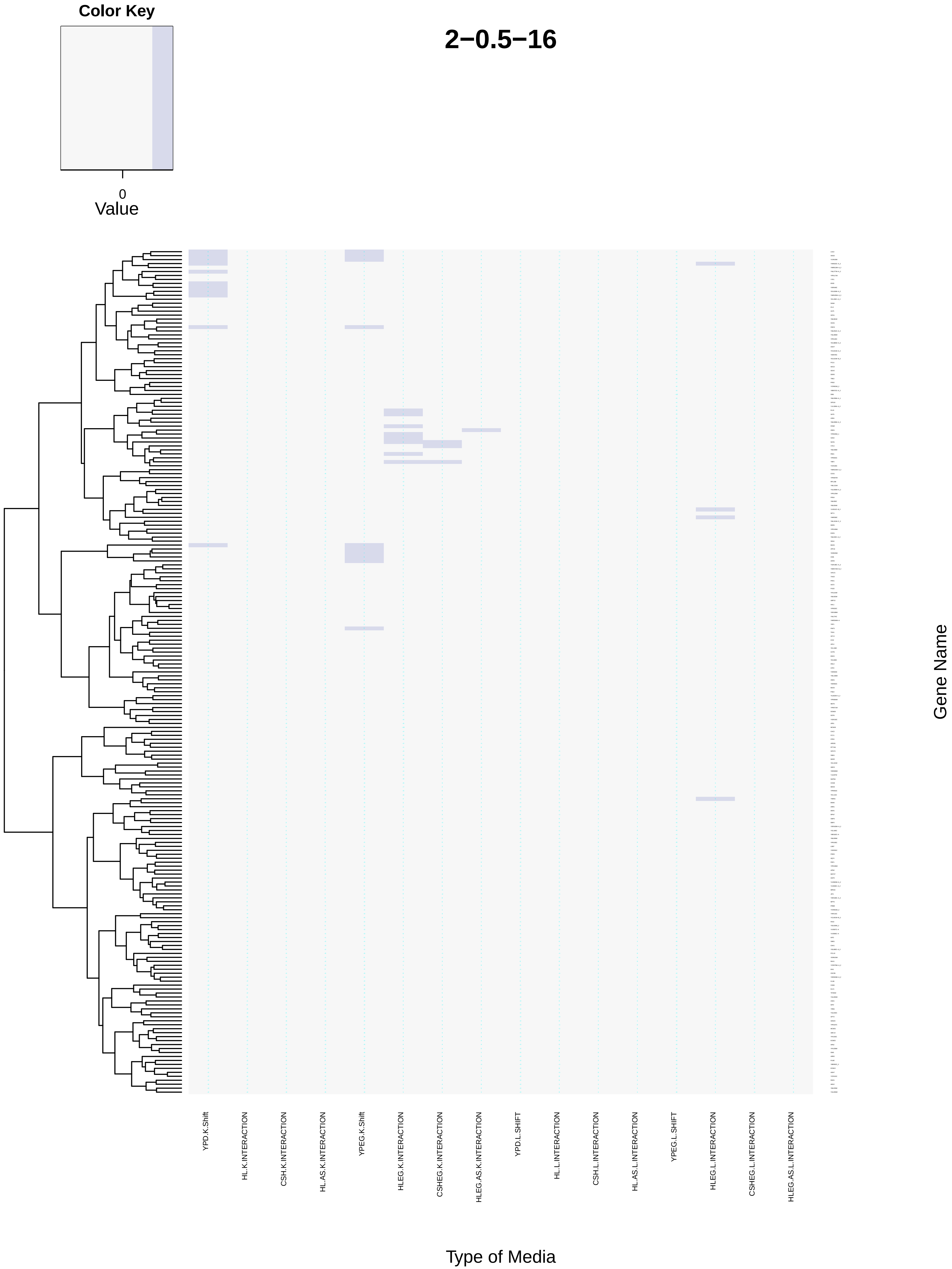
Color Key
0
Value
2−0.5−16
YPD.K.Shift
HL.K.INTERACTION
CSH.K.INTERACTION
HL.AS.K.INTERACTION
YPEG.K.Shift
HLEG.K.INTERACTION
CSHEG.K.INTERACTION
HLEG.AS.K.INTERACTION
YPD.L.SHIFT
HL.L.INTERACTION
CSH.L.INTERACTION
HL.AS.L.INTERACTION
YPEG.L.SHIFT
HLEG.L.INTERACTION
CSHEG.L.INTERACTION
HLEG.AS.L.INTERACTION
Type of Media
Gene Name

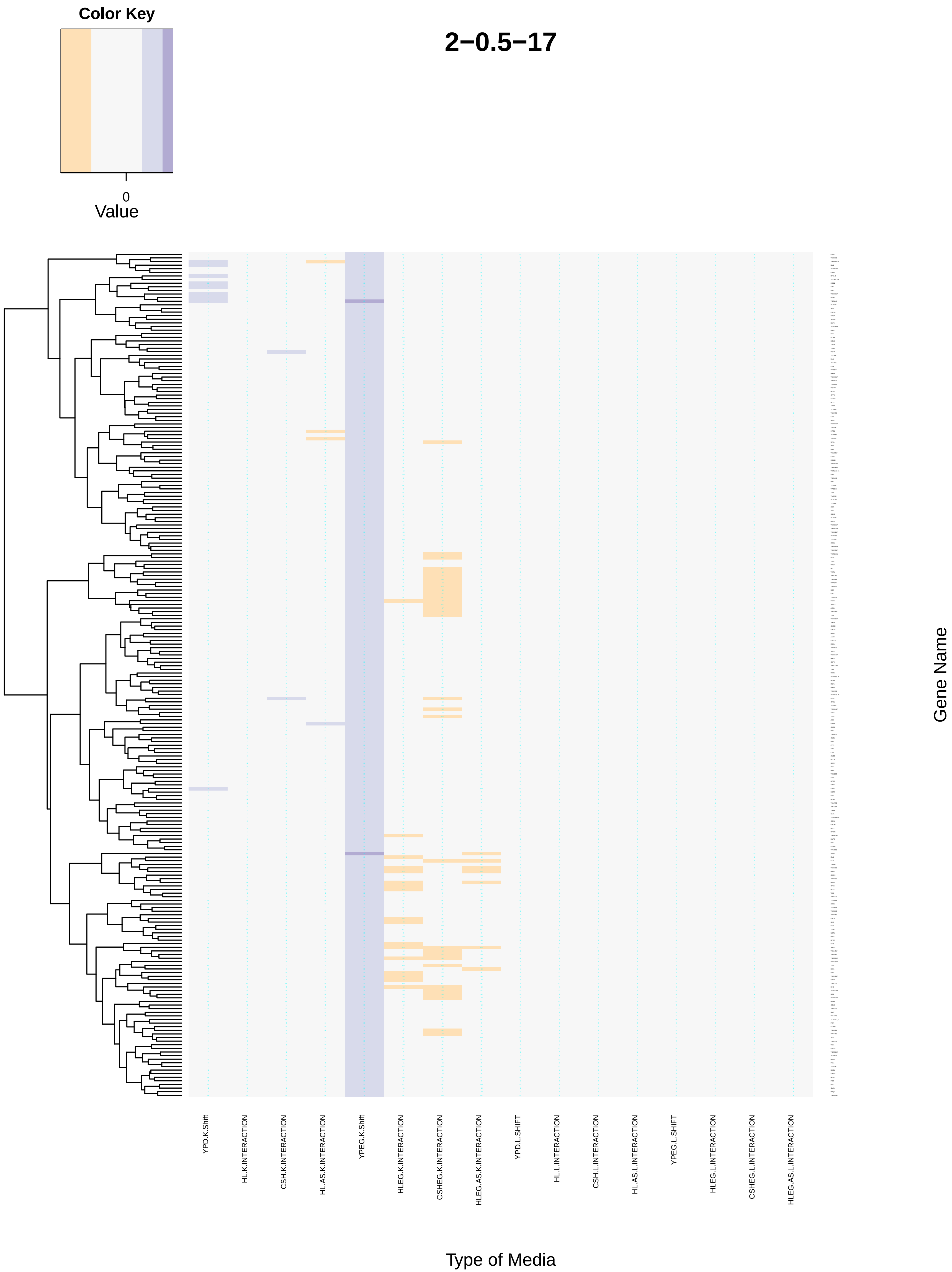
Color Key
0
Value
2−0.5−17
Gene Name
YPD.K.Shift
HL.K.INTERACTION
CSH.K.INTERACTION
HL.AS.K.INTERACTION
YPEG.K.Shift
HLEG.K.INTERACTION
CSHEG.K.INTERACTION
HLEG.AS.K.INTERACTION
YPD.L.SHIFT
HL.L.INTERACTION
CSH.L.INTERACTION
HL.AS.L.INTERACTION
YPEG.L.SHIFT
HLEG.L.INTERACTION
CSHEG.L.INTERACTION
HLEG.AS.L.INTERACTION
Type of Media

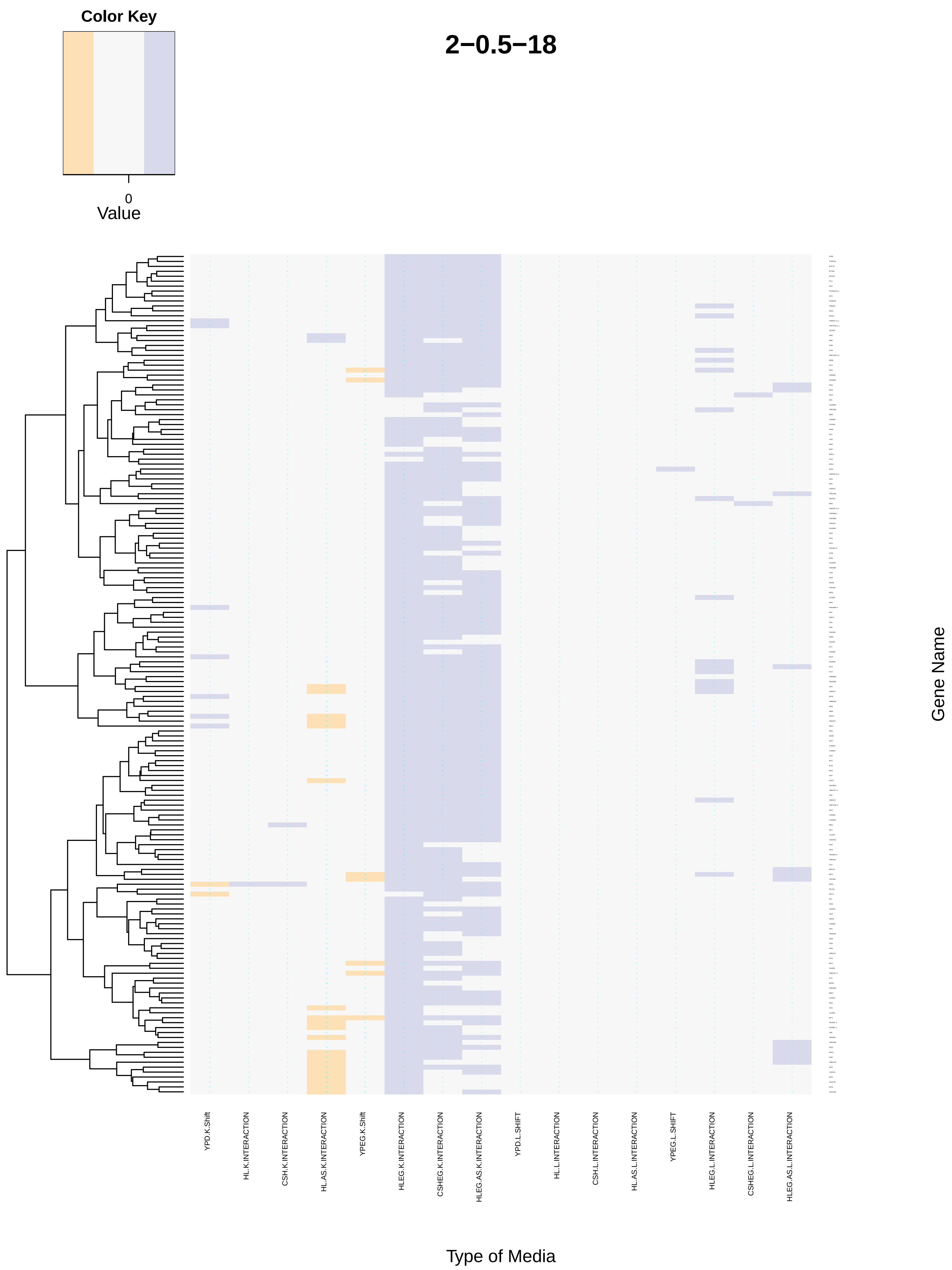
Color Key
0
Value
2−0.5−18
YPD.K.Shift
HL.K.INTERACTION
CSH.K.INTERACTION
HL.AS.K.INTERACTION
YPEG.K.Shift
HLEG.K.INTERACTION
CSHEG.K.INTERACTION
HLEG.AS.K.INTERACTION
YPD.L.SHIFT
HL.L.INTERACTION
CSH.L.INTERACTION
HL.AS.L.INTERACTION
YPEG.L.SHIFT
HLEG.L.INTERACTION
CSHEG.L.INTERACTION
HLEG.AS.L.INTERACTION
Type of Media
Gene Name

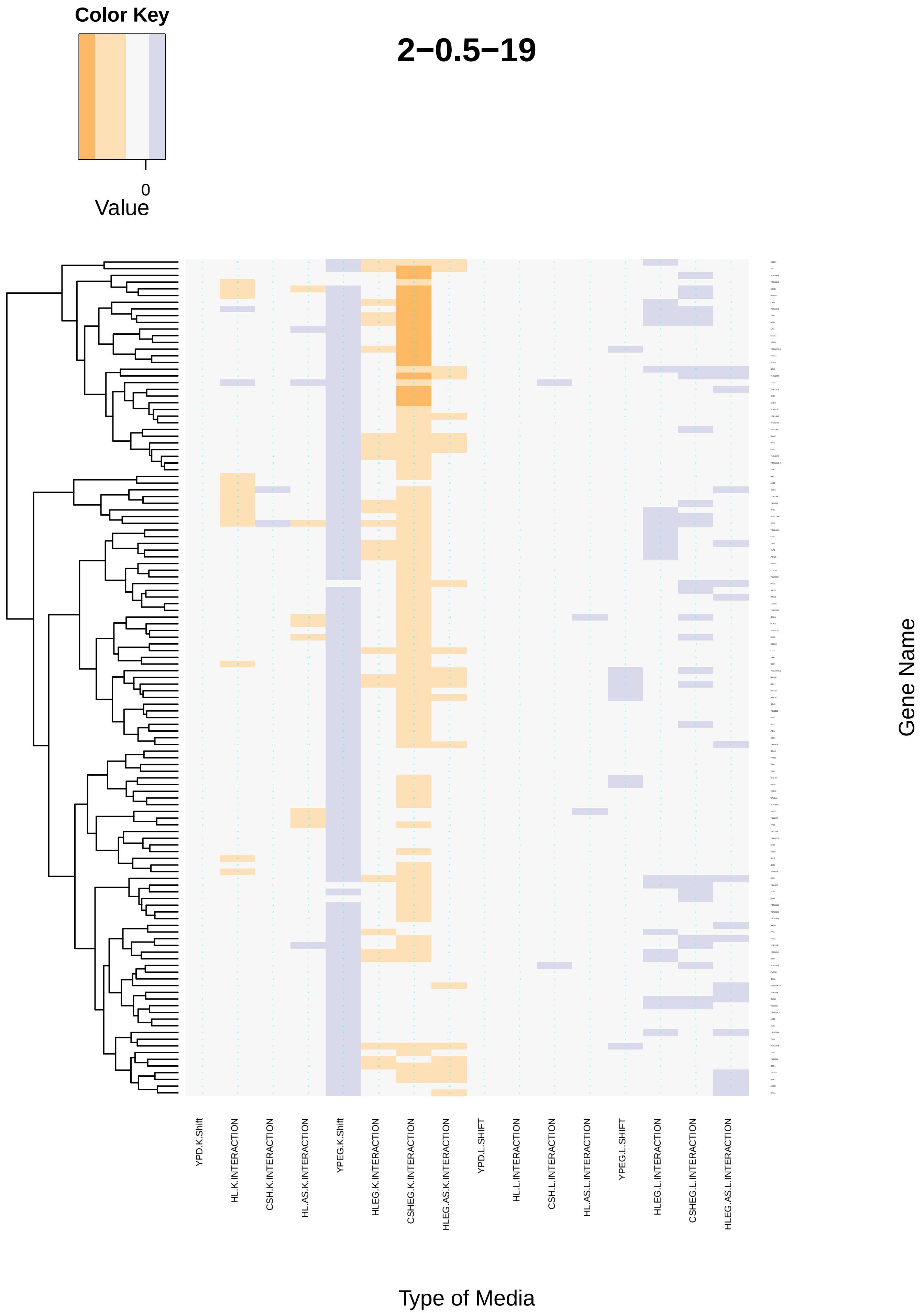

Color Key
0
Value
2−0.5−19
YPD.K.Shift
HL.K.INTERACTION
CSH.K.INTERACTION
HL.AS.K.INTERACTION
YPEG.K.Shift
HLEG.K.INTERACTION
CSHEG.K.INTERACTION
HLEG.AS.K.INTERACTION
YPD.L.SHIFT
HL.L.INTERACTION
CSH.L.INTERACTION
HL.AS.L.INTERACTION
YPEG.L.SHIFT
HLEG.L.INTERACTION
CSHEG.L.INTERACTION
HLEG.AS.L.INTERACTION
Gene Name
Type of Media

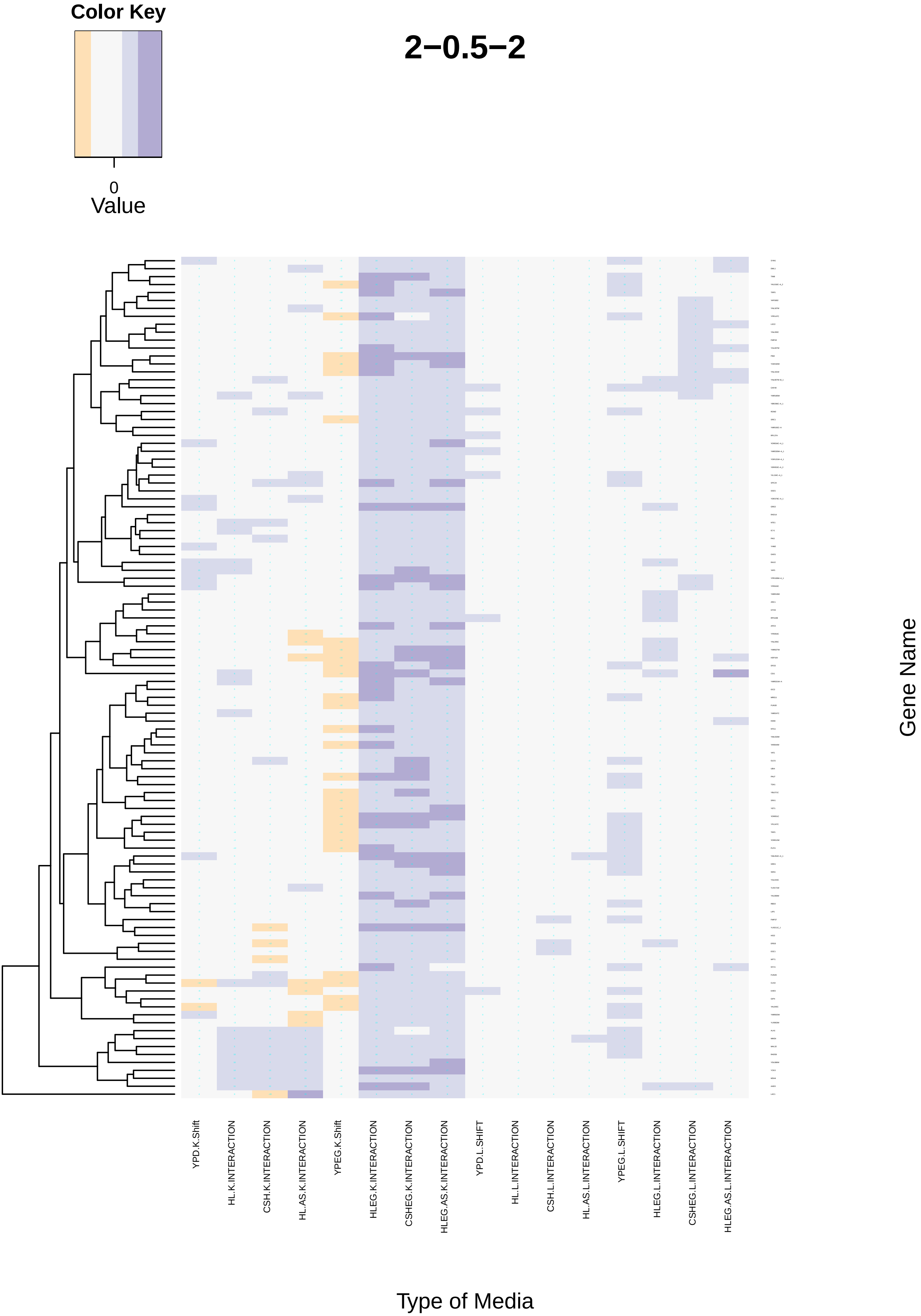
Color Key
0
Value
2−0.5−2
YPD.K.Shift
HL.K.INTERACTION
CSH.K.INTERACTION
HL.AS.K.INTERACTION
YPEG.K.Shift
HLEG.K.INTERACTION
CSHEG.K.INTERACTION
HLEG.AS.K.INTERACTION
YPD.L.SHIFT
HL.L.INTERACTION
CSH.L.INTERACTION
HL.AS.L.INTERACTION
YPEG.L.SHIFT
HLEG.L.INTERACTION
CSHEG.L.INTERACTION
HLEG.AS.L.INTERACTION
Gene Name
Type of Media

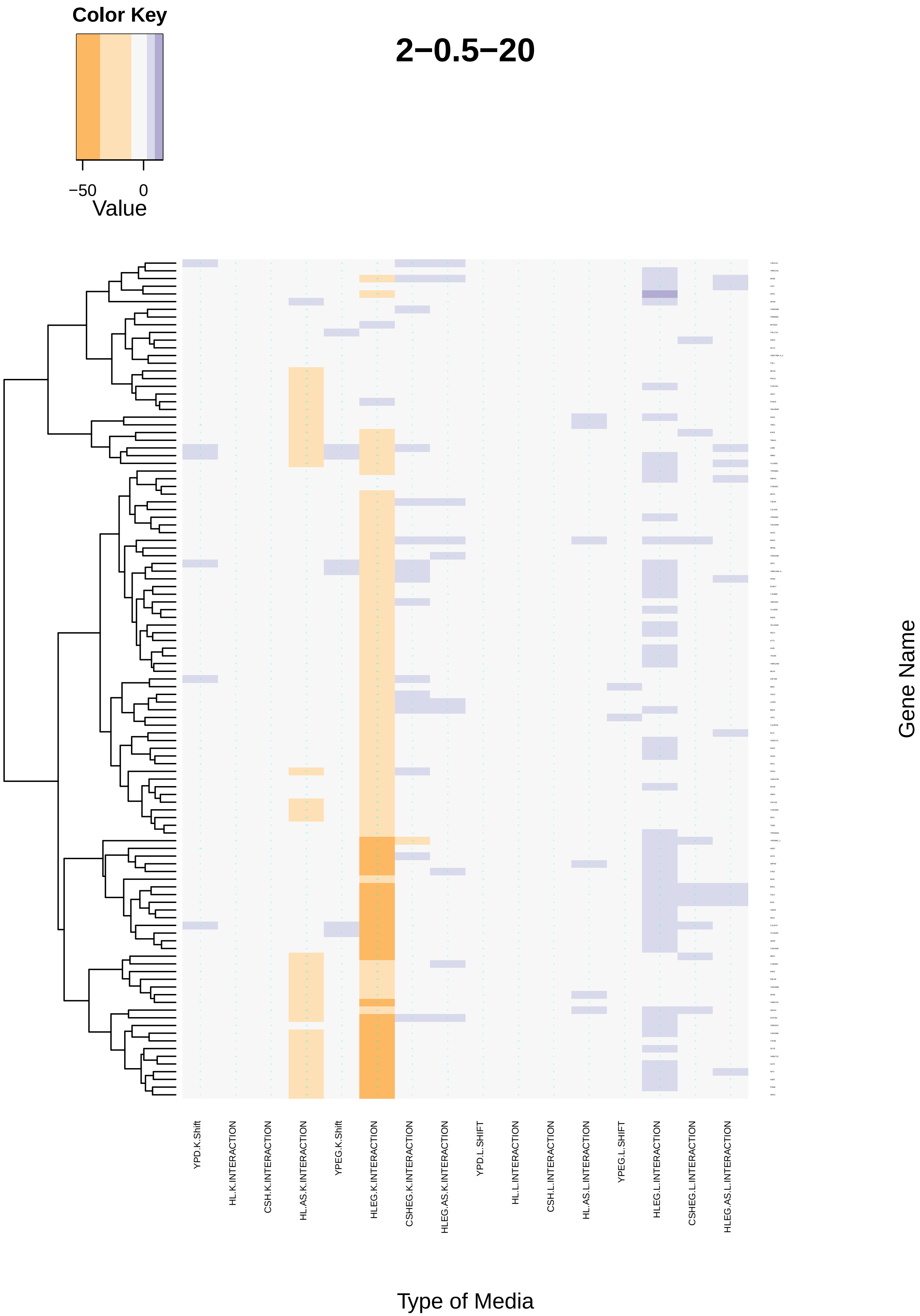

Color Key
−50
0
Value
2−0.5−20
YPD.K.Shift
HL.K.INTERACTION
CSH.K.INTERACTION
HL.AS.K.INTERACTION
YPEG.K.Shift
HLEG.K.INTERACTION
CSHEG.K.INTERACTION
HLEG.AS.K.INTERACTION
YPD.L.SHIFT
HL.L.INTERACTION
CSH.L.INTERACTION
HL.AS.L.INTERACTION
YPEG.L.SHIFT
HLEG.L.INTERACTION
CSHEG.L.INTERACTION
HLEG.AS.L.INTERACTION
Type of Media
Gene Name

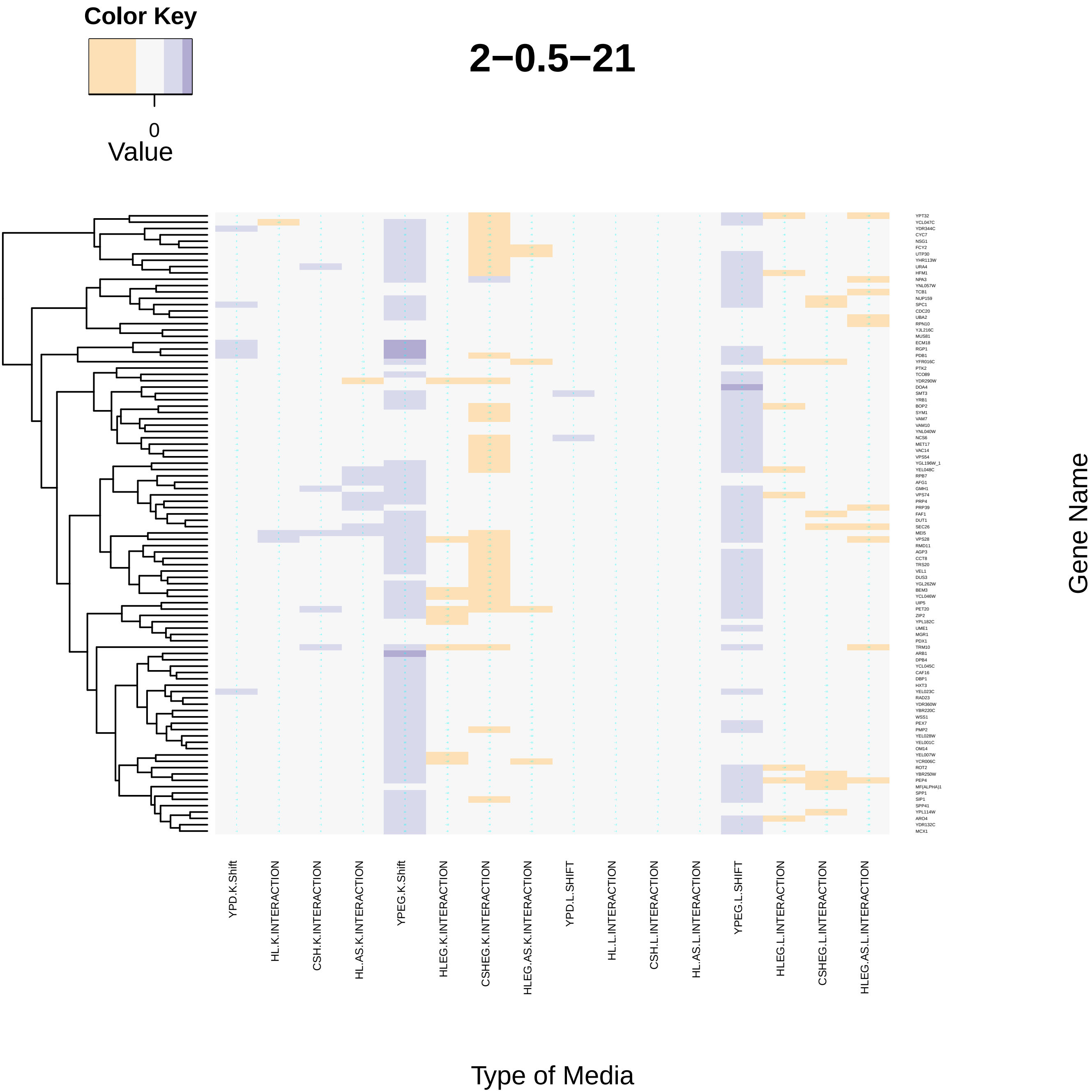
Color Key
0
Value
2−0.5−21
YPT32
YCL047C
YDR344C
CYC7
NSG1
FCY2
UTP30
YHR113W
URA4
HFM1
NPA3
YNL057W
TCB1
NUP159
SPC1
CDC20
UBA2
RPN10
YJL216C
MUS81
ECM18
RGP1
PDB1
YFR016C
PTK2
TCO89
YDR290W
DOA4
SMT3
YRB1
BOP2
SYM1
VAM7
VAM10
YNL040W
NCS6
MET17
VAC14
VPS54
YGL196W_1
YEL048C
RPB7
AFG1
GMH1
VPS74
PRP4
PRP39
FAF1
DUT1
SEC26
MEI5
VPS28
RMD11
AGP3
CCT8
TRS20
VEL1
DUS3
YGL262W
BEM3
YCL046W
UIP5
PET20
ZIP2
YPL182C
UME1
MGR1
PDX1
TRM10
ARB1
DPB4
YCL045C
CAF16
DBP1
HXT3
YEL023C
RAD23
YDR360W
YBR220C
WSS1
PEX7
PMP2
YEL028W
YEL001C
OM14
YEL007W
YCR006C
ROT2
YBR250W
PEP4
MF(ALPHA)1
SPP1
SIP1
SPP41
YPL114W
ARO4
YDR132C
MCX1
Gene Name
YPD.K.Shift
HL.K.INTERACTION
CSH.K.INTERACTION
HL.AS.K.INTERACTION
YPEG.K.Shift
HLEG.K.INTERACTION
CSHEG.K.INTERACTION
HLEG.AS.K.INTERACTION
YPD.L.SHIFT
HL.L.INTERACTION
CSH.L.INTERACTION
HL.AS.L.INTERACTION
YPEG.L.SHIFT
HLEG.L.INTERACTION
CSHEG.L.INTERACTION
HLEG.AS.L.INTERACTION
Type of Media

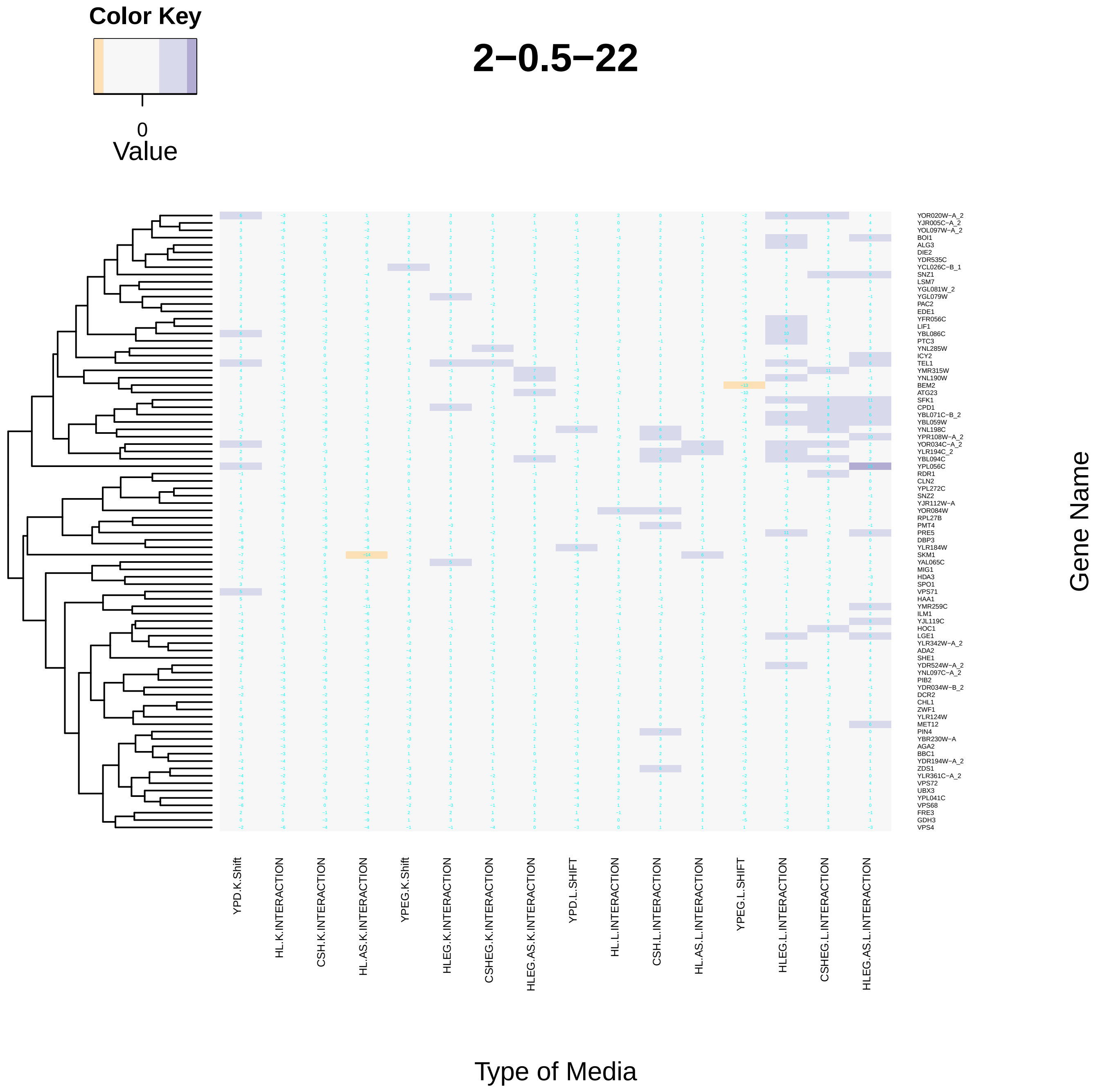

Color Key
0
Value
2−0.5−22
YOR020W−A_2
YJR005C−A_2
YOL097W−A_2
BOI1
ALG3
DIE2
YDR535C
YCL026C−B_1
SNZ1
LSM7
YGL081W_2
YGL079W
PAC2
EDE1
YFR056C
LIF1
YBL086C
PTC3
YNL285W
ICY2
TEL1
YMR315W
YNL190W
BEM2
ATG23
SFK1
CPD1
YBL071C−B_2
YBL059W
YNL198C
YPR108W−A_2
YOR034C−A_2
YLR194C_2
YBL094C
YPL056C
RDR1
CLN2
YPL272C
SNZ2
YJR112W−A
YOR084W
RPL27B
PMT4
PRE5
DBP3
YLR184W
SKM1
YAL065C
MIG1
HDA3
SPO1
VPS71
HAA1
YMR259C
ILM1
YJL119C
HOC1
LGE1
YLR342W−A_2
ADA2
SHE1
YDR524W−A_2
YNL097C−A_2
PIB2
YDR034W−B_2
DCR2
CHL1
ZWF1
YLR124W
MET12
PIN4
YBR230W−A
AGA2
BBC1
YDR194W−A_2
ZDS1
YLR361C−A_2
VPS72
UBX3
YPL041C
VPS68
FRE3
GDH3
VPS4
Gene Name
YPD.K.Shift
HL.K.INTERACTION
CSH.K.INTERACTION
HL.AS.K.INTERACTION
YPEG.K.Shift
HLEG.K.INTERACTION
CSHEG.K.INTERACTION
HLEG.AS.K.INTERACTION
YPD.L.SHIFT
HL.L.INTERACTION
CSH.L.INTERACTION
HL.AS.L.INTERACTION
YPEG.L.SHIFT
HLEG.L.INTERACTION
CSHEG.L.INTERACTION
HLEG.AS.L.INTERACTION
Type of Media

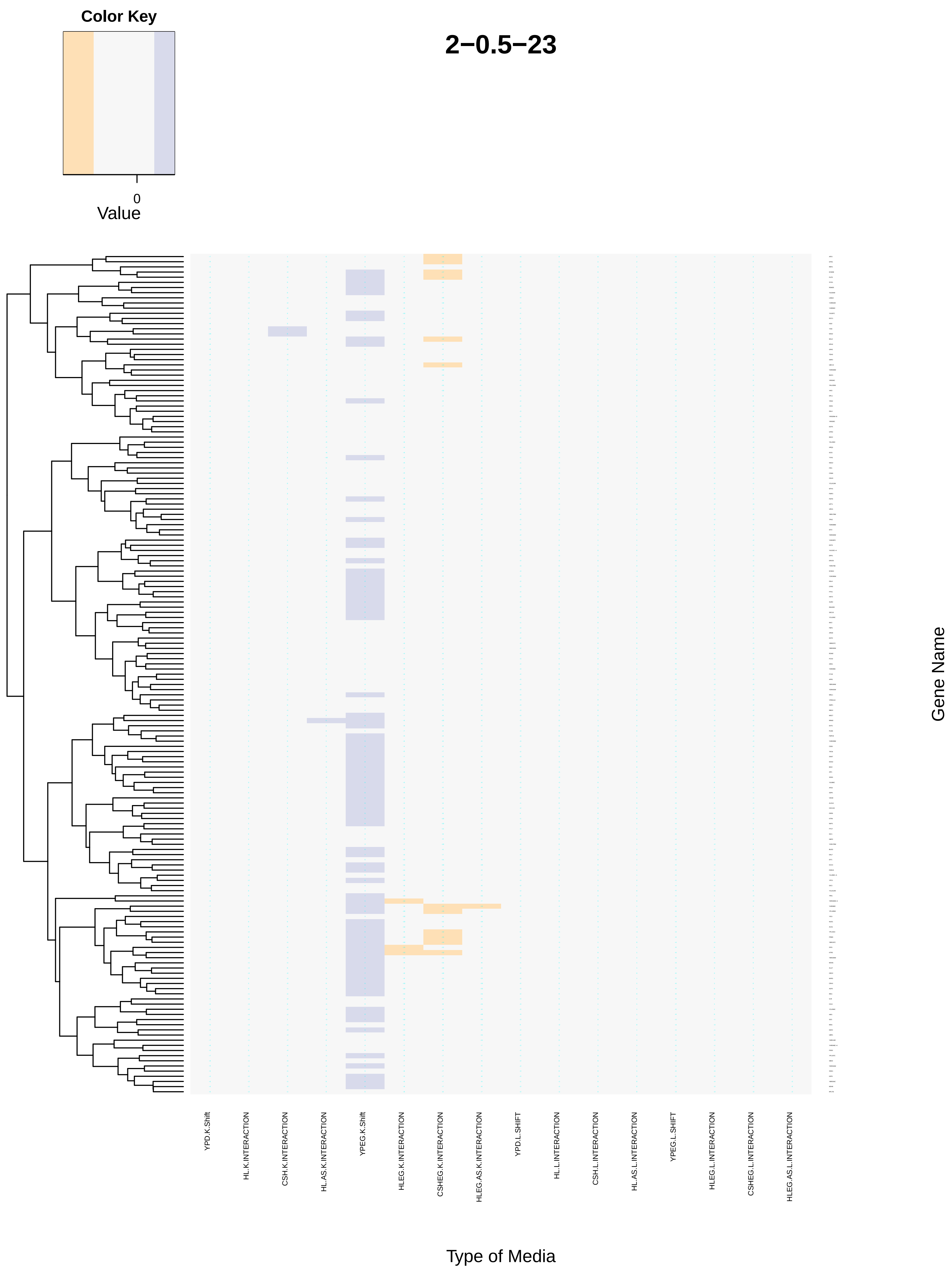
Color Key
0
Value
2−0.5−23
YPD.K.Shift
HL.K.INTERACTION
CSH.K.INTERACTION
HL.AS.K.INTERACTION
YPEG.K.Shift
HLEG.K.INTERACTION
CSHEG.K.INTERACTION
HLEG.AS.K.INTERACTION
YPD.L.SHIFT
HL.L.INTERACTION
CSH.L.INTERACTION
HL.AS.L.INTERACTION
YPEG.L.SHIFT
HLEG.L.INTERACTION
CSHEG.L.INTERACTION
HLEG.AS.L.INTERACTION
Type of Media
Gene Name

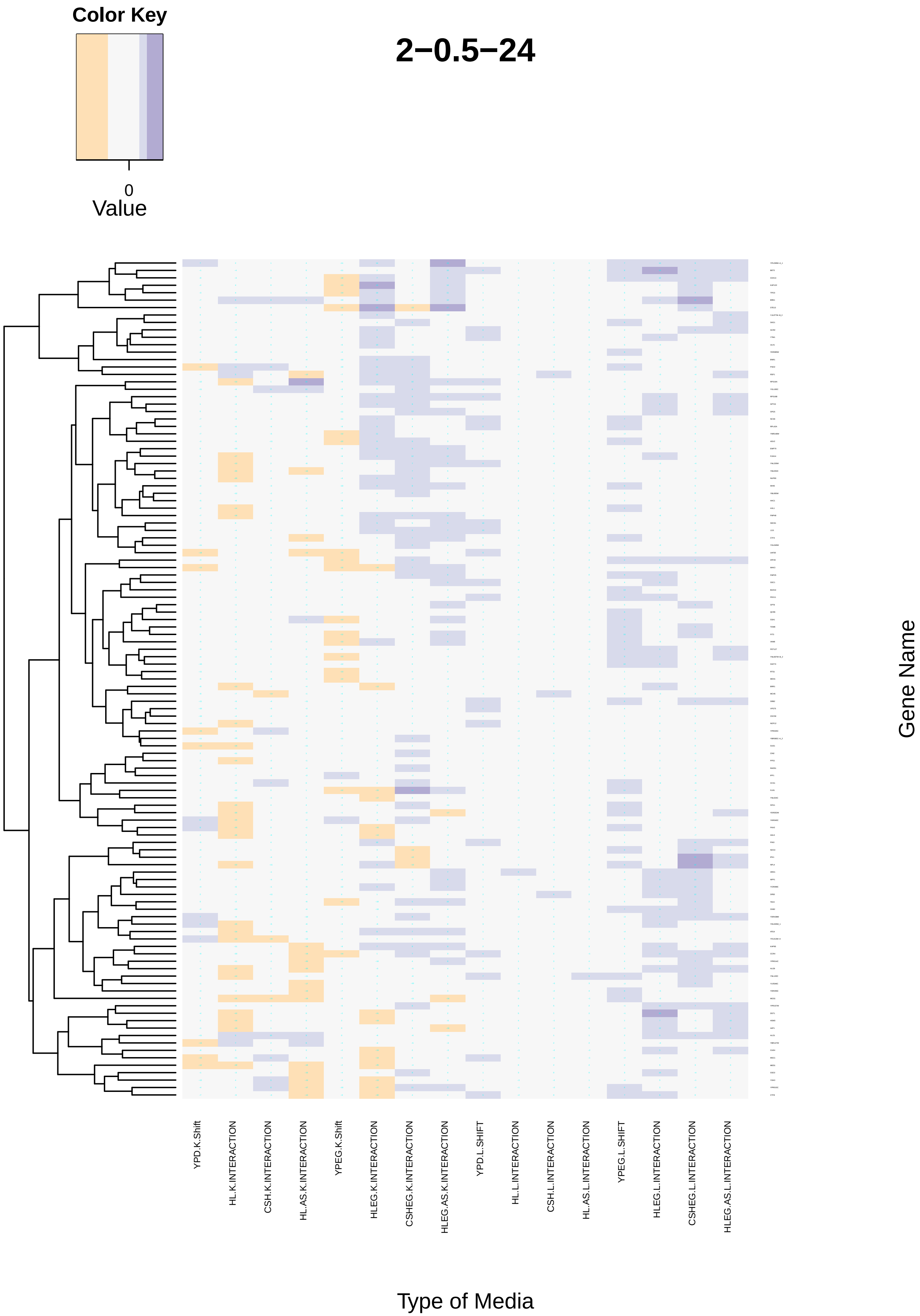

Color Key
0
Value
2−0.5−24
YPD.K.Shift
HL.K.INTERACTION
CSH.K.INTERACTION
HL.AS.K.INTERACTION
YPEG.K.Shift
HLEG.K.INTERACTION
CSHEG.K.INTERACTION
HLEG.AS.K.INTERACTION
YPD.L.SHIFT
HL.L.INTERACTION
CSH.L.INTERACTION
HL.AS.L.INTERACTION
YPEG.L.SHIFT
HLEG.L.INTERACTION
CSHEG.L.INTERACTION
HLEG.AS.L.INTERACTION
Gene Name
Type of Media

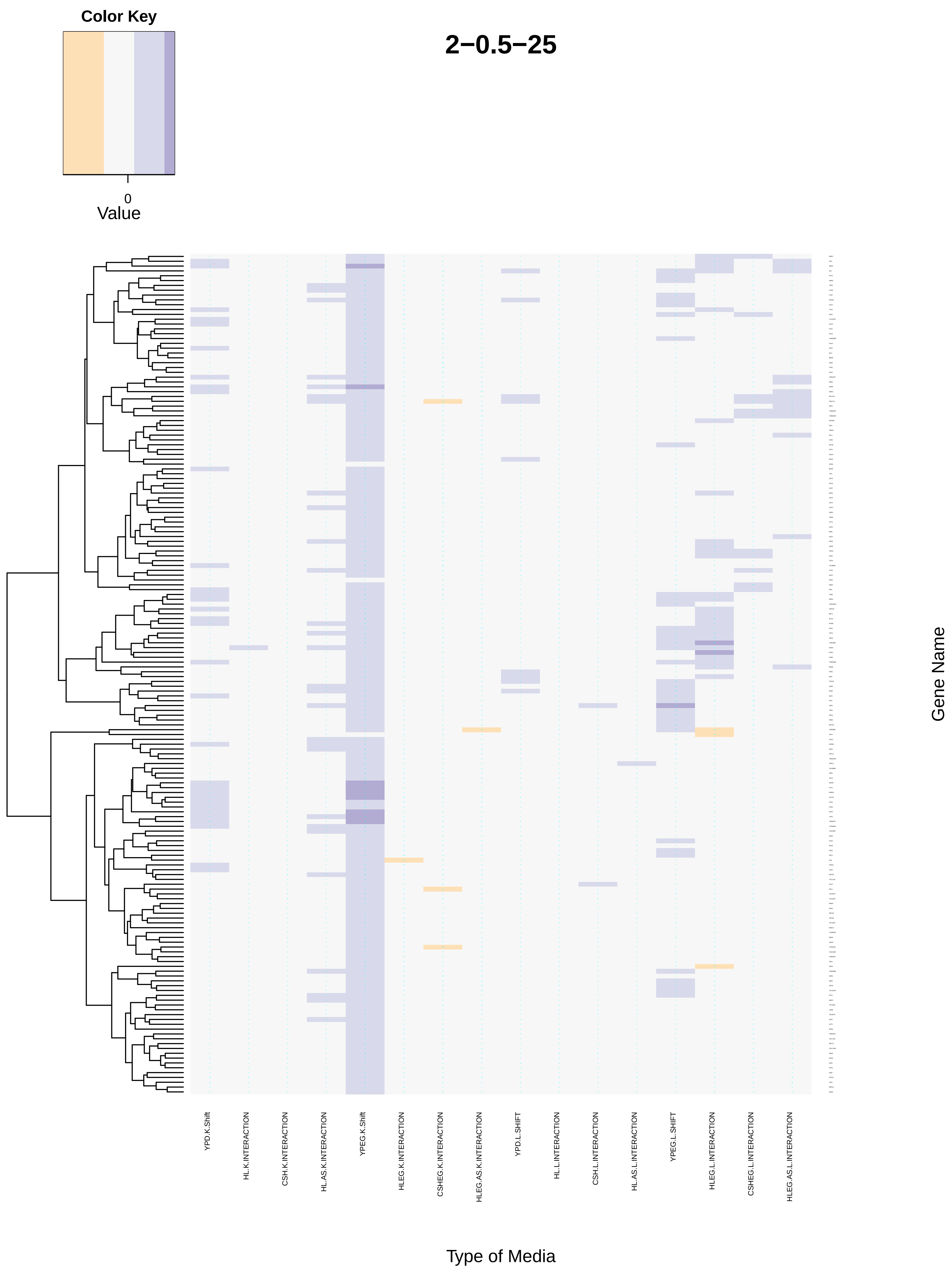
Color Key
0
Value
2−0.5−25
YPD.K.Shift
HL.K.INTERACTION
CSH.K.INTERACTION
HL.AS.K.INTERACTION
YPEG.K.Shift
HLEG.K.INTERACTION
CSHEG.K.INTERACTION
HLEG.AS.K.INTERACTION
YPD.L.SHIFT
HL.L.INTERACTION
CSH.L.INTERACTION
HL.AS.L.INTERACTION
YPEG.L.SHIFT
HLEG.L.INTERACTION
CSHEG.L.INTERACTION
HLEG.AS.L.INTERACTION
Type of Media
Gene Name

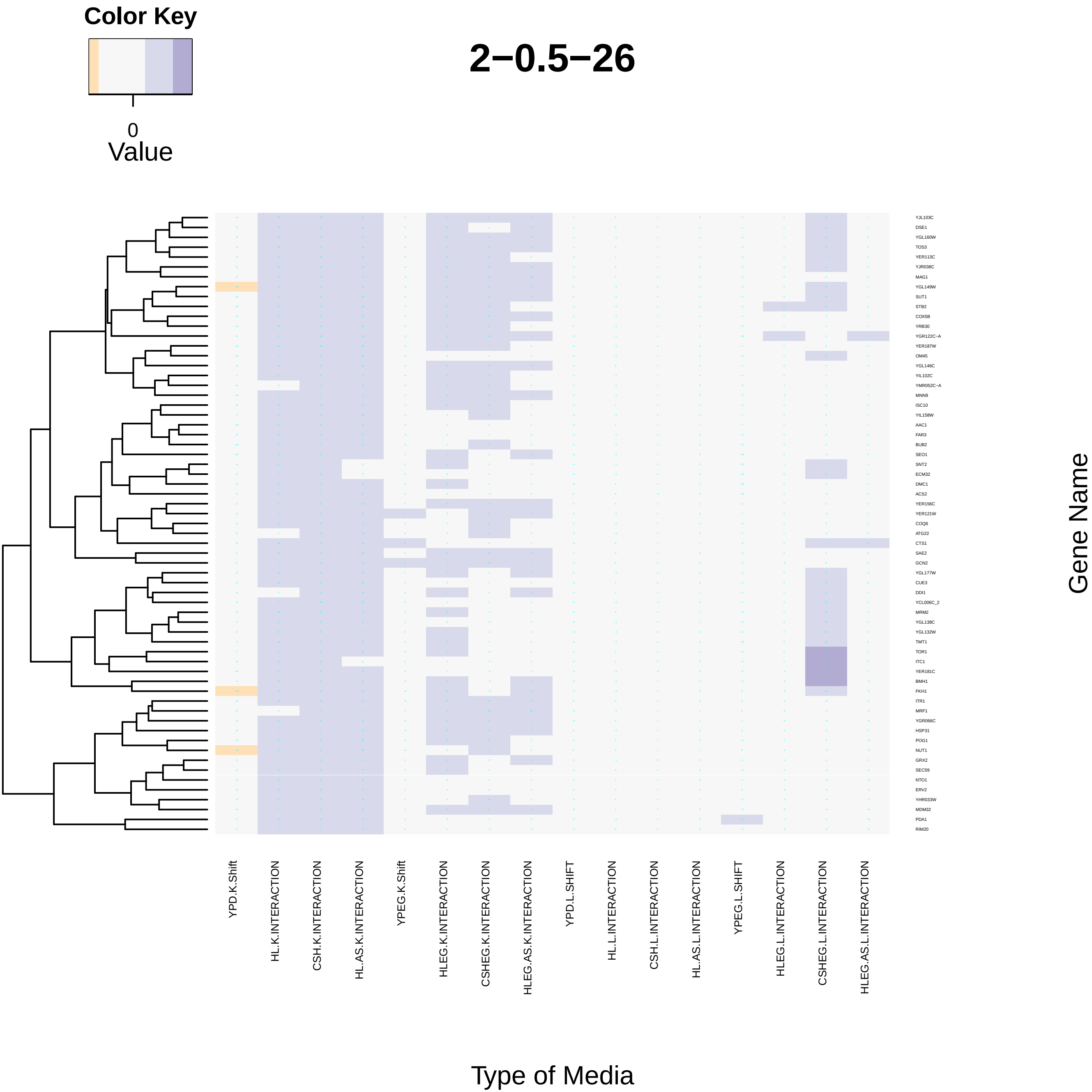
Color Key
0
Value
2−0.5−26
YPD.K.Shift
HL.K.INTERACTION
CSH.K.INTERACTION
HL.AS.K.INTERACTION
YPEG.K.Shift
HLEG.K.INTERACTION
CSHEG.K.INTERACTION
HLEG.AS.K.INTERACTION
YPD.L.SHIFT
HL.L.INTERACTION
CSH.L.INTERACTION
HL.AS.L.INTERACTION
YPEG.L.SHIFT
HLEG.L.INTERACTION
CSHEG.L.INTERACTION
HLEG.AS.L.INTERACTION
YJL103C
DSE1
YGL160W
TOS3
YER113C
YJR038C
MAG1
YGL149W
SUT1
STB2
COX5B
YRB30
YGR122C−A
YER187W
OM45
YGL146C
YIL102C
YMR052C−A
MNN9
ISC10
YIL158W
AAC1
FAR3
BUB2
SEO1
SNT2
ECM32
DMC1
ACS2
YER156C
YER121W
COQ6
ATG22
CTS1
SAE2
GCN2
YGL177W
CUE3
DDI1
YCL006C_2
MRM2
YGL138C
YGL132W
TMT1
TOR1
ITC1
YER181C
BMH1
FKH1
ITR1
MRF1
YGR066C
HSP31
POG1
NUT1
GRX2
SEC59
NTO1
ERV2
YHR033W
MDM32
PDA1
RIM20
Gene Name
Type of Media

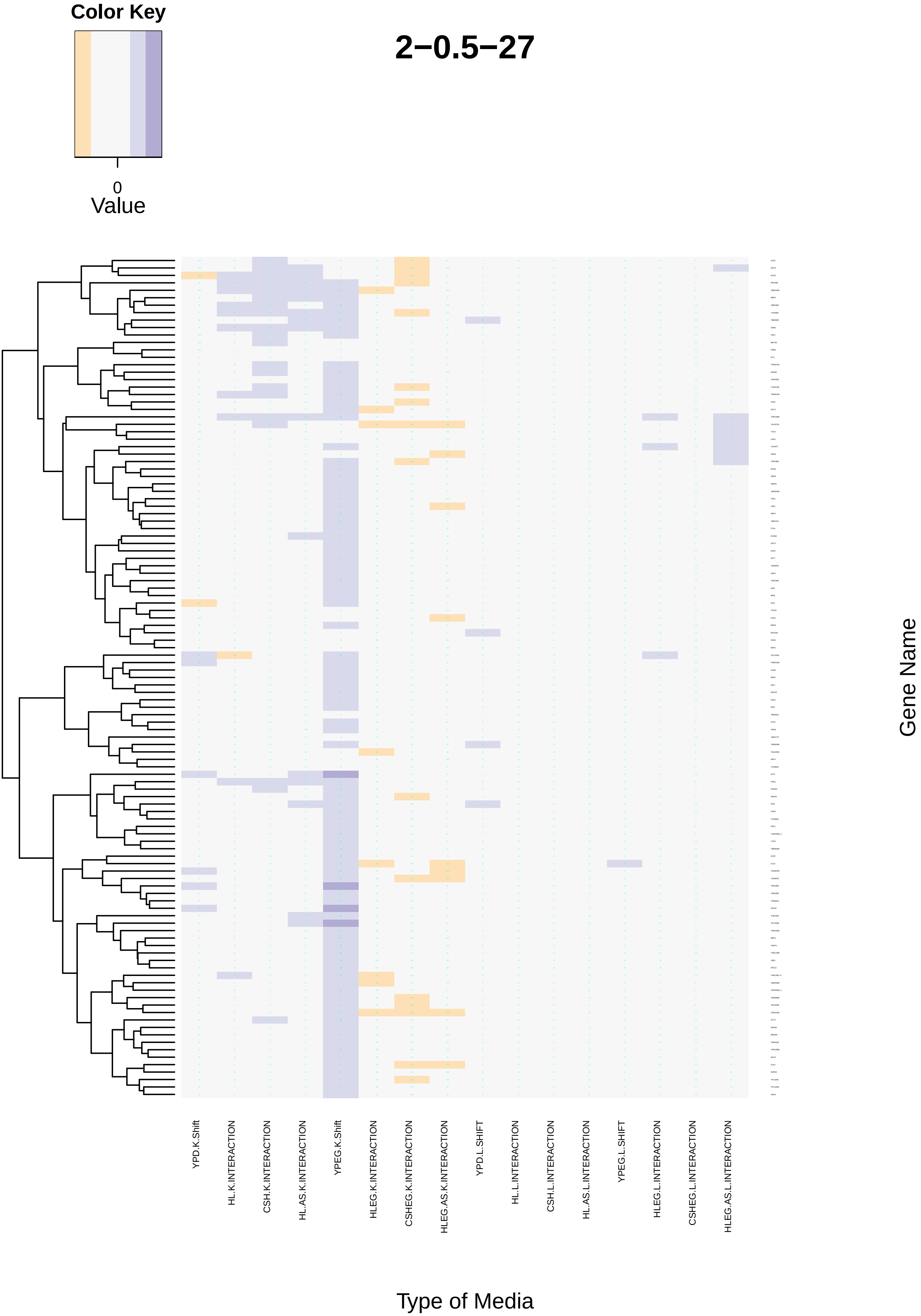
Color Key
0
Value
2−0.5−27
YPD.K.Shift
HL.K.INTERACTION
CSH.K.INTERACTION
HL.AS.K.INTERACTION
YPEG.K.Shift
HLEG.K.INTERACTION
CSHEG.K.INTERACTION
HLEG.AS.K.INTERACTION
YPD.L.SHIFT
HL.L.INTERACTION
CSH.L.INTERACTION
HL.AS.L.INTERACTION
YPEG.L.SHIFT
HLEG.L.INTERACTION
CSHEG.L.INTERACTION
HLEG.AS.L.INTERACTION
Gene Name
Type of Media

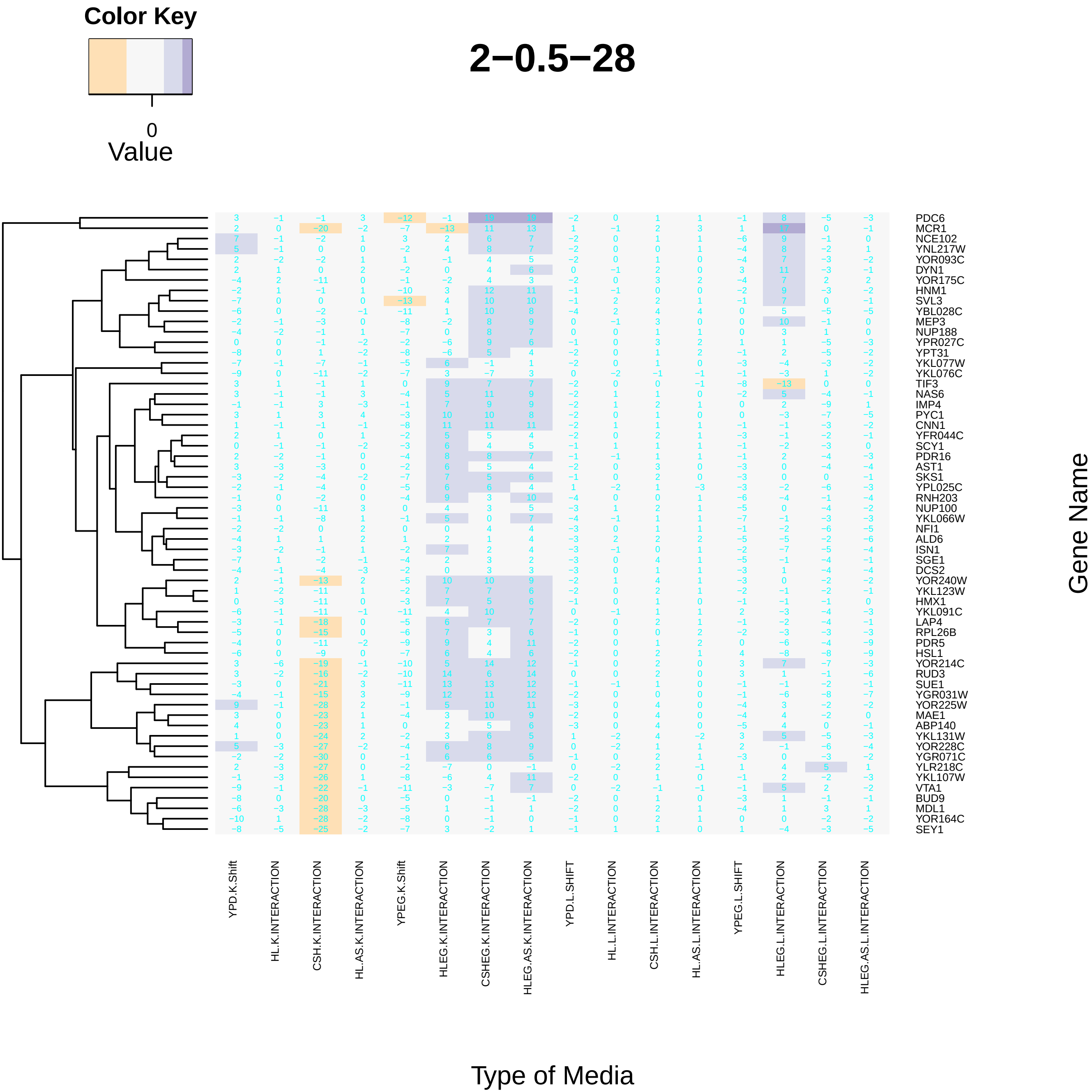

2−0.5−28
Color Key
0
Value
Type of Media
Gene Name
YPD.K.Shift
HL.K.INTERACTION
CSH.K.INTERACTION
HL.AS.K.INTERACTION
YPEG.K.Shift
HLEG.K.INTERACTION
CSHEG.K.INTERACTION
HLEG.AS.K.INTERACTION
YPD.L.SHIFT
HL.L.INTERACTION
CSH.L.INTERACTION
HL.AS.L.INTERACTION
YPEG.L.SHIFT
HLEG.L.INTERACTION
CSHEG.L.INTERACTION
HLEG.AS.L.INTERACTION
PDC6
MCR1
NCE102
YNL217W
YOR093C
DYN1
YOR175C
HNM1
SVL3
YBL028C
MEP3
NUP188
YPR027C
YPT31
YKL077W
YKL076C
TIF3
NAS6
IMP4
PYC1
CNN1
YFR044C
SCY1
PDR16
AST1
SKS1
YPL025C
RNH203
NUP100
YKL066W
NFI1
ALD6
ISN1
SGE1
DCS2
YOR240W
YKL123W
HMX1
YKL091C
LAP4
RPL26B
PDR5
HSL1
YOR214C
RUD3
SUE1
YGR031W
YOR225W
MAE1
ABP140
YKL131W
YOR228C
YGR071C
YLR218C
YKL107W
VTA1
BUD9
MDL1
YOR164C
SEY1

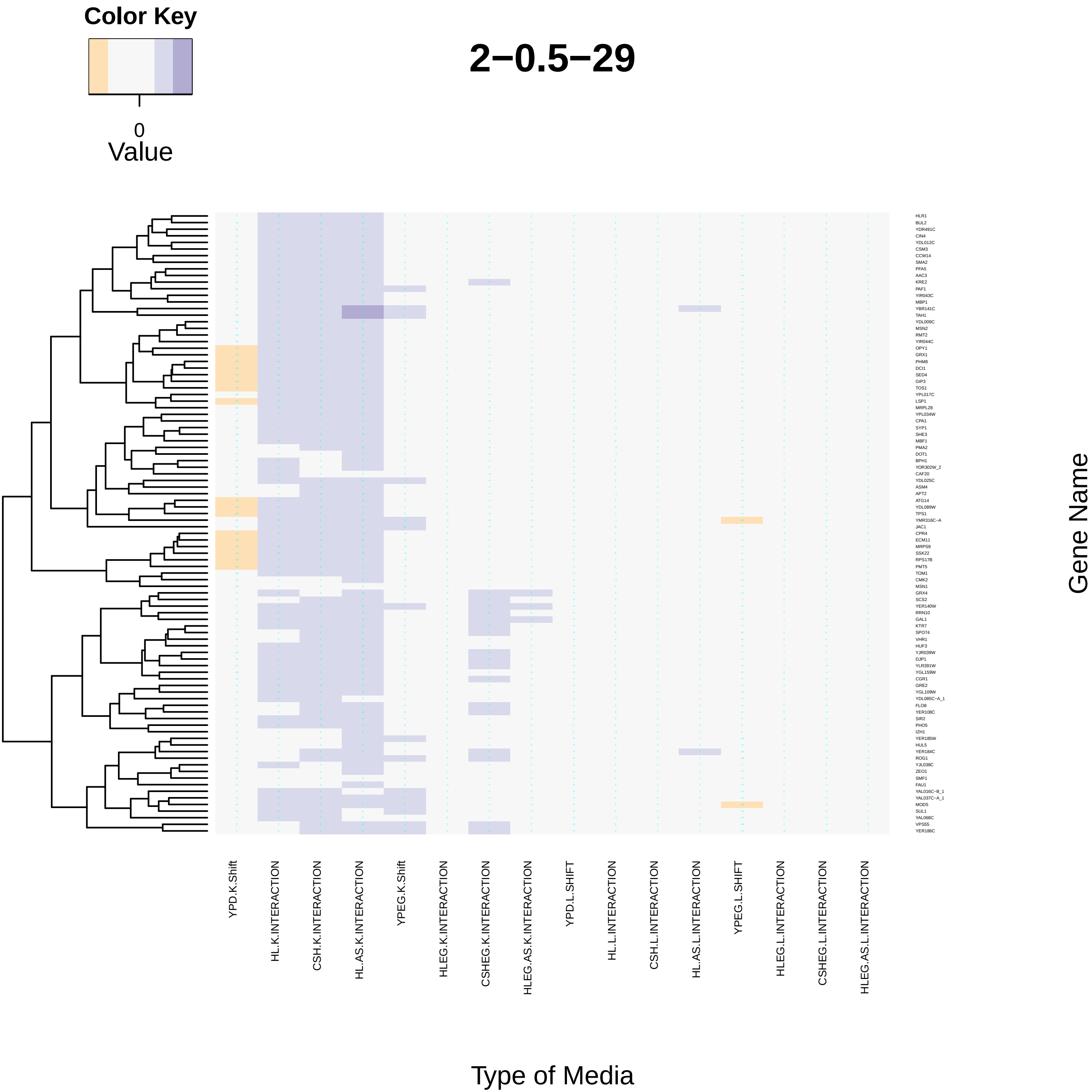
Color Key
0
Value
2−0.5−29
HLR1
BUL2
YDR491C
CIN4
YDL012C
CSM3
CCW14
SMA2
PFA5
AAC3
KRE2
PAF1
YIR043C
MBP1
YBR141C
TAH1
YDL009C
MSN2
RMT2
YIR044C
OPY1
GRX1
PHM8
DCI1
SED4
GIP3
TOS1
YPL017C
LSP1
MRPL28
YPL034W
CPA1
SYP1
SHE3
MBF1
PMA2
DOT1
BPH1
YOR302W_2
CAF20
YDL025C
ASM4
APT2
ATG14
YDL089W
TPS1
YMR316C−A
JAC1
CPR4
ECM11
MRPS9
SSK22
RPS17B
PMT5
TOM1
CMK2
MSN1
GRX4
SCS2
YER140W
RRN10
GAL1
KTR7
SPO74
VHR1
HUF3
YJR039W
DJP1
YLR391W
YGL159W
CGR1
GRE2
YGL109W
YDL085C−A_1
FLO8
YER108C
SIR2
PHO5
IZH1
YER185W
HUL5
YER184C
ROG1
YJL038C
ZEO1
SMF1
FAU1
YAL016C−B_1
YAL037C−A_1
MOD5
SUL1
YAL068C
VPS55
YER186C
Gene Name
YPD.K.Shift
HL.K.INTERACTION
CSH.K.INTERACTION
HL.AS.K.INTERACTION
YPEG.K.Shift
HLEG.K.INTERACTION
CSHEG.K.INTERACTION
HLEG.AS.K.INTERACTION
YPD.L.SHIFT
HL.L.INTERACTION
CSH.L.INTERACTION
HL.AS.L.INTERACTION
YPEG.L.SHIFT
HLEG.L.INTERACTION
CSHEG.L.INTERACTION
HLEG.AS.L.INTERACTION
Type of Media

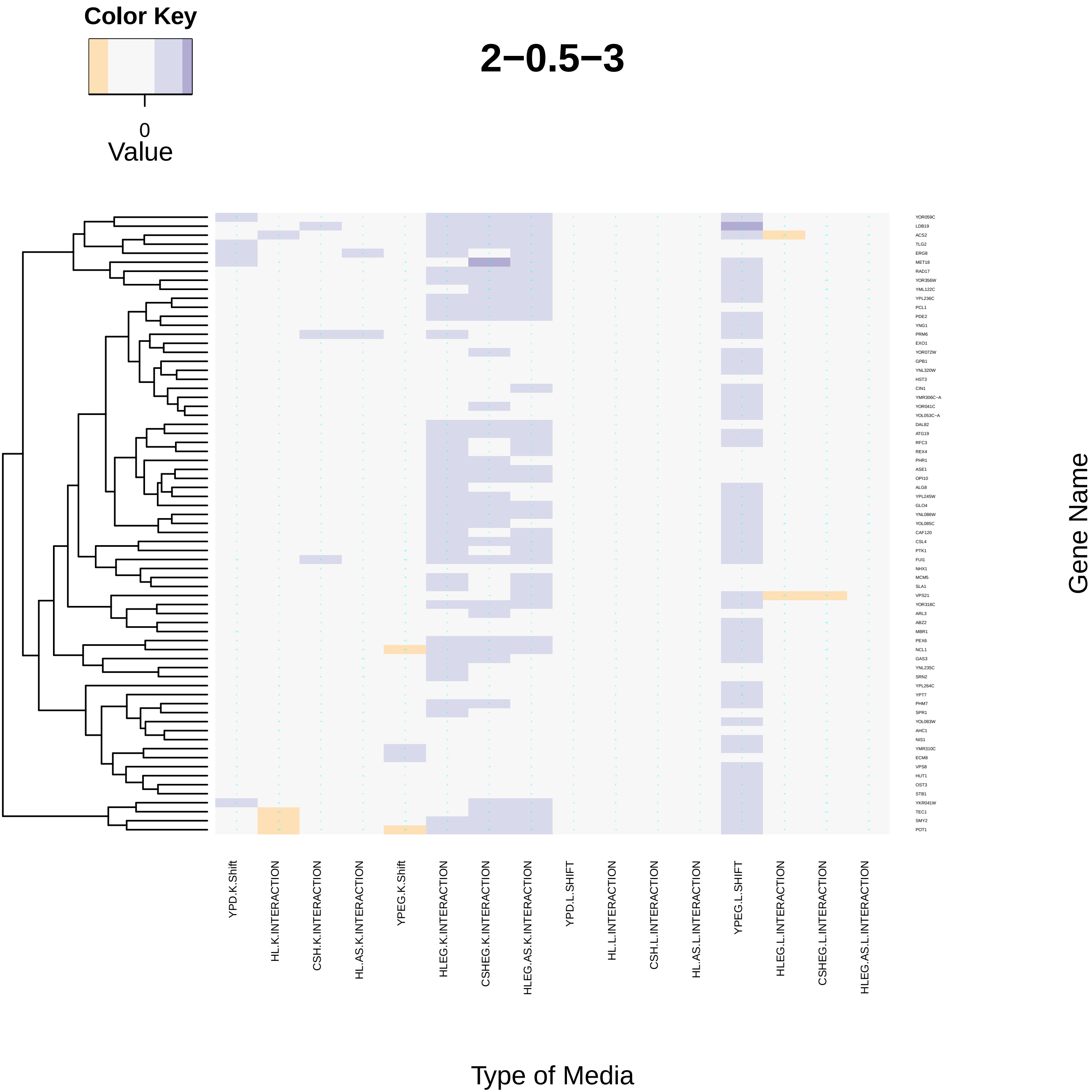

Color Key
0
Value
2−0.5−3
YOR059C
LDB19
ACS2
TLG2
ERG8
MET18
RAD17
YOR356W
YML122C
YPL236C
PCL1
PDE2
YNG1
PRM6
EXO1
YOR072W
GPB1
YNL320W
HST3
CIN1
YMR306C−A
YOR041C
YOL053C−A
DAL82
ATG19
RFC3
REX4
PHR1
ASE1
OPI10
ALG8
YPL245W
GLO4
YNL086W
YOL085C
CAF120
CSL4
PTK1
FUI1
NHX1
MCM5
SLA1
VPS21
YOR318C
ARL3
ABZ2
MBR1
PEX6
NCL1
GAS3
YNL235C
SRN2
YPL264C
YPT7
PHM7
SPR1
YOL083W
AHC1
NIS1
YMR310C
ECM8
VPS8
HUT1
OST3
STB1
YKR041W
TEC1
SMY2
POT1
Gene Name
YPD.K.Shift
HL.K.INTERACTION
CSH.K.INTERACTION
HL.AS.K.INTERACTION
YPEG.K.Shift
HLEG.K.INTERACTION
CSHEG.K.INTERACTION
HLEG.AS.K.INTERACTION
YPD.L.SHIFT
HL.L.INTERACTION
CSH.L.INTERACTION
HL.AS.L.INTERACTION
YPEG.L.SHIFT
HLEG.L.INTERACTION
CSHEG.L.INTERACTION
HLEG.AS.L.INTERACTION
Type of Media

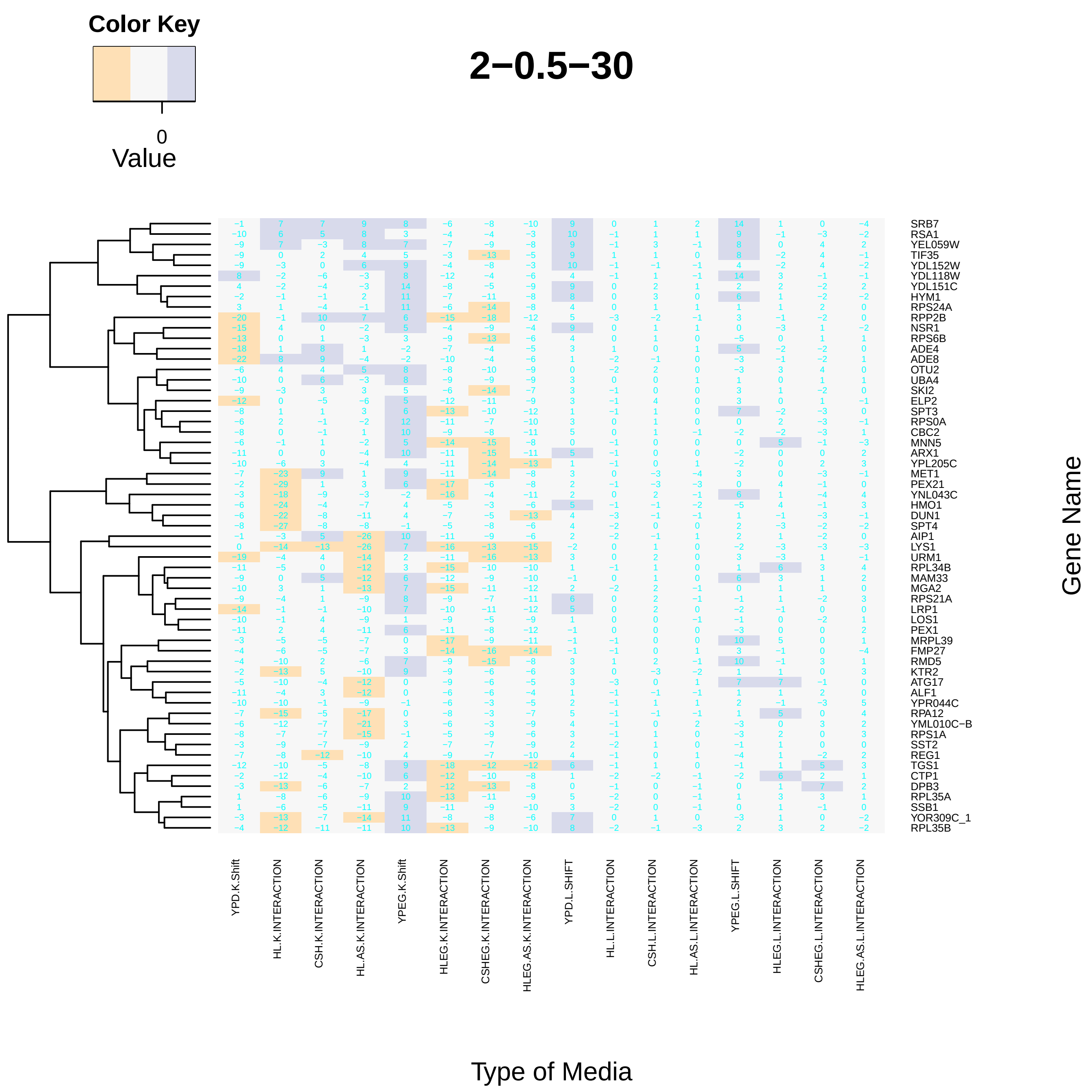
2−0.5−30
Color Key
0
Value
SRB7
RSA1
YEL059W
TIF35
YDL152W
YDL118W
YDL151C
HYM1
RPS24A
RPP2B
NSR1
RPS6B
ADE4
ADE8
OTU2
UBA4
SKI2
ELP2
SPT3
RPS0A
CBC2
MNN5
ARX1
YPL205C
MET1
PEX21
YNL043C
HMO1
DUN1
SPT4
AIP1
LYS1
URM1
RPL34B
MAM33
MGA2
RPS21A
LRP1
LOS1
PEX1
MRPL39
FMP27
RMD5
KTR2
ATG17
ALF1
YPR044C
RPA12
YML010C−B
RPS1A
SST2
REG1
TGS1
CTP1
DPB3
RPL35A
SSB1
YOR309C_1
RPL35B
Gene Name
YPD.K.Shift
HL.K.INTERACTION
CSH.K.INTERACTION
HL.AS.K.INTERACTION
YPEG.K.Shift
HLEG.K.INTERACTION
CSHEG.K.INTERACTION
HLEG.AS.K.INTERACTION
YPD.L.SHIFT
HL.L.INTERACTION
CSH.L.INTERACTION
HL.AS.L.INTERACTION
YPEG.L.SHIFT
HLEG.L.INTERACTION
CSHEG.L.INTERACTION
HLEG.AS.L.INTERACTION
Type of Media

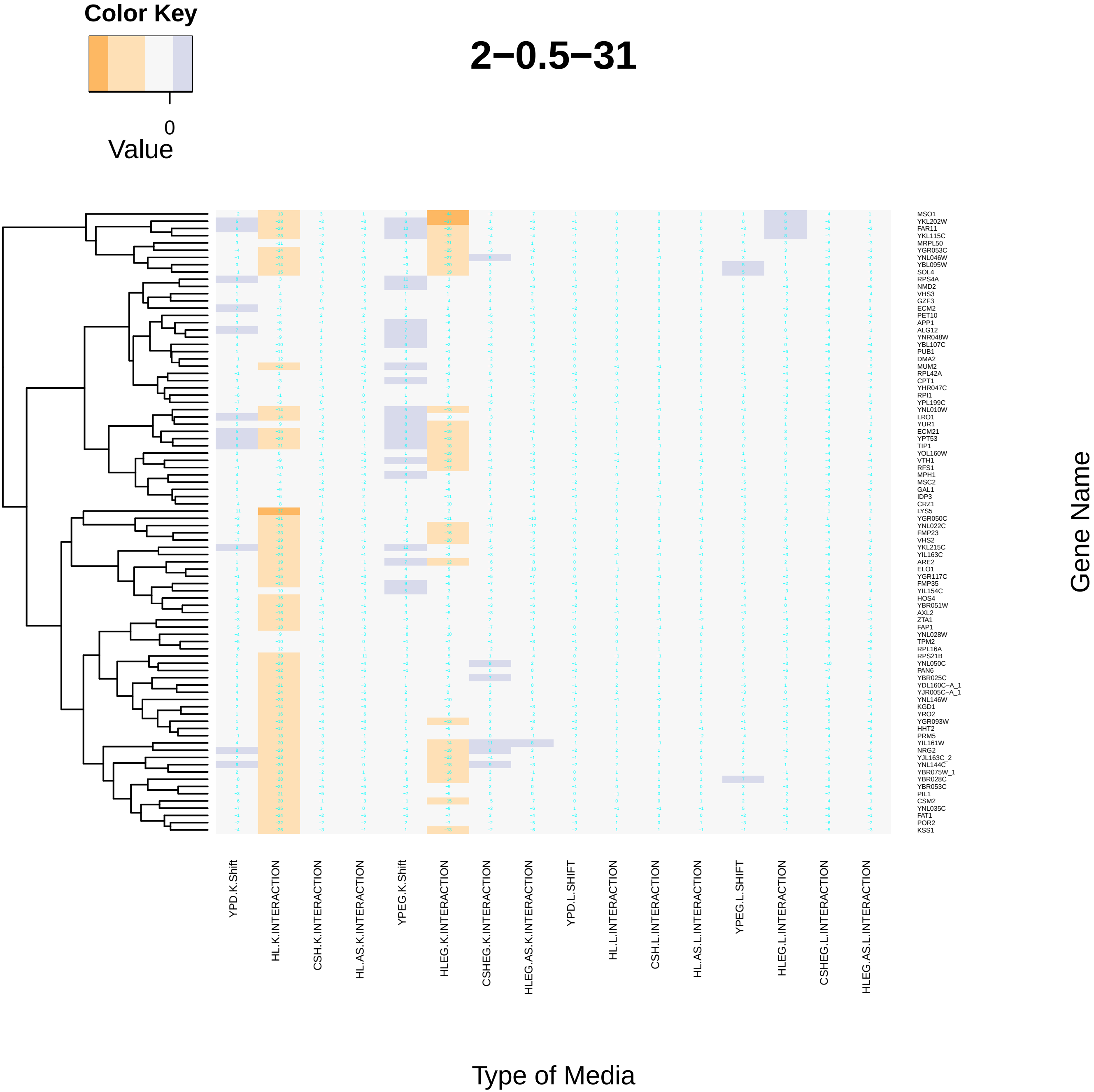
Color Key
0
Value
2−0.5−31
MSO1
YKL202W
FAR11
YKL115C
MRPL50
YGR053C
YNL046W
YBL095W
SOL4
RPS4A
NMD2
VHS3
GZF3
ECM2
PET10
APP1
ALG12
YNR048W
YBL107C
PUB1
DMA2
MUM2
RPL42A
CPT1
YHR047C
RPI1
YPL199C
YNL010W
LRO1
YUR1
ECM21
YPT53
TIP1
YOL160W
VTH1
RFS1
MPH1
MSC2
GAL1
IDP3
CRZ1
LYS5
YGR050C
YNL022C
FMP23
VHS2
YKL215C
YIL163C
ARE2
ELO1
YGR117C
FMP35
YIL154C
HOS4
YBR051W
AXL2
ZTA1
FAP1
YNL028W
TPM2
RPL16A
RPS21B
YNL050C
PAN6
YBR025C
YDL160C−A_1
YJR005C−A_1
YNL146W
KGD1
YRO2
YGR093W
HHT2
PRM5
YIL161W
NRG2
YJL163C_2
YNL144C
YBR075W_1
YBR028C
YBR053C
PIL1
CSM2
YNL035C
FAT1
POR2
KSS1
YPD.K.Shift
HL.K.INTERACTION
CSH.K.INTERACTION
HL.AS.K.INTERACTION
YPEG.K.Shift
HLEG.K.INTERACTION
CSHEG.K.INTERACTION
HLEG.AS.K.INTERACTION
YPD.L.SHIFT
HL.L.INTERACTION
CSH.L.INTERACTION
HL.AS.L.INTERACTION
YPEG.L.SHIFT
HLEG.L.INTERACTION
CSHEG.L.INTERACTION
HLEG.AS.L.INTERACTION
Type of Media
Gene Name

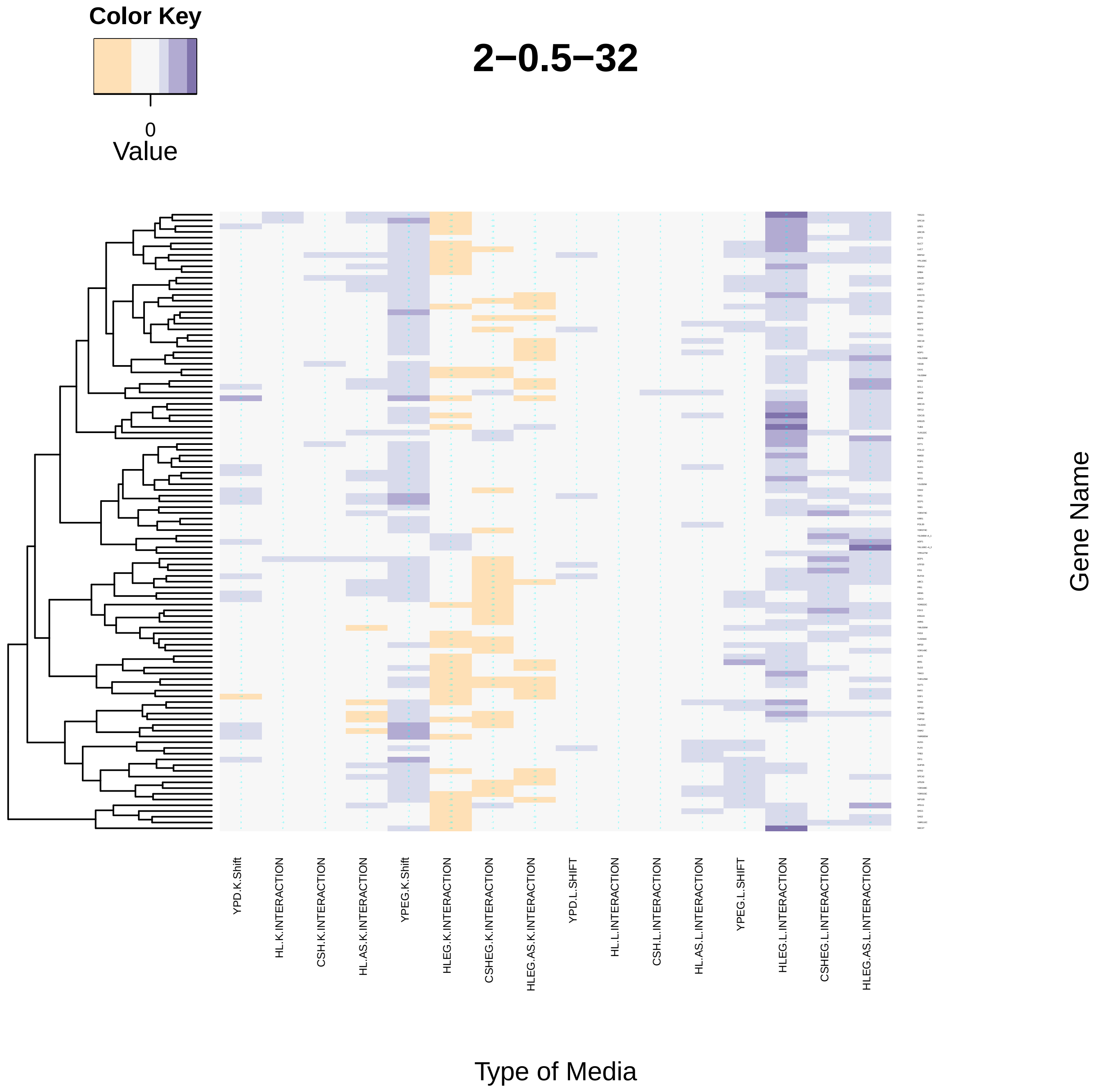
Color Key
0
Value
2−0.5−32
YPD.K.Shift
HL.K.INTERACTION
CSH.K.INTERACTION
HL.AS.K.INTERACTION
YPEG.K.Shift
HLEG.K.INTERACTION
CSHEG.K.INTERACTION
HLEG.AS.K.INTERACTION
YPD.L.SHIFT
HL.L.INTERACTION
CSH.L.INTERACTION
HL.AS.L.INTERACTION
YPEG.L.SHIFT
HLEG.L.INTERACTION
CSHEG.L.INTERACTION
HLEG.AS.L.INTERACTION
Gene Name
Type of Media

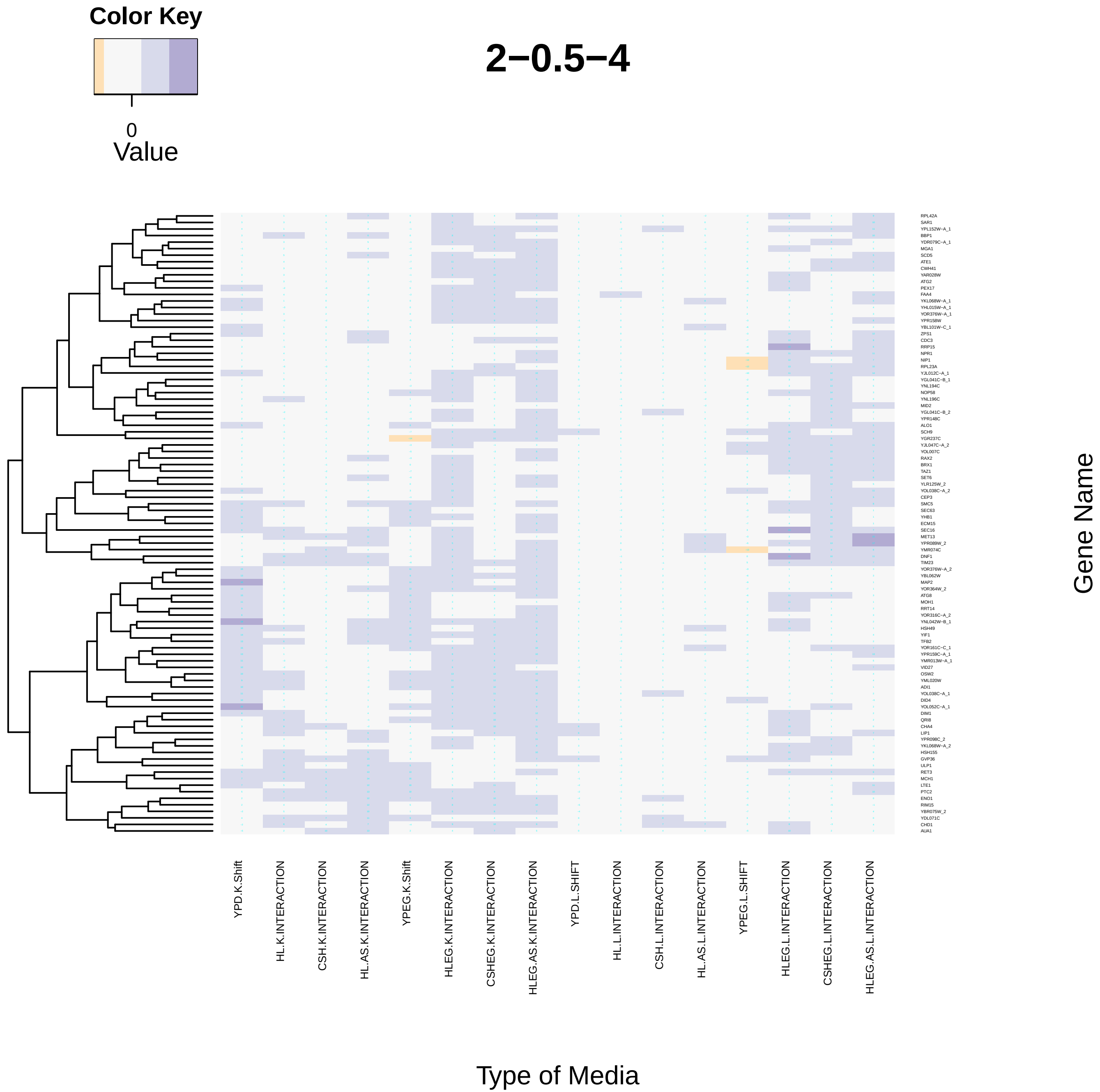
Color Key
0
Value
2−0.5−4
RPL42A
SAR1
YPL152W−A_1
BBP1
YDR079C−A_1
MGA1
SCD5
ATE1
CWH41
YAR028W
ATG2
PEX17
FAA4
YKL068W−A_1
YHL015W−A_1
YOR376W−A_1
YPR158W
YBL101W−C_1
ZPS1
CDC3
RRP15
NPR1
NIP1
RPL23A
YJL012C−A_1
YGL041C−B_1
YNL194C
NOP58
YNL196C
MID2
YGL041C−B_2
YPR148C
ALO1
SCH9
YGR237C
YJL047C−A_2
YOL007C
RAX2
BRX1
TAZ1
SET6
YLR125W_2
YOL038C−A_2
CEP3
SMC5
SEC63
YHB1
ECM15
SEC16
MET13
YPR089W_2
YMR074C
DNF1
TIM23
YOR376W−A_2
YBL062W
MAP2
YOR364W_2
ATG8
MOH1
RRT14
YOR316C−A_2
YNL042W−B_1
HSH49
YIF1
TFB2
YOR161C−C_1
YPR159C−A_1
YMR013W−A_1
VID27
OSW2
YML020W
ADI1
YOL038C−A_1
DID4
YOL052C−A_1
DIM1
QRI8
CHA4
LIP1
YPR098C_2
YKL068W−A_2
HSH155
GVP36
ULP1
RET3
MCH1
LTE1
PTC2
ENO1
RIM15
YBR075W_2
YDL071C
CHD1
AUA1
Gene Name
YPD.K.Shift
HL.K.INTERACTION
CSH.K.INTERACTION
HL.AS.K.INTERACTION
YPEG.K.Shift
HLEG.K.INTERACTION
CSHEG.K.INTERACTION
HLEG.AS.K.INTERACTION
YPD.L.SHIFT
HL.L.INTERACTION
CSH.L.INTERACTION
HL.AS.L.INTERACTION
YPEG.L.SHIFT
HLEG.L.INTERACTION
CSHEG.L.INTERACTION
HLEG.AS.L.INTERACTION
Type of Media

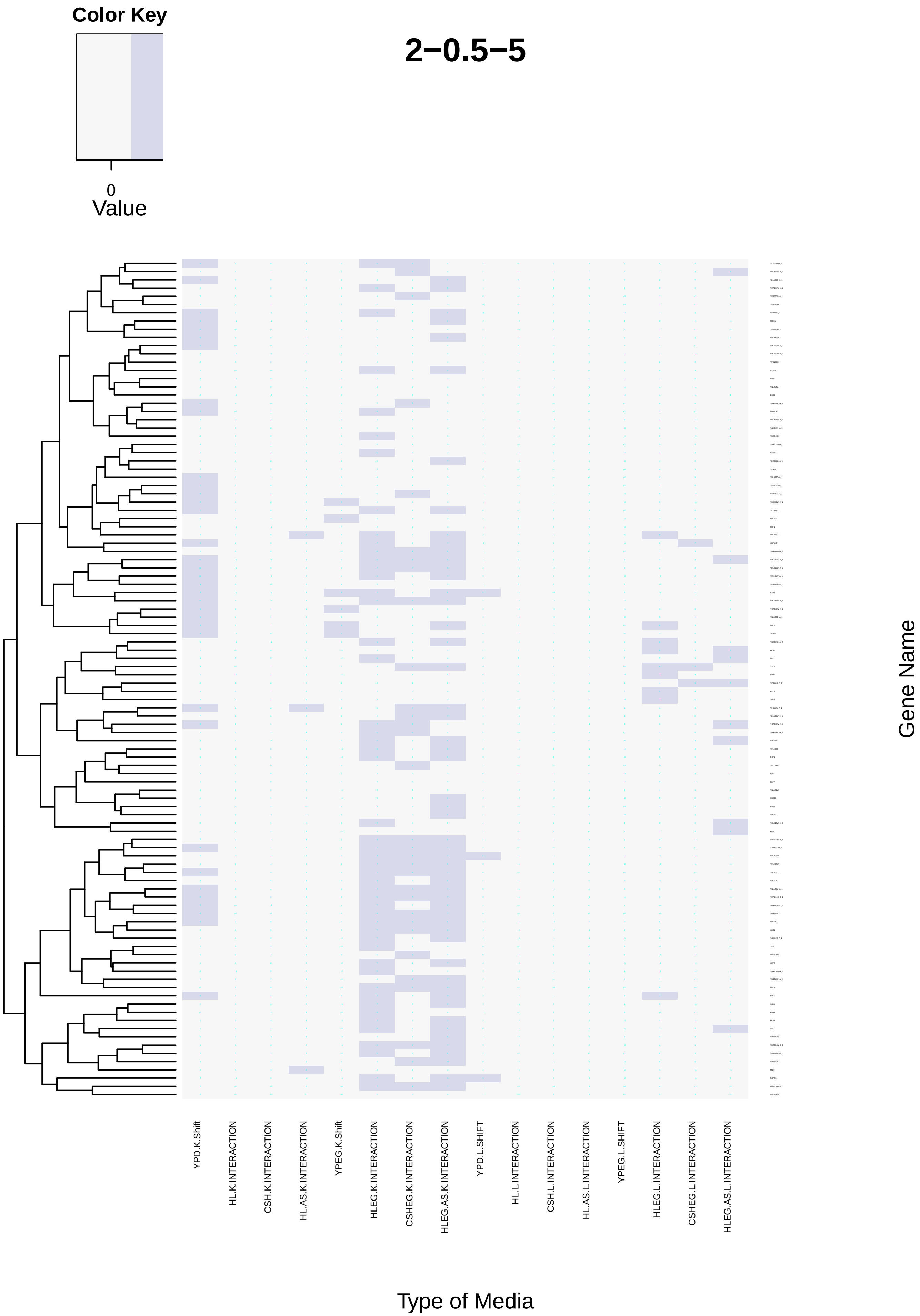
Color Key
0
Value
2−0.5−5
YPD.K.Shift
HL.K.INTERACTION
CSH.K.INTERACTION
HL.AS.K.INTERACTION
YPEG.K.Shift
HLEG.K.INTERACTION
CSHEG.K.INTERACTION
HLEG.AS.K.INTERACTION
YPD.L.SHIFT
HL.L.INTERACTION
CSH.L.INTERACTION
HL.AS.L.INTERACTION
YPEG.L.SHIFT
HLEG.L.INTERACTION
CSHEG.L.INTERACTION
HLEG.AS.L.INTERACTION
Gene Name
Type of Media

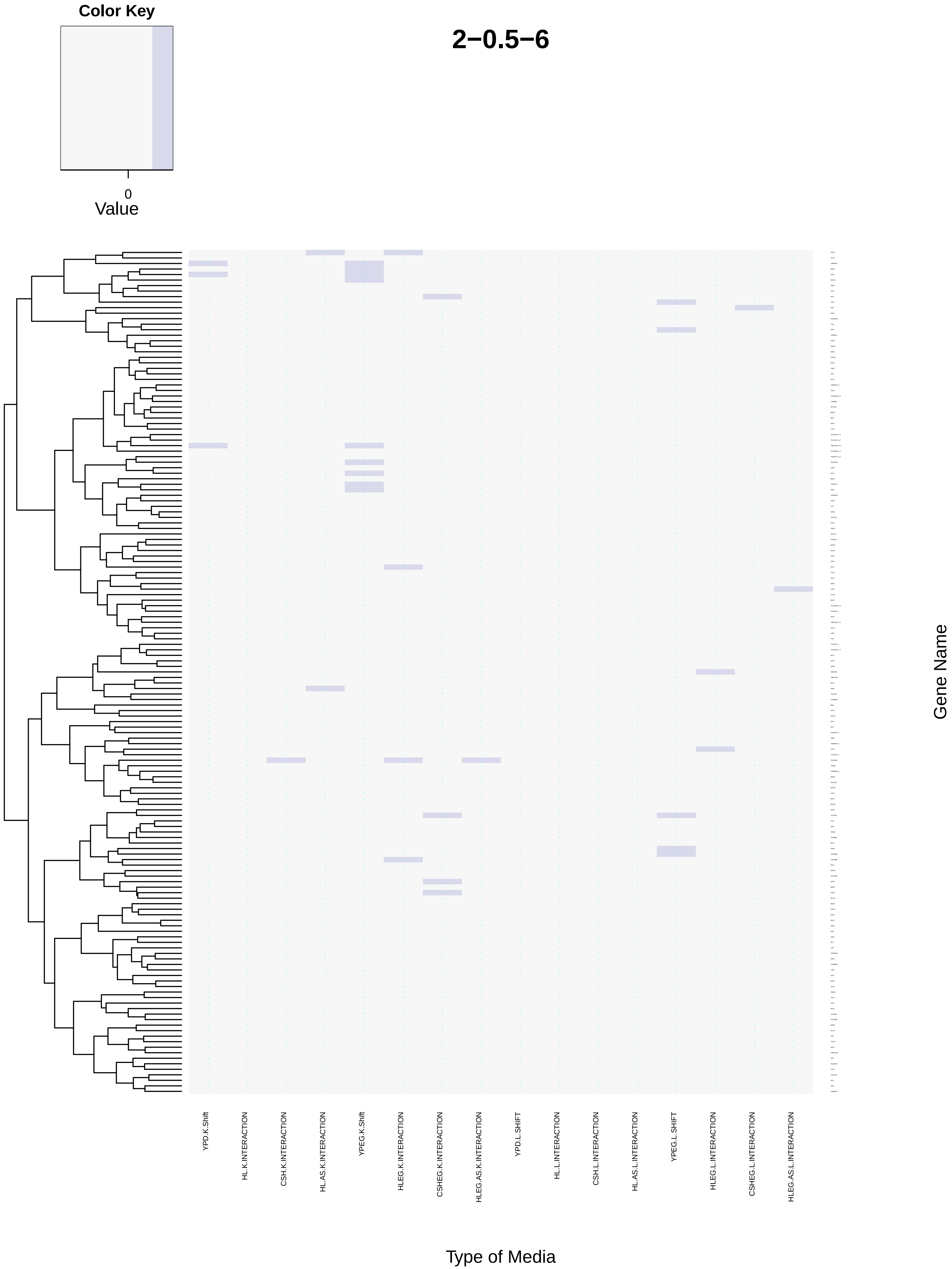
Color Key
0
Value
2−0.5−6
YPD.K.Shift
HL.K.INTERACTION
CSH.K.INTERACTION
HL.AS.K.INTERACTION
YPEG.K.Shift
HLEG.K.INTERACTION
CSHEG.K.INTERACTION
HLEG.AS.K.INTERACTION
YPD.L.SHIFT
HL.L.INTERACTION
CSH.L.INTERACTION
HL.AS.L.INTERACTION
YPEG.L.SHIFT
HLEG.L.INTERACTION
CSHEG.L.INTERACTION
HLEG.AS.L.INTERACTION
Type of Media
Gene Name

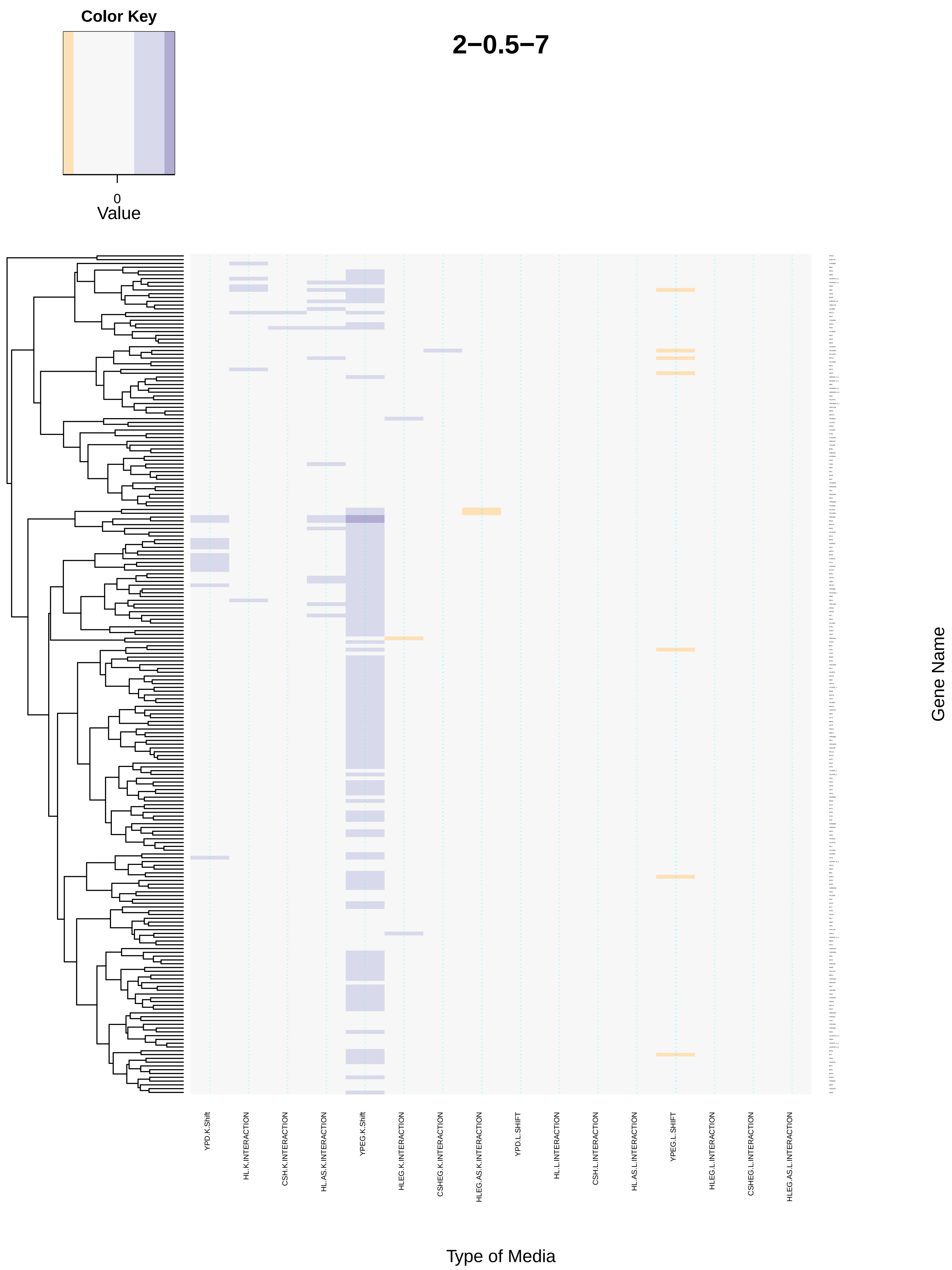

Color Key
0
Value
2−0.5−7
YPD.K.Shift
HL.K.INTERACTION
CSH.K.INTERACTION
HL.AS.K.INTERACTION
YPEG.K.Shift
HLEG.K.INTERACTION
CSHEG.K.INTERACTION
HLEG.AS.K.INTERACTION
YPD.L.SHIFT
HL.L.INTERACTION
CSH.L.INTERACTION
HL.AS.L.INTERACTION
YPEG.L.SHIFT
HLEG.L.INTERACTION
CSHEG.L.INTERACTION
HLEG.AS.L.INTERACTION
Type of Media
Gene Name

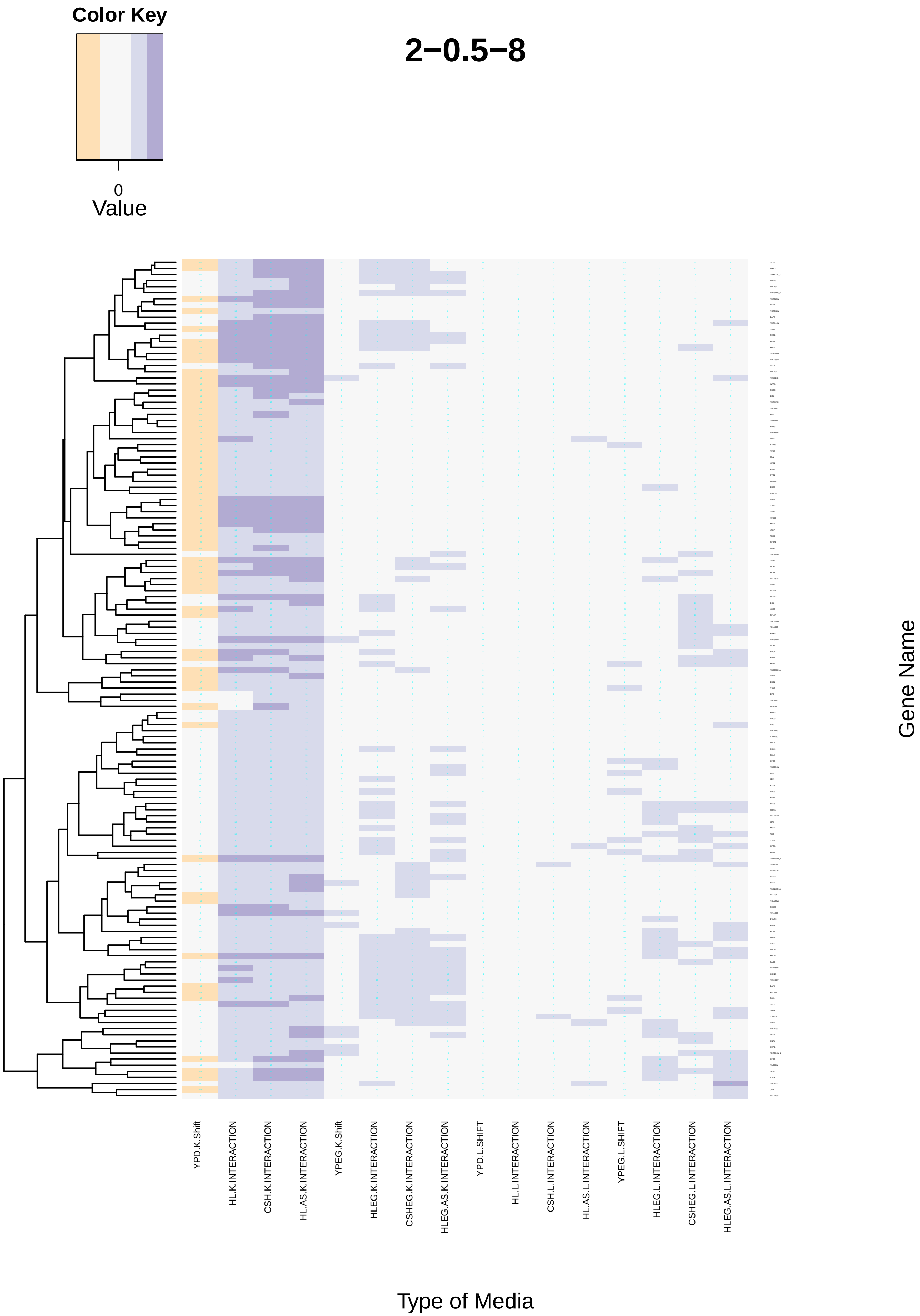
Color Key
0
Value
2−0.5−8
YPD.K.Shift
HL.K.INTERACTION
CSH.K.INTERACTION
HL.AS.K.INTERACTION
YPEG.K.Shift
HLEG.K.INTERACTION
CSHEG.K.INTERACTION
HLEG.AS.K.INTERACTION
YPD.L.SHIFT
HL.L.INTERACTION
CSH.L.INTERACTION
HL.AS.L.INTERACTION
YPEG.L.SHIFT
HLEG.L.INTERACTION
CSHEG.L.INTERACTION
HLEG.AS.L.INTERACTION
Gene Name
Type of Media

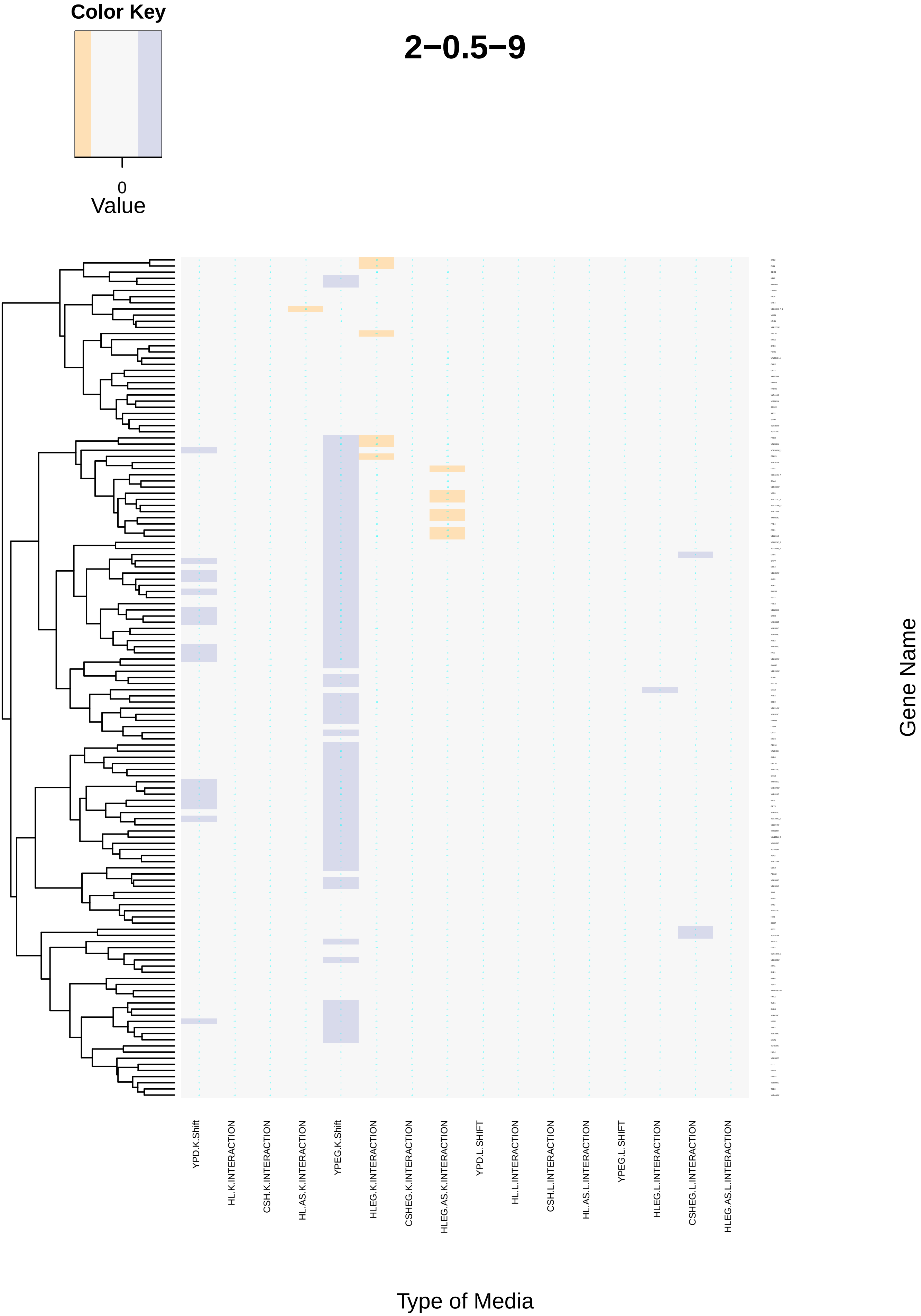
Color Key
0
Value
2−0.5−9
YPD.K.Shift
HL.K.INTERACTION
CSH.K.INTERACTION
HL.AS.K.INTERACTION
YPEG.K.Shift
HLEG.K.INTERACTION
CSHEG.K.INTERACTION
HLEG.AS.K.INTERACTION
YPD.L.SHIFT
HL.L.INTERACTION
CSH.L.INTERACTION
HL.AS.L.INTERACTION
YPEG.L.SHIFT
HLEG.L.INTERACTION
CSHEG.L.INTERACTION
HLEG.AS.L.INTERACTION
Type of Media
Gene Name

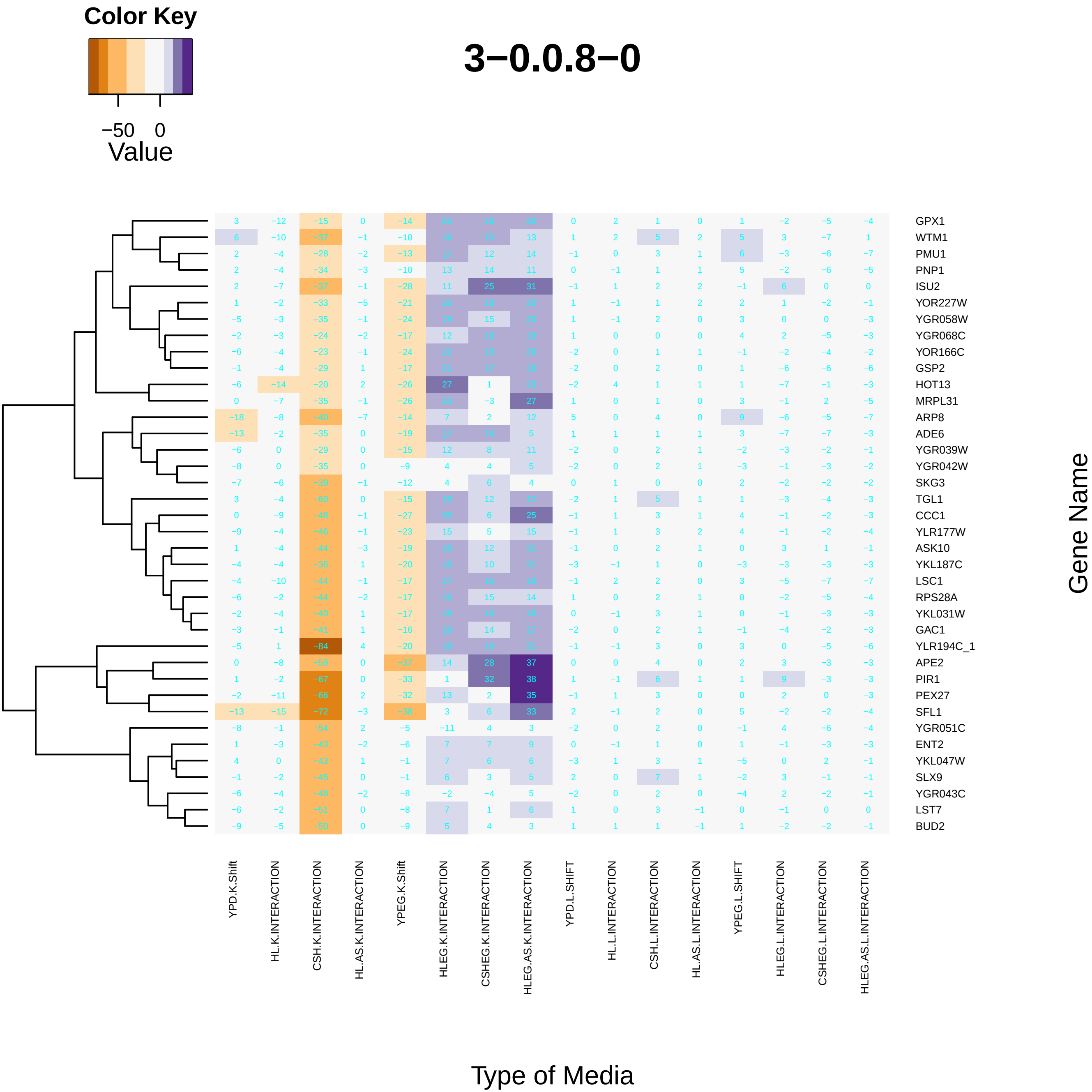
Color Key
−50
0
Value
3−0.0.8−0
GPX1
WTM1
PMU1
PNP1
ISU2
YOR227W
YGR058W
YGR068C
YOR166C
GSP2
HOT13
MRPL31
ARP8
ADE6
YGR039W
YGR042W
SKG3
TGL1
CCC1
YLR177W
ASK10
YKL187C
LSC1
RPS28A
YKL031W
GAC1
YLR194C_1
APE2
PIR1
PEX27
SFL1
YGR051C
ENT2
YKL047W
SLX9
YGR043C
LST7
BUD2
Gene Name
YPD.K.Shift
HL.K.INTERACTION
CSH.K.INTERACTION
HL.AS.K.INTERACTION
YPEG.K.Shift
HLEG.K.INTERACTION
CSHEG.K.INTERACTION
HLEG.AS.K.INTERACTION
YPD.L.SHIFT
HL.L.INTERACTION
CSH.L.INTERACTION
HL.AS.L.INTERACTION
YPEG.L.SHIFT
HLEG.L.INTERACTION
CSHEG.L.INTERACTION
HLEG.AS.L.INTERACTION
Type of Media

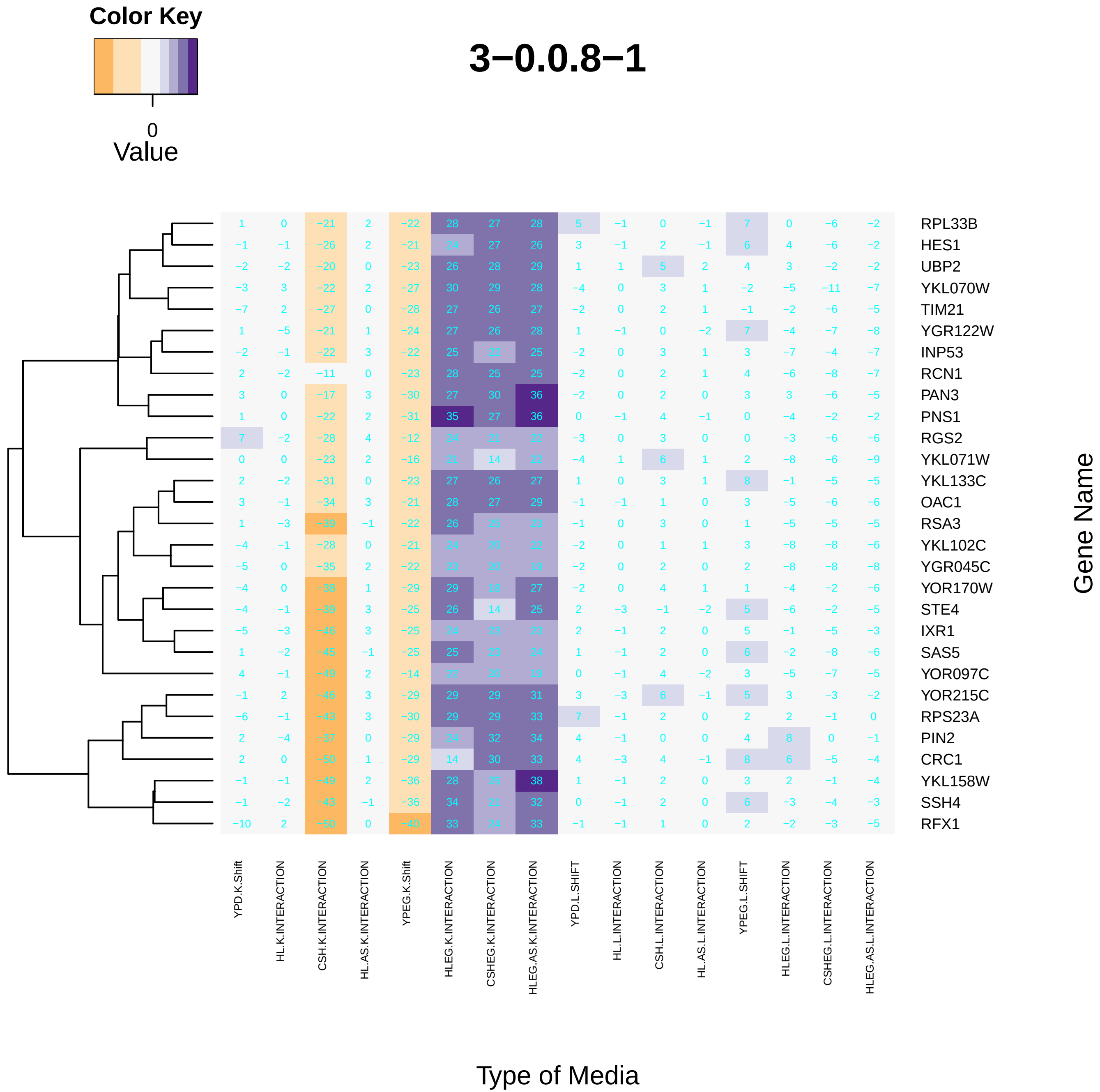

Color Key
0
Value
3−0.0.8−1
RPL33B: 1 0 −21 2 −22 28 27 28 5 −1 0 −1 7 0 −6 −2
HES1: −1 −1 −26 2 −21 24 27 26 3 −1 2 −1 6 4 −6 −2
UBP2: −2 −2 −20 0 −23 26 28 29 1 1 5 2 4 3 −2 −2
YKL070W: −3 3 −22 2 −27 30 29 28 −4 0 3 1 −2 −5 −11 −7
TIM21: −7 2 −27 0 −28 27 26 27 −2 0 2 1 −1 −2 −6 −5
YGR122W: 1 −5 −21 1 −24 27 26 28 1 −1 0 −2 7 −4 −7 −8
INP53: −2 −1 −22 3 −22 25 22 25 −2 0 3 1 3 −7 −4 −7
RCN1: 2 −2 −11 0 −23 28 25 25 −2 0 2 1 4 −6 −8 −7
PAN3: 3 0 −17 3 −30 27 30 36 −2 0 2 0 3 3 −6 −5
PNS1: 1 0 −22 2 −31 35 27 36 0 −1 4 −1 0 −4 −2 −2
RGS2: 7 −2 −28 4 −12 24 21 22 −3 0 3 0 0 −3 −6 −6
YKL071W: 0 0 −23 2 −16 21 14 22 −4 1 6 1 2 −8 −6 −9
YKL133C: 2 −2 −31 0 −23 27 26 27 1 0 3 1 8 −1 −5 −5
OAC1: 3 −1 −34 3 −21 28 27 29 −1 −1 1 0 3 −5 −6 −6
RSA3: 1 −3 −39 −1 −22 26 25 23 −1 0 3 0 1 −5 −5 −5
YKL102C: −4 −1 −28 0 −21 24 20 22 −2 0 1 1 3 −8 −8 −6
YGR045C: −5 0 −35 2 −22 23 20 19 −2 0 2 0 2 −8 −8 −8
YOR170W: −4 0 −38 1 −29 29 18 27 −2 0 4 1 1 −4 −2 −6
STE4: −4 −1 −39 3 −25 26 14 25 2 −3 −1 −2 5 −6 −2 −5
IXR1: −5 −3 −46 3 −25 24 23 23 2 −1 2 0 5 −1 −5 −3
SAS5: 1 −2 −45 −1 −25 25 23 24 1 −1 2 0 6 −2 −8 −6
YOR097C: 4 −1 −49 2 −14 22 20 19 0 −1 4 −2 3 −5 −7 −5
YOR215C: −1 2 −46 3 −29 29 29 31 3 −3 6 −1 5 3 −3 −2
RPS23A: −6 −1 −43 3 −30 29 29 33 7 −1 2 0 2 2 −1 0
PIN2: 2 −4 −37 0 −29 24 32 34 4 −1 0 0 4 8 0 −1
CRC1: 2 0 −50 1 −29 14 30 33 4 −3 4 −1 8 6 −5 −4
YKL158W: −1 −1 −49 2 −36 28 25 38 1 −1 2 0 3 2 −1 −4
SSH4: −1 −2 −43 −1 −36 34 21 32 0 −1 2 0 6 −3 −4 −3
RFX1: −10 2 −50 0 −40 33 24 33 −1 −1 1 0 2 −2 −3 −5
YPD.K.Shift
HL.K.INTERACTION
CSH.K.INTERACTION
HL.AS.K.INTERACTION
YPEG.K.Shift
HLEG.K.INTERACTION
CSHEG.K.INTERACTION
HLEG.AS.K.INTERACTION
YPD.L.SHIFT
HL.L.INTERACTION
CSH.L.INTERACTION
HL.AS.L.INTERACTION
YPEG.L.SHIFT
HLEG.L.INTERACTION
CSHEG.L.INTERACTION
HLEG.AS.L.INTERACTION
Gene Name
Type of Media

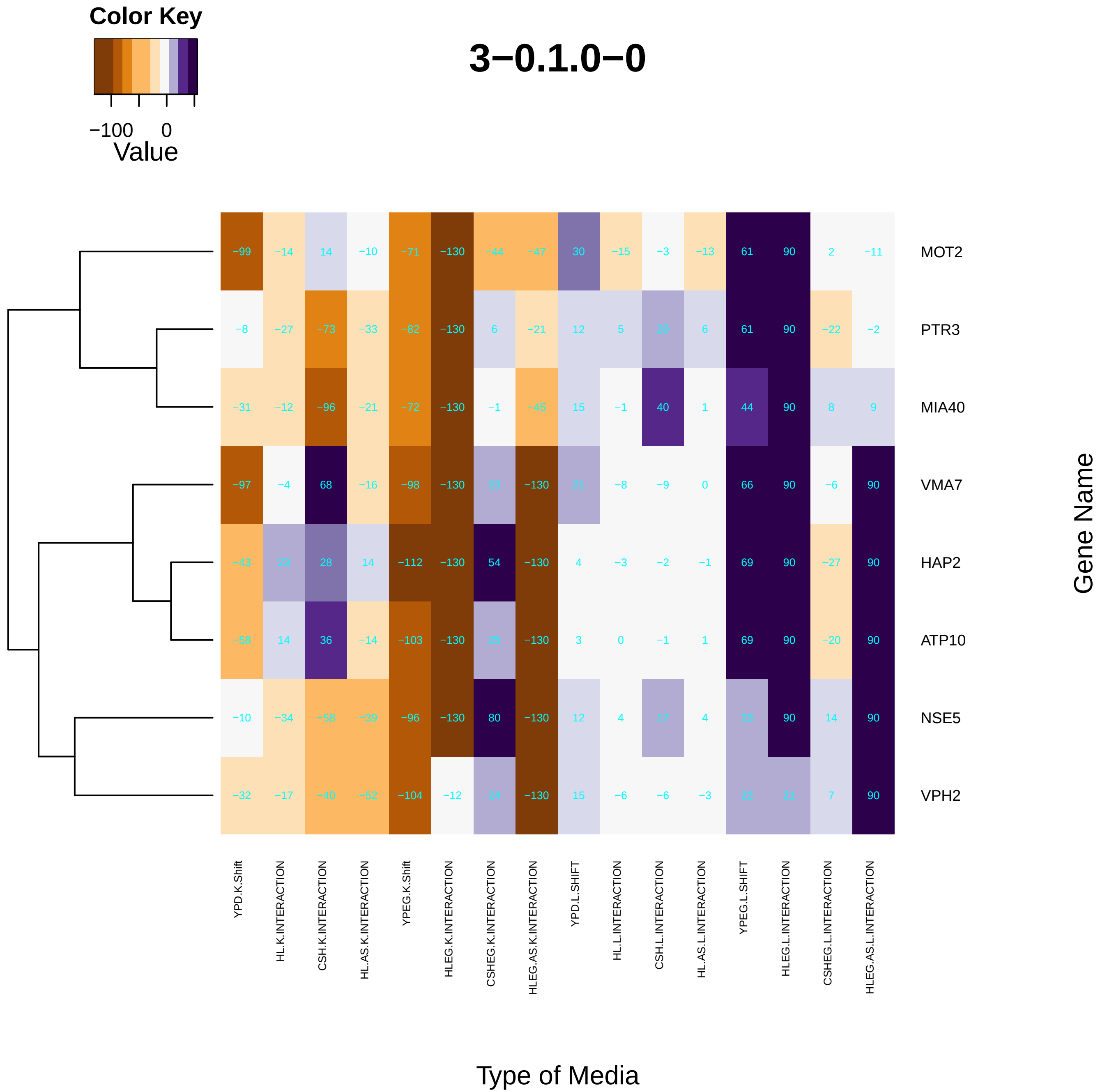

Color Key
−100
0
Value
3−0.1.0−0
MOT2
PTR3
MIA40
VMA7
HAP2
ATP10
NSE5
VPH2
Gene Name
YPD.K.Shift
HL.K.INTERACTION
CSH.K.INTERACTION
HL.AS.K.INTERACTION
YPEG.K.Shift
HLEG.K.INTERACTION
CSHEG.K.INTERACTION
HLEG.AS.K.INTERACTION
YPD.L.SHIFT
HL.L.INTERACTION
CSH.L.INTERACTION
HL.AS.L.INTERACTION
YPEG.L.SHIFT
HLEG.L.INTERACTION
CSHEG.L.INTERACTION
HLEG.AS.L.INTERACTION
Type of Media

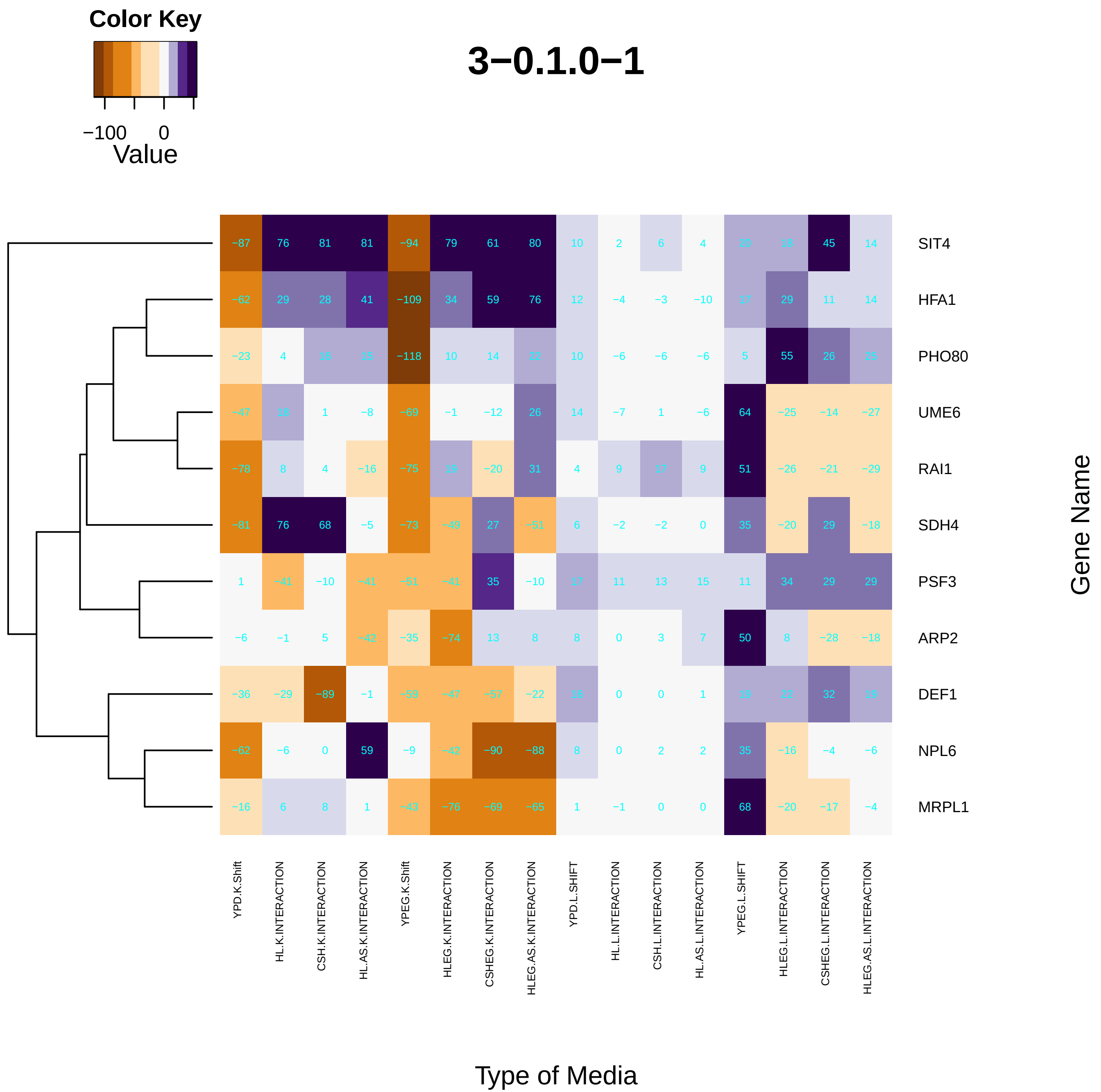
Color Key
−100
0
Value
3−0.1.0−1
SIT4
HFA1
PHO80
UME6
RAI1
SDH4
PSF3
ARP2
DEF1
NPL6
MRPL1
Gene Name
YPD.K.Shift
HL.K.INTERACTION
CSH.K.INTERACTION
HL.AS.K.INTERACTION
YPEG.K.Shift
HLEG.K.INTERACTION
CSHEG.K.INTERACTION
HLEG.AS.K.INTERACTION
YPD.L.SHIFT
HL.L.INTERACTION
CSH.L.INTERACTION
HL.AS.L.INTERACTION
YPEG.L.SHIFT
HLEG.L.INTERACTION
CSHEG.L.INTERACTION
HLEG.AS.L.INTERACTION
Type of Media

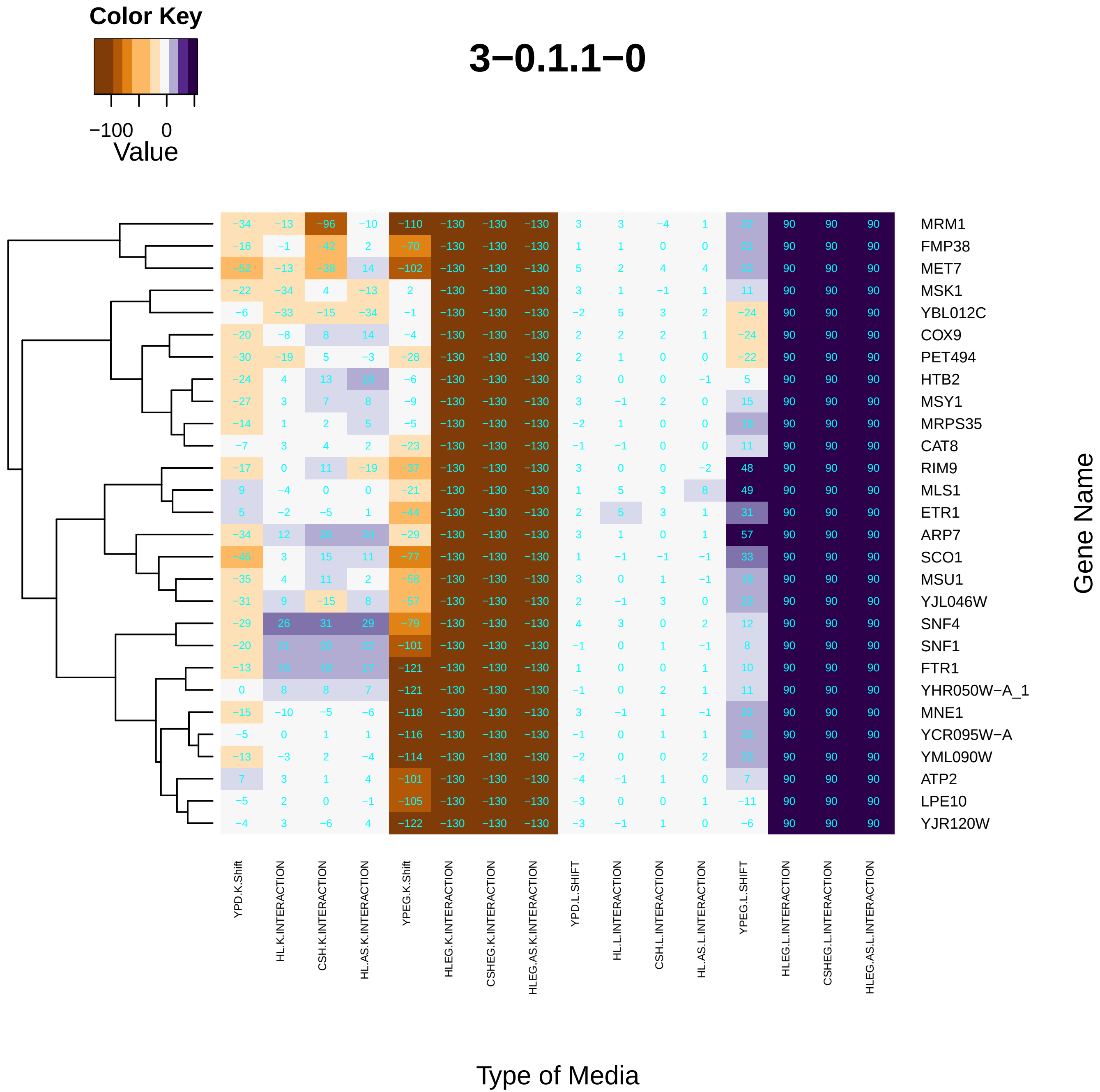
Color Key
−100
0
Value
3−0.1.1−0
MRM1
FMP38
MET7
MSK1
YBL012C
COX9
PET494
HTB2
MSY1
MRPS35
CAT8
RIM9
MLS1
ETR1
ARP7
SCO1
MSU1
YJL046W
SNF4
SNF1
FTR1
YHR050W−A_1
MNE1
YCR095W−A
YML090W
ATP2
LPE10
YJR120W
Gene Name
YPD.K.Shift
HL.K.INTERACTION
CSH.K.INTERACTION
HL.AS.K.INTERACTION
YPEG.K.Shift
HLEG.K.INTERACTION
CSHEG.K.INTERACTION
HLEG.AS.K.INTERACTION
YPD.L.SHIFT
HL.L.INTERACTION
CSH.L.INTERACTION
HL.AS.L.INTERACTION
YPEG.L.SHIFT
HLEG.L.INTERACTION
CSHEG.L.INTERACTION
HLEG.AS.L.INTERACTION
Type of Media

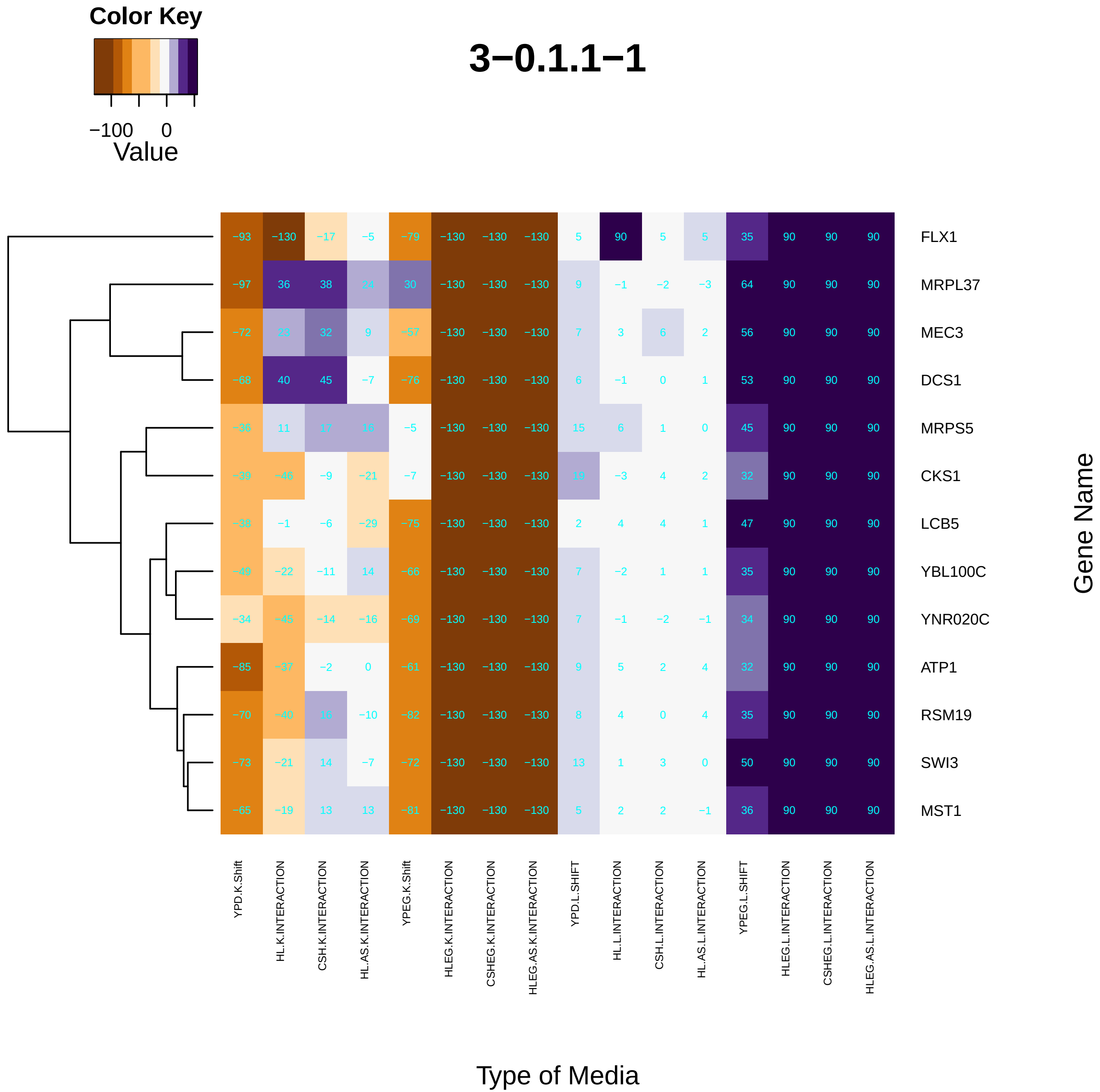
Color Key
−100 0
Value
3−0.1.1−1
FLX1 −93 −130 −17 −5 −79 −130 −130 −130 5 90 5 5 35 90 90 90
MRPL37 −97 36 38 24 30 −130 −130 −130 9 −1 −2 −3 64 90 90 90
MEC3 −72 23 32 9 −57 −130 −130 −130 7 3 6 2 56 90 90 90
DCS1 −68 40 45 −7 −76 −130 −130 −130 6 −1 0 1 53 90 90 90
MRPS5 −36 11 17 16 −5 −130 −130 −130 15 6 1 0 45 90 90 90
CKS1 −39 −46 −9 −21 −7 −130 −130 −130 19 −3 4 2 32 90 90 90
LCB5 −38 −1 −6 −29 −75 −130 −130 −130 2 4 4 1 47 90 90 90
YBL100C −49 −22 −11 14 −66 −130 −130 −130 7 −2 1 1 35 90 90 90
YNR020C −34 −45 −14 −16 −69 −130 −130 −130 7 −1 −2 −1 34 90 90 90
ATP1 −85 −37 −2 0 −61 −130 −130 −130 9 5 2 4 32 90 90 90
RSM19 −70 −40 16 −10 −82 −130 −130 −130 8 4 0 4 35 90 90 90
SWI3 −73 −21 14 −7 −72 −130 −130 −130 13 1 3 0 50 90 90 90
MST1 −65 −19 13 13 −81 −130 −130 −130 5 2 2 −1 36 90 90 90
YPD.K.Shift
HL.K.INTERACTION
CSH.K.INTERACTION
HL.AS.K.INTERACTION
YPEG.K.Shift
HLEG.K.INTERACTION
CSHEG.K.INTERACTION
HLEG.AS.K.INTERACTION
YPD.L.SHIFT
HL.L.INTERACTION
CSH.L.INTERACTION
HL.AS.L.INTERACTION
YPEG.L.SHIFT
HLEG.L.INTERACTION
CSHEG.L.INTERACTION
HLEG.AS.L.INTERACTION
Gene Name
Type of Media

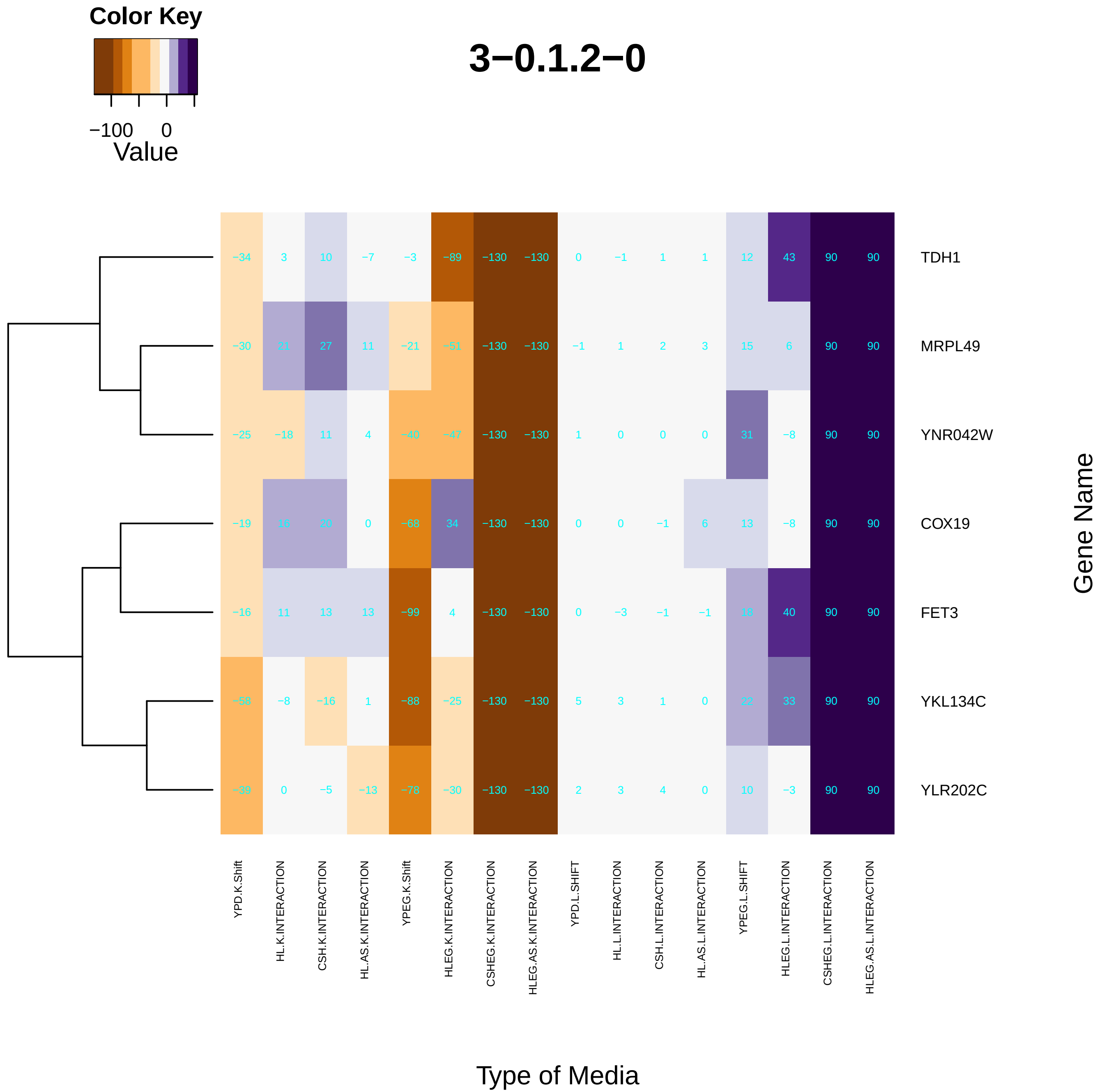

Color Key
−100
0
Value
3−0.1.2−0
TDH1
MRPL49
YNR042W
COX19
FET3
YKL134C
YLR202C
Gene Name
YPD.K.Shift
HL.K.INTERACTION
CSH.K.INTERACTION
HL.AS.K.INTERACTION
YPEG.K.Shift
HLEG.K.INTERACTION
CSHEG.K.INTERACTION
HLEG.AS.K.INTERACTION
YPD.L.SHIFT
HL.L.INTERACTION
CSH.L.INTERACTION
HL.AS.L.INTERACTION
YPEG.L.SHIFT
HLEG.L.INTERACTION
CSHEG.L.INTERACTION
HLEG.AS.L.INTERACTION
Type of Media

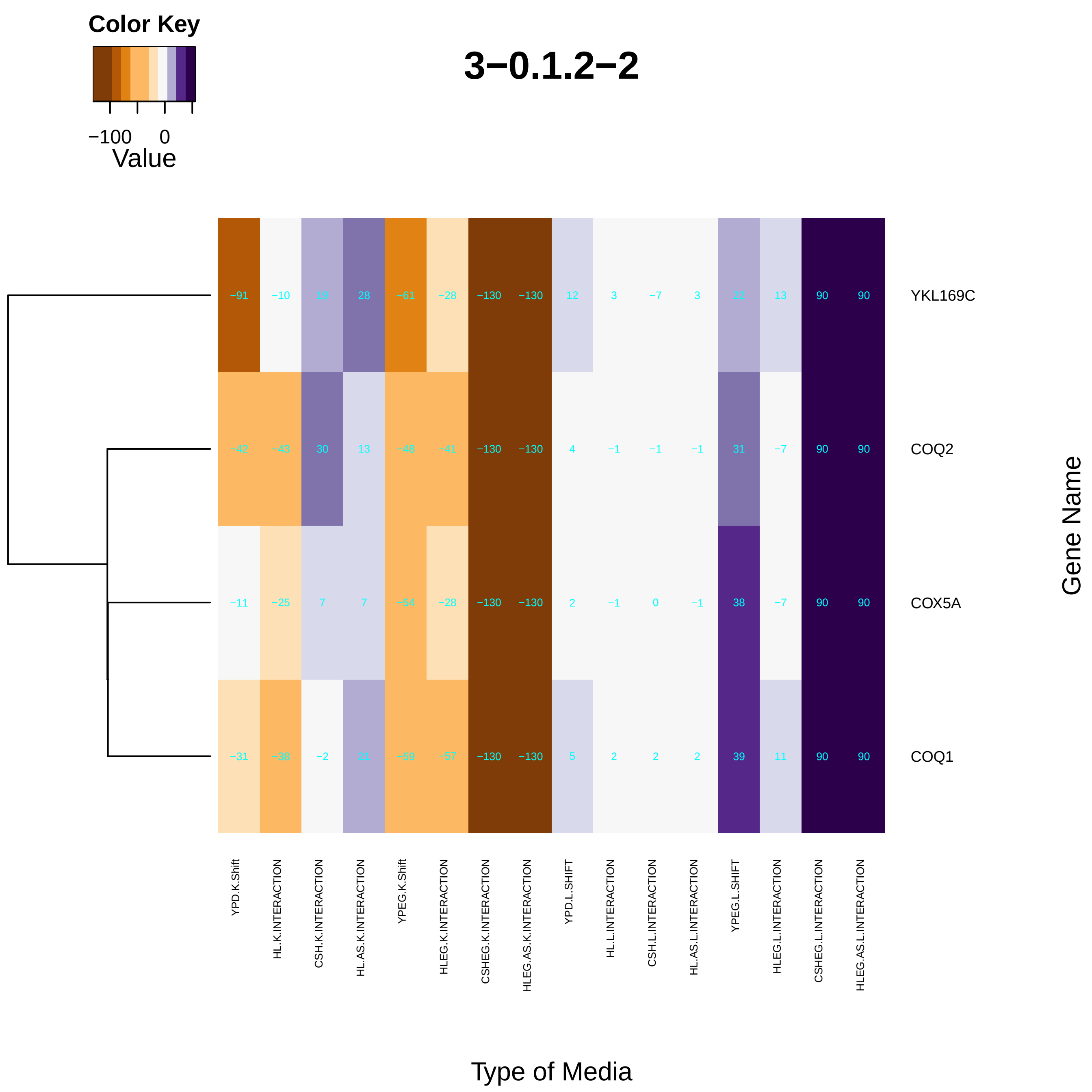

3−0.1.2−2
Color Key
−100
0
Value
YKL169C
COQ2
COX5A
COQ1
Gene Name
Type of Media
YPD.K.Shift
HL.K.INTERACTION
CSH.K.INTERACTION
HL.AS.K.INTERACTION
YPEG.K.Shift
HLEG.K.INTERACTION
CSHEG.K.INTERACTION
HLEG.AS.K.INTERACTION
YPD.L.SHIFT
HL.L.INTERACTION
CSH.L.INTERACTION
HL.AS.L.INTERACTION
YPEG.L.SHIFT
HLEG.L.INTERACTION
CSHEG.L.INTERACTION
HLEG.AS.L.INTERACTION
−91 −10 19 28 −61 −28 −130 −130 12 3 −7 3 22 13 90 90
−42 −43 30 13 −48 −41 −130 −130 4 −1 −1 −1 31 −7 90 90
−11 −25 7 7 −54 −28 −130 −130 2 −1 0 −1 38 −7 90 90
−31 −38 −2 21 −59 −57 −130 −130 5 2 2 2 39 11 90 90

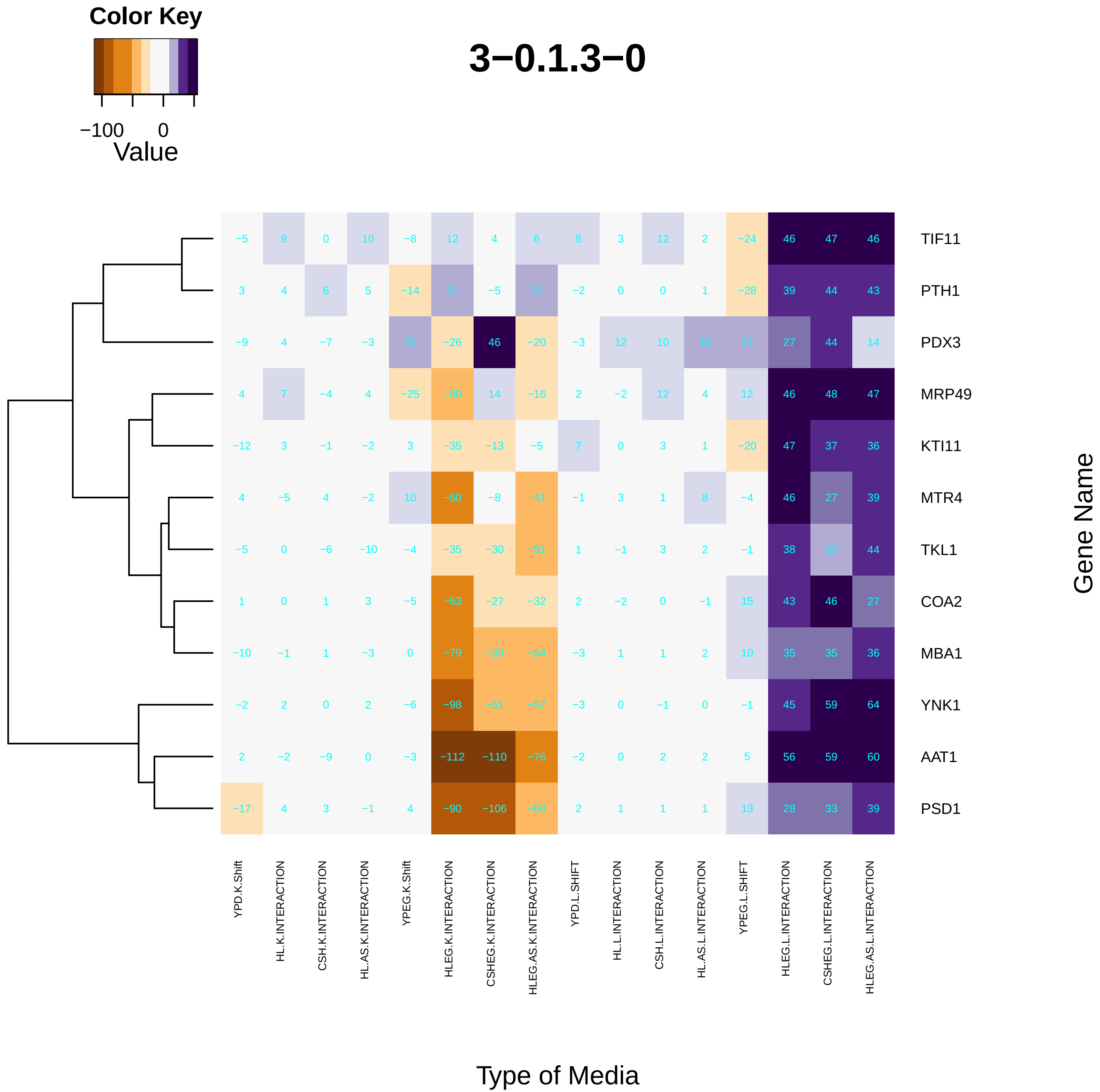
Color Key
−100
0
Value
3−0.1.3−0
TIF11: −5 9 0 10 −8 12 4 6 8 3 12 2 −24 46 47 46
PTH1: 3 4 6 5 −14 20 −5 22 −2 0 0 1 −28 39 44 43
PDX3: −9 4 −7 −3 20 −26 46 −20 −3 12 10 16 17 27 44 14
MRP49: 4 7 −4 4 −25 −50 14 −16 2 −2 12 4 12 46 48 47
KTI11: −12 3 −1 −2 3 −35 −13 −5 7 0 3 1 −20 47 37 36
MTR4: 4 −5 4 −2 10 −60 −8 −41 −1 3 1 8 −4 46 27 39
TKL1: −5 0 −6 −10 −4 −35 −30 −51 1 −1 3 2 −1 38 23 44
COA2: 1 0 1 3 −5 −63 −27 −32 2 −2 0 −1 15 43 46 27
MBA1: −10 −1 1 −3 0 −79 −39 −54 −3 1 1 2 10 35 35 36
YNK1: −2 2 0 2 −6 −98 −51 −57 −3 0 −1 0 −1 45 59 64
AAT1: 2 −2 −9 0 −3 −112 −110 −76 −2 0 2 2 5 56 59 60
PSD1: −17 4 3 −1 4 −90 −106 −60 2 1 1 1 13 28 33 39
YPD.K.Shift
HL.K.INTERACTION
CSH.K.INTERACTION
HL.AS.K.INTERACTION
YPEG.K.Shift
HLEG.K.INTERACTION
CSHEG.K.INTERACTION
HLEG.AS.K.INTERACTION
YPD.L.SHIFT
HL.L.INTERACTION
CSH.L.INTERACTION
HL.AS.L.INTERACTION
YPEG.L.SHIFT
HLEG.L.INTERACTION
CSHEG.L.INTERACTION
HLEG.AS.L.INTERACTION
Gene Name
Type of Media

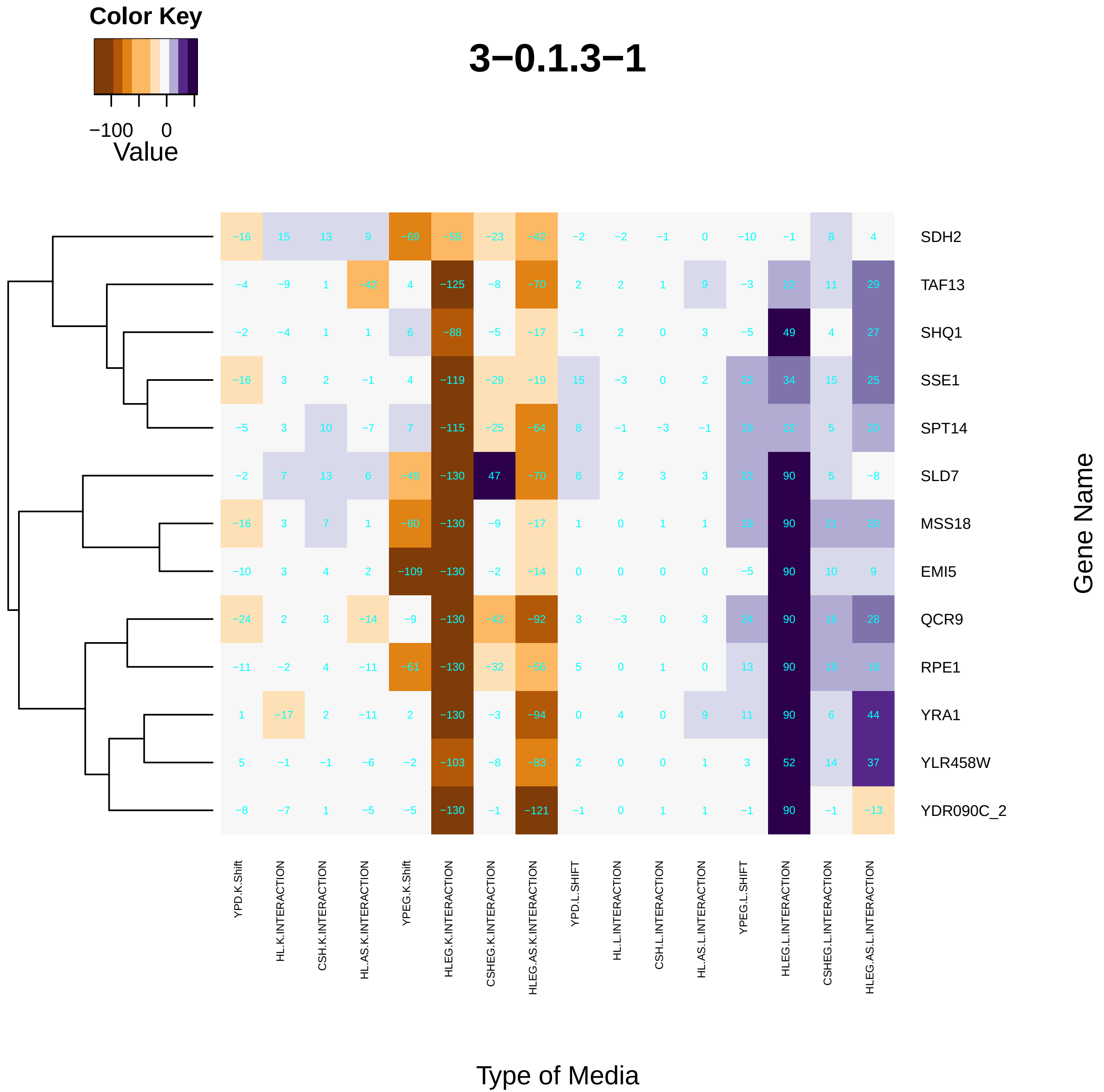
Color Key
−100
0
Value
3−0.1.3−1
−16 15 13 9 −69 −58 −23 −42 −2 −2 −1 0 −10 −1 8 4 SDH2
−4 −9 1 −42 4 −125 −8 −70 2 2 1 9 −3 22 11 29 TAF13
−2 −4 1 1 6 −88 −5 −17 −1 2 0 3 −5 49 4 27 SHQ1
−16 3 2 −1 4 −119 −29 −19 15 −3 0 2 22 34 15 25 SSE1
−5 3 10 −7 7 −115 −25 −64 8 −1 −3 −1 19 22 5 20 SPT14
−2 7 13 6 −49 −130 47 −70 6 2 3 3 22 90 5 −8 SLD7
−16 3 7 1 −80 −130 −9 −17 1 0 1 1 19 90 21 20 MSS18
−10 3 4 2 −109 −130 −2 −14 0 0 0 0 −5 90 10 9 EMI5
−24 2 3 −14 −9 −130 −43 −92 3 −3 0 3 24 90 16 28 QCR9
−11 −2 4 −11 −61 −130 −32 −56 5 0 1 0 13 90 18 18 RPE1
1 −17 2 −11 2 −130 −3 −94 0 4 0 9 11 90 6 44 YRA1
5 −1 −1 −6 −2 −103 −8 −83 2 0 0 1 3 52 14 37 YLR458W
−8 −7 1 −5 −5 −130 −1 −121 −1 0 1 1 −1 90 −1 −13 YDR090C_2
YPD.K.Shift
HL.K.INTERACTION
CSH.K.INTERACTION
HL.AS.K.INTERACTION
YPEG.K.Shift
HLEG.K.INTERACTION
CSHEG.K.INTERACTION
HLEG.AS.K.INTERACTION
YPD.L.SHIFT
HL.L.INTERACTION
CSH.L.INTERACTION
HL.AS.L.INTERACTION
YPEG.L.SHIFT
HLEG.L.INTERACTION
CSHEG.L.INTERACTION
HLEG.AS.L.INTERACTION
Gene Name
Type of Media

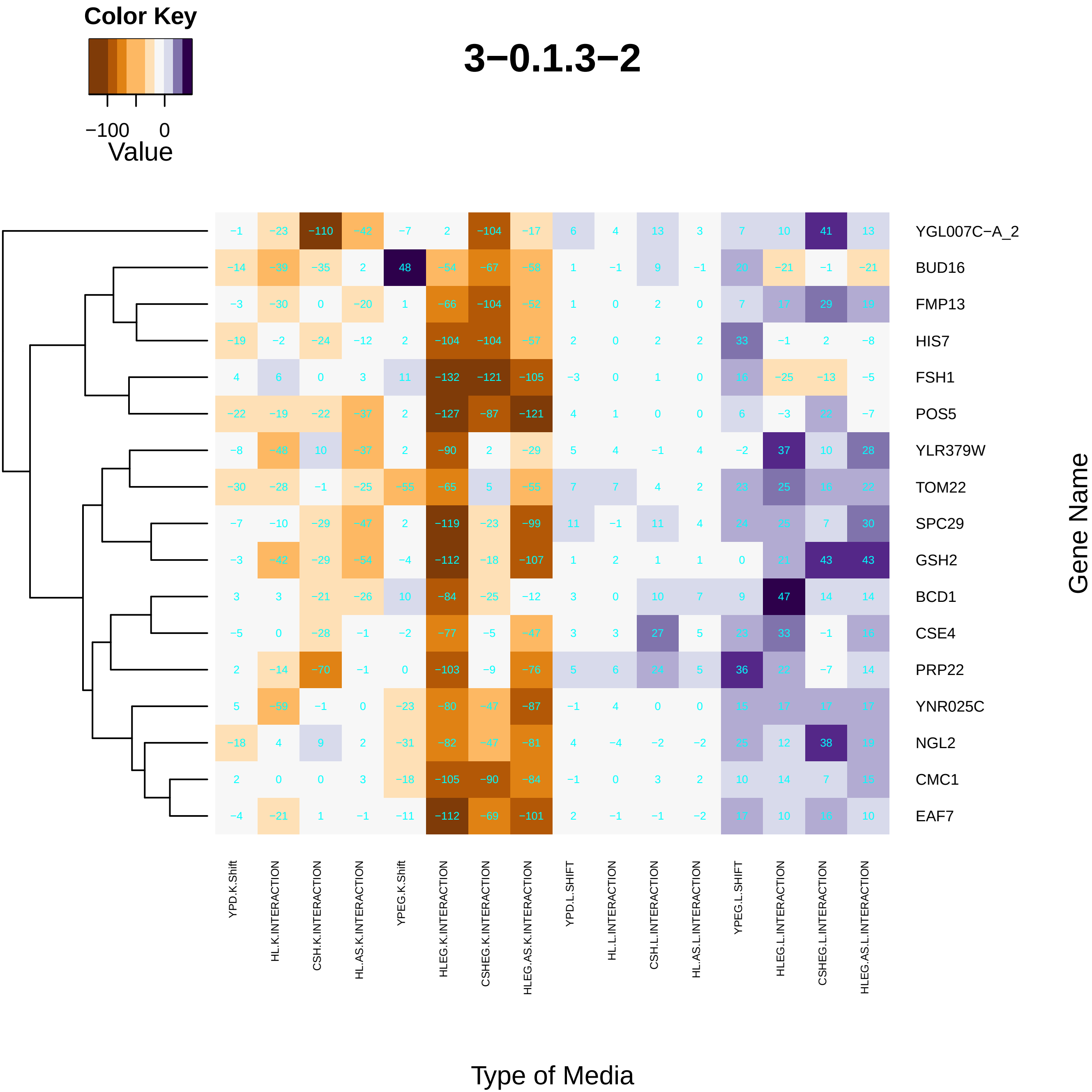

Color Key
−100
0
Value
3−0.1.3−2
YGL007C−A_2
BUD16
FMP13
HIS7
FSH1
POS5
YLR379W
TOM22
SPC29
GSH2
BCD1
CSE4
PRP22
YNR025C
NGL2
CMC1
EAF7
Gene Name
YPD.K.Shift
HL.K.INTERACTION
CSH.K.INTERACTION
HL.AS.K.INTERACTION
YPEG.K.Shift
HLEG.K.INTERACTION
CSHEG.K.INTERACTION
HLEG.AS.K.INTERACTION
YPD.L.SHIFT
HL.L.INTERACTION
CSH.L.INTERACTION
HL.AS.L.INTERACTION
YPEG.L.SHIFT
HLEG.L.INTERACTION
CSHEG.L.INTERACTION
HLEG.AS.L.INTERACTION
Type of Media

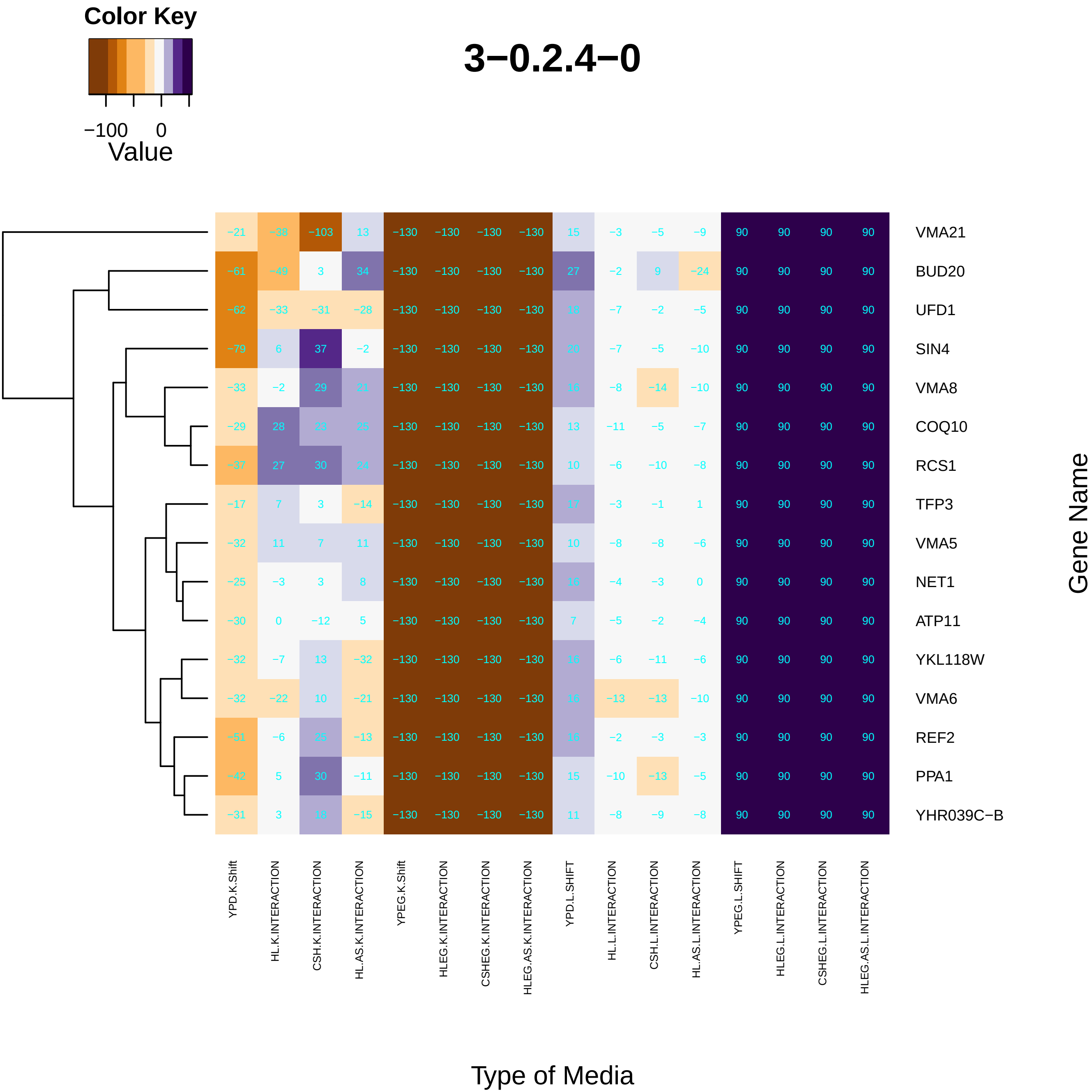
3−0.2.4−0
Color Key
−100
0
Value
VMA21
BUD20
UFD1
SIN4
VMA8
COQ10
RCS1
TFP3
VMA5
NET1
ATP11
YKL118W
VMA6
REF2
PPA1
YHR039C−B
Gene Name
YPD.K.Shift
HL.K.INTERACTION
CSH.K.INTERACTION
HL.AS.K.INTERACTION
YPEG.K.Shift
HLEG.K.INTERACTION
CSHEG.K.INTERACTION
HLEG.AS.K.INTERACTION
YPD.L.SHIFT
HL.L.INTERACTION
CSH.L.INTERACTION
HL.AS.L.INTERACTION
YPEG.L.SHIFT
HLEG.L.INTERACTION
CSHEG.L.INTERACTION
HLEG.AS.L.INTERACTION
Type of Media

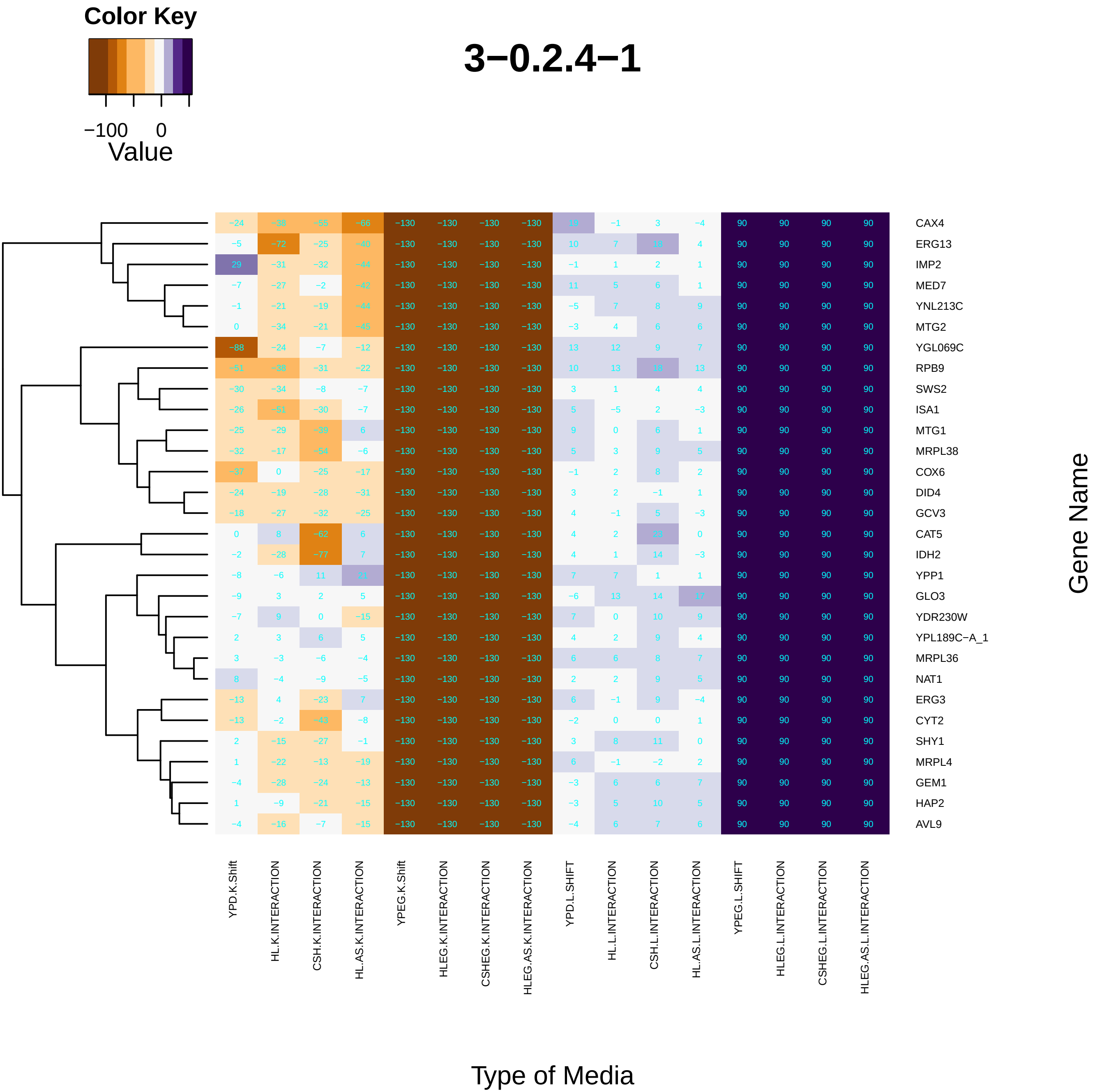

3−0.2.4−1
Color Key
−100
0
Value
CAX4
ERG13
IMP2
MED7
YNL213C
MTG2
YGL069C
RPB9
SWS2
ISA1
MTG1
MRPL38
COX6
DID4
GCV3
CAT5
IDH2
YPP1
GLO3
YDR230W
YPL189C−A_1
MRPL36
NAT1
ERG3
CYT2
SHY1
MRPL4
GEM1
HAP2
AVL9
YPD.K.Shift
HL.K.INTERACTION
CSH.K.INTERACTION
HL.AS.K.INTERACTION
YPEG.K.Shift
HLEG.K.INTERACTION
CSHEG.K.INTERACTION
HLEG.AS.K.INTERACTION
YPD.L.SHIFT
HL.L.INTERACTION
CSH.L.INTERACTION
HL.AS.L.INTERACTION
YPEG.L.SHIFT
HLEG.L.INTERACTION
CSHEG.L.INTERACTION
HLEG.AS.L.INTERACTION
Gene Name
Type of Media

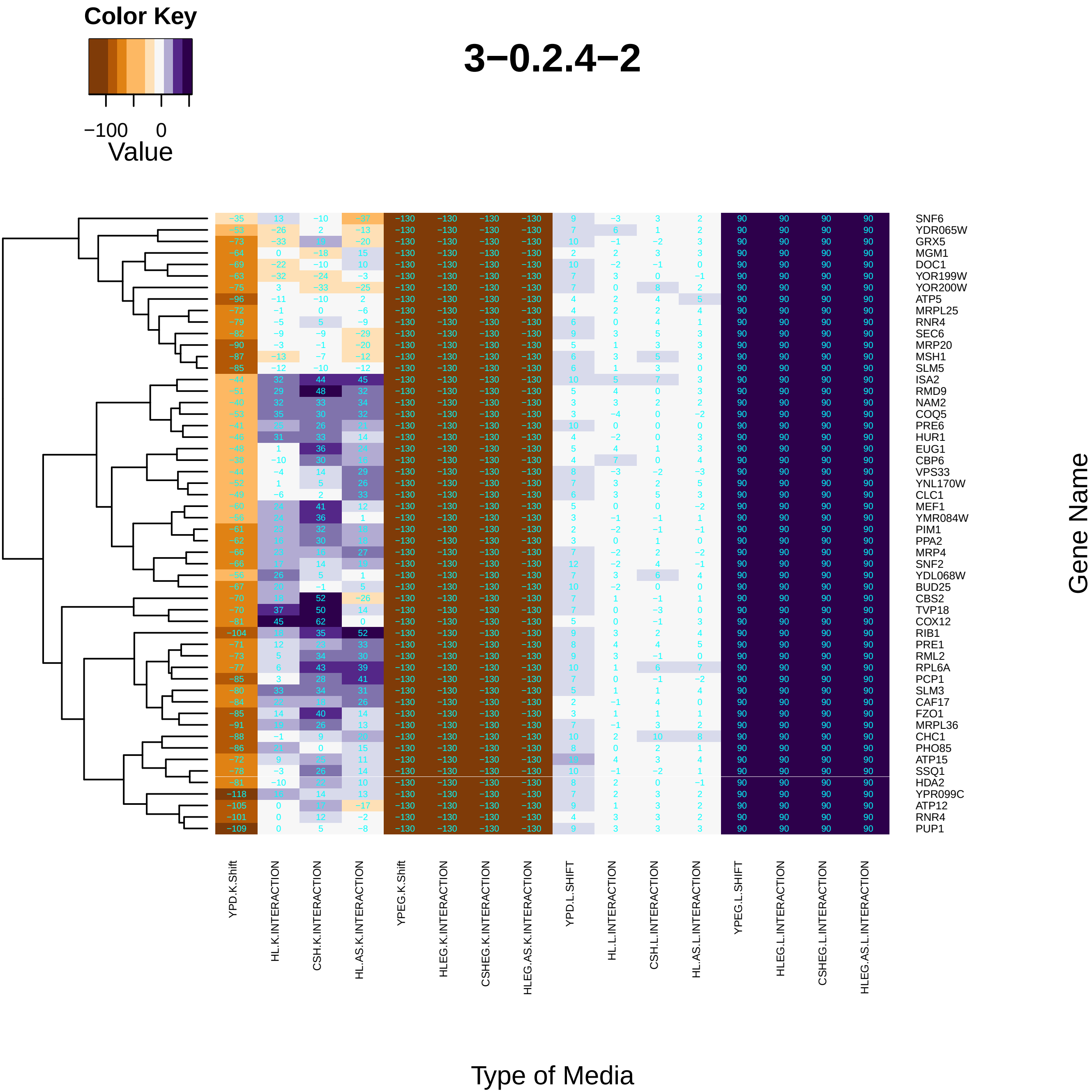

Color Key
−100
0
Value
3−0.2.4−2
SNF6
YDR065W
GRX5
MGM1
DOC1
YOR199W
YOR200W
ATP5
MRPL25
RNR4
SEC6
MRP20
MSH1
SLM5
ISA2
RMD9
NAM2
COQ5
PRE6
HUR1
EUG1
CBP6
VPS33
YNL170W
CLC1
MEF1
YMR084W
PIM1
PPA2
MRP4
SNF2
YDL068W
BUD25
CBS2
TVP18
COX12
RIB1
PRE1
RML2
RPL6A
PCP1
SLM3
CAF17
FZO1
MRPL36
CHC1
PHO85
ATP15
SSQ1
HDA2
YPR099C
ATP12
RNR4
PUP1
Gene Name
YPD.K.Shift
HL.K.INTERACTION
CSH.K.INTERACTION
HL.AS.K.INTERACTION
YPEG.K.Shift
HLEG.K.INTERACTION
CSHEG.K.INTERACTION
HLEG.AS.K.INTERACTION
YPD.L.SHIFT
HL.L.INTERACTION
CSH.L.INTERACTION
HL.AS.L.INTERACTION
YPEG.L.SHIFT
HLEG.L.INTERACTION
CSHEG.L.INTERACTION
HLEG.AS.L.INTERACTION
Type of Media

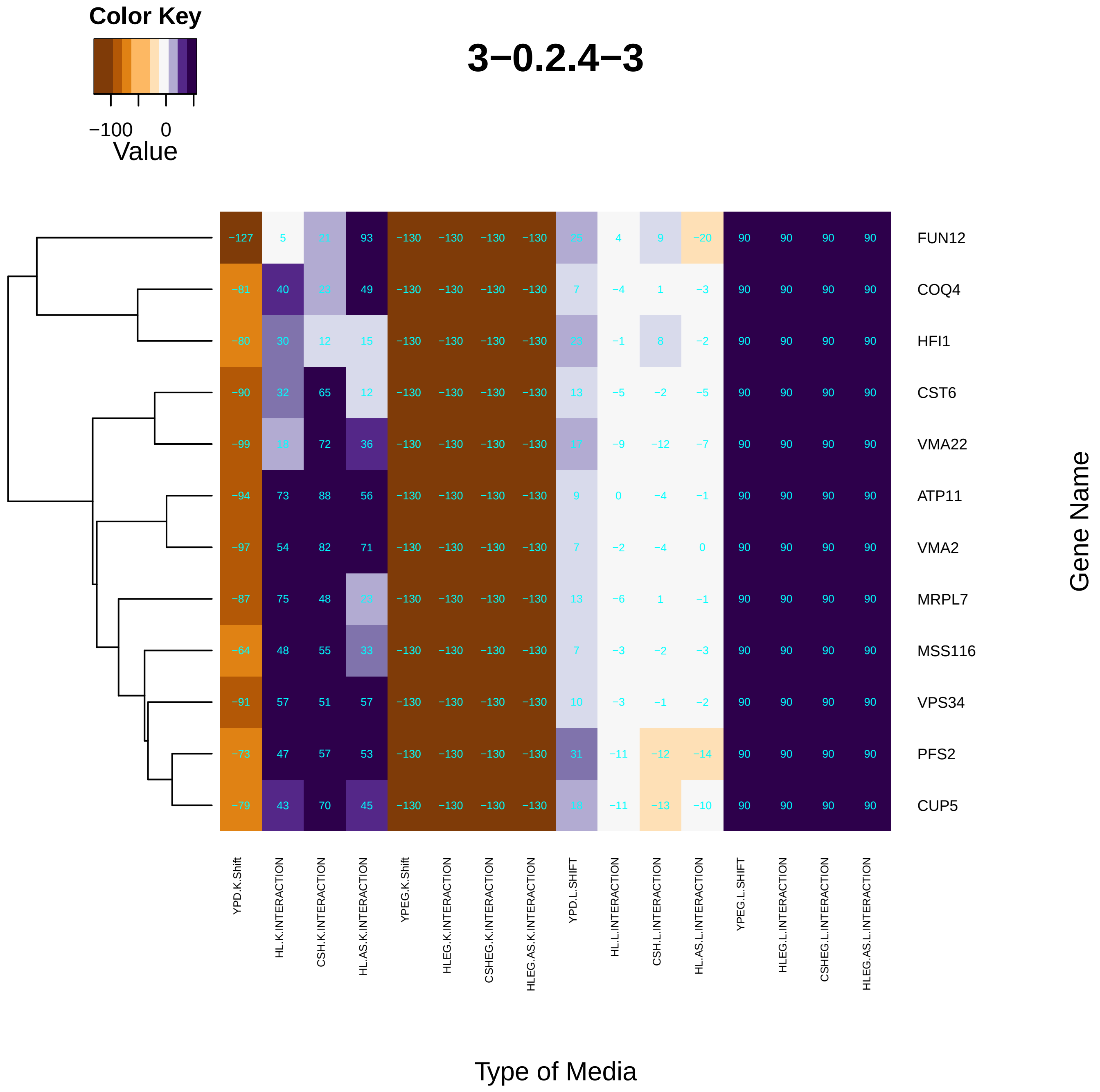
Color Key
−100
0
Value
3−0.2.4−3
YPD.K.Shift
HL.K.INTERACTION
CSH.K.INTERACTION
HL.AS.K.INTERACTION
YPEG.K.Shift
HLEG.K.INTERACTION
CSHEG.K.INTERACTION
HLEG.AS.K.INTERACTION
YPD.L.SHIFT
HL.L.INTERACTION
CSH.L.INTERACTION
HL.AS.L.INTERACTION
YPEG.L.SHIFT
HLEG.L.INTERACTION
CSHEG.L.INTERACTION
HLEG.AS.L.INTERACTION
FUN12 −127 5 21 93 −130 −130 −130 −130 25 4 9 −20 90 90 90 90
COQ4 −81 40 23 49 −130 −130 −130 −130 7 −4 1 −3 90 90 90 90
HFI1 −80 30 12 15 −130 −130 −130 −130 23 −1 8 −2 90 90 90 90
CST6 −90 32 65 12 −130 −130 −130 −130 13 −5 −2 −5 90 90 90 90
VMA22 −99 18 72 36 −130 −130 −130 −130 17 −9 −12 −7 90 90 90 90
ATP11 −94 73 88 56 −130 −130 −130 −130 9 0 −4 −1 90 90 90 90
VMA2 −97 54 82 71 −130 −130 −130 −130 7 −2 −4 0 90 90 90 90
MRPL7 −87 75 48 23 −130 −130 −130 −130 13 −6 1 −1 90 90 90 90
MSS116 −64 48 55 33 −130 −130 −130 −130 7 −3 −2 −3 90 90 90 90
VPS34 −91 57 51 57 −130 −130 −130 −130 10 −3 −1 −2 90 90 90 90
PFS2 −73 47 57 53 −130 −130 −130 −130 31 −11 −12 −14 90 90 90 90
CUP5 −79 43 70 45 −130 −130 −130 −130 18 −11 −13 −10 90 90 90 90
Gene Name
Type of Media

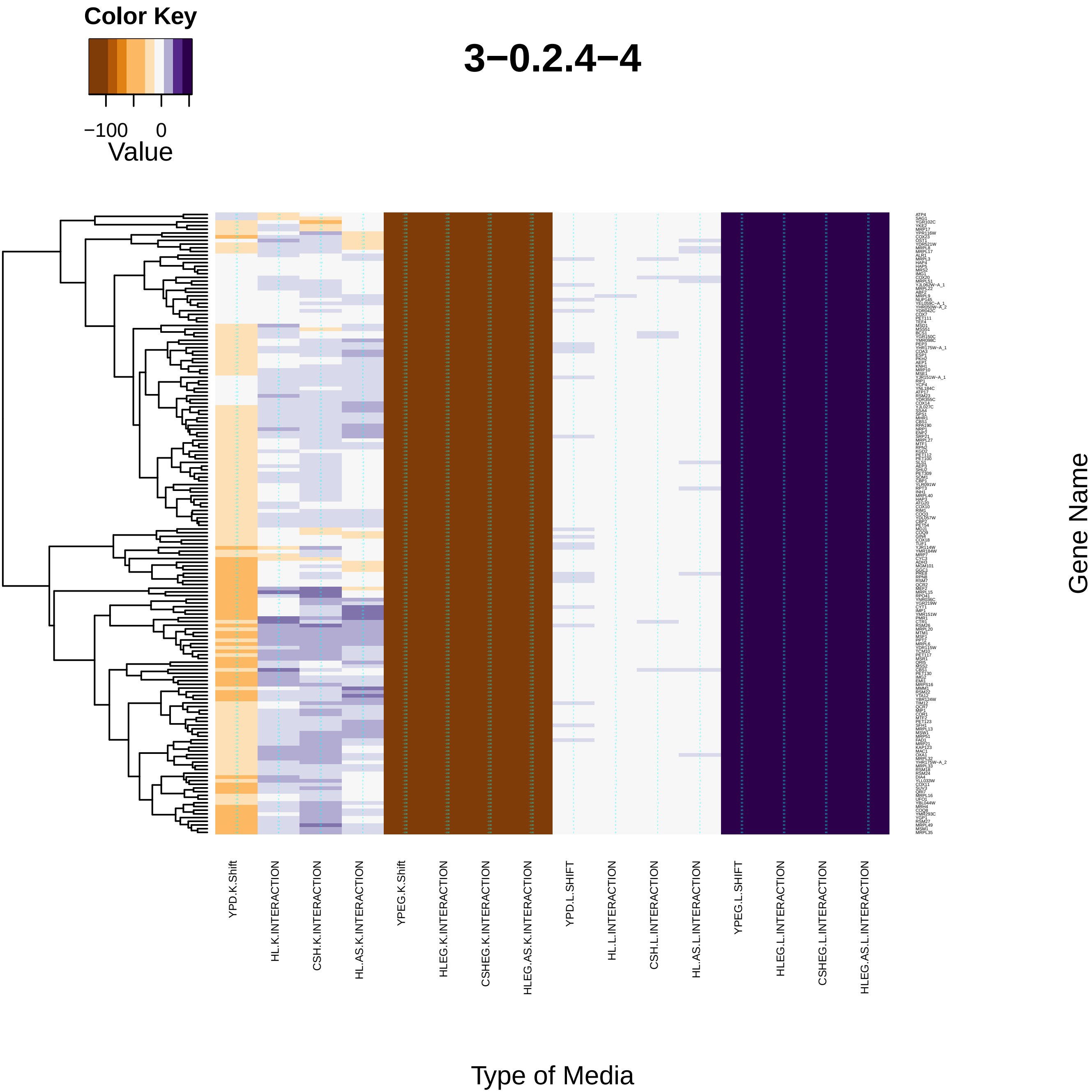
Color Key
−100
0
Value
3−0.2.4−4
YPD.K.Shift
HL.K.INTERACTION
CSH.K.INTERACTION
HL.AS.K.INTERACTION
YPEG.K.Shift
HLEG.K.INTERACTION
CSHEG.K.INTERACTION
HLEG.AS.K.INTERACTION
YPD.L.SHIFT
HL.L.INTERACTION
CSH.L.INTERACTION
HL.AS.L.INTERACTION
YPEG.L.SHIFT
HLEG.L.INTERACTION
CSHEG.L.INTERACTION
HLEG.AS.L.INTERACTION
Type of Media
Gene Name

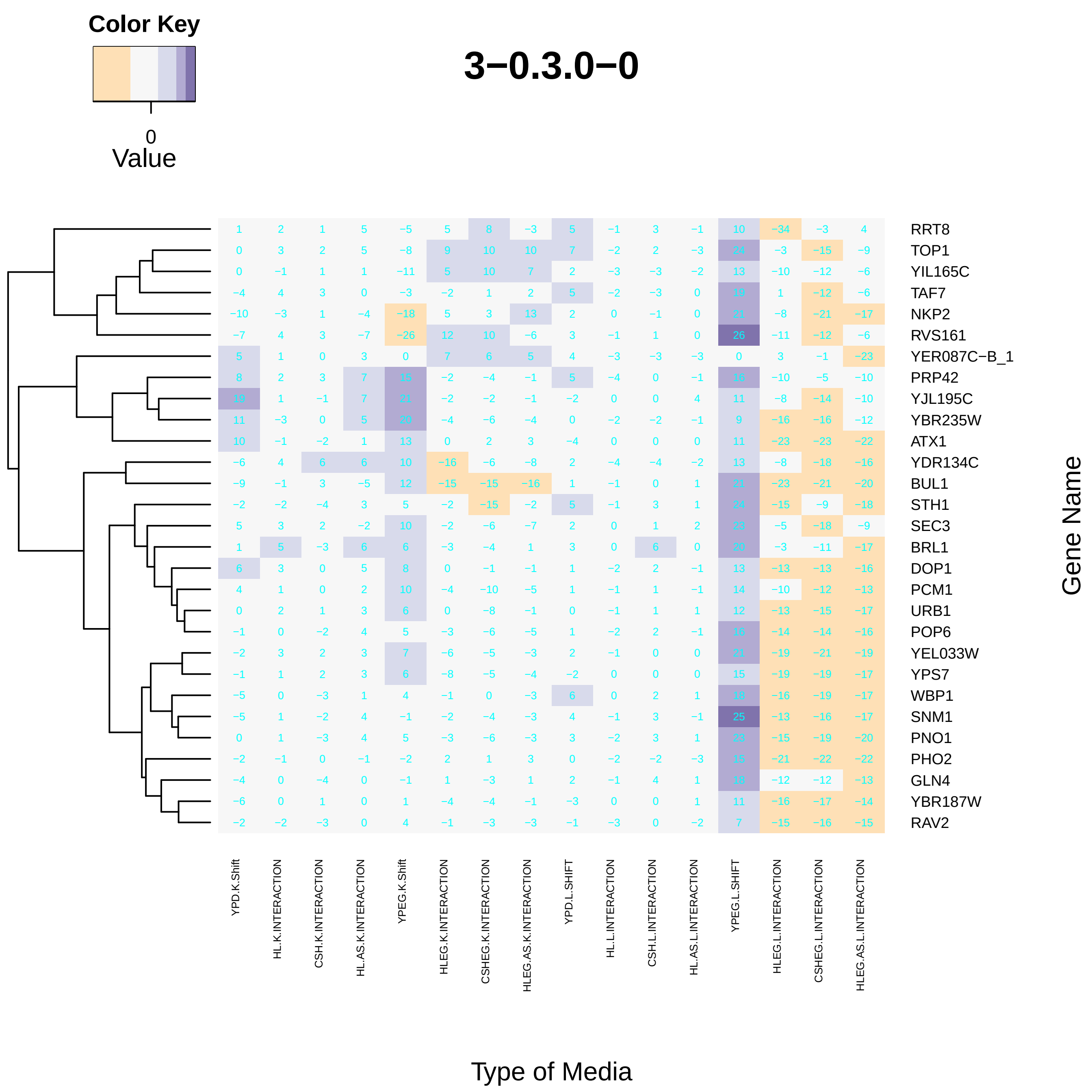
Color Key
0
Value
3−0.3.0−0
RRT8
TOP1
YIL165C
TAF7
NKP2
RVS161
YER087C−B_1
PRP42
YJL195C
YBR235W
ATX1
YDR134C
BUL1
STH1
SEC3
BRL1
DOP1
PCM1
URB1
POP6
YEL033W
YPS7
WBP1
SNM1
PNO1
PHO2
GLN4
YBR187W
RAV2
Gene Name
YPD.K.Shift
HL.K.INTERACTION
CSH.K.INTERACTION
HL.AS.K.INTERACTION
YPEG.K.Shift
HLEG.K.INTERACTION
CSHEG.K.INTERACTION
HLEG.AS.K.INTERACTION
YPD.L.SHIFT
HL.L.INTERACTION
CSH.L.INTERACTION
HL.AS.L.INTERACTION
YPEG.L.SHIFT
HLEG.L.INTERACTION
CSHEG.L.INTERACTION
HLEG.AS.L.INTERACTION
Type of Media

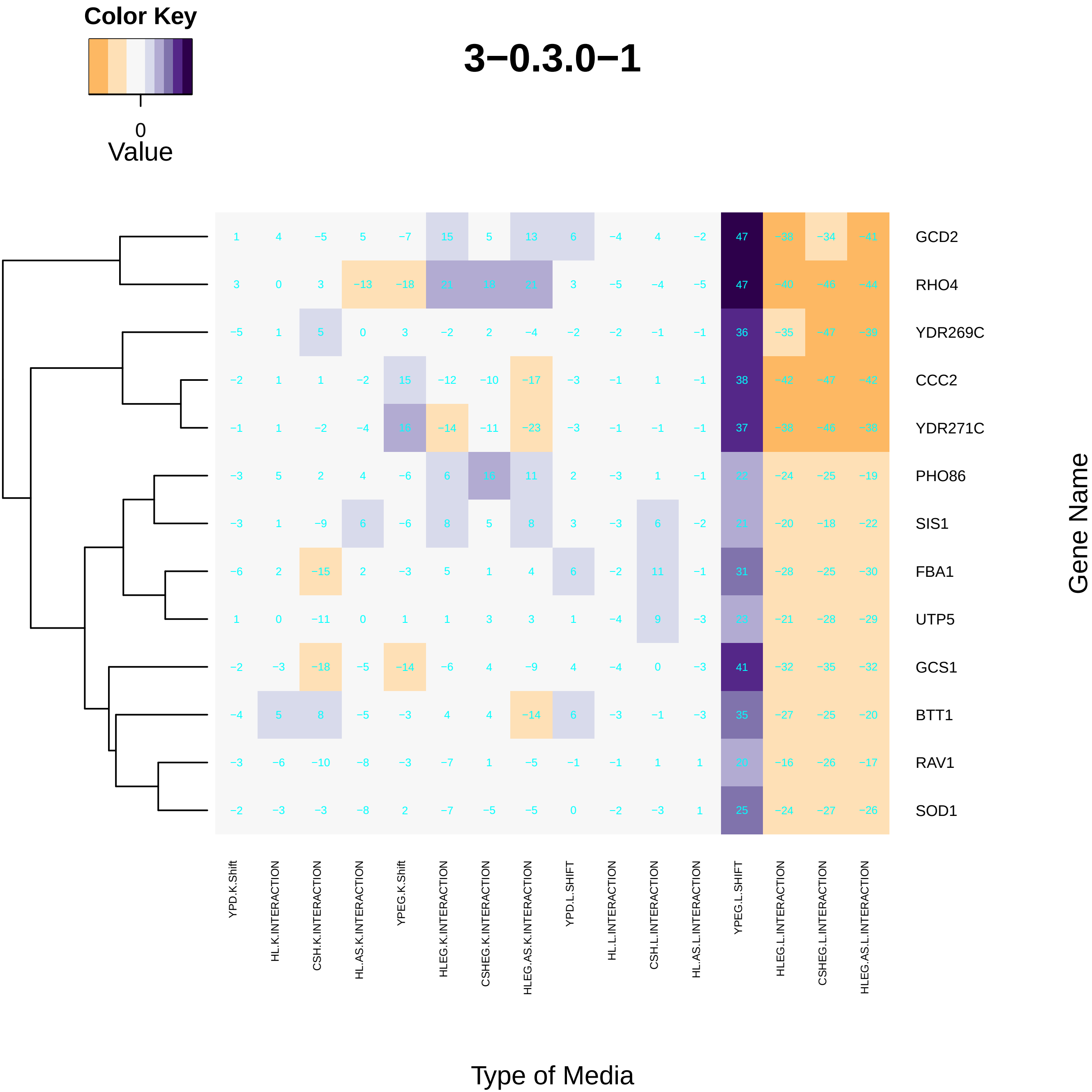
Color Key
0
Value
3−0.3.0−1
GCD2: 1 4 −5 5 −7 15 5 13 6 −4 4 −2 47 −38 −34 −41
RHO4: 3 0 3 −13 −18 21 18 21 3 −5 −4 −5 47 −40 −46 −44
YDR269C: −5 1 5 0 3 −2 2 −4 −2 −2 −1 −1 36 −35 −47 −39
CCC2: −2 1 1 −2 15 −12 −10 −17 −3 −1 1 −1 38 −42 −47 −42
YDR271C: −1 1 −2 −4 16 −14 −11 −23 −3 −1 −1 −1 37 −38 −46 −38
PHO86: −3 5 2 4 −6 6 16 11 2 −3 1 −1 22 −24 −25 −19
SIS1: −3 1 −9 6 −6 8 5 8 3 −3 6 −2 21 −20 −18 −22
FBA1: −6 2 −15 2 −3 5 1 4 6 −2 11 −1 31 −28 −25 −30
UTP5: 1 0 −11 0 1 1 3 3 1 −4 9 −3 23 −21 −28 −29
GCS1: −2 −3 −18 −5 −14 −6 4 −9 4 −4 0 −3 41 −32 −35 −32
BTT1: −4 5 8 −5 −3 4 4 −14 6 −3 −1 −3 35 −27 −25 −20
RAV1: −3 −6 −10 −8 −3 −7 1 −5 −1 −1 1 1 20 −16 −26 −17
SOD1: −2 −3 −3 −8 2 −7 −5 −5 0 −2 −3 1 25 −24 −27 −26
YPD.K.Shift
HL.K.INTERACTION
CSH.K.INTERACTION
HL.AS.K.INTERACTION
YPEG.K.Shift
HLEG.K.INTERACTION
CSHEG.K.INTERACTION
HLEG.AS.K.INTERACTION
YPD.L.SHIFT
HL.L.INTERACTION
CSH.L.INTERACTION
HL.AS.L.INTERACTION
YPEG.L.SHIFT
HLEG.L.INTERACTION
CSHEG.L.INTERACTION
HLEG.AS.L.INTERACTION
Gene Name
Type of Media

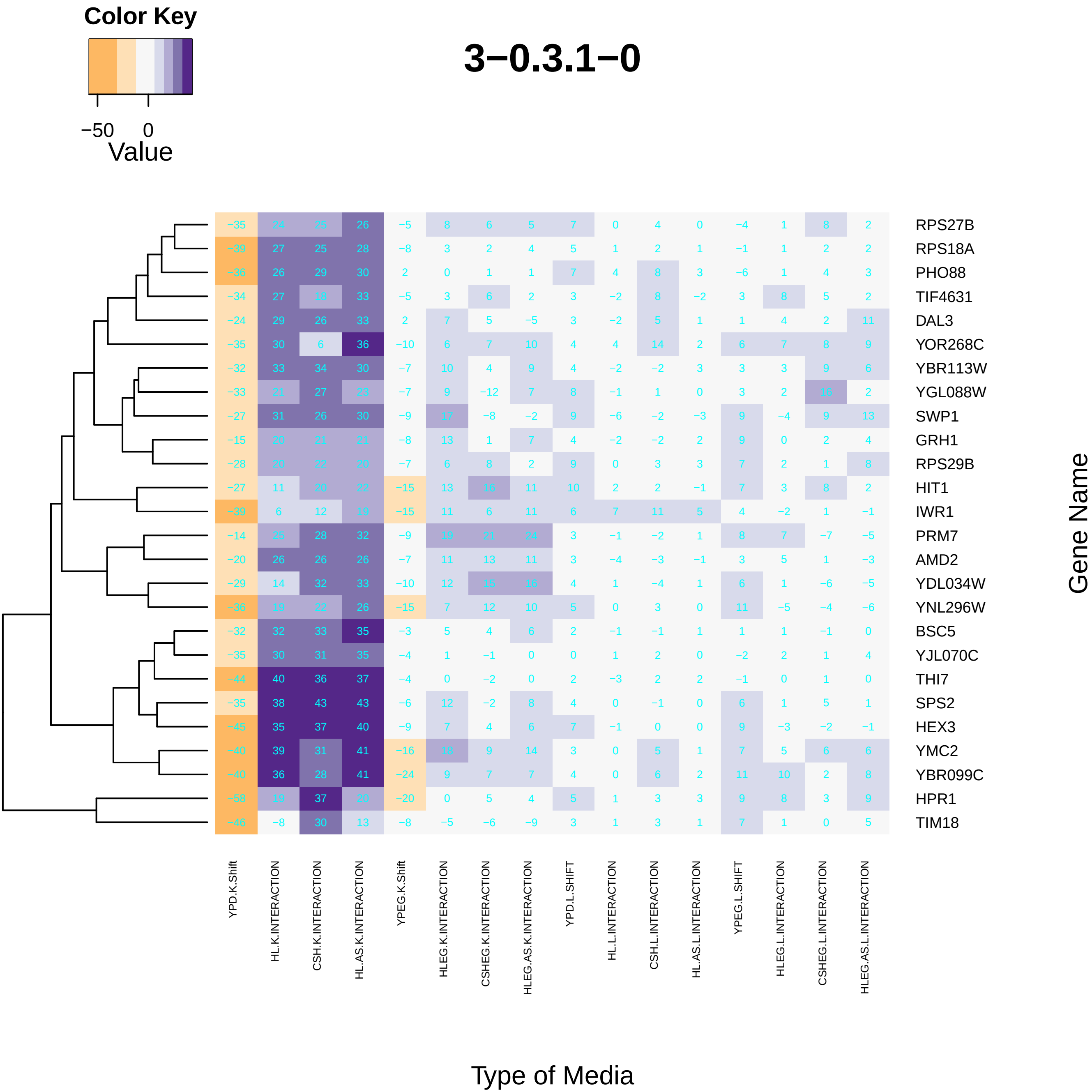
Color Key
−50
0
Value
3−0.3.1−0
RPS27B
RPS18A
PHO88
TIF4631
DAL3
YOR268C
YBR113W
YGL088W
SWP1
GRH1
RPS29B
HIT1
IWR1
PRM7
AMD2
YDL034W
YNL296W
BSC5
YJL070C
THI7
SPS2
HEX3
YMC2
YBR099C
HPR1
TIM18
Gene Name
YPD.K.Shift
HL.K.INTERACTION
CSH.K.INTERACTION
HL.AS.K.INTERACTION
YPEG.K.Shift
HLEG.K.INTERACTION
CSHEG.K.INTERACTION
HLEG.AS.K.INTERACTION
YPD.L.SHIFT
HL.L.INTERACTION
CSH.L.INTERACTION
HL.AS.L.INTERACTION
YPEG.L.SHIFT
HLEG.L.INTERACTION
CSHEG.L.INTERACTION
HLEG.AS.L.INTERACTION
Type of Media

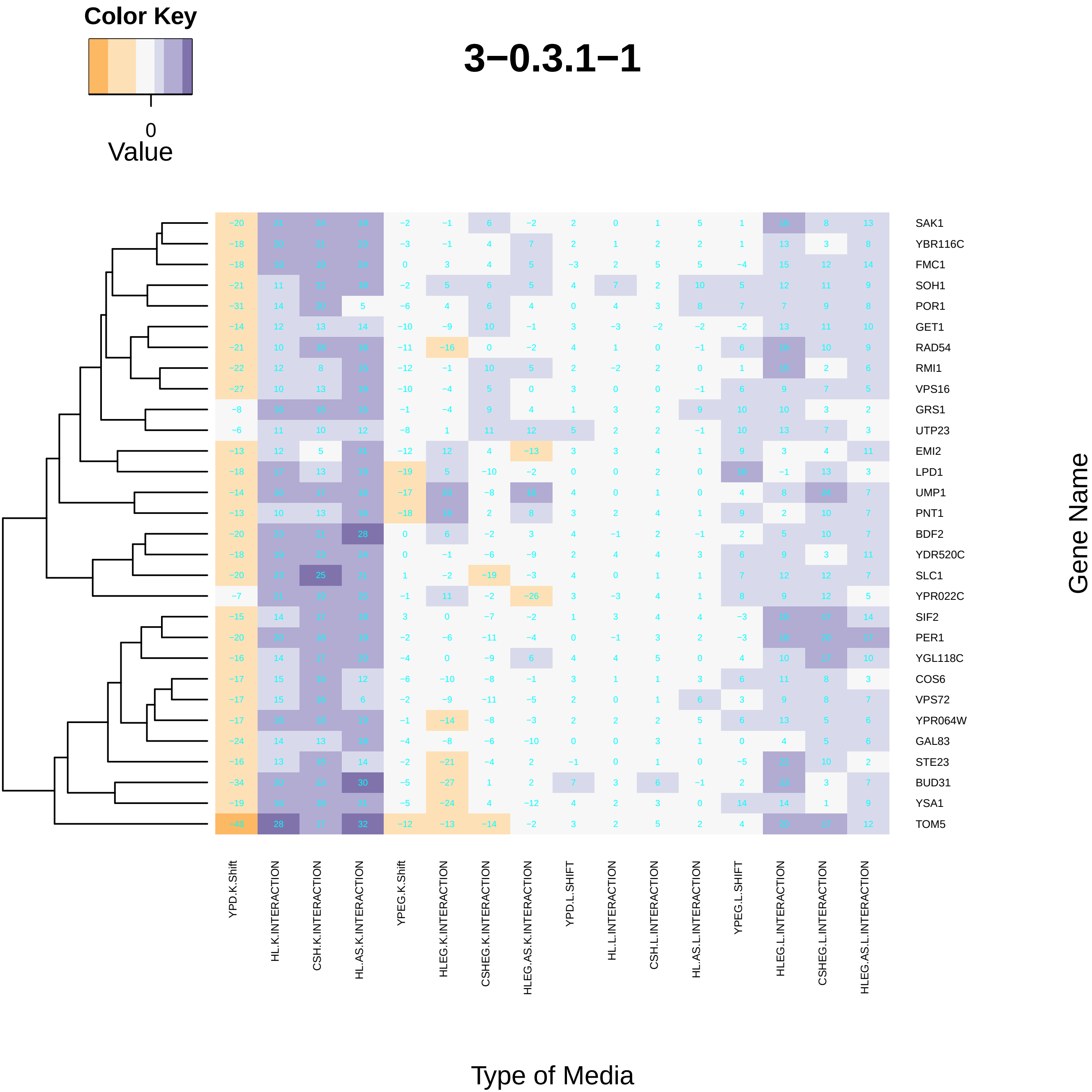
Color Key
0
Value
3−0.3.1−1
SAK1
YBR116C
FMC1
SOH1
POR1
GET1
RAD54
RMI1
VPS16
GRS1
UTP23
EMI2
LPD1
UMP1
PNT1
BDF2
YDR520C
SLC1
YPR022C
SIF2
PER1
YGL118C
COS6
VPS72
YPR064W
GAL83
STE23
BUD31
YSA1
TOM5
Gene Name
YPD.K.Shift
HL.K.INTERACTION
CSH.K.INTERACTION
HL.AS.K.INTERACTION
YPEG.K.Shift
HLEG.K.INTERACTION
CSHEG.K.INTERACTION
HLEG.AS.K.INTERACTION
YPD.L.SHIFT
HL.L.INTERACTION
CSH.L.INTERACTION
HL.AS.L.INTERACTION
YPEG.L.SHIFT
HLEG.L.INTERACTION
CSHEG.L.INTERACTION
HLEG.AS.L.INTERACTION
Type of Media

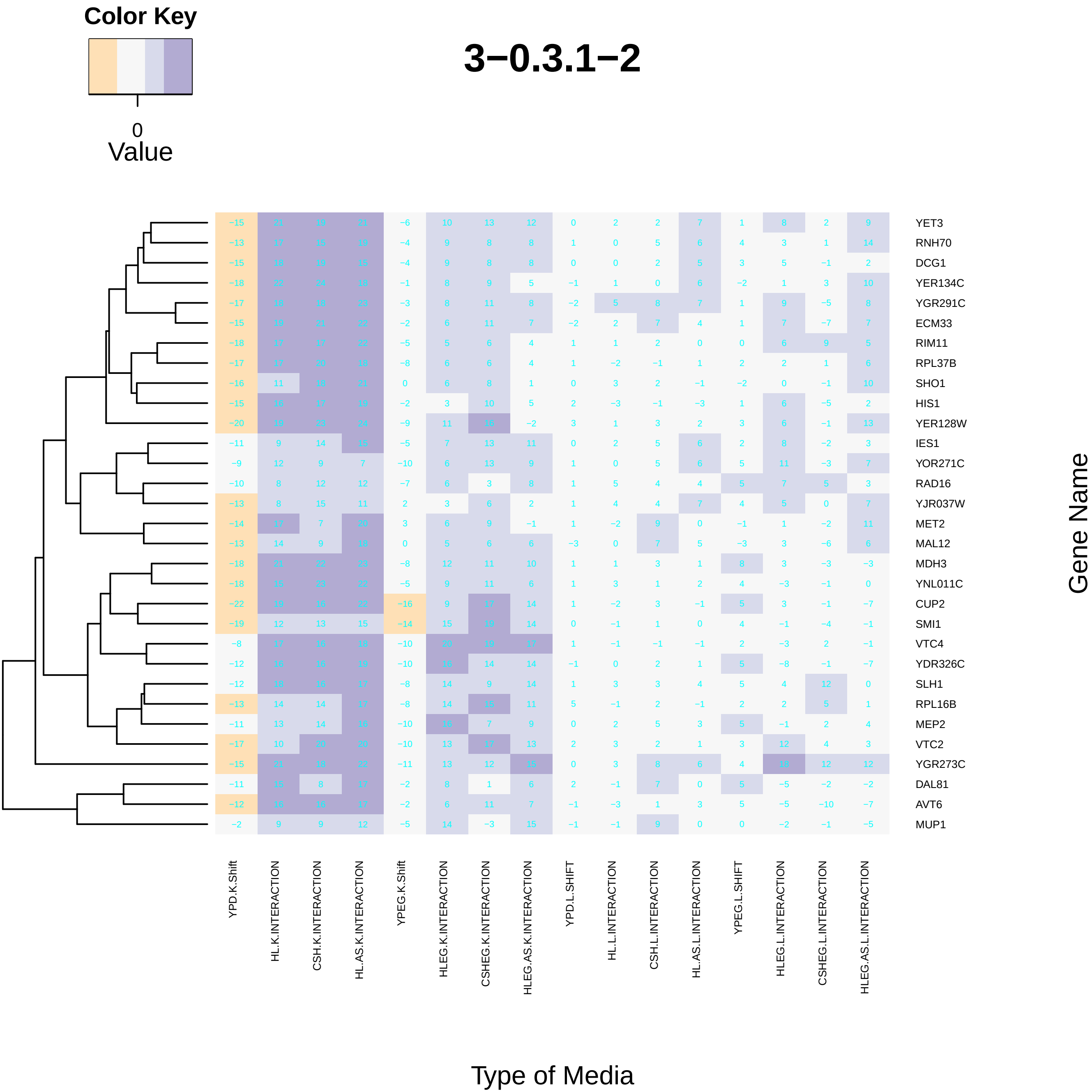

Color Key
0
Value
3−0.3.1−2
YET3
RNH70
DCG1
YER134C
YGR291C
ECM33
RIM11
RPL37B
SHO1
HIS1
YER128W
IES1
YOR271C
RAD16
YJR037W
MET2
MAL12
MDH3
YNL011C
CUP2
SMI1
VTC4
YDR326C
SLH1
RPL16B
MEP2
VTC2
YGR273C
DAL81
AVT6
MUP1
Gene Name
YPD.K.Shift
HL.K.INTERACTION
CSH.K.INTERACTION
HL.AS.K.INTERACTION
YPEG.K.Shift
HLEG.K.INTERACTION
CSHEG.K.INTERACTION
HLEG.AS.K.INTERACTION
YPD.L.SHIFT
HL.L.INTERACTION
CSH.L.INTERACTION
HL.AS.L.INTERACTION
YPEG.L.SHIFT
HLEG.L.INTERACTION
CSHEG.L.INTERACTION
HLEG.AS.L.INTERACTION
Type of Media

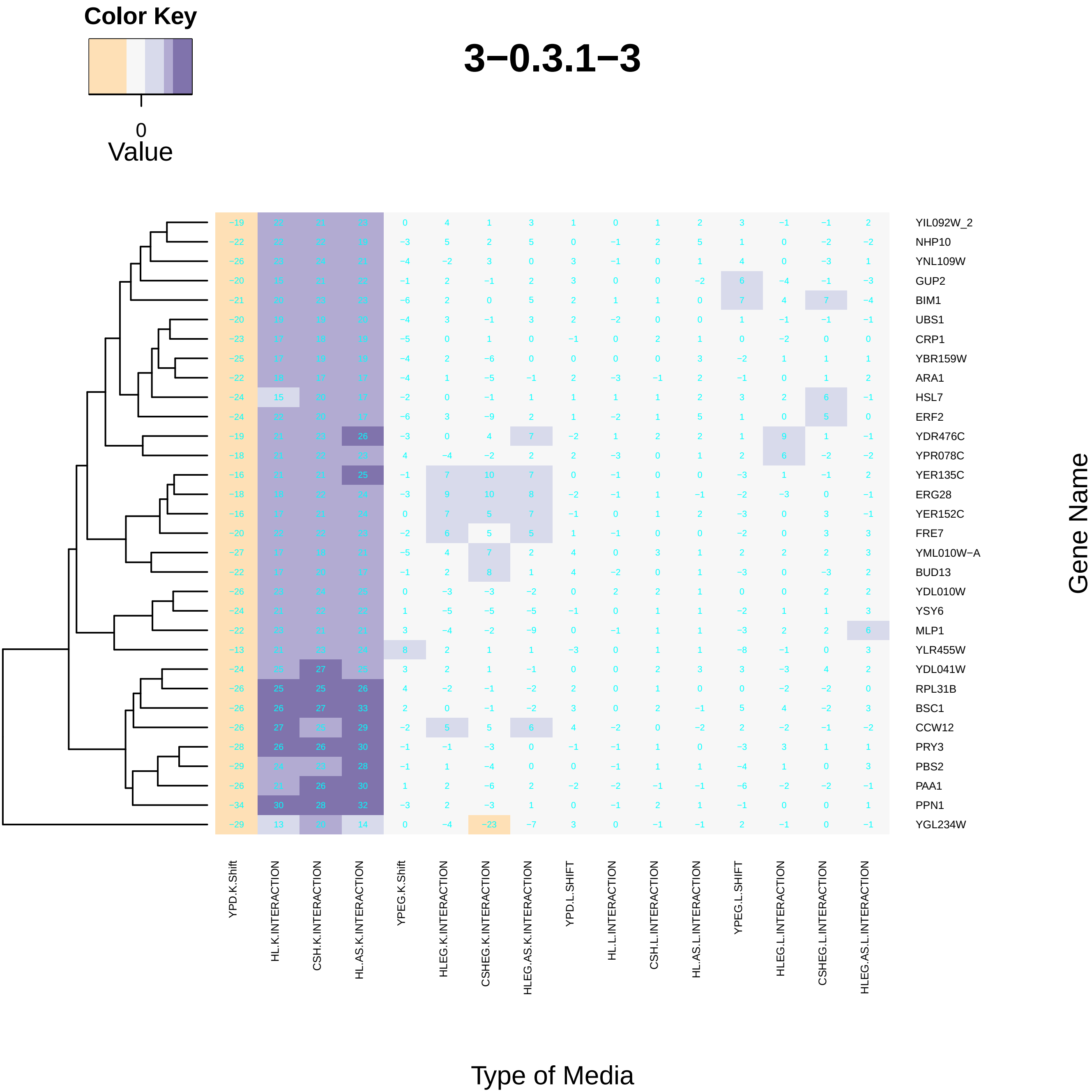
Color Key
0
Value
3−0.3.1−3
YIL092W_2
NHP10
YNL109W
GUP2
BIM1
UBS1
CRP1
YBR159W
ARA1
HSL7
ERF2
YDR476C
YPR078C
YER135C
ERG28
YER152C
FRE7
YML010W−A
BUD13
YDL010W
YSY6
MLP1
YLR455W
YDL041W
RPL31B
BSC1
CCW12
PRY3
PBS2
PAA1
PPN1
YGL234W
Gene Name
YPD.K.Shift
HL.K.INTERACTION
CSH.K.INTERACTION
HL.AS.K.INTERACTION
YPEG.K.Shift
HLEG.K.INTERACTION
CSHEG.K.INTERACTION
HLEG.AS.K.INTERACTION
YPD.L.SHIFT
HL.L.INTERACTION
CSH.L.INTERACTION
HL.AS.L.INTERACTION
YPEG.L.SHIFT
HLEG.L.INTERACTION
CSHEG.L.INTERACTION
HLEG.AS.L.INTERACTION
Type of Media

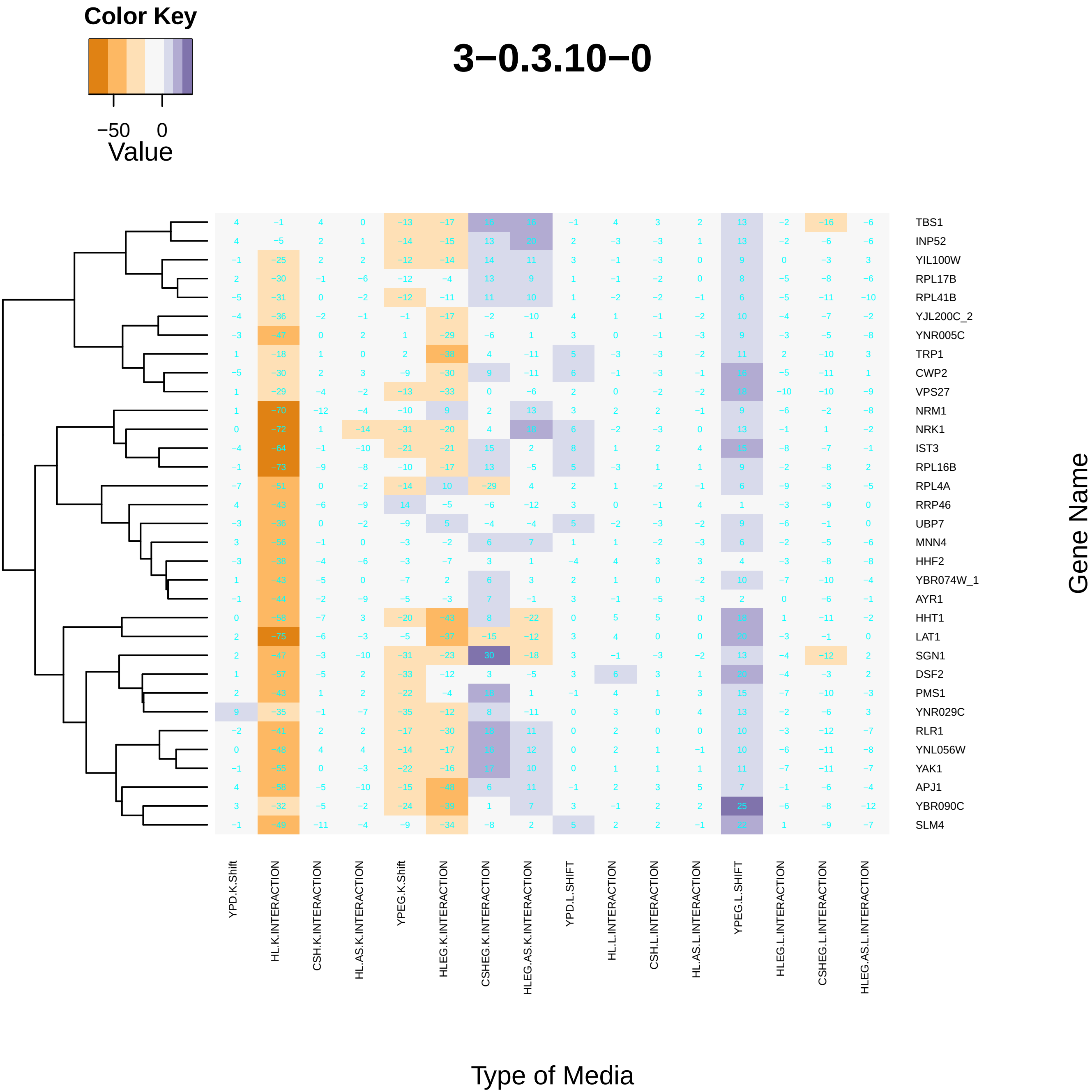

Color Key
−50
0
Value
3−0.3.10−0
TBS1
INP52
YIL100W
RPL17B
RPL41B
YJL200C_2
YNR005C
TRP1
CWP2
VPS27
NRM1
NRK1
IST3
RPL16B
RPL4A
RRP46
UBP7
MNN4
HHF2
YBR074W_1
AYR1
HHT1
LAT1
SGN1
DSF2
PMS1
YNR029C
RLR1
YNL056W
YAK1
APJ1
YBR090C
SLM4
Gene Name
YPD.K.Shift
HL.K.INTERACTION
CSH.K.INTERACTION
HL.AS.K.INTERACTION
YPEG.K.Shift
HLEG.K.INTERACTION
CSHEG.K.INTERACTION
HLEG.AS.K.INTERACTION
YPD.L.SHIFT
HL.L.INTERACTION
CSH.L.INTERACTION
HL.AS.L.INTERACTION
YPEG.L.SHIFT
HLEG.L.INTERACTION
CSHEG.L.INTERACTION
HLEG.AS.L.INTERACTION
Type of Media

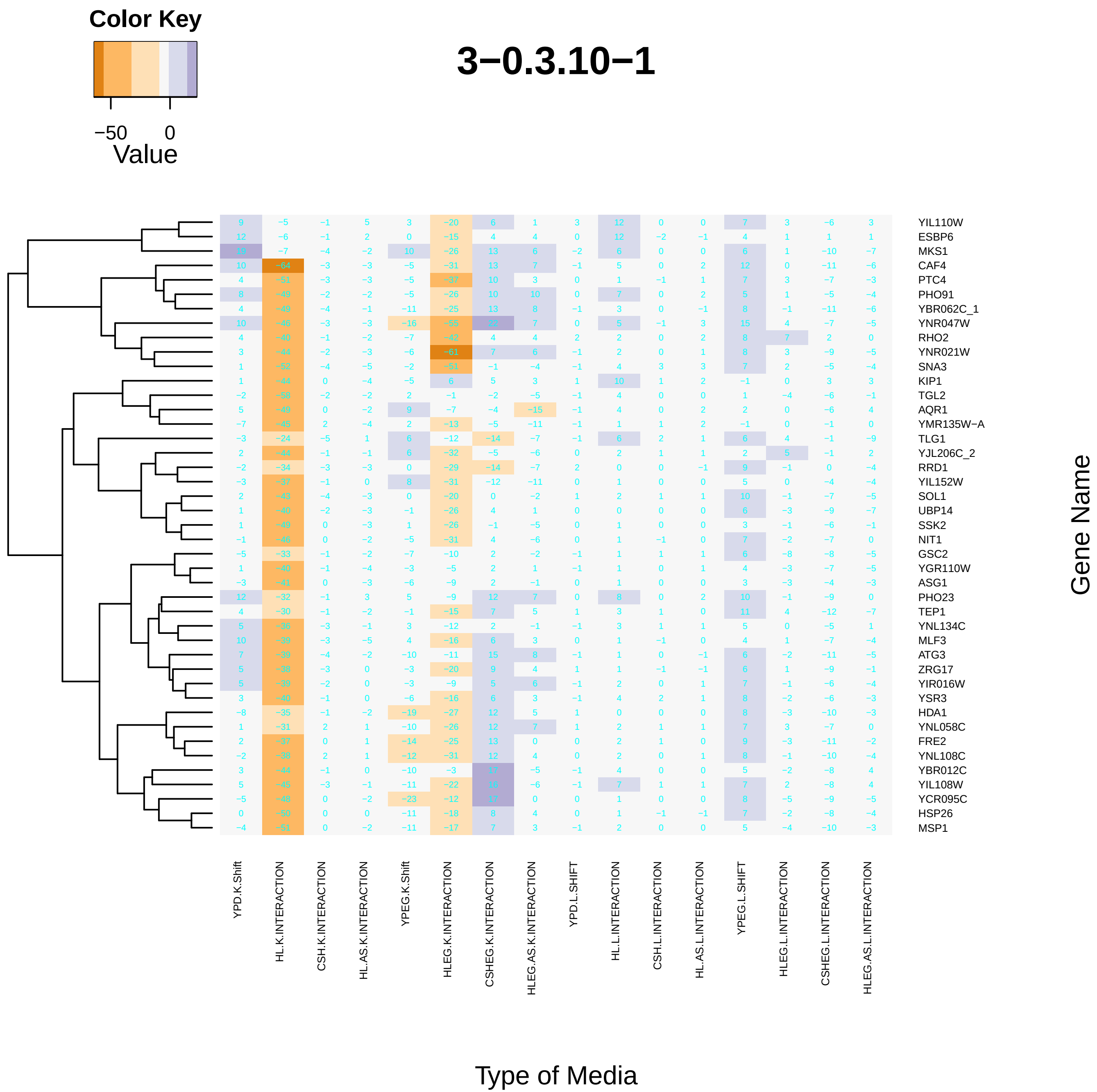
Color Key
−50
0
Value
3−0.3.10−1
YPD.K.Shift
HL.K.INTERACTION
CSH.K.INTERACTION
HL.AS.K.INTERACTION
YPEG.K.Shift
HLEG.K.INTERACTION
CSHEG.K.INTERACTION
HLEG.AS.K.INTERACTION
YPD.L.SHIFT
HL.L.INTERACTION
CSH.L.INTERACTION
HL.AS.L.INTERACTION
YPEG.L.SHIFT
HLEG.L.INTERACTION
CSHEG.L.INTERACTION
HLEG.AS.L.INTERACTION
YIL110W
ESBP6
MKS1
CAF4
PTC4
PHO91
YBR062C_1
YNR047W
RHO2
YNR021W
SNA3
KIP1
TGL2
AQR1
YMR135W−A
TLG1
YJL206C_2
RRD1
YIL152W
SOL1
UBP14
SSK2
NIT1
GSC2
YGR110W
ASG1
PHO23
TEP1
YNL134C
MLF3
ATG3
ZRG17
YIR016W
YSR3
HDA1
YNL058C
FRE2
YNL108C
YBR012C
YIL108W
YCR095C
HSP26
MSP1
Gene Name
Type of Media

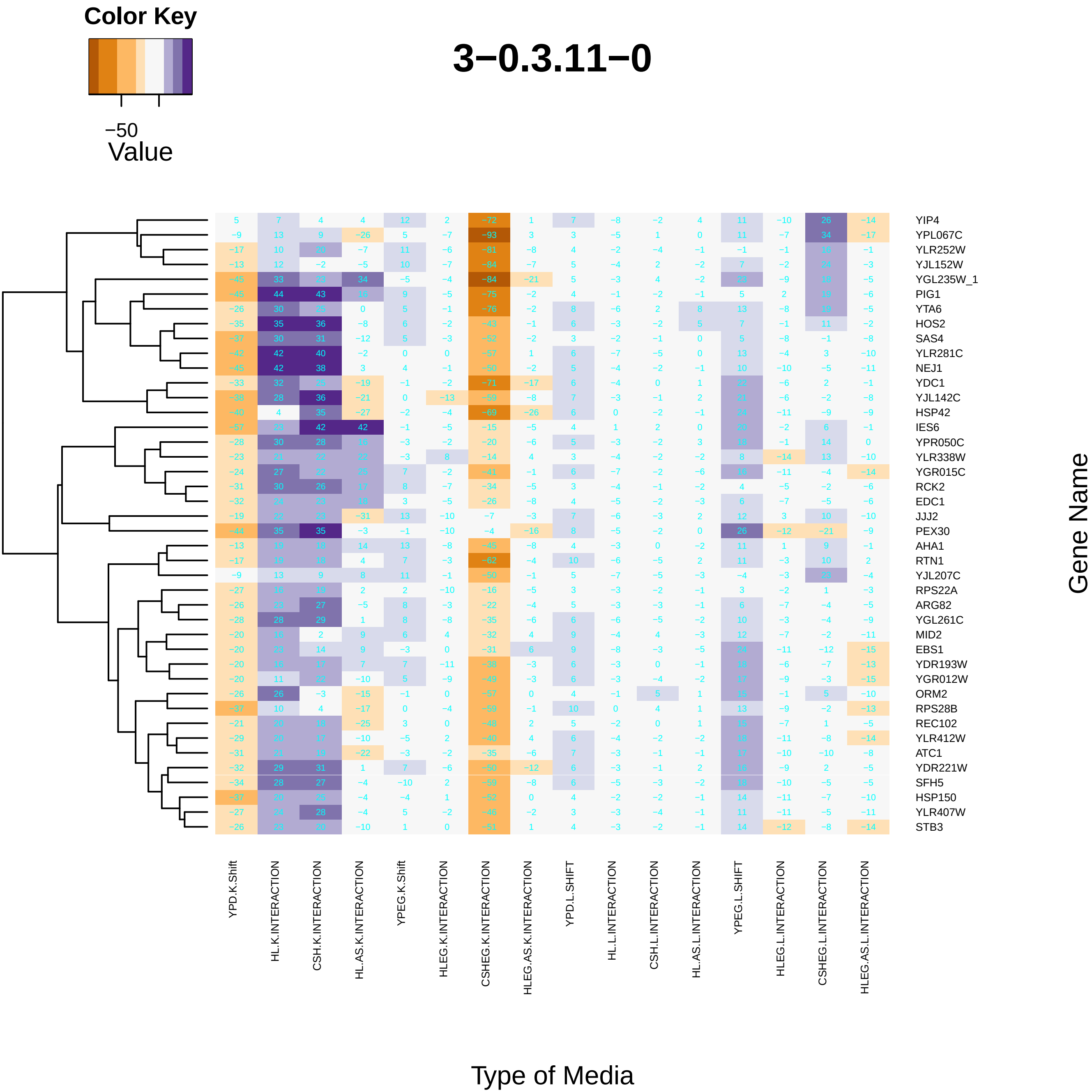

Color Key
−50
Value
3−0.3.11−0
YIP4
YPL067C
YLR252W
YJL152W
YGL235W_1
PIG1
YTA6
HOS2
SAS4
YLR281C
NEJ1
YDC1
YJL142C
HSP42
IES6
YPR050C
YLR338W
YGR015C
RCK2
EDC1
JJJ2
PEX30
AHA1
RTN1
YJL207C
RPS22A
ARG82
YGL261C
MID2
EBS1
YDR193W
YGR012W
ORM2
RPS28B
REC102
YLR412W
ATC1
YDR221W
SFH5
HSP150
YLR407W
STB3
Gene Name
YPD.K.Shift
HL.K.INTERACTION
CSH.K.INTERACTION
HL.AS.K.INTERACTION
YPEG.K.Shift
HLEG.K.INTERACTION
CSHEG.K.INTERACTION
HLEG.AS.K.INTERACTION
YPD.L.SHIFT
HL.L.INTERACTION
CSH.L.INTERACTION
HL.AS.L.INTERACTION
YPEG.L.SHIFT
HLEG.L.INTERACTION
CSHEG.L.INTERACTION
HLEG.AS.L.INTERACTION
Type of Media

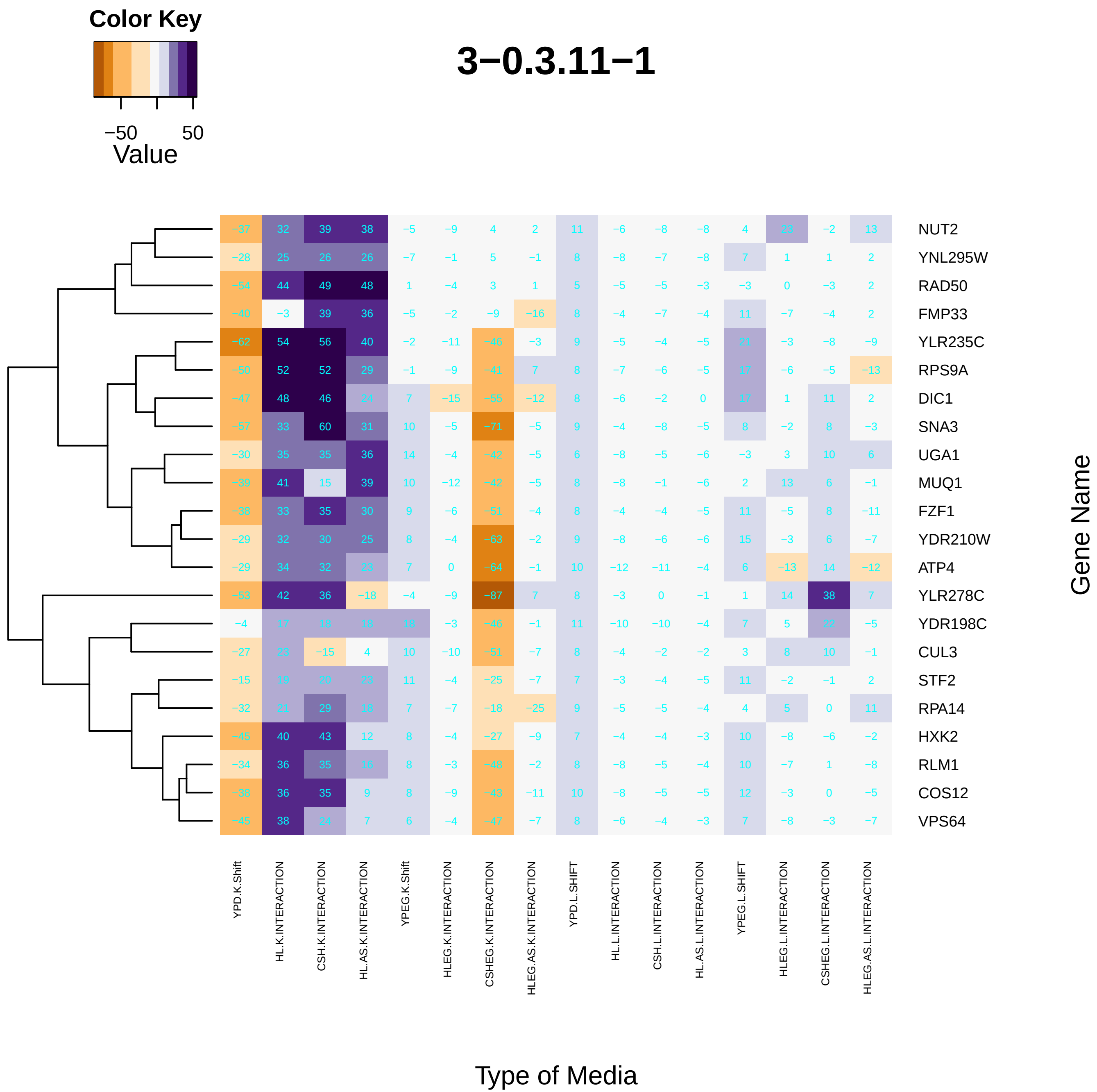
Color Key
−50
50
Value
3−0.3.11−1
NUT2
YNL295W
RAD50
FMP33
YLR235C
RPS9A
DIC1
SNA3
UGA1
MUQ1
FZF1
YDR210W
ATP4
YLR278C
YDR198C
CUL3
STF2
RPA14
HXK2
RLM1
COS12
VPS64
Gene Name
YPD.K.Shift
HL.K.INTERACTION
CSH.K.INTERACTION
HL.AS.K.INTERACTION
YPEG.K.Shift
HLEG.K.INTERACTION
CSHEG.K.INTERACTION
HLEG.AS.K.INTERACTION
YPD.L.SHIFT
HL.L.INTERACTION
CSH.L.INTERACTION
HL.AS.L.INTERACTION
YPEG.L.SHIFT
HLEG.L.INTERACTION
CSHEG.L.INTERACTION
HLEG.AS.L.INTERACTION
Type of Media

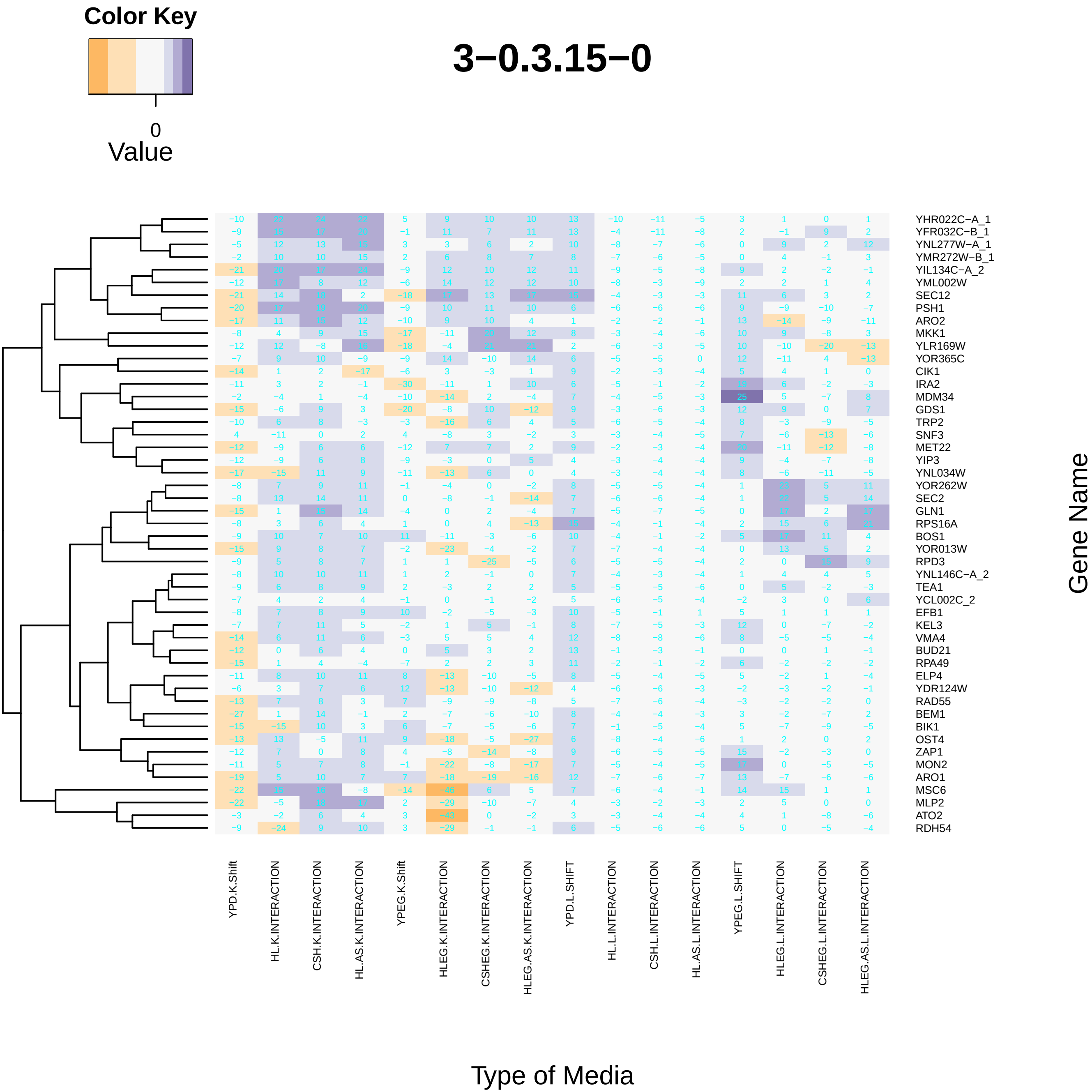
Color Key
0
Value
3−0.3.15−0
YHR022C−A_1
YFR032C−B_1
YNL277W−A_1
YMR272W−B_1
YIL134C−A_2
YML002W
SEC12
PSH1
ARO2
MKK1
YLR169W
YOR365C
CIK1
IRA2
MDM34
GDS1
TRP2
SNF3
MET22
YIP3
YNL034W
YOR262W
SEC2
GLN1
RPS16A
BOS1
YOR013W
RPD3
YNL146C−A_2
TEA1
YCL002C_2
EFB1
KEL3
VMA4
BUD21
RPA49
ELP4
YDR124W
RAD55
BEM1
BIK1
OST4
ZAP1
MON2
ARO1
MSC6
MLP2
ATO2
RDH54
Gene Name
YPD.K.Shift
HL.K.INTERACTION
CSH.K.INTERACTION
HL.AS.K.INTERACTION
YPEG.K.Shift
HLEG.K.INTERACTION
CSHEG.K.INTERACTION
HLEG.AS.K.INTERACTION
YPD.L.SHIFT
HL.L.INTERACTION
CSH.L.INTERACTION
HL.AS.L.INTERACTION
YPEG.L.SHIFT
HLEG.L.INTERACTION
CSHEG.L.INTERACTION
HLEG.AS.L.INTERACTION
Type of Media

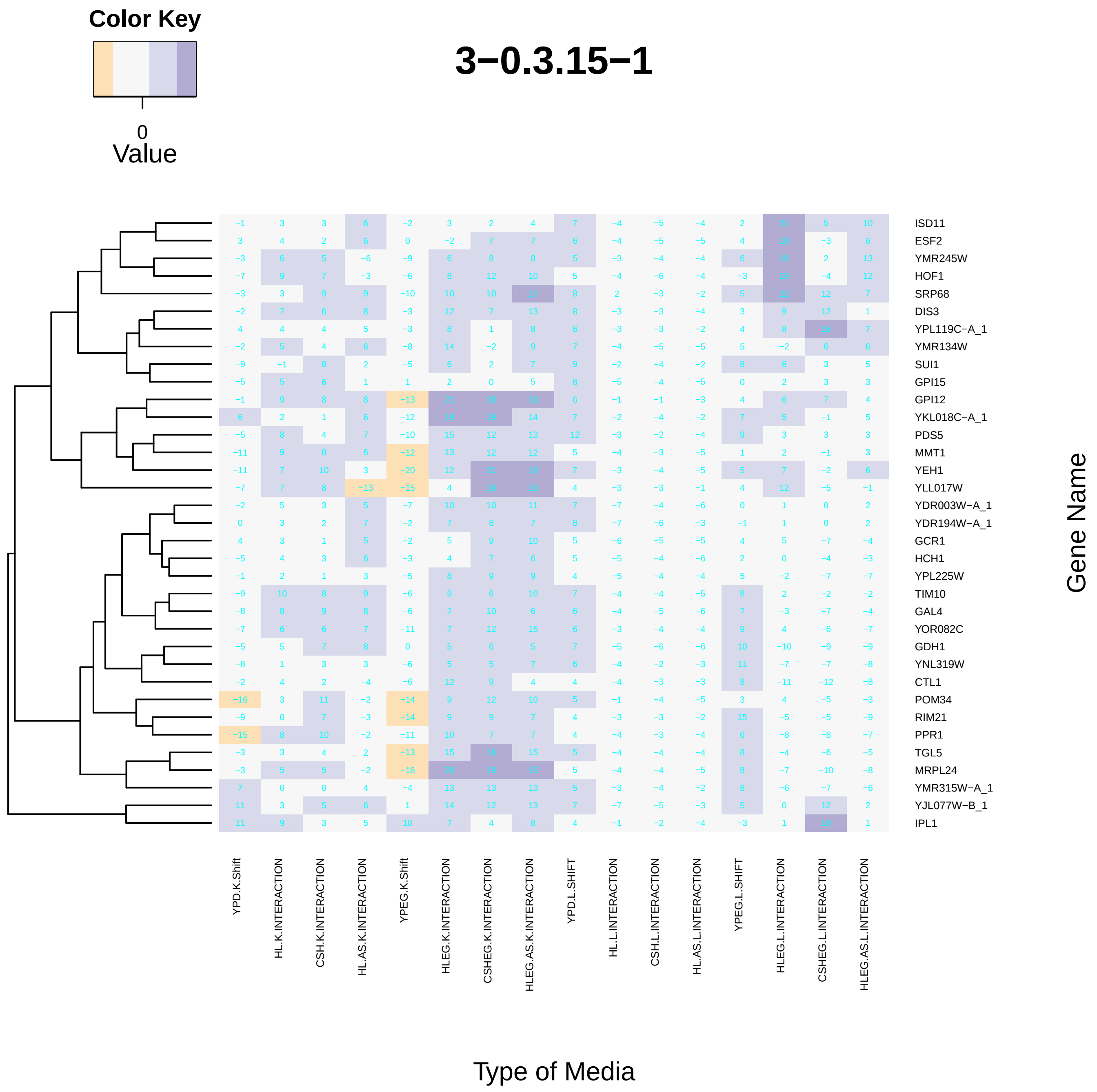
3−0.3.15−1
Color Key
0
Value
ISD11
ESF2
YMR245W
HOF1
SRP68
DIS3
YPL119C−A_1
YMR134W
SUI1
GPI15
GPI12
YKL018C−A_1
PDS5
MMT1
YEH1
YLL017W
YDR003W−A_1
YDR194W−A_1
GCR1
HCH1
YPL225W
TIM10
GAL4
YOR082C
GDH1
YNL319W
CTL1
POM34
RIM21
PPR1
TGL5
MRPL24
YMR315W−A_1
YJL077W−B_1
IPL1
Gene Name
YPD.K.Shift
HL.K.INTERACTION
CSH.K.INTERACTION
HL.AS.K.INTERACTION
YPEG.K.Shift
HLEG.K.INTERACTION
CSHEG.K.INTERACTION
HLEG.AS.K.INTERACTION
YPD.L.SHIFT
HL.L.INTERACTION
CSH.L.INTERACTION
HL.AS.L.INTERACTION
YPEG.L.SHIFT
HLEG.L.INTERACTION
CSHEG.L.INTERACTION
HLEG.AS.L.INTERACTION
Type of Media

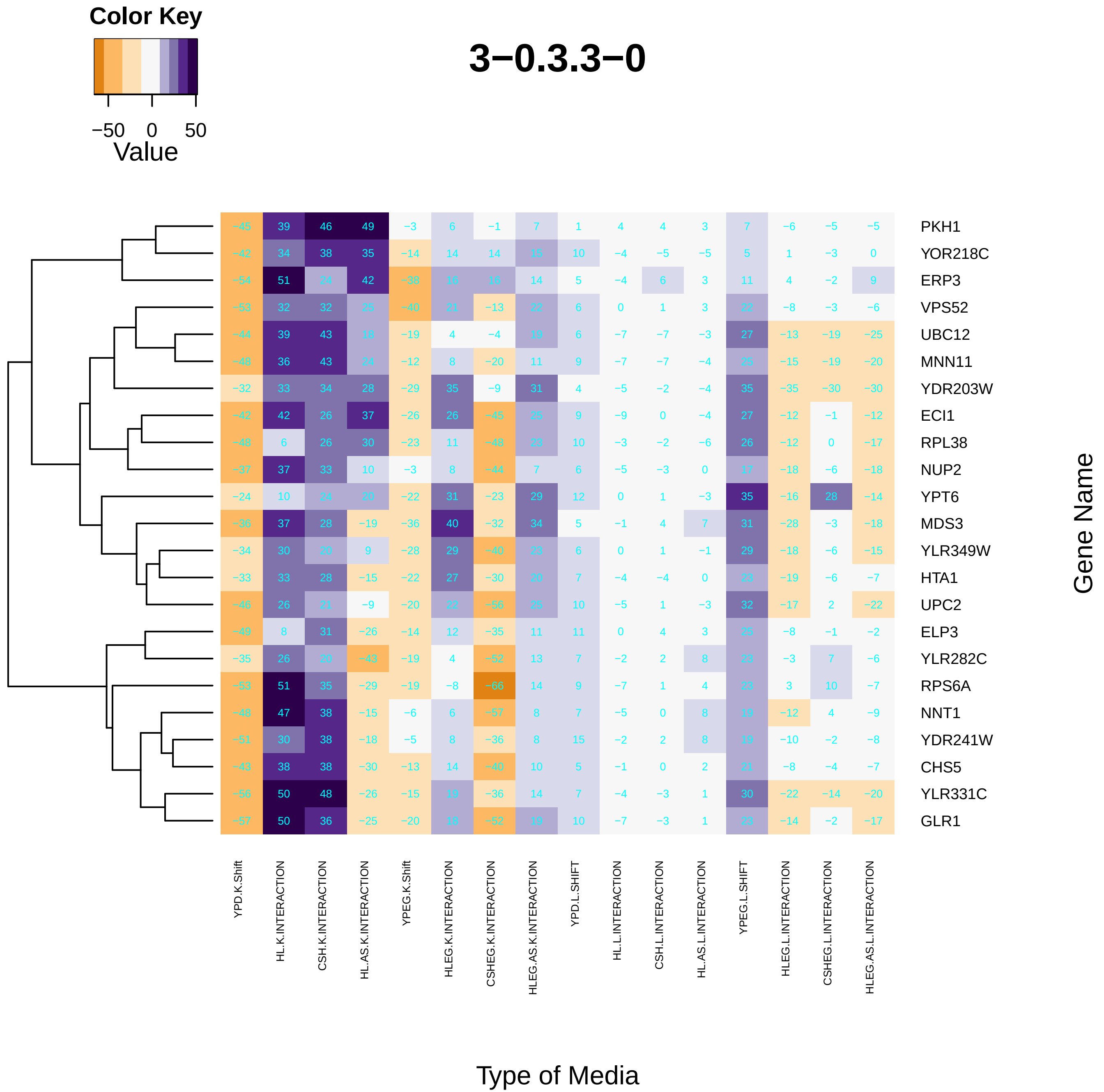
Color Key
−50 0 50
Value
3−0.3.3−0
PKH1
YOR218C
ERP3
VPS52
UBC12
MNN11
YDR203W
ECI1
RPL38
NUP2
YPT6
MDS3
YLR349W
HTA1
UPC2
ELP3
YLR282C
RPS6A
NNT1
YDR241W
CHS5
YLR331C
GLR1
Gene Name
YPD.K.Shift
HL.K.INTERACTION
CSH.K.INTERACTION
HL.AS.K.INTERACTION
YPEG.K.Shift
HLEG.K.INTERACTION
CSHEG.K.INTERACTION
HLEG.AS.K.INTERACTION
YPD.L.SHIFT
HL.L.INTERACTION
CSH.L.INTERACTION
HL.AS.L.INTERACTION
YPEG.L.SHIFT
HLEG.L.INTERACTION
CSHEG.L.INTERACTION
HLEG.AS.L.INTERACTION
Type of Media

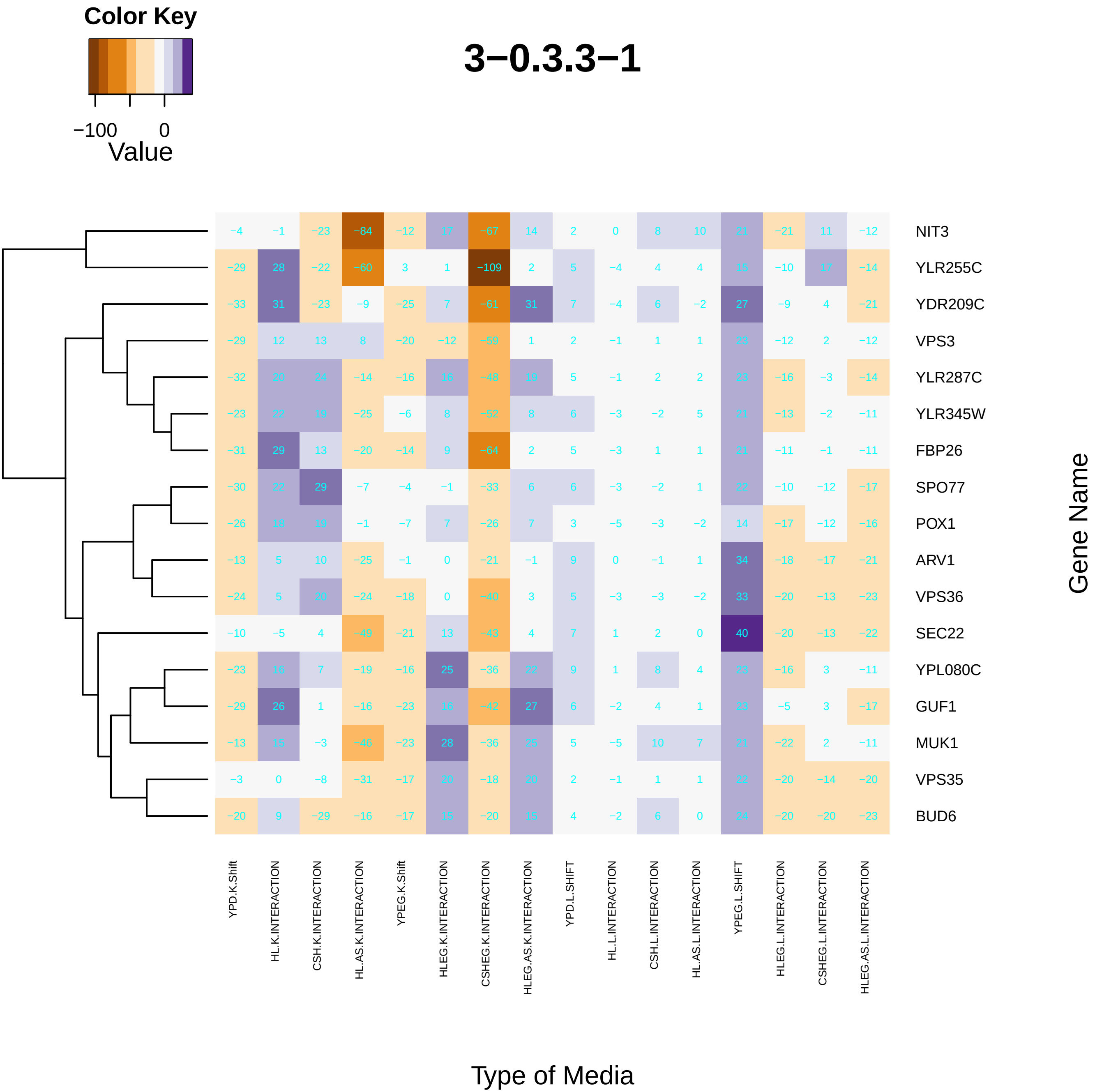
3−0.3.3−1
Color Key
−100
0
Value
YPD.K.Shift
HL.K.INTERACTION
CSH.K.INTERACTION
HL.AS.K.INTERACTION
YPEG.K.Shift
HLEG.K.INTERACTION
CSHEG.K.INTERACTION
HLEG.AS.K.INTERACTION
YPD.L.SHIFT
HL.L.INTERACTION
CSH.L.INTERACTION
HL.AS.L.INTERACTION
YPEG.L.SHIFT
HLEG.L.INTERACTION
CSHEG.L.INTERACTION
HLEG.AS.L.INTERACTION
NIT3 −4 −1 −23 −84 −12 17 −67 14 2 0 8 10 21 −21 11 −12
YLR255C −29 28 −22 −60 3 1 −109 2 5 −4 4 4 15 −10 17 −14
YDR209C −33 31 −23 −9 −25 7 −61 31 7 −4 6 −2 27 −9 4 −21
VPS3 −29 12 13 8 −20 −12 −59 1 2 −1 1 1 23 −12 2 −12
YLR287C −32 20 24 −14 −16 16 −48 19 5 −1 2 2 23 −16 −3 −14
YLR345W −23 22 19 −25 −6 8 −52 8 6 −3 −2 5 21 −13 −2 −11
FBP26 −31 29 13 −20 −14 9 −64 2 5 −3 1 1 21 −11 −1 −11
SPO77 −30 22 29 −7 −4 −1 −33 6 6 −3 −2 1 22 −10 −12 −17
POX1 −26 18 19 −1 −7 7 −26 7 3 −5 −3 −2 14 −17 −12 −16
ARV1 −13 5 10 −25 −1 0 −21 −1 9 0 −1 1 34 −18 −17 −21
VPS36 −24 5 20 −24 −18 0 −40 3 5 −3 −3 −2 33 −20 −13 −23
SEC22 −10 −5 4 −49 −21 13 −43 4 7 1 2 0 40 −20 −13 −22
YPL080C −23 16 7 −19 −16 25 −36 22 9 1 8 4 23 −16 3 −11
GUF1 −29 26 1 −16 −23 16 −42 27 6 −2 4 1 23 −5 3 −17
MUK1 −13 15 −3 −46 −23 28 −36 25 5 −5 10 7 21 −22 2 −11
VPS35 −3 0 −8 −31 −17 20 −18 20 2 −1 1 1 22 −20 −14 −20
BUD6 −20 9 −29 −16 −17 15 −20 15 4 −2 6 0 24 −20 −20 −23
Gene Name
Type of Media

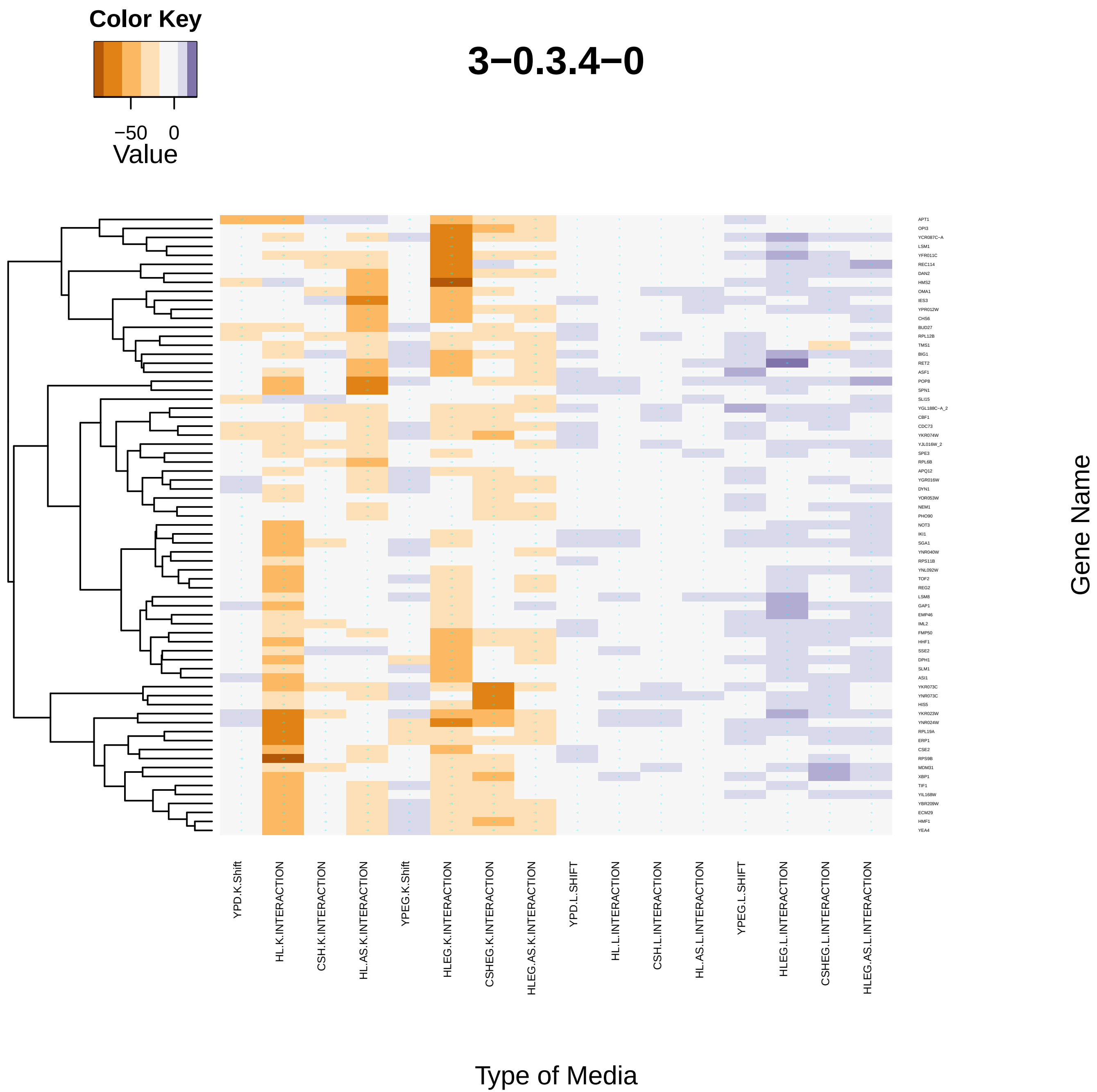
Color Key
−50
0
Value
3−0.3.4−0
APT1
OPI3
YCR087C−A
LSM1
YFR011C
REC114
DAN2
HMS2
OMA1
IES3
YPR012W
CHS6
BUD27
RPL12B
TMS1
BIG1
RET2
ASF1
POP8
SPN1
SLI15
YGL188C−A_2
CBF1
CDC73
YKR074W
YJL016W_2
SPE3
RPL6B
APQ12
YGR016W
DYN1
YOR053W
NEM1
PHO90
NOT3
IKI1
SGA1
YNR040W
RPS11B
YNL092W
TOF2
REG2
LSM8
GAP1
EMP46
IML2
FMP50
HHF1
SSE2
DPH1
SLM1
ASI1
YKR073C
YNR073C
HIS5
YKR023W
YNR024W
RPL19A
ERP1
CSE2
RPS9B
MDM31
XBP1
TIF1
YIL168W
YBR209W
ECM29
HMF1
YEA4
Gene Name
YPD.K.Shift
HL.K.INTERACTION
CSH.K.INTERACTION
HL.AS.K.INTERACTION
YPEG.K.Shift
HLEG.K.INTERACTION
CSHEG.K.INTERACTION
HLEG.AS.K.INTERACTION
YPD.L.SHIFT
HL.L.INTERACTION
CSH.L.INTERACTION
HL.AS.L.INTERACTION
YPEG.L.SHIFT
HLEG.L.INTERACTION
CSHEG.L.INTERACTION
HLEG.AS.L.INTERACTION
Type of Media

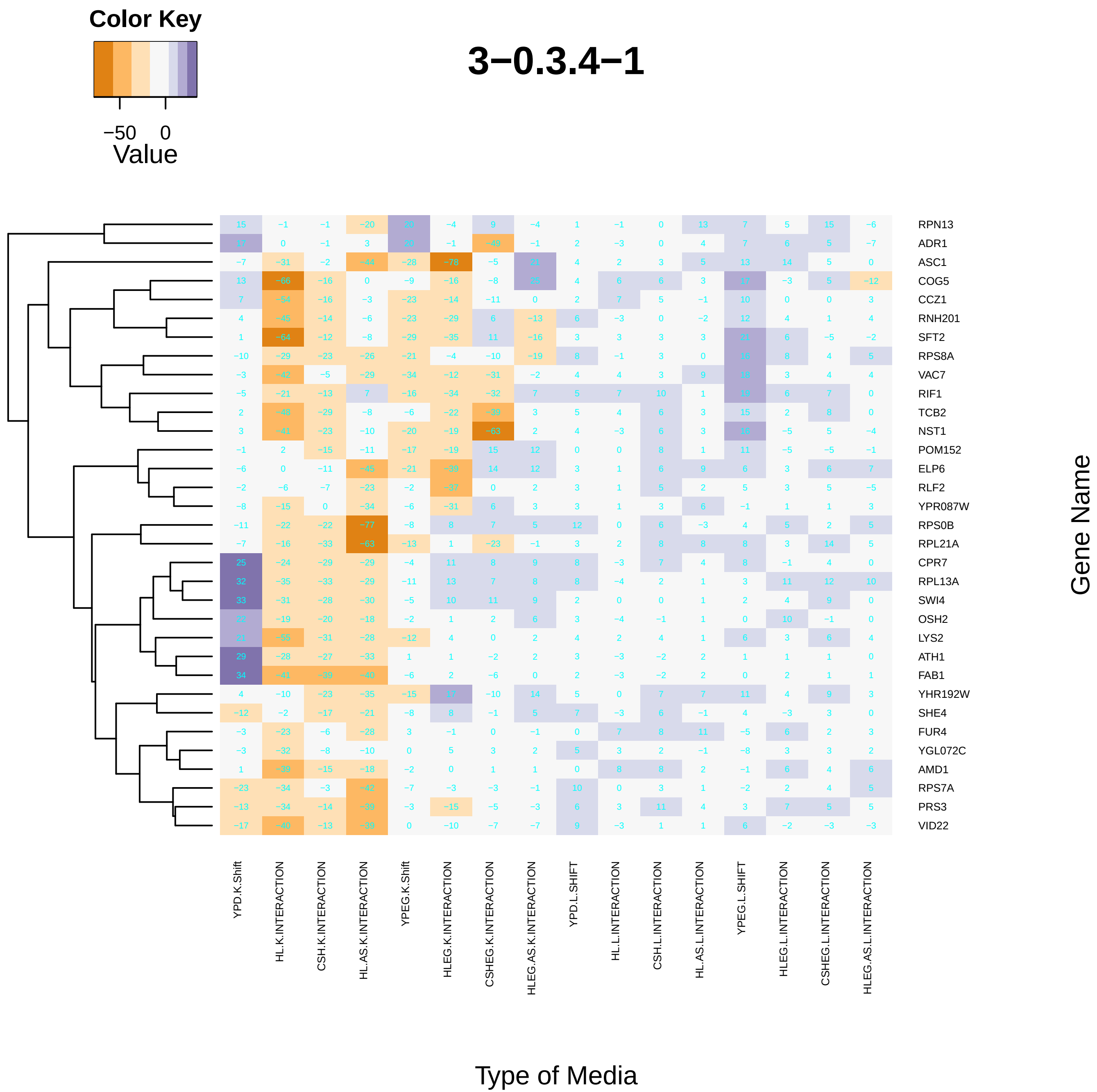

Color Key
−50
0
Value
3−0.3.4−1
RPN13
ADR1
ASC1
COG5
CCZ1
RNH201
SFT2
RPS8A
VAC7
RIF1
TCB2
NST1
POM152
ELP6
RLF2
YPR087W
RPS0B
RPL21A
CPR7
RPL13A
SWI4
OSH2
LYS2
ATH1
FAB1
YHR192W
SHE4
FUR4
YGL072C
AMD1
RPS7A
PRS3
VID22
Gene Name
YPD.K.Shift
HL.K.INTERACTION
CSH.K.INTERACTION
HL.AS.K.INTERACTION
YPEG.K.Shift
HLEG.K.INTERACTION
CSHEG.K.INTERACTION
HLEG.AS.K.INTERACTION
YPD.L.SHIFT
HL.L.INTERACTION
CSH.L.INTERACTION
HL.AS.L.INTERACTION
YPEG.L.SHIFT
HLEG.L.INTERACTION
CSHEG.L.INTERACTION
HLEG.AS.L.INTERACTION
Type of Media

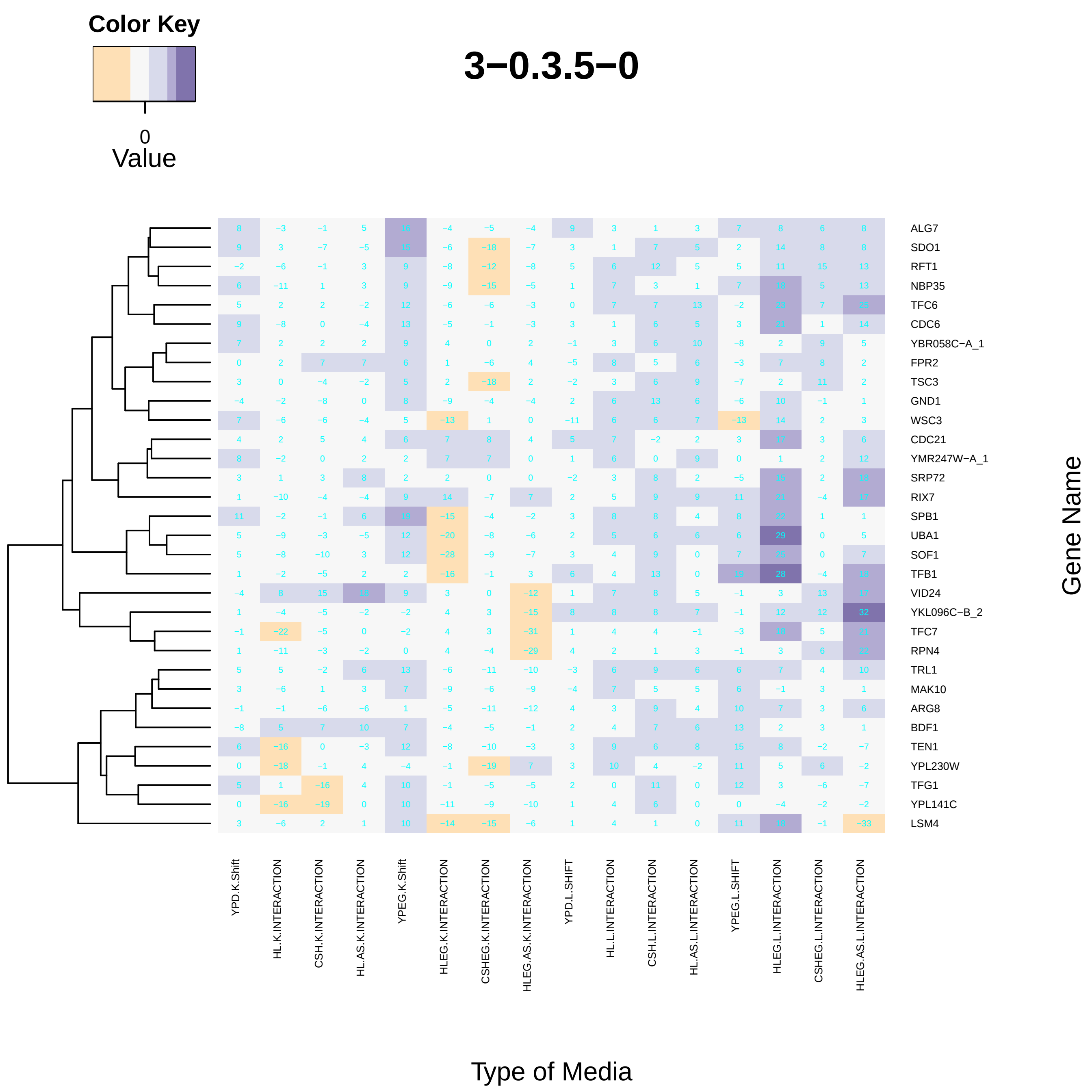
3−0.3.5−0
Color Key
0
Value
ALG7
SDO1
RFT1
NBP35
TFC6
CDC6
YBR058C−A_1
FPR2
TSC3
GND1
WSC3
CDC21
YMR247W−A_1
SRP72
RIX7
SPB1
UBA1
SOF1
TFB1
VID24
YKL096C−B_2
TFC7
RPN4
TRL1
MAK10
ARG8
BDF1
TEN1
YPL230W
TFG1
YPL141C
LSM4
Gene Name
YPD.K.Shift
HL.K.INTERACTION
CSH.K.INTERACTION
HL.AS.K.INTERACTION
YPEG.K.Shift
HLEG.K.INTERACTION
CSHEG.K.INTERACTION
HLEG.AS.K.INTERACTION
YPD.L.SHIFT
HL.L.INTERACTION
CSH.L.INTERACTION
HL.AS.L.INTERACTION
YPEG.L.SHIFT
HLEG.L.INTERACTION
CSHEG.L.INTERACTION
HLEG.AS.L.INTERACTION
Type of Media

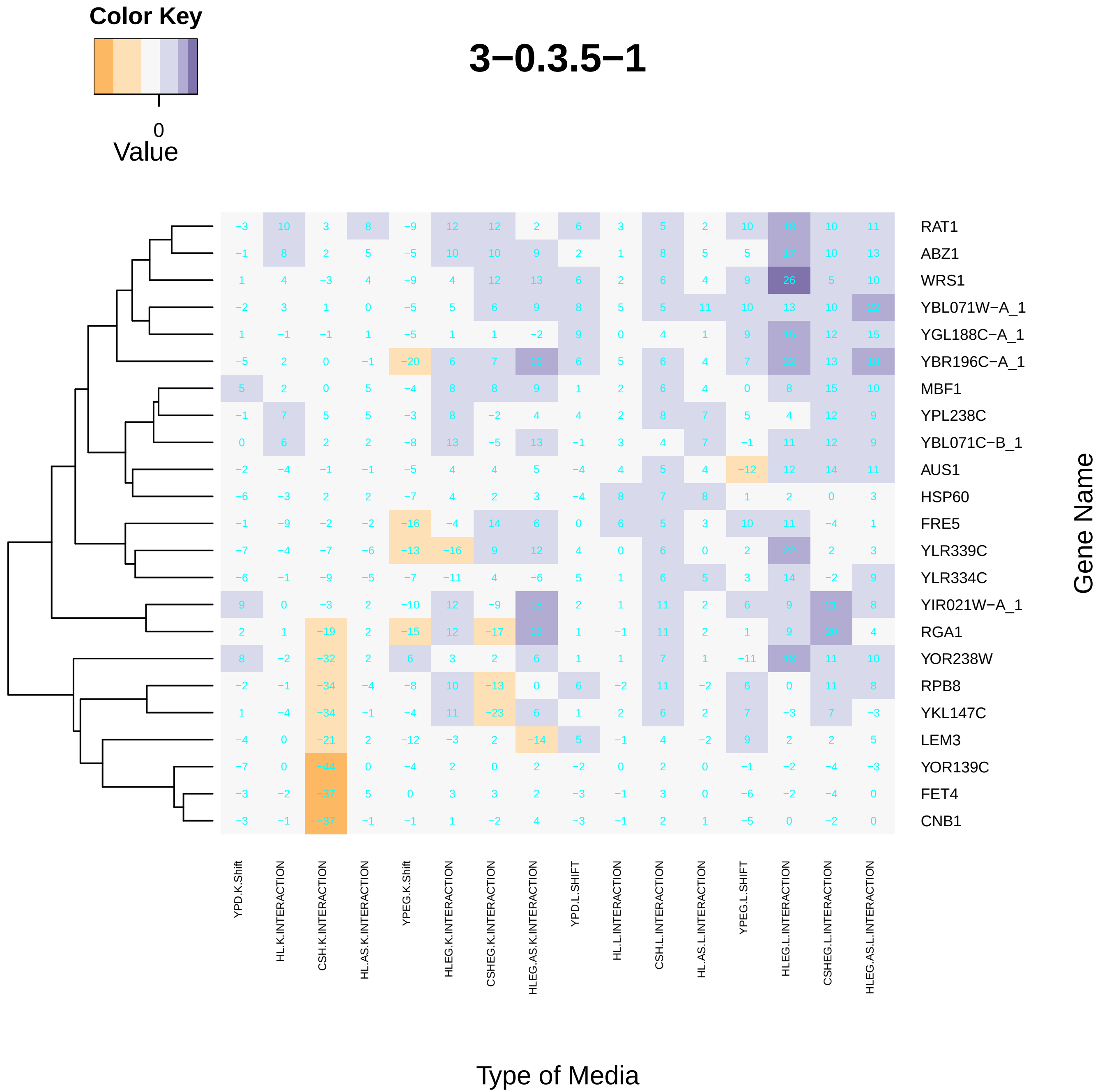
Color Key
0
Value
3−0.3.5−1
RAT1 −3 10 3 8 −9 12 12 2 6 3 5 2 10 18 10 11
ABZ1 −1 8 2 5 −5 10 10 9 2 1 8 5 5 17 10 13
WRS1 1 4 −3 4 −9 4 12 13 6 2 6 4 9 26 5 10
YBL071W−A_1 −2 3 1 0 −5 5 6 9 8 5 5 11 10 13 10 22
YGL188C−A_1 1 −1 −1 1 −5 1 1 −2 9 0 4 1 9 15 12 15
YBR196C−A_1 −5 2 0 −1 −20 6 7 19 6 5 6 4 7 22 13 18
MBF1 5 2 0 5 −4 8 8 9 1 2 6 4 0 8 15 10
YPL238C −1 7 5 5 −3 8 −2 4 4 2 8 7 5 4 12 9
YBL071C−B_1 0 6 2 2 −8 13 −5 13 −1 3 4 7 −1 11 12 9
AUS1 −2 −4 −1 −1 −5 4 4 5 −4 4 5 4 −12 12 14 11
HSP60 −6 −3 2 2 −7 4 2 3 −4 8 7 8 1 2 0 3
FRE5 −1 −9 −2 −2 −16 −4 14 6 0 6 5 3 10 11 −4 1
YLR339C −7 −4 −7 −6 −13 −16 9 12 4 0 6 0 2 22 2 3
YLR334C −6 −1 −9 −5 −7 −11 4 −6 5 1 6 5 3 14 −2 9
YIR021W−A_1 9 0 −3 2 −10 12 −9 15 2 1 11 2 6 9 21 8
RGA1 2 1 −19 2 −15 12 −17 15 1 −1 11 2 1 9 20 4
YOR238W 8 −2 −32 2 6 3 2 6 1 1 7 1 −11 18 11 10
RPB8 −2 −1 −34 −4 −8 10 −13 0 6 −2 11 −2 6 0 11 8
YKL147C 1 −4 −34 −1 −4 11 −23 6 1 2 6 2 7 −3 7 −3
LEM3 −4 0 −21 2 −12 −3 2 −14 5 −1 4 −2 9 2 2 5
YOR139C −7 0 −44 0 −4 2 0 2 −2 0 2 0 −1 −2 −4 −3
FET4 −3 −2 −37 5 0 3 3 2 −3 −1 3 0 −6 −2 −4 0
CNB1 −3 −1 −37 −1 −1 1 −2 4 −3 −1 2 1 −5 0 −2 0
YPD.K.Shift
HL.K.INTERACTION
CSH.K.INTERACTION
HL.AS.K.INTERACTION
YPEG.K.Shift
HLEG.K.INTERACTION
CSHEG.K.INTERACTION
HLEG.AS.K.INTERACTION
YPD.L.SHIFT
HL.L.INTERACTION
CSH.L.INTERACTION
HL.AS.L.INTERACTION
YPEG.L.SHIFT
HLEG.L.INTERACTION
CSHEG.L.INTERACTION
HLEG.AS.L.INTERACTION
Gene Name
Type of Media

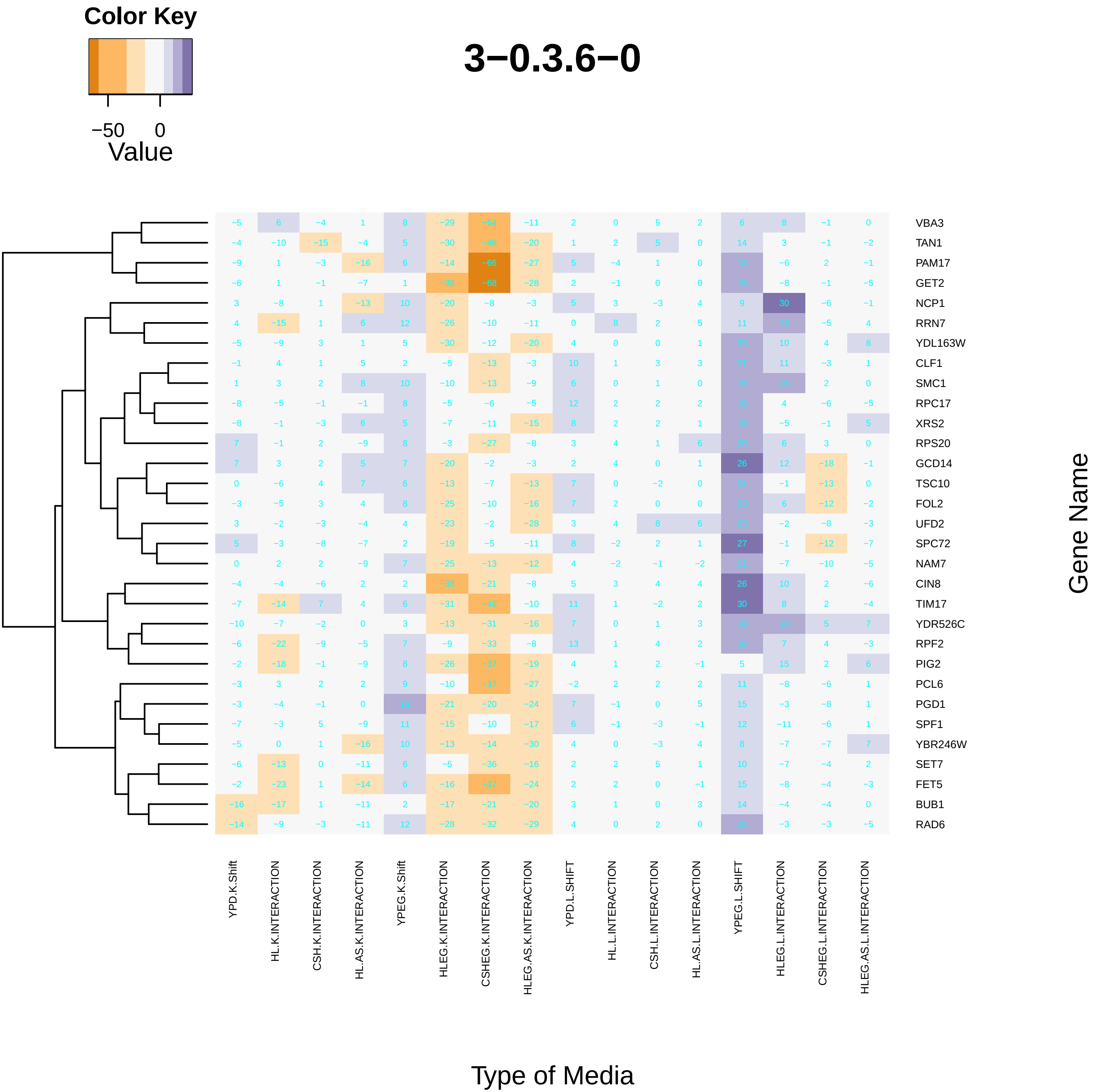

Color Key
−50
0
Value
3−0.3.6−0
VBA3
TAN1
PAM17
GET2
NCP1
RRN7
YDL163W
CLF1
SMC1
RPC17
XRS2
RPS20
GCD14
TSC10
FOL2
UFD2
SPC72
NAM7
CIN8
TIM17
YDR526C
RPF2
PIG2
PCL6
PGD1
SPF1
YBR246W
SET7
FET5
BUB1
RAD6
Gene Name
YPD.K.Shift
HL.K.INTERACTION
CSH.K.INTERACTION
HL.AS.K.INTERACTION
YPEG.K.Shift
HLEG.K.INTERACTION
CSHEG.K.INTERACTION
HLEG.AS.K.INTERACTION
YPD.L.SHIFT
HL.L.INTERACTION
CSH.L.INTERACTION
HL.AS.L.INTERACTION
YPEG.L.SHIFT
HLEG.L.INTERACTION
CSHEG.L.INTERACTION
HLEG.AS.L.INTERACTION
Type of Media

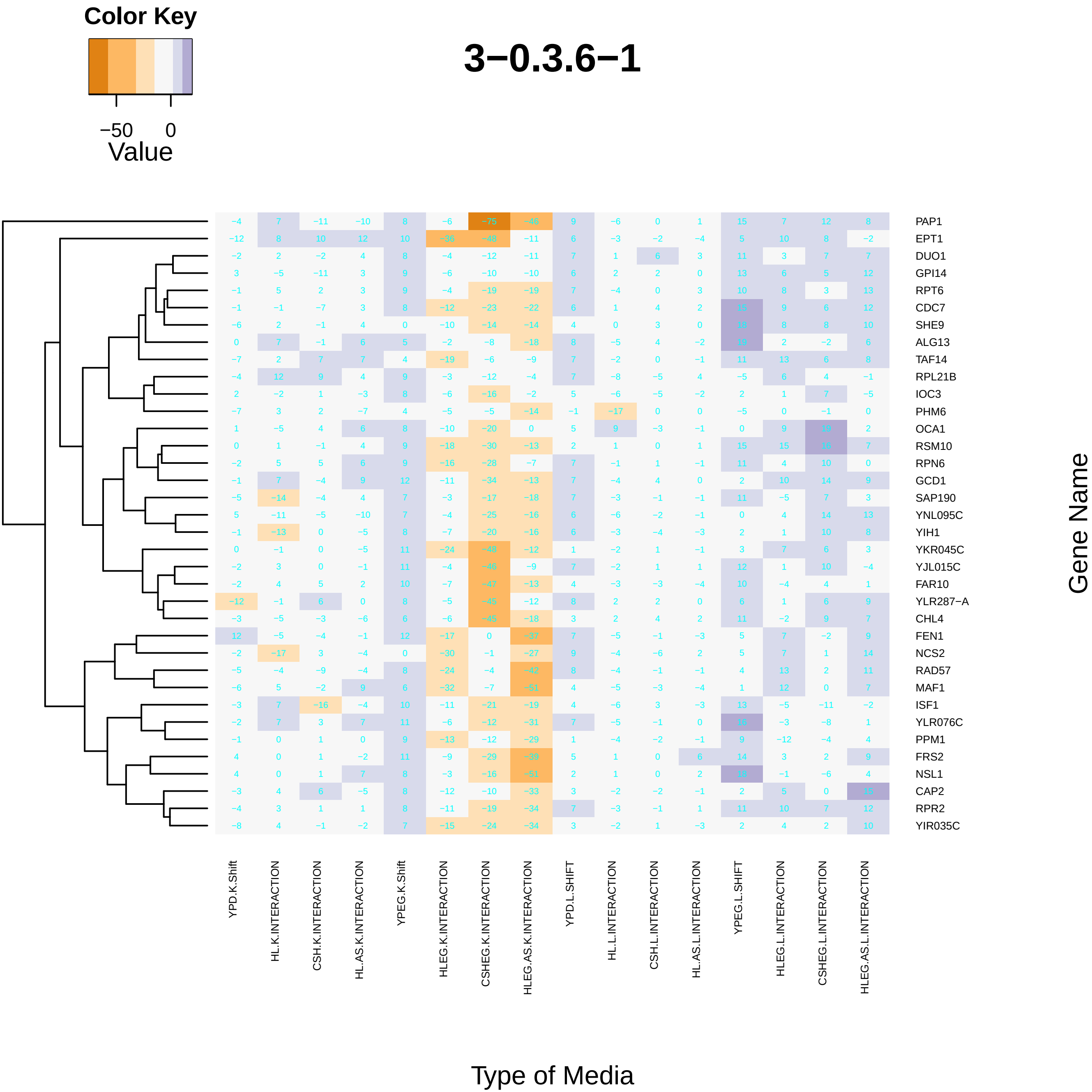
Color Key
−50
0
Value
3−0.3.6−1
PAP1
EPT1
DUO1
GPI14
RPT6
CDC7
SHE9
ALG13
TAF14
RPL21B
IOC3
PHM6
OCA1
RSM10
RPN6
GCD1
SAP190
YNL095C
YIH1
YKR045C
YJL015C
FAR10
YLR287−A
CHL4
FEN1
NCS2
RAD57
MAF1
ISF1
YLR076C
PPM1
FRS2
NSL1
CAP2
RPR2
YIR035C
Gene Name
YPD.K.Shift
HL.K.INTERACTION
CSH.K.INTERACTION
HL.AS.K.INTERACTION
YPEG.K.Shift
HLEG.K.INTERACTION
CSHEG.K.INTERACTION
HLEG.AS.K.INTERACTION
YPD.L.SHIFT
HL.L.INTERACTION
CSH.L.INTERACTION
HL.AS.L.INTERACTION
YPEG.L.SHIFT
HLEG.L.INTERACTION
CSHEG.L.INTERACTION
HLEG.AS.L.INTERACTION
Type of Media

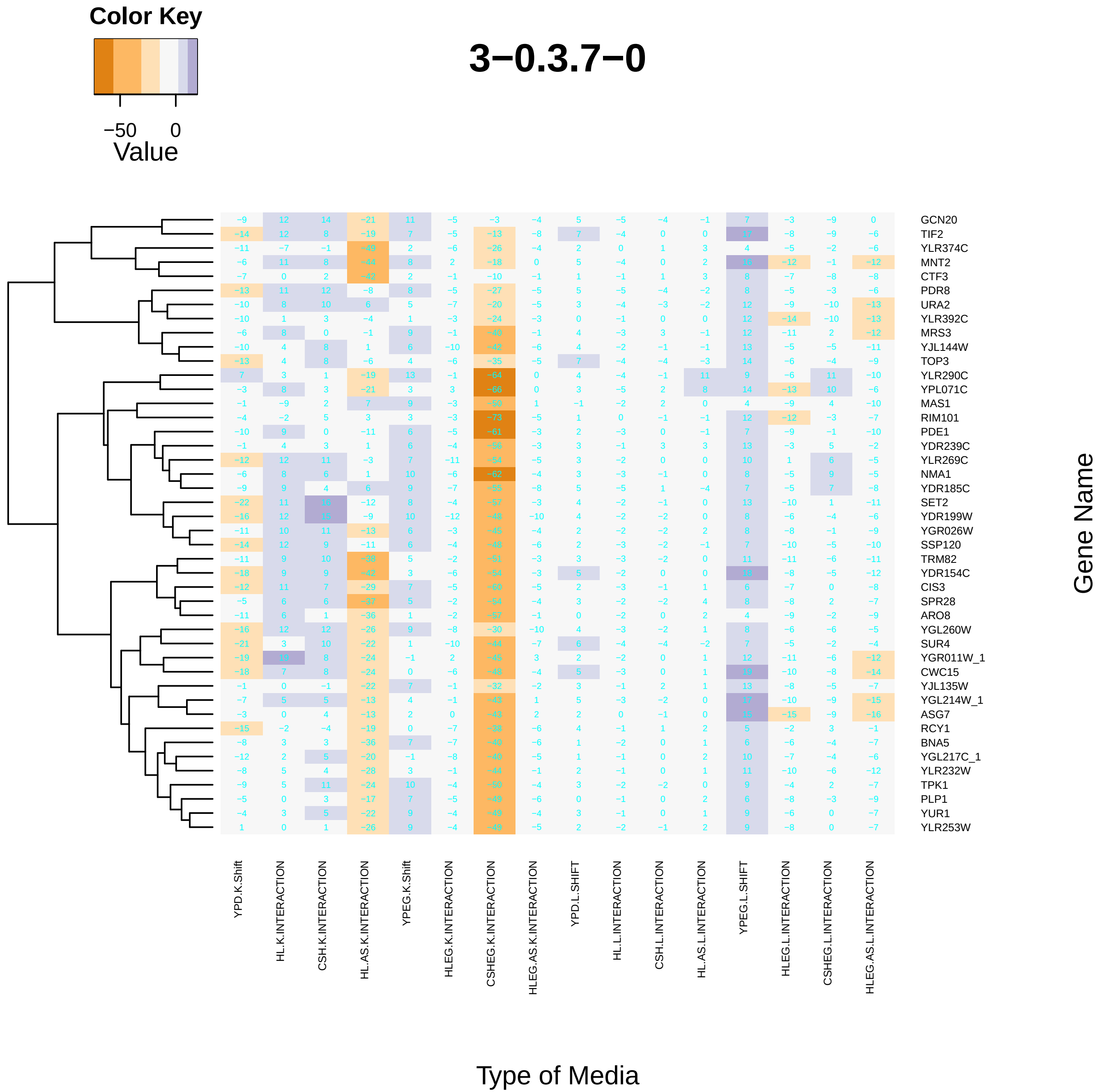
Color Key
−50
0
Value
3−0.3.7−0
GCN20
TIF2
YLR374C
MNT2
CTF3
PDR8
URA2
YLR392C
MRS3
YJL144W
TOP3
YLR290C
YPL071C
MAS1
RIM101
PDE1
YDR239C
YLR269C
NMA1
YDR185C
SET2
YDR199W
YGR026W
SSP120
TRM82
YDR154C
CIS3
SPR28
ARO8
YGL260W
SUR4
YGR011W_1
CWC15
YJL135W
YGL214W_1
ASG7
RCY1
BNA5
YGL217C_1
YLR232W
TPK1
PLP1
YUR1
YLR253W
Gene Name
YPD.K.Shift
HL.K.INTERACTION
CSH.K.INTERACTION
HL.AS.K.INTERACTION
YPEG.K.Shift
HLEG.K.INTERACTION
CSHEG.K.INTERACTION
HLEG.AS.K.INTERACTION
YPD.L.SHIFT
HL.L.INTERACTION
CSH.L.INTERACTION
HL.AS.L.INTERACTION
YPEG.L.SHIFT
HLEG.L.INTERACTION
CSHEG.L.INTERACTION
HLEG.AS.L.INTERACTION
Type of Media

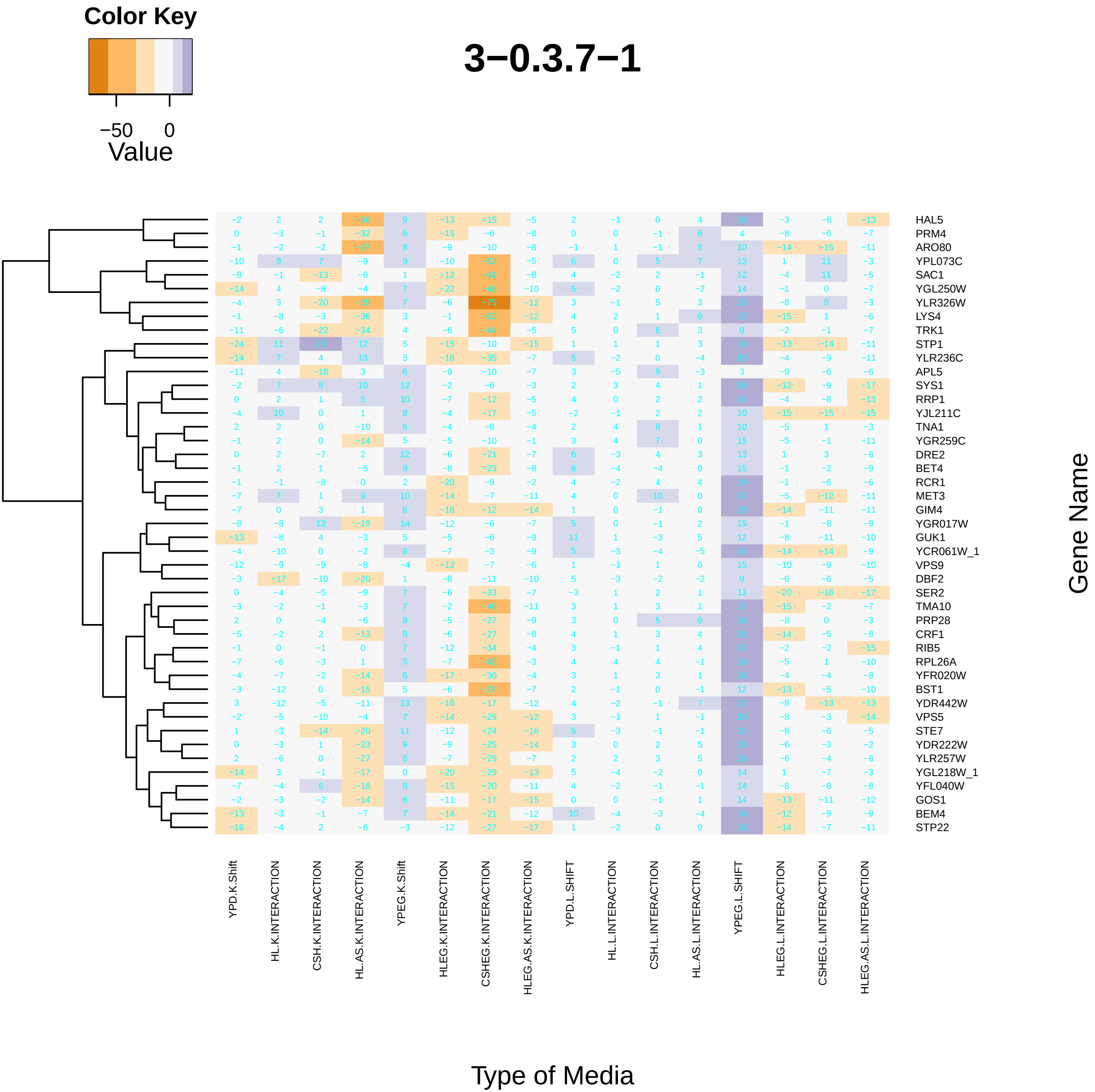
Color Key
−50
0
Value
3−0.3.7−1
YPD.K.Shift
HL.K.INTERACTION
CSH.K.INTERACTION
HL.AS.K.INTERACTION
YPEG.K.Shift
HLEG.K.INTERACTION
CSHEG.K.INTERACTION
HLEG.AS.K.INTERACTION
YPD.L.SHIFT
HL.L.INTERACTION
CSH.L.INTERACTION
HL.AS.L.INTERACTION
YPEG.L.SHIFT
HLEG.L.INTERACTION
CSHEG.L.INTERACTION
HLEG.AS.L.INTERACTION
HAL5
PRM4
ARO80
YPL073C
SAC1
YGL250W
YLR326W
LYS4
TRK1
STP1
YLR236C
APL5
SYS1
RRP1
YJL211C
TNA1
YGR259C
DRE2
BET4
RCR1
MET3
GIM4
YGR017W
GUK1
YCR061W_1
VPS9
DBF2
SER2
TMA10
PRP28
CRF1
RIB5
RPL26A
YFR020W
BST1
YDR442W
VPS5
STE7
YDR222W
YLR257W
YGL218W_1
YFL040W
GOS1
BEM4
STP22
Gene Name
Type of Media

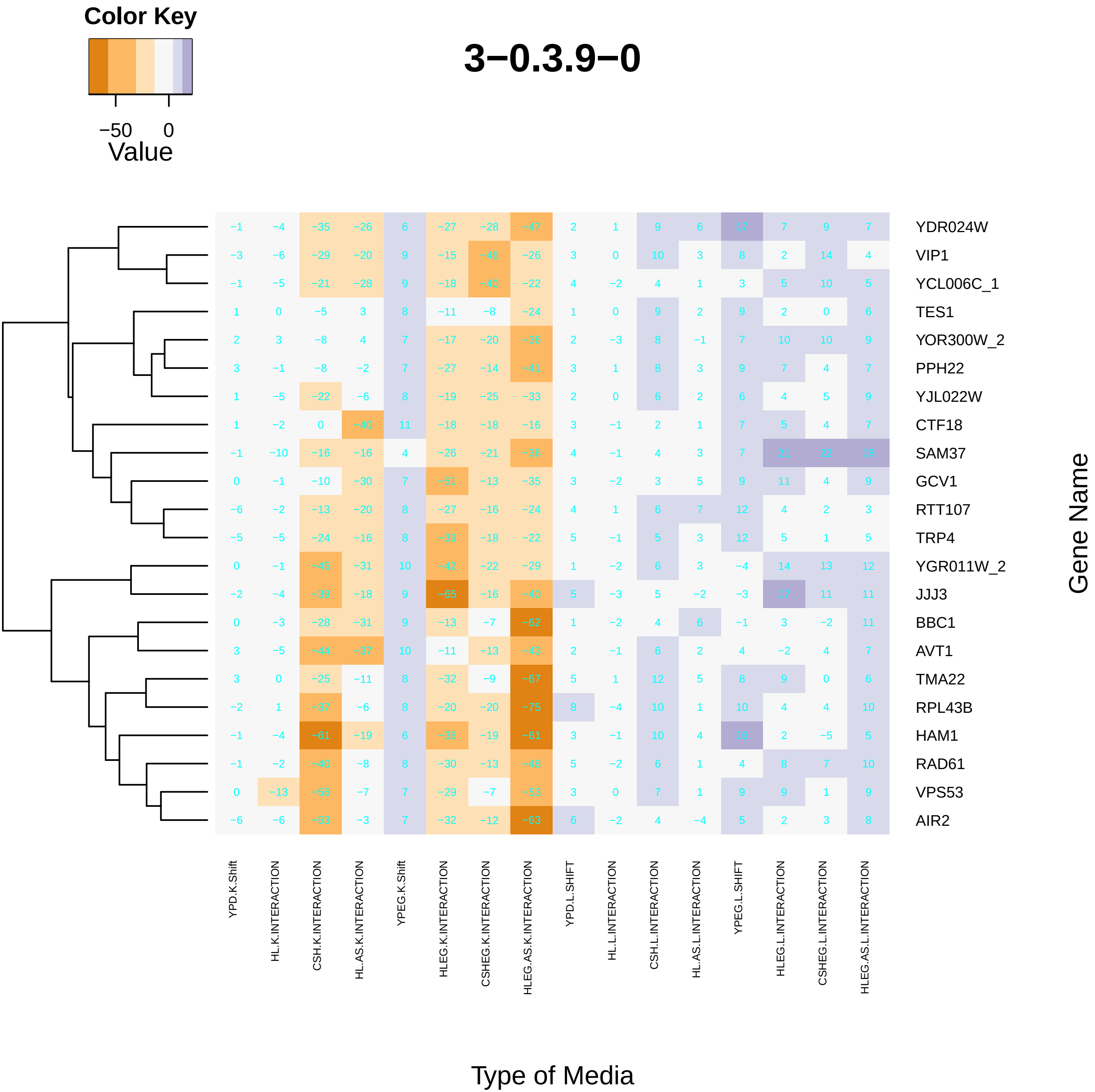

Color Key
−50
0
Value
3−0.3.9−0
YDR024W
VIP1
YCL006C_1
TES1
YOR300W_2
PPH22
YJL022W
CTF18
SAM37
GCV1
RTT107
TRP4
YGR011W_2
JJJ3
BBC1
AVT1
TMA22
RPL43B
HAM1
RAD61
VPS53
AIR2
Gene Name
YPD.K.Shift
HL.K.INTERACTION
CSH.K.INTERACTION
HL.AS.K.INTERACTION
YPEG.K.Shift
HLEG.K.INTERACTION
CSHEG.K.INTERACTION
HLEG.AS.K.INTERACTION
YPD.L.SHIFT
HL.L.INTERACTION
CSH.L.INTERACTION
HL.AS.L.INTERACTION
YPEG.L.SHIFT
HLEG.L.INTERACTION
CSHEG.L.INTERACTION
HLEG.AS.L.INTERACTION
Type of Media

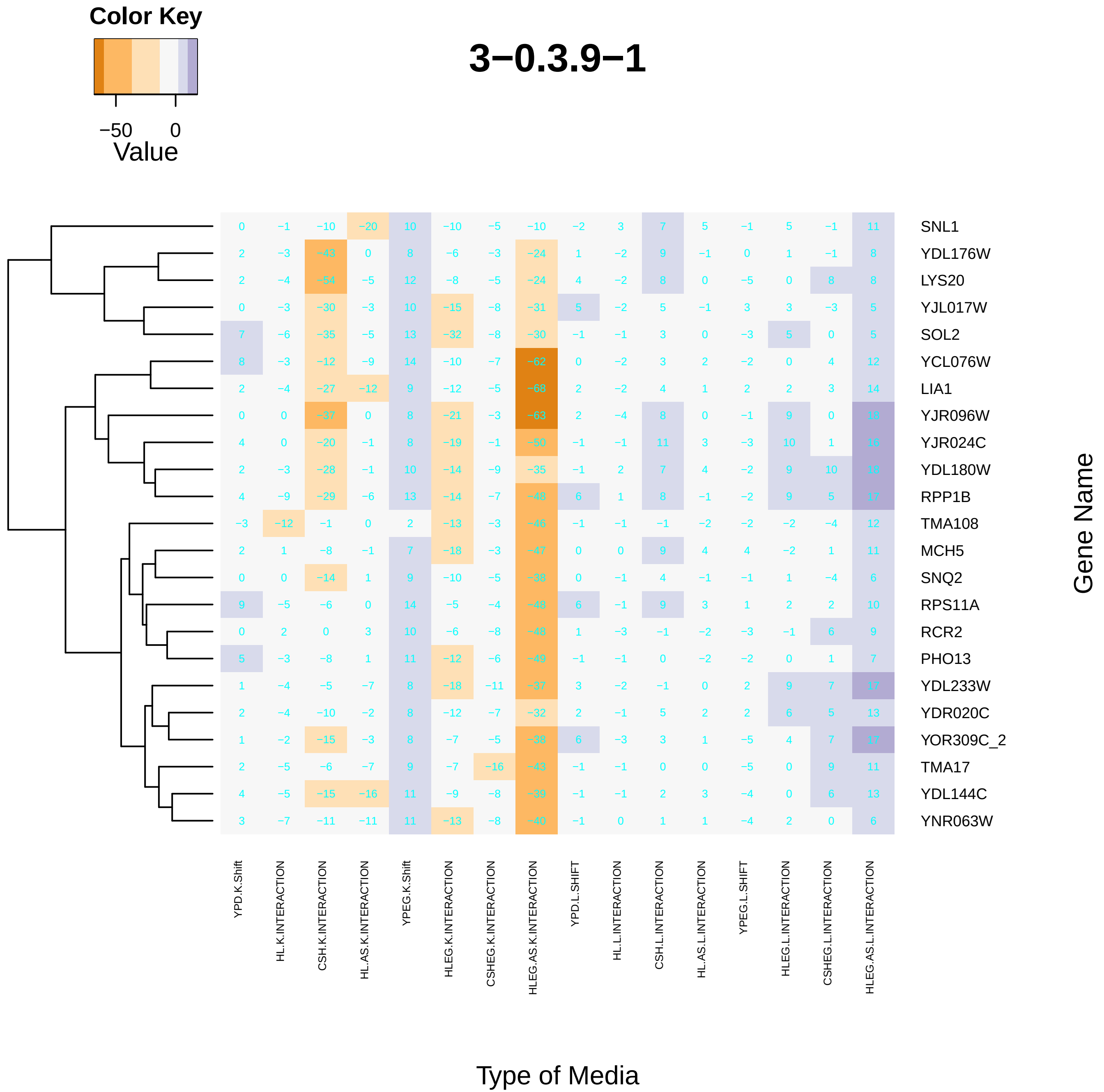
Color Key
−50
0
Value
3−0.3.9−1
SNL1
YDL176W
LYS20
YJL017W
SOL2
YCL076W
LIA1
YJR096W
YJR024C
YDL180W
RPP1B
TMA108
MCH5
SNQ2
RPS11A
RCR2
PHO13
YDL233W
YDR020C
YOR309C_2
TMA17
YDL144C
YNR063W
Gene Name
YPD.K.Shift
HL.K.INTERACTION
CSH.K.INTERACTION
HL.AS.K.INTERACTION
YPEG.K.Shift
HLEG.K.INTERACTION
CSHEG.K.INTERACTION
HLEG.AS.K.INTERACTION
YPD.L.SHIFT
HL.L.INTERACTION
CSH.L.INTERACTION
HL.AS.L.INTERACTION
YPEG.L.SHIFT
HLEG.L.INTERACTION
CSHEG.L.INTERACTION
HLEG.AS.L.INTERACTION
Type of Media

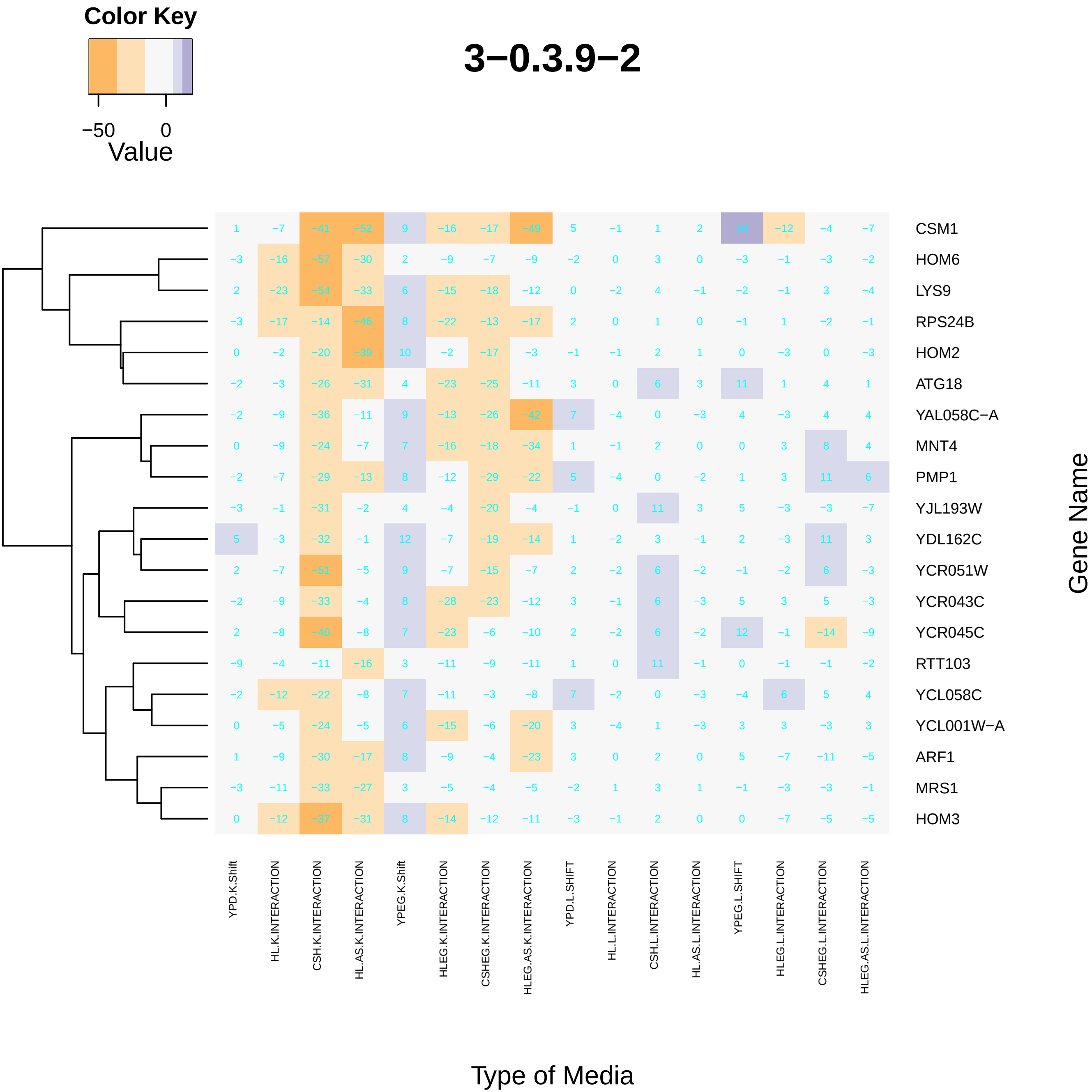
Color Key
−50 0
Value
3−0.3.9−2
CSM1: 1 −7 −41 −52 9 −16 −17 −49 5 −1 1 2 19 −12 −4 −7
HOM6: −3 −16 −57 −30 2 −9 −7 −9 −2 0 3 0 −3 −1 −3 −2
LYS9: 2 −23 −54 −33 6 −15 −18 −12 0 −2 4 −1 −2 −1 3 −4
RPS24B: −3 −17 −14 −46 8 −22 −13 −17 2 0 1 0 −1 1 −2 −1
HOM2: 0 −2 −20 −39 10 −2 −17 −3 −1 −1 2 1 0 −3 0 −3
ATG18: −2 −3 −26 −31 4 −23 −25 −11 3 0 6 3 11 1 4 1
YAL058C−A: −2 −9 −36 −11 9 −13 −26 −42 7 −4 0 −3 4 −3 4 4
MNT4: 0 −9 −24 −7 7 −16 −18 −34 1 −1 2 0 0 3 8 4
PMP1: −2 −7 −29 −13 8 −12 −29 −22 5 −4 0 −2 1 3 11 6
YJL193W: −3 −1 −31 −2 4 −4 −20 −4 −1 0 11 3 5 −3 −3 −7
YDL162C: 5 −3 −32 −1 12 −7 −19 −14 1 −2 3 −1 2 −3 11 3
YCR051W: 2 −7 −51 −5 9 −7 −15 −7 2 −2 6 −2 −1 −2 6 −3
YCR043C: −2 −9 −33 −4 8 −28 −23 −12 3 −1 6 −3 5 3 5 −3
YCR045C: 2 −8 −40 −8 7 −23 −6 −10 2 −2 6 −2 12 −1 −14 −9
RTT103: −9 −4 −11 −16 3 −11 −9 −11 1 0 11 −1 0 −1 −1 −2
YCL058C: −2 −12 −22 −8 7 −11 −3 −8 7 −2 0 −3 −4 6 5 4
YCL001W−A: 0 −5 −24 −5 6 −15 −6 −20 3 −4 1 −3 3 3 −3 3
ARF1: 1 −9 −30 −17 8 −9 −4 −23 3 0 2 0 5 −7 −11 −5
MRS1: −3 −11 −33 −27 3 −5 −4 −5 −2 1 3 1 −1 −3 −3 −1
HOM3: 0 −12 −37 −31 8 −14 −12 −11 −3 −1 2 0 0 −7 −5 −5
Gene Name
YPD.K.Shift
HL.K.INTERACTION
CSH.K.INTERACTION
HL.AS.K.INTERACTION
YPEG.K.Shift
HLEG.K.INTERACTION
CSHEG.K.INTERACTION
HLEG.AS.K.INTERACTION
YPD.L.SHIFT
HL.L.INTERACTION
CSH.L.INTERACTION
HL.AS.L.INTERACTION
YPEG.L.SHIFT
HLEG.L.INTERACTION
CSHEG.L.INTERACTION
HLEG.AS.L.INTERACTION
Type of Media

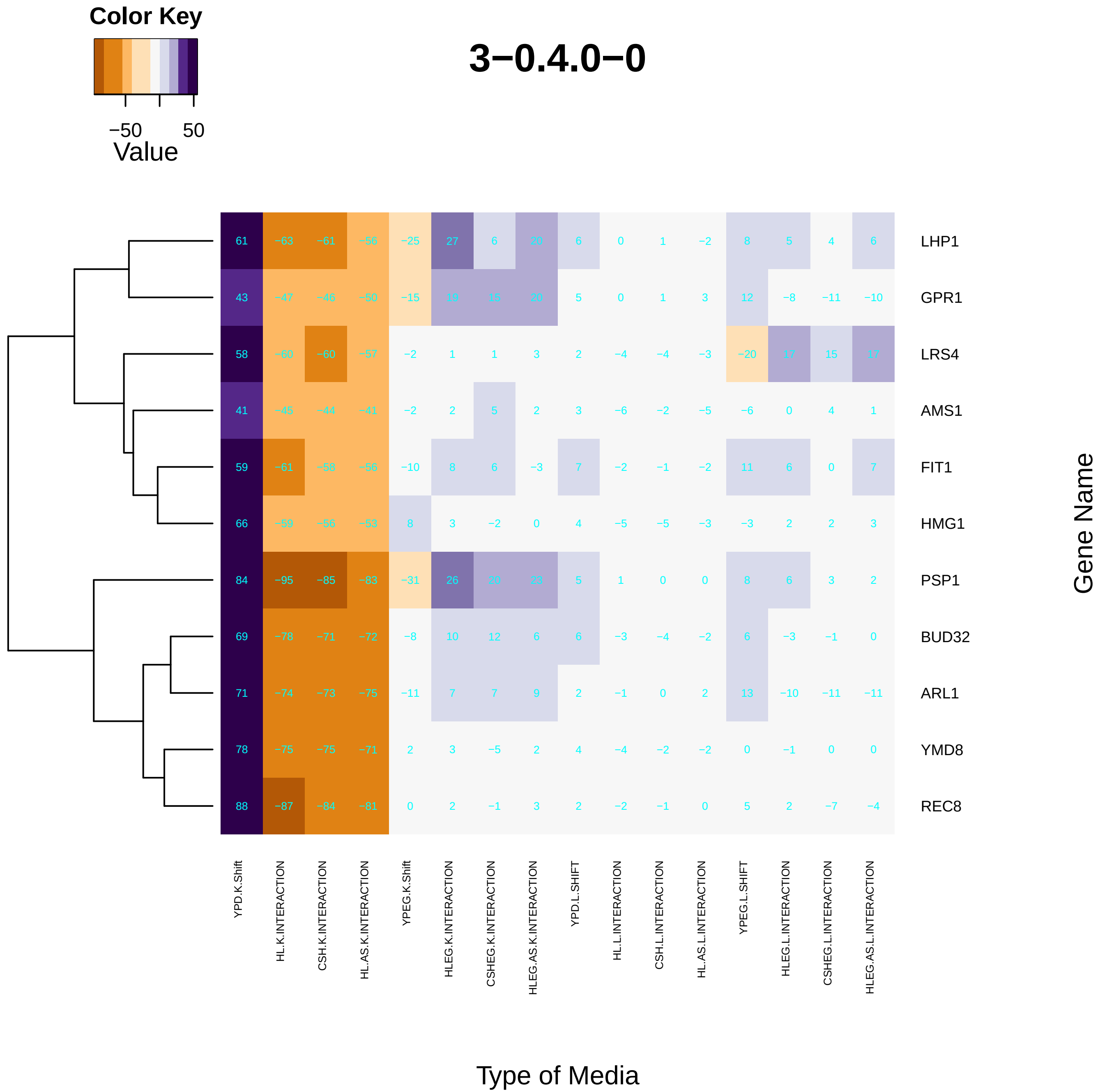
Color Key
−50 50
Value
3−0.4.0−0
YPD.K.Shift
HL.K.INTERACTION
CSH.K.INTERACTION
HL.AS.K.INTERACTION
YPEG.K.Shift
HLEG.K.INTERACTION
CSHEG.K.INTERACTION
HLEG.AS.K.INTERACTION
YPD.L.SHIFT
HL.L.INTERACTION
CSH.L.INTERACTION
HL.AS.L.INTERACTION
YPEG.L.SHIFT
HLEG.L.INTERACTION
CSHEG.L.INTERACTION
HLEG.AS.L.INTERACTION
LHP1 61 −63 −61 −56 −25 27 6 20 6 0 1 −2 8 5 4 6
GPR1 43 −47 −46 −50 −15 19 15 20 5 0 1 3 12 −8 −11 −10
LRS4 58 −60 −60 −57 −2 1 1 3 2 −4 −4 −3 −20 17 15 17
AMS1 41 −45 −44 −41 −2 2 5 2 3 −6 −2 −5 −6 0 4 1
FIT1 59 −61 −58 −56 −10 8 6 −3 7 −2 −1 −2 11 6 0 7
HMG1 66 −59 −56 −53 8 3 −2 0 4 −5 −5 −3 −3 2 2 3
PSP1 84 −95 −85 −83 −31 26 20 23 5 1 0 0 8 6 3 2
BUD32 69 −78 −71 −72 −8 10 12 6 6 −3 −4 −2 6 −3 −1 0
ARL1 71 −74 −73 −75 −11 7 7 9 2 −1 0 2 13 −10 −11 −11
YMD8 78 −75 −75 −71 2 3 −5 2 4 −4 −2 −2 0 −1 0 0
REC8 88 −87 −84 −81 0 2 −1 3 2 −2 −1 0 5 2 −7 −4
Gene Name
Type of Media

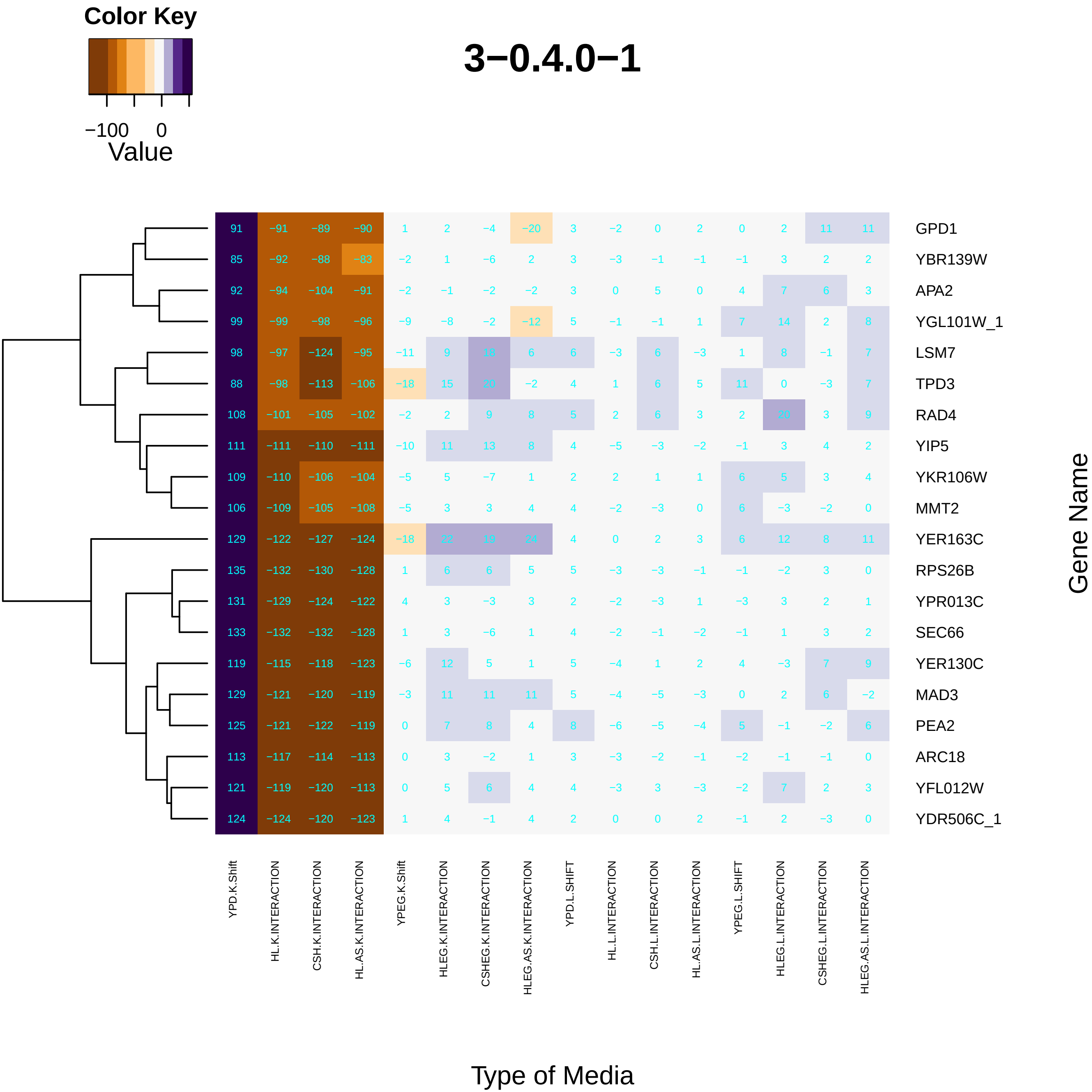
Color Key
−100 0
Value
3−0.4.0−1
YPD.K.Shift
HL.K.INTERACTION
CSH.K.INTERACTION
HL.AS.K.INTERACTION
YPEG.K.Shift
HLEG.K.INTERACTION
CSHEG.K.INTERACTION
HLEG.AS.K.INTERACTION
YPD.L.SHIFT
HL.L.INTERACTION
CSH.L.INTERACTION
HL.AS.L.INTERACTION
YPEG.L.SHIFT
HLEG.L.INTERACTION
CSHEG.L.INTERACTION
HLEG.AS.L.INTERACTION
GPD1 91 −91 −89 −90 1 2 −4 −20 3 −2 0 2 0 2 11 11
YBR139W 85 −92 −88 −83 −2 1 −6 2 3 −3 −1 −1 −1 3 2 2
APA2 92 −94 −104 −91 −2 −1 −2 −2 3 0 5 0 4 7 6 3
YGL101W_1 99 −99 −98 −96 −9 −8 −2 −12 5 −1 −1 1 7 14 2 8
LSM7 98 −97 −124 −95 −11 9 18 6 6 −3 6 −3 1 8 −1 7
TPD3 88 −98 −113 −106 −18 15 20 −2 4 1 6 5 11 0 −3 7
RAD4 108 −101 −105 −102 −2 2 9 8 5 2 6 3 2 20 3 9
YIP5 111 −111 −110 −111 −10 11 13 8 4 −5 −3 −2 −1 3 4 2
YKR106W 109 −110 −106 −104 −5 5 −7 1 2 2 1 1 6 5 3 4
MMT2 106 −109 −105 −108 −5 3 3 4 4 −2 −3 0 6 −3 −2 0
YER163C 129 −122 −127 −124 −18 22 19 24 4 0 2 3 6 12 8 11
RPS26B 135 −132 −130 −128 1 6 6 5 5 −3 −3 −1 −1 −2 3 0
YPR013C 131 −129 −124 −122 4 3 −3 3 2 −2 −3 1 −3 3 2 1
SEC66 133 −132 −132 −128 1 3 −6 1 4 −2 −1 −2 −1 1 3 2
YER130C 119 −115 −118 −123 −6 12 5 1 5 −4 1 2 4 −3 7 9
MAD3 129 −121 −120 −119 −3 11 11 11 5 −4 −5 −3 0 2 6 −2
PEA2 125 −121 −122 −119 0 7 8 4 8 −6 −5 −4 5 −1 −2 6
ARC18 113 −117 −114 −113 0 3 −2 1 3 −3 −2 −1 −2 −1 −1 0
YFL012W 121 −119 −120 −113 0 5 6 4 4 −3 3 −3 −2 7 2 3
YDR506C_1 124 −124 −120 −123 1 4 −1 4 2 0 0 2 −1 2 −3 0
Gene Name
Type of Media

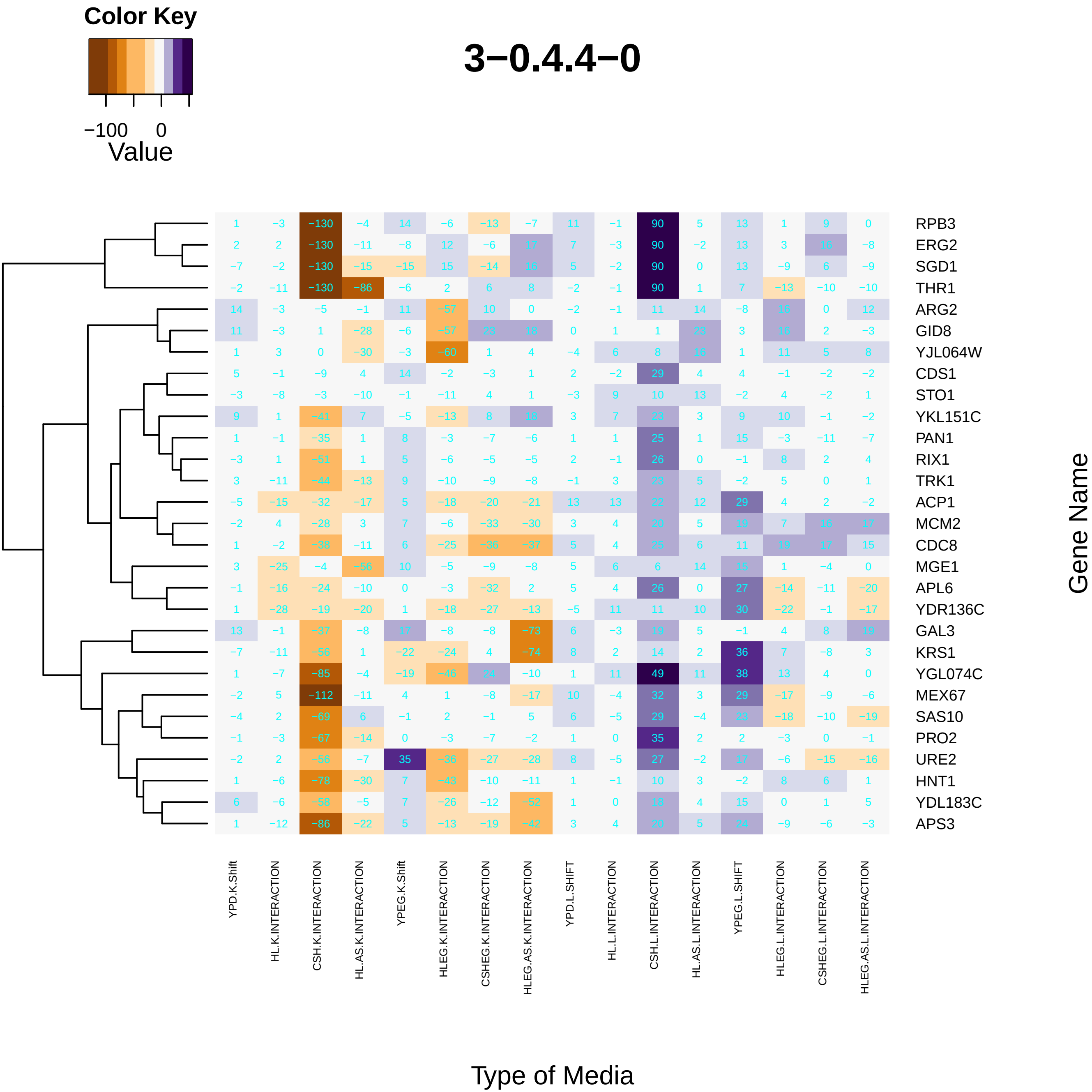
Color Key
−100
0
Value
3−0.4.4−0
RPB3
ERG2
SGD1
THR1
ARG2
GID8
YJL064W
CDS1
STO1
YKL151C
PAN1
RIX1
TRK1
ACP1
MCM2
CDC8
MGE1
APL6
YDR136C
GAL3
KRS1
YGL074C
MEX67
SAS10
PRO2
URE2
HNT1
YDL183C
APS3
Gene Name
YPD.K.Shift
HL.K.INTERACTION
CSH.K.INTERACTION
HL.AS.K.INTERACTION
YPEG.K.Shift
HLEG.K.INTERACTION
CSHEG.K.INTERACTION
HLEG.AS.K.INTERACTION
YPD.L.SHIFT
HL.L.INTERACTION
CSH.L.INTERACTION
HL.AS.L.INTERACTION
YPEG.L.SHIFT
HLEG.L.INTERACTION
CSHEG.L.INTERACTION
HLEG.AS.L.INTERACTION
Type of Media

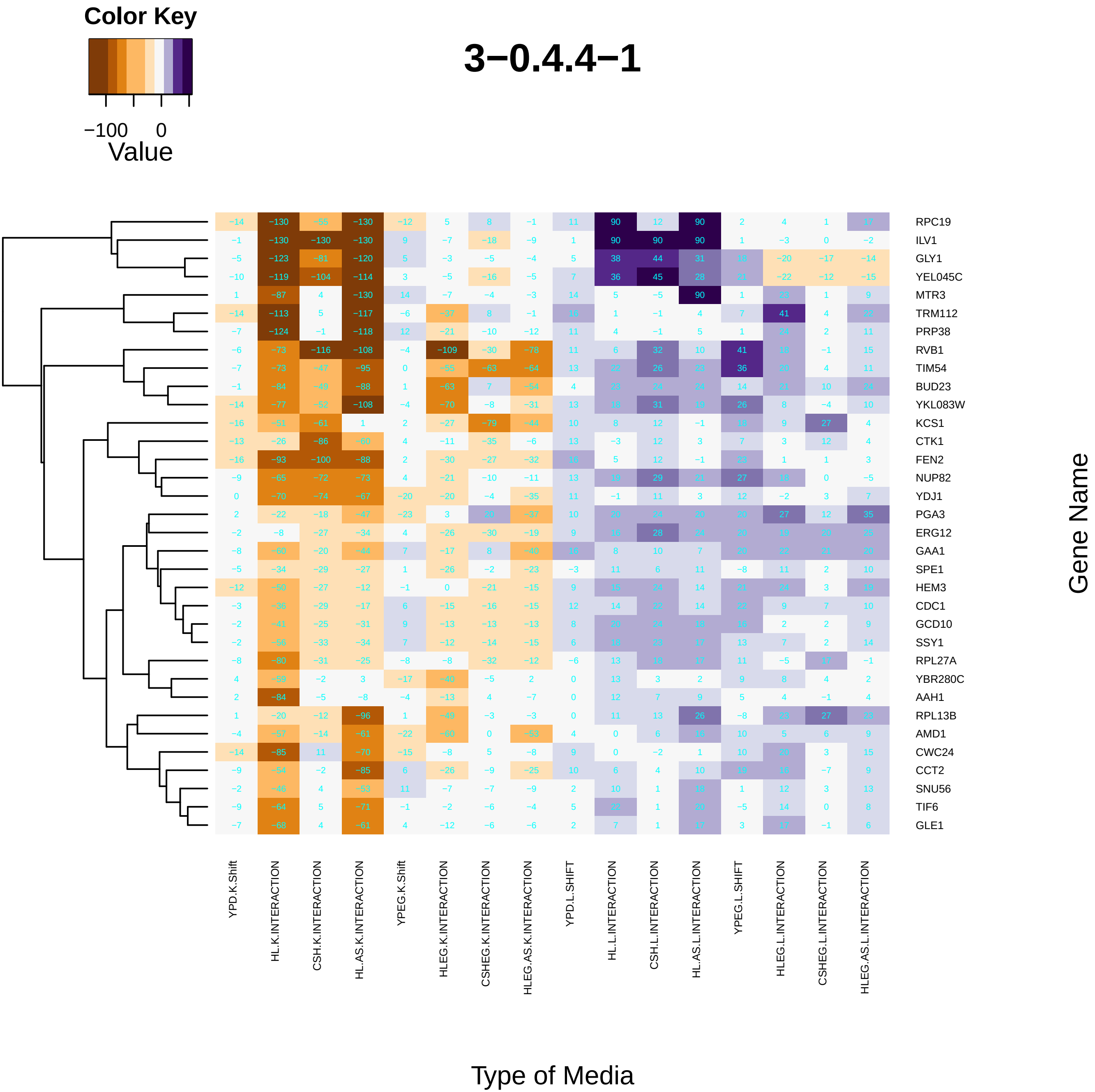
Color Key
−100
0
Value
3−0.4.4−1
RPC19
ILV1
GLY1
YEL045C
MTR3
TRM112
PRP38
RVB1
TIM54
BUD23
YKL083W
KCS1
CTK1
FEN2
NUP82
YDJ1
PGA3
ERG12
GAA1
SPE1
HEM3
CDC1
GCD10
SSY1
RPL27A
YBR280C
AAH1
RPL13B
AMD1
CWC24
CCT2
SNU56
TIF6
GLE1
Gene Name
YPD.K.Shift
HL.K.INTERACTION
CSH.K.INTERACTION
HL.AS.K.INTERACTION
YPEG.K.Shift
HLEG.K.INTERACTION
CSHEG.K.INTERACTION
HLEG.AS.K.INTERACTION
YPD.L.SHIFT
HL.L.INTERACTION
CSH.L.INTERACTION
HL.AS.L.INTERACTION
YPEG.L.SHIFT
HLEG.L.INTERACTION
CSHEG.L.INTERACTION
HLEG.AS.L.INTERACTION
Type of Media

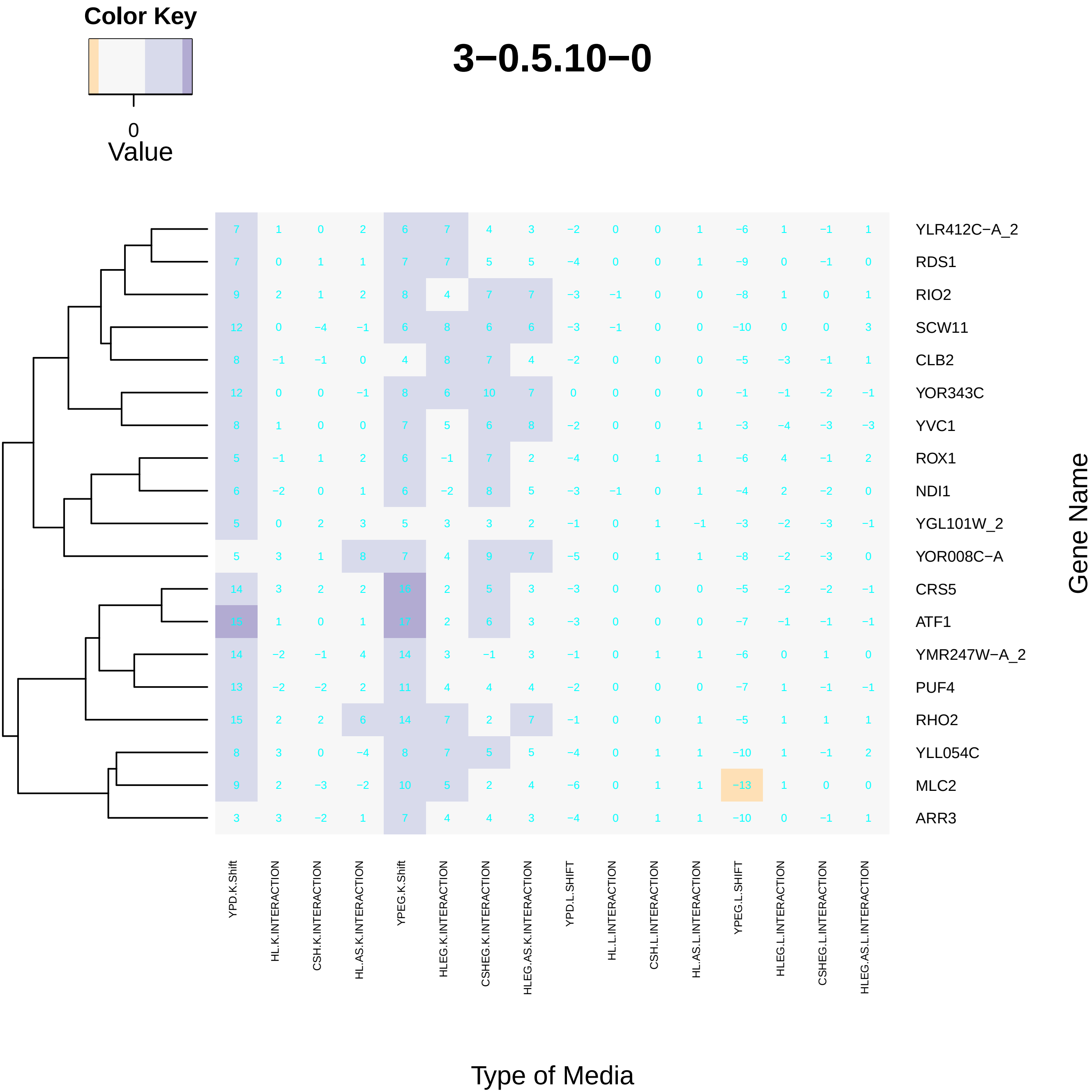
Color Key
0
Value
3−0.5.10−0
YLR412C−A_2 7 1 0 2 6 7 4 3 −2 0 0 1 −6 1 −1 1
RDS1 7 0 1 1 7 7 5 5 −4 0 0 1 −9 0 −1 0
RIO2 9 2 1 2 8 4 7 7 −3 −1 0 0 −8 1 0 1
SCW11 12 0 −4 −1 6 8 6 6 −3 −1 0 0 −10 0 0 3
CLB2 8 −1 −1 0 4 8 7 4 −2 0 0 0 −5 −3 −1 1
YOR343C 12 0 0 −1 8 6 10 7 0 0 0 0 −1 −1 −2 −1
YVC1 8 1 0 0 7 5 6 8 −2 0 0 1 −3 −4 −3 −3
ROX1 5 −1 1 2 6 −1 7 2 −4 0 1 1 −6 4 −1 2
NDI1 6 −2 0 1 6 −2 8 5 −3 −1 0 1 −4 2 −2 0
YGL101W_2 5 0 2 3 5 3 3 2 −1 0 1 −1 −3 −2 −3 −1
YOR008C−A 5 3 1 8 7 4 9 7 −5 0 1 1 −8 −2 −3 0
CRS5 14 3 2 2 16 2 5 3 −3 0 0 0 −5 −2 −2 −1
ATF1 15 1 0 1 17 2 6 3 −3 0 0 0 −7 −1 −1 −1
YMR247W−A_2 14 −2 −1 4 14 3 −1 3 −1 0 1 1 −6 0 1 0
PUF4 13 −2 −2 2 11 4 4 4 −2 0 0 0 −7 1 −1 −1
RHO2 15 2 2 6 14 7 2 7 −1 0 0 1 −5 1 1 1
YLL054C 8 3 0 −4 8 7 5 5 −4 0 1 1 −10 1 −1 2
MLC2 9 2 −3 −2 10 5 2 4 −6 0 1 1 −13 1 0 0
ARR3 3 3 −2 1 7 4 4 3 −4 0 1 1 −10 0 −1 1
YPD.K.Shift
HL.K.INTERACTION
CSH.K.INTERACTION
HL.AS.K.INTERACTION
YPEG.K.Shift
HLEG.K.INTERACTION
CSHEG.K.INTERACTION
HLEG.AS.K.INTERACTION
YPD.L.SHIFT
HL.L.INTERACTION
CSH.L.INTERACTION
HL.AS.L.INTERACTION
YPEG.L.SHIFT
HLEG.L.INTERACTION
CSHEG.L.INTERACTION
HLEG.AS.L.INTERACTION
Gene Name
Type of Media

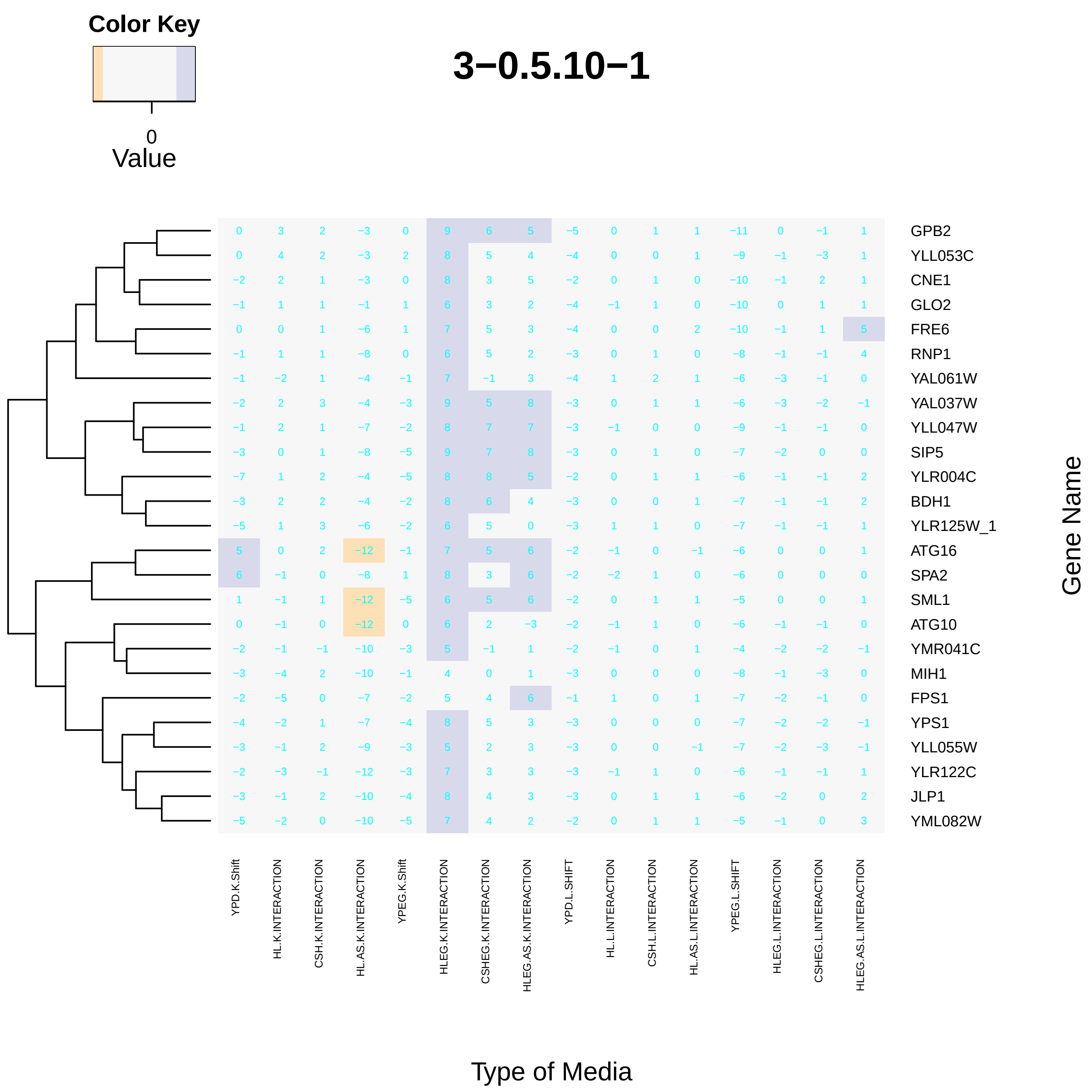
3−0.5.10−1
Color Key
0
Value
Gene Name
Type of Media
YPD.K.Shift HL.K.INTERACTION CSH.K.INTERACTION HL.AS.K.INTERACTION YPEG.K.Shift HLEG.K.INTERACTION CSHEG.K.INTERACTION HLEG.AS.K.INTERACTION YPD.L.SHIFT HL.L.INTERACTION CSH.L.INTERACTION HL.AS.L.INTERACTION YPEG.L.SHIFT HLEG.L.INTERACTION CSHEG.L.INTERACTION HLEG.AS.L.INTERACTION
GPB2 0 3 2 −3 0 9 6 5 −5 0 1 1 −11 0 −1 1
YLL053C 0 4 2 −3 2 8 5 4 −4 0 0 1 −9 −1 −3 1
CNE1 −2 2 1 −3 0 8 3 5 −2 0 1 0 −10 −1 2 1
GLO2 −1 1 1 −1 1 6 3 2 −4 −1 1 0 −10 0 1 1
FRE6 0 0 1 −6 1 7 5 3 −4 0 0 2 −10 −1 1 5
RNP1 −1 1 1 −8 0 6 5 2 −3 0 1 0 −8 −1 −1 4
YAL061W −1 −2 1 −4 −1 7 −1 3 −4 1 2 1 −6 −3 −1 0
YAL037W −2 2 3 −4 −3 9 5 8 −3 0 1 1 −6 −3 −2 −1
YLL047W −1 2 1 −7 −2 8 7 7 −3 −1 0 0 −9 −1 −1 0
SIP5 −3 0 1 −8 −5 9 7 8 −3 0 1 0 −7 −2 0 0
YLR004C −7 1 2 −4 −5 8 8 5 −2 0 1 1 −6 −1 −1 2
BDH1 −3 2 2 −4 −2 8 6 4 −3 0 0 1 −7 −1 −1 2
YLR125W_1 −5 1 3 −6 −2 6 5 0 −3 1 1 0 −7 −1 −1 1
ATG16 5 0 2 −12 −1 7 5 6 −2 −1 0 −1 −6 0 0 1
SPA2 6 −1 0 −8 1 8 3 6 −2 −2 1 0 −6 0 0 0
SML1 1 −1 1 −12 −5 6 5 6 −2 0 1 1 −5 0 0 1
ATG10 0 −1 0 −12 0 6 2 −3 −2 −1 1 0 −6 −1 −1 0
YMR041C −2 −1 −1 −10 −3 5 −1 1 −2 −1 0 1 −4 −2 −2 −1
MIH1 −3 −4 2 −10 −1 4 0 1 −3 0 0 0 −8 −1 −3 0
FPS1 −2 −5 0 −7 −2 5 4 6 −1 1 0 1 −7 −2 −1 0
YPS1 −4 −2 1 −7 −4 8 5 3 −3 0 0 0 −7 −2 −2 −1
YLL055W −3 −1 2 −9 −3 5 2 3 −3 0 0 −1 −7 −2 −3 −1
YLR122C −2 −3 −1 −12 −3 7 3 3 −3 −1 1 0 −6 −1 −1 1
JLP1 −3 −1 2 −10 −4 8 4 3 −3 0 1 1 −6 −2 0 2
YML082W −5 −2 0 −10 −5 7 4 2 −2 0 1 1 −5 −1 0 3

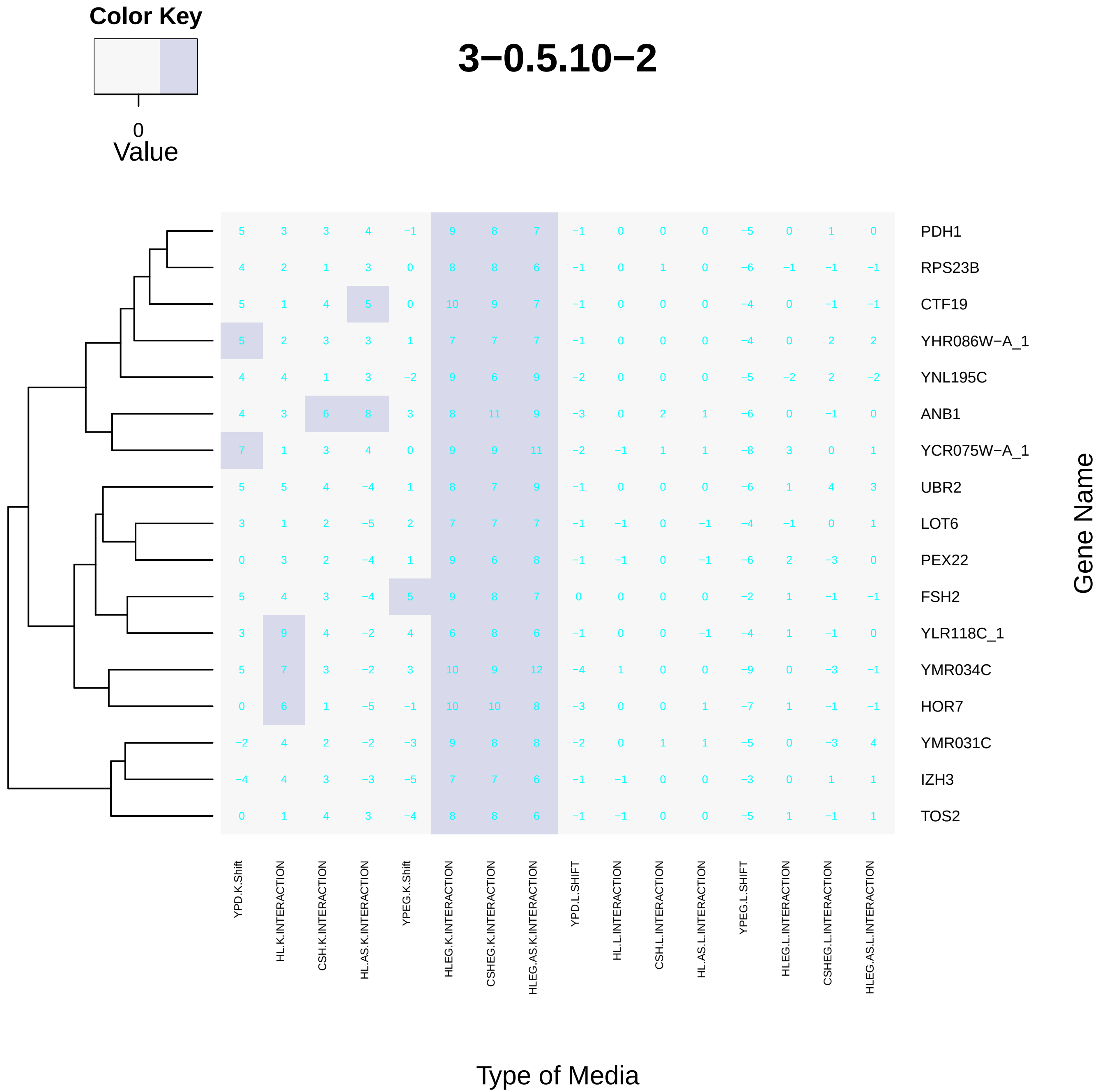
Color Key
0
Value
3−0.5.10−2
YPD.K.Shift
HL.K.INTERACTION
CSH.K.INTERACTION
HL.AS.K.INTERACTION
YPEG.K.Shift
HLEG.K.INTERACTION
CSHEG.K.INTERACTION
HLEG.AS.K.INTERACTION
YPD.L.SHIFT
HL.L.INTERACTION
CSH.L.INTERACTION
HL.AS.L.INTERACTION
YPEG.L.SHIFT
HLEG.L.INTERACTION
CSHEG.L.INTERACTION
HLEG.AS.L.INTERACTION
PDH1 5 3 3 4 −1 9 8 7 −1 0 0 0 −5 0 1 0
RPS23B 4 2 1 3 0 8 8 6 −1 0 1 0 −6 −1 −1 −1
CTF19 5 1 4 5 0 10 9 7 −1 0 0 0 −4 0 −1 −1
YHR086W−A_1 5 2 3 3 1 7 7 7 −1 0 0 0 −4 0 2 2
YNL195C 4 4 1 3 −2 9 6 9 −2 0 0 0 −5 −2 2 −2
ANB1 4 3 6 8 3 8 11 9 −3 0 2 1 −6 0 −1 0
YCR075W−A_1 7 1 3 4 0 9 9 11 −2 −1 1 1 −8 3 0 1
UBR2 5 5 4 −4 1 8 7 9 −1 0 0 0 −6 1 4 3
LOT6 3 1 2 −5 2 7 7 7 −1 −1 0 −1 −4 −1 0 1
PEX22 0 3 2 −4 1 9 6 8 −1 −1 0 −1 −6 2 −3 0
FSH2 5 4 3 −4 5 9 8 7 0 0 0 0 −2 1 −1 −1
YLR118C_1 3 9 4 −2 4 6 8 6 −1 0 0 −1 −4 1 −1 0
YMR034C 5 7 3 −2 3 10 9 12 −4 1 0 0 −9 0 −3 −1
HOR7 0 6 1 −5 −1 10 10 8 −3 0 0 1 −7 1 −1 −1
YMR031C −2 4 2 −2 −3 9 8 8 −2 0 1 1 −5 0 −3 4
IZH3 −4 4 3 −3 −5 7 7 6 −1 −1 0 0 −3 0 1 1
TOS2 0 1 4 3 −4 8 8 6 −1 −1 0 0 −5 1 −1 1
Gene Name
Type of Media

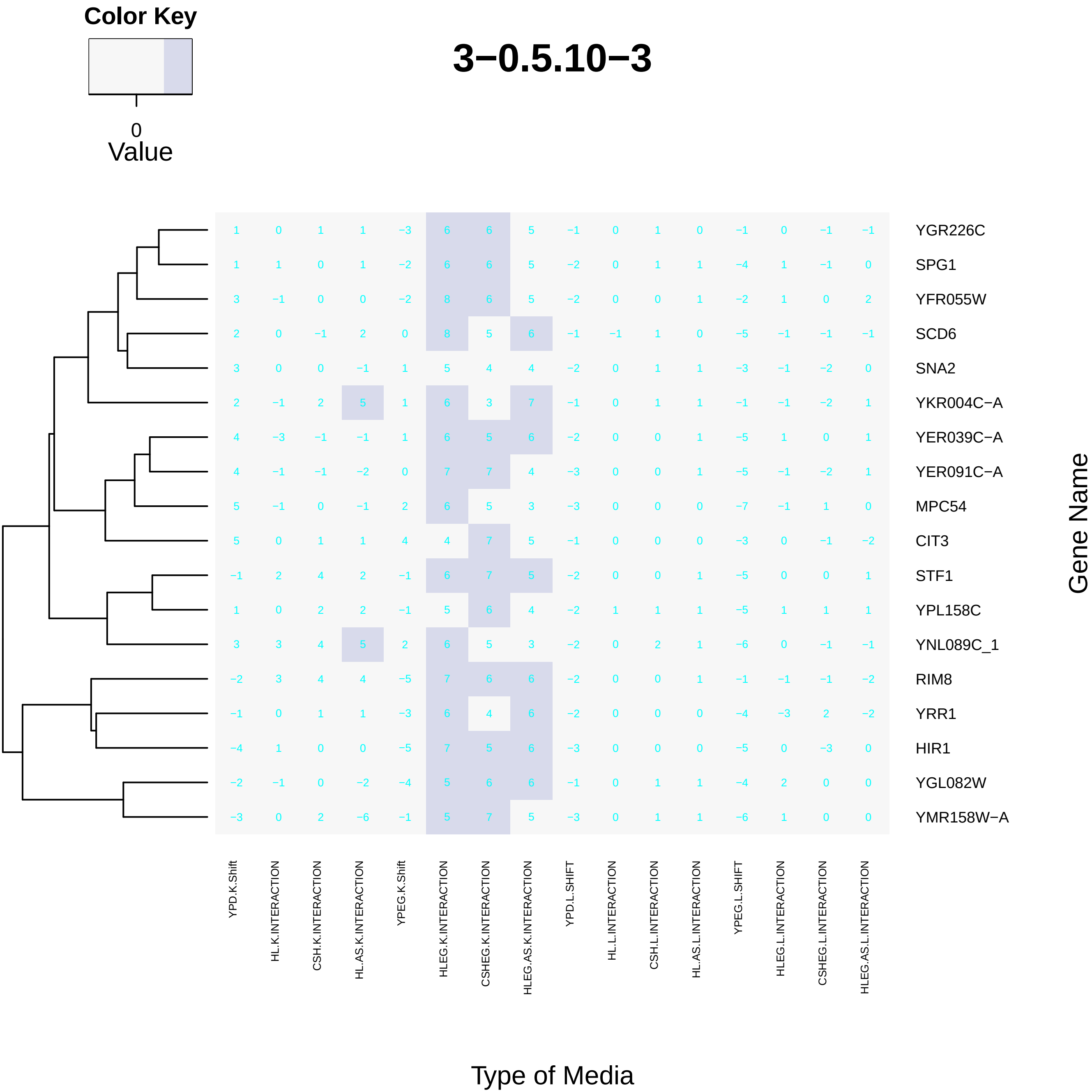
3−0.5.10−3
Color Key
0
Value
Gene Name
Type of Media
YPD.K.Shift
HL.K.INTERACTION
CSH.K.INTERACTION
HL.AS.K.INTERACTION
YPEG.K.Shift
HLEG.K.INTERACTION
CSHEG.K.INTERACTION
HLEG.AS.K.INTERACTION
YPD.L.SHIFT
HL.L.INTERACTION
CSH.L.INTERACTION
HL.AS.L.INTERACTION
YPEG.L.SHIFT
HLEG.L.INTERACTION
CSHEG.L.INTERACTION
HLEG.AS.L.INTERACTION
YGR226C 1 0 1 1 −3 6 6 5 −1 0 1 0 −1 0 −1 −1
SPG1 1 1 0 1 −2 6 6 5 −2 0 1 1 −4 1 −1 0
YFR055W 3 −1 0 0 −2 8 6 5 −2 0 0 1 −2 1 0 2
SCD6 2 0 −1 2 0 8 5 6 −1 −1 1 0 −5 −1 −1 −1
SNA2 3 0 0 −1 1 5 4 4 −2 0 1 1 −3 −1 −2 0
YKR004C−A 2 −1 2 5 1 6 3 7 −1 0 1 1 −1 −1 −2 1
YER039C−A 4 −3 −1 −1 1 6 5 6 −2 0 0 1 −5 1 0 1
YER091C−A 4 −1 −1 −2 0 7 7 4 −3 0 0 1 −5 −1 −2 1
MPC54 5 −1 0 −1 2 6 5 3 −3 0 0 0 −7 −1 1 0
CIT3 5 0 1 1 4 4 7 5 −1 0 0 0 −3 0 −1 −2
STF1 −1 2 4 2 −1 6 7 5 −2 0 0 1 −5 0 0 1
YPL158C 1 0 2 2 −1 5 6 4 −2 1 1 1 −5 1 1 1
YNL089C_1 3 3 4 5 2 6 5 3 −2 0 2 1 −6 0 −1 −1
RIM8 −2 3 4 4 −5 7 6 6 −2 0 0 1 −1 −1 −1 −2
YRR1 −1 0 1 1 −3 6 4 6 −2 0 0 0 −4 −3 2 −2
HIR1 −4 1 0 0 −5 7 5 6 −3 0 0 0 −5 0 −3 0
YGL082W −2 −1 0 −2 −4 5 6 6 −1 0 1 1 −4 2 0 0
YMR158W−A −3 0 2 −6 −1 5 7 5 −3 0 1 1 −6 1 0 0

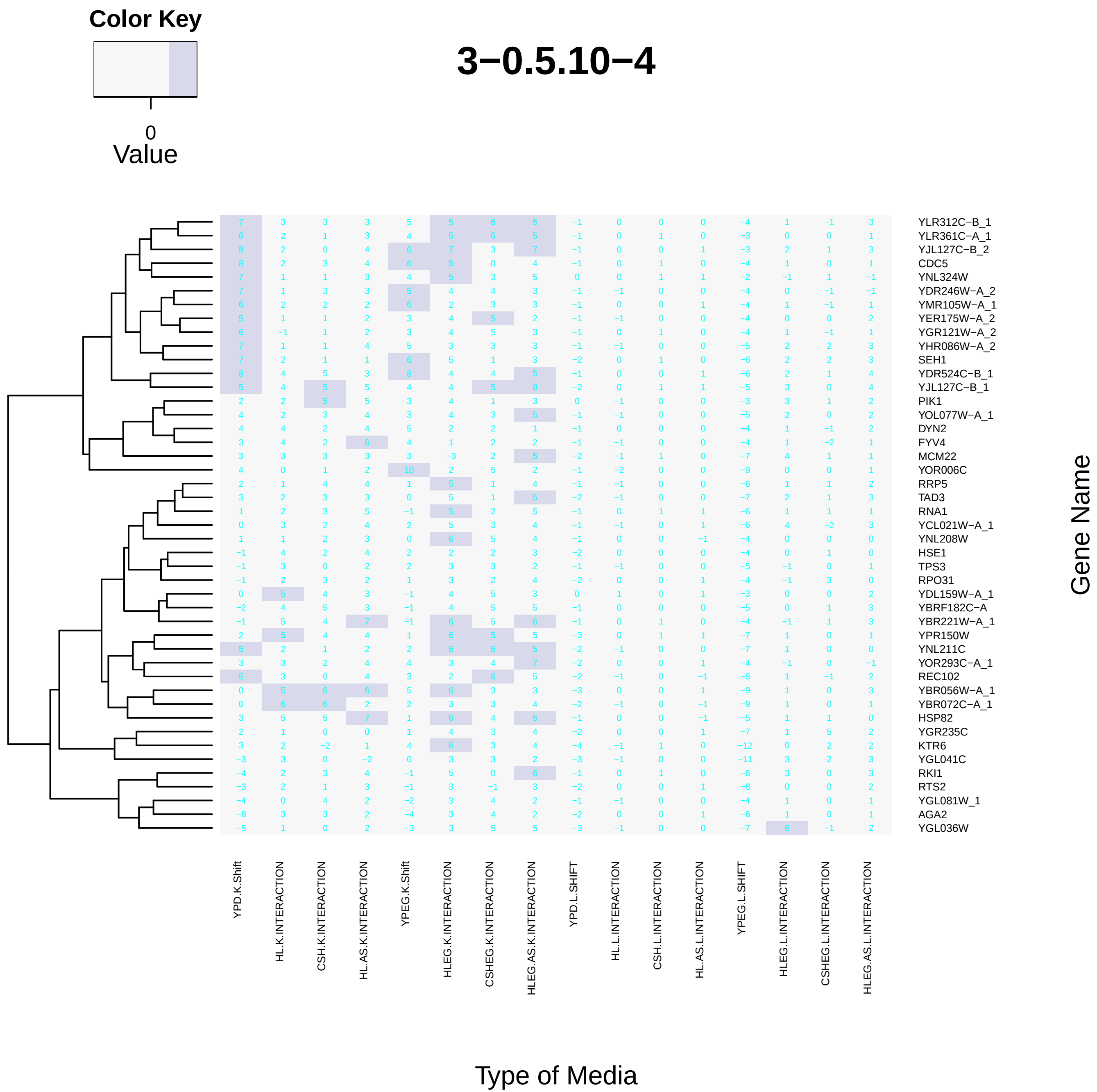
Color Key
0
Value
3−0.5.10−4
YLR312C−B_1
YLR361C−A_1
YJL127C−B_2
CDC5
YNL324W
YDR246W−A_2
YMR105W−A_1
YER175W−A_2
YGR121W−A_2
YHR086W−A_2
SEH1
YDR524C−B_1
YJL127C−B_1
PIK1
YOL077W−A_1
DYN2
FYV4
MCM22
YOR006C
RRP5
TAD3
RNA1
YCL021W−A_1
YNL208W
HSE1
TPS3
RPO31
YDL159W−A_1
YBRF182C−A
YBR221W−A_1
YPR150W
YNL211C
YOR293C−A_1
REC102
YBR056W−A_1
YBR072C−A_1
HSP82
YGR235C
KTR6
YGL041C
RKI1
RTS2
YGL081W_1
AGA2
YGL036W
Gene Name
YPD.K.Shift
HL.K.INTERACTION
CSH.K.INTERACTION
HL.AS.K.INTERACTION
YPEG.K.Shift
HLEG.K.INTERACTION
CSHEG.K.INTERACTION
HLEG.AS.K.INTERACTION
YPD.L.SHIFT
HL.L.INTERACTION
CSH.L.INTERACTION
HL.AS.L.INTERACTION
YPEG.L.SHIFT
HLEG.L.INTERACTION
CSHEG.L.INTERACTION
HLEG.AS.L.INTERACTION
Type of Media

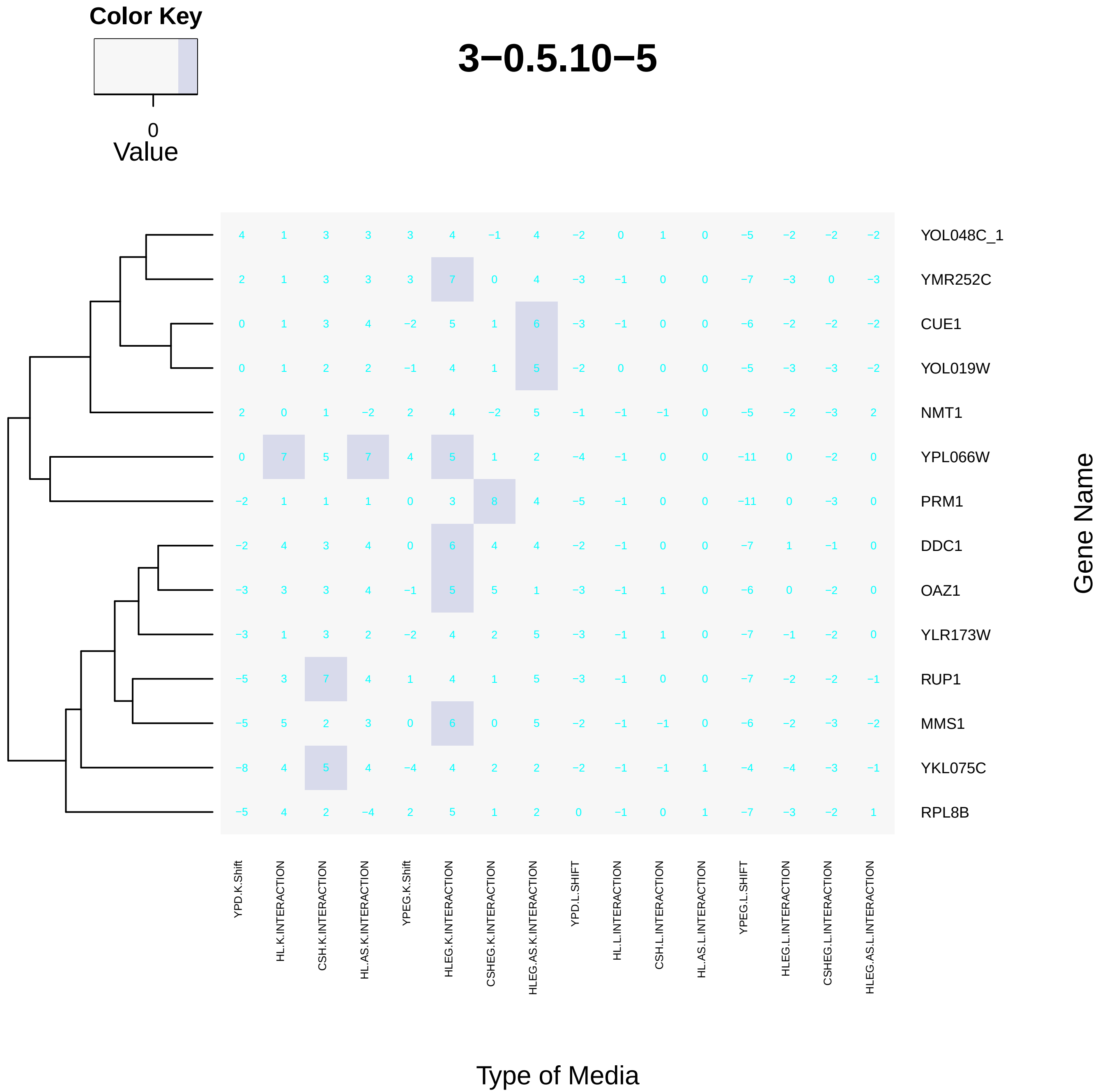
Color Key
0
Value
3−0.5.10−5
YOL048C_1 4 1 3 3 3 4 −1 4 −2 0 1 0 −5 −2 −2 −2
YMR252C 2 1 3 3 3 7 0 4 −3 −1 0 0 −7 −3 0 −3
CUE1 0 1 3 4 −2 5 1 6 −3 −1 0 0 −6 −2 −2 −2
YOL019W 0 1 2 2 −1 4 1 5 −2 0 0 0 −5 −3 −3 −2
NMT1 2 0 1 −2 2 4 −2 5 −1 −1 −1 0 −5 −2 −3 2
YPL066W 0 7 5 7 4 5 1 2 −4 −1 0 0 −11 0 −2 0
PRM1 −2 1 1 1 0 3 8 4 −5 −1 0 0 −11 0 −3 0
DDC1 −2 4 3 4 0 6 4 4 −2 −1 0 0 −7 1 −1 0
OAZ1 −3 3 3 4 −1 5 5 1 −3 −1 1 0 −6 0 −2 0
YLR173W −3 1 3 2 −2 4 2 5 −3 −1 1 0 −7 −1 −2 0
RUP1 −5 3 7 4 1 4 1 5 −3 −1 0 0 −7 −2 −2 −1
MMS1 −5 5 2 3 0 6 0 5 −2 −1 −1 0 −6 −2 −3 −2
YKL075C −8 4 5 4 −4 4 2 2 −2 −1 −1 1 −4 −4 −3 −1
RPL8B −5 4 2 −4 2 5 1 2 0 −1 0 1 −7 −3 −2 1
Gene Name
YPD.K.Shift
HL.K.INTERACTION
CSH.K.INTERACTION
HL.AS.K.INTERACTION
YPEG.K.Shift
HLEG.K.INTERACTION
CSHEG.K.INTERACTION
HLEG.AS.K.INTERACTION
YPD.L.SHIFT
HL.L.INTERACTION
CSH.L.INTERACTION
HL.AS.L.INTERACTION
YPEG.L.SHIFT
HLEG.L.INTERACTION
CSHEG.L.INTERACTION
HLEG.AS.L.INTERACTION
Type of Media

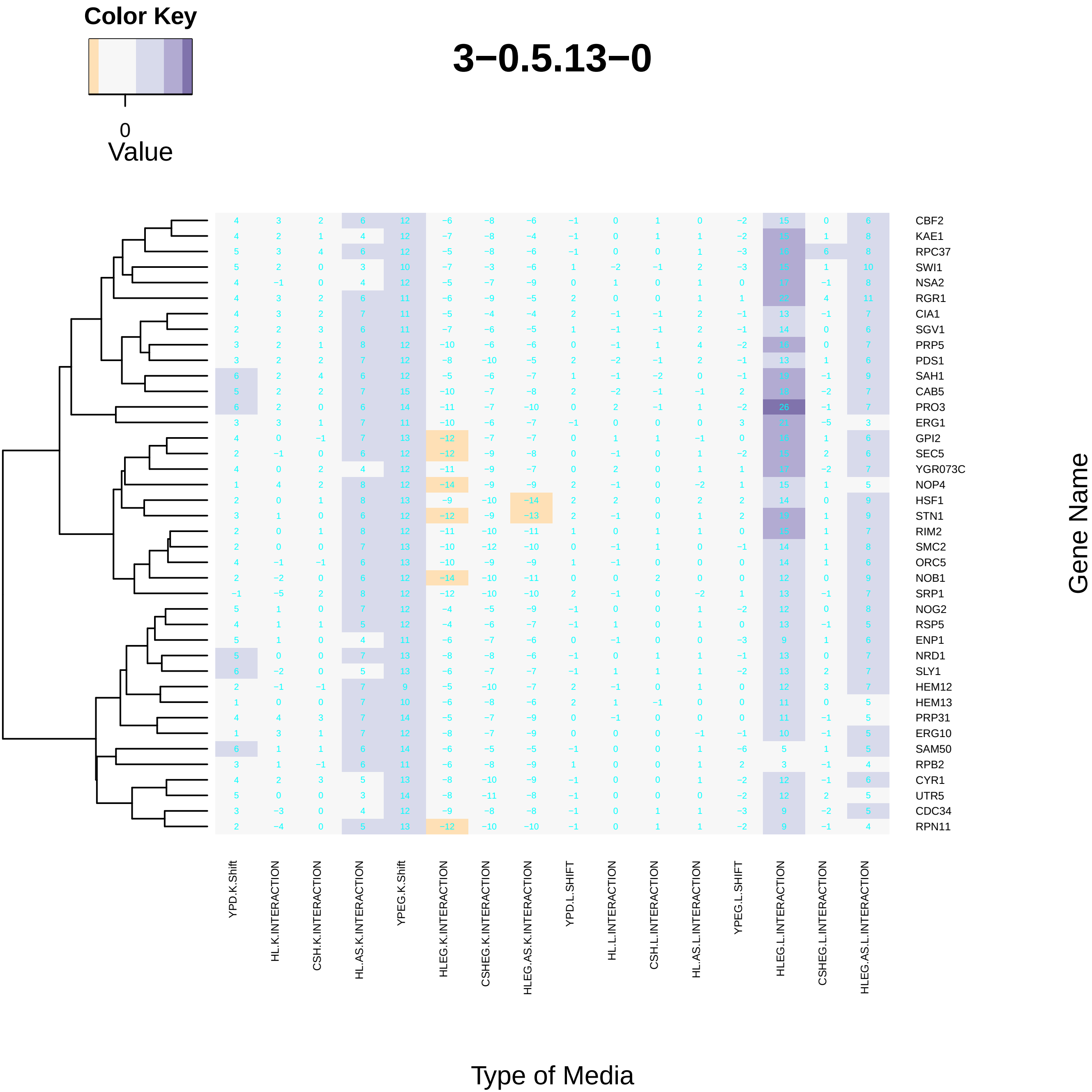
Color Key
0
Value
3−0.5.13−0
CBF2
KAE1
RPC37
SWI1
NSA2
RGR1
CIA1
SGV1
PRP5
PDS1
SAH1
CAB5
PRO3
ERG1
GPI2
SEC5
YGR073C
NOP4
HSF1
STN1
RIM2
SMC2
ORC5
NOB1
SRP1
NOG2
RSP5
ENP1
NRD1
SLY1
HEM12
HEM13
PRP31
ERG10
SAM50
RPB2
CYR1
UTR5
CDC34
RPN11
Gene Name
YPD.K.Shift
HL.K.INTERACTION
CSH.K.INTERACTION
HL.AS.K.INTERACTION
YPEG.K.Shift
HLEG.K.INTERACTION
CSHEG.K.INTERACTION
HLEG.AS.K.INTERACTION
YPD.L.SHIFT
HL.L.INTERACTION
CSH.L.INTERACTION
HL.AS.L.INTERACTION
YPEG.L.SHIFT
HLEG.L.INTERACTION
CSHEG.L.INTERACTION
HLEG.AS.L.INTERACTION
Type of Media

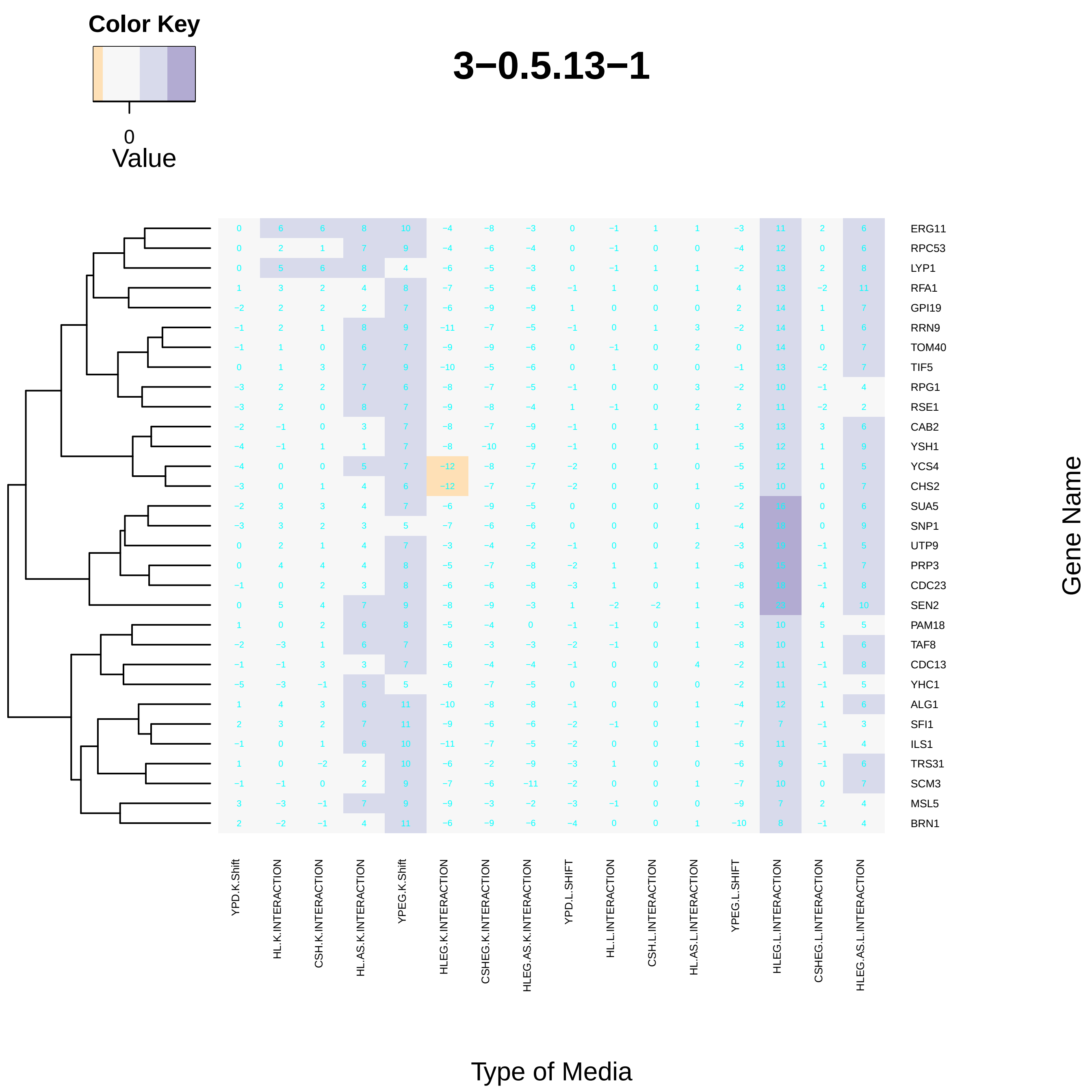
3−0.5.13−1
Color Key
0
Value
ERG11
RPC53
LYP1
RFA1
GPI19
RRN9
TOM40
TIF5
RPG1
RSE1
CAB2
YSH1
YCS4
CHS2
SUA5
SNP1
UTP9
PRP3
CDC23
SEN2
PAM18
TAF8
CDC13
YHC1
ALG1
SFI1
ILS1
TRS31
SCM3
MSL5
BRN1
Gene Name
YPD.K.Shift
HL.K.INTERACTION
CSH.K.INTERACTION
HL.AS.K.INTERACTION
YPEG.K.Shift
HLEG.K.INTERACTION
CSHEG.K.INTERACTION
HLEG.AS.K.INTERACTION
YPD.L.SHIFT
HL.L.INTERACTION
CSH.L.INTERACTION
HL.AS.L.INTERACTION
YPEG.L.SHIFT
HLEG.L.INTERACTION
CSHEG.L.INTERACTION
HLEG.AS.L.INTERACTION
Type of Media

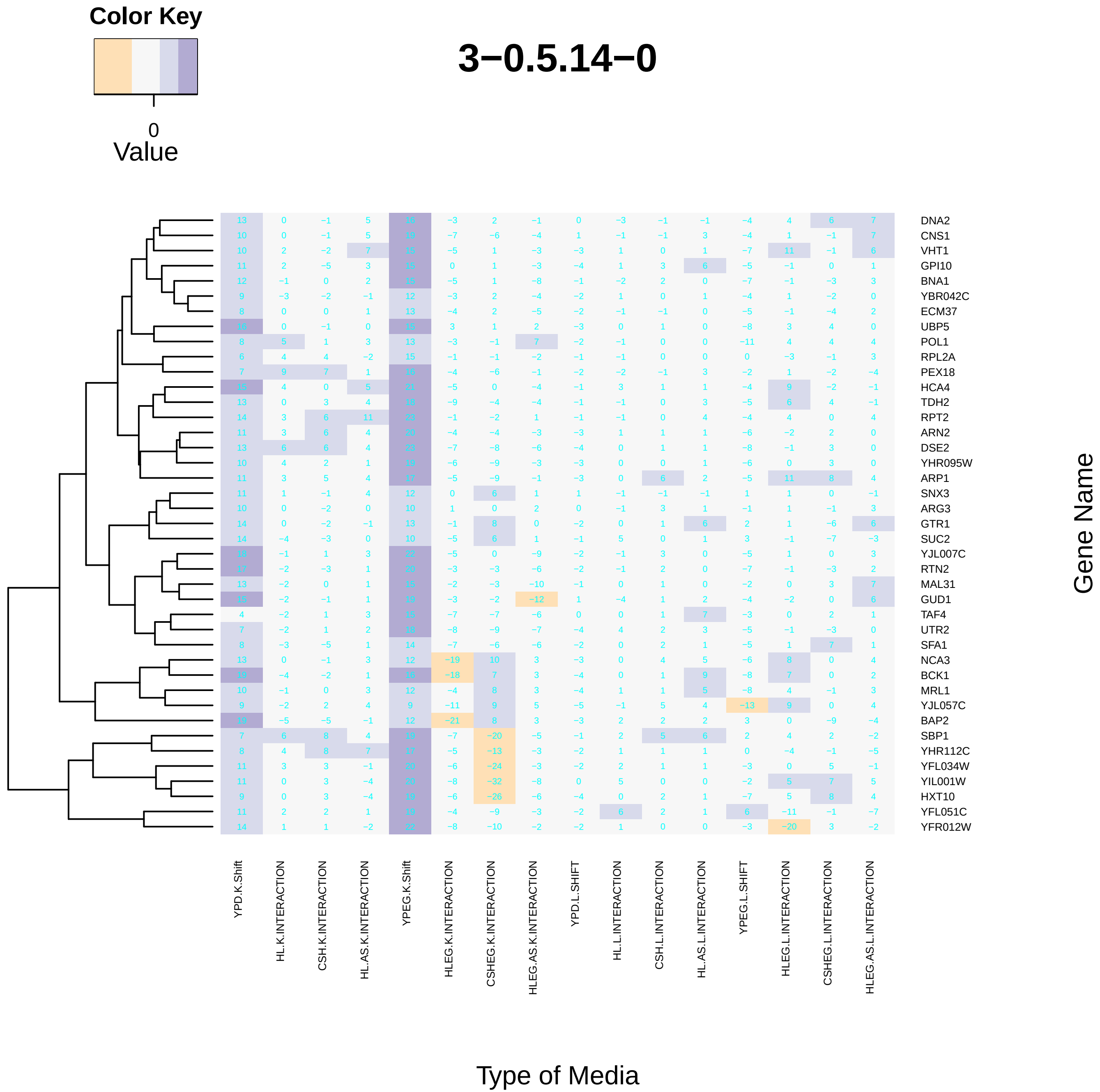

Color Key
0
Value
3−0.5.14−0
DNA2
CNS1
VHT1
GPI10
BNA1
YBR042C
ECM37
UBP5
POL1
RPL2A
PEX18
HCA4
TDH2
RPT2
ARN2
DSE2
YHR095W
ARP1
SNX3
ARG3
GTR1
SUC2
YJL007C
RTN2
MAL31
GUD1
TAF4
UTR2
SFA1
NCA3
BCK1
MRL1
YJL057C
BAP2
SBP1
YHR112C
YFL034W
YIL001W
HXT10
YFL051C
YFR012W
Gene Name
YPD.K.Shift
HL.K.INTERACTION
CSH.K.INTERACTION
HL.AS.K.INTERACTION
YPEG.K.Shift
HLEG.K.INTERACTION
CSHEG.K.INTERACTION
HLEG.AS.K.INTERACTION
YPD.L.SHIFT
HL.L.INTERACTION
CSH.L.INTERACTION
HL.AS.L.INTERACTION
YPEG.L.SHIFT
HLEG.L.INTERACTION
CSHEG.L.INTERACTION
HLEG.AS.L.INTERACTION
Type of Media

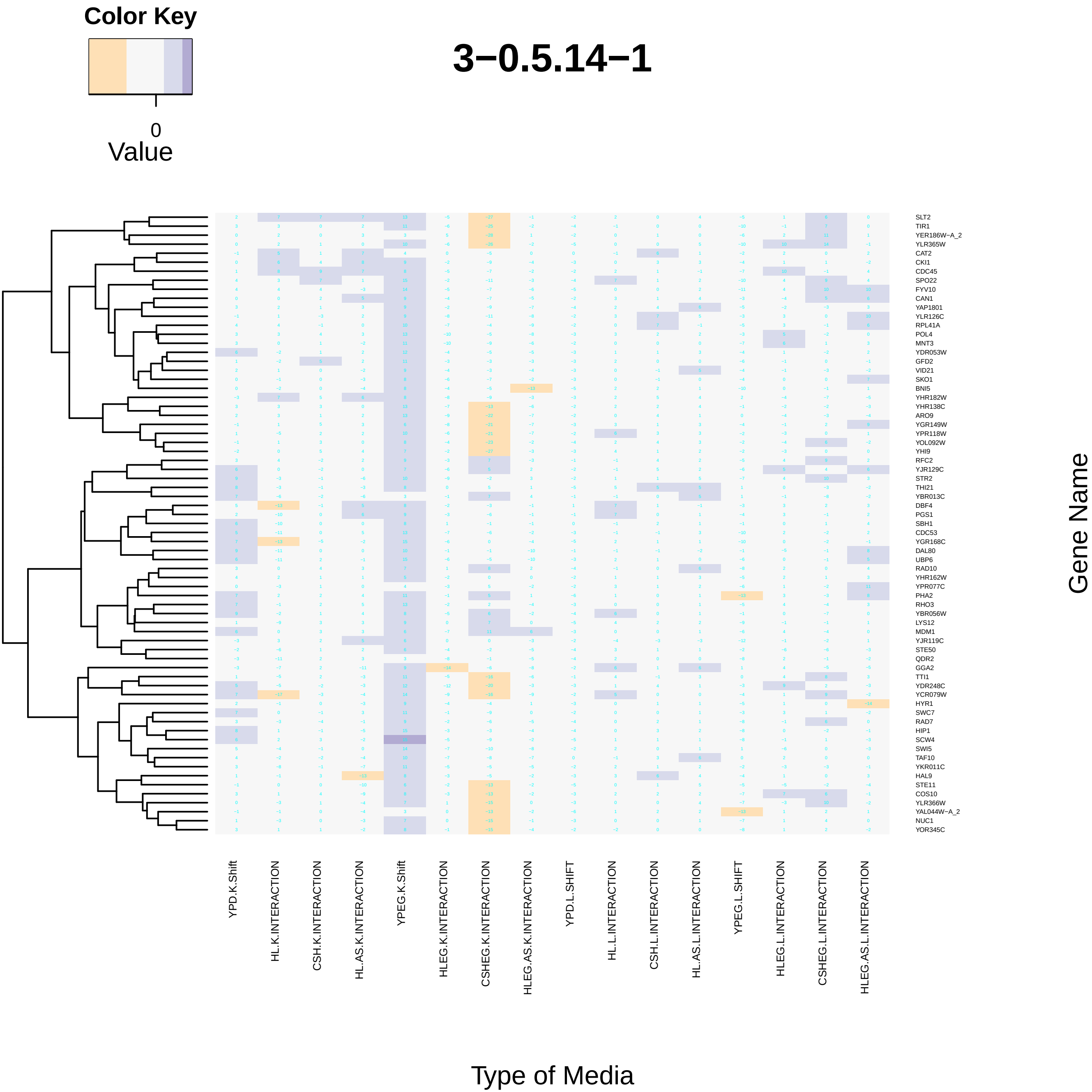
Color Key
0
Value
3−0.5.14−1
SLT2
TIR1
YER186W−A_2
YLR365W
CAT2
CKI1
CDC45
SPO22
FYV10
CAN1
YAP1801
YLR126C
RPL41A
POL4
MNT3
YDR053W
GFD2
VID21
SKO1
BNI5
YHR182W
YHR138C
ARO9
YGR149W
YPR118W
YOL092W
YHI9
RFC2
YJR129C
STR2
THI21
YBR013C
DBF4
PGS1
SBH1
CDC53
YGR168C
DAL80
UBP6
RAD10
YHR162W
YPR077C
PHA2
RHO3
YBR056W
LYS12
MDM1
YJR119C
STE50
QDR2
GGA2
TTI1
YDR248C
YCR079W
HYR1
SWC7
RAD7
HIP1
SCW4
SWI5
TAF10
YKR011C
HAL9
STE11
COS10
YLR366W
YAL044W−A_2
NUC1
YOR345C
Gene Name
YPD.K.Shift
HL.K.INTERACTION
CSH.K.INTERACTION
HL.AS.K.INTERACTION
YPEG.K.Shift
HLEG.K.INTERACTION
CSHEG.K.INTERACTION
HLEG.AS.K.INTERACTION
YPD.L.SHIFT
HL.L.INTERACTION
CSH.L.INTERACTION
HL.AS.L.INTERACTION
YPEG.L.SHIFT
HLEG.L.INTERACTION
CSHEG.L.INTERACTION
HLEG.AS.L.INTERACTION
Type of Media

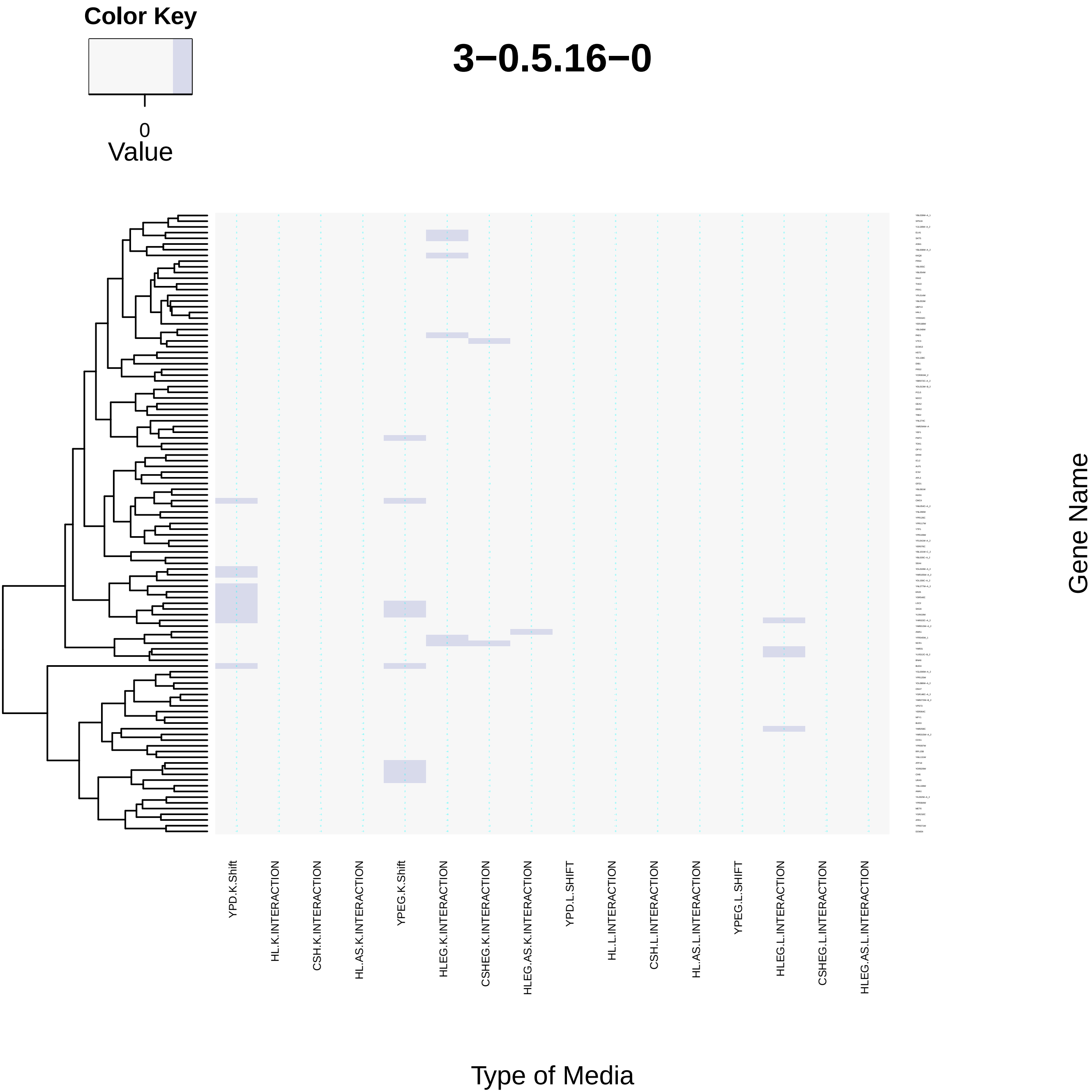
Color Key
0
Value
3−0.5.16−0
Gene Name
YPD.K.Shift
HL.K.INTERACTION
CSH.K.INTERACTION
HL.AS.K.INTERACTION
YPEG.K.Shift
HLEG.K.INTERACTION
CSHEG.K.INTERACTION
HLEG.AS.K.INTERACTION
YPD.L.SHIFT
HL.L.INTERACTION
CSH.L.INTERACTION
HL.AS.L.INTERACTION
YPEG.L.SHIFT
HLEG.L.INTERACTION
CSHEG.L.INTERACTION
HLEG.AS.L.INTERACTION
Type of Media

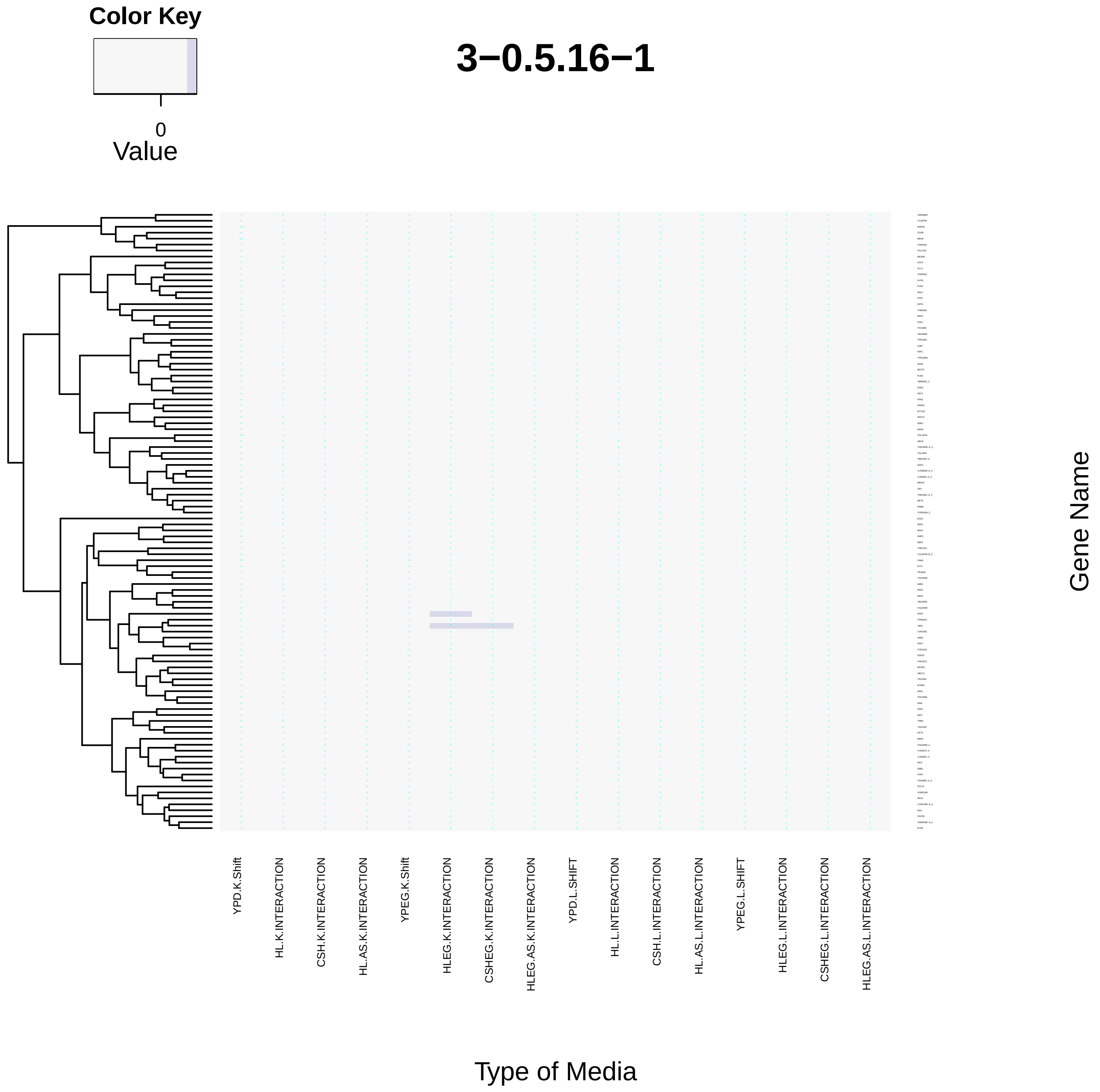

Color Key
0
Value
3−0.5.16−1
Gene Name
YPD.K.Shift
HL.K.INTERACTION
CSH.K.INTERACTION
HL.AS.K.INTERACTION
YPEG.K.Shift
HLEG.K.INTERACTION
CSHEG.K.INTERACTION
HLEG.AS.K.INTERACTION
YPD.L.SHIFT
HL.L.INTERACTION
CSH.L.INTERACTION
HL.AS.L.INTERACTION
YPEG.L.SHIFT
HLEG.L.INTERACTION
CSHEG.L.INTERACTION
HLEG.AS.L.INTERACTION
Type of Media

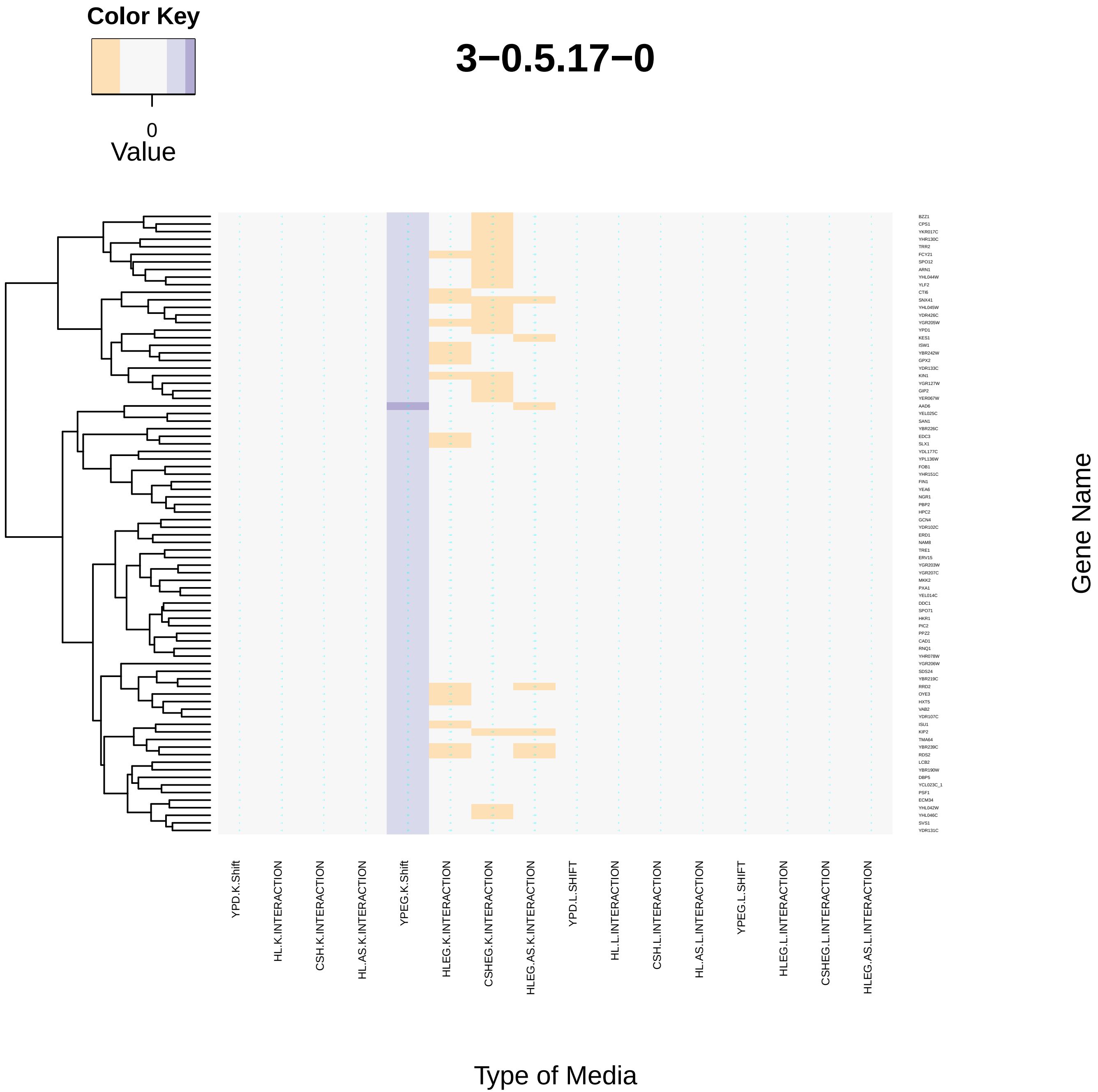

Color Key
0
Value
3−0.5.17−0
BZZ1
CPS1
YKR017C
YHR130C
TRR2
FCY21
SPO12
ARN1
YHL044W
YLF2
CTI6
SNX41
YHL045W
YDR426C
YGR205W
YPD1
KES1
ISW1
YBR242W
GPX2
YDR133C
KIN1
YGR127W
GIP2
YER067W
AAD6
YEL025C
SAN1
YBR226C
EDC3
SLX1
YDL177C
YPL136W
FOB1
YHR151C
FIN1
YEA6
NGR1
PBP2
HPC2
GCN4
YDR102C
ERD1
NAM8
TRE1
ERV15
YGR203W
YGR207C
MKK2
PXA1
YEL014C
DDC1
SPO71
HKR1
PIC2
PPZ2
CAD1
RNQ1
YHR078W
YGR206W
SDS24
YBR219C
RRD2
OYE3
HXT5
VAB2
YDR107C
ISU1
KIP2
TMA64
YBR239C
RDS2
LCB2
YBR190W
DBP5
YCL023C_1
PSF1
ECM34
YHL042W
YHL046C
SVS1
YDR131C
Gene Name
YPD.K.Shift
HL.K.INTERACTION
CSH.K.INTERACTION
HL.AS.K.INTERACTION
YPEG.K.Shift
HLEG.K.INTERACTION
CSHEG.K.INTERACTION
HLEG.AS.K.INTERACTION
YPD.L.SHIFT
HL.L.INTERACTION
CSH.L.INTERACTION
HL.AS.L.INTERACTION
YPEG.L.SHIFT
HLEG.L.INTERACTION
CSHEG.L.INTERACTION
HLEG.AS.L.INTERACTION
Type of Media

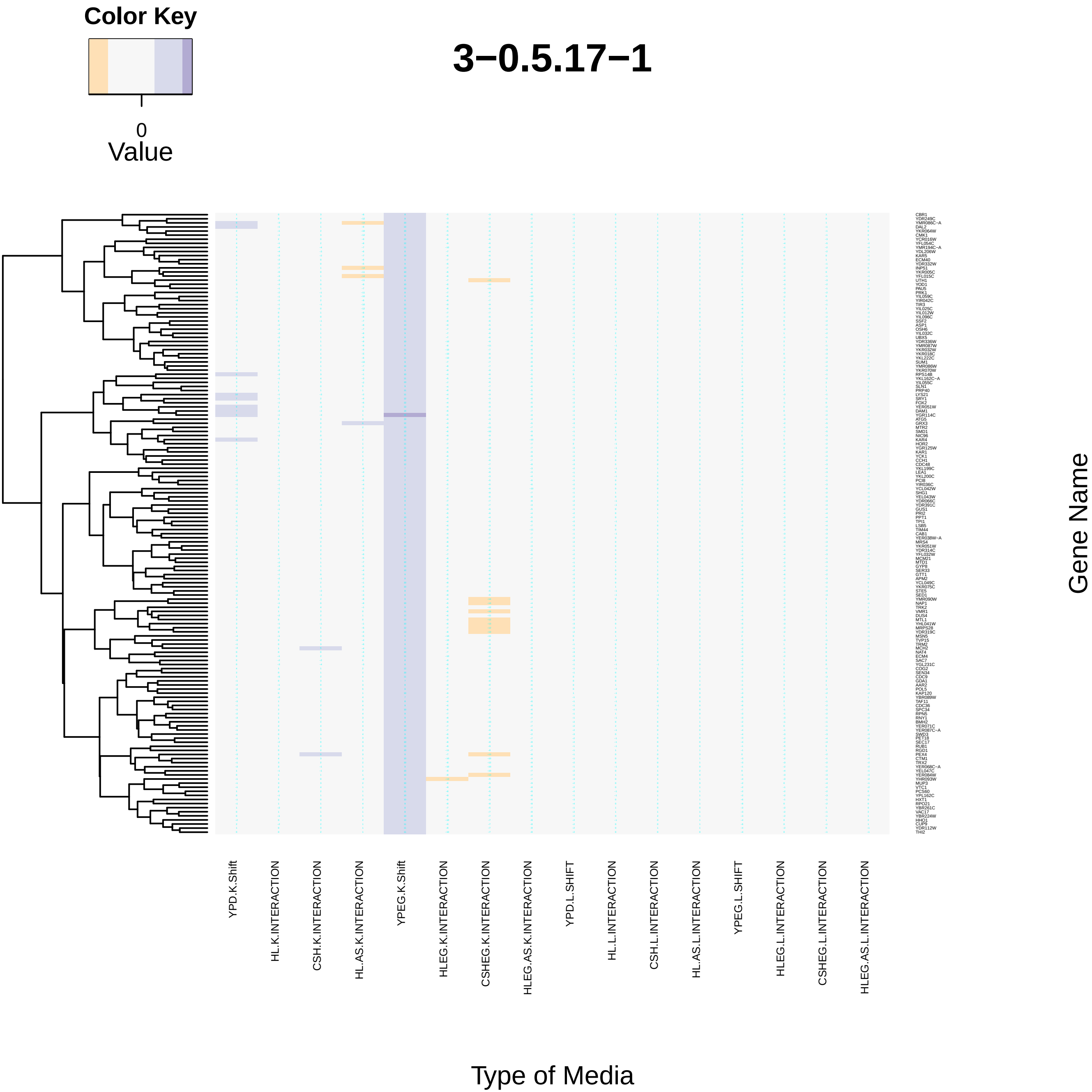
Color Key
0
Value
3−0.5.17−1
YPD.K.Shift
HL.K.INTERACTION
CSH.K.INTERACTION
HL.AS.K.INTERACTION
YPEG.K.Shift
HLEG.K.INTERACTION
CSHEG.K.INTERACTION
HLEG.AS.K.INTERACTION
YPD.L.SHIFT
HL.L.INTERACTION
CSH.L.INTERACTION
HL.AS.L.INTERACTION
YPEG.L.SHIFT
HLEG.L.INTERACTION
CSHEG.L.INTERACTION
HLEG.AS.L.INTERACTION
Type of Media
Gene Name

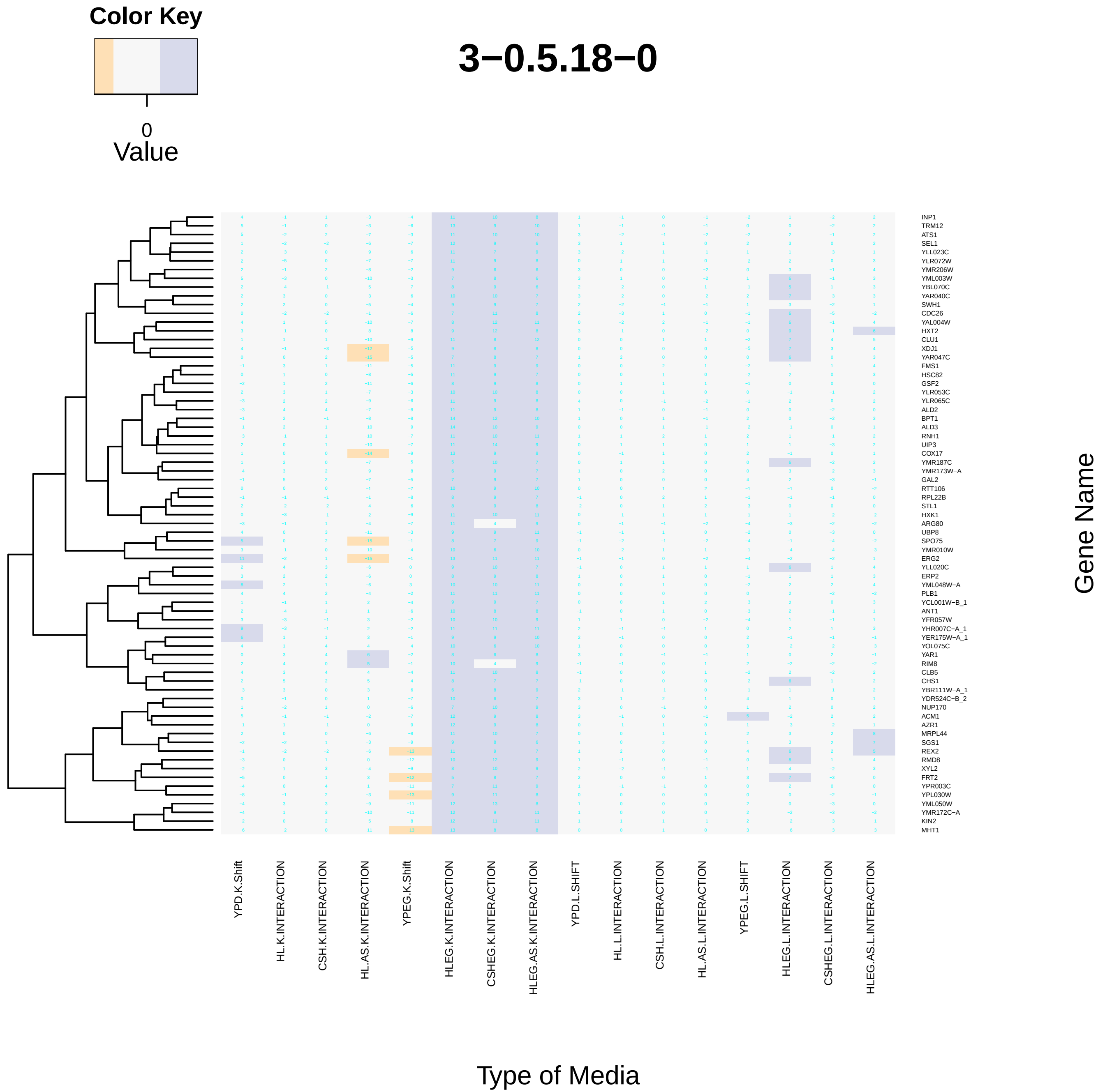

Color Key
0
Value
3−0.5.18−0
INP1
TRM12
ATS1
SEL1
YLL023C
YLR072W
YMR206W
YML003W
YBL070C
YAR040C
SWH1
CDC26
YAL004W
HXT2
CLU1
XDJ1
YAR047C
FMS1
HSC82
GSF2
YLR053C
YLR065C
ALD2
BPT1
ALD3
RNH1
UIP3
COX17
YMR187C
YMR173W−A
GAL2
RTT106
RPL22B
STL1
HXK1
ARG80
UBP8
SPO75
YMR010W
ERG2
YLL020C
ERP2
YML048W−A
PLB1
YCL001W−B_1
ANT1
YFR057W
YHR007C−A_1
YER175W−A_1
YOL075C
YAR1
RIM8
CLB5
CHS1
YBR111W−A_1
YDR524C−B_2
NUP170
ACM1
AZR1
MRPL44
SGS1
REX2
RMD8
XYL2
FRT2
YPR003C
YPL030W
YML050W
YMR172C−A
KIN2
MHT1
Gene Name
YPD.K.Shift
HL.K.INTERACTION
CSH.K.INTERACTION
HL.AS.K.INTERACTION
YPEG.K.Shift
HLEG.K.INTERACTION
CSHEG.K.INTERACTION
HLEG.AS.K.INTERACTION
YPD.L.SHIFT
HL.L.INTERACTION
CSH.L.INTERACTION
HL.AS.L.INTERACTION
YPEG.L.SHIFT
HLEG.L.INTERACTION
CSHEG.L.INTERACTION
HLEG.AS.L.INTERACTION
Type of Media

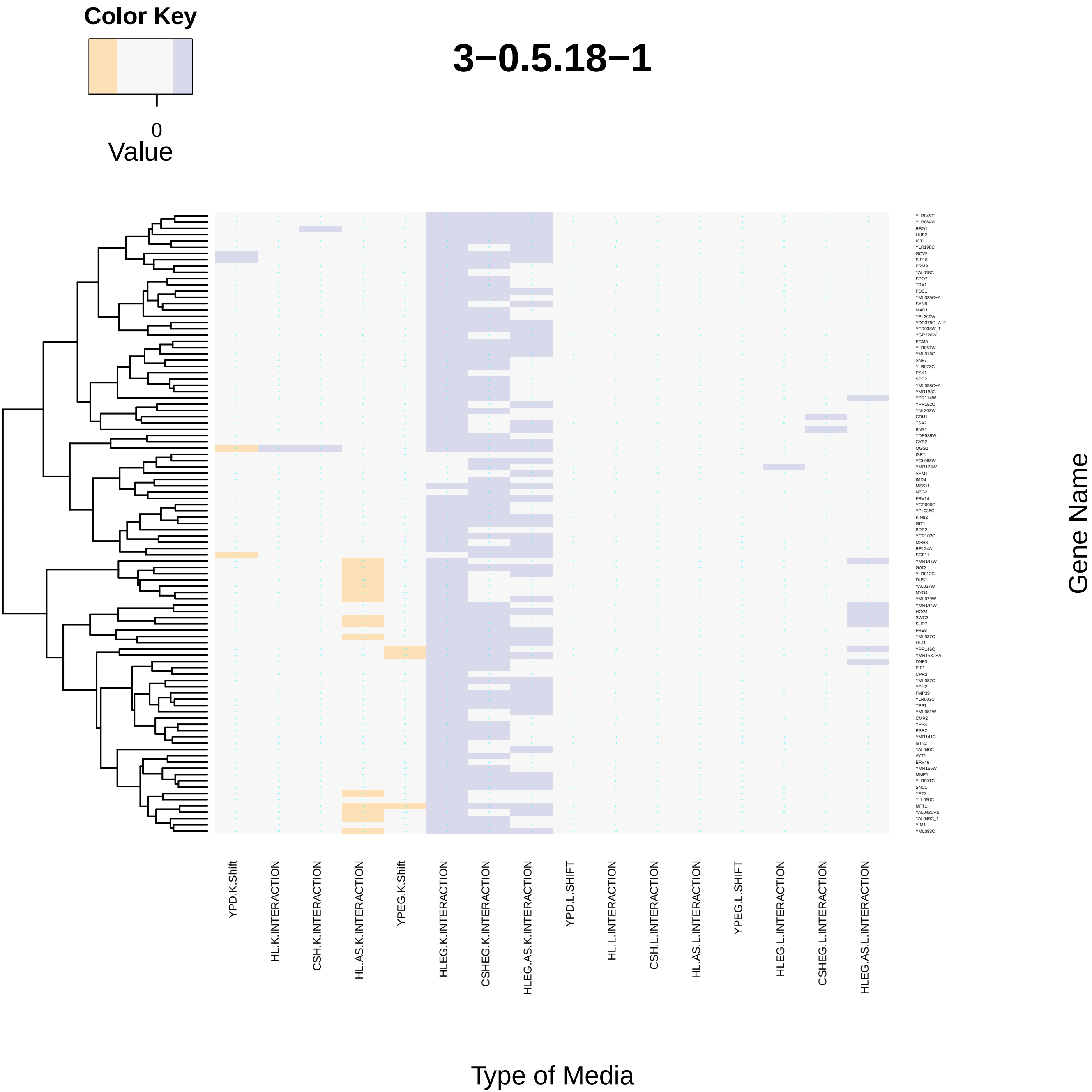

Color Key
0
Value
3−0.5.18−1
YLR049C
YLR064W
RBG1
HUF2
ICT1
YLR198C
GCV2
SIP18
PRM9
YAL018C
SPO7
TRX1
PDC1
YML035C−A
SYN8
MAD1
YPL260W
YDR379C−A_2
YFR038W_1
YGR228W
ECM5
YLR057W
YML018C
SNF7
YLR073C
PSK1
SPC2
YML058C−A
YMR163C
YPR114W
YPR152C
YNL303W
CDH1
TSA2
BNS1
YDR539W
CYB2
OGG1
ISR1
YGL085W
YMR178W
SEM1
IMD4
MSS11
NTG2
ERV14
YCR090C
YPL035C
KIN82
GIT1
BRE2
YCR102C
MSH3
RPL24A
SGF11
YMR147W
GAT3
YLR012C
DUS1
YAL027W
MYO4
YML079W
YMR144W
HOG1
SWC3
SUR7
FRE8
YML037C
HLJ1
YPR146C
YMR153C−A
DNF3
PIF1
CPR3
YML087C
YEH2
FMP39
YLR003C
TPP1
YML081W
CMP2
YPS3
PSR2
YMR141C
GTT2
YAL046C
AYT1
ERV46
YMR155W
MMP1
YLR001C
SNC1
YET2
YLL056C
MFT1
YAL043C−a
YAL049C_1
YIM1
YML083C
Gene Name
YPD.K.Shift
HL.K.INTERACTION
CSH.K.INTERACTION
HL.AS.K.INTERACTION
YPEG.K.Shift
HLEG.K.INTERACTION
CSHEG.K.INTERACTION
HLEG.AS.K.INTERACTION
YPD.L.SHIFT
HL.L.INTERACTION
CSH.L.INTERACTION
HL.AS.L.INTERACTION
YPEG.L.SHIFT
HLEG.L.INTERACTION
CSHEG.L.INTERACTION
HLEG.AS.L.INTERACTION
Type of Media

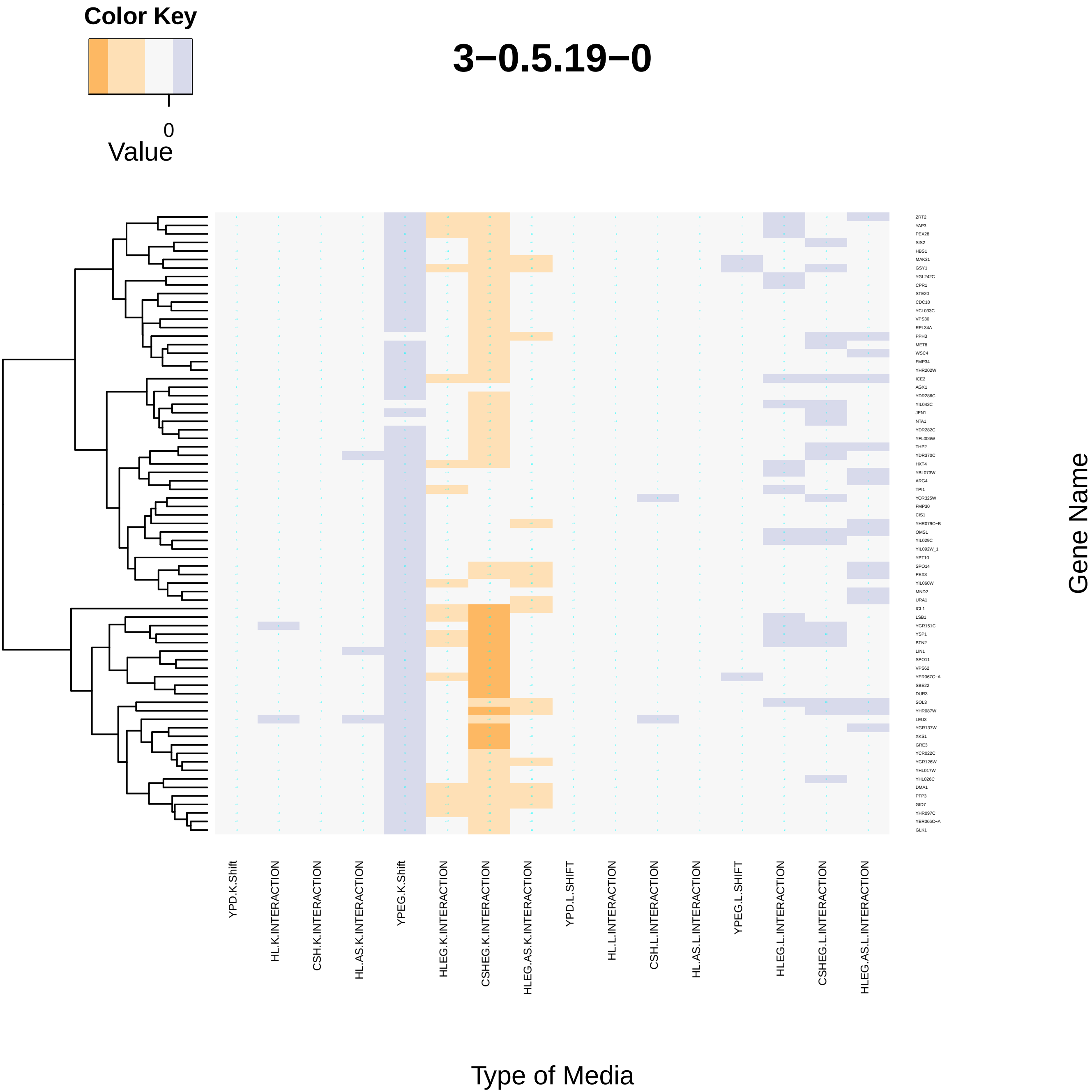
Color Key
0
Value
3−0.5.19−0
ZRT2
YAP3
PEX28
SIS2
HBS1
MAK31
GSY1
YGL242C
CPR1
STE20
CDC10
YCL033C
VPS30
RPL34A
PPH3
MET8
WSC4
FMP34
YHR202W
ICE2
AGX1
YDR286C
YIL042C
JEN1
NTA1
YDR282C
YFL006W
THP2
YDR370C
HXT4
YBL073W
ARG4
TPI1
YOR325W
FMP30
CIS1
YHR079C−B
OMS1
YIL029C
YIL092W_1
YPT10
SPO14
PEX3
YIL060W
MND2
URA1
ICL1
LSB1
YGR151C
YSP1
BTN2
LIN1
SPO11
VPS62
YER067C−A
SBE22
DUR3
SOL3
YHR087W
LEU3
YGR137W
XKS1
GRE3
YCR022C
YGR126W
YHL017W
YHL026C
DMA1
PTP3
GID7
YHR097C
YER066C−A
GLK1
Gene Name
YPD.K.Shift
HL.K.INTERACTION
CSH.K.INTERACTION
HL.AS.K.INTERACTION
YPEG.K.Shift
HLEG.K.INTERACTION
CSHEG.K.INTERACTION
HLEG.AS.K.INTERACTION
YPD.L.SHIFT
HL.L.INTERACTION
CSH.L.INTERACTION
HL.AS.L.INTERACTION
YPEG.L.SHIFT
HLEG.L.INTERACTION
CSHEG.L.INTERACTION
HLEG.AS.L.INTERACTION
Type of Media

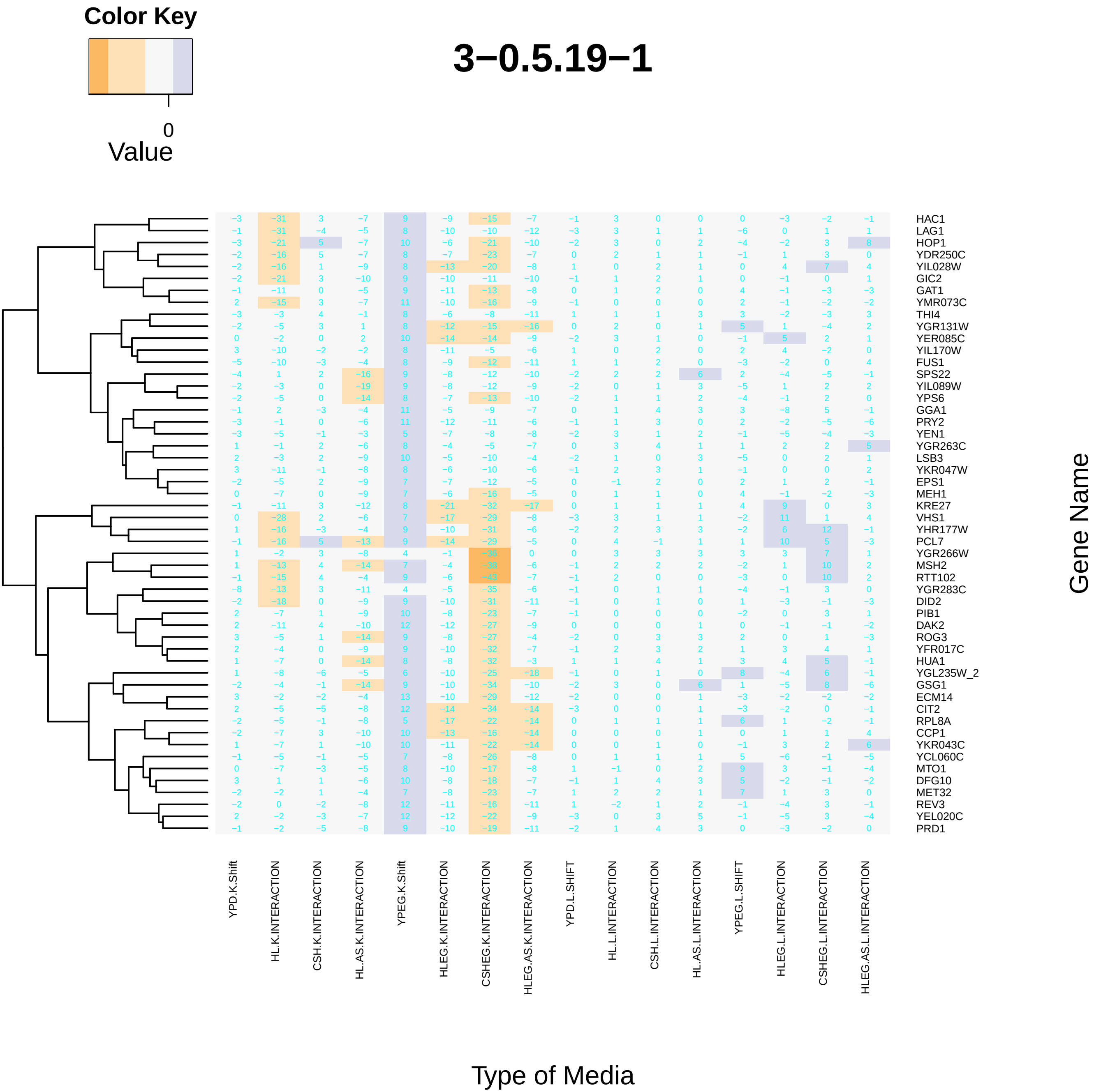

Color Key
0
Value
3−0.5.19−1
HAC1
LAG1
HOP1
YDR250C
YIL028W
GIC2
GAT1
YMR073C
THI4
YGR131W
YER085C
YIL170W
FUS1
SPS22
YIL089W
YPS6
GGA1
PRY2
YEN1
YGR263C
LSB3
YKR047W
EPS1
MEH1
KRE27
VHS1
YHR177W
PCL7
YGR266W
MSH2
RTT102
YGR283C
DID2
PIB1
DAK2
ROG3
YFR017C
HUA1
YGL235W_2
GSG1
ECM14
CIT2
RPL8A
CCP1
YKR043C
YCL060C
MTO1
DFG10
MET32
REV3
YEL020C
PRD1
YPD.K.Shift
HL.K.INTERACTION
CSH.K.INTERACTION
HL.AS.K.INTERACTION
YPEG.K.Shift
HLEG.K.INTERACTION
CSHEG.K.INTERACTION
HLEG.AS.K.INTERACTION
YPD.L.SHIFT
HL.L.INTERACTION
CSH.L.INTERACTION
HL.AS.L.INTERACTION
YPEG.L.SHIFT
HLEG.L.INTERACTION
CSHEG.L.INTERACTION
HLEG.AS.L.INTERACTION
Gene Name
Type of Media

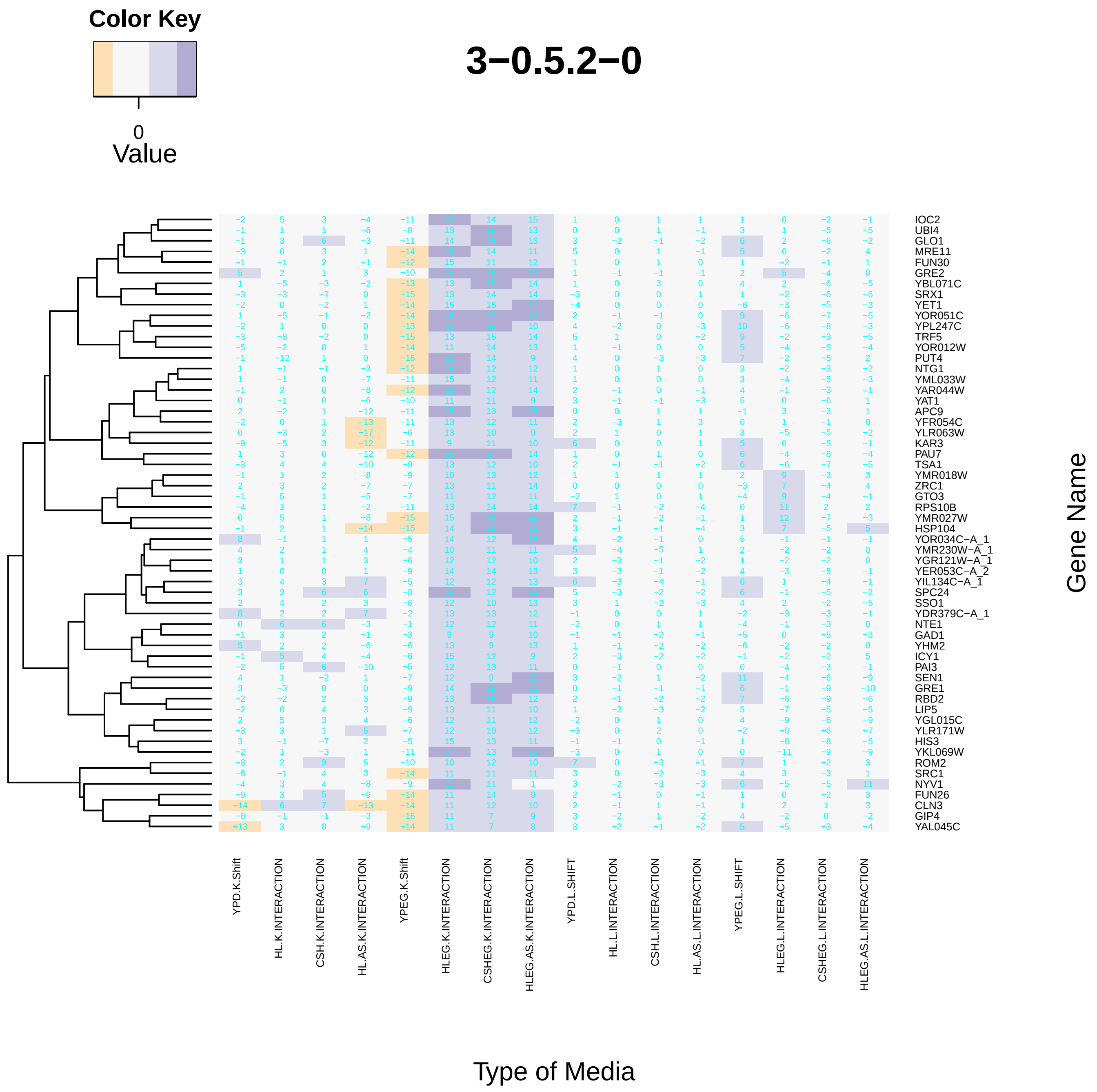
Color Key
0
Value
3−0.5.2−0
IOC2
UBI4
GLO1
MRE11
FUN30
GRE2
YBL071C
SRX1
YET1
YOR051C
YPL247C
TRF5
YOR012W
PUT4
NTG1
YML033W
YAR044W
YAT1
APC9
YFR054C
YLR063W
KAR3
PAU7
TSA1
YMR018W
ZRC1
GTO3
RPS10B
YMR027W
HSP104
YOR034C−A_1
YMR230W−A_1
YGR121W−A_1
YER053C−A_2
YIL134C−A_1
SPC24
SSO1
YDR379C−A_1
NTE1
GAD1
YHM2
ICY1
PAI3
SEN1
GRE1
RBD2
LIP5
YGL015C
YLR171W
HIS3
YKL069W
ROM2
SRC1
NYV1
FUN26
CLN3
GIP4
YAL045C
Gene Name
YPD.K.Shift
HL.K.INTERACTION
CSH.K.INTERACTION
HL.AS.K.INTERACTION
YPEG.K.Shift
HLEG.K.INTERACTION
CSHEG.K.INTERACTION
HLEG.AS.K.INTERACTION
YPD.L.SHIFT
HL.L.INTERACTION
CSH.L.INTERACTION
HL.AS.L.INTERACTION
YPEG.L.SHIFT
HLEG.L.INTERACTION
CSHEG.L.INTERACTION
HLEG.AS.L.INTERACTION
Type of Media

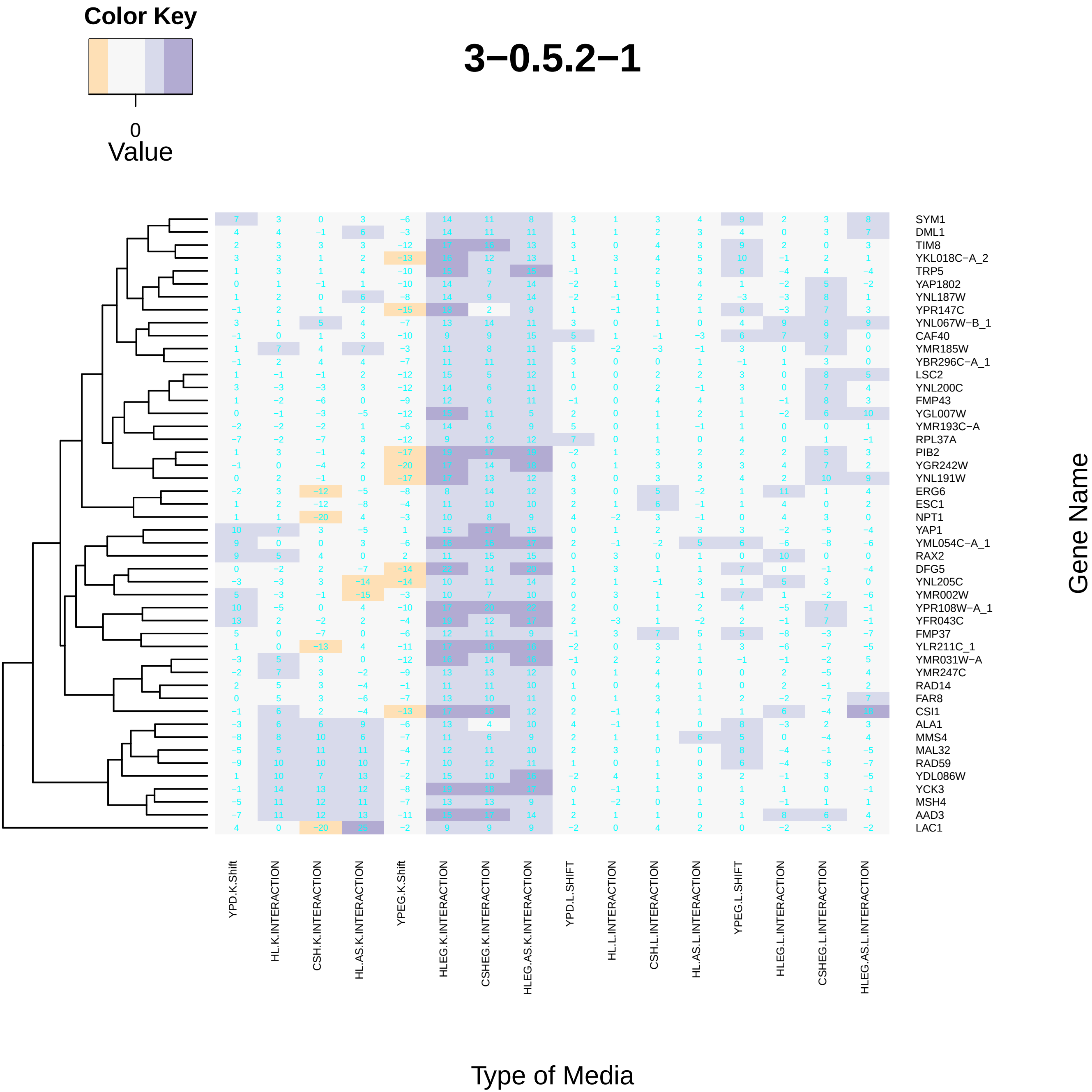

Color Key
0
Value
3−0.5.2−1
SYM1
DML1
TIM8
YKL018C−A_2
TRP5
YAP1802
YNL187W
YPR147C
YNL067W−B_1
CAF40
YMR185W
YBR296C−A_1
LSC2
YNL200C
FMP43
YGL007W
YMR193C−A
RPL37A
PIB2
YGR242W
YNL191W
ERG6
ESC1
NPT1
YAP1
YML054C−A_1
RAX2
DFG5
YNL205C
YMR002W
YPR108W−A_1
YFR043C
FMP37
YLR211C_1
YMR031W−A
YMR247C
RAD14
FAR8
CSI1
ALA1
MMS4
MAL32
RAD59
YDL086W
YCK3
MSH4
AAD3
LAC1
Gene Name
YPD.K.Shift
HL.K.INTERACTION
CSH.K.INTERACTION
HL.AS.K.INTERACTION
YPEG.K.Shift
HLEG.K.INTERACTION
CSHEG.K.INTERACTION
HLEG.AS.K.INTERACTION
YPD.L.SHIFT
HL.L.INTERACTION
CSH.L.INTERACTION
HL.AS.L.INTERACTION
YPEG.L.SHIFT
HLEG.L.INTERACTION
CSHEG.L.INTERACTION
HLEG.AS.L.INTERACTION
Type of Media

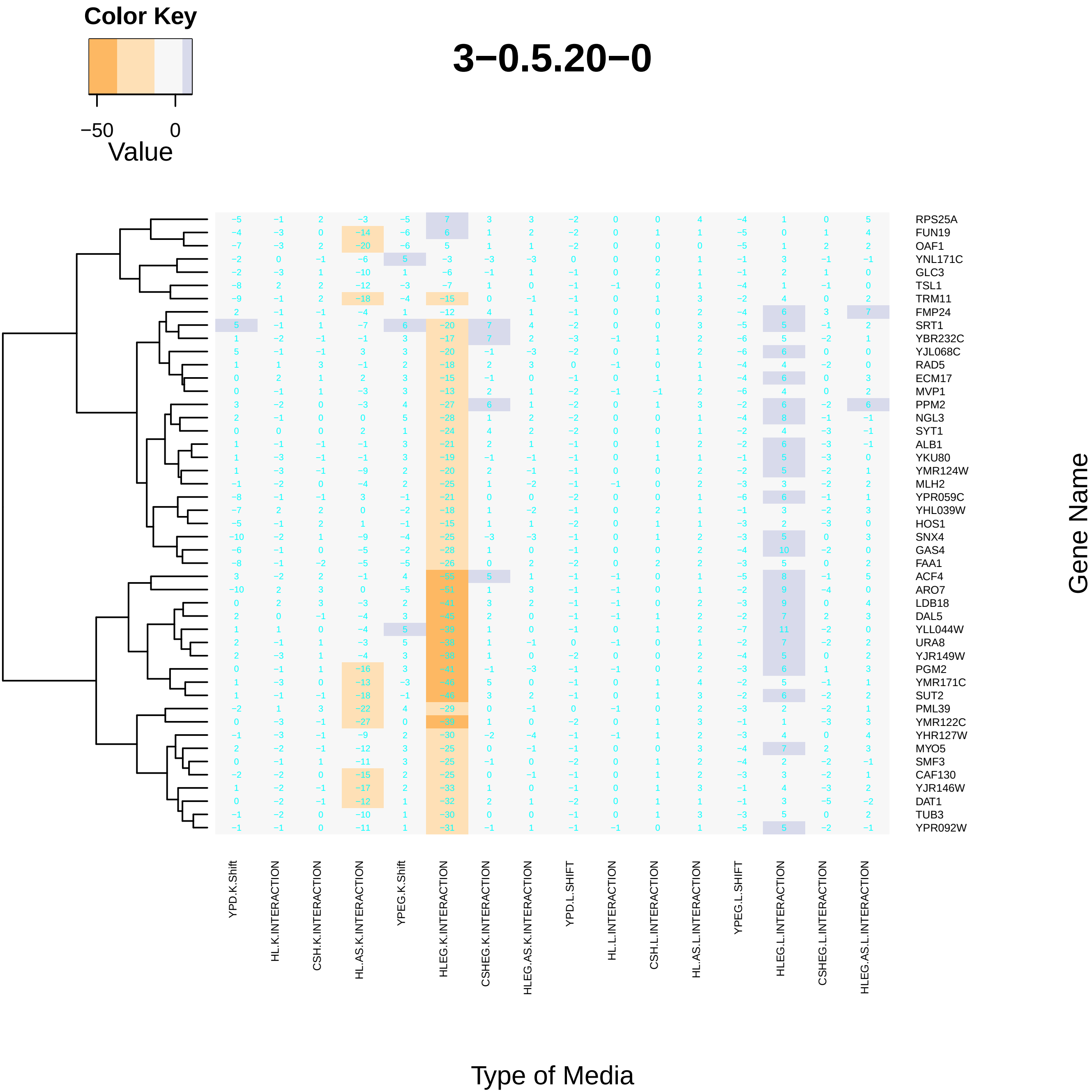

3−0.5.20−0
Color Key
−50
0
Value
YPD.K.Shift
HL.K.INTERACTION
CSH.K.INTERACTION
HL.AS.K.INTERACTION
YPEG.K.Shift
HLEG.K.INTERACTION
CSHEG.K.INTERACTION
HLEG.AS.K.INTERACTION
YPD.L.SHIFT
HL.L.INTERACTION
CSH.L.INTERACTION
HL.AS.L.INTERACTION
YPEG.L.SHIFT
HLEG.L.INTERACTION
CSHEG.L.INTERACTION
HLEG.AS.L.INTERACTION
Type of Media
Gene Name
RPS25A
FUN19
OAF1
YNL171C
GLC3
TSL1
TRM11
FMP24
SRT1
YBR232C
YJL068C
RAD5
ECM17
MVP1
PPM2
NGL3
SYT1
ALB1
YKU80
YMR124W
MLH2
YPR059C
YHL039W
HOS1
SNX4
GAS4
FAA1
ACF4
ARO7
LDB18
DAL5
YLL044W
URA8
YJR149W
PGM2
YMR171C
SUT2
PML39
YMR122C
YHR127W
MYO5
SMF3
CAF130
YJR146W
DAT1
TUB3
YPR092W

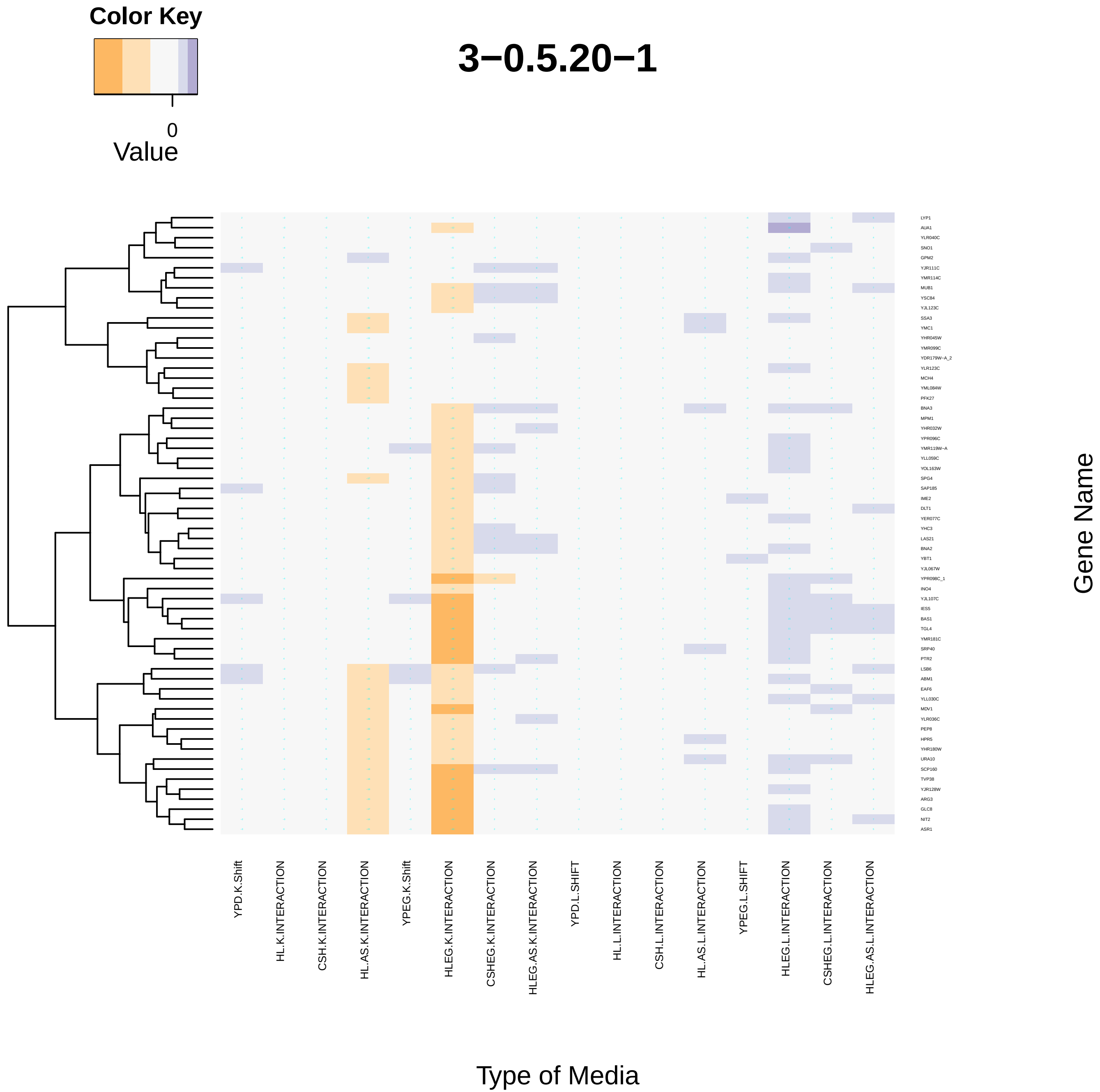
Color Key
0
Value
3−0.5.20−1
LYP1
AUA1
YLR040C
SNO1
GPM2
YJR111C
YMR114C
MUB1
YSC84
YJL123C
SSA3
YMC1
YHR045W
YMR099C
YDR179W−A_2
YLR123C
MCH4
YML084W
PFK27
BNA3
MPM1
YHR032W
YPR096C
YMR119W−A
YLL059C
YOL163W
SPG4
SAP185
IME2
DLT1
YER077C
YHC3
LAS21
BNA2
YBT1
YJL067W
YPR098C_1
INO4
YJL107C
IES5
BAS1
TGL4
YMR181C
SRP40
PTR2
LSB6
ABM1
EAF6
YLL030C
MDV1
YLR036C
PEP8
HPR5
YHR180W
URA10
SCP160
TVP38
YJR128W
ARG3
GLC8
NIT2
ASR1
Gene Name
YPD.K.Shift
HL.K.INTERACTION
CSH.K.INTERACTION
HL.AS.K.INTERACTION
YPEG.K.Shift
HLEG.K.INTERACTION
CSHEG.K.INTERACTION
HLEG.AS.K.INTERACTION
YPD.L.SHIFT
HL.L.INTERACTION
CSH.L.INTERACTION
HL.AS.L.INTERACTION
YPEG.L.SHIFT
HLEG.L.INTERACTION
CSHEG.L.INTERACTION
HLEG.AS.L.INTERACTION
Type of Media

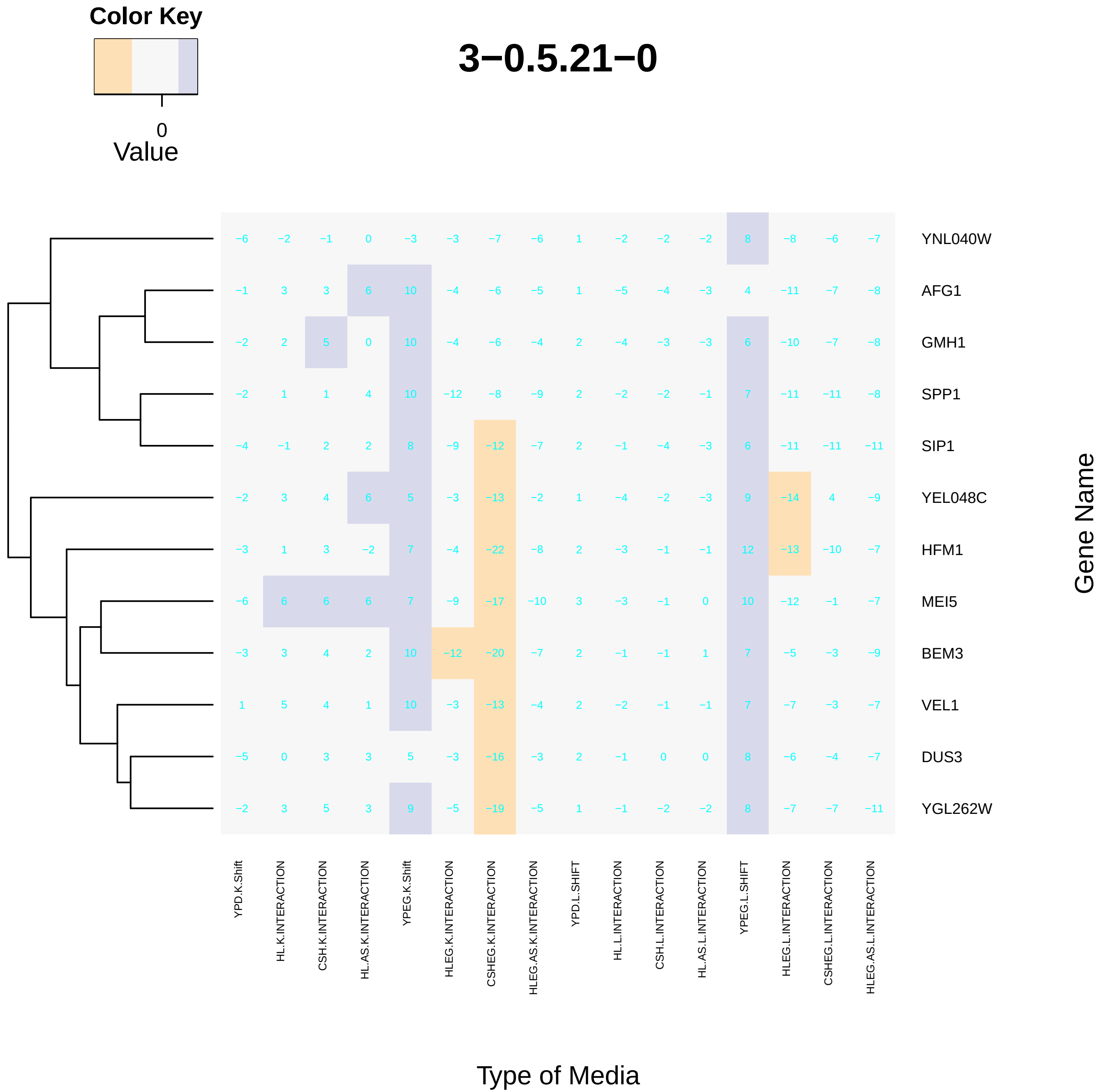

Gene	YPD.K.Shift	HL.K.INTERACTION	CSH.K.INTERACTION	HL.AS.K.INTERACTION	YPEG.K.Shift	HLEG.K.INTERACTION	CSHEG.K.INTERACTION	HLEG.AS.K.INTERACTION	YPD.L.SHIFT	HL.L.INTERACTION	CSH.L.INTERACTION	HL.AS.L.INTERACTION	YPEG.L.SHIFT	HLEG.L.INTERACTION	CSHEG.L.INTERACTION	HLEG.AS.L.INTERACTION
YNL040W	−6	−2	−1	0	−3	−3	−7	−6	1	−2	−2	−2	8	−8	−6	−7
AFG1	−1	3	3	6	10	−4	−6	−5	1	−5	−4	−3	4	−11	−7	−8
GMH1	−2	2	5	0	10	−4	−6	−4	2	−4	−3	−3	6	−10	−7	−8
SPP1	−2	1	1	4	10	−12	−8	−9	2	−2	−2	−1	7	−11	−11	−8
SIP1	−4	−1	2	2	8	−9	−12	−7	2	−1	−4	−3	6	−11	−11	−11
YEL048C	−2	3	4	6	5	−3	−13	−2	1	−4	−2	−3	9	−14	4	−9
HFM1	−3	1	3	−2	7	−4	−22	−8	2	−3	−1	−1	12	−13	−10	−7
MEI5	−6	6	6	6	7	−9	−17	−10	3	−3	−1	0	10	−12	−1	−7
BEM3	−3	3	4	2	10	−12	−20	−7	2	−1	−1	1	7	−5	−3	−9
VEL1	1	5	4	1	10	−3	−13	−4	2	−2	−1	−1	7	−7	−3	−7
DUS3	−5	0	3	3	5	−3	−16	−3	2	−1	0	0	8	−6	−4	−7
YGL262W	−2	3	5	3	9	−5	−19	−5	1	−1	−2	−2	8	−7	−7	−11

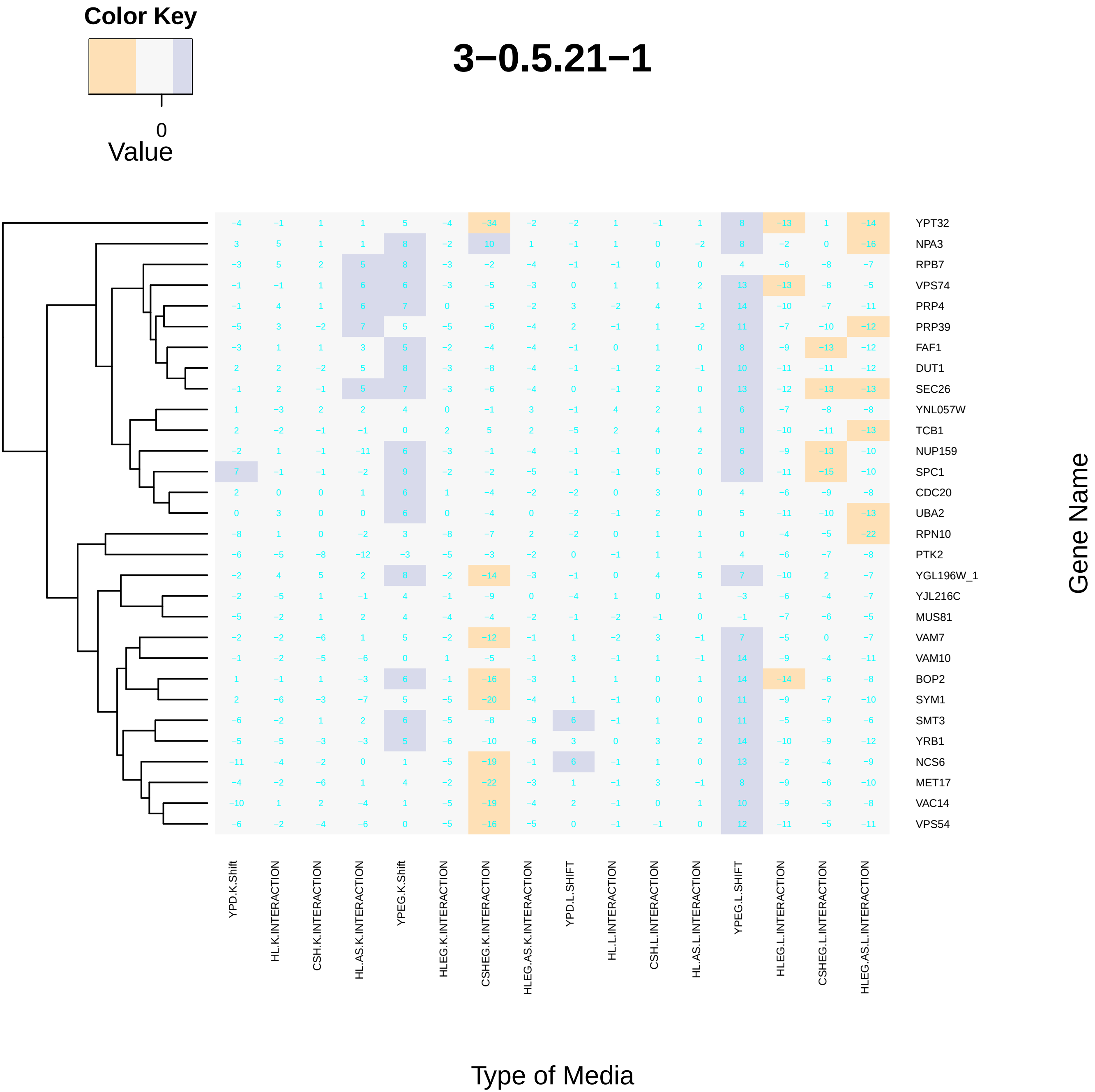

Color Key
0
Value
3−0.5.21−1
YPT32
NPA3
RPB7
VPS74
PRP4
PRP39
FAF1
DUT1
SEC26
YNL057W
TCB1
NUP159
SPC1
CDC20
UBA2
RPN10
PTK2
YGL196W_1
YJL216C
MUS81
VAM7
VAM10
BOP2
SYM1
SMT3
YRB1
NCS6
MET17
VAC14
VPS54
Gene Name
YPD.K.Shift
HL.K.INTERACTION
CSH.K.INTERACTION
HL.AS.K.INTERACTION
YPEG.K.Shift
HLEG.K.INTERACTION
CSHEG.K.INTERACTION
HLEG.AS.K.INTERACTION
YPD.L.SHIFT
HL.L.INTERACTION
CSH.L.INTERACTION
HL.AS.L.INTERACTION
YPEG.L.SHIFT
HLEG.L.INTERACTION
CSHEG.L.INTERACTION
HLEG.AS.L.INTERACTION
Type of Media

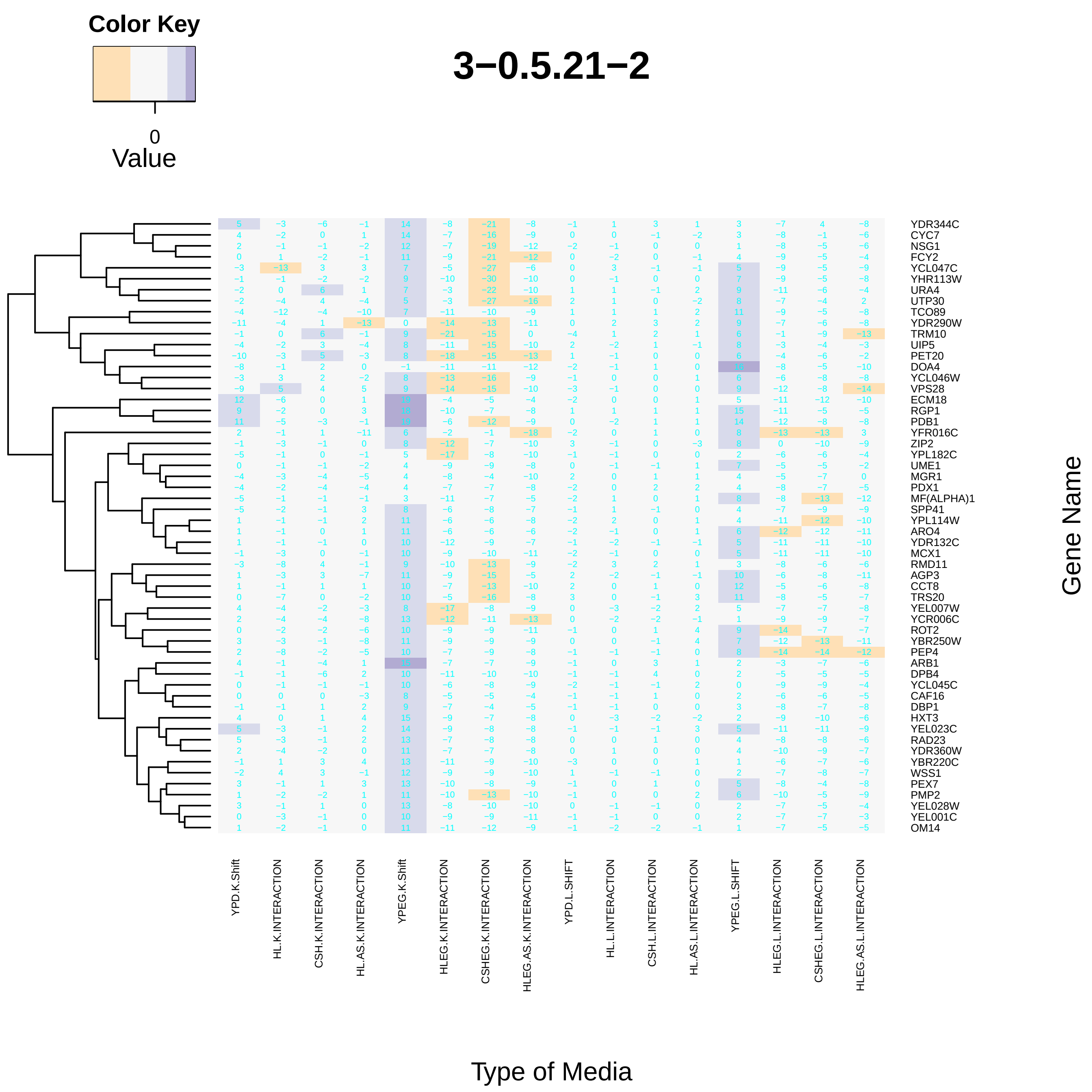
Color Key
0
Value
3−0.5.21−2
YDR344C
CYC7
NSG1
FCY2
YCL047C
YHR113W
URA4
UTP30
TCO89
YDR290W
TRM10
UIP5
PET20
DOA4
YCL046W
VPS28
ECM18
RGP1
PDB1
YFR016C
ZIP2
YPL182C
UME1
MGR1
PDX1
MF(ALPHA)1
SPP41
YPL114W
ARO4
YDR132C
MCX1
RMD11
AGP3
CCT8
TRS20
YEL007W
YCR006C
ROT2
YBR250W
PEP4
ARB1
DPB4
YCL045C
CAF16
DBP1
HXT3
YEL023C
RAD23
YDR360W
YBR220C
WSS1
PEX7
PMP2
YEL028W
YEL001C
OM14
Gene Name
YPD.K.Shift
HL.K.INTERACTION
CSH.K.INTERACTION
HL.AS.K.INTERACTION
YPEG.K.Shift
HLEG.K.INTERACTION
CSHEG.K.INTERACTION
HLEG.AS.K.INTERACTION
YPD.L.SHIFT
HL.L.INTERACTION
CSH.L.INTERACTION
HL.AS.L.INTERACTION
YPEG.L.SHIFT
HLEG.L.INTERACTION
CSHEG.L.INTERACTION
HLEG.AS.L.INTERACTION
Type of Media

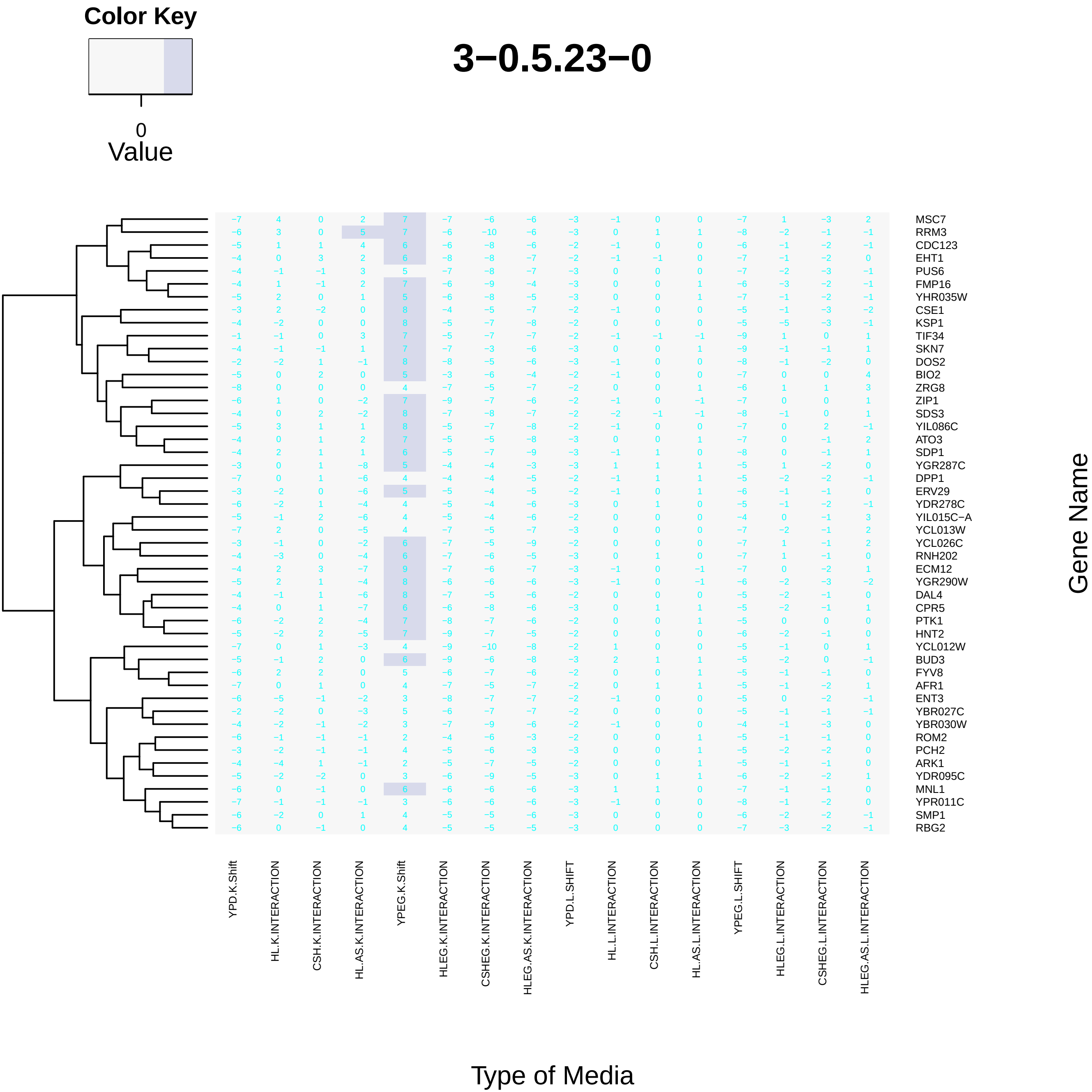
3−0.5.23−0
Color Key
0
Value
MSC7
RRM3
CDC123
EHT1
PUS6
FMP16
YHR035W
CSE1
KSP1
TIF34
SKN7
DOS2
BIO2
ZRG8
ZIP1
SDS3
YIL086C
ATO3
SDP1
YGR287C
DPP1
ERV29
YDR278C
YIL015C−A
YCL013W
YCL026C
RNH202
ECM12
YGR290W
DAL4
CPR5
PTK1
HNT2
YCL012W
BUD3
FYV8
AFR1
ENT3
YBR027C
YBR030W
ROM2
PCH2
ARK1
YDR095C
MNL1
YPR011C
SMP1
RBG2
Gene Name
YPD.K.Shift
HL.K.INTERACTION
CSH.K.INTERACTION
HL.AS.K.INTERACTION
YPEG.K.Shift
HLEG.K.INTERACTION
CSHEG.K.INTERACTION
HLEG.AS.K.INTERACTION
YPD.L.SHIFT
HL.L.INTERACTION
CSH.L.INTERACTION
HL.AS.L.INTERACTION
YPEG.L.SHIFT
HLEG.L.INTERACTION
CSHEG.L.INTERACTION
HLEG.AS.L.INTERACTION
Type of Media

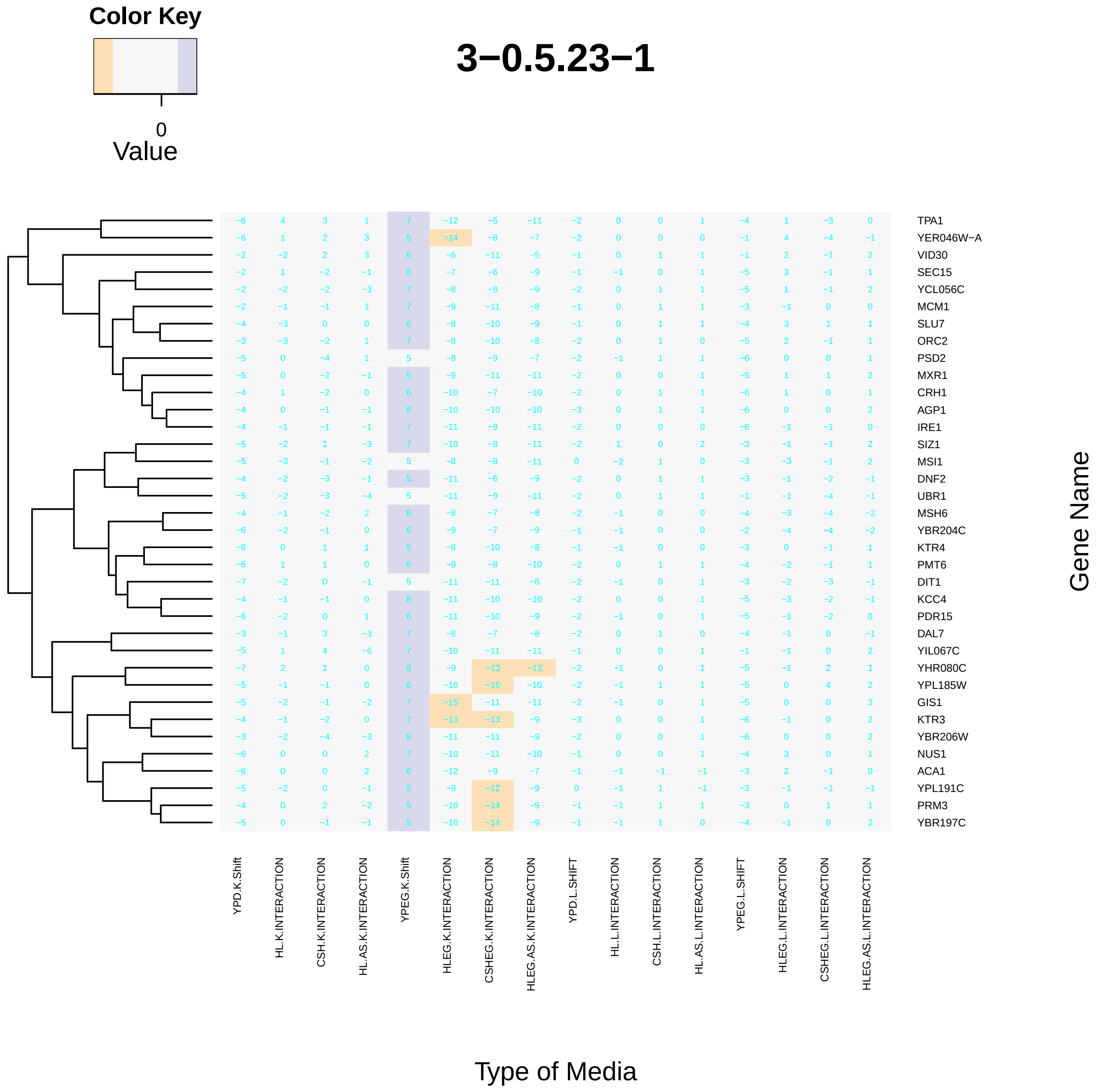
Color Key
0
Value
3−0.5.23−1
YPD.K.Shift HL.K.INTERACTION CSH.K.INTERACTION HL.AS.K.INTERACTION YPEG.K.Shift HLEG.K.INTERACTION CSHEG.K.INTERACTION HLEG.AS.K.INTERACTION YPD.L.SHIFT HL.L.INTERACTION CSH.L.INTERACTION HL.AS.L.INTERACTION YPEG.L.SHIFT HLEG.L.INTERACTION CSHEG.L.INTERACTION HLEG.AS.L.INTERACTION
TPA1 −6 4 3 1 7 −12 −5 −11 −2 0 0 1 −4 1 −3 0
YER046W−A −6 1 2 3 5 −14 −8 −7 −2 0 0 0 −1 4 −4 −1
VID30 −2 −2 2 3 6 −6 −11 −5 −1 0 1 1 −1 2 −1 2
SEC15 −2 1 −2 −1 8 −7 −6 −9 −1 −1 0 1 −5 3 −1 1
YCL056C −2 −2 −2 −3 7 −8 −8 −9 −2 0 1 1 −5 1 −1 2
MCM1 −2 −1 −1 1 7 −9 −11 −8 −1 0 1 1 −3 −1 0 0
SLU7 −4 −3 0 0 6 −8 −10 −9 −1 0 1 1 −4 3 1 1
ORC2 −3 −3 −2 1 7 −8 −10 −8 −2 0 1 0 −5 2 −1 1
PSD2 −5 0 −4 1 5 −8 −9 −7 −2 −1 1 1 −6 0 0 1
MXR1 −5 0 −2 −1 5 −9 −11 −11 −2 0 0 1 −5 1 1 2
CRH1 −4 1 −2 0 6 −10 −7 −10 −2 0 1 1 −6 1 0 1
AGP1 −4 0 −1 −1 8 −10 −10 −10 −3 0 1 1 −6 0 0 2
IRE1 −4 −1 −1 −1 7 −11 −9 −11 −2 0 0 0 −6 −1 −1 0
SIZ1 −5 −2 1 −3 7 −10 −8 −11 −2 1 0 2 −3 −1 −1 2
MSI1 −5 −3 −1 −2 5 −8 −8 −11 0 −2 1 0 −3 −3 −1 2
DNF2 −4 −2 −3 −1 5 −11 −6 −9 −2 0 1 1 −3 −1 −2 −1
UBR1 −5 −2 −3 −4 5 −11 −9 −11 −2 0 1 1 −1 −1 −4 −1
MSH6 −4 −1 −2 2 6 −8 −7 −8 −2 −1 0 0 −4 −3 −4 −2
YBR204C −6 −2 −1 0 6 −9 −7 −9 −1 −1 0 0 −2 −4 −4 −2
KTR4 −6 0 1 1 5 −8 −10 −8 −1 −1 0 0 −3 0 −1 1
PMT6 −6 1 1 0 6 −9 −8 −10 −2 0 1 1 −4 −2 −1 1
DIT1 −7 −2 0 −1 5 −11 −11 −8 −2 −1 0 1 −3 −2 −3 −1
KCC4 −4 −1 −1 0 8 −11 −10 −10 −2 0 0 1 −5 −3 −2 −1
PDR15 −6 −2 0 1 6 −11 −10 −9 −2 −1 0 1 −5 −1 −2 0
DAL7 −3 −1 3 −3 7 −8 −7 −8 −2 0 1 0 −4 −1 0 −1
YIL067C −5 1 4 −6 7 −10 −11 −11 −1 0 0 1 −1 −1 0 2
YHR080C −7 2 1 0 8 −9 −13 −13 −2 −1 0 1 −5 −1 2 1
YPL185W −5 −1 −1 0 6 −10 −15 −10 −2 −1 1 1 −5 0 4 2
GIS1 −5 −2 −1 −2 7 −15 −11 −11 −2 −1 0 1 −5 0 0 3
KTR3 −4 −1 −2 0 7 −13 −13 −9 −3 0 0 1 −6 −1 0 2
YBR206W −3 −2 −4 −3 8 −11 −11 −9 −2 0 0 1 −6 0 0 2
NUS1 −6 0 0 2 7 −10 −11 −10 −1 0 0 1 −4 3 0 1
ACA1 −6 0 0 2 6 −12 −9 −7 −1 −1 −1 −1 −3 2 −1 0
YPL191C −5 −2 0 −1 5 −9 −12 −9 0 −1 1 −1 −3 −1 −1 −1
PRM3 −4 0 2 −2 5 −10 −14 −9 −1 −1 1 1 −3 0 1 1
YBR197C −5 0 −1 −1 5 −10 −14 −9 −1 −1 1 0 −4 −1 0 2
Gene Name
Type of Media

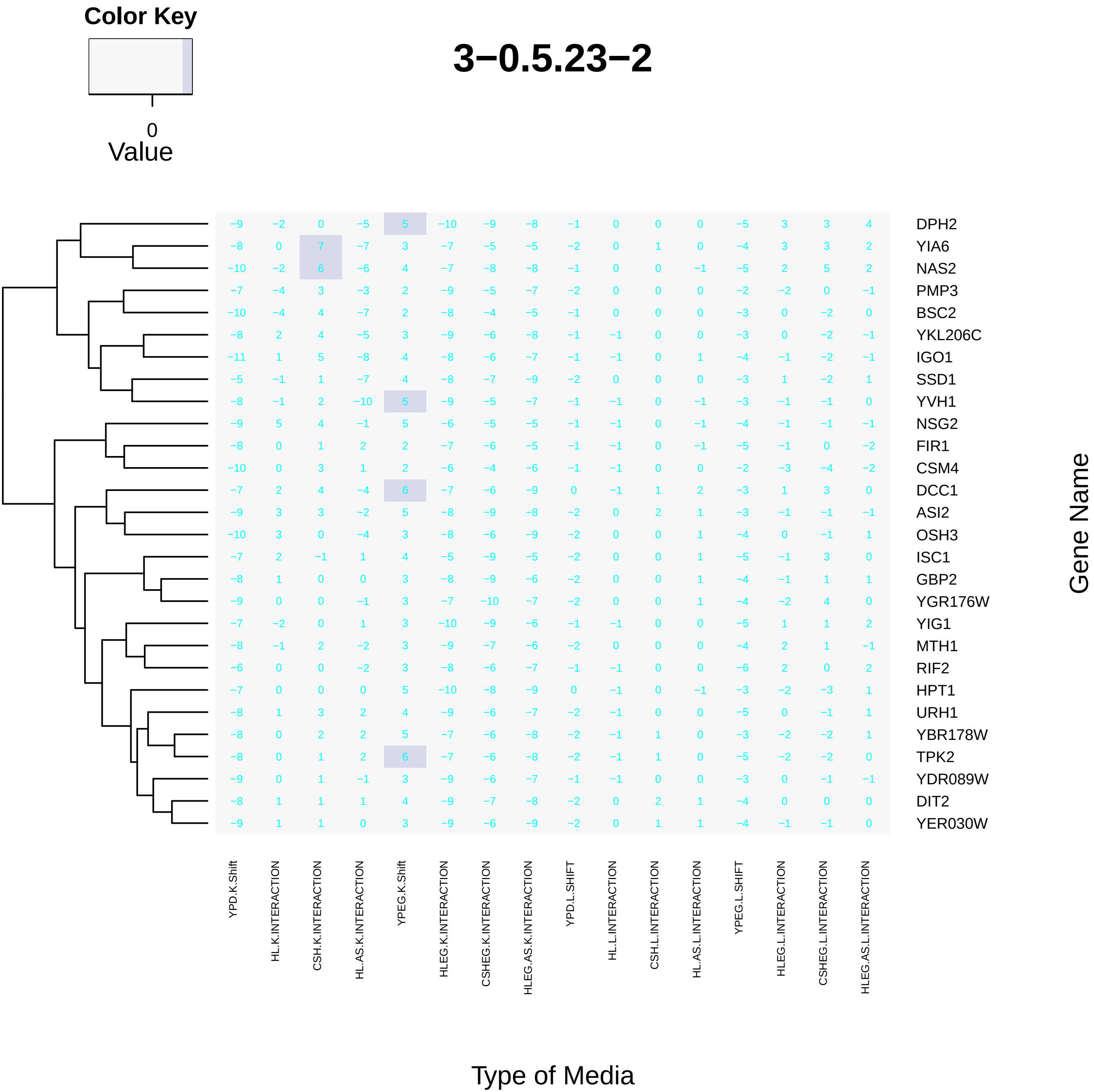
Color Key
0
Value
3−0.5.23−2
YPD.K.Shift HL.K.INTERACTION CSH.K.INTERACTION HL.AS.K.INTERACTION YPEG.K.Shift HLEG.K.INTERACTION CSHEG.K.INTERACTION HLEG.AS.K.INTERACTION YPD.L.SHIFT HL.L.INTERACTION CSH.L.INTERACTION HL.AS.L.INTERACTION YPEG.L.SHIFT HLEG.L.INTERACTION CSHEG.L.INTERACTION HLEG.AS.L.INTERACTION
DPH2 −9 −2 0 −5 5 −10 −9 −8 −1 0 0 0 −5 3 3 4
YIA6 −8 0 7 −7 3 −7 −5 −5 −2 0 1 0 −4 3 3 2
NAS2 −10 −2 6 −6 4 −7 −8 −8 −1 0 0 −1 −5 2 5 2
PMP3 −7 −4 3 −3 2 −9 −5 −7 −2 0 0 0 −2 −2 0 −1
BSC2 −10 −4 4 −7 2 −8 −4 −5 −1 0 0 0 −3 0 −2 0
YKL206C −8 2 4 −5 3 −9 −6 −8 −1 −1 0 0 −3 0 −2 −1
IGO1 −11 1 5 −8 4 −8 −6 −7 −1 −1 0 1 −4 −1 −2 −1
SSD1 −5 −1 1 −7 4 −8 −7 −9 −2 0 0 0 −3 1 −2 1
YVH1 −8 −1 2 −10 5 −9 −5 −7 −1 −1 0 −1 −3 −1 −1 0
NSG2 −9 5 4 −1 5 −6 −5 −5 −1 −1 0 −1 −4 −1 −1 −1
FIR1 −8 0 1 2 2 −7 −6 −5 −1 −1 0 −1 −5 −1 0 −2
CSM4 −10 0 3 1 2 −6 −4 −6 −1 −1 0 0 −2 −3 −4 −2
DCC1 −7 2 4 −4 6 −7 −6 −9 0 −1 1 2 −3 1 3 0
ASI2 −9 3 3 −2 5 −8 −9 −8 −2 0 2 1 −3 −1 −1 −1
OSH3 −10 3 0 −4 3 −8 −6 −9 −2 0 0 1 −4 0 −1 1
ISC1 −7 2 −1 1 4 −5 −9 −5 −2 0 0 1 −5 −1 3 0
GBP2 −8 1 0 0 3 −8 −9 −6 −2 0 0 1 −4 −1 1 1
YGR176W −9 0 0 −1 3 −7 −10 −7 −2 0 0 1 −4 −2 4 0
YIG1 −7 −2 0 1 3 −10 −9 −6 −1 −1 0 0 −5 1 1 2
MTH1 −8 −1 2 −2 3 −9 −7 −6 −2 0 0 0 −4 2 1 −1
RIF2 −6 0 0 −2 3 −8 −6 −7 −1 −1 0 0 −6 2 0 2
HPT1 −7 0 0 0 5 −10 −8 −9 0 −1 0 −1 −3 −2 −3 1
URH1 −8 1 3 2 4 −9 −6 −7 −2 −1 0 0 −5 0 −1 1
YBR178W −8 0 2 2 5 −7 −6 −8 −2 −1 1 0 −3 −2 −2 1
TPK2 −8 0 1 2 6 −7 −6 −8 −2 −1 1 0 −5 −2 −2 0
YDR089W −9 0 1 −1 3 −9 −6 −7 −1 −1 0 0 −3 0 −1 −1
DIT2 −8 1 1 1 4 −9 −7 −8 −2 0 2 1 −4 0 0 0
YER030W −9 1 1 0 3 −9 −6 −9 −2 0 1 1 −4 −1 −1 0
Gene Name
Type of Media

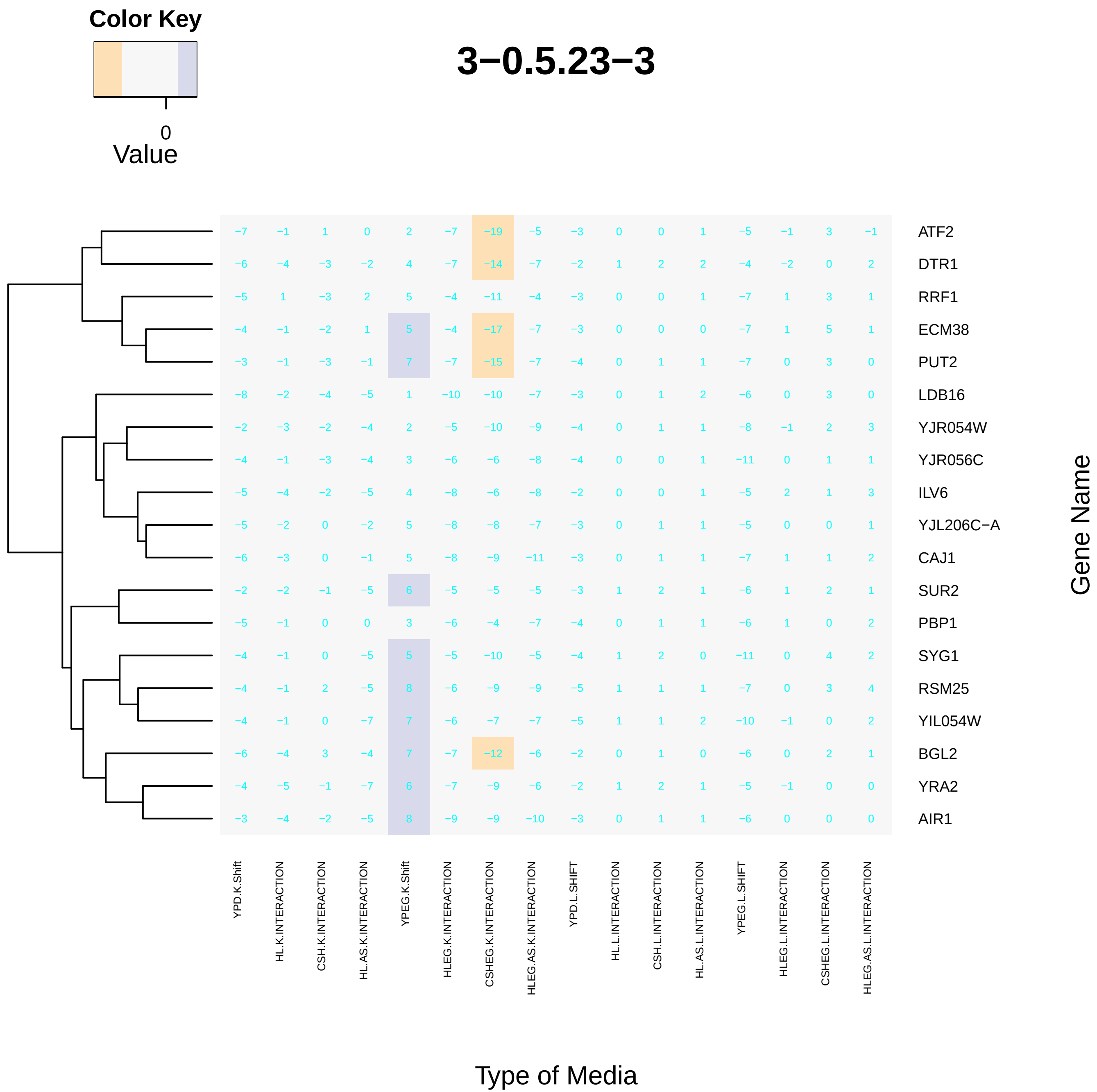
Color Key
0
Value
3−0.5.23−3
ATF2 −7 −1 1 0 2 −7 −19 −5 −3 0 0 1 −5 −1 3 −1
DTR1 −6 −4 −3 −2 4 −7 −14 −7 −2 1 2 2 −4 −2 0 2
RRF1 −5 1 −3 2 5 −4 −11 −4 −3 0 0 1 −7 1 3 1
ECM38 −4 −1 −2 1 5 −4 −17 −7 −3 0 0 0 −7 1 5 1
PUT2 −3 −1 −3 −1 7 −7 −15 −7 −4 0 1 1 −7 0 3 0
LDB16 −8 −2 −4 −5 1 −10 −10 −7 −3 0 1 2 −6 0 3 0
YJR054W −2 −3 −2 −4 2 −5 −10 −9 −4 0 1 1 −8 −1 2 3
YJR056C −4 −1 −3 −4 3 −6 −6 −8 −4 0 0 1 −11 0 1 1
ILV6 −5 −4 −2 −5 4 −8 −6 −8 −2 0 0 1 −5 2 1 3
YJL206C−A −5 −2 0 −2 5 −8 −8 −7 −3 0 1 1 −5 0 0 1
CAJ1 −6 −3 0 −1 5 −8 −9 −11 −3 0 1 1 −7 1 1 2
SUR2 −2 −2 −1 −5 6 −5 −5 −5 −3 1 2 1 −6 1 2 1
PBP1 −5 −1 0 0 3 −6 −4 −7 −4 0 1 1 −6 1 0 2
SYG1 −4 −1 0 −5 5 −5 −10 −5 −4 1 2 0 −11 0 4 2
RSM25 −4 −1 2 −5 8 −6 −9 −9 −5 1 1 1 −7 0 3 4
YIL054W −4 −1 0 −7 7 −6 −7 −7 −5 1 1 2 −10 −1 0 2
BGL2 −6 −4 3 −4 7 −7 −12 −6 −2 0 1 0 −6 0 2 1
YRA2 −4 −5 −1 −7 6 −7 −9 −6 −2 1 2 1 −5 −1 0 0
AIR1 −3 −4 −2 −5 8 −9 −9 −10 −3 0 1 1 −6 0 0 0
Gene Name
YPD.K.Shift
HL.K.INTERACTION
CSH.K.INTERACTION
HL.AS.K.INTERACTION
YPEG.K.Shift
HLEG.K.INTERACTION
CSHEG.K.INTERACTION
HLEG.AS.K.INTERACTION
YPD.L.SHIFT
HL.L.INTERACTION
CSH.L.INTERACTION
HL.AS.L.INTERACTION
YPEG.L.SHIFT
HLEG.L.INTERACTION
CSHEG.L.INTERACTION
HLEG.AS.L.INTERACTION
Type of Media

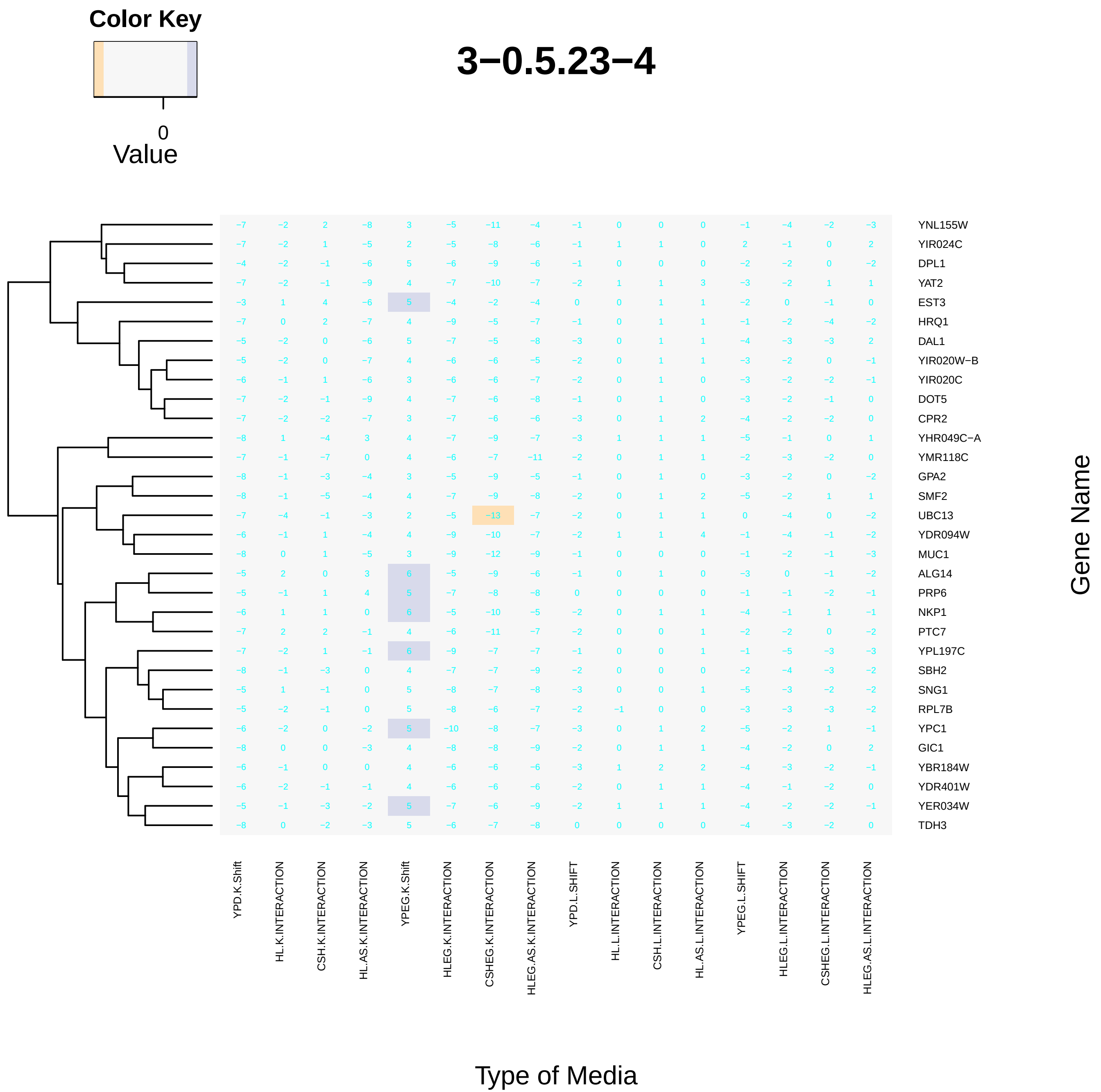
Color Key
0
Value
3−0.5.23−4
−7 −2 2 −8 3 −5 −11 −4 −1 0 0 0 −1 −4 −2 −3 YNL155W
−7 −2 1 −5 2 −5 −8 −6 −1 1 1 0 2 −1 0 2 YIR024C
−4 −2 −1 −6 5 −6 −9 −6 −1 0 0 0 −2 −2 0 −2 DPL1
−7 −2 −1 −9 4 −7 −10 −7 −2 1 1 3 −3 −2 1 1 YAT2
−3 1 4 −6 5 −4 −2 −4 0 0 1 1 −2 0 −1 0 EST3
−7 0 2 −7 4 −9 −5 −7 −1 0 1 1 −1 −2 −4 −2 HRQ1
−5 −2 0 −6 5 −7 −5 −8 −3 0 1 1 −4 −3 −3 2 DAL1
−5 −2 0 −7 4 −6 −6 −5 −2 0 1 1 −3 −2 0 −1 YIR020W−B
−6 −1 1 −6 3 −6 −6 −7 −2 0 1 0 −3 −2 −2 −1 YIR020C
−7 −2 −1 −9 4 −7 −6 −8 −1 0 1 0 −3 −2 −1 0 DOT5
−7 −2 −2 −7 3 −7 −6 −6 −3 0 1 2 −4 −2 −2 0 CPR2
−8 1 −4 3 4 −7 −9 −7 −3 1 1 1 −5 −1 0 1 YHR049C−A
−7 −1 −7 0 4 −6 −7 −11 −2 0 1 1 −2 −3 −2 0 YMR118C
−8 −1 −3 −4 3 −5 −9 −5 −1 0 1 0 −3 −2 0 −2 GPA2
−8 −1 −5 −4 4 −7 −9 −8 −2 0 1 2 −5 −2 1 1 SMF2
−7 −4 −1 −3 2 −5 −13 −7 −2 0 1 1 0 −4 0 −2 UBC13
−6 −1 1 −4 4 −9 −10 −7 −2 1 1 4 −1 −4 −1 −2 YDR094W
−8 0 1 −5 3 −9 −12 −9 −1 0 0 0 −1 −2 −1 −3 MUC1
−5 2 0 3 6 −5 −9 −6 −1 0 1 0 −3 0 −1 −2 ALG14
−5 −1 1 4 5 −7 −8 −8 0 0 0 0 −1 −1 −2 −1 PRP6
−6 1 1 0 6 −5 −10 −5 −2 0 1 1 −4 −1 1 −1 NKP1
−7 2 2 −1 4 −6 −11 −7 −2 0 0 1 −2 −2 0 −2 PTC7
−7 −2 1 −1 6 −9 −7 −7 −1 0 0 1 −1 −5 −3 −3 YPL197C
−8 −1 −3 0 4 −7 −7 −9 −2 0 0 0 −2 −4 −3 −2 SBH2
−5 1 −1 0 5 −8 −7 −8 −3 0 0 1 −5 −3 −2 −2 SNG1
−5 −2 −1 0 5 −8 −6 −7 −2 −1 0 0 −3 −3 −3 −2 RPL7B
−6 −2 0 −2 5 −10 −8 −7 −3 0 1 2 −5 −2 1 −1 YPC1
−8 0 0 −3 4 −8 −8 −9 −2 0 1 1 −4 −2 0 2 GIC1
−6 −1 0 0 4 −6 −6 −6 −3 1 2 2 −4 −3 −2 −1 YBR184W
−6 −2 −1 −1 4 −6 −6 −6 −2 0 1 1 −4 −1 −2 0 YDR401W
−5 −1 −3 −2 5 −7 −6 −9 −2 1 1 1 −4 −2 −2 −1 YER034W
−8 0 −2 −3 5 −6 −7 −8 0 0 0 0 −4 −3 −2 0 TDH3
YPD.K.Shift
HL.K.INTERACTION
CSH.K.INTERACTION
HL.AS.K.INTERACTION
YPEG.K.Shift
HLEG.K.INTERACTION
CSHEG.K.INTERACTION
HLEG.AS.K.INTERACTION
YPD.L.SHIFT
HL.L.INTERACTION
CSH.L.INTERACTION
HL.AS.L.INTERACTION
YPEG.L.SHIFT
HLEG.L.INTERACTION
CSHEG.L.INTERACTION
HLEG.AS.L.INTERACTION
Gene Name
Type of Media

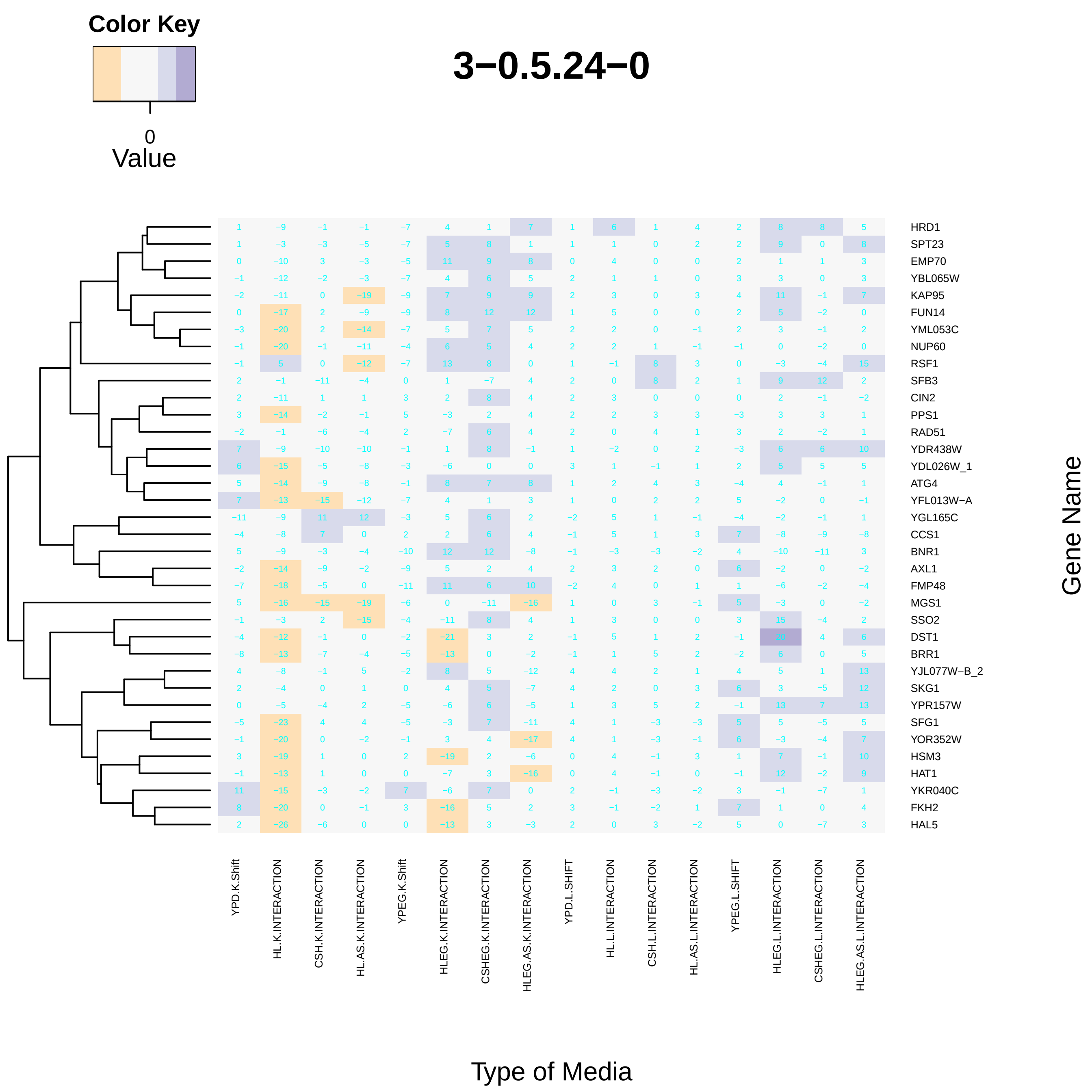
3−0.5.24−0
Color Key
0
Value
HRD1
SPT23
EMP70
YBL065W
KAP95
FUN14
YML053C
NUP60
RSF1
SFB3
CIN2
PPS1
RAD51
YDR438W
YDL026W_1
ATG4
YFL013W−A
YGL165C
CCS1
BNR1
AXL1
FMP48
MGS1
SSO2
DST1
BRR1
YJL077W−B_2
SKG1
YPR157W
SFG1
YOR352W
HSM3
HAT1
YKR040C
FKH2
HAL5
Gene Name
YPD.K.Shift
HL.K.INTERACTION
CSH.K.INTERACTION
HL.AS.K.INTERACTION
YPEG.K.Shift
HLEG.K.INTERACTION
CSHEG.K.INTERACTION
HLEG.AS.K.INTERACTION
YPD.L.SHIFT
HL.L.INTERACTION
CSH.L.INTERACTION
HL.AS.L.INTERACTION
YPEG.L.SHIFT
HLEG.L.INTERACTION
CSHEG.L.INTERACTION
HLEG.AS.L.INTERACTION
Type of Media

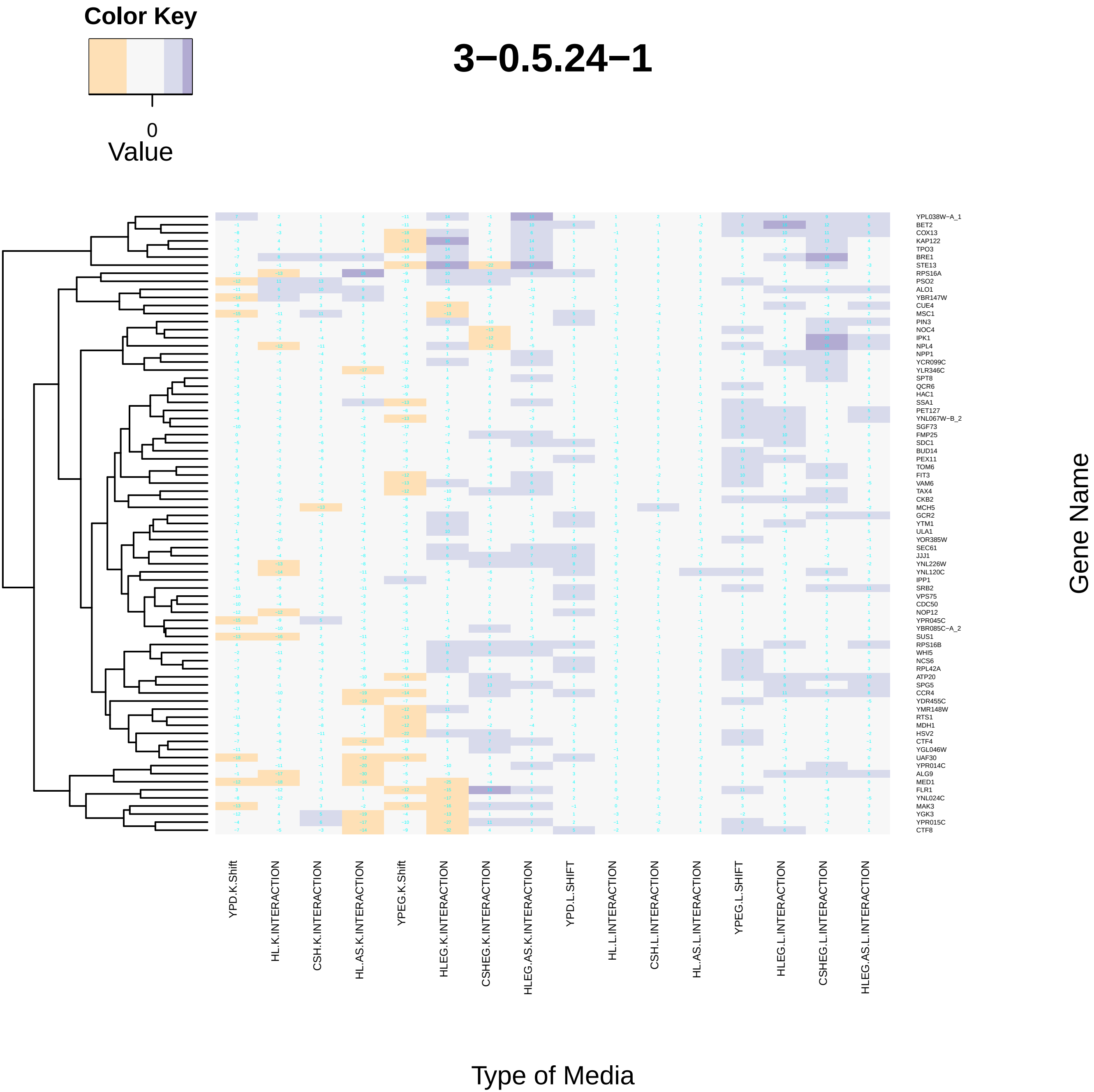
3−0.5.24−1
Color Key
0
Value
YPL038W−A_1
BET2
COX13
KAP122
TPO3
BRE1
STE13
RPS16A
PSO2
ALO1
YBR147W
CUE4
MSC1
PIN3
NOC4
IPK1
NPL4
NPP1
YCR099C
YLR346C
SPT8
QCR6
HAC1
SSA1
PET127
YNL067W−B_2
SGF73
FMP25
SDC1
BUD14
PEX11
TOM6
FIT3
VAM6
TAX4
CKB2
MCH5
GCR2
YTM1
ULA1
YOR385W
SEC61
JJJ1
YNL226W
YNL120C
IPP1
SRB2
VPS75
CDC50
NOP12
YPR045C
YBR085C−A_2
SUS1
RPS16B
WHI5
NCS6
RPL42A
ATP20
SPG5
CCR4
YDR455C
YMR148W
RTS1
MDH1
HSV2
CTF4
YGL046W
UAF30
YPR014C
ALG9
MED1
FLR1
YNL024C
MAK3
YGK3
YPR015C
CTF8
Gene Name
YPD.K.Shift
HL.K.INTERACTION
CSH.K.INTERACTION
HL.AS.K.INTERACTION
YPEG.K.Shift
HLEG.K.INTERACTION
CSHEG.K.INTERACTION
HLEG.AS.K.INTERACTION
YPD.L.SHIFT
HL.L.INTERACTION
CSH.L.INTERACTION
HL.AS.L.INTERACTION
YPEG.L.SHIFT
HLEG.L.INTERACTION
CSHEG.L.INTERACTION
HLEG.AS.L.INTERACTION
Type of Media

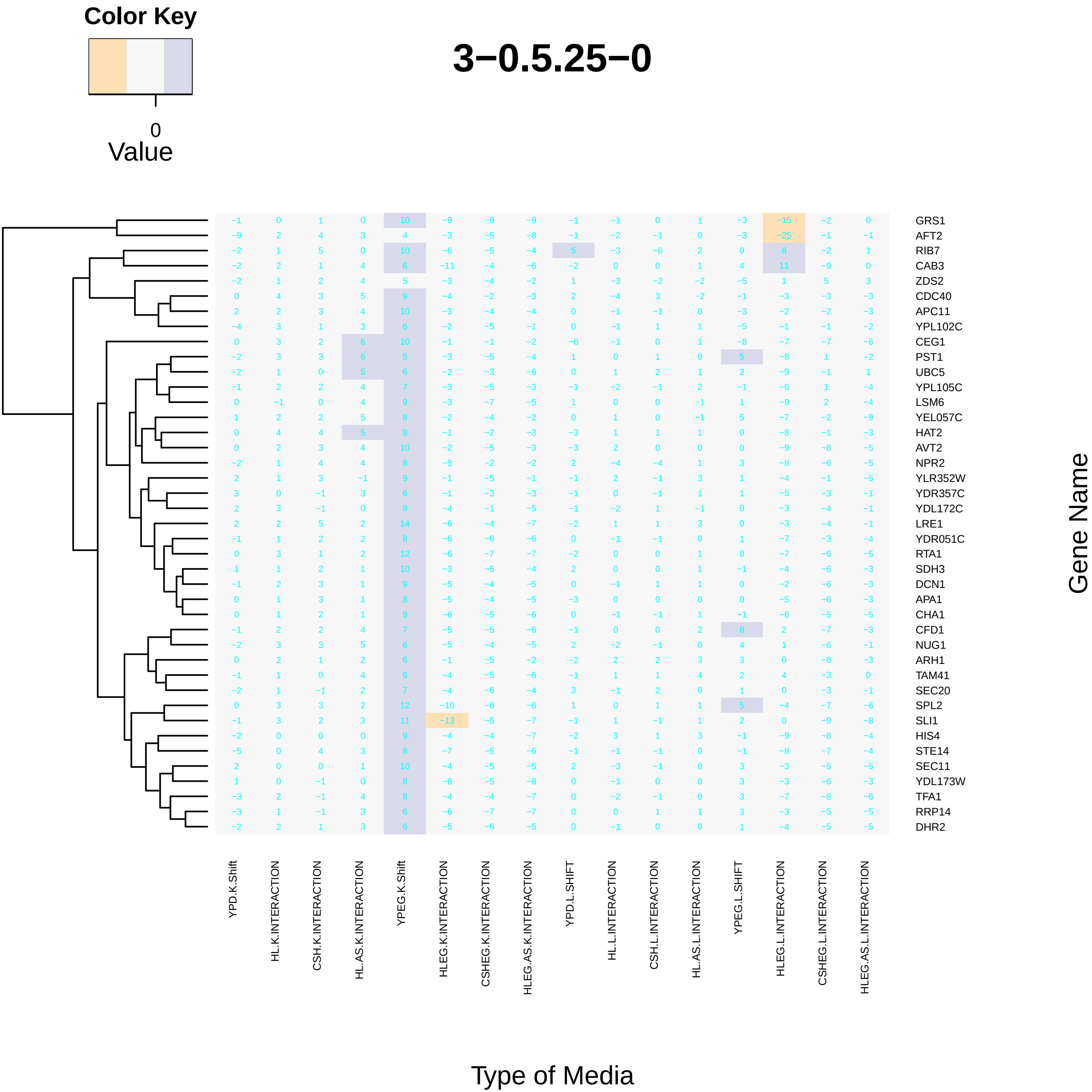
Color Key
0
Value
3−0.5.25−0
GRS1
AFT2
RIB7
CAB3
ZDS2
CDC40
APC11
YPL102C
CEG1
PST1
UBC5
YPL105C
LSM6
YEL057C
HAT2
AVT2
NPR2
YLR352W
YDR357C
YDL172C
LRE1
YDR051C
RTA1
SDH3
DCN1
APA1
CHA1
CFD1
NUG1
ARH1
TAM41
SEC20
SPL2
SLI1
HIS4
STE14
SEC11
YDL173W
TFA1
RRP14
DHR2
Gene Name
YPD.K.Shift
HL.K.INTERACTION
CSH.K.INTERACTION
HL.AS.K.INTERACTION
YPEG.K.Shift
HLEG.K.INTERACTION
CSHEG.K.INTERACTION
HLEG.AS.K.INTERACTION
YPD.L.SHIFT
HL.L.INTERACTION
CSH.L.INTERACTION
HL.AS.L.INTERACTION
YPEG.L.SHIFT
HLEG.L.INTERACTION
CSHEG.L.INTERACTION
HLEG.AS.L.INTERACTION
Type of Media

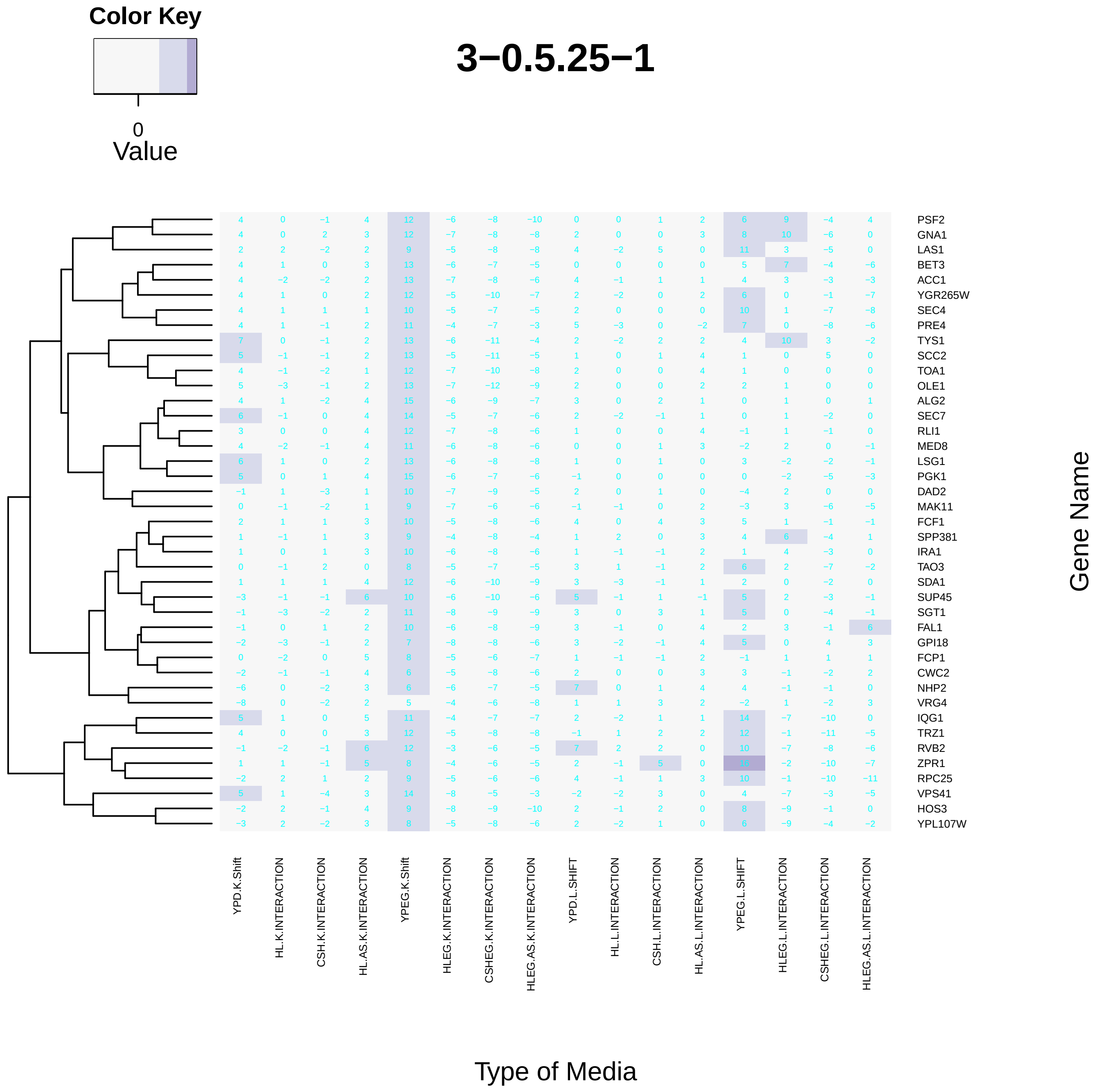
Color Key
0
Value
3−0.5.25−1
PSF2
GNA1
LAS1
BET3
ACC1
YGR265W
SEC4
PRE4
TYS1
SCC2
TOA1
OLE1
ALG2
SEC7
RLI1
MED8
LSG1
PGK1
DAD2
MAK11
FCF1
SPP381
IRA1
TAO3
SDA1
SUP45
SGT1
FAL1
GPI18
FCP1
CWC2
NHP2
VRG4
IQG1
TRZ1
RVB2
ZPR1
RPC25
VPS41
HOS3
YPL107W
Gene Name
YPD.K.Shift
HL.K.INTERACTION
CSH.K.INTERACTION
HL.AS.K.INTERACTION
YPEG.K.Shift
HLEG.K.INTERACTION
CSHEG.K.INTERACTION
HLEG.AS.K.INTERACTION
YPD.L.SHIFT
HL.L.INTERACTION
CSH.L.INTERACTION
HL.AS.L.INTERACTION
YPEG.L.SHIFT
HLEG.L.INTERACTION
CSHEG.L.INTERACTION
HLEG.AS.L.INTERACTION
Type of Media

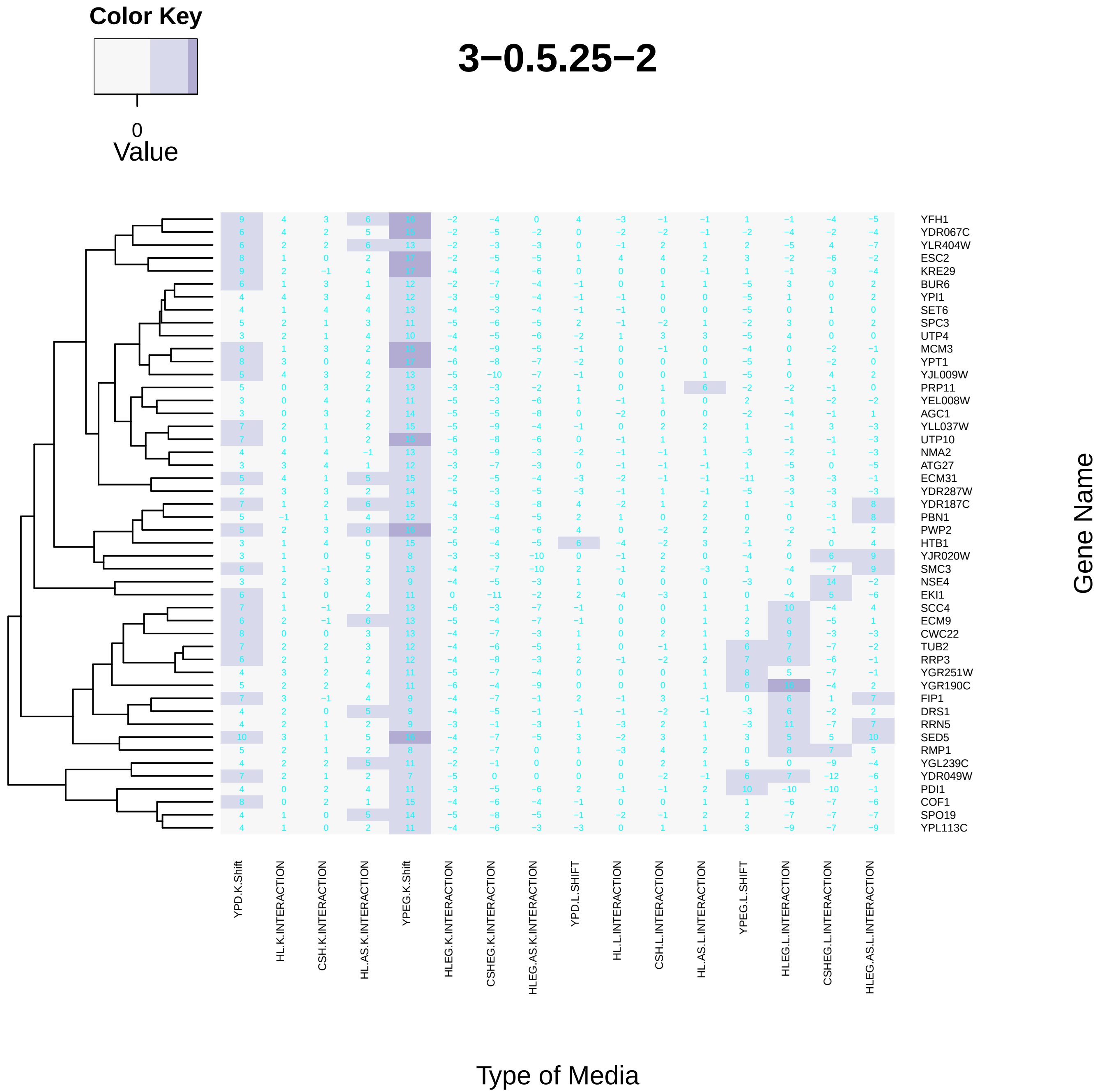

Color Key
0
Value
3−0.5.25−2
YFH1
YDR067C
YLR404W
ESC2
KRE29
BUR6
YPI1
SET6
SPC3
UTP4
MCM3
YPT1
YJL009W
PRP11
YEL008W
AGC1
YLL037W
UTP10
NMA2
ATG27
ECM31
YDR287W
YDR187C
PBN1
PWP2
HTB1
YJR020W
SMC3
NSE4
EKI1
SCC4
ECM9
CWC22
TUB2
RRP3
YGR251W
YGR190C
FIP1
DRS1
RRN5
SED5
RMP1
YGL239C
YDR049W
PDI1
COF1
SPO19
YPL113C
Gene Name
YPD.K.Shift
HL.K.INTERACTION
CSH.K.INTERACTION
HL.AS.K.INTERACTION
YPEG.K.Shift
HLEG.K.INTERACTION
CSHEG.K.INTERACTION
HLEG.AS.K.INTERACTION
YPD.L.SHIFT
HL.L.INTERACTION
CSH.L.INTERACTION
HL.AS.L.INTERACTION
YPEG.L.SHIFT
HLEG.L.INTERACTION
CSHEG.L.INTERACTION
HLEG.AS.L.INTERACTION
Type of Media

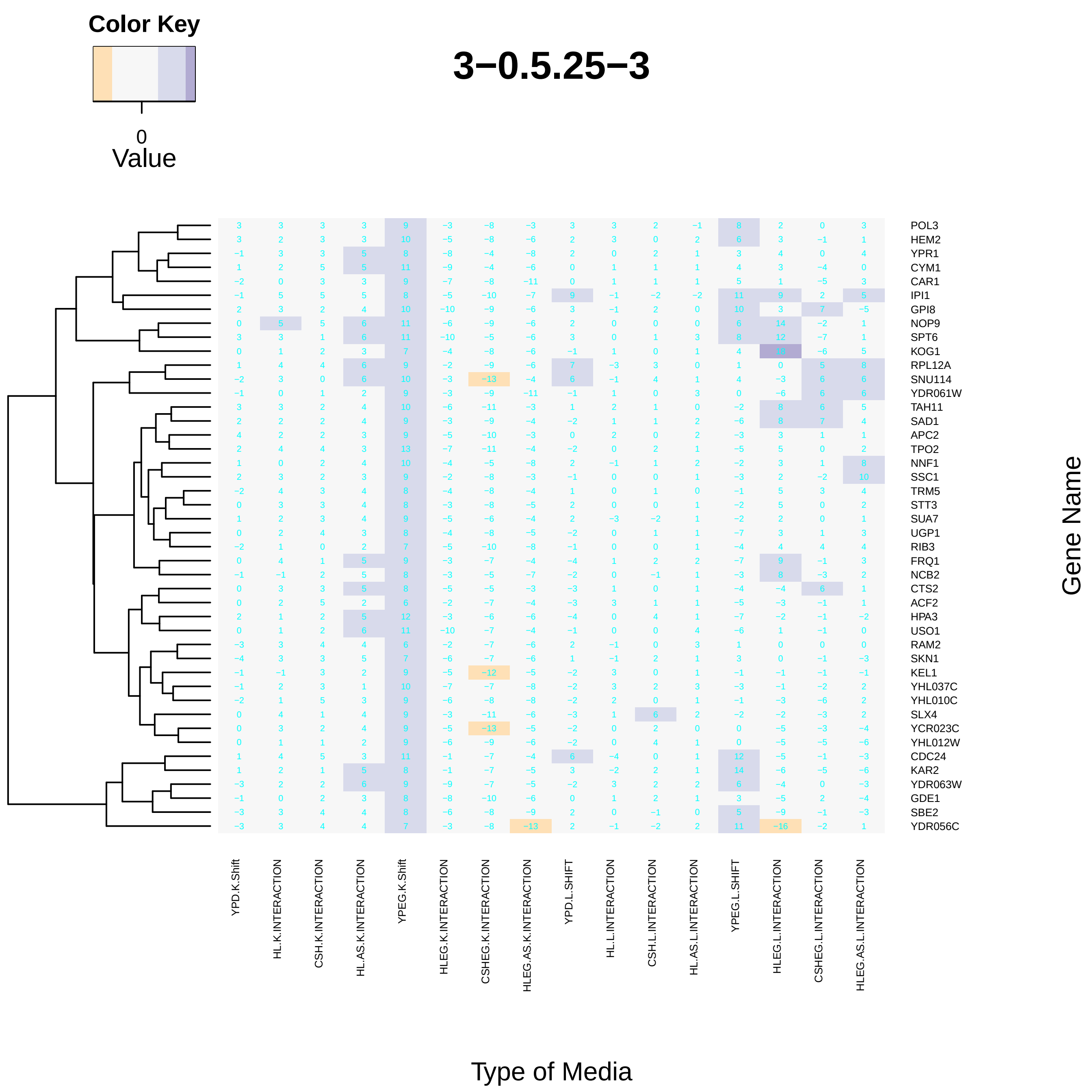
Color Key
0
Value
3−0.5.25−3
POL3
HEM2
YPR1
CYM1
CAR1
IPI1
GPI8
NOP9
SPT6
KOG1
RPL12A
SNU114
YDR061W
TAH11
SAD1
APC2
TPO2
NNF1
SSC1
TRM5
STT3
SUA7
UGP1
RIB3
FRQ1
NCB2
CTS2
ACF2
HPA3
USO1
RAM2
SKN1
KEL1
YHL037C
YHL010C
SLX4
YCR023C
YHL012W
CDC24
KAR2
YDR063W
GDE1
SBE2
YDR056C
Gene Name
YPD.K.Shift
HL.K.INTERACTION
CSH.K.INTERACTION
HL.AS.K.INTERACTION
YPEG.K.Shift
HLEG.K.INTERACTION
CSHEG.K.INTERACTION
HLEG.AS.K.INTERACTION
YPD.L.SHIFT
HL.L.INTERACTION
CSH.L.INTERACTION
HL.AS.L.INTERACTION
YPEG.L.SHIFT
HLEG.L.INTERACTION
CSHEG.L.INTERACTION
HLEG.AS.L.INTERACTION
Type of Media

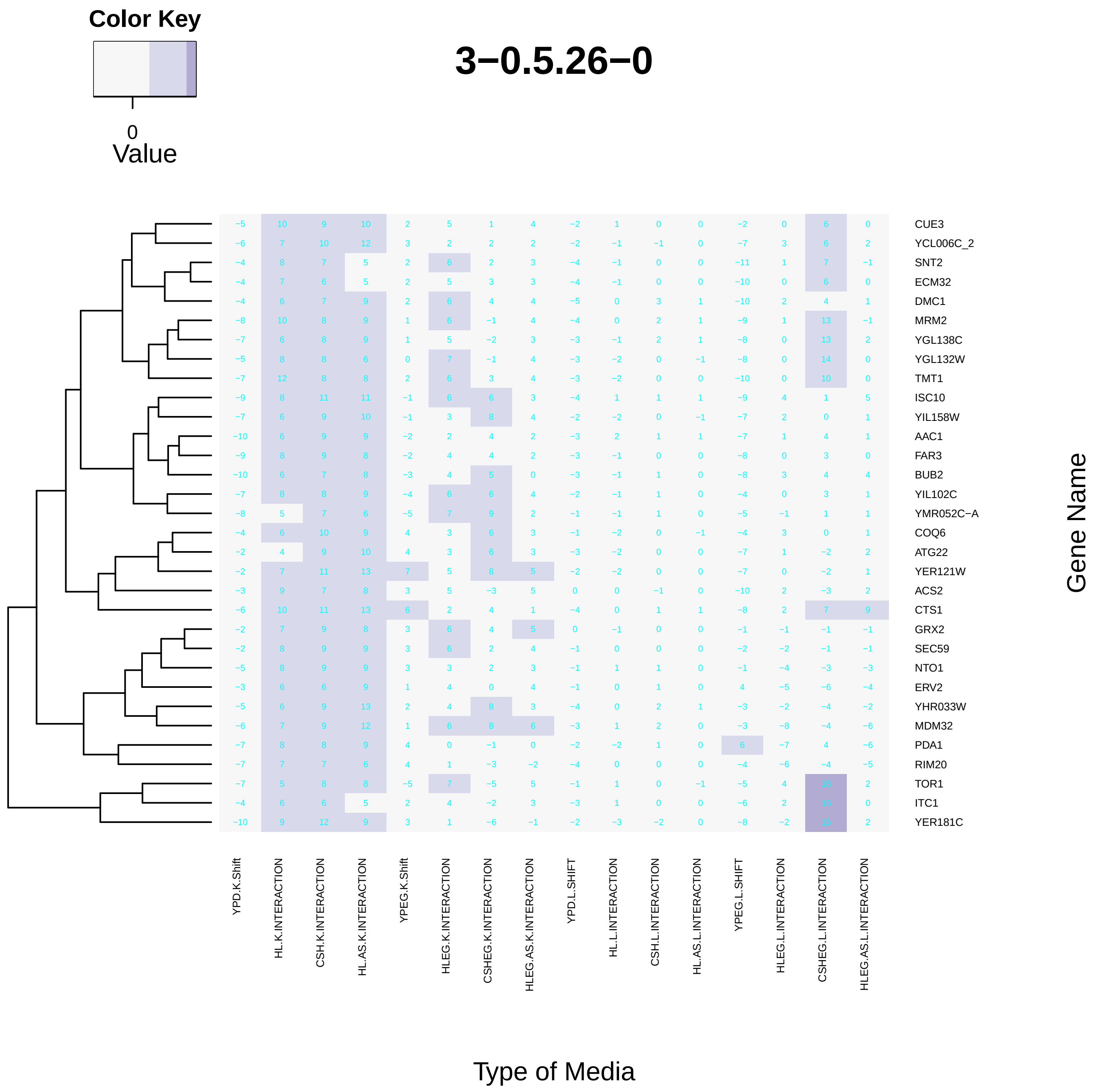
Color Key
0
Value
3−0.5.26−0
YPD.K.Shift
HL.K.INTERACTION
CSH.K.INTERACTION
HL.AS.K.INTERACTION
YPEG.K.Shift
HLEG.K.INTERACTION
CSHEG.K.INTERACTION
HLEG.AS.K.INTERACTION
YPD.L.SHIFT
HL.L.INTERACTION
CSH.L.INTERACTION
HL.AS.L.INTERACTION
YPEG.L.SHIFT
HLEG.L.INTERACTION
CSHEG.L.INTERACTION
HLEG.AS.L.INTERACTION
CUE3
YCL006C_2
SNT2
ECM32
DMC1
MRM2
YGL138C
YGL132W
TMT1
ISC10
YIL158W
AAC1
FAR3
BUB2
YIL102C
YMR052C−A
COQ6
ATG22
YER121W
ACS2
CTS1
GRX2
SEC59
NTO1
ERV2
YHR033W
MDM32
PDA1
RIM20
TOR1
ITC1
YER181C
Gene Name
Type of Media

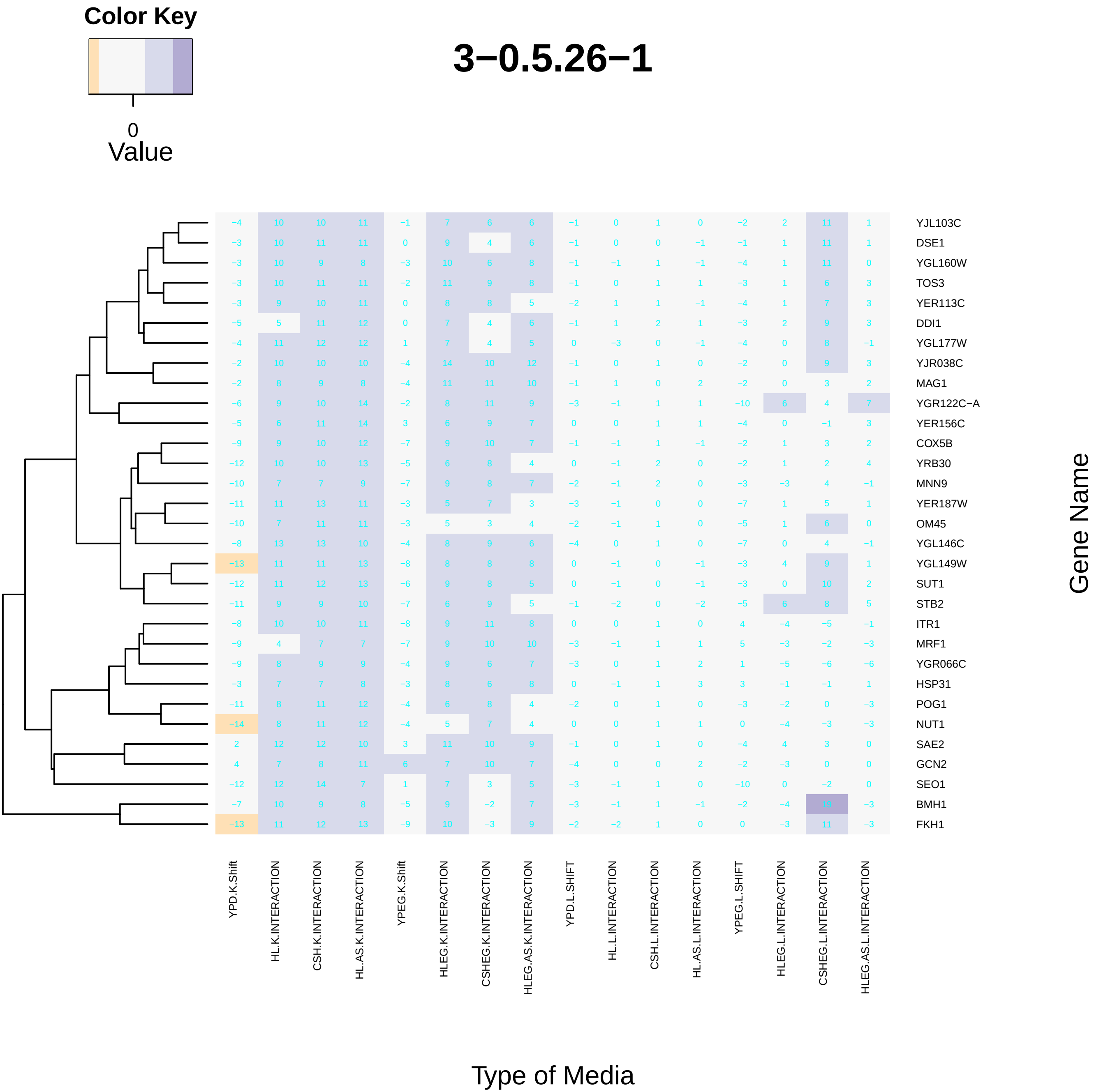

Color Key
0
Value
3−0.5.26−1
YJL103C
DSE1
YGL160W
TOS3
YER113C
DDI1
YGL177W
YJR038C
MAG1
YGR122C−A
YER156C
COX5B
YRB30
MNN9
YER187W
OM45
YGL146C
YGL149W
SUT1
STB2
ITR1
MRF1
YGR066C
HSP31
POG1
NUT1
SAE2
GCN2
SEO1
BMH1
FKH1
Gene Name
YPD.K.Shift
HL.K.INTERACTION
CSH.K.INTERACTION
HL.AS.K.INTERACTION
YPEG.K.Shift
HLEG.K.INTERACTION
CSHEG.K.INTERACTION
HLEG.AS.K.INTERACTION
YPD.L.SHIFT
HL.L.INTERACTION
CSH.L.INTERACTION
HL.AS.L.INTERACTION
YPEG.L.SHIFT
HLEG.L.INTERACTION
CSHEG.L.INTERACTION
HLEG.AS.L.INTERACTION
Type of Media

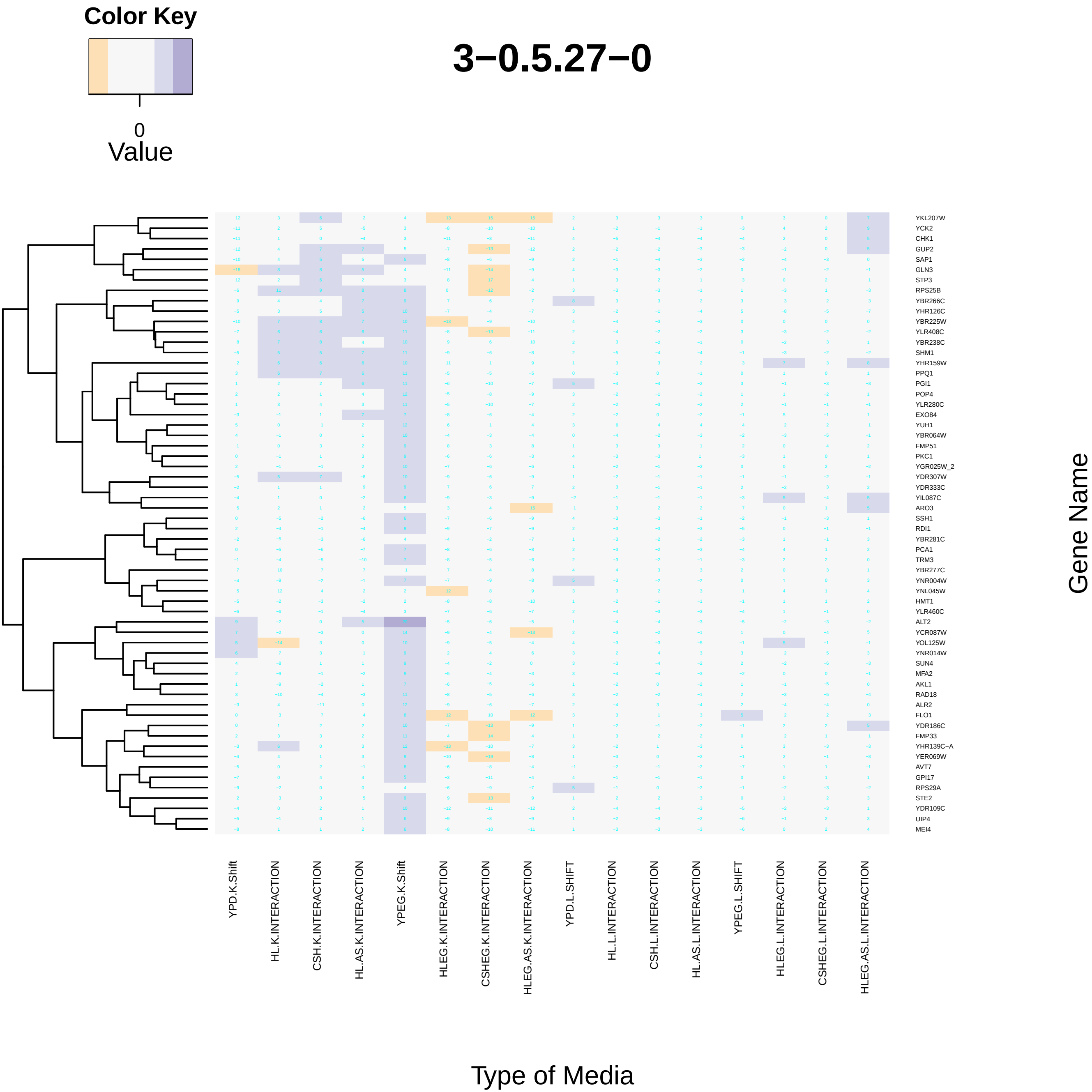
3−0.5.27−0
Color Key
0
Value
YKL207W
YCK2
CHK1
GUP2
SAP1
GLN3
STP3
RPS25B
YBR266C
YHR126C
YBR225W
YLR408C
YBR238C
SHM1
YHR159W
PPQ1
PGI1
POP4
YLR280C
EXO84
YUH1
YBR064W
FMP51
PKC1
YGR025W_2
YDR307W
YDR333C
YIL087C
ARO3
SSH1
RDI1
YBR281C
PCA1
TRM3
YBR277C
YNR004W
YNL045W
HMT1
YLR460C
ALT2
YCR087W
YOL125W
YNR014W
SUN4
MFA2
AKL1
RAD18
ALR2
FLO1
YDR186C
FMP33
YHR139C−A
YER069W
AVT7
GPI17
RPS29A
STE2
YDR109C
UIP4
MEI4
Gene Name
YPD.K.Shift
HL.K.INTERACTION
CSH.K.INTERACTION
HL.AS.K.INTERACTION
YPEG.K.Shift
HLEG.K.INTERACTION
CSHEG.K.INTERACTION
HLEG.AS.K.INTERACTION
YPD.L.SHIFT
HL.L.INTERACTION
CSH.L.INTERACTION
HL.AS.L.INTERACTION
YPEG.L.SHIFT
HLEG.L.INTERACTION
CSHEG.L.INTERACTION
HLEG.AS.L.INTERACTION
Type of Media

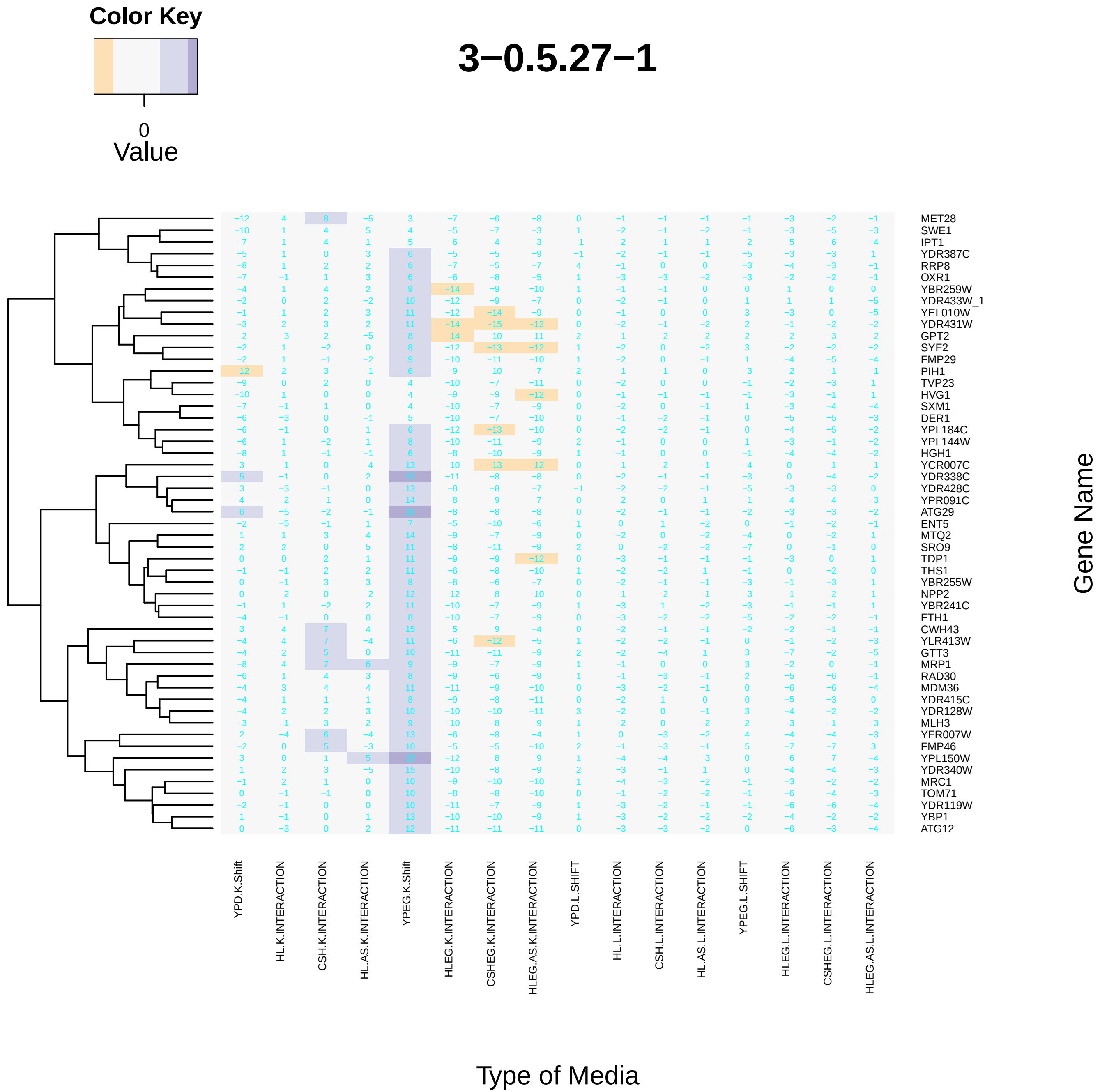
Color Key
0
Value
3−0.5.27−1
MET28
SWE1
IPT1
YDR387C
RRP8
OXR1
YBR259W
YDR433W_1
YEL010W
YDR431W
GPT2
SYF2
FMP29
PIH1
TVP23
HVG1
SXM1
DER1
YPL184C
YPL144W
HGH1
YCR007C
YDR338C
YDR428C
YPR091C
ATG29
ENT5
MTQ2
SRO9
TDP1
THS1
YBR255W
NPP2
YBR241C
FTH1
CWH43
YLR413W
GTT3
MRP1
RAD30
MDM36
YDR415C
YDR128W
MLH3
YFR007W
FMP46
YPL150W
YDR340W
MRC1
TOM71
YDR119W
YBP1
ATG12
Gene Name
YPD.K.Shift
HL.K.INTERACTION
CSH.K.INTERACTION
HL.AS.K.INTERACTION
YPEG.K.Shift
HLEG.K.INTERACTION
CSHEG.K.INTERACTION
HLEG.AS.K.INTERACTION
YPD.L.SHIFT
HL.L.INTERACTION
CSH.L.INTERACTION
HL.AS.L.INTERACTION
YPEG.L.SHIFT
HLEG.L.INTERACTION
CSHEG.L.INTERACTION
HLEG.AS.L.INTERACTION
Type of Media

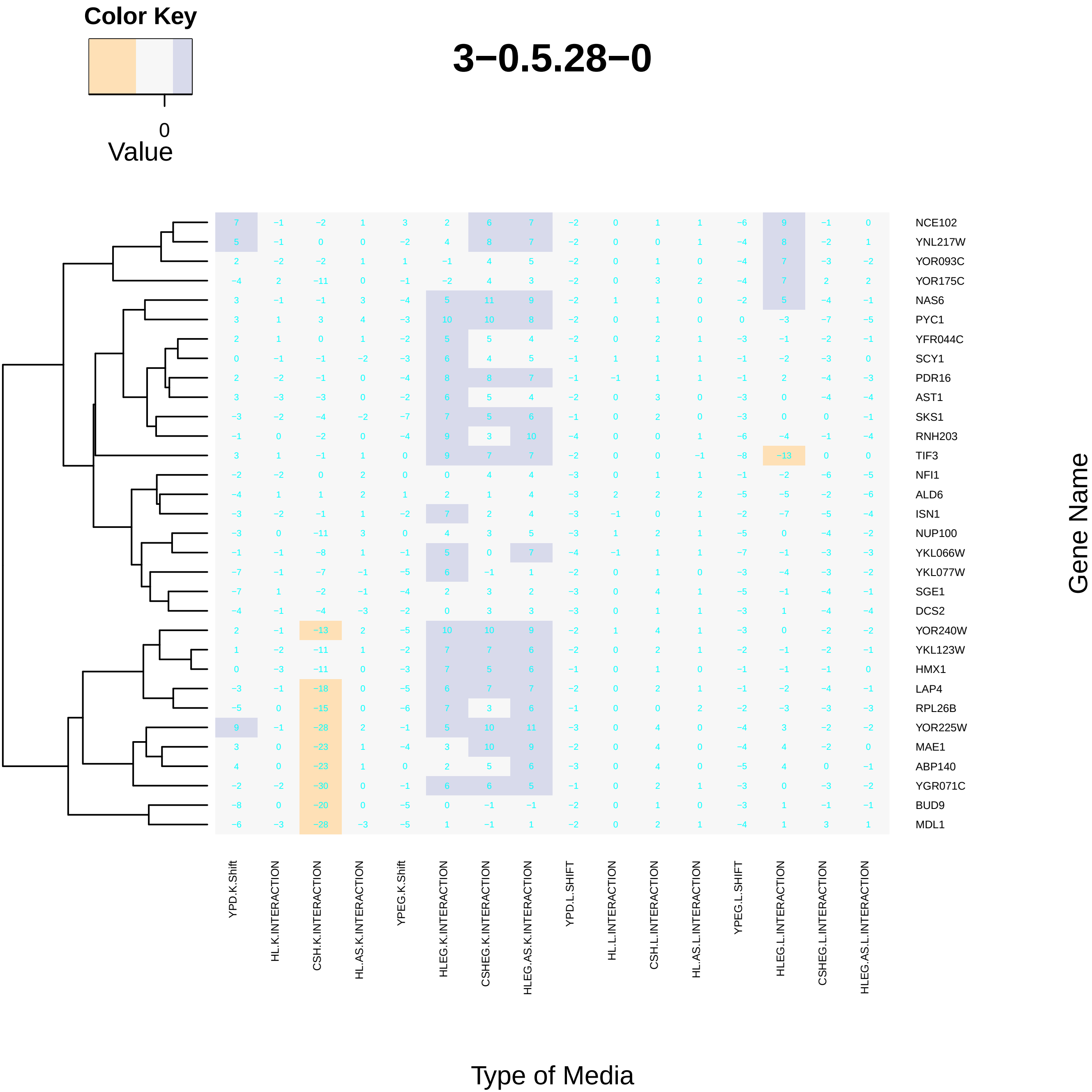
Color Key
0
Value
3−0.5.28−0
7 −1 −2 1 3 2 6 7 −2 0 1 1 −6 9 −1 0 NCE102
5 −1 0 0 −2 4 8 7 −2 0 0 1 −4 8 −2 1 YNL217W
2 −2 −2 1 1 −1 4 5 −2 0 1 0 −4 7 −3 −2 YOR093C
−4 2 −11 0 −1 −2 4 3 −2 0 3 2 −4 7 2 2 YOR175C
3 −1 −1 3 −4 5 11 9 −2 1 1 0 −2 5 −4 −1 NAS6
3 1 3 4 −3 10 10 8 −2 0 1 0 0 −3 −7 −5 PYC1
2 1 0 1 −2 5 5 4 −2 0 2 1 −3 −1 −2 −1 YFR044C
0 −1 −1 −2 −3 6 4 5 −1 1 1 1 −1 −2 −3 0 SCY1
2 −2 −1 0 −4 8 8 7 −1 −1 1 1 −1 2 −4 −3 PDR16
3 −3 −3 0 −2 6 5 4 −2 0 3 0 −3 0 −4 −4 AST1
−3 −2 −4 −2 −7 7 5 6 −1 0 2 0 −3 0 0 −1 SKS1
−1 0 −2 0 −4 9 3 10 −4 0 0 1 −6 −4 −1 −4 RNH203
3 1 −1 1 0 9 7 7 −2 0 0 −1 −8 −13 0 0 TIF3
−2 −2 0 2 0 0 4 4 −3 0 1 1 −1 −2 −6 −5 NFI1
−4 1 1 2 1 2 1 4 −3 2 2 2 −5 −5 −2 −6 ALD6
−3 −2 −1 1 −2 7 2 4 −3 −1 0 1 −2 −7 −5 −4 ISN1
−3 0 −11 3 0 4 3 5 −3 1 2 1 −5 0 −4 −2 NUP100
−1 −1 −8 1 −1 5 0 7 −4 −1 1 1 −7 −1 −3 −3 YKL066W
−7 −1 −7 −1 −5 6 −1 1 −2 0 1 0 −3 −4 −3 −2 YKL077W
−7 1 −2 −1 −4 2 3 2 −3 0 4 1 −5 −1 −4 −1 SGE1
−4 −1 −4 −3 −2 0 3 3 −3 0 1 1 −3 1 −4 −4 DCS2
2 −1 −13 2 −5 10 10 9 −2 1 4 1 −3 0 −2 −2 YOR240W
1 −2 −11 1 −2 7 7 6 −2 0 2 1 −2 −1 −2 −1 YKL123W
0 −3 −11 0 −3 7 5 6 −1 0 1 0 −1 −1 −1 0 HMX1
−3 −1 −18 0 −5 6 7 7 −2 0 2 1 −1 −2 −4 −1 LAP4
−5 0 −15 0 −6 7 3 6 −1 0 0 2 −2 −3 −3 −3 RPL26B
9 −1 −28 2 −1 5 10 11 −3 0 4 0 −4 3 −2 −2 YOR225W
3 0 −23 1 −4 3 10 9 −2 0 4 0 −4 4 −2 0 MAE1
4 0 −23 1 0 2 5 6 −3 0 4 0 −5 4 0 −1 ABP140
−2 −2 −30 0 −1 6 6 5 −1 0 2 1 −3 0 −3 −2 YGR071C
−8 0 −20 0 −5 0 −1 −1 −2 0 1 0 −3 1 −1 −1 BUD9
−6 −3 −28 −3 −5 1 −1 1 −2 0 2 1 −4 1 3 1 MDL1
YPD.K.Shift
HL.K.INTERACTION
CSH.K.INTERACTION
HL.AS.K.INTERACTION
YPEG.K.Shift
HLEG.K.INTERACTION
CSHEG.K.INTERACTION
HLEG.AS.K.INTERACTION
YPD.L.SHIFT
HL.L.INTERACTION
CSH.L.INTERACTION
HL.AS.L.INTERACTION
YPEG.L.SHIFT
HLEG.L.INTERACTION
CSHEG.L.INTERACTION
HLEG.AS.L.INTERACTION
Gene Name
Type of Media

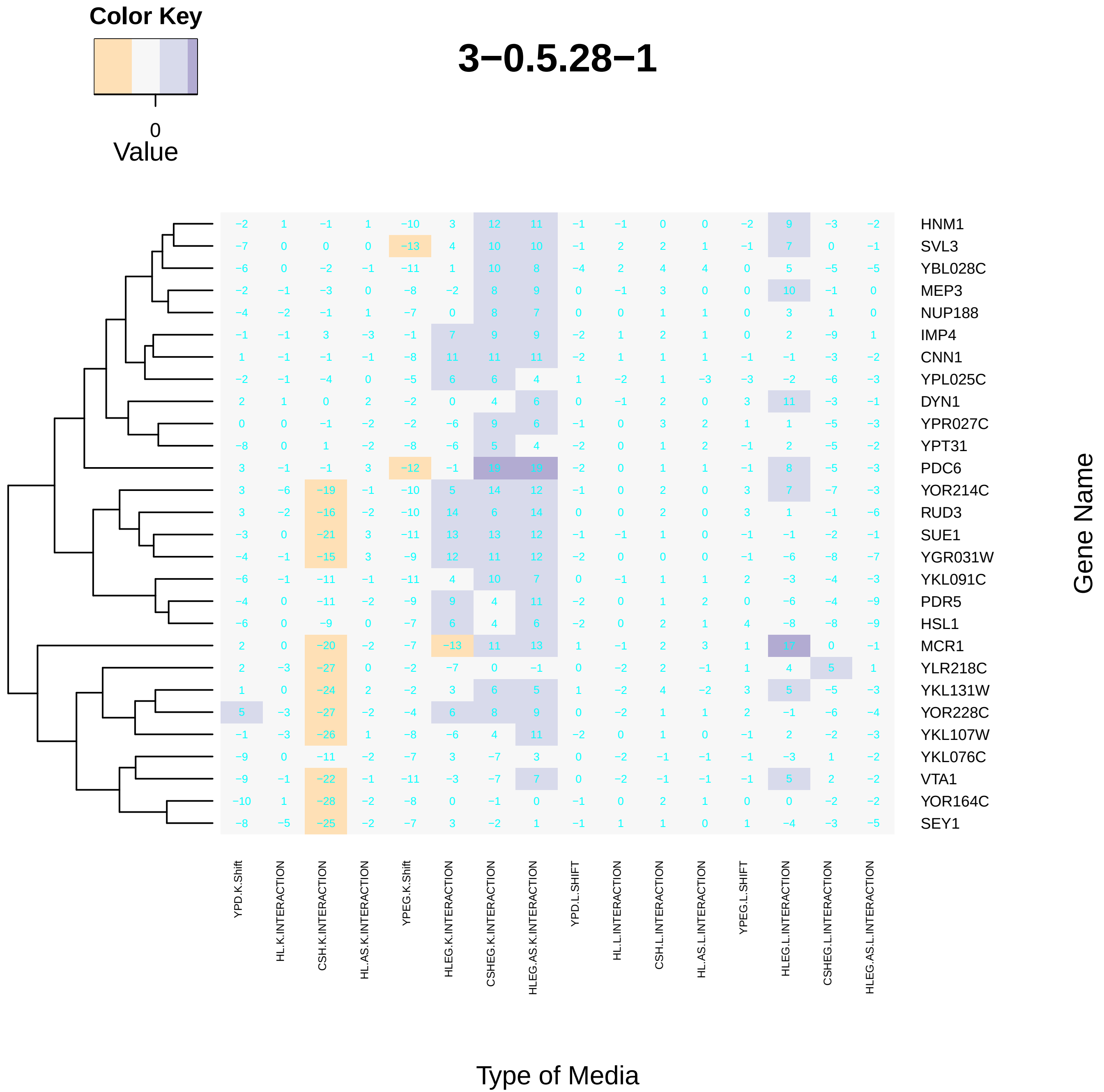

3−0.5.28−1
Color Key
0
Value
YPD.K.Shift HL.K.INTERACTION CSH.K.INTERACTION HL.AS.K.INTERACTION YPEG.K.Shift HLEG.K.INTERACTION CSHEG.K.INTERACTION HLEG.AS.K.INTERACTION YPD.L.SHIFT HL.L.INTERACTION CSH.L.INTERACTION HL.AS.L.INTERACTION YPEG.L.SHIFT HLEG.L.INTERACTION CSHEG.L.INTERACTION HLEG.AS.L.INTERACTION
HNM1 −2 1 −1 1 −10 3 12 11 −1 −1 0 0 −2 9 −3 −2
SVL3 −7 0 0 0 −13 4 10 10 −1 2 2 1 −1 7 0 −1
YBL028C −6 0 −2 −1 −11 1 10 8 −4 2 4 4 0 5 −5 −5
MEP3 −2 −1 −3 0 −8 −2 8 9 0 −1 3 0 0 10 −1 0
NUP188 −4 −2 −1 1 −7 0 8 7 0 0 1 1 0 3 1 0
IMP4 −1 −1 3 −3 −1 7 9 9 −2 1 2 1 0 2 −9 1
CNN1 1 −1 −1 −1 −8 11 11 11 −2 1 1 1 −1 −1 −3 −2
YPL025C −2 −1 −4 0 −5 6 6 4 1 −2 1 −3 −3 −2 −6 −3
DYN1 2 1 0 2 −2 0 4 6 0 −1 2 0 3 11 −3 −1
YPR027C 0 0 −1 −2 −2 −6 9 6 −1 0 3 2 1 1 −5 −3
YPT31 −8 0 1 −2 −8 −6 5 4 −2 0 1 2 −1 2 −5 −2
PDC6 3 −1 −1 3 −12 −1 19 19 −2 0 1 1 −1 8 −5 −3
YOR214C 3 −6 −19 −1 −10 5 14 12 −1 0 2 0 3 7 −7 −3
RUD3 3 −2 −16 −2 −10 14 6 14 0 0 2 0 3 1 −1 −6
SUE1 −3 0 −21 3 −11 13 13 12 −1 −1 1 0 −1 −1 −2 −1
YGR031W −4 −1 −15 3 −9 12 11 12 −2 0 0 0 −1 −6 −8 −7
YKL091C −6 −1 −11 −1 −11 4 10 7 0 −1 1 1 2 −3 −4 −3
PDR5 −4 0 −11 −2 −9 9 4 11 −2 0 1 2 0 −6 −4 −9
HSL1 −6 0 −9 0 −7 6 4 6 −2 0 2 1 4 −8 −8 −9
MCR1 2 0 −20 −2 −7 −13 11 13 1 −1 2 3 1 17 0 −1
YLR218C 2 −3 −27 0 −2 −7 0 −1 0 −2 2 −1 1 4 5 1
YKL131W 1 0 −24 2 −2 3 6 5 1 −2 4 −2 3 5 −5 −3
YOR228C 5 −3 −27 −2 −4 6 8 9 0 −2 1 1 2 −1 −6 −4
YKL107W −1 −3 −26 1 −8 −6 4 11 −2 0 1 0 −1 2 −2 −3
YKL076C −9 0 −11 −2 −7 3 −7 3 0 −2 −1 −1 −1 −3 1 −2
VTA1 −9 −1 −22 −1 −11 −3 −7 7 0 −2 −1 −1 −1 5 2 −2
YOR164C −10 1 −28 −2 −8 0 −1 0 −1 0 2 1 0 0 −2 −2
SEY1 −8 −5 −25 −2 −7 3 −2 1 −1 1 1 0 1 −4 −3 −5
Gene Name
Type of Media

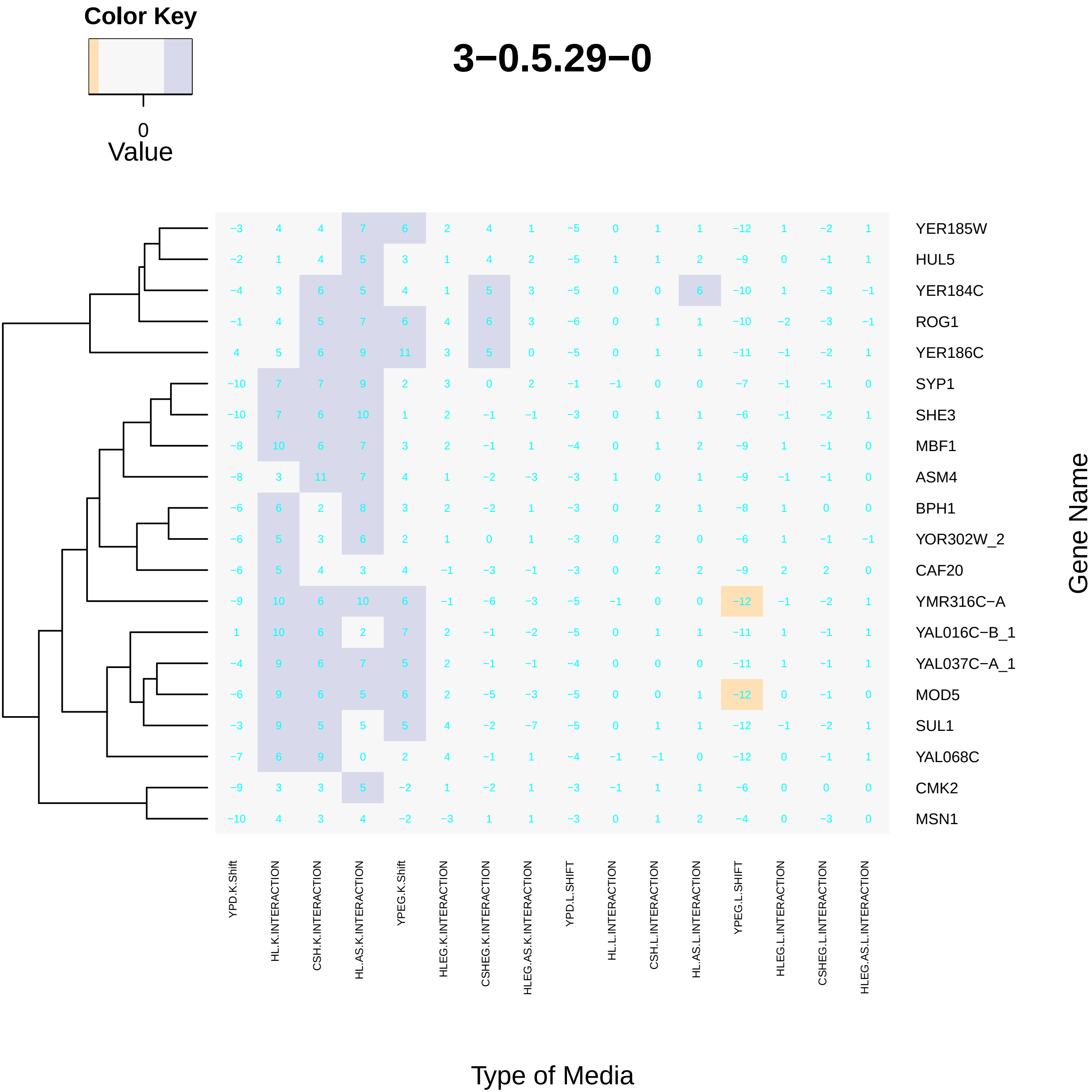

Color Key
0
Value
3−0.5.29−0
YER185W −3 4 4 7 6 2 4 1 −5 0 1 1 −12 1 −2 1
HUL5 −2 1 4 5 3 1 4 2 −5 1 1 2 −9 0 −1 1
YER184C −4 3 6 5 4 1 5 3 −5 0 0 6 −10 1 −3 −1
ROG1 −1 4 5 7 6 4 6 3 −6 0 1 1 −10 −2 −3 −1
YER186C 4 5 6 9 11 3 5 0 −5 0 1 1 −11 −1 −2 1
SYP1 −10 7 7 9 2 3 0 2 −1 −1 0 0 −7 −1 −1 0
SHE3 −10 7 6 10 1 2 −1 −1 −3 0 1 1 −6 −1 −2 1
MBF1 −8 10 6 7 3 2 −1 1 −4 0 1 2 −9 1 −1 0
ASM4 −8 3 11 7 4 1 −2 −3 −3 1 0 1 −9 −1 −1 0
BPH1 −6 6 2 8 3 2 −2 1 −3 0 2 1 −8 1 0 0
YOR302W_2 −6 5 3 6 2 1 0 1 −3 0 2 0 −6 1 −1 −1
CAF20 −6 5 4 3 4 −1 −3 −1 −3 0 2 2 −9 2 2 0
YMR316C−A −9 10 6 10 6 −1 −6 −3 −5 −1 0 0 −12 −1 −2 1
YAL016C−B_1 1 10 6 2 7 2 −1 −2 −5 0 1 1 −11 1 −1 1
YAL037C−A_1 −4 9 6 7 5 2 −1 −1 −4 0 0 0 −11 1 −1 1
MOD5 −6 9 6 5 6 2 −5 −3 −5 0 0 1 −12 0 −1 0
SUL1 −3 9 5 5 5 4 −2 −7 −5 0 1 1 −12 −1 −2 1
YAL068C −7 6 9 0 2 4 −1 1 −4 −1 −1 0 −12 0 −1 1
CMK2 −9 3 3 5 −2 1 −2 1 −3 −1 1 1 −6 0 0 0
MSN1 −10 4 3 4 −2 −3 1 1 −3 0 1 2 −4 0 −3 0
Gene Name
YPD.K.Shift
HL.K.INTERACTION
CSH.K.INTERACTION
HL.AS.K.INTERACTION
YPEG.K.Shift
HLEG.K.INTERACTION
CSHEG.K.INTERACTION
HLEG.AS.K.INTERACTION
YPD.L.SHIFT
HL.L.INTERACTION
CSH.L.INTERACTION
HL.AS.L.INTERACTION
YPEG.L.SHIFT
HLEG.L.INTERACTION
CSHEG.L.INTERACTION
HLEG.AS.L.INTERACTION
Type of Media

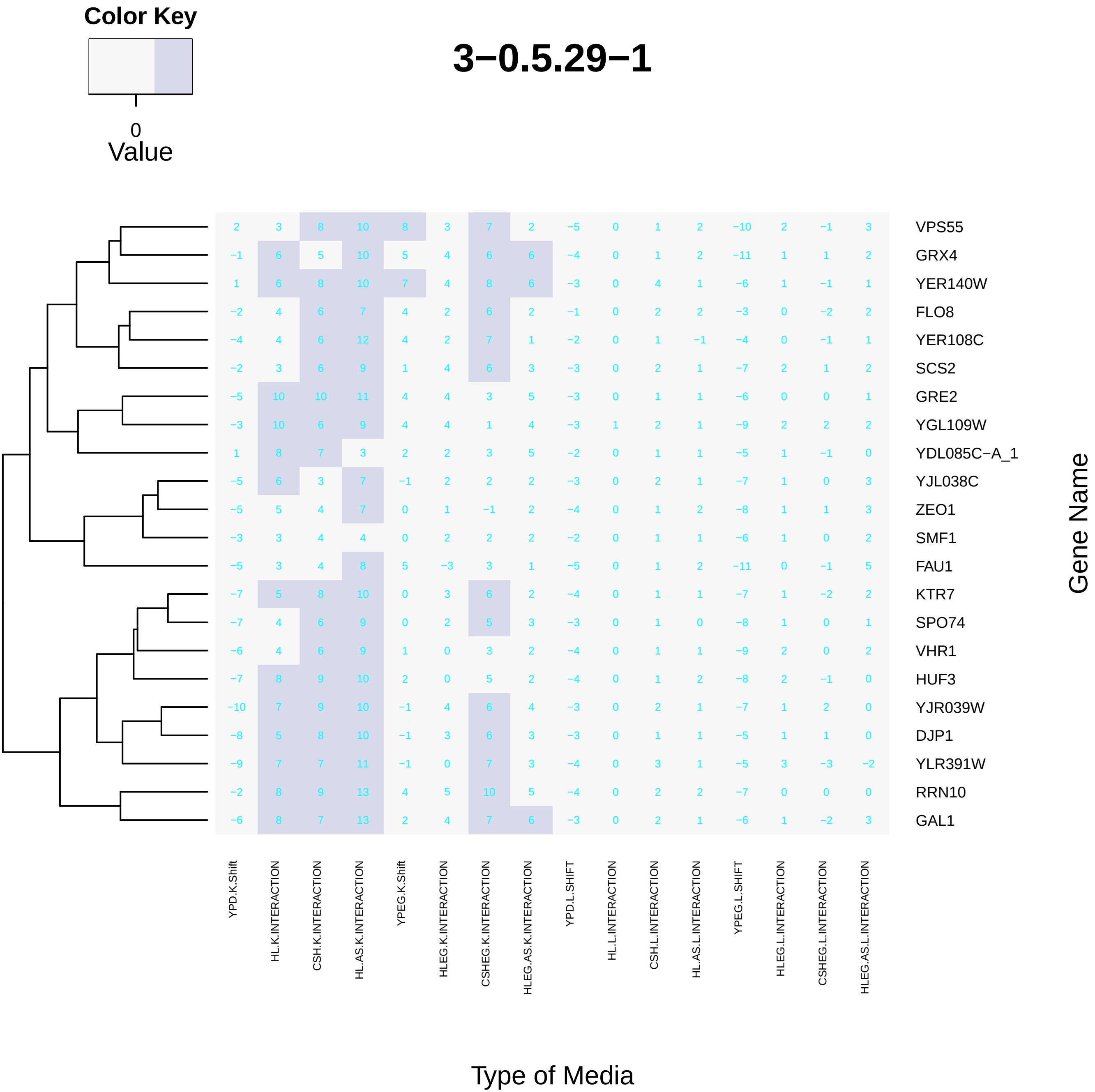
Color Key
0
Value
3−0.5.29−1
YPD.K.Shift
HL.K.INTERACTION
CSH.K.INTERACTION
HL.AS.K.INTERACTION
YPEG.K.Shift
HLEG.K.INTERACTION
CSHEG.K.INTERACTION
HLEG.AS.K.INTERACTION
YPD.L.SHIFT
HL.L.INTERACTION
CSH.L.INTERACTION
HL.AS.L.INTERACTION
YPEG.L.SHIFT
HLEG.L.INTERACTION
CSHEG.L.INTERACTION
HLEG.AS.L.INTERACTION
VPS55 2 3 8 10 8 3 7 2 −5 0 1 2 −10 2 −1 3
GRX4 −1 6 5 10 5 4 6 6 −4 0 1 2 −11 1 1 2
YER140W 1 6 8 10 7 4 8 6 −3 0 4 1 −6 1 −1 1
FLO8 −2 4 6 7 4 2 6 2 −1 0 2 2 −3 0 −2 2
YER108C −4 4 6 12 4 2 7 1 −2 0 1 −1 −4 0 −1 1
SCS2 −2 3 6 9 1 4 6 3 −3 0 2 1 −7 2 1 2
GRE2 −5 10 10 11 4 4 3 5 −3 0 1 1 −6 0 0 1
YGL109W −3 10 6 9 4 4 1 4 −3 1 2 1 −9 2 2 2
YDL085C−A_1 1 8 7 3 2 2 3 5 −2 0 1 1 −5 1 −1 0
YJL038C −5 6 3 7 −1 2 2 2 −3 0 2 1 −7 1 0 3
ZEO1 −5 5 4 7 0 1 −1 2 −4 0 1 2 −8 1 1 3
SMF1 −3 3 4 4 0 2 2 2 −2 0 1 1 −6 1 0 2
FAU1 −5 3 4 8 5 −3 3 1 −5 0 1 2 −11 0 −1 5
KTR7 −7 5 8 10 0 3 6 2 −4 0 1 1 −7 1 −2 2
SPO74 −7 4 6 9 0 2 5 3 −3 0 1 0 −8 1 0 1
VHR1 −6 4 6 9 1 0 3 2 −4 0 1 1 −9 2 0 2
HUF3 −7 8 9 10 2 0 5 2 −4 0 1 2 −8 2 −1 0
YJR039W −10 7 9 10 −1 4 6 4 −3 0 2 1 −7 1 2 0
DJP1 −8 5 8 10 −1 3 6 3 −3 0 1 1 −5 1 1 0
YLR391W −9 7 7 11 −1 0 7 3 −4 0 3 1 −5 3 −3 −2
RRN10 −2 8 9 13 4 5 10 5 −4 0 2 2 −7 0 0 0
GAL1 −6 8 7 13 2 4 7 6 −3 0 2 1 −6 1 −2 3
Gene Name
Type of Media

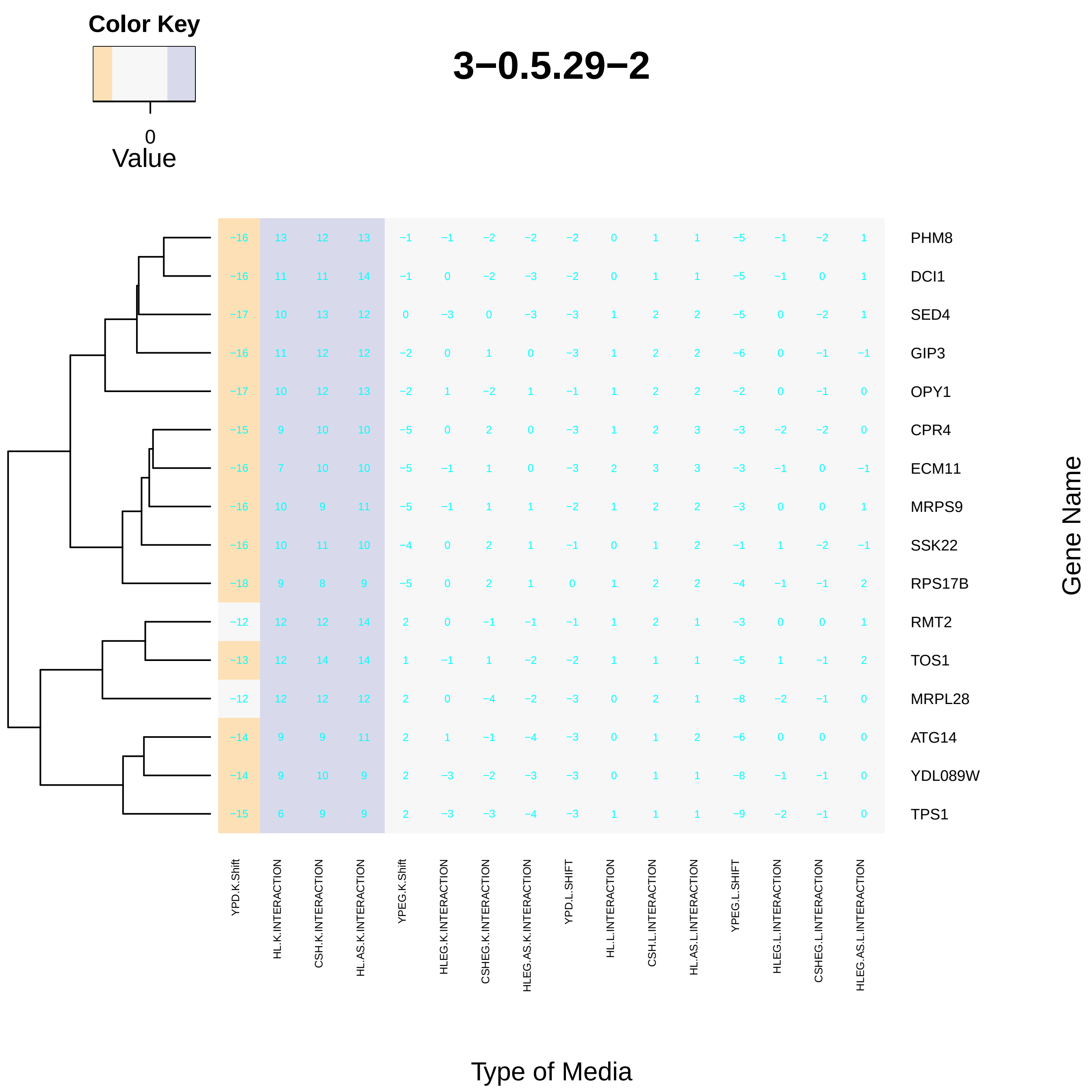
Color Key
0
Value
3−0.5.29−2
YPD.K.Shift HL.K.INTERACTION CSH.K.INTERACTION HL.AS.K.INTERACTION YPEG.K.Shift HLEG.K.INTERACTION CSHEG.K.INTERACTION HLEG.AS.K.INTERACTION YPD.L.SHIFT HL.L.INTERACTION CSH.L.INTERACTION HL.AS.L.INTERACTION YPEG.L.SHIFT HLEG.L.INTERACTION CSHEG.L.INTERACTION HLEG.AS.L.INTERACTION
PHM8 −16 13 12 13 −1 −1 −2 −2 −2 0 1 1 −5 −1 −2 1
DCI1 −16 11 11 14 −1 0 −2 −3 −2 0 1 1 −5 −1 0 1
SED4 −17 10 13 12 0 −3 0 −3 −3 1 2 2 −5 0 −2 1
GIP3 −16 11 12 12 −2 0 1 0 −3 1 2 2 −6 0 −1 −1
OPY1 −17 10 12 13 −2 1 −2 1 −1 1 2 2 −2 0 −1 0
CPR4 −15 9 10 10 −5 0 2 0 −3 1 2 3 −3 −2 −2 0
ECM11 −16 7 10 10 −5 −1 1 0 −3 2 3 3 −3 −1 0 −1
MRPS9 −16 10 9 11 −5 −1 1 1 −2 1 2 2 −3 0 0 1
SSK22 −16 10 11 10 −4 0 2 1 −1 0 1 2 −1 1 −2 −1
RPS17B −18 9 8 9 −5 0 2 1 0 1 2 2 −4 −1 −1 2
RMT2 −12 12 12 14 2 0 −1 −1 −1 1 2 1 −3 0 0 1
TOS1 −13 12 14 14 1 −1 1 −2 −2 1 1 1 −5 1 −1 2
MRPL28 −12 12 12 12 2 0 −4 −2 −3 0 2 1 −8 −2 −1 0
ATG14 −14 9 9 11 2 1 −1 −4 −3 0 1 2 −6 0 0 0
YDL089W −14 9 10 9 2 −3 −2 −3 −3 0 1 1 −8 −1 −1 0
TPS1 −15 6 9 9 2 −3 −3 −4 −3 1 1 1 −9 −2 −1 0
Gene Name
Type of Media

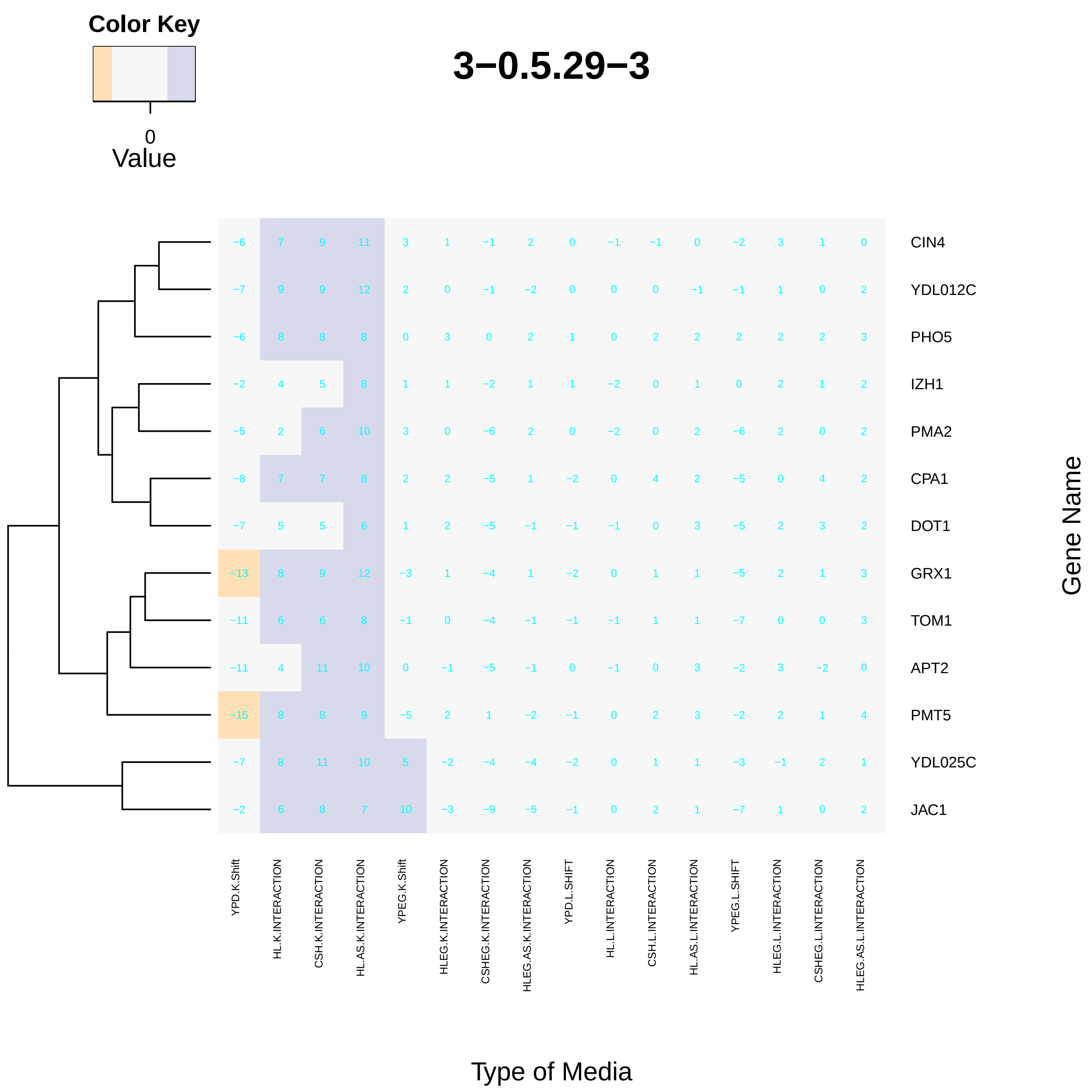
Color Key
0
Value
3−0.5.29−3
−6 7 9 11 3 1 −1 2 0 −1 −1 0 −2 3 1 0 CIN4
−7 9 9 12 2 0 −1 −2 0 0 0 −1 −1 1 0 2 YDL012C
−6 8 8 8 0 3 0 2 1 0 2 2 2 2 2 3 PHO5
−2 4 5 8 1 1 −2 1 1 −2 0 1 0 2 1 2 IZH1
−5 2 6 10 3 0 −6 2 0 −2 0 2 −6 2 0 2 PMA2
−8 7 7 8 2 2 −5 1 −2 0 4 2 −5 0 4 2 CPA1
−7 5 5 6 1 2 −5 −1 −1 −1 0 3 −5 2 3 2 DOT1
−13 8 9 12 −3 1 −4 1 −2 0 1 1 −5 2 1 3 GRX1
−11 6 6 8 −1 0 −4 −1 −1 −1 1 1 −7 0 0 3 TOM1
−11 4 11 10 0 −1 −5 −1 0 −1 0 3 −2 3 −2 0 APT2
−15 8 8 9 −5 2 1 −2 −1 0 2 3 −2 2 1 4 PMT5
−7 8 11 10 5 −2 −4 −4 −2 0 1 1 −3 −1 2 1 YDL025C
−2 6 8 7 10 −3 −9 −5 −1 0 2 1 −7 1 0 2 JAC1
Gene Name
YPD.K.Shift
HL.K.INTERACTION
CSH.K.INTERACTION
HL.AS.K.INTERACTION
YPEG.K.Shift
HLEG.K.INTERACTION
CSHEG.K.INTERACTION
HLEG.AS.K.INTERACTION
YPD.L.SHIFT
HL.L.INTERACTION
CSH.L.INTERACTION
HL.AS.L.INTERACTION
YPEG.L.SHIFT
HLEG.L.INTERACTION
CSHEG.L.INTERACTION
HLEG.AS.L.INTERACTION
Type of Media

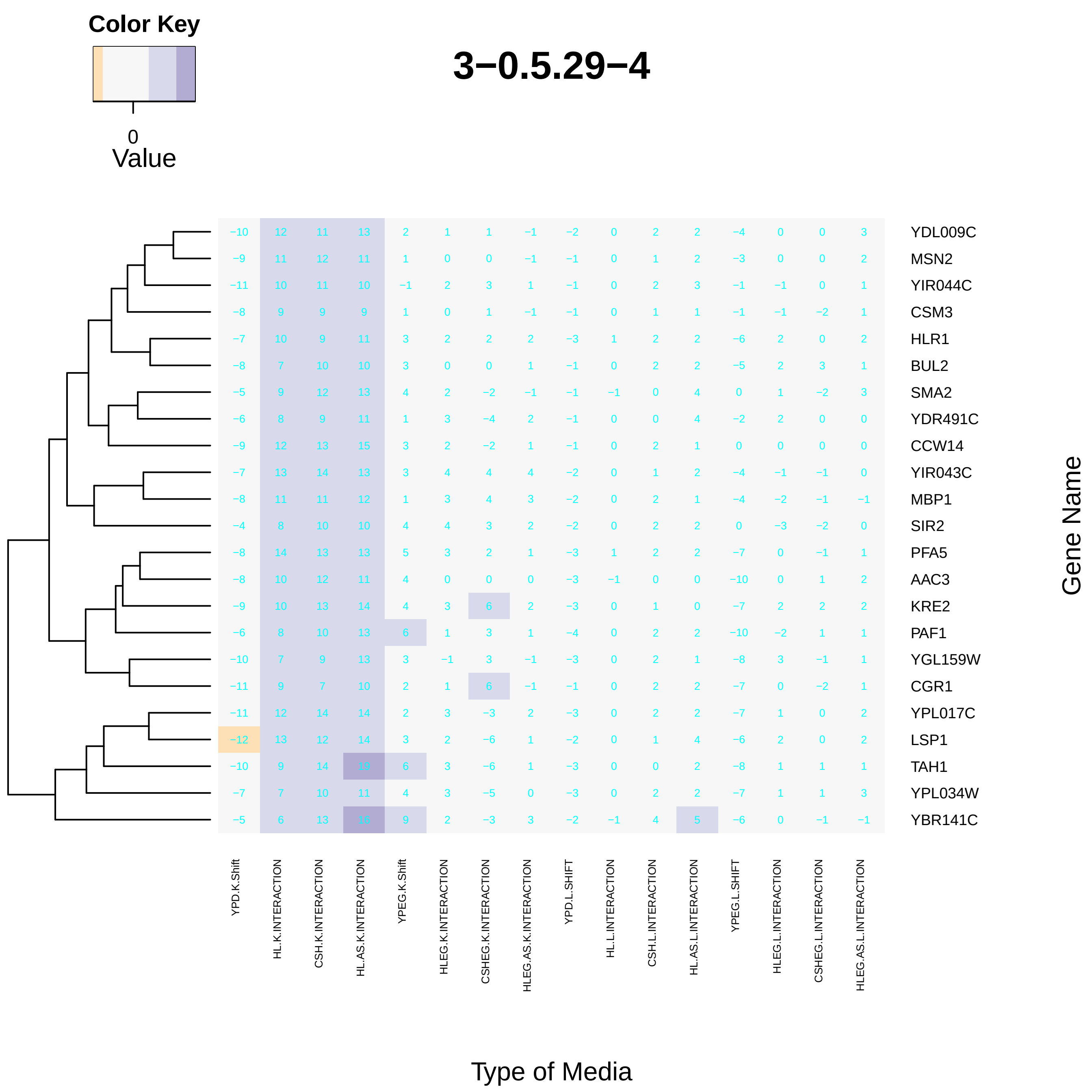
Color Key
0
Value
3−0.5.29−4
YPD.K.Shift
HL.K.INTERACTION
CSH.K.INTERACTION
HL.AS.K.INTERACTION
YPEG.K.Shift
HLEG.K.INTERACTION
CSHEG.K.INTERACTION
HLEG.AS.K.INTERACTION
YPD.L.SHIFT
HL.L.INTERACTION
CSH.L.INTERACTION
HL.AS.L.INTERACTION
YPEG.L.SHIFT
HLEG.L.INTERACTION
CSHEG.L.INTERACTION
HLEG.AS.L.INTERACTION
YDL009C −10 12 11 13 2 1 1 −1 −2 0 2 2 −4 0 0 3
MSN2 −9 11 12 11 1 0 0 −1 −1 0 1 2 −3 0 0 2
YIR044C −11 10 11 10 −1 2 3 1 −1 0 2 3 −1 −1 0 1
CSM3 −8 9 9 9 1 0 1 −1 −1 0 1 1 −1 −1 −2 1
HLR1 −7 10 9 11 3 2 2 2 −3 1 2 2 −6 2 0 2
BUL2 −8 7 10 10 3 0 0 1 −1 0 2 2 −5 2 3 1
SMA2 −5 9 12 13 4 2 −2 −1 −1 −1 0 4 0 1 −2 3
YDR491C −6 8 9 11 1 3 −4 2 −1 0 0 4 −2 2 0 0
CCW14 −9 12 13 15 3 2 −2 1 −1 0 2 1 0 0 0 0
YIR043C −7 13 14 13 3 4 4 4 −2 0 1 2 −4 −1 −1 0
MBP1 −8 11 11 12 1 3 4 3 −2 0 2 1 −4 −2 −1 −1
SIR2 −4 8 10 10 4 4 3 2 −2 0 2 2 0 −3 −2 0
PFA5 −8 14 13 13 5 3 2 1 −3 1 2 2 −7 0 −1 1
AAC3 −8 10 12 11 4 0 0 0 −3 −1 0 0 −10 0 1 2
KRE2 −9 10 13 14 4 3 6 2 −3 0 1 0 −7 2 2 2
PAF1 −6 8 10 13 6 1 3 1 −4 0 2 2 −10 −2 1 1
YGL159W −10 7 9 13 3 −1 3 −1 −3 0 2 1 −8 3 −1 1
CGR1 −11 9 7 10 2 1 6 −1 −1 0 2 2 −7 0 −2 1
YPL017C −11 12 14 14 2 3 −3 2 −3 0 2 2 −7 1 0 2
LSP1 −12 13 12 14 3 2 −6 1 −2 0 1 4 −6 2 0 2
TAH1 −10 9 14 19 6 3 −6 1 −3 0 0 2 −8 1 1 1
YPL034W −7 7 10 11 4 3 −5 0 −3 0 2 2 −7 1 1 3
YBR141C −5 6 13 16 9 2 −3 3 −2 −1 4 5 −6 0 −1 −1
Gene Name
Type of Media

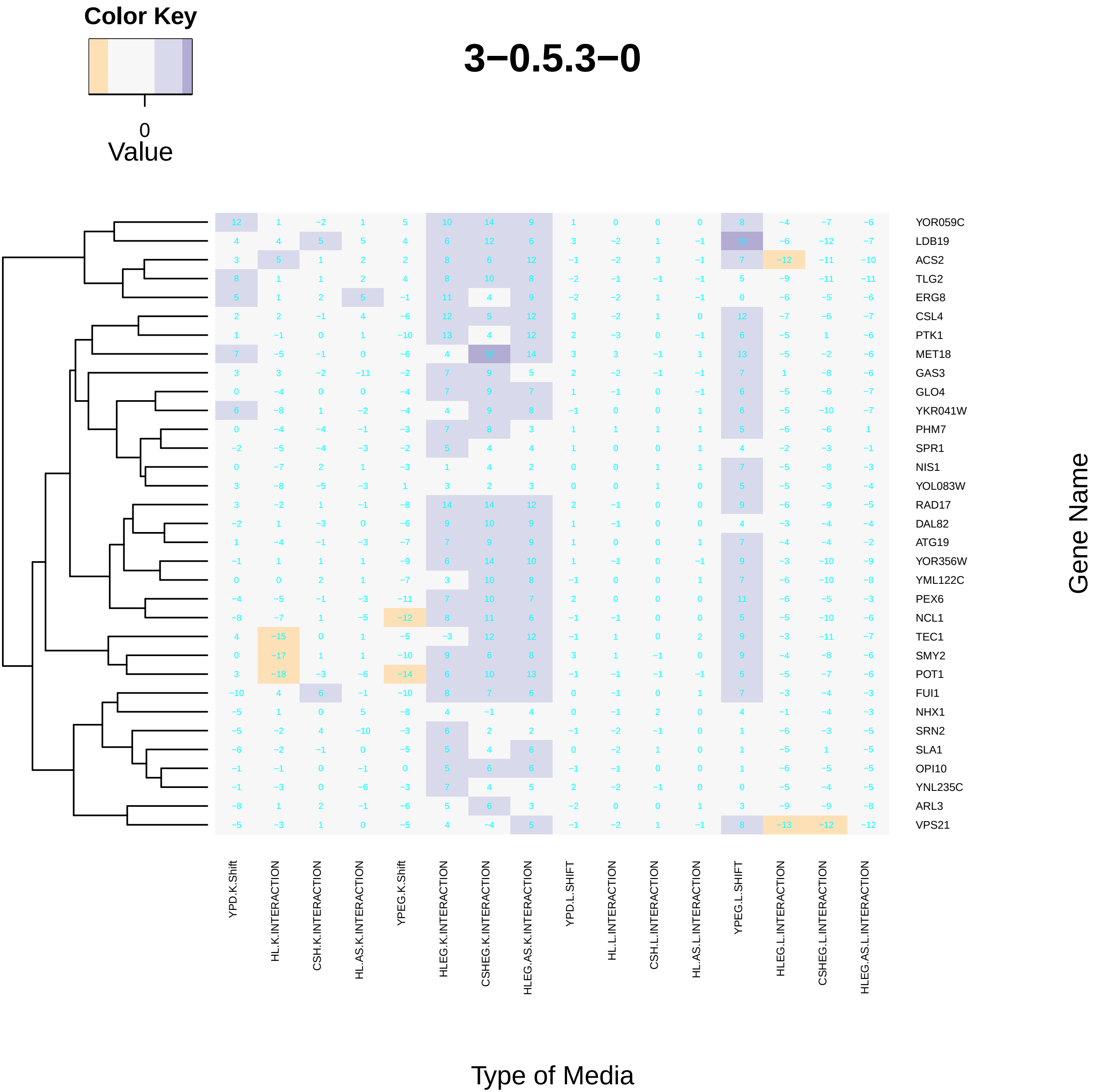

Color Key
0
Value
3−0.5.3−0
YPD.K.Shift HL.K.INTERACTION CSH.K.INTERACTION HL.AS.K.INTERACTION YPEG.K.Shift HLEG.K.INTERACTION CSHEG.K.INTERACTION HLEG.AS.K.INTERACTION YPD.L.SHIFT HL.L.INTERACTION CSH.L.INTERACTION HL.AS.L.INTERACTION YPEG.L.SHIFT HLEG.L.INTERACTION CSHEG.L.INTERACTION HLEG.AS.L.INTERACTION
YOR059C 12 1 −2 1 5 10 14 9 1 0 0 0 8 −4 −7 −6
LDB19 4 4 5 5 4 6 12 6 3 −2 1 −1 15 −6 −12 −7
ACS2 3 5 1 2 2 8 6 12 −1 −2 3 −1 7 −12 −11 −10
TLG2 8 1 1 2 4 8 10 8 −2 −1 −1 −1 5 −9 −11 −11
ERG8 5 1 2 5 −1 11 4 9 −2 −2 1 −1 0 −6 −5 −6
CSL4 2 2 −1 4 −6 12 5 12 3 −2 1 0 12 −7 −6 −7
PTK1 1 −1 0 1 −10 13 4 12 2 −3 0 −1 6 −5 1 −6
MET18 7 −5 −1 0 −6 4 16 14 3 3 −1 1 13 −5 −2 −6
GAS3 3 3 −2 −11 −2 7 9 5 2 −2 −1 −1 7 1 −8 −6
GLO4 0 −4 0 0 −4 7 9 7 1 −1 0 −1 6 −5 −6 −7
YKR041W 6 −8 1 −2 −4 4 9 8 −1 0 0 1 6 −5 −10 −7
PHM7 0 −4 −4 −1 −3 7 8 3 1 1 1 1 5 −6 −6 1
SPR1 −2 −5 −4 −3 −2 5 4 4 1 0 0 1 4 −2 −3 −1
NIS1 0 −7 2 1 −3 1 4 2 0 0 1 1 7 −5 −8 −3
YOL083W 3 −8 −5 −3 1 3 2 3 0 0 1 0 5 −5 −3 −4
RAD17 3 −2 1 −1 −8 14 14 12 2 −1 0 0 9 −6 −9 −5
DAL82 −2 1 −3 0 −6 9 10 9 1 −1 0 0 4 −3 −4 −4
ATG19 1 −4 −1 −3 −7 7 9 9 1 0 0 1 7 −4 −4 −2
YOR356W −1 1 1 1 −9 6 14 10 1 −1 0 −1 9 −3 −10 −9
YML122C 0 0 2 1 −7 3 10 8 −1 0 0 1 7 −6 −10 −8
PEX6 −4 −5 −1 −3 −11 7 10 7 2 0 0 0 11 −6 −5 −3
NCL1 −8 −7 1 −5 −12 8 11 6 −1 −1 0 0 5 −5 −10 −6
TEC1 4 −15 0 1 −5 −3 12 12 −1 1 0 2 9 −3 −11 −7
SMY2 0 −17 1 1 −10 9 6 8 3 1 −1 0 9 −4 −8 −6
POT1 3 −18 −3 −6 −14 6 10 13 −1 −1 −1 −1 5 −5 −7 −6
FUI1 −10 4 6 −1 −10 8 7 6 0 −1 0 1 7 −3 −4 −3
NHX1 −5 1 0 5 −8 4 −1 4 0 −1 2 0 4 −1 −4 −3
SRN2 −5 −2 4 −10 −3 6 2 2 −1 −2 −1 0 1 −6 −3 −5
SLA1 −6 −2 −1 0 −5 5 4 6 0 −2 1 0 1 −5 1 −5
OPI10 −1 −1 0 −1 0 5 6 6 −1 −1 0 0 1 −6 −5 −5
YNL235C −1 −3 0 −6 −3 7 4 5 2 −2 −1 0 0 −5 −4 −5
ARL3 −8 1 2 −1 −6 5 6 3 −2 0 0 1 3 −9 −9 −8
VPS21 −5 −3 1 0 −5 4 −4 5 −1 −2 1 −1 8 −13 −12 −12
Gene Name
Type of Media

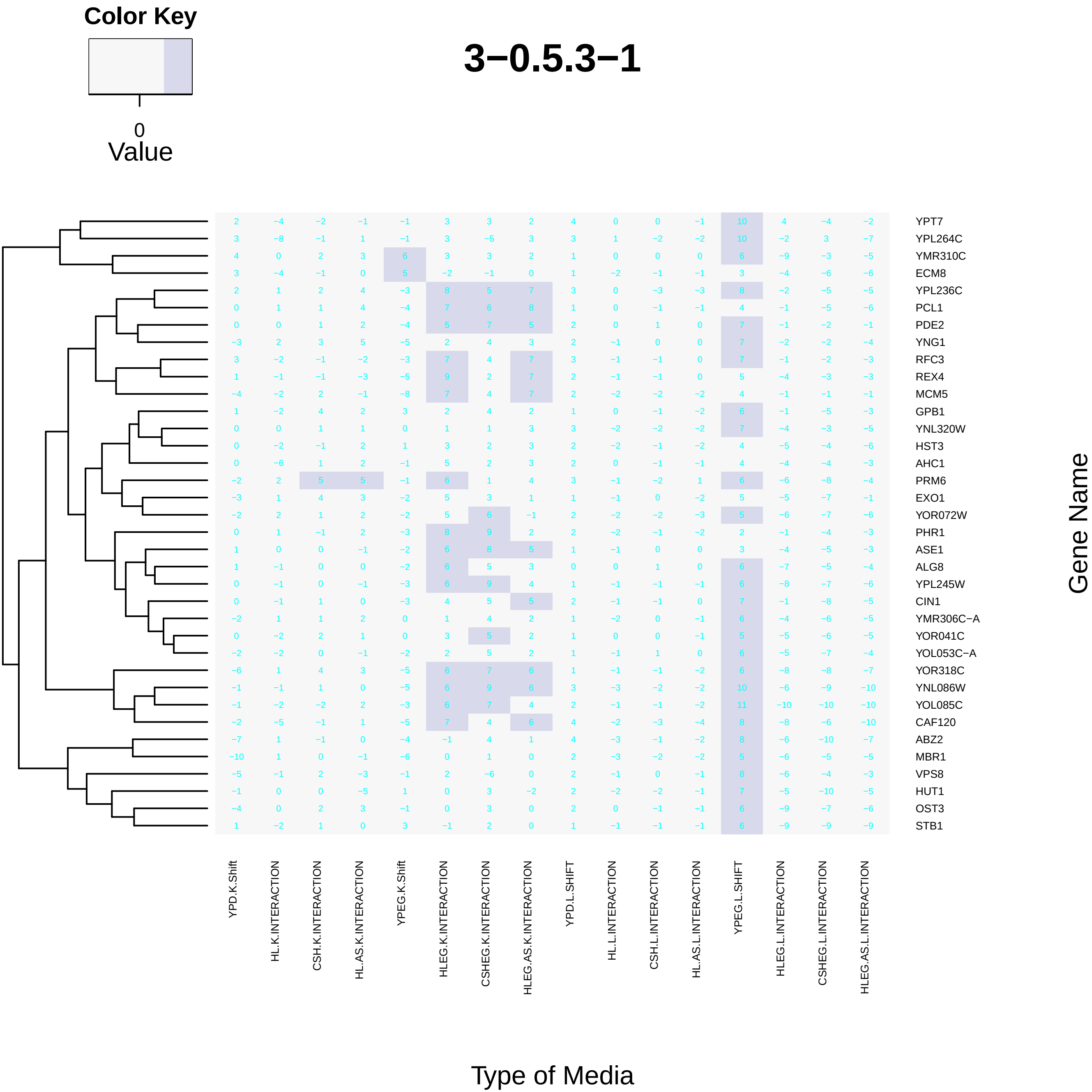
Color Key
0
Value
3−0.5.3−1
YPT7
YPL264C
YMR310C
ECM8
YPL236C
PCL1
PDE2
YNG1
RFC3
REX4
MCM5
GPB1
YNL320W
HST3
AHC1
PRM6
EXO1
YOR072W
PHR1
ASE1
ALG8
YPL245W
CIN1
YMR306C−A
YOR041C
YOL053C−A
YOR318C
YNL086W
YOL085C
CAF120
ABZ2
MBR1
VPS8
HUT1
OST3
STB1
Gene Name
YPD.K.Shift
HL.K.INTERACTION
CSH.K.INTERACTION
HL.AS.K.INTERACTION
YPEG.K.Shift
HLEG.K.INTERACTION
CSHEG.K.INTERACTION
HLEG.AS.K.INTERACTION
YPD.L.SHIFT
HL.L.INTERACTION
CSH.L.INTERACTION
HL.AS.L.INTERACTION
YPEG.L.SHIFT
HLEG.L.INTERACTION
CSHEG.L.INTERACTION
HLEG.AS.L.INTERACTION
Type of Media

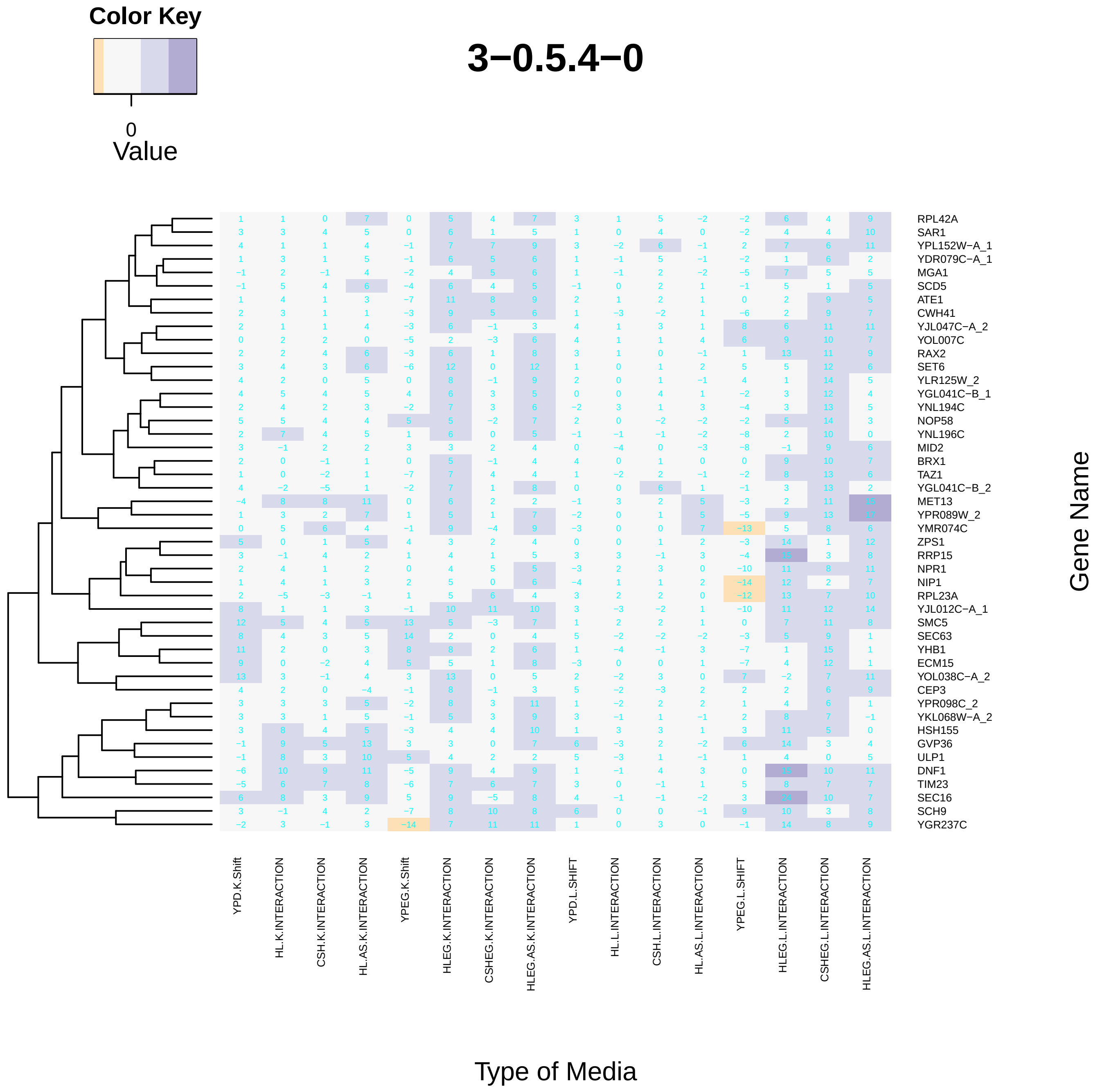

3−0.5.4−0
Color Key
0
Value
RPL42A
SAR1
YPL152W−A_1
YDR079C−A_1
MGA1
SCD5
ATE1
CWH41
YJL047C−A_2
YOL007C
RAX2
SET6
YLR125W_2
YGL041C−B_1
YNL194C
NOP58
YNL196C
MID2
BRX1
TAZ1
YGL041C−B_2
MET13
YPR089W_2
YMR074C
ZPS1
RRP15
NPR1
NIP1
RPL23A
YJL012C−A_1
SMC5
SEC63
YHB1
ECM15
YOL038C−A_2
CEP3
YPR098C_2
YKL068W−A_2
HSH155
GVP36
ULP1
DNF1
TIM23
SEC16
SCH9
YGR237C
Gene Name
YPD.K.Shift
HL.K.INTERACTION
CSH.K.INTERACTION
HL.AS.K.INTERACTION
YPEG.K.Shift
HLEG.K.INTERACTION
CSHEG.K.INTERACTION
HLEG.AS.K.INTERACTION
YPD.L.SHIFT
HL.L.INTERACTION
CSH.L.INTERACTION
HL.AS.L.INTERACTION
YPEG.L.SHIFT
HLEG.L.INTERACTION
CSHEG.L.INTERACTION
HLEG.AS.L.INTERACTION
Type of Media

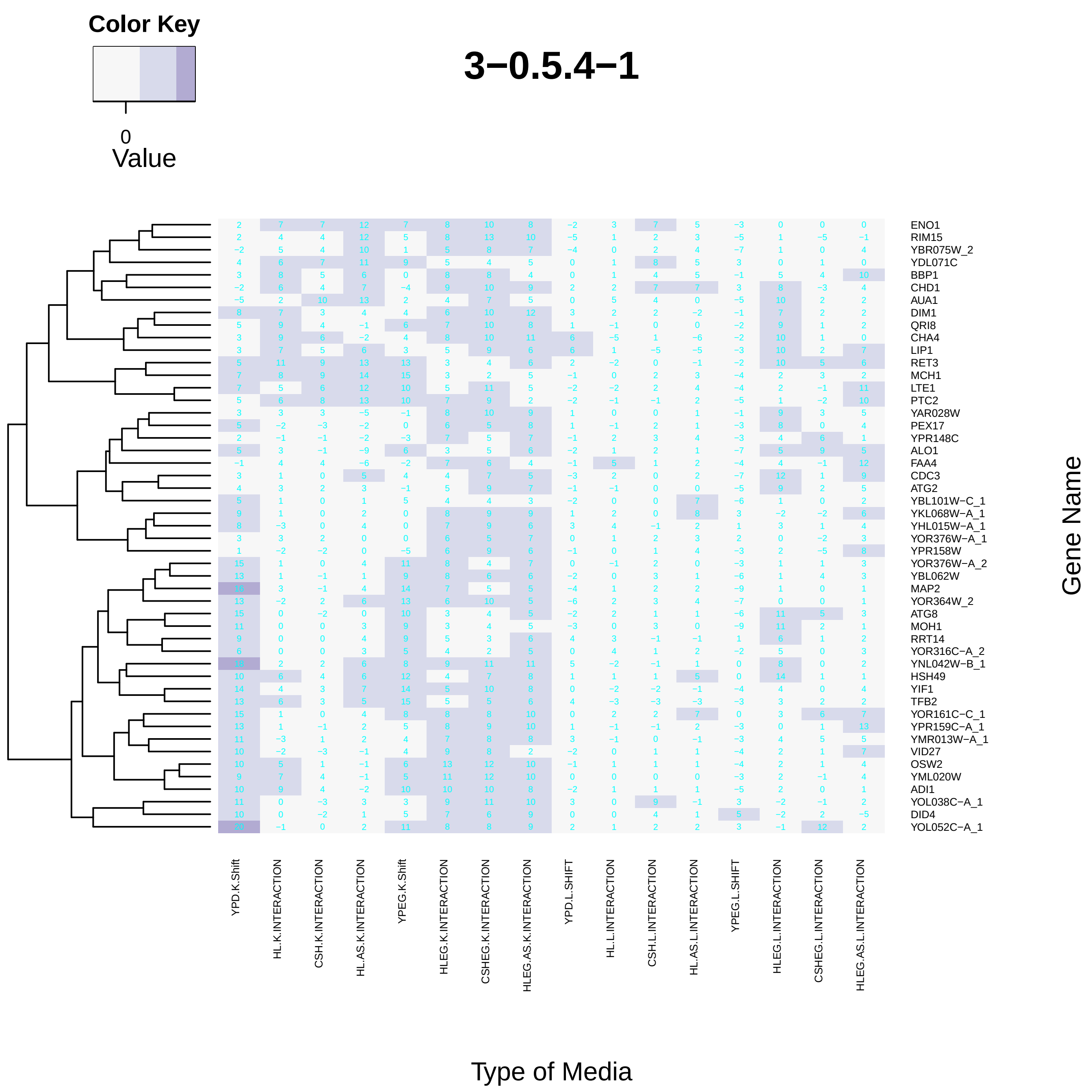
3−0.5.4−1
Color Key
0
Value
ENO1
RIM15
YBR075W_2
YDL071C
BBP1
CHD1
AUA1
DIM1
QRI8
CHA4
LIP1
RET3
MCH1
LTE1
PTC2
YAR028W
PEX17
YPR148C
ALO1
FAA4
CDC3
ATG2
YBL101W−C_1
YKL068W−A_1
YHL015W−A_1
YOR376W−A_1
YPR158W
YOR376W−A_2
YBL062W
MAP2
YOR364W_2
ATG8
MOH1
RRT14
YOR316C−A_2
YNL042W−B_1
HSH49
YIF1
TFB2
YOR161C−C_1
YPR159C−A_1
YMR013W−A_1
VID27
OSW2
YML020W
ADI1
YOL038C−A_1
DID4
YOL052C−A_1
Gene Name
YPD.K.Shift
HL.K.INTERACTION
CSH.K.INTERACTION
HL.AS.K.INTERACTION
YPEG.K.Shift
HLEG.K.INTERACTION
CSHEG.K.INTERACTION
HLEG.AS.K.INTERACTION
YPD.L.SHIFT
HL.L.INTERACTION
CSH.L.INTERACTION
HL.AS.L.INTERACTION
YPEG.L.SHIFT
HLEG.L.INTERACTION
CSHEG.L.INTERACTION
HLEG.AS.L.INTERACTION
Type of Media

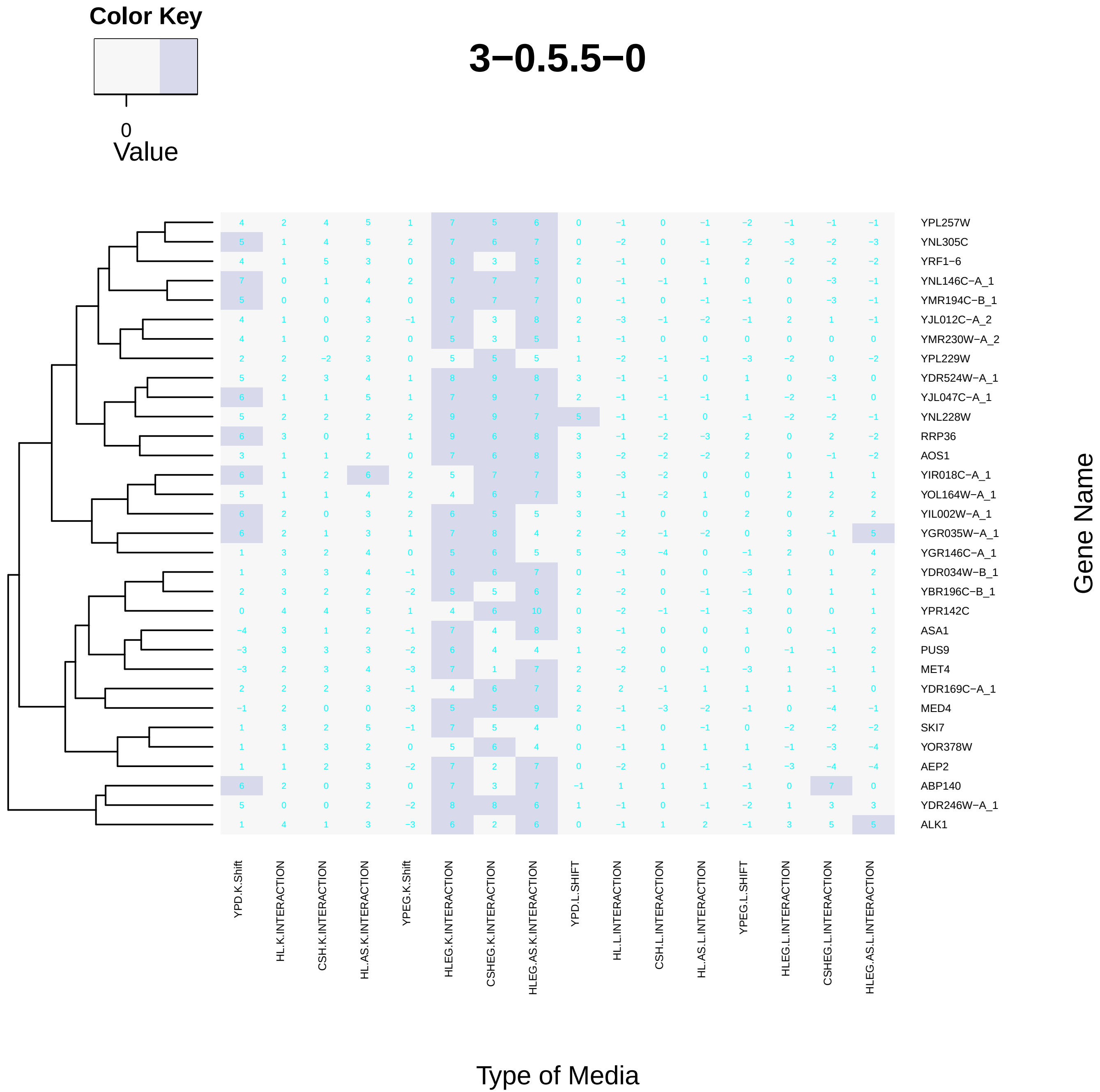

Color Key
0
Value
3−0.5.5−0
YPL257W
YNL305C
YRF1−6
YNL146C−A_1
YMR194C−B_1
YJL012C−A_2
YMR230W−A_2
YPL229W
YDR524W−A_1
YJL047C−A_1
YNL228W
RRP36
AOS1
YIR018C−A_1
YOL164W−A_1
YIL002W−A_1
YGR035W−A_1
YGR146C−A_1
YDR034W−B_1
YBR196C−B_1
YPR142C
ASA1
PUS9
MET4
YDR169C−A_1
MED4
SKI7
YOR378W
AEP2
ABP140
YDR246W−A_1
ALK1
Gene Name
YPD.K.Shift
HL.K.INTERACTION
CSH.K.INTERACTION
HL.AS.K.INTERACTION
YPEG.K.Shift
HLEG.K.INTERACTION
CSHEG.K.INTERACTION
HLEG.AS.K.INTERACTION
YPD.L.SHIFT
HL.L.INTERACTION
CSH.L.INTERACTION
HL.AS.L.INTERACTION
YPEG.L.SHIFT
HLEG.L.INTERACTION
CSHEG.L.INTERACTION
HLEG.AS.L.INTERACTION
Type of Media

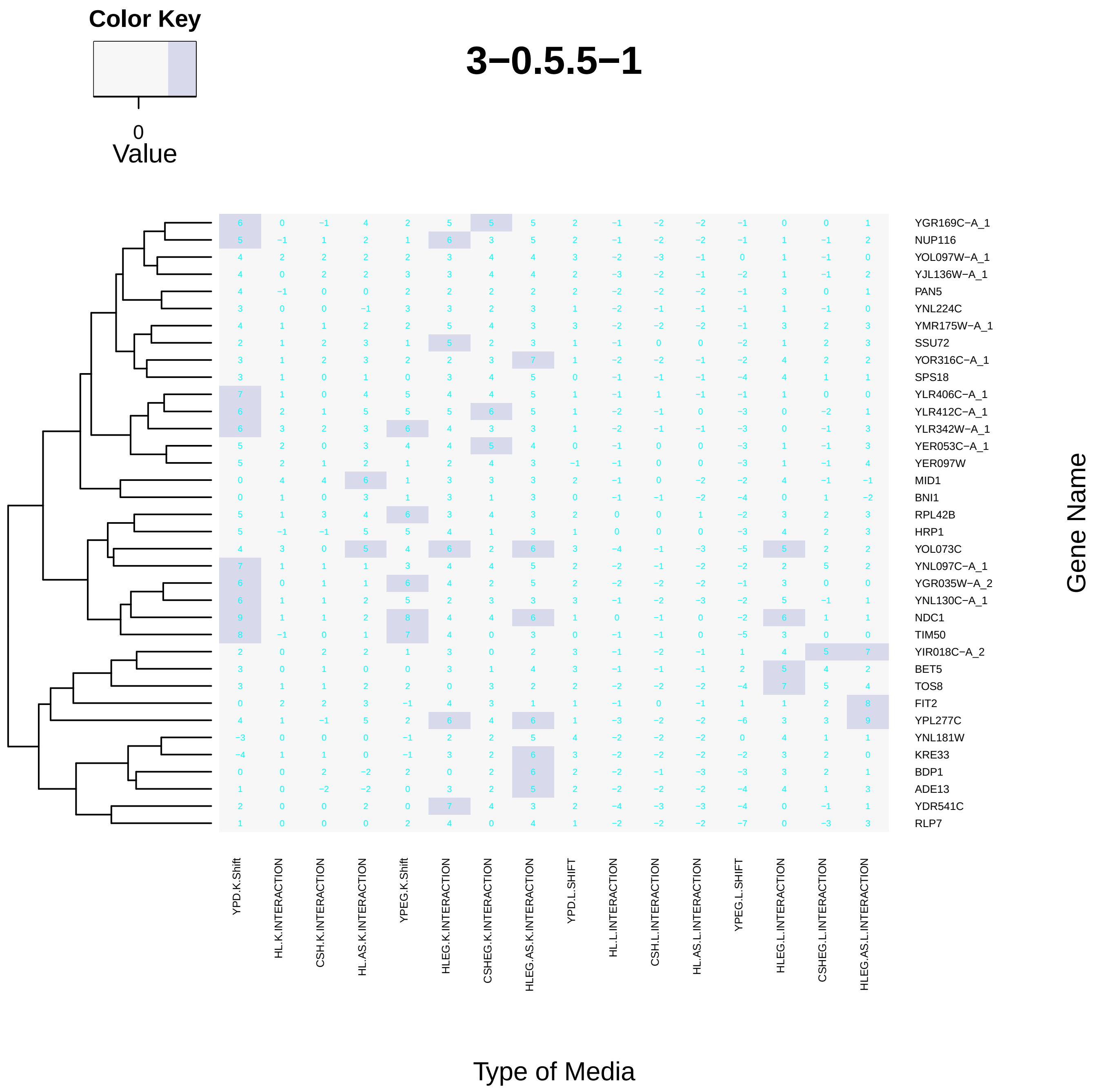

Color Key
0
Value
3−0.5.5−1
YGR169C−A_1
NUP116
YOL097W−A_1
YJL136W−A_1
PAN5
YNL224C
YMR175W−A_1
SSU72
YOR316C−A_1
SPS18
YLR406C−A_1
YLR412C−A_1
YLR342W−A_1
YER053C−A_1
YER097W
MID1
BNI1
RPL42B
HRP1
YOL073C
YNL097C−A_1
YGR035W−A_2
YNL130C−A_1
NDC1
TIM50
YIR018C−A_2
BET5
TOS8
FIT2
YPL277C
YNL181W
KRE33
BDP1
ADE13
YDR541C
RLP7
Gene Name
YPD.K.Shift
HL.K.INTERACTION
CSH.K.INTERACTION
HL.AS.K.INTERACTION
YPEG.K.Shift
HLEG.K.INTERACTION
CSHEG.K.INTERACTION
HLEG.AS.K.INTERACTION
YPD.L.SHIFT
HL.L.INTERACTION
CSH.L.INTERACTION
HL.AS.L.INTERACTION
YPEG.L.SHIFT
HLEG.L.INTERACTION
CSHEG.L.INTERACTION
HLEG.AS.L.INTERACTION
Type of Media

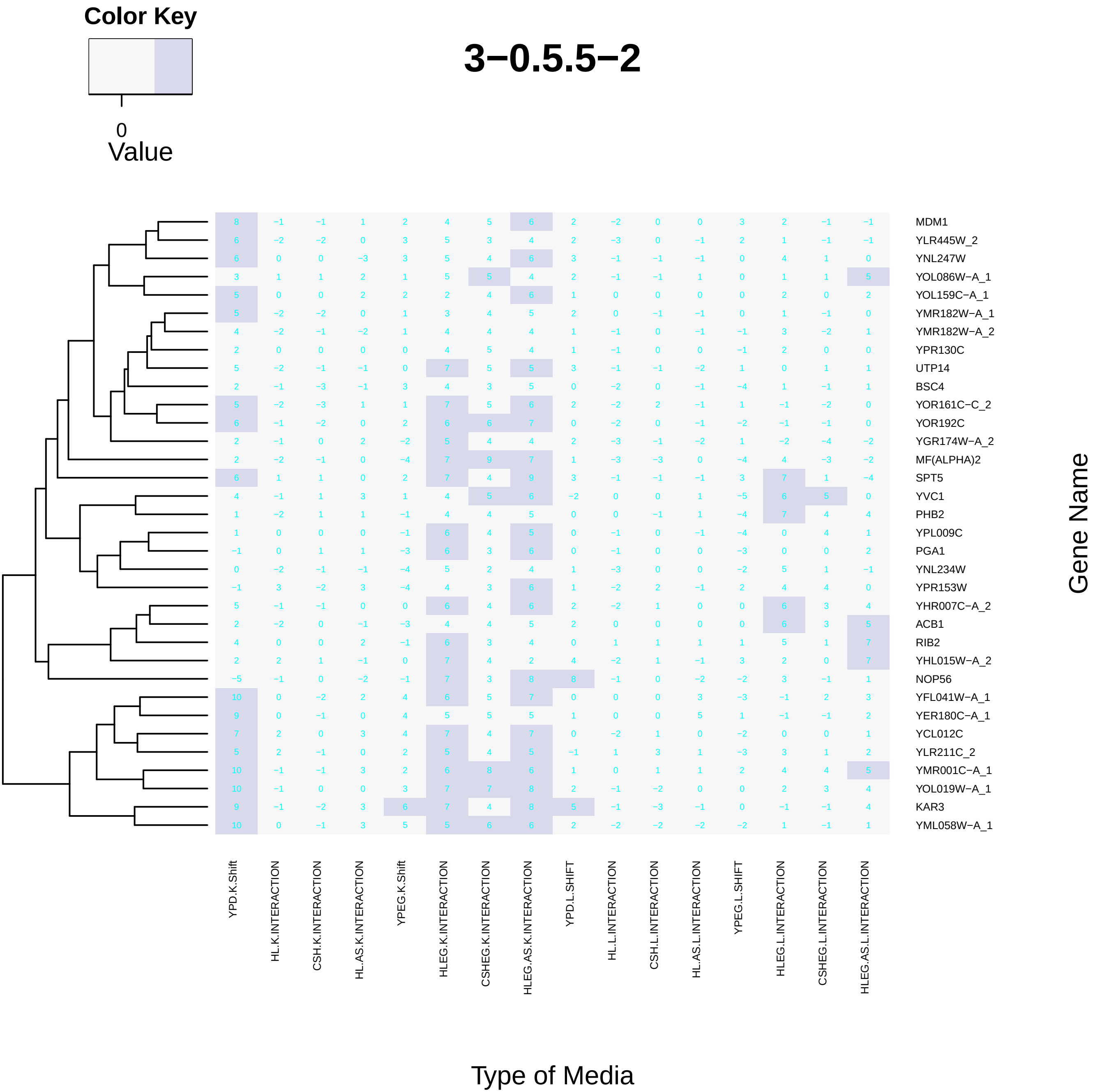
Color Key
0
Value
3−0.5.5−2
MDM1
YLR445W_2
YNL247W
YOL086W−A_1
YOL159C−A_1
YMR182W−A_1
YMR182W−A_2
YPR130C
UTP14
BSC4
YOR161C−C_2
YOR192C
YGR174W−A_2
MF(ALPHA)2
SPT5
YVC1
PHB2
YPL009C
PGA1
YNL234W
YPR153W
YHR007C−A_2
ACB1
RIB2
YHL015W−A_2
NOP56
YFL041W−A_1
YER180C−A_1
YCL012C
YLR211C_2
YMR001C−A_1
YOL019W−A_1
KAR3
YML058W−A_1
Gene Name
YPD.K.Shift
HL.K.INTERACTION
CSH.K.INTERACTION
HL.AS.K.INTERACTION
YPEG.K.Shift
HLEG.K.INTERACTION
CSHEG.K.INTERACTION
HLEG.AS.K.INTERACTION
YPD.L.SHIFT
HL.L.INTERACTION
CSH.L.INTERACTION
HL.AS.L.INTERACTION
YPEG.L.SHIFT
HLEG.L.INTERACTION
CSHEG.L.INTERACTION
HLEG.AS.L.INTERACTION
Type of Media

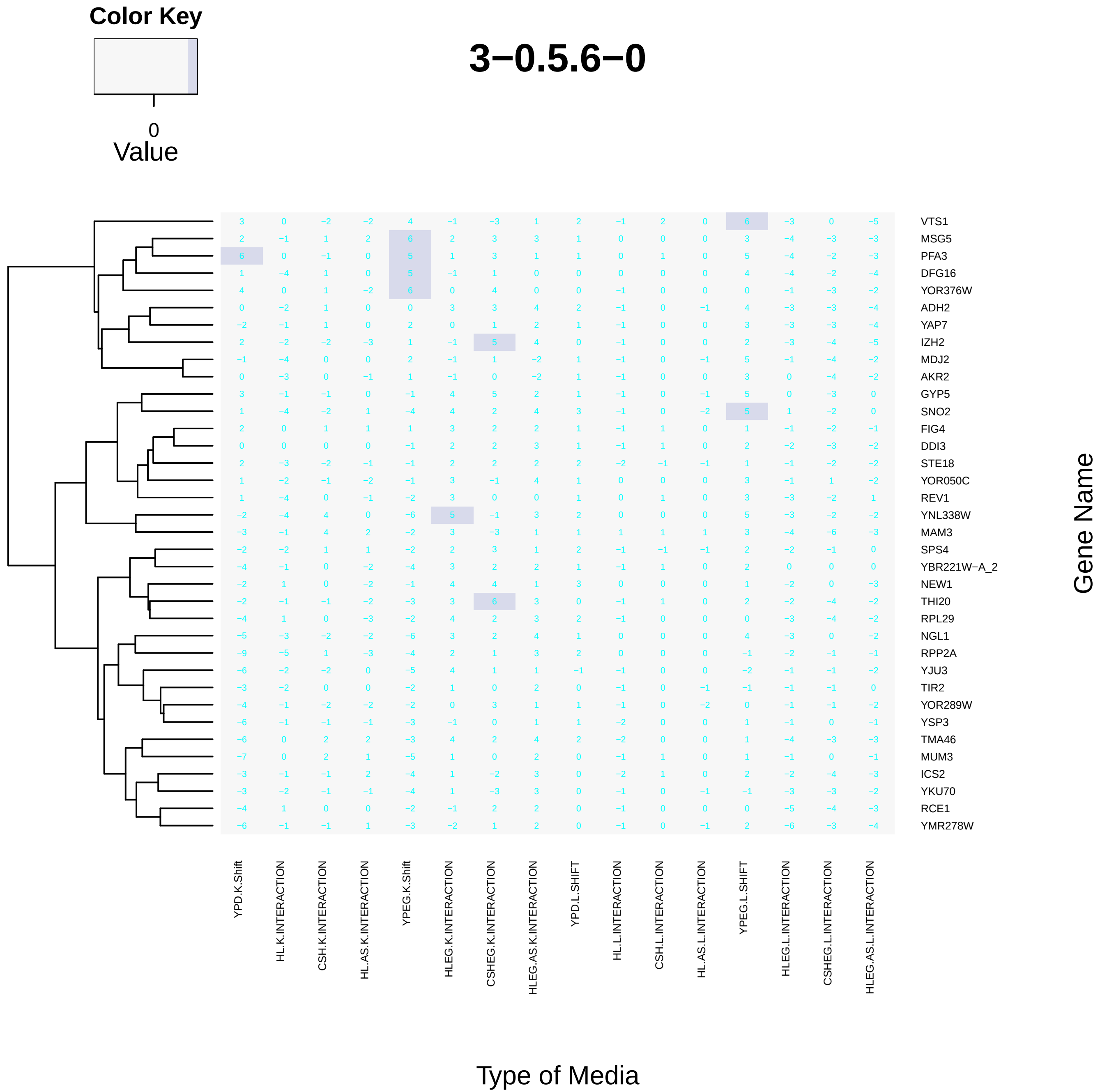
Color Key
0
Value
3−0.5.6−0
VTS1 3 0 −2 −2 4 −1 −3 1 2 −1 2 0 6 −3 0 −5
MSG5 2 −1 1 2 6 2 3 3 1 0 0 0 3 −4 −3 −3
PFA3 6 0 −1 0 5 1 3 1 1 0 1 0 5 −4 −2 −3
DFG16 1 −4 1 0 5 −1 1 0 0 0 0 0 4 −4 −2 −4
YOR376W 4 0 1 −2 6 0 4 0 0 −1 0 0 0 −1 −3 −2
ADH2 0 −2 1 0 0 3 3 4 2 −1 0 −1 4 −3 −3 −4
YAP7 −2 −1 1 0 2 0 1 2 1 −1 0 0 3 −3 −3 −4
IZH2 2 −2 −2 −3 1 −1 5 4 0 −1 0 0 2 −3 −4 −5
MDJ2 −1 −4 0 0 2 −1 1 −2 1 −1 0 −1 5 −1 −4 −2
AKR2 0 −3 0 −1 1 −1 0 −2 1 −1 0 0 3 0 −4 −2
GYP5 3 −1 −1 0 −1 4 5 2 1 −1 0 −1 5 0 −3 0
SNO2 1 −4 −2 1 −4 4 2 4 3 −1 0 −2 5 1 −2 0
FIG4 2 0 1 1 1 3 2 2 1 −1 1 0 1 −1 −2 −1
DDI3 0 0 0 0 −1 2 2 3 1 −1 1 0 2 −2 −3 −2
STE18 2 −3 −2 −1 −1 2 2 2 2 −2 −1 −1 1 −1 −2 −2
YOR050C 1 −2 −1 −2 −1 3 −1 4 1 0 0 0 3 −1 1 −2
REV1 1 −4 0 −1 −2 3 0 0 1 0 1 0 3 −3 −2 1
YNL338W −2 −4 4 0 −6 5 −1 3 2 0 0 0 5 −3 −2 −2
MAM3 −3 −1 4 2 −2 3 −3 1 1 1 1 1 3 −4 −6 −3
SPS4 −2 −2 1 1 −2 2 3 1 2 −1 −1 −1 2 −2 −1 0
YBR221W−A_2 −4 −1 0 −2 −4 3 2 2 1 −1 1 0 2 0 0 0
NEW1 −2 1 0 −2 −1 4 4 1 3 0 0 0 1 −2 0 −3
THI20 −2 −1 −1 −2 −3 3 6 3 0 −1 1 0 2 −2 −4 −2
RPL29 −4 1 0 −3 −2 4 2 3 2 −1 0 0 0 −3 −4 −2
NGL1 −5 −3 −2 −2 −6 3 2 4 1 0 0 0 4 −3 0 −2
RPP2A −9 −5 1 −3 −4 2 1 3 2 0 0 0 −1 −2 −1 −1
YJU3 −6 −2 −2 0 −5 4 1 1 −1 −1 0 0 −2 −1 −1 −2
TIR2 −3 −2 0 0 −2 1 0 2 0 −1 0 −1 −1 −1 −1 0
YOR289W −4 −1 −2 −2 −2 0 3 1 1 −1 0 −2 0 −1 −1 −2
YSP3 −6 −1 −1 −1 −3 −1 0 1 1 −2 0 0 1 −1 0 −1
TMA46 −6 0 2 2 −3 4 2 4 2 −2 0 0 1 −4 −3 −3
MUM3 −7 0 2 1 −5 1 0 2 0 −1 1 0 1 −1 0 −1
ICS2 −3 −1 −1 2 −4 1 −2 3 0 −2 1 0 2 −2 −4 −3
YKU70 −3 −2 −1 −1 −4 1 −3 3 0 −1 0 −1 −1 −3 −3 −2
RCE1 −4 1 0 0 −2 −1 2 2 0 −1 0 0 0 −5 −4 −3
YMR278W −6 −1 −1 1 −3 −2 1 2 0 −1 0 −1 2 −6 −3 −4
Gene Name
YPD.K.Shift
HL.K.INTERACTION
CSH.K.INTERACTION
HL.AS.K.INTERACTION
YPEG.K.Shift
HLEG.K.INTERACTION
CSHEG.K.INTERACTION
HLEG.AS.K.INTERACTION
YPD.L.SHIFT
HL.L.INTERACTION
CSH.L.INTERACTION
HL.AS.L.INTERACTION
YPEG.L.SHIFT
HLEG.L.INTERACTION
CSHEG.L.INTERACTION
HLEG.AS.L.INTERACTION
Type of Media

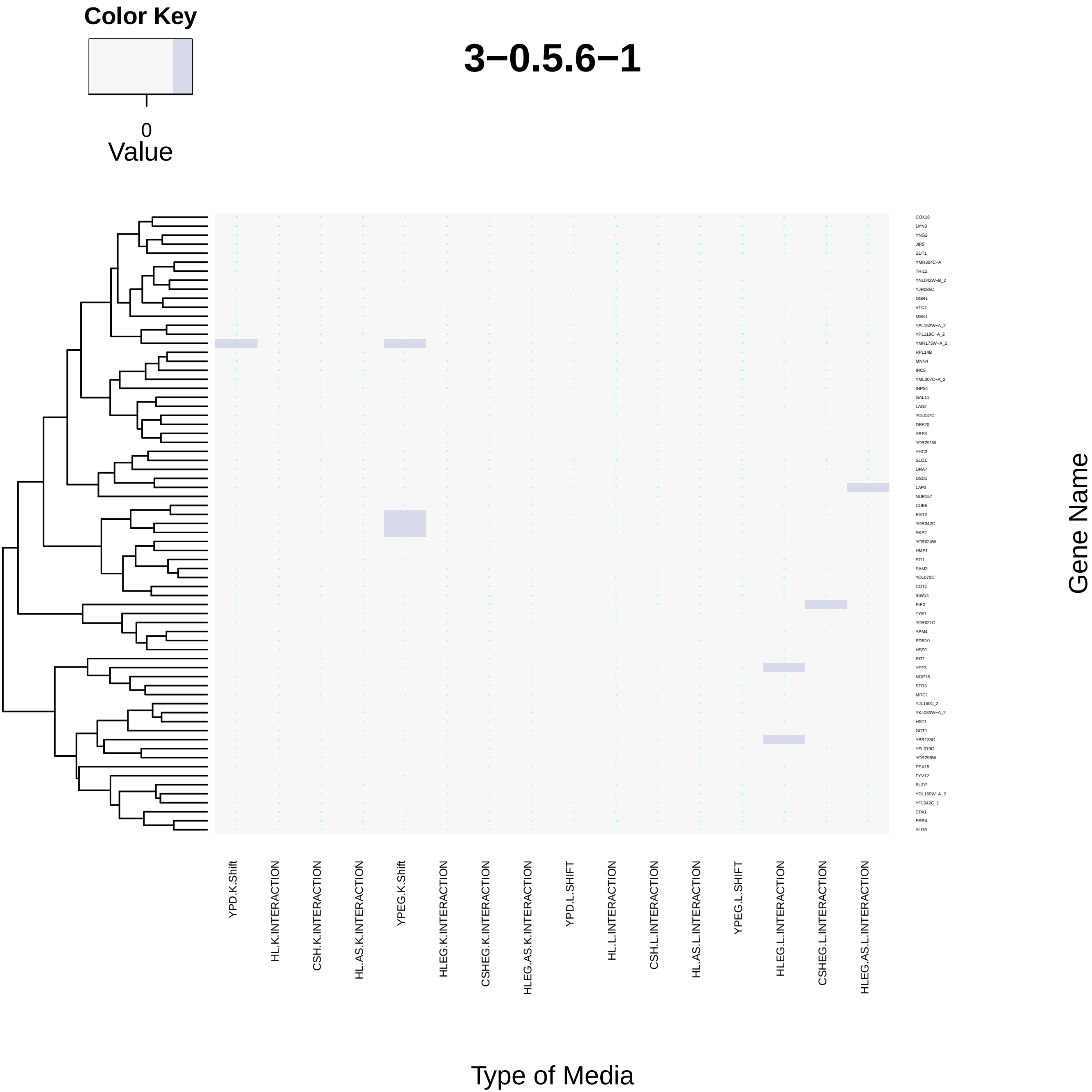
Color Key
0
Value
3−0.5.6−1
COX18
DYN3
YNG2
JIP5
SDT1
YMR304C−A
THI12
YNL042W−B_2
YJR085C
DGR1
VTC4
MEK1
YPL152W−A_2
YPL119C−A_2
YMR175W−A_2
RPL14B
MNN4
IRC5
YML007C−A_2
INP54
GAL11
LAG2
YOL047C
DBF20
ARF3
YOR291W
YHC3
SLO1
URA7
DSD1
LAP3
NUP157
CUE5
EGT2
YOR342C
SKP2
YOR024W
HMS1
STI1
SAM3
YOL070C
COT1
SIW14
PIP2
TYE7
YOR021C
APM4
PDR10
HSD1
RIT1
YEF3
NOP15
STR3
MRC1
YJL160C_2
YKL033W−A_2
HST1
GOT1
YBR138C
YFL019C
YOR296W
PEX15
FYV12
BUD7
YDL159W−A_2
YFL042C_1
CPA1
ERP4
ALG6
Gene Name
YPD.K.Shift
HL.K.INTERACTION
CSH.K.INTERACTION
HL.AS.K.INTERACTION
YPEG.K.Shift
HLEG.K.INTERACTION
CSHEG.K.INTERACTION
HLEG.AS.K.INTERACTION
YPD.L.SHIFT
HL.L.INTERACTION
CSH.L.INTERACTION
HL.AS.L.INTERACTION
YPEG.L.SHIFT
HLEG.L.INTERACTION
CSHEG.L.INTERACTION
HLEG.AS.L.INTERACTION
Type of Media

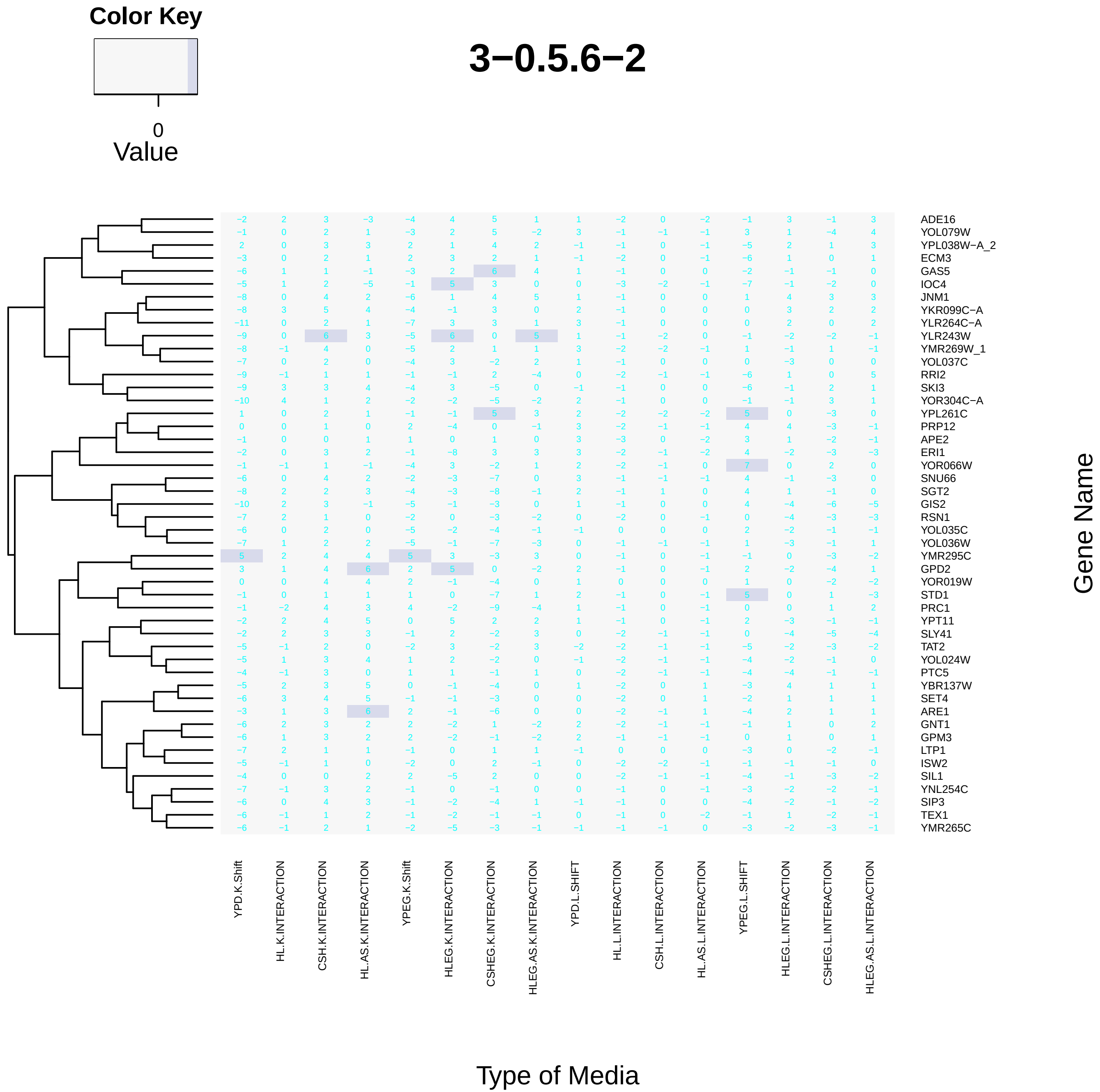

Color Key
0
Value
3−0.5.6−2
ADE16
YOL079W
YPL038W−A_2
ECM3
GAS5
IOC4
JNM1
YKR099C−A
YLR264C−A
YLR243W
YMR269W_1
YOL037C
RRI2
SKI3
YOR304C−A
YPL261C
PRP12
APE2
ERI1
YOR066W
SNU66
SGT2
GIS2
RSN1
YOL035C
YOL036W
YMR295C
GPD2
YOR019W
STD1
PRC1
YPT11
SLY41
TAT2
YOL024W
PTC5
YBR137W
SET4
ARE1
GNT1
GPM3
LTP1
ISW2
SIL1
YNL254C
SIP3
TEX1
YMR265C
Gene Name
YPD.K.Shift
HL.K.INTERACTION
CSH.K.INTERACTION
HL.AS.K.INTERACTION
YPEG.K.Shift
HLEG.K.INTERACTION
CSHEG.K.INTERACTION
HLEG.AS.K.INTERACTION
YPD.L.SHIFT
HL.L.INTERACTION
CSH.L.INTERACTION
HL.AS.L.INTERACTION
YPEG.L.SHIFT
HLEG.L.INTERACTION
CSHEG.L.INTERACTION
HLEG.AS.L.INTERACTION
Type of Media

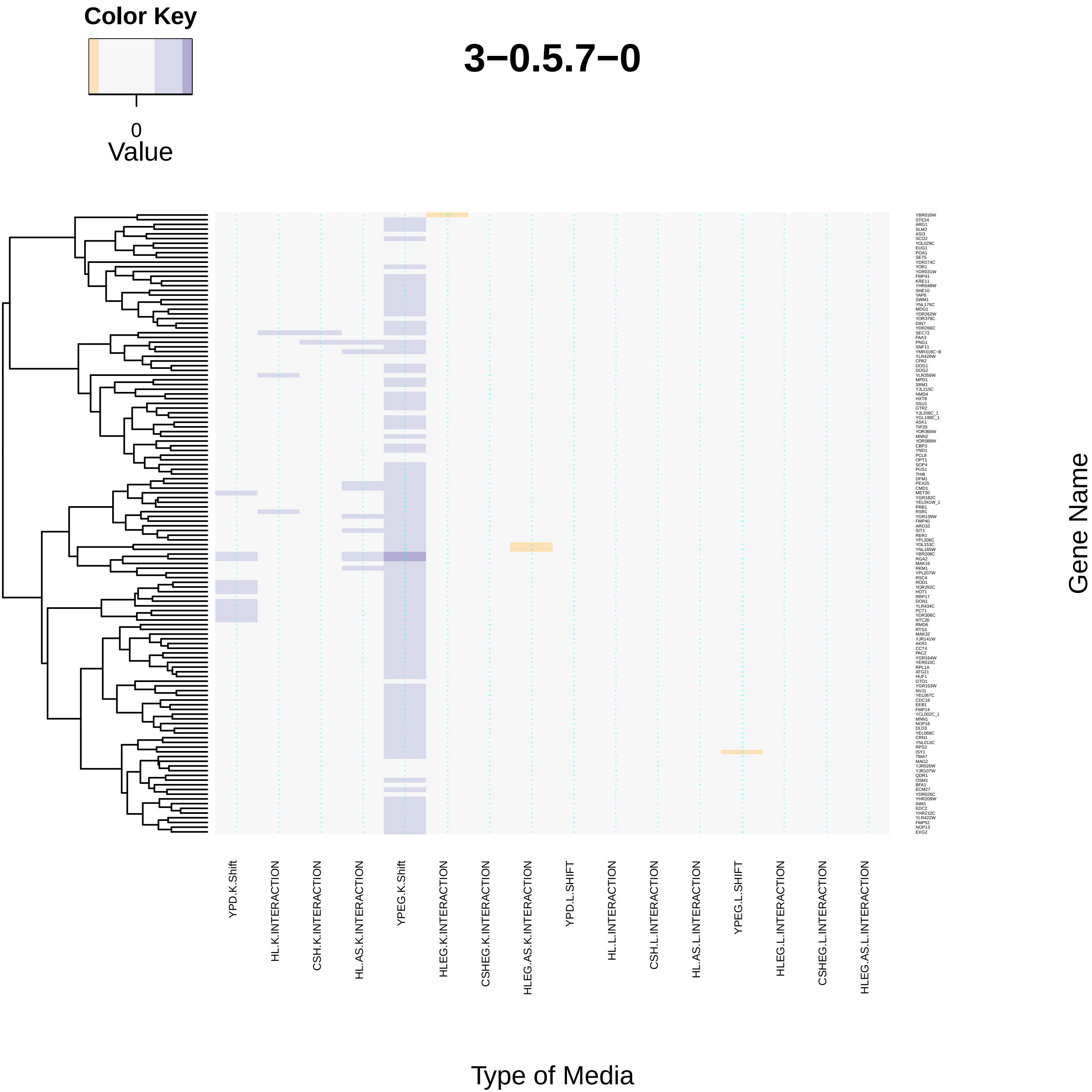
Color Key
0
Value
3−0.5.7−0
YBR016W
STE24
ARG1
SLM2
ASI3
SCO2
YOL029C
EUG1
POA1
SET5
YDR274C
YOR1
YDR031W
FMP41
KRE11
YHR048W
SHE10
YAP6
SWM1
YNL176C
MDG1
YDR262W
YOR379C
DIN7
YDR266C
SEC72
FAA3
PNG1
SNF11
YMR316C−B
YLR426W
CPA2
DOG1
DOG2
YLR356W
MPD1
SRM1
YJL215C
NMD4
HXT8
SSU1
GTR2
YJL206C_1
YGL199C_1
ASK1
TIP20
YOR366W
MNN2
YOR088W
CBP3
YND1
PCL8
OPT1
SOP4
PUS1
THI6
DFM1
PEX25
CMD1
MET30
YGR182C
YEL041W_1
PRB1
RSR1
YGR139W
FMP40
ARO10
SIT1
RER1
YPL206C
YOL153C
YNL165W
YBR208C
RGA2
MAK16
RKM1
YPL207W
RSC4
ROD1
YOR292C
HOT1
RRP17
DON1
YLR434C
PCT1
YDR306C
NTC20
RMD6
RTS3
MAK32
YJR141W
AKR1
CCT4
PAC2
YGR164W
YER010C
RPL1A
ATG21
HUF1
GTO1
YGR153W
NVJ1
YEL067C
CDC16
EEB1
FMP14
YCL002C_1
MNN1
NOP16
DLD3
YEL068C
CRN1
YNL013C
RPS2
ISY1
TMA7
MAG2
YJR026W
YJR107W
QDR1
OSM1
BFA1
ECM27
YDR026C
YHR209W
INM1
EDC2
YHR210C
YLR422W
FMP52
NOP13
EXG2
Gene Name
YPD.K.Shift
HL.K.INTERACTION
CSH.K.INTERACTION
HL.AS.K.INTERACTION
YPEG.K.Shift
HLEG.K.INTERACTION
CSHEG.K.INTERACTION
HLEG.AS.K.INTERACTION
YPD.L.SHIFT
HL.L.INTERACTION
CSH.L.INTERACTION
HL.AS.L.INTERACTION
YPEG.L.SHIFT
HLEG.L.INTERACTION
CSHEG.L.INTERACTION
HLEG.AS.L.INTERACTION
Type of Media

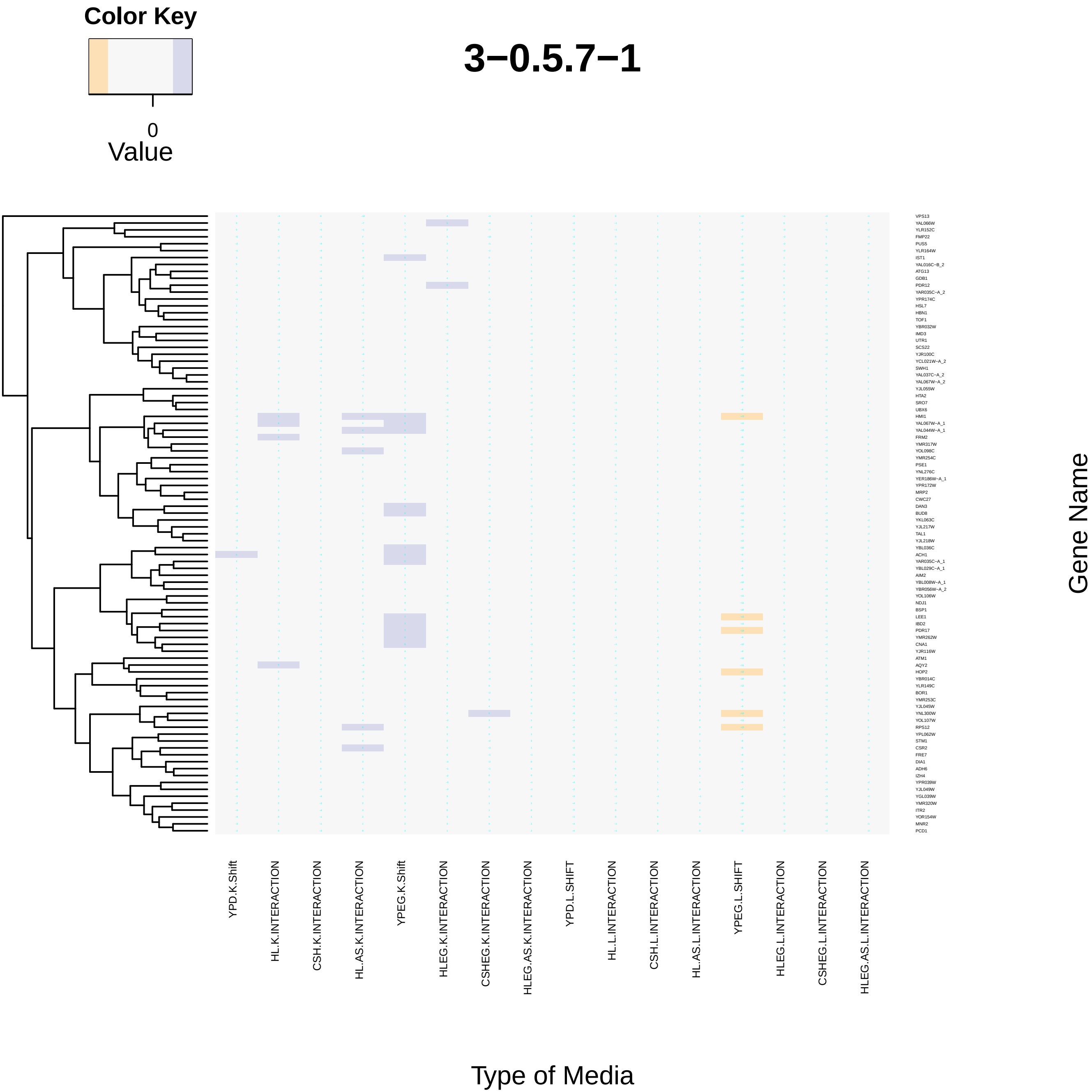
Color Key
0
Value
3−0.5.7−1
Gene Name
Type of Media
YPD.K.Shift
HL.K.INTERACTION
CSH.K.INTERACTION
HL.AS.K.INTERACTION
YPEG.K.Shift
HLEG.K.INTERACTION
CSHEG.K.INTERACTION
HLEG.AS.K.INTERACTION
YPD.L.SHIFT
HL.L.INTERACTION
CSH.L.INTERACTION
HL.AS.L.INTERACTION
YPEG.L.SHIFT
HLEG.L.INTERACTION
CSHEG.L.INTERACTION
HLEG.AS.L.INTERACTION
VPS13
YAL066W
YLR152C
FMP22
PUS5
YLR164W
IST1
YAL016C−B_2
ATG13
GDB1
PDR12
YAR035C−A_2
YPR174C
HSL7
HBN1
TOF1
YBR032W
IMD3
UTR1
SCS22
YJR100C
YCL021W−A_2
SWH1
YAL037C−A_2
YAL067W−A_2
YJL055W
HTA2
SRO7
UBX6
HMI1
YAL067W−A_1
YAL044W−A_1
FRM2
YMR317W
YOL098C
YMR254C
PSE1
YNL276C
YER186W−A_1
YPR172W
MRP2
CWC27
DAN3
BUD8
YKL063C
YJL217W
TAL1
YJL218W
YBL036C
ACH1
YAR035C−A_1
YBL029C−A_1
AIM2
YBL008W−A_1
YBR056W−A_2
YOL106W
NDJ1
BSP1
LEE1
IBD2
PDR17
YMR262W
CNA1
YJR116W
ATM1
AQY2
HOP2
YBR014C
YLR149C
BOR1
YMR253C
YJL045W
YNL300W
YOL107W
RPS12
YPL062W
STM1
CSR2
FRE7
DIA1
ADH6
IZH4
YPR039W
YJL049W
YGL039W
YMR320W
ITR2
YOR154W
MNR2
PCD1

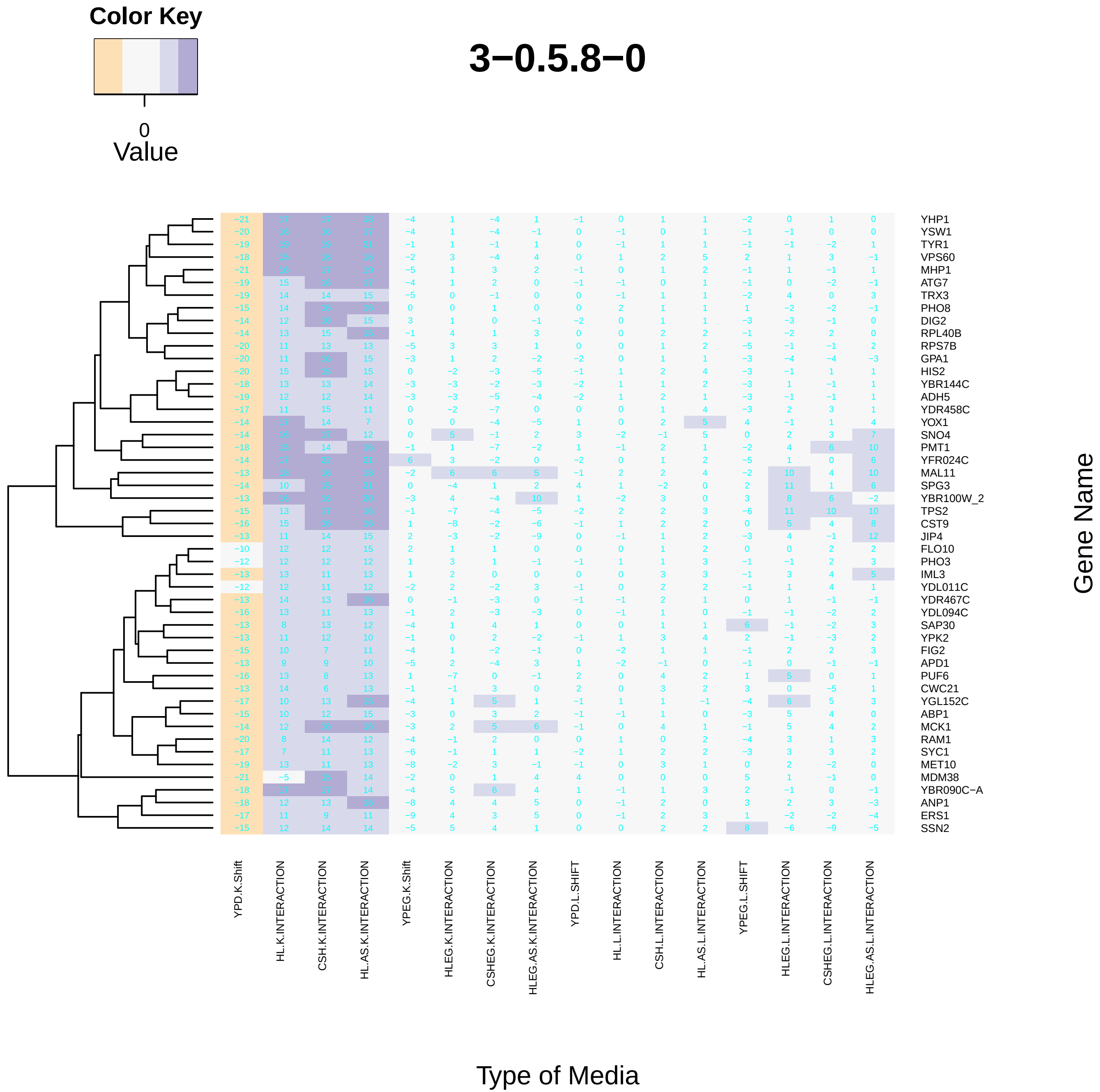

Color Key
0
Value
3−0.5.8−0
YHP1
YSW1
TYR1
VPS60
MHP1
ATG7
TRX3
PHO8
DIG2
RPL40B
RPS7B
GPA1
HIS2
YBR144C
ADH5
YDR458C
YOX1
SNO4
PMT1
YFR024C
MAL11
SPG3
YBR100W_2
TPS2
CST9
JIP4
FLO10
PHO3
IML3
YDL011C
YDR467C
YDL094C
SAP30
YPK2
FIG2
APD1
PUF6
CWC21
YGL152C
ABP1
MCK1
RAM1
SYC1
MET10
MDM38
YBR090C−A
ANP1
ERS1
SSN2
Gene Name
YPD.K.Shift
HL.K.INTERACTION
CSH.K.INTERACTION
HL.AS.K.INTERACTION
YPEG.K.Shift
HLEG.K.INTERACTION
CSHEG.K.INTERACTION
HLEG.AS.K.INTERACTION
YPD.L.SHIFT
HL.L.INTERACTION
CSH.L.INTERACTION
HL.AS.L.INTERACTION
YPEG.L.SHIFT
HLEG.L.INTERACTION
CSHEG.L.INTERACTION
HLEG.AS.L.INTERACTION
Type of Media

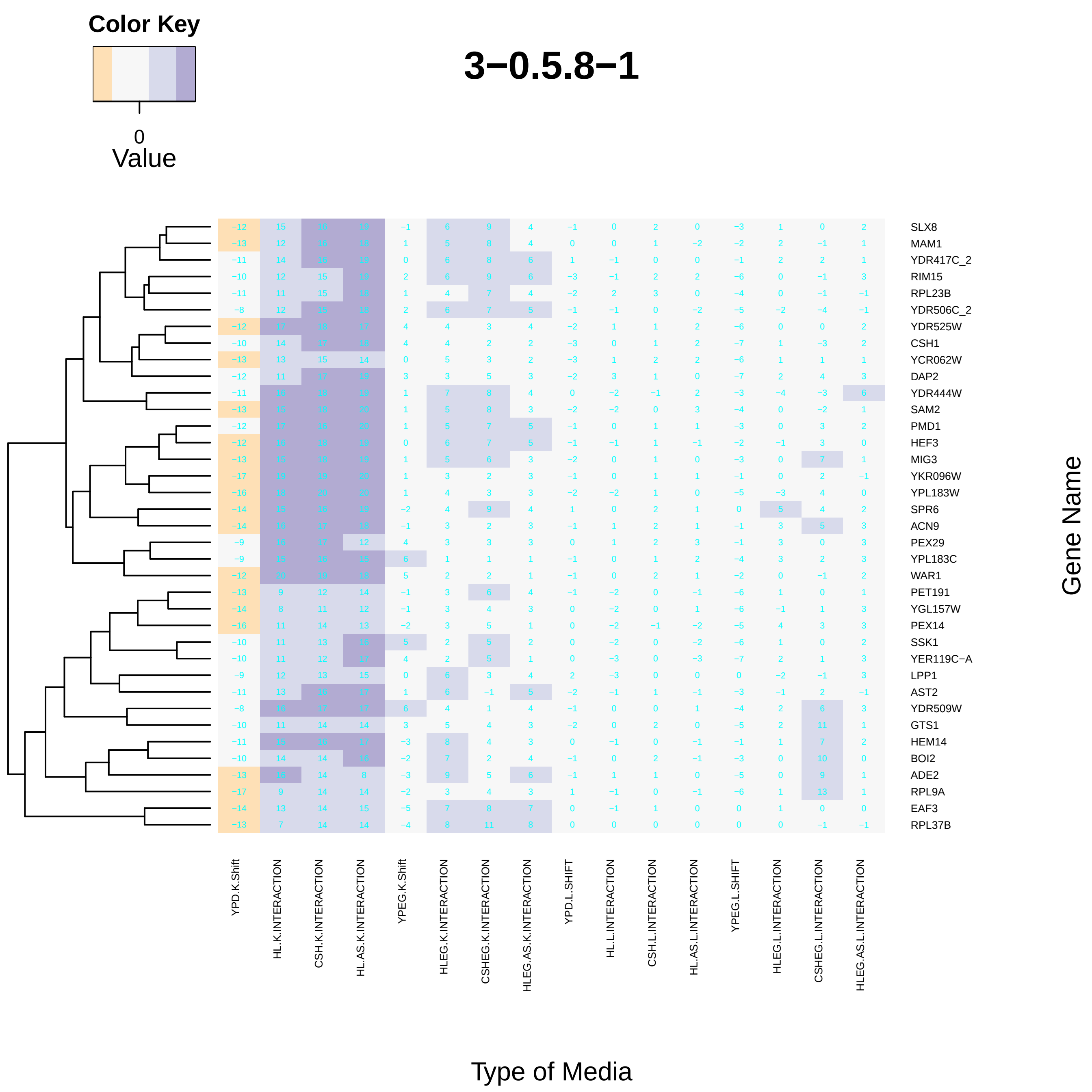

Color Key
0
Value
3−0.5.8−1
SLX8
MAM1
YDR417C_2
RIM15
RPL23B
YDR506C_2
YDR525W
CSH1
YCR062W
DAP2
YDR444W
SAM2
PMD1
HEF3
MIG3
YKR096W
YPL183W
SPR6
ACN9
PEX29
YPL183C
WAR1
PET191
YGL157W
PEX14
SSK1
YER119C−A
LPP1
AST2
YDR509W
GTS1
HEM14
BOI2
ADE2
RPL9A
EAF3
RPL37B
Gene Name
YPD.K.Shift
HL.K.INTERACTION
CSH.K.INTERACTION
HL.AS.K.INTERACTION
YPEG.K.Shift
HLEG.K.INTERACTION
CSHEG.K.INTERACTION
HLEG.AS.K.INTERACTION
YPD.L.SHIFT
HL.L.INTERACTION
CSH.L.INTERACTION
HL.AS.L.INTERACTION
YPEG.L.SHIFT
HLEG.L.INTERACTION
CSHEG.L.INTERACTION
HLEG.AS.L.INTERACTION
Type of Media

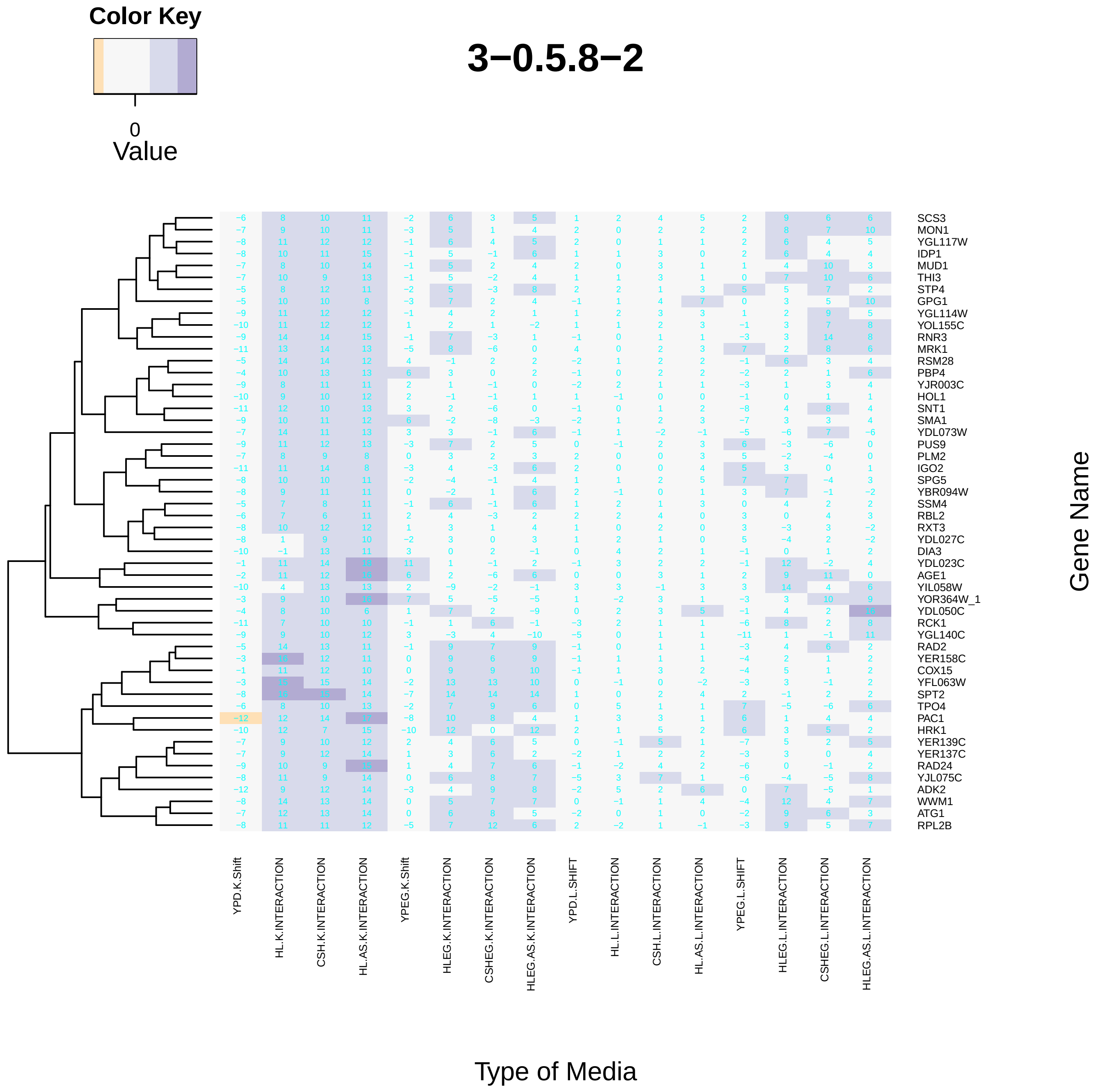

3−0.5.8−2
Color Key
0
Value
Gene Name
Type of Media
YPD.K.Shift
HL.K.INTERACTION
CSH.K.INTERACTION
HL.AS.K.INTERACTION
YPEG.K.Shift
HLEG.K.INTERACTION
CSHEG.K.INTERACTION
HLEG.AS.K.INTERACTION
YPD.L.SHIFT
HL.L.INTERACTION
CSH.L.INTERACTION
HL.AS.L.INTERACTION
YPEG.L.SHIFT
HLEG.L.INTERACTION
CSHEG.L.INTERACTION
HLEG.AS.L.INTERACTION
SCS3
MON1
YGL117W
IDP1
MUD1
THI3
STP4
GPG1
YGL114W
YOL155C
RNR3
MRK1
RSM28
PBP4
YJR003C
HOL1
SNT1
SMA1
YDL073W
PUS9
PLM2
IGO2
SPG5
YBR094W
SSM4
RBL2
RXT3
YDL027C
DIA3
YDL023C
AGE1
YIL058W
YOR364W_1
YDL050C
RCK1
YGL140C
RAD2
YER158C
COX15
YFL063W
SPT2
TPO4
PAC1
HRK1
YER139C
YER137C
RAD24
YJL075C
ADK2
WWM1
ATG1
RPL2B

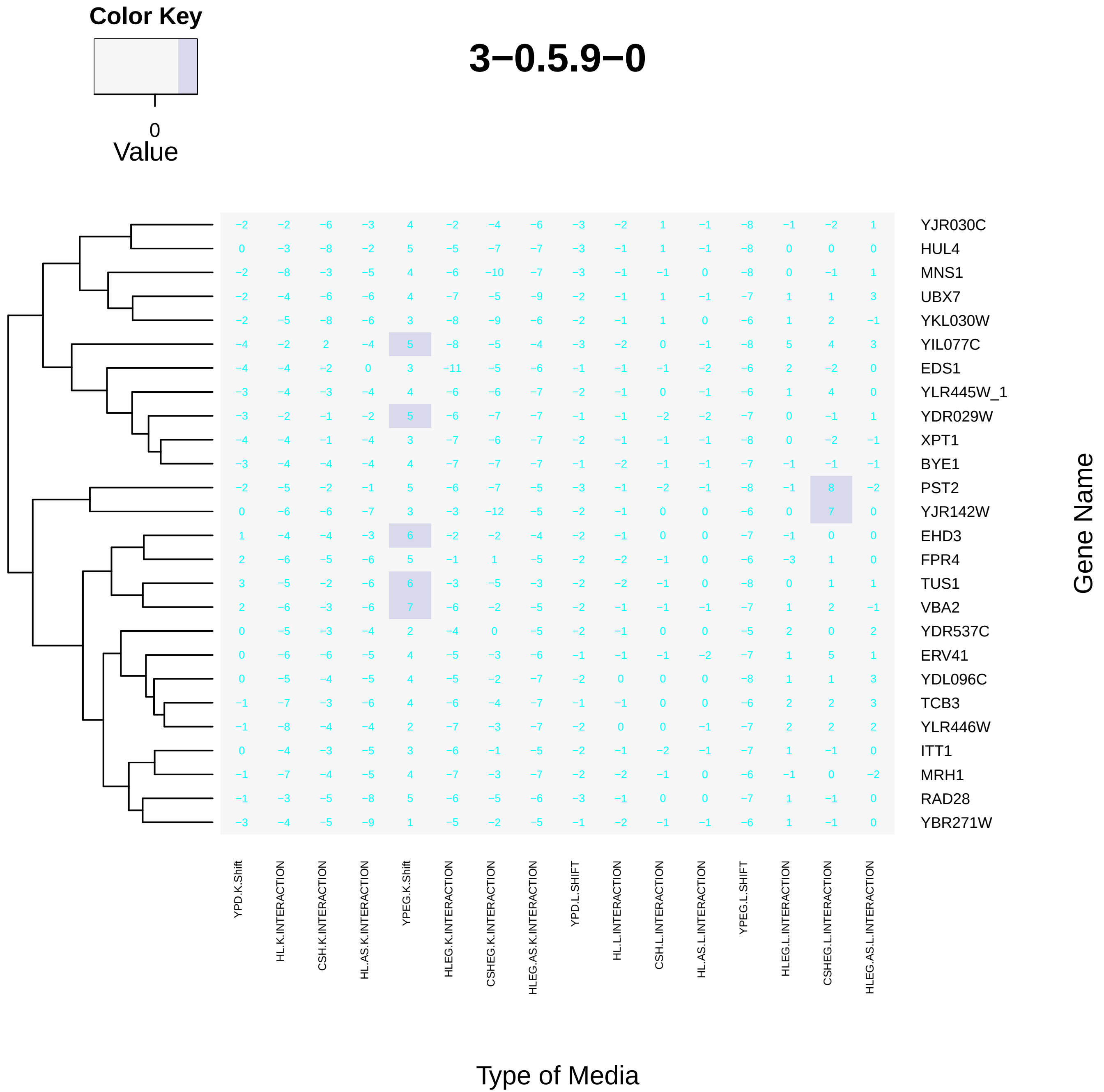

Color Key
0
Value
3−0.5.9−0
YJR030C
HUL4
MNS1
UBX7
YKL030W
YIL077C
EDS1
YLR445W_1
YDR029W
XPT1
BYE1
PST2
YJR142W
EHD3
FPR4
TUS1
VBA2
YDR537C
ERV41
YDL096C
TCB3
YLR446W
ITT1
MRH1
RAD28
YBR271W
Gene Name
YPD.K.Shift
HL.K.INTERACTION
CSH.K.INTERACTION
HL.AS.K.INTERACTION
YPEG.K.Shift
HLEG.K.INTERACTION
CSHEG.K.INTERACTION
HLEG.AS.K.INTERACTION
YPD.L.SHIFT
HL.L.INTERACTION
CSH.L.INTERACTION
HL.AS.L.INTERACTION
YPEG.L.SHIFT
HLEG.L.INTERACTION
CSHEG.L.INTERACTION
HLEG.AS.L.INTERACTION
Type of Media

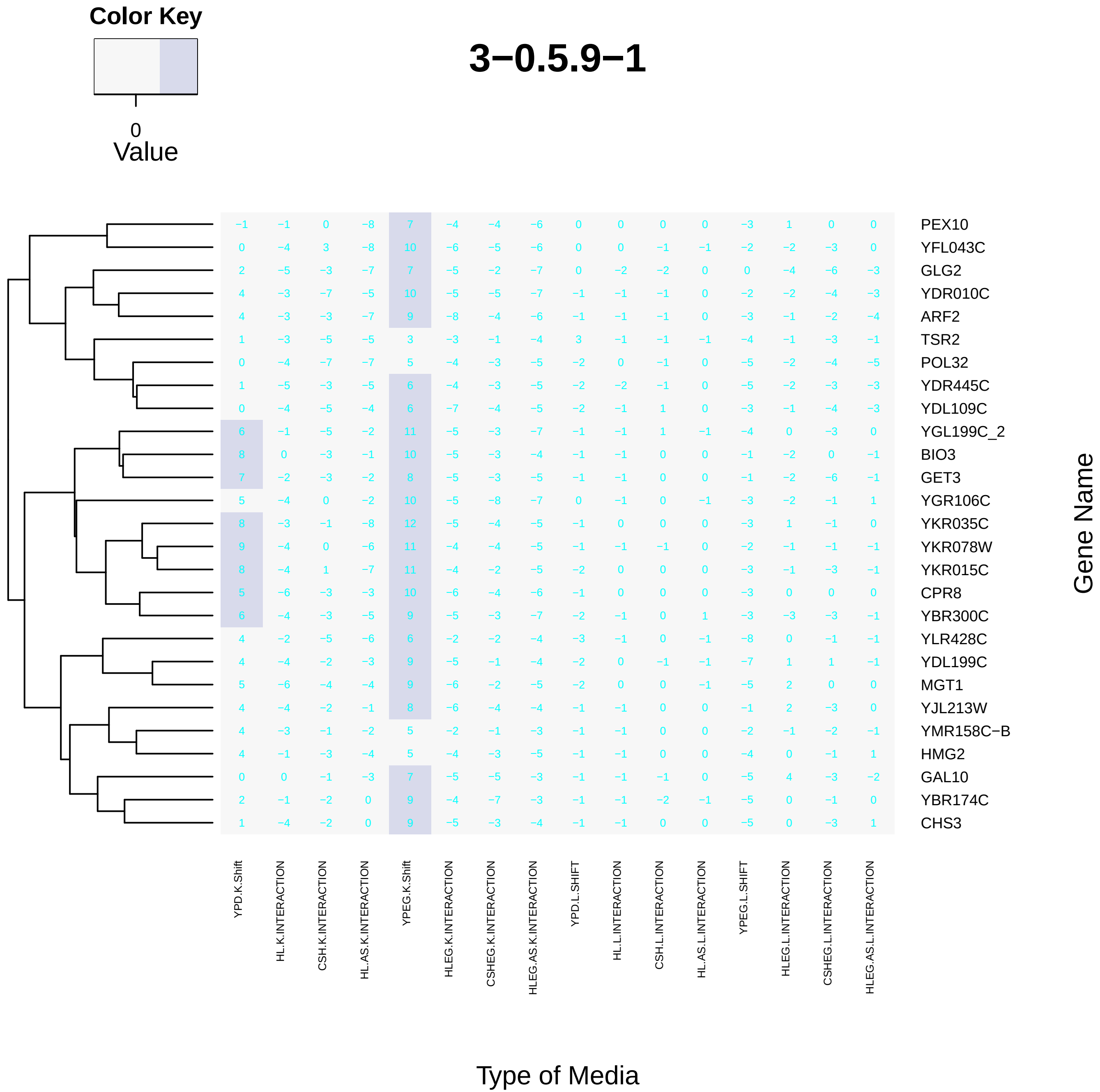
Color Key
0
Value
3−0.5.9−1
YPD.K.Shift
HL.K.INTERACTION
CSH.K.INTERACTION
HL.AS.K.INTERACTION
YPEG.K.Shift
HLEG.K.INTERACTION
CSHEG.K.INTERACTION
HLEG.AS.K.INTERACTION
YPD.L.SHIFT
HL.L.INTERACTION
CSH.L.INTERACTION
HL.AS.L.INTERACTION
YPEG.L.SHIFT
HLEG.L.INTERACTION
CSHEG.L.INTERACTION
HLEG.AS.L.INTERACTION
PEX10 −1 −1 0 −8 7 −4 −4 −6 0 0 0 0 −3 1 0 0
YFL043C 0 −4 3 −8 10 −6 −5 −6 0 0 −1 −1 −2 −2 −3 0
GLG2 2 −5 −3 −7 7 −5 −2 −7 0 −2 −2 0 0 −4 −6 −3
YDR010C 4 −3 −7 −5 10 −5 −5 −7 −1 −1 −1 0 −2 −2 −4 −3
ARF2 4 −3 −3 −7 9 −8 −4 −6 −1 −1 −1 0 −3 −1 −2 −4
TSR2 1 −3 −5 −5 3 −3 −1 −4 3 −1 −1 −1 −4 −1 −3 −1
POL32 0 −4 −7 −7 5 −4 −3 −5 −2 0 −1 0 −5 −2 −4 −5
YDR445C 1 −5 −3 −5 6 −4 −3 −5 −2 −2 −1 0 −5 −2 −3 −3
YDL109C 0 −4 −5 −4 6 −7 −4 −5 −2 −1 1 0 −3 −1 −4 −3
YGL199C_2 6 −1 −5 −2 11 −5 −3 −7 −1 −1 1 −1 −4 0 −3 0
BIO3 8 0 −3 −1 10 −5 −3 −4 −1 −1 0 0 −1 −2 0 −1
GET3 7 −2 −3 −2 8 −5 −3 −5 −1 −1 0 0 −1 −2 −6 −1
YGR106C 5 −4 0 −2 10 −5 −8 −7 0 −1 0 −1 −3 −2 −1 1
YKR035C 8 −3 −1 −8 12 −5 −4 −5 −1 0 0 0 −3 1 −1 0
YKR078W 9 −4 0 −6 11 −4 −4 −5 −1 −1 −1 0 −2 −1 −1 −1
YKR015C 8 −4 1 −7 11 −4 −2 −5 −2 0 0 0 −3 −1 −3 −1
CPR8 5 −6 −3 −3 10 −6 −4 −6 −1 0 0 0 −3 0 0 0
YBR300C 6 −4 −3 −5 9 −5 −3 −7 −2 −1 0 1 −3 −3 −3 −1
YLR428C 4 −2 −5 −6 6 −2 −2 −4 −3 −1 0 −1 −8 0 −1 −1
YDL199C 4 −4 −2 −3 9 −5 −1 −4 −2 0 −1 −1 −7 1 1 −1
MGT1 5 −6 −4 −4 9 −6 −2 −5 −2 0 0 −1 −5 2 0 0
YJL213W 4 −4 −2 −1 8 −6 −4 −4 −1 −1 0 0 −1 2 −3 0
YMR158C−B 4 −3 −1 −2 5 −2 −1 −3 −1 −1 0 0 −2 −1 −2 −1
HMG2 4 −1 −3 −4 5 −4 −3 −5 −1 −1 0 0 −4 0 −1 1
GAL10 0 0 −1 −3 7 −5 −5 −3 −1 −1 −1 0 −5 4 −3 −2
YBR174C 2 −1 −2 0 9 −4 −7 −3 −1 −1 −2 −1 −5 0 −1 0
CHS3 1 −4 −2 0 9 −5 −3 −4 −1 −1 0 0 −5 0 −3 1
Gene Name
Type of Media

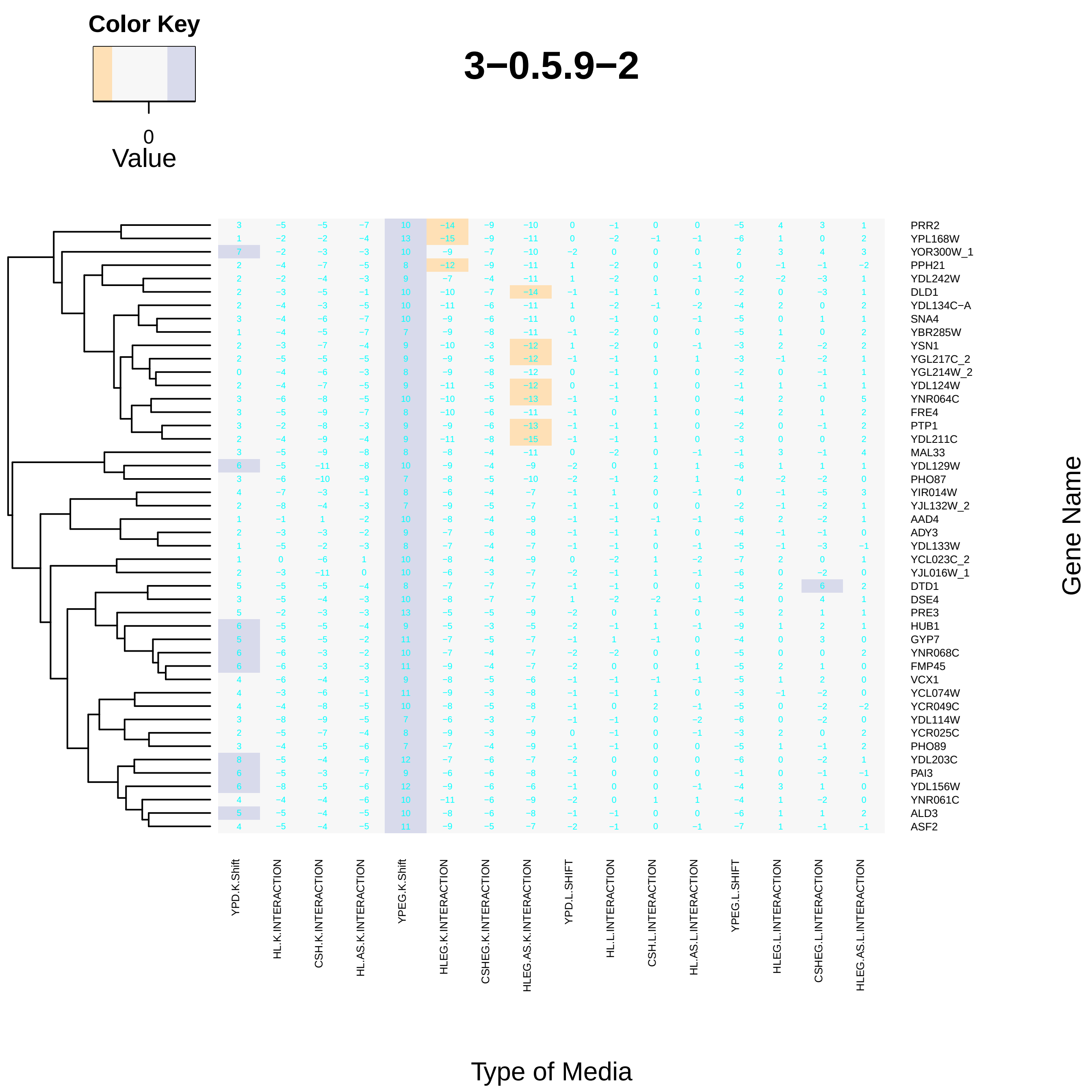
Color Key
0
Value
3−0.5.9−2
YPD.K.Shift
HL.K.INTERACTION
CSH.K.INTERACTION
HL.AS.K.INTERACTION
YPEG.K.Shift
HLEG.K.INTERACTION
CSHEG.K.INTERACTION
HLEG.AS.K.INTERACTION
YPD.L.SHIFT
HL.L.INTERACTION
CSH.L.INTERACTION
HL.AS.L.INTERACTION
YPEG.L.SHIFT
HLEG.L.INTERACTION
CSHEG.L.INTERACTION
HLEG.AS.L.INTERACTION
PRR2
YPL168W
YOR300W_1
PPH21
YDL242W
DLD1
YDL134C−A
SNA4
YBR285W
YSN1
YGL217C_2
YGL214W_2
YDL124W
YNR064C
FRE4
PTP1
YDL211C
MAL33
YDL129W
PHO87
YIR014W
YJL132W_2
AAD4
ADY3
YDL133W
YCL023C_2
YJL016W_1
DTD1
DSE4
PRE3
HUB1
GYP7
YNR068C
FMP45
VCX1
YCL074W
YCR049C
YDL114W
YCR025C
PHO89
YDL203C
PAI3
YDL156W
YNR061C
ALD3
ASF2
Gene Name
Type of Media

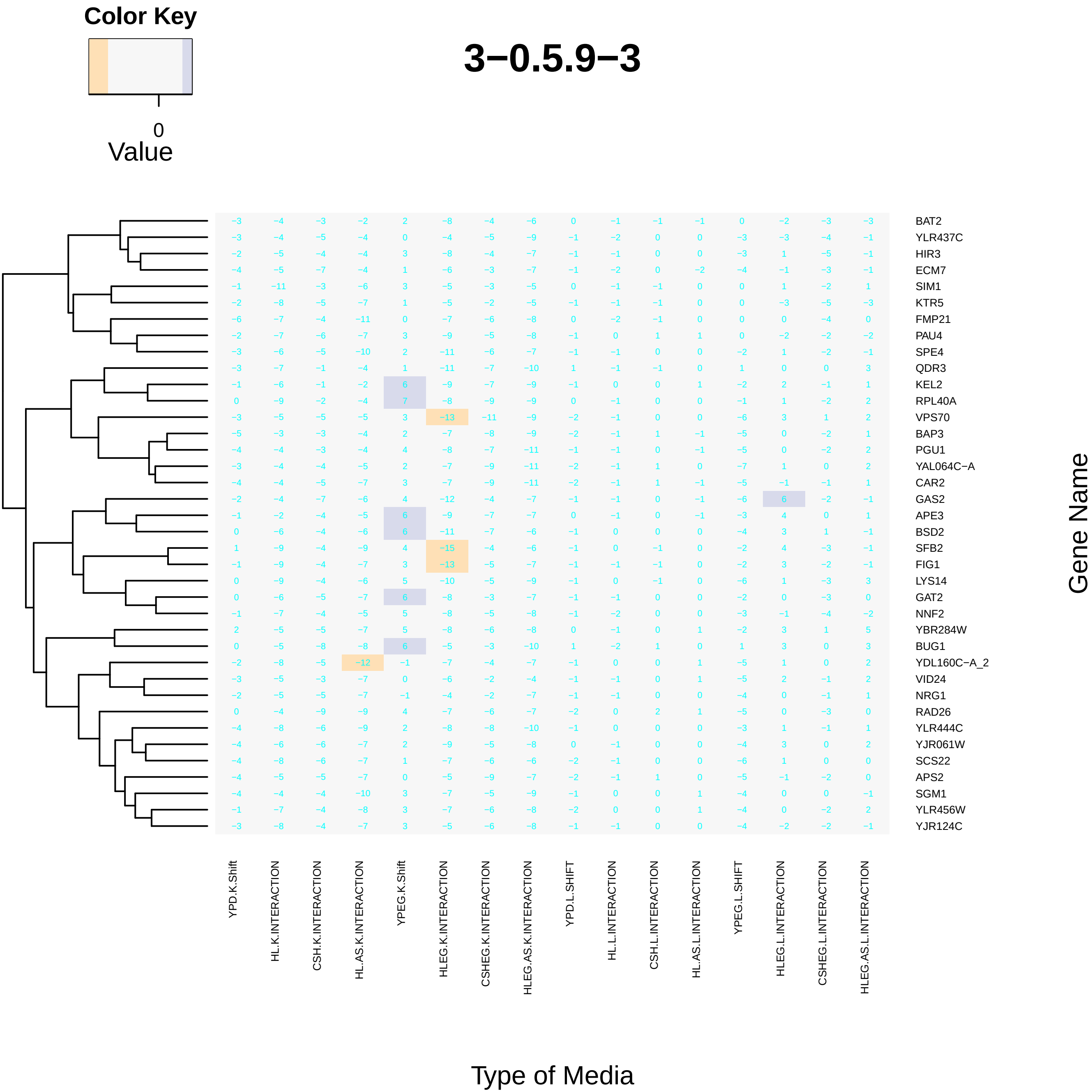
Color Key
0
Value
3−0.5.9−3
YPD.K.Shift HL.K.INTERACTION CSH.K.INTERACTION HL.AS.K.INTERACTION YPEG.K.Shift HLEG.K.INTERACTION CSHEG.K.INTERACTION HLEG.AS.K.INTERACTION YPD.L.SHIFT HL.L.INTERACTION CSH.L.INTERACTION HL.AS.L.INTERACTION YPEG.L.SHIFT HLEG.L.INTERACTION CSHEG.L.INTERACTION HLEG.AS.L.INTERACTION
BAT2 −3 −4 −3 −2 2 −8 −4 −6 0 −1 −1 −1 0 −2 −3 −3
YLR437C −3 −4 −5 −4 0 −4 −5 −9 −1 −2 0 0 −3 −3 −4 −1
HIR3 −2 −5 −4 −4 3 −8 −4 −7 −1 −1 0 0 −3 1 −5 −1
ECM7 −4 −5 −7 −4 1 −6 −3 −7 −1 −2 0 −2 −4 −1 −3 −1
SIM1 −1 −11 −3 −6 3 −5 −3 −5 0 −1 −1 0 0 1 −2 1
KTR5 −2 −8 −5 −7 1 −5 −2 −5 −1 −1 −1 0 0 −3 −5 −3
FMP21 −6 −7 −4 −11 0 −7 −6 −8 0 −2 −1 0 0 0 −4 0
PAU4 −2 −7 −6 −7 3 −9 −5 −8 −1 0 1 1 0 −2 −2 −2
SPE4 −3 −6 −5 −10 2 −11 −6 −7 −1 −1 0 0 −2 1 −2 −1
QDR3 −3 −7 −1 −4 1 −11 −7 −10 1 −1 −1 0 1 0 0 3
KEL2 −1 −6 −1 −2 6 −9 −7 −9 −1 0 0 1 −2 2 −1 1
RPL40A 0 −9 −2 −4 7 −8 −9 −9 0 −1 0 0 −1 1 −2 2
VPS70 −3 −5 −5 −5 3 −13 −11 −9 −2 −1 0 0 −6 3 1 2
BAP3 −5 −3 −3 −4 2 −7 −8 −9 −2 −1 1 −1 −5 0 −2 1
PGU1 −4 −4 −3 −4 4 −8 −7 −11 −1 −1 0 −1 −5 0 −2 2
YAL064C−A −3 −4 −4 −5 2 −7 −9 −11 −2 −1 1 0 −7 1 0 2
CAR2 −4 −4 −5 −7 3 −7 −9 −11 −2 −1 1 −1 −5 −1 −1 1
GAS2 −2 −4 −7 −6 4 −12 −4 −7 −1 −1 0 −1 −6 6 −2 −1
APE3 −1 −2 −4 −5 6 −9 −7 −7 0 −1 0 −1 −3 4 0 1
BSD2 0 −6 −4 −6 6 −11 −7 −6 −1 0 0 0 −4 3 1 −1
SFB2 1 −9 −4 −9 4 −15 −4 −6 −1 0 −1 0 −2 4 −3 −1
FIG1 −1 −9 −4 −7 3 −13 −5 −7 −1 −1 −1 0 −2 3 −2 −1
LYS14 0 −9 −4 −6 5 −10 −5 −9 −1 0 −1 0 −6 1 −3 3
GAT2 0 −6 −5 −7 6 −8 −3 −7 −1 −1 0 0 −2 0 −3 0
NNF2 −1 −7 −4 −5 5 −8 −5 −8 −1 −2 0 0 −3 −1 −4 −2
YBR284W 2 −5 −5 −7 5 −8 −6 −8 0 −1 0 1 −2 3 1 5
BUG1 0 −5 −8 −8 6 −5 −3 −10 1 −2 1 0 1 3 0 3
YDL160C−A_2 −2 −8 −5 −12 −1 −7 −4 −7 −1 0 0 1 −5 1 0 2
VID24 −3 −5 −3 −7 0 −6 −2 −4 −1 −1 0 1 −5 2 −1 2
NRG1 −2 −5 −5 −7 −1 −4 −2 −7 −1 −1 0 0 −4 0 −1 1
RAD26 0 −4 −9 −9 4 −7 −6 −7 −2 0 2 1 −5 0 −3 0
YLR444C −4 −8 −6 −9 2 −8 −8 −10 −1 0 0 0 −3 1 −1 1
YJR061W −4 −6 −6 −7 2 −9 −5 −8 0 −1 0 0 −4 3 0 2
SCS22 −4 −8 −6 −7 1 −7 −6 −6 −2 −1 0 0 −6 1 0 0
APS2 −4 −5 −5 −7 0 −5 −9 −7 −2 −1 1 0 −5 −1 −2 0
SGM1 −4 −4 −4 −10 3 −7 −5 −9 −1 0 0 1 −4 0 0 −1
YLR456W −1 −7 −4 −8 3 −7 −6 −8 −2 0 0 1 −4 0 −2 2
YJR124C −3 −8 −4 −7 3 −5 −6 −8 −1 −1 0 0 −4 −2 −2 −1
Gene Name
Type of Media

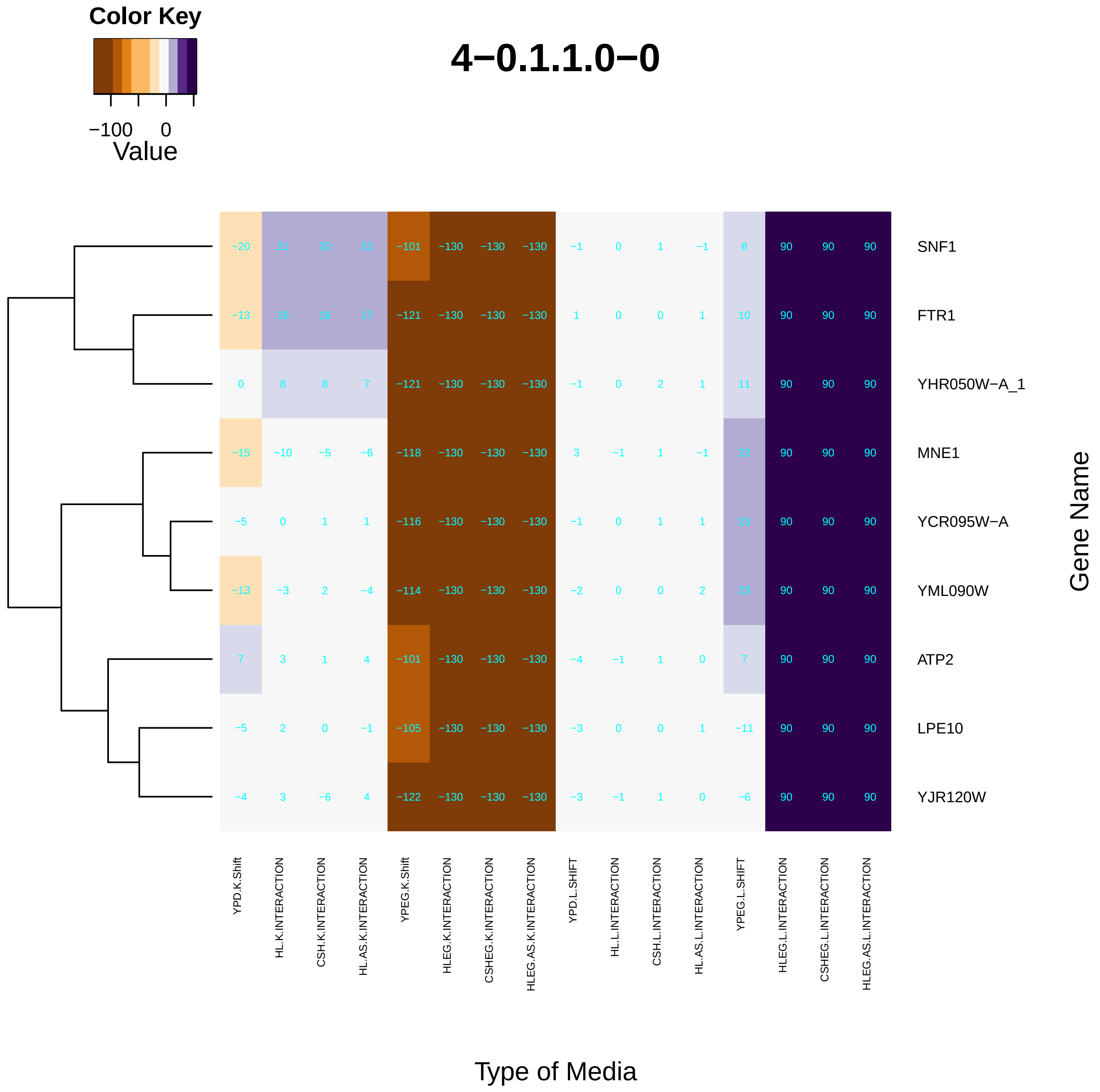
Color Key
−100
0
Value
4−0.1.1.0−0
−20 21 20 22 −101 −130 −130 −130 −1 0 1 −1 8 90 90 90
−13 16 18 17 −121 −130 −130 −130 1 0 0 1 10 90 90 90
0 8 8 7 −121 −130 −130 −130 −1 0 2 1 11 90 90 90
−15 −10 −5 −6 −118 −130 −130 −130 3 −1 1 −1 22 90 90 90
−5 0 1 1 −116 −130 −130 −130 −1 0 1 1 23 90 90 90
−13 −3 2 −4 −114 −130 −130 −130 −2 0 0 2 23 90 90 90
7 3 1 4 −101 −130 −130 −130 −4 −1 1 0 7 90 90 90
−5 2 0 −1 −105 −130 −130 −130 −3 0 0 1 −11 90 90 90
−4 3 −6 4 −122 −130 −130 −130 −3 −1 1 0 −6 90 90 90
SNF1
FTR1
YHR050W−A_1
MNE1
YCR095W−A
YML090W
ATP2
LPE10
YJR120W
Gene Name
YPD.K.Shift
HL.K.INTERACTION
CSH.K.INTERACTION
HL.AS.K.INTERACTION
YPEG.K.Shift
HLEG.K.INTERACTION
CSHEG.K.INTERACTION
HLEG.AS.K.INTERACTION
YPD.L.SHIFT
HL.L.INTERACTION
CSH.L.INTERACTION
HL.AS.L.INTERACTION
YPEG.L.SHIFT
HLEG.L.INTERACTION
CSHEG.L.INTERACTION
HLEG.AS.L.INTERACTION
Type of Media

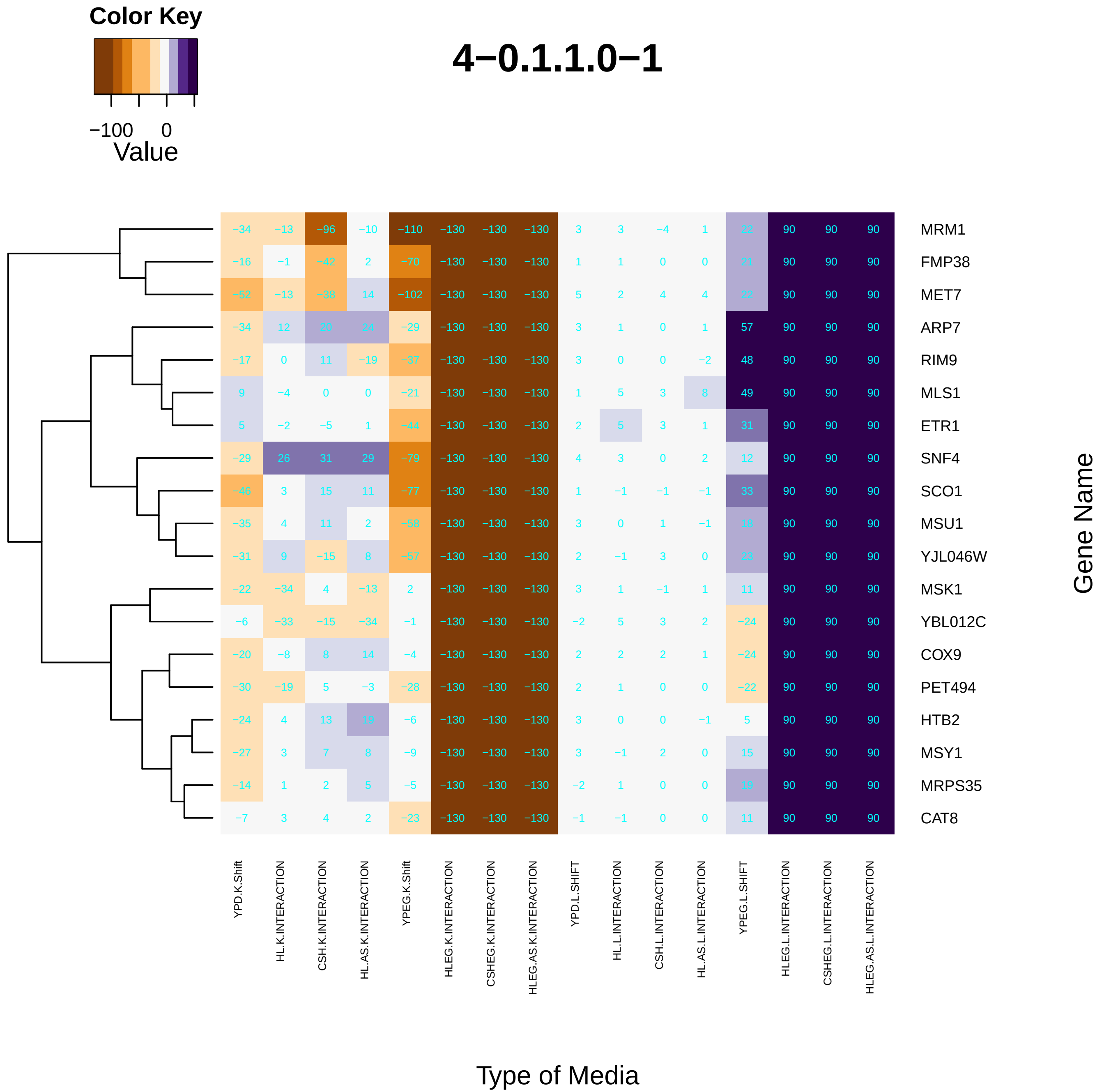
Color Key
−100 0
Value
4−0.1.1.0−1
YPD.K.Shift
HL.K.INTERACTION
CSH.K.INTERACTION
HL.AS.K.INTERACTION
YPEG.K.Shift
HLEG.K.INTERACTION
CSHEG.K.INTERACTION
HLEG.AS.K.INTERACTION
YPD.L.SHIFT
HL.L.INTERACTION
CSH.L.INTERACTION
HL.AS.L.INTERACTION
YPEG.L.SHIFT
HLEG.L.INTERACTION
CSHEG.L.INTERACTION
HLEG.AS.L.INTERACTION
MRM1 −34 −13 −96 −10 −110 −130 −130 −130 3 3 −4 1 22 90 90 90
FMP38 −16 −1 −42 2 −70 −130 −130 −130 1 1 0 0 21 90 90 90
MET7 −52 −13 −38 14 −102 −130 −130 −130 5 2 4 4 22 90 90 90
ARP7 −34 12 26 24 −29 −130 −130 −130 3 1 0 1 57 90 90 90
RIM9 −17 0 11 −19 −37 −130 −130 −130 3 0 0 −2 48 90 90 90
MLS1 9 −4 0 0 −21 −130 −130 −130 1 5 3 8 49 90 90 90
ETR1 5 −2 −5 1 −44 −130 −130 −130 2 5 3 1 31 90 90 90
SNF4 −29 26 31 29 −79 −130 −130 −130 4 3 0 2 12 90 90 90
SCO1 −46 3 15 11 −77 −130 −130 −130 1 −1 −1 −1 33 90 90 90
MSU1 −35 4 11 2 −58 −130 −130 −130 3 0 1 −1 18 90 90 90
YJL046W −31 9 −15 8 −57 −130 −130 −130 2 −1 3 0 23 90 90 90
MSK1 −22 −34 4 −13 2 −130 −130 −130 3 1 −1 1 11 90 90 90
YBL012C −6 −33 −15 −34 −1 −130 −130 −130 −2 5 3 2 −24 90 90 90
COX9 −20 −8 8 14 −4 −130 −130 −130 2 2 2 1 −24 90 90 90
PET494 −30 −19 5 −3 −28 −130 −130 −130 2 1 0 0 −22 90 90 90
HTB2 −24 4 13 19 −6 −130 −130 −130 3 0 0 −1 5 90 90 90
MSY1 −27 3 7 8 −9 −130 −130 −130 3 −1 2 0 15 90 90 90
MRPS35 −14 1 2 5 −5 −130 −130 −130 −2 1 0 0 19 90 90 90
CAT8 −7 3 4 2 −23 −130 −130 −130 −1 −1 0 0 11 90 90 90
Gene Name
Type of Media

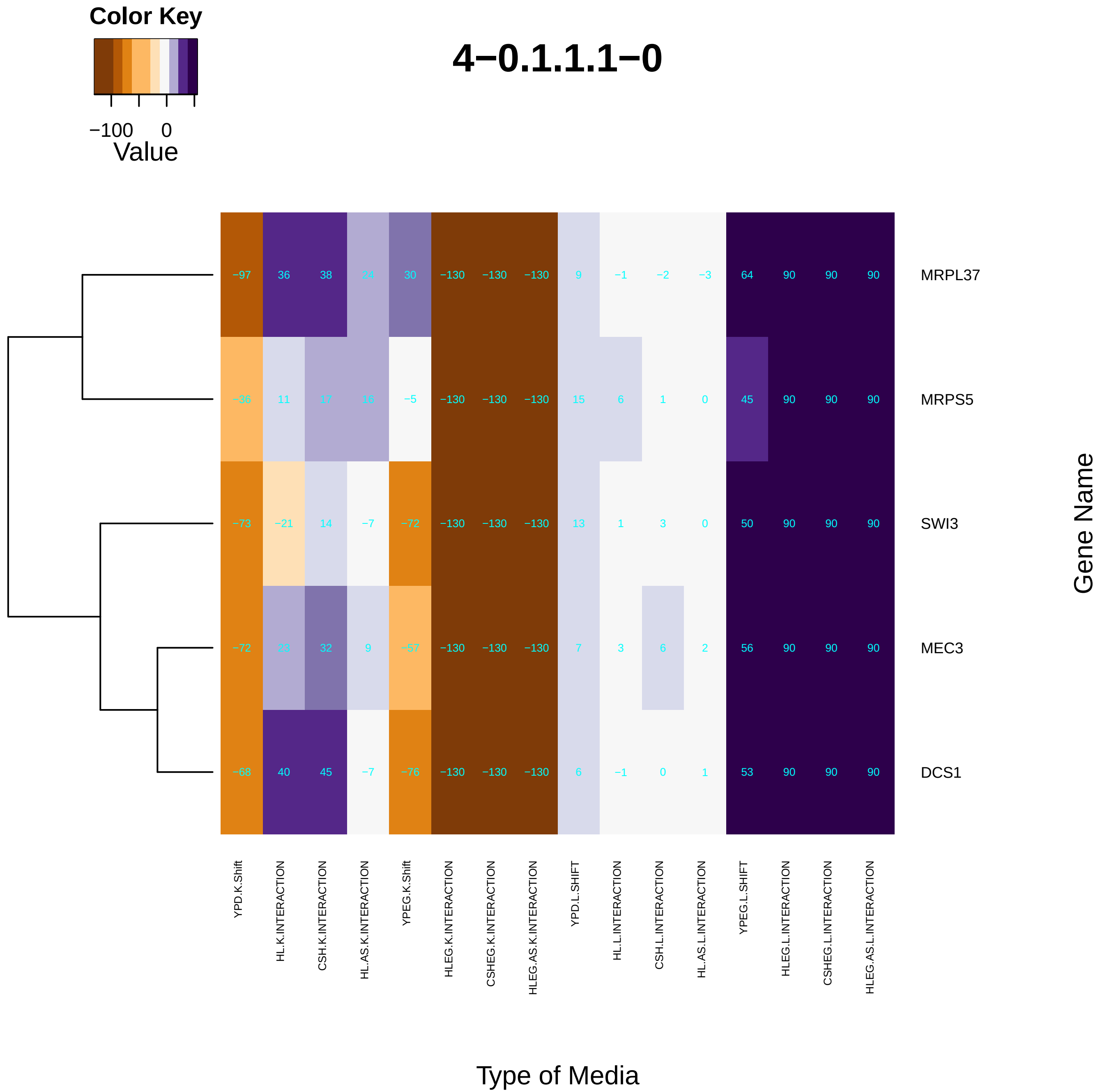

Color Key
−100
0
Value
4−0.1.1.1−0
MRPL37
MRPS5
SWI3
MEC3
DCS1
Gene Name
YPD.K.Shift
HL.K.INTERACTION
CSH.K.INTERACTION
HL.AS.K.INTERACTION
YPEG.K.Shift
HLEG.K.INTERACTION
CSHEG.K.INTERACTION
HLEG.AS.K.INTERACTION
YPD.L.SHIFT
HL.L.INTERACTION
CSH.L.INTERACTION
HL.AS.L.INTERACTION
YPEG.L.SHIFT
HLEG.L.INTERACTION
CSHEG.L.INTERACTION
HLEG.AS.L.INTERACTION
Type of Media

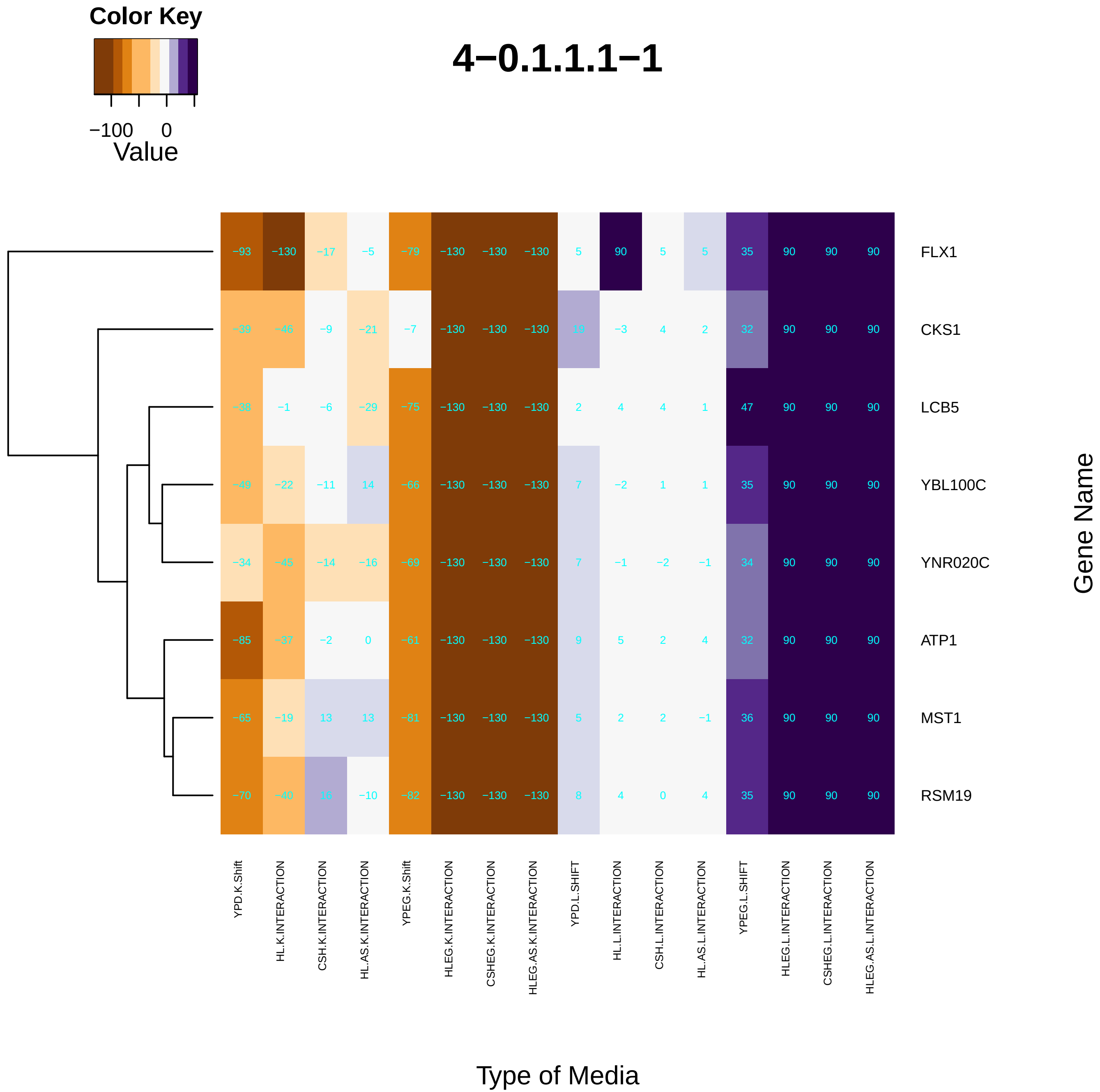

Color Key
−100
0
Value
4−0.1.1.1−1
FLX1
CKS1
LCB5
YBL100C
YNR020C
ATP1
MST1
RSM19
Gene Name
YPD.K.Shift
HL.K.INTERACTION
CSH.K.INTERACTION
HL.AS.K.INTERACTION
YPEG.K.Shift
HLEG.K.INTERACTION
CSHEG.K.INTERACTION
HLEG.AS.K.INTERACTION
YPD.L.SHIFT
HL.L.INTERACTION
CSH.L.INTERACTION
HL.AS.L.INTERACTION
YPEG.L.SHIFT
HLEG.L.INTERACTION
CSHEG.L.INTERACTION
HLEG.AS.L.INTERACTION
−93 −130 −17 −5 −79 −130 −130 −130 5 90 5 5 35 90 90 90
−39 −46 −9 −21 −7 −130 −130 −130 19 −3 4 2 32 90 90 90
−38 −1 −6 −29 −75 −130 −130 −130 2 4 4 1 47 90 90 90
−49 −22 −11 14 −66 −130 −130 −130 7 −2 1 1 35 90 90 90
−34 −45 −14 −16 −69 −130 −130 −130 7 −1 −2 −1 34 90 90 90
−85 −37 −2 0 −61 −130 −130 −130 9 5 2 4 32 90 90 90
−65 −19 13 13 −81 −130 −130 −130 5 2 2 −1 36 90 90 90
−70 −40 16 −10 −82 −130 −130 −130 8 4 0 4 35 90 90 90
Type of Media

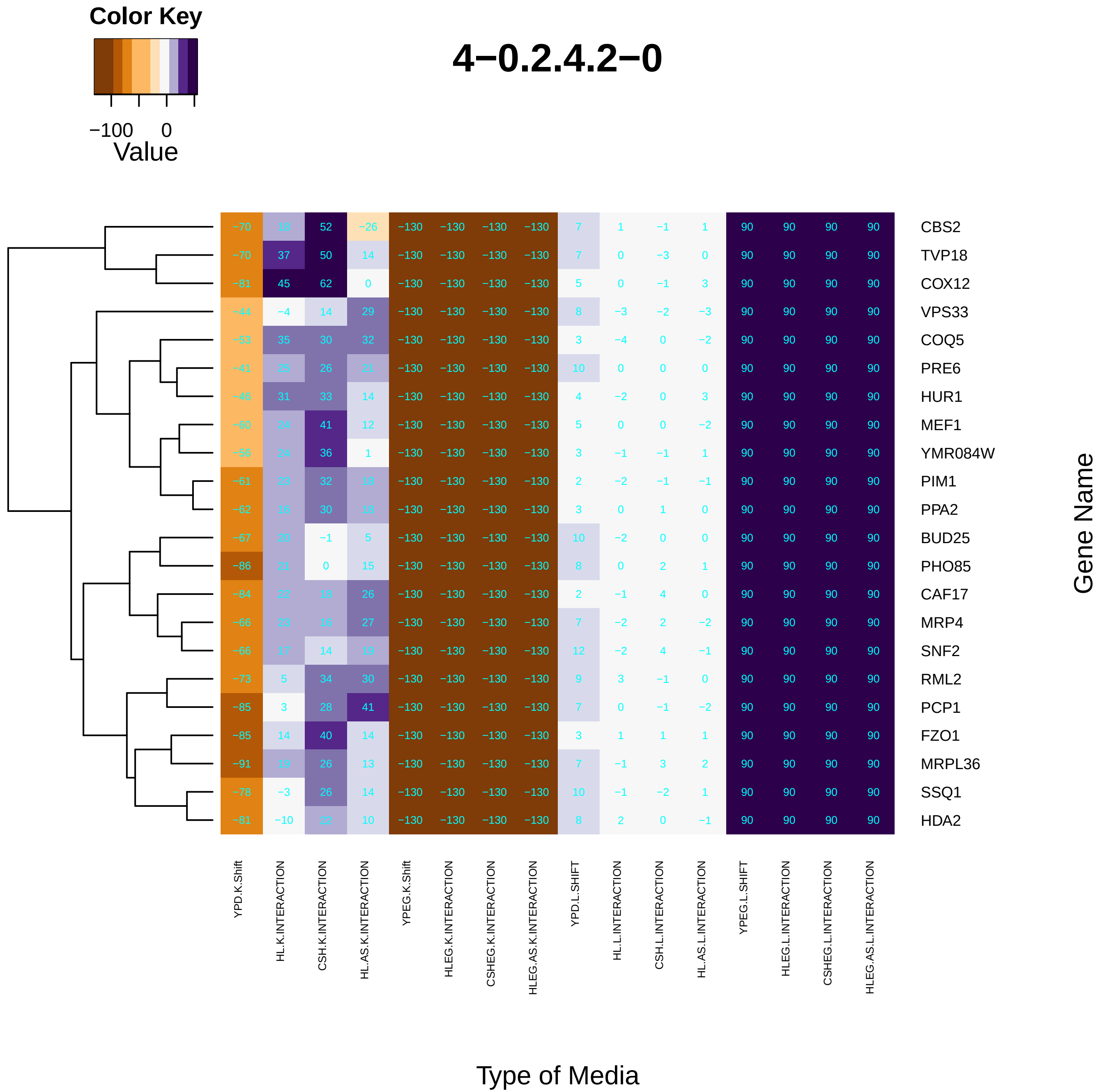
Color Key
−100
0
Value
4−0.2.4.2−0
CBS2
TVP18
COX12
VPS33
COQ5
PRE6
HUR1
MEF1
YMR084W
PIM1
PPA2
BUD25
PHO85
CAF17
MRP4
SNF2
RML2
PCP1
FZO1
MRPL36
SSQ1
HDA2
Gene Name
YPD.K.Shift
HL.K.INTERACTION
CSH.K.INTERACTION
HL.AS.K.INTERACTION
YPEG.K.Shift
HLEG.K.INTERACTION
CSHEG.K.INTERACTION
HLEG.AS.K.INTERACTION
YPD.L.SHIFT
HL.L.INTERACTION
CSH.L.INTERACTION
HL.AS.L.INTERACTION
YPEG.L.SHIFT
HLEG.L.INTERACTION
CSHEG.L.INTERACTION
HLEG.AS.L.INTERACTION
Type of Media

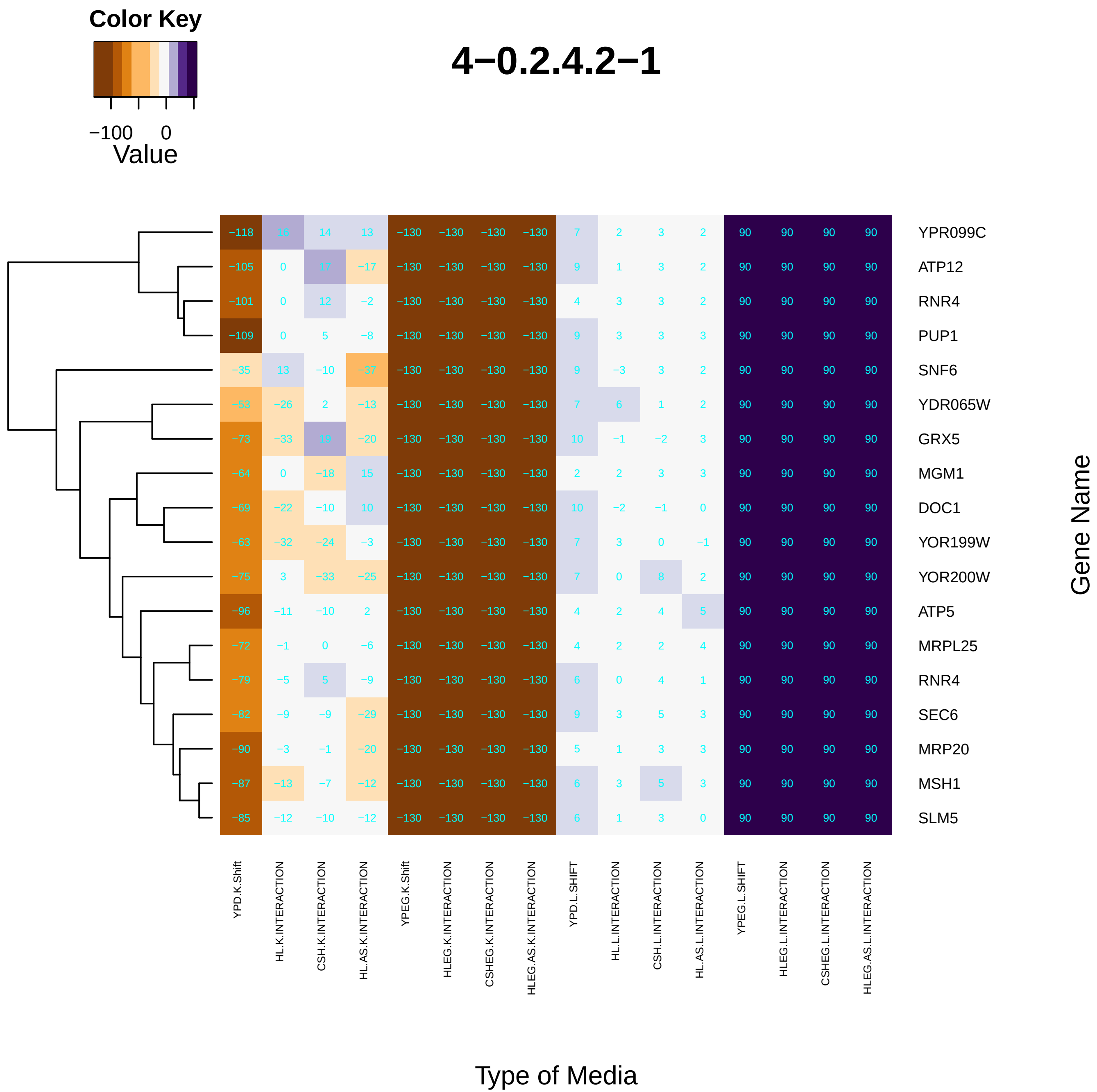
Color Key
−100
0
Value
4−0.2.4.2−1
−118 16 14 13 −130 −130 −130 −130 7 2 3 2 90 90 90 90
−105 0 17 −17 −130 −130 −130 −130 9 1 3 2 90 90 90 90
−101 0 12 −2 −130 −130 −130 −130 4 3 3 2 90 90 90 90
−109 0 5 −8 −130 −130 −130 −130 9 3 3 3 90 90 90 90
−35 13 −10 −37 −130 −130 −130 −130 9 −3 3 2 90 90 90 90
−53 −26 2 −13 −130 −130 −130 −130 7 6 1 2 90 90 90 90
−73 −33 19 −20 −130 −130 −130 −130 10 −1 −2 3 90 90 90 90
−64 0 −18 15 −130 −130 −130 −130 2 2 3 3 90 90 90 90
−69 −22 −10 10 −130 −130 −130 −130 10 −2 −1 0 90 90 90 90
−63 −32 −24 −3 −130 −130 −130 −130 7 3 0 −1 90 90 90 90
−75 3 −33 −25 −130 −130 −130 −130 7 0 8 2 90 90 90 90
−96 −11 −10 2 −130 −130 −130 −130 4 2 4 5 90 90 90 90
−72 −1 0 −6 −130 −130 −130 −130 4 2 2 4 90 90 90 90
−79 −5 5 −9 −130 −130 −130 −130 6 0 4 1 90 90 90 90
−82 −9 −9 −29 −130 −130 −130 −130 9 3 5 3 90 90 90 90
−90 −3 −1 −20 −130 −130 −130 −130 5 1 3 3 90 90 90 90
−87 −13 −7 −12 −130 −130 −130 −130 6 3 5 3 90 90 90 90
−85 −12 −10 −12 −130 −130 −130 −130 6 1 3 0 90 90 90 90
YPR099C
ATP12
RNR4
PUP1
SNF6
YDR065W
GRX5
MGM1
DOC1
YOR199W
YOR200W
ATP5
MRPL25
RNR4
SEC6
MRP20
MSH1
SLM5
Gene Name
YPD.K.Shift
HL.K.INTERACTION
CSH.K.INTERACTION
HL.AS.K.INTERACTION
YPEG.K.Shift
HLEG.K.INTERACTION
CSHEG.K.INTERACTION
HLEG.AS.K.INTERACTION
YPD.L.SHIFT
HL.L.INTERACTION
CSH.L.INTERACTION
HL.AS.L.INTERACTION
YPEG.L.SHIFT
HLEG.L.INTERACTION
CSHEG.L.INTERACTION
HLEG.AS.L.INTERACTION
Type of Media

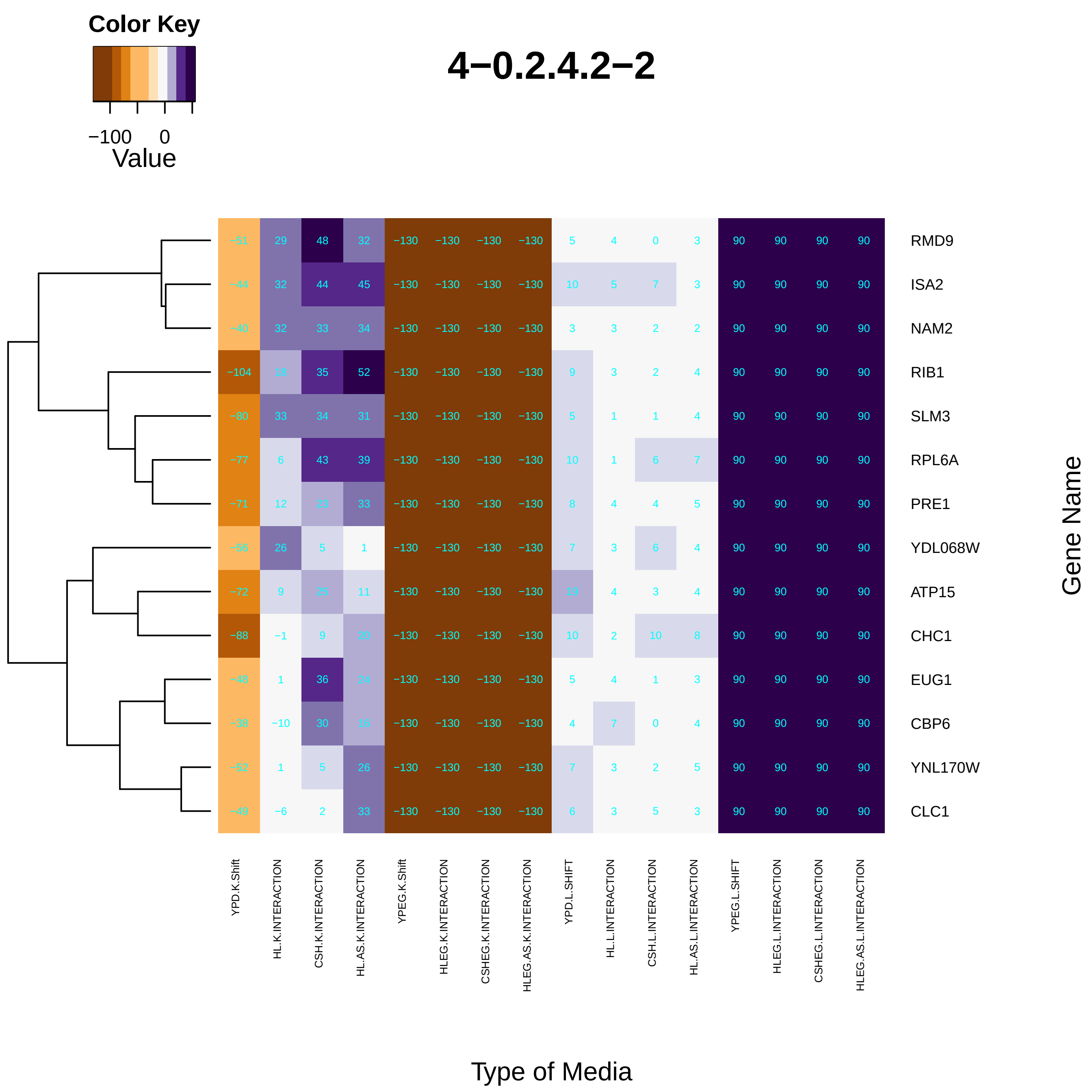
Color Key
−100
0
Value
4−0.2.4.2−2
RMD9
ISA2
NAM2
RIB1
SLM3
RPL6A
PRE1
YDL068W
ATP15
CHC1
EUG1
CBP6
YNL170W
CLC1
Gene Name
YPD.K.Shift
HL.K.INTERACTION
CSH.K.INTERACTION
HL.AS.K.INTERACTION
YPEG.K.Shift
HLEG.K.INTERACTION
CSHEG.K.INTERACTION
HLEG.AS.K.INTERACTION
YPD.L.SHIFT
HL.L.INTERACTION
CSH.L.INTERACTION
HL.AS.L.INTERACTION
YPEG.L.SHIFT
HLEG.L.INTERACTION
CSHEG.L.INTERACTION
HLEG.AS.L.INTERACTION
Type of Media

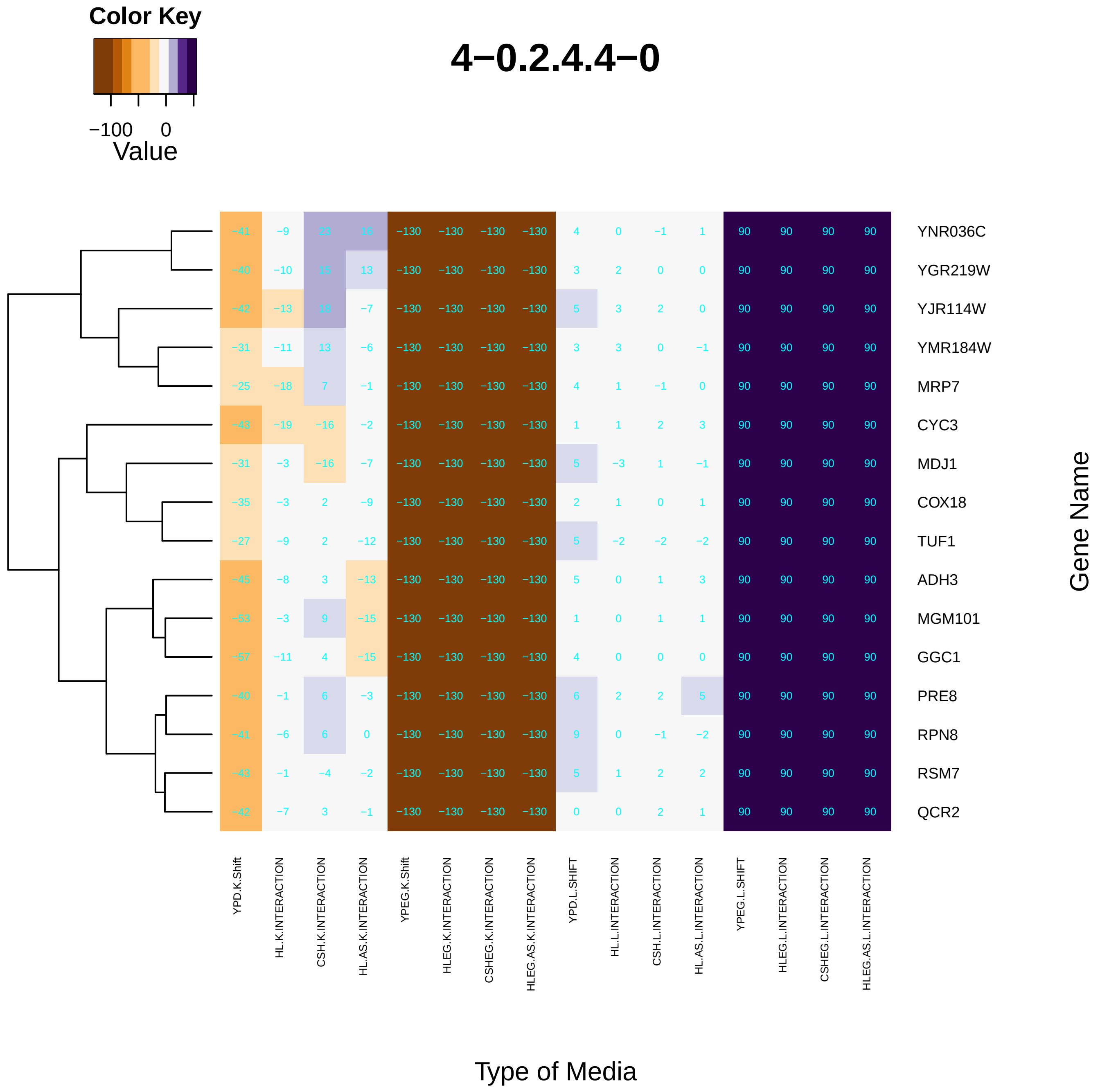
Color Key
−100
0
Value
4−0.2.4.4−0
YPD.K.Shift
HL.K.INTERACTION
CSH.K.INTERACTION
HL.AS.K.INTERACTION
YPEG.K.Shift
HLEG.K.INTERACTION
CSHEG.K.INTERACTION
HLEG.AS.K.INTERACTION
YPD.L.SHIFT
HL.L.INTERACTION
CSH.L.INTERACTION
HL.AS.L.INTERACTION
YPEG.L.SHIFT
HLEG.L.INTERACTION
CSHEG.L.INTERACTION
HLEG.AS.L.INTERACTION
YNR036C −41 −9 23 16 −130 −130 −130 −130 4 0 −1 1 90 90 90 90
YGR219W −40 −10 15 13 −130 −130 −130 −130 3 2 0 0 90 90 90 90
YJR114W −42 −13 18 −7 −130 −130 −130 −130 5 3 2 0 90 90 90 90
YMR184W −31 −11 13 −6 −130 −130 −130 −130 3 3 0 −1 90 90 90 90
MRP7 −25 −18 7 −1 −130 −130 −130 −130 4 1 −1 0 90 90 90 90
CYC3 −43 −19 −16 −2 −130 −130 −130 −130 1 1 2 3 90 90 90 90
MDJ1 −31 −3 −16 −7 −130 −130 −130 −130 5 −3 1 −1 90 90 90 90
COX18 −35 −3 2 −9 −130 −130 −130 −130 2 1 0 1 90 90 90 90
TUF1 −27 −9 2 −12 −130 −130 −130 −130 5 −2 −2 −2 90 90 90 90
ADH3 −45 −8 3 −13 −130 −130 −130 −130 5 0 1 3 90 90 90 90
MGM101 −53 −3 9 −15 −130 −130 −130 −130 1 0 1 1 90 90 90 90
GGC1 −57 −11 4 −15 −130 −130 −130 −130 4 0 0 0 90 90 90 90
PRE8 −40 −1 6 −3 −130 −130 −130 −130 6 2 2 5 90 90 90 90
RPN8 −41 −6 6 0 −130 −130 −130 −130 9 0 −1 −2 90 90 90 90
RSM7 −43 −1 −4 −2 −130 −130 −130 −130 5 1 2 2 90 90 90 90
QCR2 −42 −7 3 −1 −130 −130 −130 −130 0 0 2 1 90 90 90 90
Gene Name
Type of Media

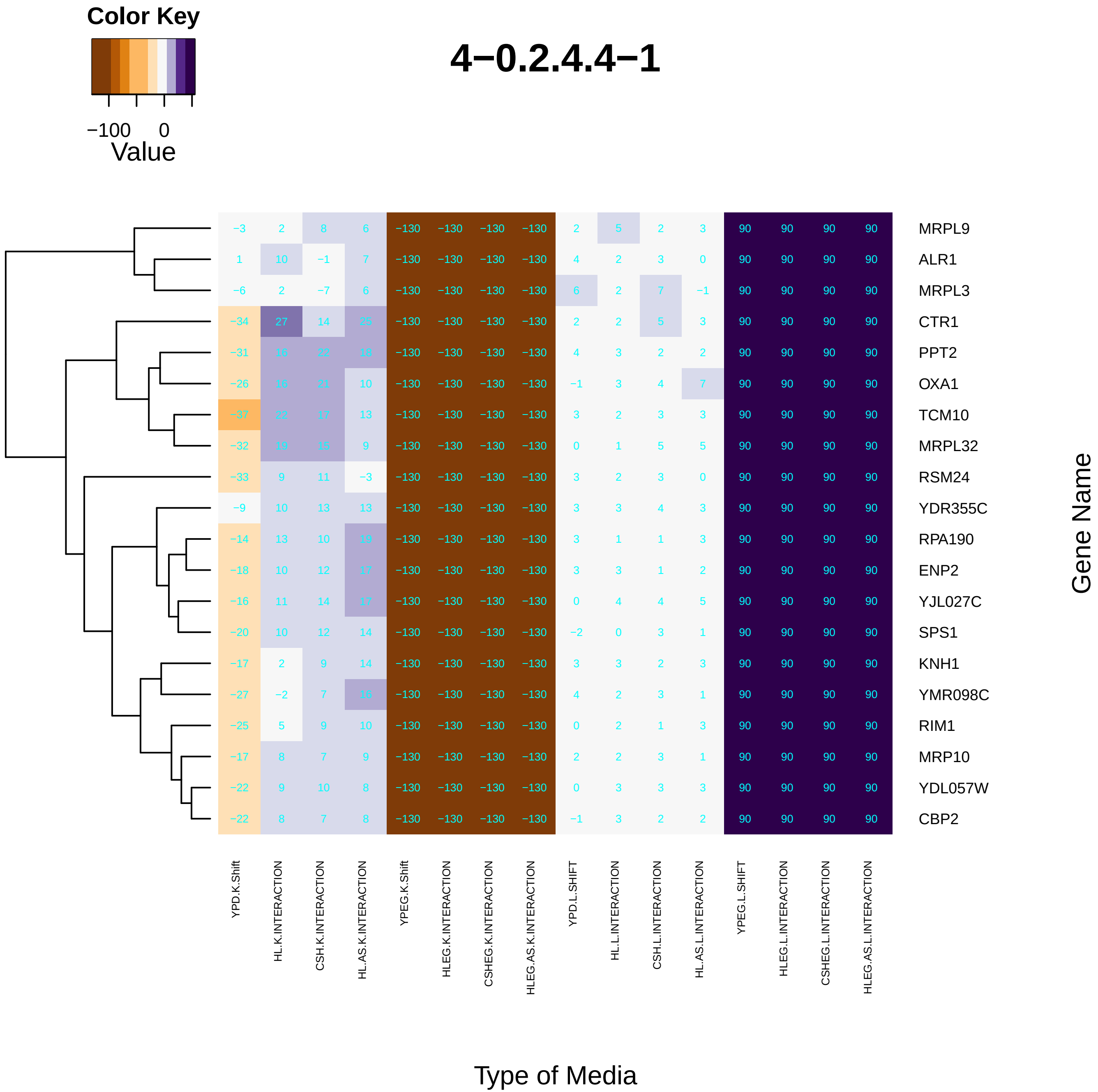
4−0.2.4.4−1
Color Key
−100
0
Value
MRPL9
ALR1
MRPL3
CTR1
PPT2
OXA1
TCM10
MRPL32
RSM24
YDR355C
RPA190
ENP2
YJL027C
SPS1
KNH1
YMR098C
RIM1
MRP10
YDL057W
CBP2
YPD.K.Shift
HL.K.INTERACTION
CSH.K.INTERACTION
HL.AS.K.INTERACTION
YPEG.K.Shift
HLEG.K.INTERACTION
CSHEG.K.INTERACTION
HLEG.AS.K.INTERACTION
YPD.L.SHIFT
HL.L.INTERACTION
CSH.L.INTERACTION
HL.AS.L.INTERACTION
YPEG.L.SHIFT
HLEG.L.INTERACTION
CSHEG.L.INTERACTION
HLEG.AS.L.INTERACTION
Gene Name
Type of Media

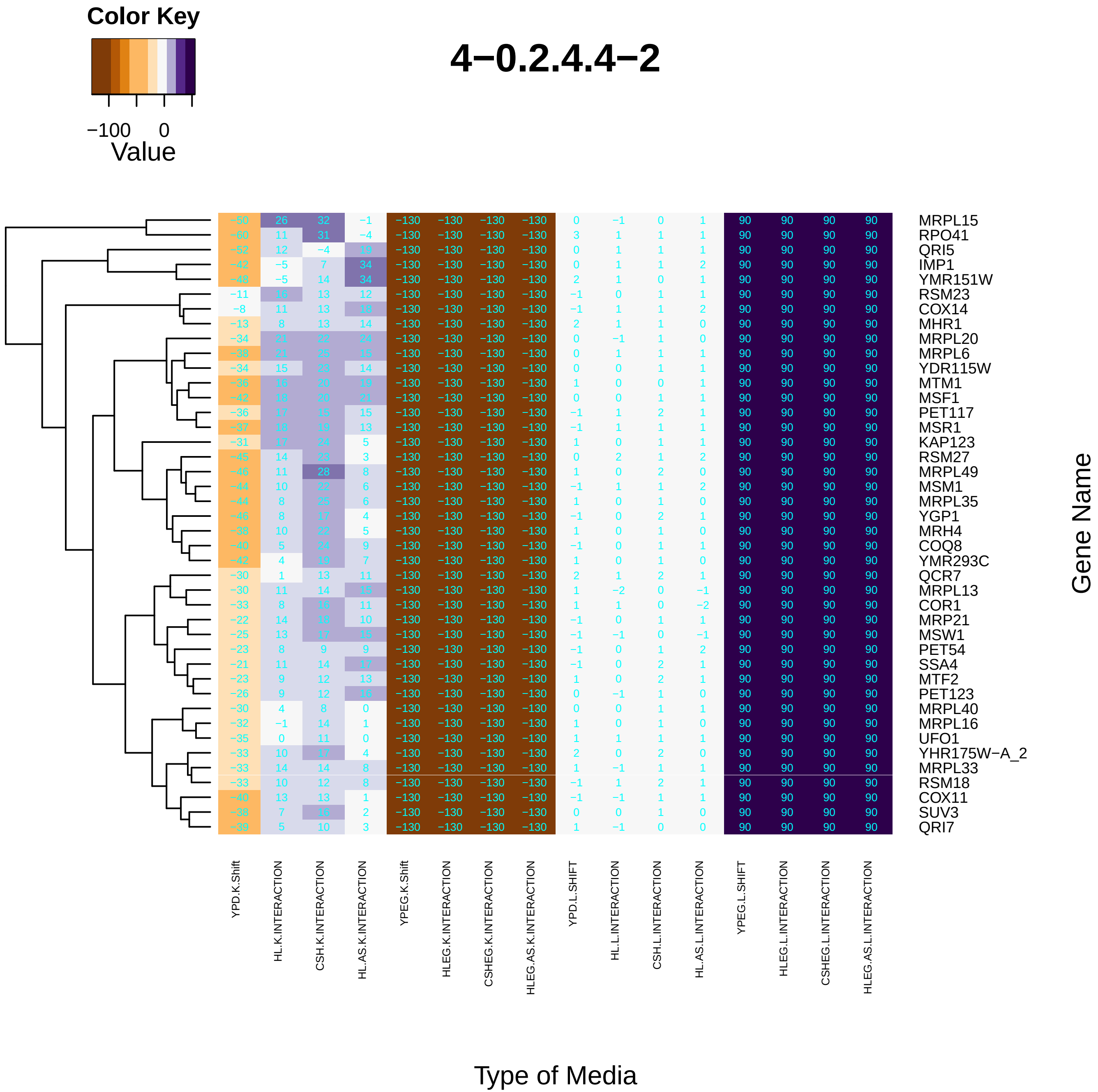
4−0.2.4.4−2
Color Key
−100
0
Value
YPD.K.Shift
HL.K.INTERACTION
CSH.K.INTERACTION
HL.AS.K.INTERACTION
YPEG.K.Shift
HLEG.K.INTERACTION
CSHEG.K.INTERACTION
HLEG.AS.K.INTERACTION
YPD.L.SHIFT
HL.L.INTERACTION
CSH.L.INTERACTION
HL.AS.L.INTERACTION
YPEG.L.SHIFT
HLEG.L.INTERACTION
CSHEG.L.INTERACTION
HLEG.AS.L.INTERACTION
MRPL15
RPO41
QRI5
IMP1
YMR151W
RSM23
COX14
MHR1
MRPL20
MRPL6
YDR115W
MTM1
MSF1
PET117
MSR1
KAP123
RSM27
MRPL49
MSM1
MRPL35
YGP1
MRH4
COQ8
YMR293C
QCR7
MRPL13
COR1
MRP21
MSW1
PET54
SSA4
MTF2
PET123
MRPL40
MRPL16
UFO1
YHR175W−A_2
MRPL33
RSM18
COX11
SUV3
QRI7
Gene Name
Type of Media

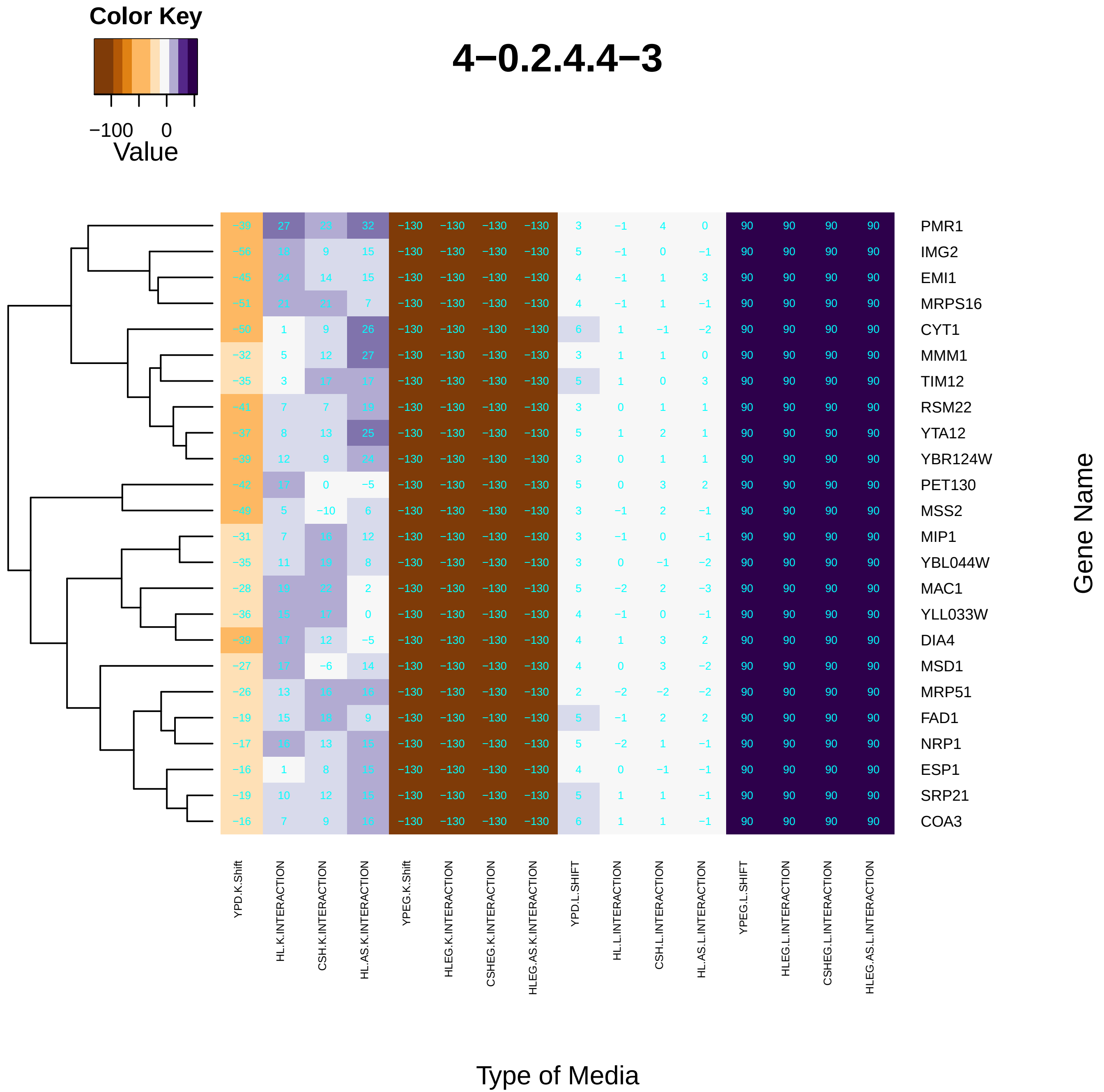
Color Key
−100
0
Value
4−0.2.4.4−3
PMR1
IMG2
EMI1
MRPS16
CYT1
MMM1
TIM12
RSM22
YTA12
YBR124W
PET130
MSS2
MIP1
YBL044W
MAC1
YLL033W
DIA4
MSD1
MRP51
FAD1
NRP1
ESP1
SRP21
COA3
Gene Name
YPD.K.Shift
HL.K.INTERACTION
CSH.K.INTERACTION
HL.AS.K.INTERACTION
YPEG.K.Shift
HLEG.K.INTERACTION
CSHEG.K.INTERACTION
HLEG.AS.K.INTERACTION
YPD.L.SHIFT
HL.L.INTERACTION
CSH.L.INTERACTION
HL.AS.L.INTERACTION
YPEG.L.SHIFT
HLEG.L.INTERACTION
CSHEG.L.INTERACTION
HLEG.AS.L.INTERACTION
Type of Media

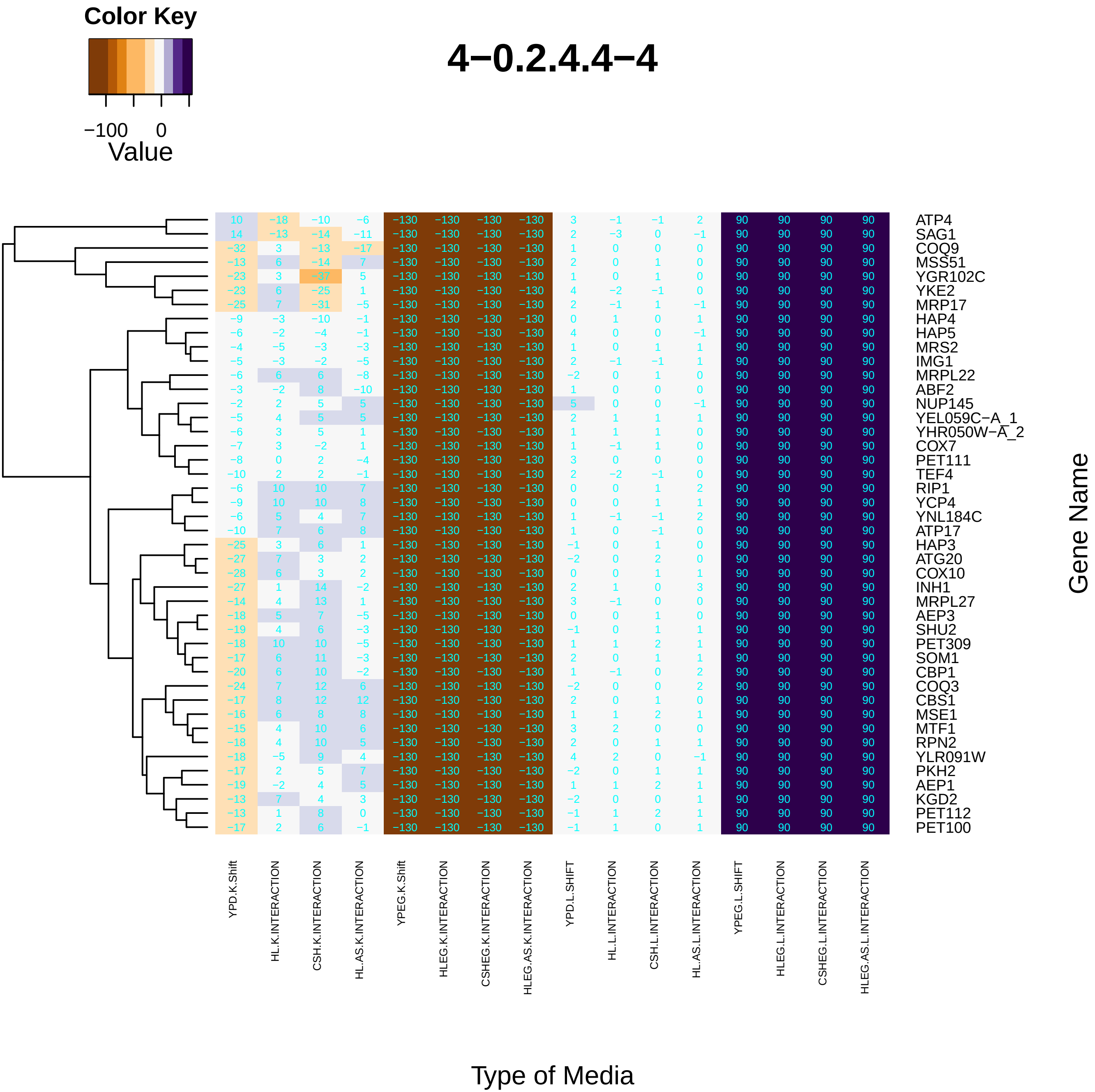
Color Key
−100
0
Value
4−0.2.4.4−4
ATP4
SAG1
COQ9
MSS51
YGR102C
YKE2
MRP17
HAP4
HAP5
MRS2
IMG1
MRPL22
ABF2
NUP145
YEL059C−A_1
YHR050W−A_2
COX7
PET111
TEF4
RIP1
YCP4
YNL184C
ATP17
HAP3
ATG20
COX10
INH1
MRPL27
AEP3
SHU2
PET309
SOM1
CBP1
COQ3
CBS1
MSE1
MTF1
RPN2
YLR091W
PKH2
AEP1
KGD2
PET112
PET100
Gene Name
YPD.K.Shift
HL.K.INTERACTION
CSH.K.INTERACTION
HL.AS.K.INTERACTION
YPEG.K.Shift
HLEG.K.INTERACTION
CSHEG.K.INTERACTION
HLEG.AS.K.INTERACTION
YPD.L.SHIFT
HL.L.INTERACTION
CSH.L.INTERACTION
HL.AS.L.INTERACTION
YPEG.L.SHIFT
HLEG.L.INTERACTION
CSHEG.L.INTERACTION
HLEG.AS.L.INTERACTION
Type of Media

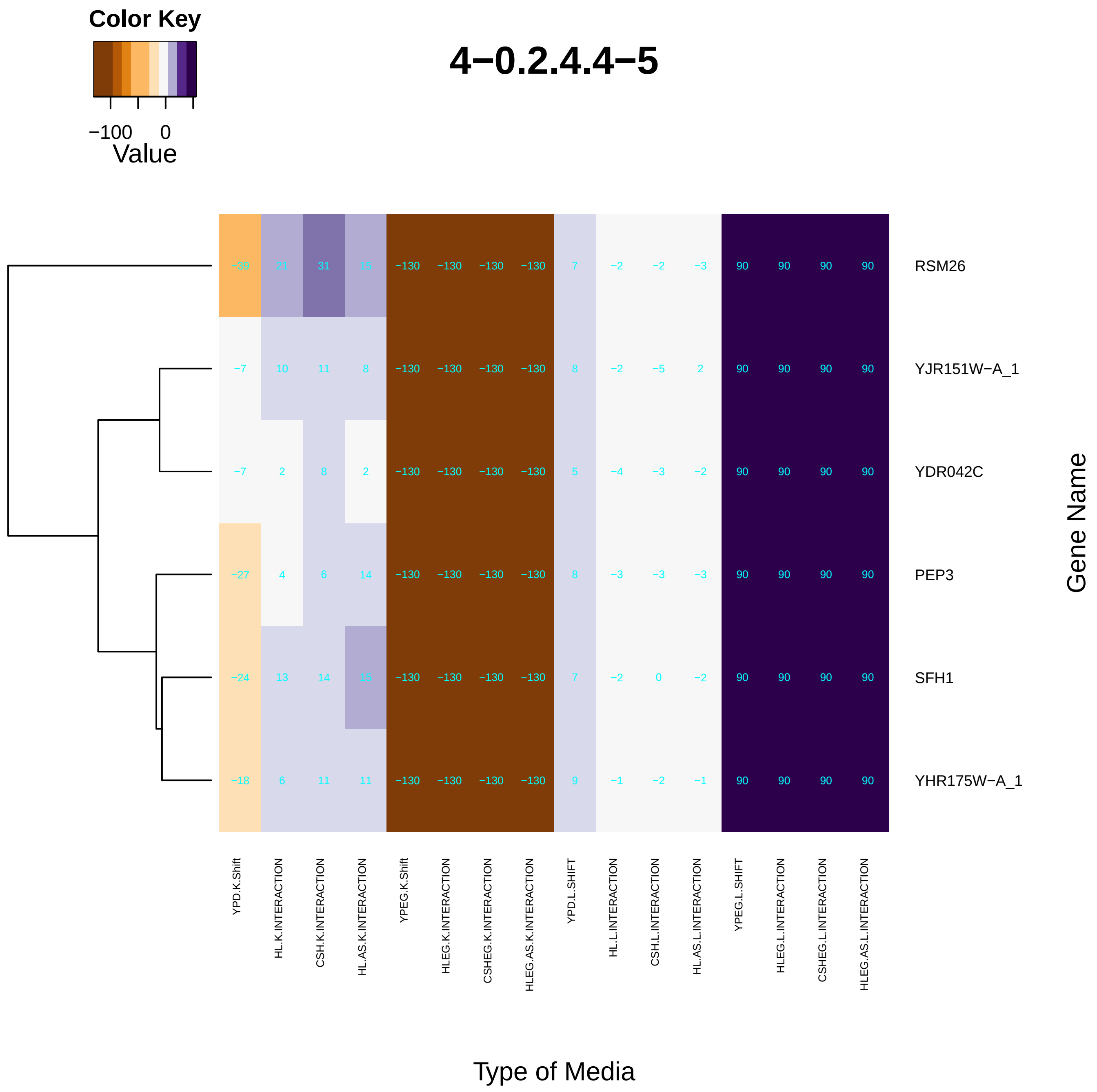
Color Key
−100
0
Value
4−0.2.4.4−5
RSM26
YJR151W−A_1
YDR042C
PEP3
SFH1
YHR175W−A_1
Gene Name
YPD.K.Shift
HL.K.INTERACTION
CSH.K.INTERACTION
HL.AS.K.INTERACTION
YPEG.K.Shift
HLEG.K.INTERACTION
CSHEG.K.INTERACTION
HLEG.AS.K.INTERACTION
YPD.L.SHIFT
HL.L.INTERACTION
CSH.L.INTERACTION
HL.AS.L.INTERACTION
YPEG.L.SHIFT
HLEG.L.INTERACTION
CSHEG.L.INTERACTION
HLEG.AS.L.INTERACTION
Type of Media
−39 21 31 15 −130 −130 −130 −130 7 −2 −2 −3 90 90 90 90
−7 10 11 8 −130 −130 −130 −130 8 −2 −5 2 90 90 90 90
−7 2 8 2 −130 −130 −130 −130 5 −4 −3 −2 90 90 90 90
−27 4 6 14 −130 −130 −130 −130 8 −3 −3 −3 90 90 90 90
−24 13 14 15 −130 −130 −130 −130 7 −2 0 −2 90 90 90 90
−18 6 11 11 −130 −130 −130 −130 9 −1 −2 −1 90 90 90 90

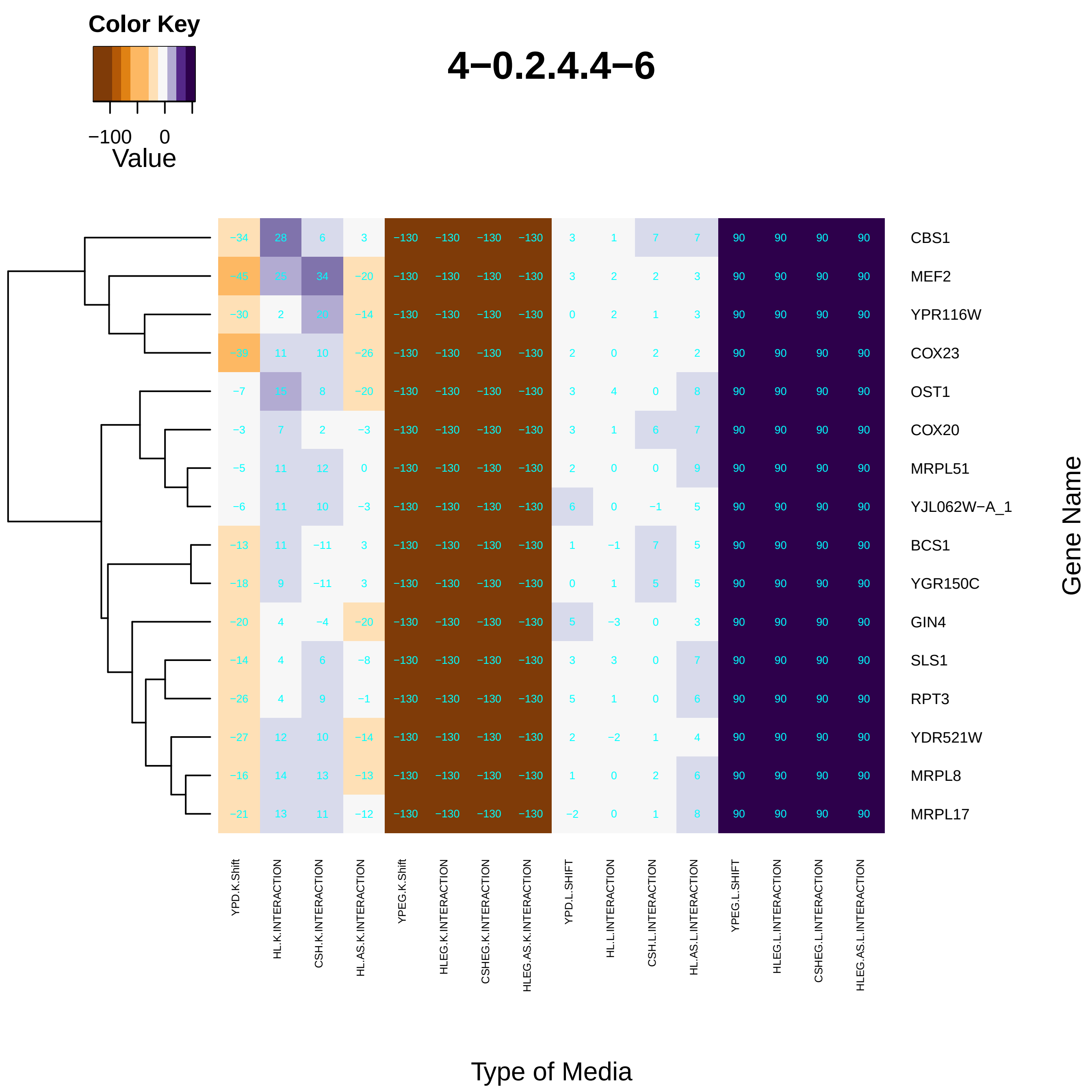
Color Key
−100
0
Value
4−0.2.4.4−6
YPD.K.Shift
HL.K.INTERACTION
CSH.K.INTERACTION
HL.AS.K.INTERACTION
YPEG.K.Shift
HLEG.K.INTERACTION
CSHEG.K.INTERACTION
HLEG.AS.K.INTERACTION
YPD.L.SHIFT
HL.L.INTERACTION
CSH.L.INTERACTION
HL.AS.L.INTERACTION
YPEG.L.SHIFT
HLEG.L.INTERACTION
CSHEG.L.INTERACTION
HLEG.AS.L.INTERACTION
CBS1 −34 28 6 3 −130 −130 −130 −130 3 1 7 7 90 90 90 90
MEF2 −45 25 34 −20 −130 −130 −130 −130 3 2 2 3 90 90 90 90
YPR116W −30 2 20 −14 −130 −130 −130 −130 0 2 1 3 90 90 90 90
COX23 −39 11 10 −26 −130 −130 −130 −130 2 0 2 2 90 90 90 90
OST1 −7 15 8 −20 −130 −130 −130 −130 3 4 0 8 90 90 90 90
COX20 −3 7 2 −3 −130 −130 −130 −130 3 1 6 7 90 90 90 90
MRPL51 −5 11 12 0 −130 −130 −130 −130 2 0 0 9 90 90 90 90
YJL062W−A_1 −6 11 10 −3 −130 −130 −130 −130 6 0 −1 5 90 90 90 90
BCS1 −13 11 −11 3 −130 −130 −130 −130 1 −1 7 5 90 90 90 90
YGR150C −18 9 −11 3 −130 −130 −130 −130 0 1 5 5 90 90 90 90
GIN4 −20 4 −4 −20 −130 −130 −130 −130 5 −3 0 3 90 90 90 90
SLS1 −14 4 6 −8 −130 −130 −130 −130 3 3 0 7 90 90 90 90
RPT3 −26 4 9 −1 −130 −130 −130 −130 5 1 0 6 90 90 90 90
YDR521W −27 12 10 −14 −130 −130 −130 −130 2 −2 1 4 90 90 90 90
MRPL8 −16 14 13 −13 −130 −130 −130 −130 1 0 2 6 90 90 90 90
MRPL17 −21 13 11 −12 −130 −130 −130 −130 −2 0 1 8 90 90 90 90
Gene Name
Type of Media

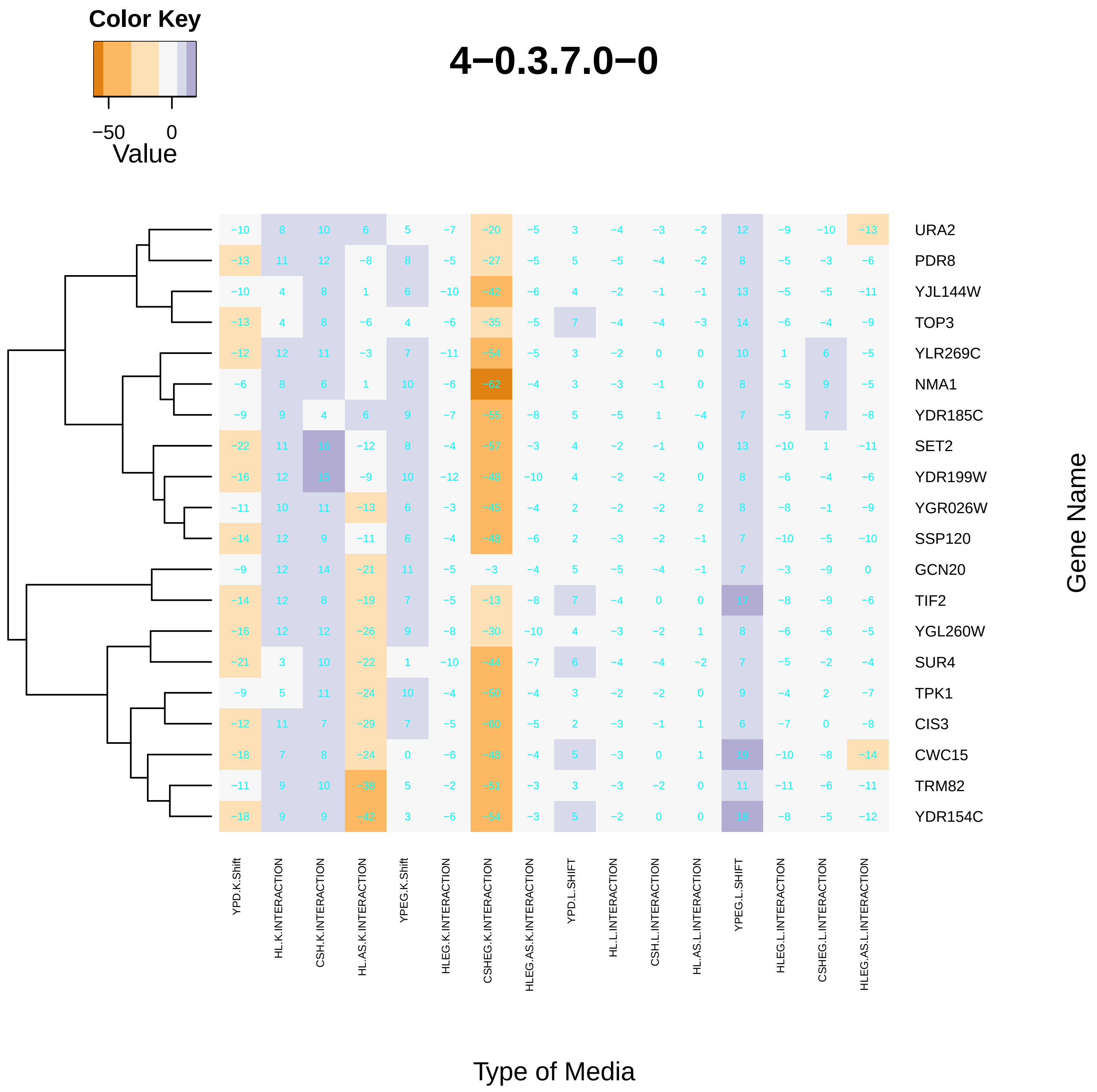
Color Key
−50
0
Value
4−0.3.7.0−0
URA2
PDR8
YJL144W
TOP3
YLR269C
NMA1
YDR185C
SET2
YDR199W
YGR026W
SSP120
GCN20
TIF2
YGL260W
SUR4
TPK1
CIS3
CWC15
TRM82
YDR154C
Gene Name
YPD.K.Shift
HL.K.INTERACTION
CSH.K.INTERACTION
HL.AS.K.INTERACTION
YPEG.K.Shift
HLEG.K.INTERACTION
CSHEG.K.INTERACTION
HLEG.AS.K.INTERACTION
YPD.L.SHIFT
HL.L.INTERACTION
CSH.L.INTERACTION
HL.AS.L.INTERACTION
YPEG.L.SHIFT
HLEG.L.INTERACTION
CSHEG.L.INTERACTION
HLEG.AS.L.INTERACTION
Type of Media

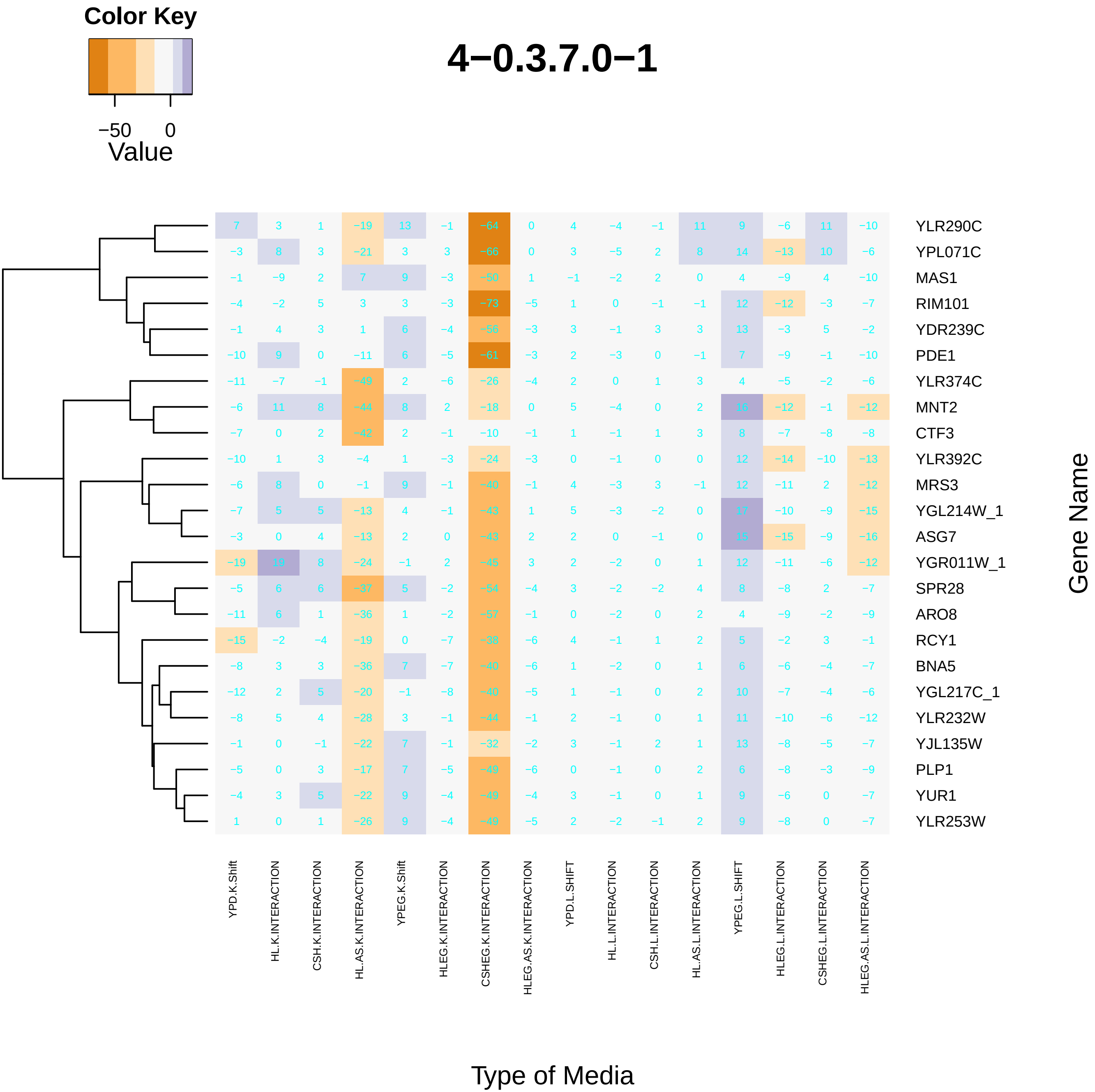
Color Key
−50
0
Value
4−0.3.7.0−1
YLR290C
YPL071C
MAS1
RIM101
YDR239C
PDE1
YLR374C
MNT2
CTF3
YLR392C
MRS3
YGL214W_1
ASG7
YGR011W_1
SPR28
ARO8
RCY1
BNA5
YGL217C_1
YLR232W
YJL135W
PLP1
YUR1
YLR253W
Gene Name
YPD.K.Shift
HL.K.INTERACTION
CSH.K.INTERACTION
HL.AS.K.INTERACTION
YPEG.K.Shift
HLEG.K.INTERACTION
CSHEG.K.INTERACTION
HLEG.AS.K.INTERACTION
YPD.L.SHIFT
HL.L.INTERACTION
CSH.L.INTERACTION
HL.AS.L.INTERACTION
YPEG.L.SHIFT
HLEG.L.INTERACTION
CSHEG.L.INTERACTION
HLEG.AS.L.INTERACTION
Type of Media

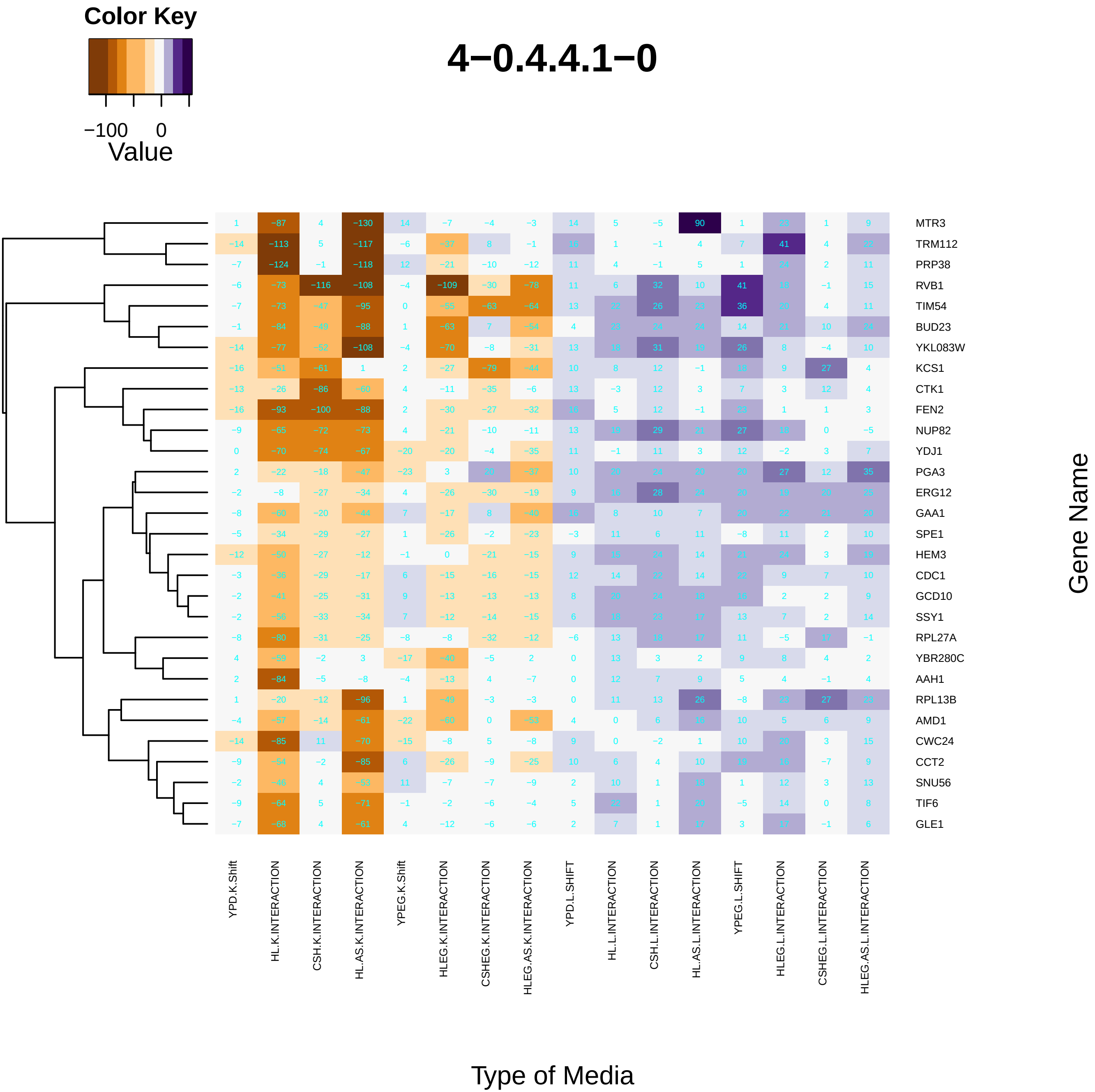
4−0.4.4.1−0
Color Key
−100
0
Value
MTR3
TRM112
PRP38
RVB1
TIM54
BUD23
YKL083W
KCS1
CTK1
FEN2
NUP82
YDJ1
PGA3
ERG12
GAA1
SPE1
HEM3
CDC1
GCD10
SSY1
RPL27A
YBR280C
AAH1
RPL13B
AMD1
CWC24
CCT2
SNU56
TIF6
GLE1
Gene Name
YPD.K.Shift
HL.K.INTERACTION
CSH.K.INTERACTION
HL.AS.K.INTERACTION
YPEG.K.Shift
HLEG.K.INTERACTION
CSHEG.K.INTERACTION
HLEG.AS.K.INTERACTION
YPD.L.SHIFT
HL.L.INTERACTION
CSH.L.INTERACTION
HL.AS.L.INTERACTION
YPEG.L.SHIFT
HLEG.L.INTERACTION
CSHEG.L.INTERACTION
HLEG.AS.L.INTERACTION
Type of Media

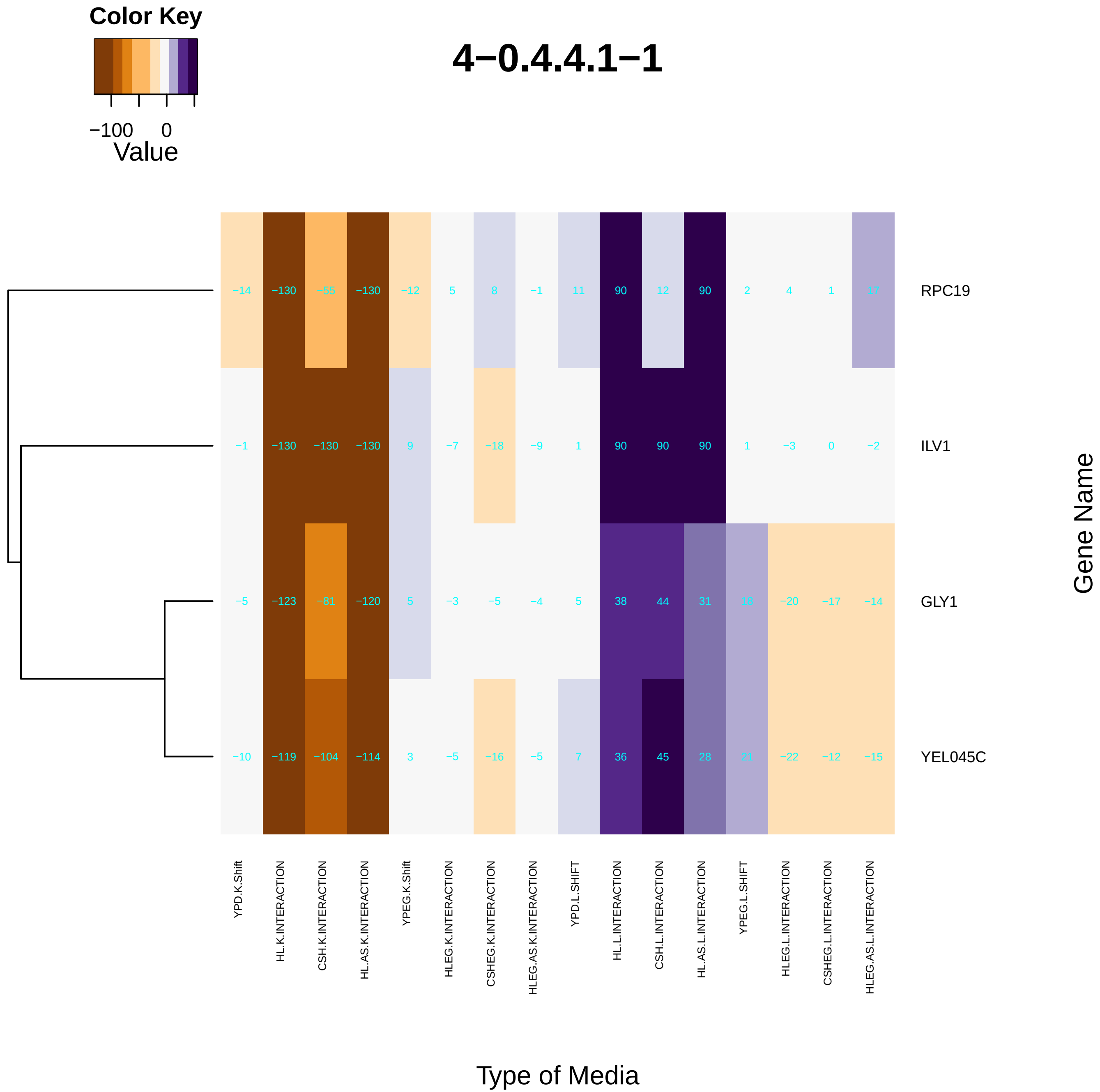

Color Key
−100
0
Value
4−0.4.4.1−1
−14 −130 −55 −130 −12 5 8 −1 11 90 12 90 2 4 1 17
−1 −130 −130 −130 9 −7 −18 −9 1 90 90 90 1 −3 0 −2
−5 −123 −81 −120 5 −3 −5 −4 5 38 44 31 18 −20 −17 −14
−10 −119 −104 −114 3 −5 −16 −5 7 36 45 28 21 −22 −12 −15
RPC19
ILV1
GLY1
YEL045C
Gene Name
YPD.K.Shift
HL.K.INTERACTION
CSH.K.INTERACTION
HL.AS.K.INTERACTION
YPEG.K.Shift
HLEG.K.INTERACTION
CSHEG.K.INTERACTION
HLEG.AS.K.INTERACTION
YPD.L.SHIFT
HL.L.INTERACTION
CSH.L.INTERACTION
HL.AS.L.INTERACTION
YPEG.L.SHIFT
HLEG.L.INTERACTION
CSHEG.L.INTERACTION
HLEG.AS.L.INTERACTION
Type of Media

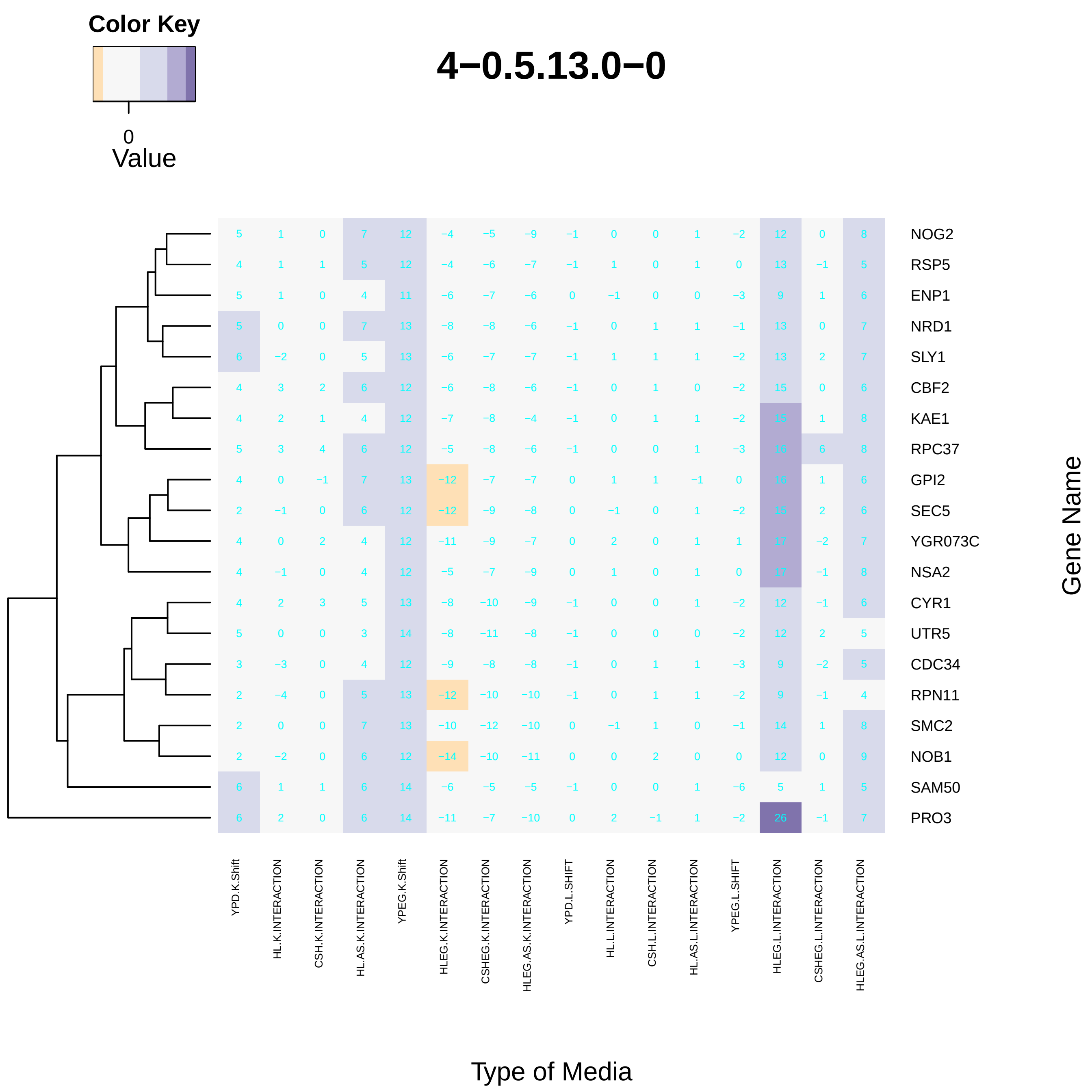
Color Key
0
Value
4−0.5.13.0−0
Gene Name
Type of Media
YPD.K.Shift
HL.K.INTERACTION
CSH.K.INTERACTION
HL.AS.K.INTERACTION
YPEG.K.Shift
HLEG.K.INTERACTION
CSHEG.K.INTERACTION
HLEG.AS.K.INTERACTION
YPD.L.SHIFT
HL.L.INTERACTION
CSH.L.INTERACTION
HL.AS.L.INTERACTION
YPEG.L.SHIFT
HLEG.L.INTERACTION
CSHEG.L.INTERACTION
HLEG.AS.L.INTERACTION
NOG2 5 1 0 7 12 −4 −5 −9 −1 0 0 1 −2 12 0 8
RSP5 4 1 1 5 12 −4 −6 −7 −1 1 0 1 0 13 −1 5
ENP1 5 1 0 4 11 −6 −7 −6 0 −1 0 0 −3 9 1 6
NRD1 5 0 0 7 13 −8 −8 −6 −1 0 1 1 −1 13 0 7
SLY1 6 −2 0 5 13 −6 −7 −7 −1 1 1 1 −2 13 2 7
CBF2 4 3 2 6 12 −6 −8 −6 −1 0 1 0 −2 15 0 6
KAE1 4 2 1 4 12 −7 −8 −4 −1 0 1 1 −2 15 1 8
RPC37 5 3 4 6 12 −5 −8 −6 −1 0 0 1 −3 16 6 8
GPI2 4 0 −1 7 13 −12 −7 −7 0 1 1 −1 0 16 1 6
SEC5 2 −1 0 6 12 −12 −9 −8 0 −1 0 1 −2 15 2 6
YGR073C 4 0 2 4 12 −11 −9 −7 0 2 0 1 1 17 −2 7
NSA2 4 −1 0 4 12 −5 −7 −9 0 1 0 1 0 17 −1 8
CYR1 4 2 3 5 13 −8 −10 −9 −1 0 0 1 −2 12 −1 6
UTR5 5 0 0 3 14 −8 −11 −8 −1 0 0 0 −2 12 2 5
CDC34 3 −3 0 4 12 −9 −8 −8 −1 0 1 1 −3 9 −2 5
RPN11 2 −4 0 5 13 −12 −10 −10 −1 0 1 1 −2 9 −1 4
SMC2 2 0 0 7 13 −10 −12 −10 0 −1 1 0 −1 14 1 8
NOB1 2 −2 0 6 12 −14 −10 −11 0 0 2 0 0 12 0 9
SAM50 6 1 1 6 14 −6 −5 −5 −1 0 0 1 −6 5 1 5
PRO3 6 2 0 6 14 −11 −7 −10 0 2 −1 1 −2 26 −1 7

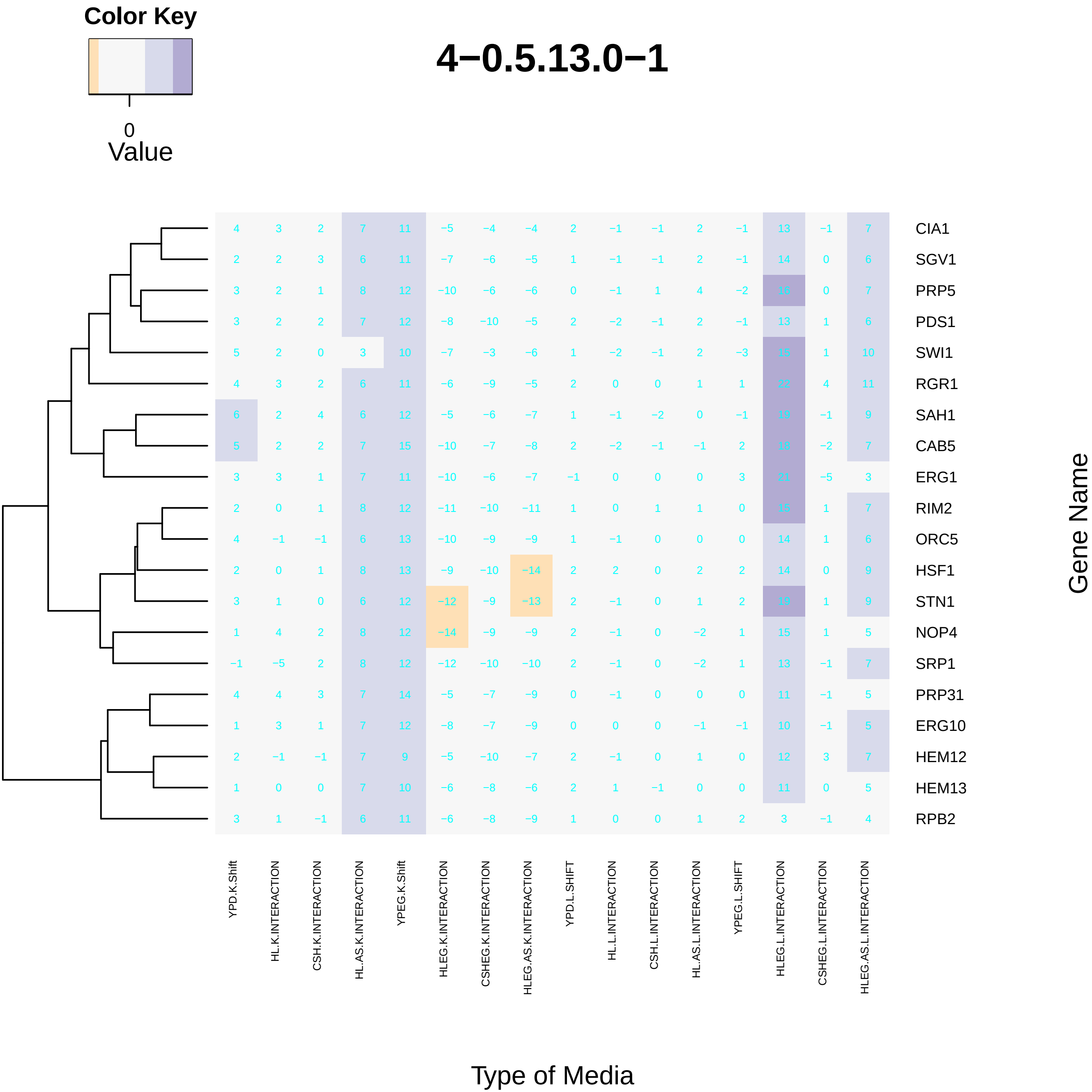
Color Key
0
Value
4−0.5.13.0−1
CIA1 4 3 2 7 11 −5 −4 −4 2 −1 −1 2 −1 13 −1 7
SGV1 2 2 3 6 11 −7 −6 −5 1 −1 −1 2 −1 14 0 6
PRP5 3 2 1 8 12 −10 −6 −6 0 −1 1 4 −2 16 0 7
PDS1 3 2 2 7 12 −8 −10 −5 2 −2 −1 2 −1 13 1 6
SWI1 5 2 0 3 10 −7 −3 −6 1 −2 −1 2 −3 15 1 10
RGR1 4 3 2 6 11 −6 −9 −5 2 0 0 1 1 22 4 11
SAH1 6 2 4 6 12 −5 −6 −7 1 −1 −2 0 −1 19 −1 9
CAB5 5 2 2 7 15 −10 −7 −8 2 −2 −1 −1 2 18 −2 7
ERG1 3 3 1 7 11 −10 −6 −7 −1 0 0 0 3 21 −5 3
RIM2 2 0 1 8 12 −11 −10 −11 1 0 1 1 0 15 1 7
ORC5 4 −1 −1 6 13 −10 −9 −9 1 −1 0 0 0 14 1 6
HSF1 2 0 1 8 13 −9 −10 −14 2 2 0 2 2 14 0 9
STN1 3 1 0 6 12 −12 −9 −13 2 −1 0 1 2 19 1 9
NOP4 1 4 2 8 12 −14 −9 −9 2 −1 0 −2 1 15 1 5
SRP1 −1 −5 2 8 12 −12 −10 −10 2 −1 0 −2 1 13 −1 7
PRP31 4 4 3 7 14 −5 −7 −9 0 −1 0 0 0 11 −1 5
ERG10 1 3 1 7 12 −8 −7 −9 0 0 0 −1 −1 10 −1 5
HEM12 2 −1 −1 7 9 −5 −10 −7 2 −1 0 1 0 12 3 7
HEM13 1 0 0 7 10 −6 −8 −6 2 1 −1 0 0 11 0 5
RPB2 3 1 −1 6 11 −6 −8 −9 1 0 0 1 2 3 −1 4
YPD.K.Shift
HL.K.INTERACTION
CSH.K.INTERACTION
HL.AS.K.INTERACTION
YPEG.K.Shift
HLEG.K.INTERACTION
CSHEG.K.INTERACTION
HLEG.AS.K.INTERACTION
YPD.L.SHIFT
HL.L.INTERACTION
CSH.L.INTERACTION
HL.AS.L.INTERACTION
YPEG.L.SHIFT
HLEG.L.INTERACTION
CSHEG.L.INTERACTION
HLEG.AS.L.INTERACTION
Gene Name
Type of Media

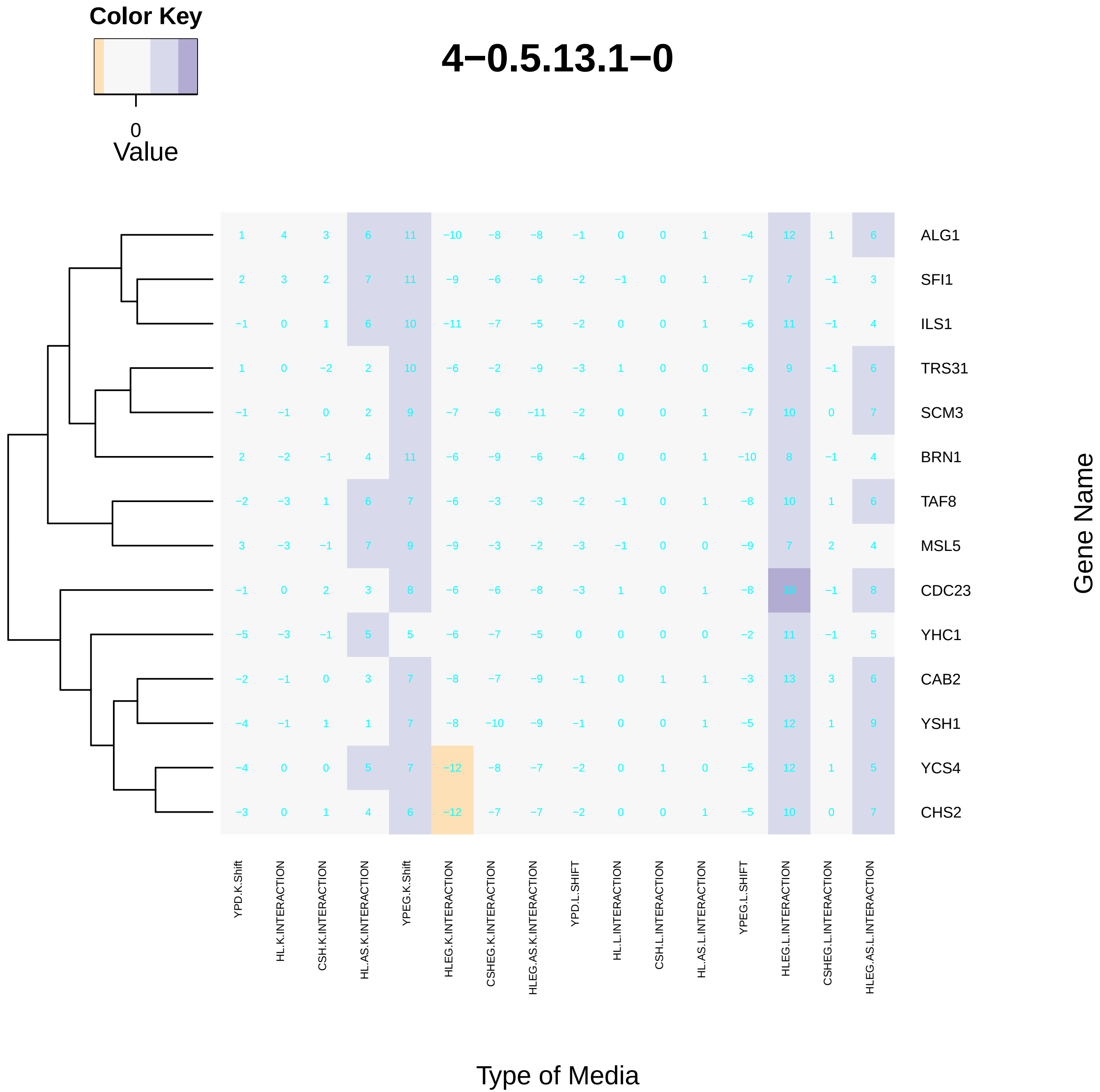
Color Key
0
Value
4−0.5.13.1−0
ALG1: 1 4 3 6 11 −10 −8 −8 −1 0 0 1 −4 12 1 6
SFI1: 2 3 2 7 11 −9 −6 −6 −2 −1 0 1 −7 7 −1 3
ILS1: −1 0 1 6 10 −11 −7 −5 −2 0 0 1 −6 11 −1 4
TRS31: 1 0 −2 2 10 −6 −2 −9 −3 1 0 0 −6 9 −1 6
SCM3: −1 −1 0 2 9 −7 −6 −11 −2 0 0 1 −7 10 0 7
BRN1: 2 −2 −1 4 11 −6 −9 −6 −4 0 0 1 −10 8 −1 4
TAF8: −2 −3 1 6 7 −6 −3 −3 −2 −1 0 1 −8 10 1 6
MSL5: 3 −3 −1 7 9 −9 −3 −2 −3 −1 0 0 −9 7 2 4
CDC23: −1 0 2 3 8 −6 −6 −8 −3 1 0 1 −8 18 −1 8
YHC1: −5 −3 −1 5 5 −6 −7 −5 0 0 0 0 −2 11 −1 5
CAB2: −2 −1 0 3 7 −8 −7 −9 −1 0 1 1 −3 13 3 6
YSH1: −4 −1 1 1 7 −8 −10 −9 −1 0 0 1 −5 12 1 9
YCS4: −4 0 0 5 7 −12 −8 −7 −2 0 1 0 −5 12 1 5
CHS2: −3 0 1 4 6 −12 −7 −7 −2 0 0 1 −5 10 0 7
YPD.K.Shift
HL.K.INTERACTION
CSH.K.INTERACTION
HL.AS.K.INTERACTION
YPEG.K.Shift
HLEG.K.INTERACTION
CSHEG.K.INTERACTION
HLEG.AS.K.INTERACTION
YPD.L.SHIFT
HL.L.INTERACTION
CSH.L.INTERACTION
HL.AS.L.INTERACTION
YPEG.L.SHIFT
HLEG.L.INTERACTION
CSHEG.L.INTERACTION
HLEG.AS.L.INTERACTION
Gene Name
Type of Media

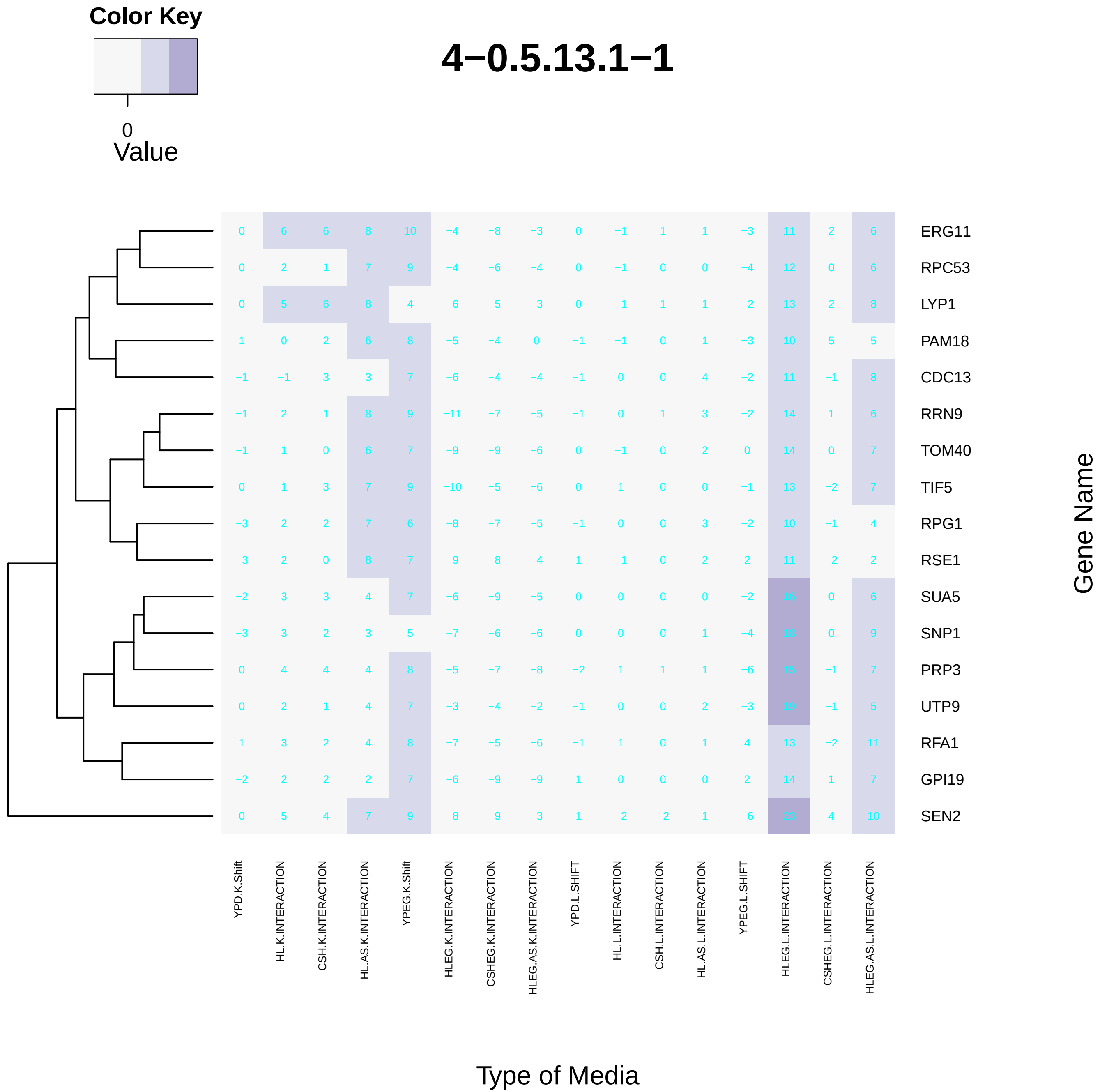

Color Key
0
Value
4−0.5.13.1−1
YPD.K.Shift
HL.K.INTERACTION
CSH.K.INTERACTION
HL.AS.K.INTERACTION
YPEG.K.Shift
HLEG.K.INTERACTION
CSHEG.K.INTERACTION
HLEG.AS.K.INTERACTION
YPD.L.SHIFT
HL.L.INTERACTION
CSH.L.INTERACTION
HL.AS.L.INTERACTION
YPEG.L.SHIFT
HLEG.L.INTERACTION
CSHEG.L.INTERACTION
HLEG.AS.L.INTERACTION
ERG11 0 6 6 8 10 −4 −8 −3 0 −1 1 1 −3 11 2 6
RPC53 0 2 1 7 9 −4 −6 −4 0 −1 0 0 −4 12 0 6
LYP1 0 5 6 8 4 −6 −5 −3 0 −1 1 1 −2 13 2 8
PAM18 1 0 2 6 8 −5 −4 0 −1 −1 0 1 −3 10 5 5
CDC13 −1 −1 3 3 7 −6 −4 −4 −1 0 0 4 −2 11 −1 8
RRN9 −1 2 1 8 9 −11 −7 −5 −1 0 1 3 −2 14 1 6
TOM40 −1 1 0 6 7 −9 −9 −6 0 −1 0 2 0 14 0 7
TIF5 0 1 3 7 9 −10 −5 −6 0 1 0 0 −1 13 −2 7
RPG1 −3 2 2 7 6 −8 −7 −5 −1 0 0 3 −2 10 −1 4
RSE1 −3 2 0 8 7 −9 −8 −4 1 −1 0 2 2 11 −2 2
SUA5 −2 3 3 4 7 −6 −9 −5 0 0 0 0 −2 16 0 6
SNP1 −3 3 2 3 5 −7 −6 −6 0 0 0 1 −4 18 0 9
PRP3 0 4 4 4 8 −5 −7 −8 −2 1 1 1 −6 15 −1 7
UTP9 0 2 1 4 7 −3 −4 −2 −1 0 0 2 −3 19 −1 5
RFA1 1 3 2 4 8 −7 −5 −6 −1 1 0 1 4 13 −2 11
GPI19 −2 2 2 2 7 −6 −9 −9 1 0 0 0 2 14 1 7
SEN2 0 5 4 7 9 −8 −9 −3 1 −2 −2 1 −6 23 4 10
Gene Name
Type of Media

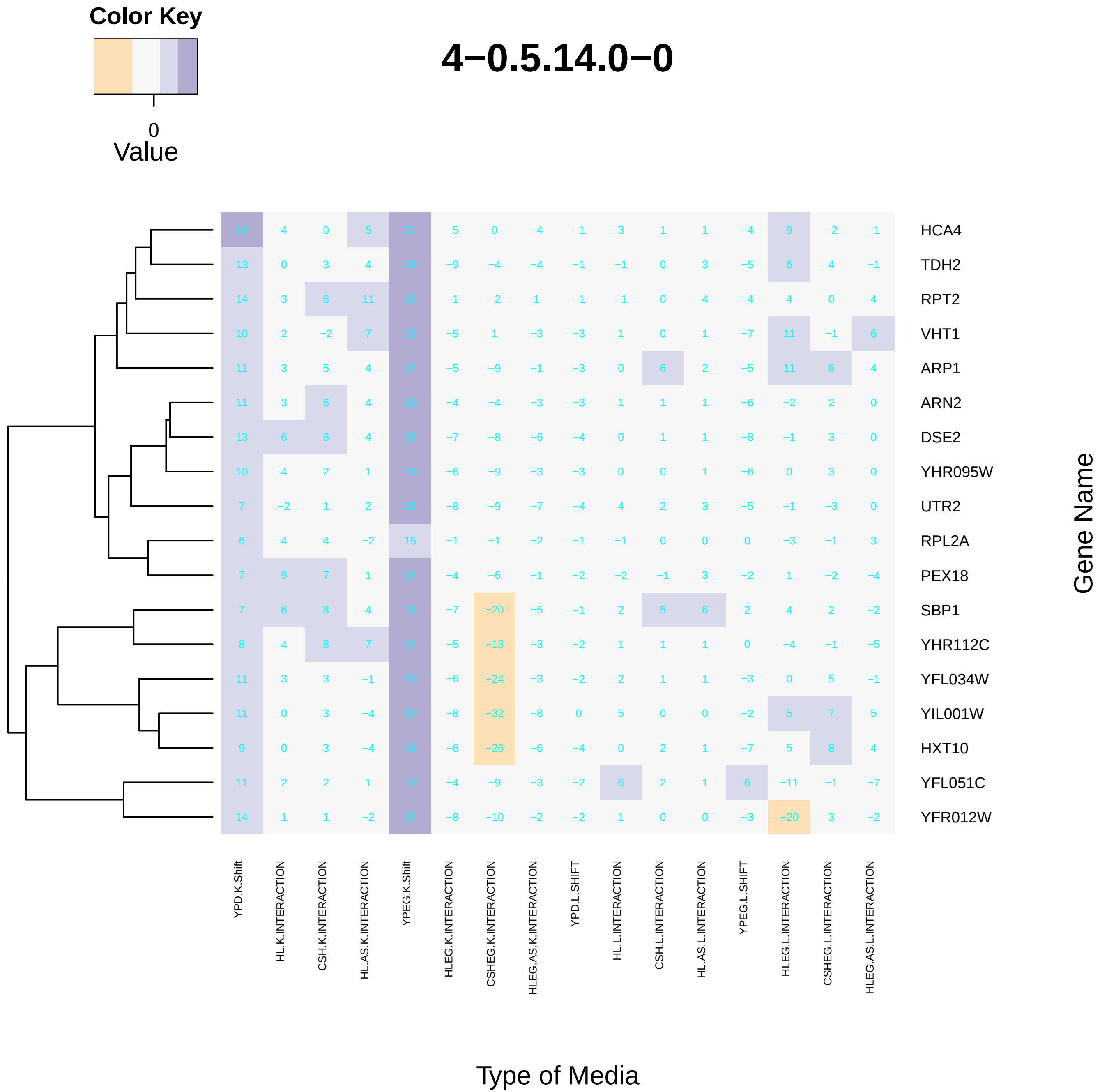
Color Key
0
Value
4−0.5.14.0−0
YPD.K.Shift
HL.K.INTERACTION
CSH.K.INTERACTION
HL.AS.K.INTERACTION
YPEG.K.Shift
HLEG.K.INTERACTION
CSHEG.K.INTERACTION
HLEG.AS.K.INTERACTION
YPD.L.SHIFT
HL.L.INTERACTION
CSH.L.INTERACTION
HL.AS.L.INTERACTION
YPEG.L.SHIFT
HLEG.L.INTERACTION
CSHEG.L.INTERACTION
HLEG.AS.L.INTERACTION
15 4 0 5 21 −5 0 −4 −1 3 1 1 −4 9 −2 −1 HCA4
13 0 3 4 18 −9 −4 −4 −1 −1 0 3 −5 6 4 −1 TDH2
14 3 6 11 23 −1 −2 1 −1 −1 0 4 −4 4 0 4 RPT2
10 2 −2 7 15 −5 1 −3 −3 1 0 1 −7 11 −1 6 VHT1
11 3 5 4 17 −5 −9 −1 −3 0 6 2 −5 11 8 4 ARP1
11 3 6 4 20 −4 −4 −3 −3 1 1 1 −6 −2 2 0 ARN2
13 6 6 4 23 −7 −8 −6 −4 0 1 1 −8 −1 3 0 DSE2
10 4 2 1 19 −6 −9 −3 −3 0 0 1 −6 0 3 0 YHR095W
7 −2 1 2 18 −8 −9 −7 −4 4 2 3 −5 −1 −3 0 UTR2
6 4 4 −2 15 −1 −1 −2 −1 −1 0 0 0 −3 −1 3 RPL2A
7 9 7 1 16 −4 −6 −1 −2 −2 −1 3 −2 1 −2 −4 PEX18
7 6 8 4 19 −7 −20 −5 −1 2 5 6 2 4 2 −2 SBP1
8 4 8 7 17 −5 −13 −3 −2 1 1 1 0 −4 −1 −5 YHR112C
11 3 3 −1 20 −6 −24 −3 −2 2 1 1 −3 0 5 −1 YFL034W
11 0 3 −4 20 −8 −32 −8 0 5 0 0 −2 5 7 5 YIL001W
9 0 3 −4 19 −6 −26 −6 −4 0 2 1 −7 5 8 4 HXT10
11 2 2 1 19 −4 −9 −3 −2 6 2 1 6 −11 −1 −7 YFL051C
14 1 1 −2 22 −8 −10 −2 −2 1 0 0 −3 −20 3 −2 YFR012W
Gene Name
Type of Media

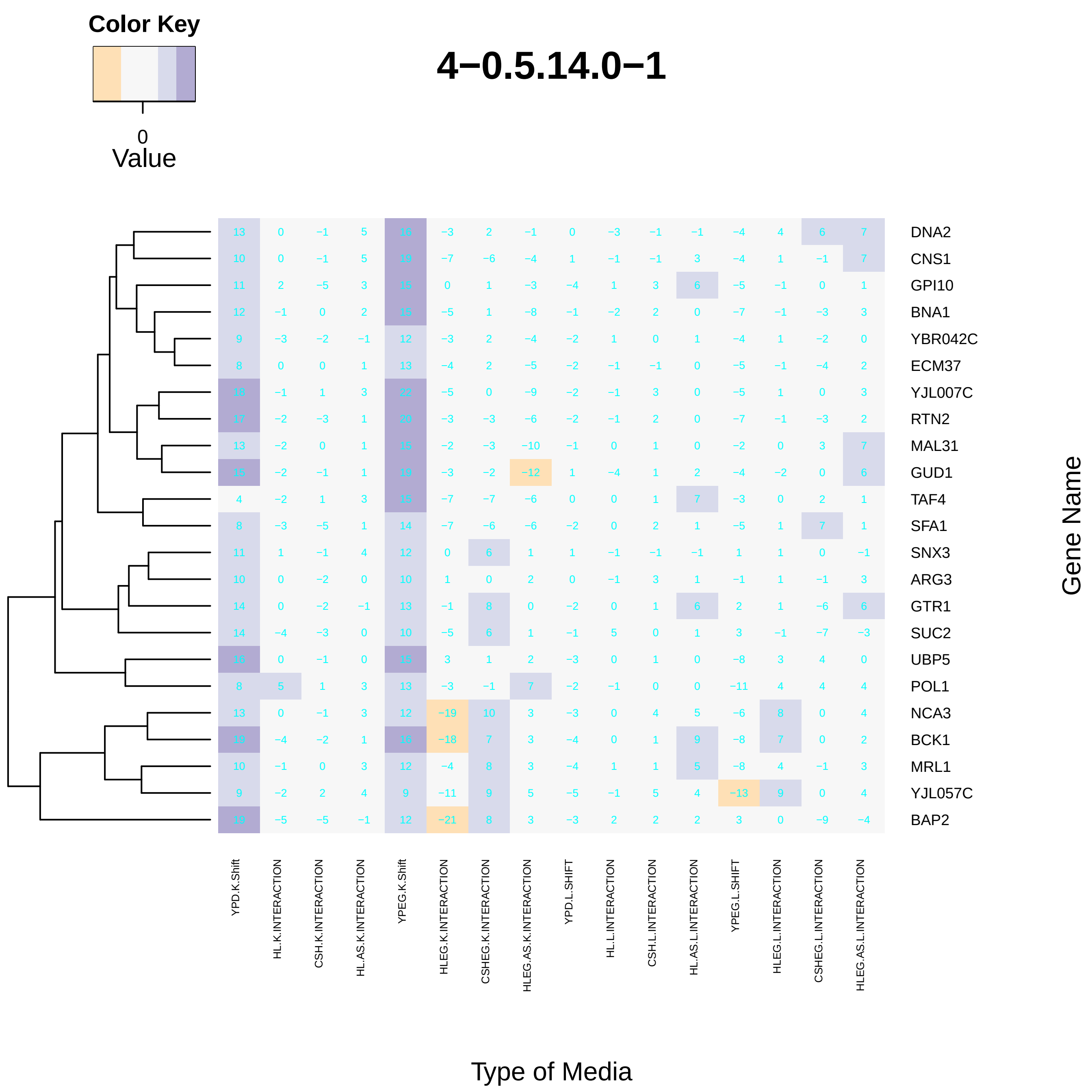

Color Key
0
Value
4−0.5.14.0−1
DNA2 13 0 −1 5 16 −3 2 −1 0 −3 −1 −1 −4 4 6 7
CNS1 10 0 −1 5 19 −7 −6 −4 1 −1 −1 3 −4 1 −1 7
GPI10 11 2 −5 3 15 0 1 −3 −4 1 3 6 −5 −1 0 1
BNA1 12 −1 0 2 15 −5 1 −8 −1 −2 2 0 −7 −1 −3 3
YBR042C 9 −3 −2 −1 12 −3 2 −4 −2 1 0 1 −4 1 −2 0
ECM37 8 0 0 1 13 −4 2 −5 −2 −1 −1 0 −5 −1 −4 2
YJL007C 18 −1 1 3 22 −5 0 −9 −2 −1 3 0 −5 1 0 3
RTN2 17 −2 −3 1 20 −3 −3 −6 −2 −1 2 0 −7 −1 −3 2
MAL31 13 −2 0 1 15 −2 −3 −10 −1 0 1 0 −2 0 3 7
GUD1 16 −2 −1 1 19 −3 −2 −12 1 −4 1 2 −4 −2 0 6
TAF4 4 −2 1 3 15 −7 −7 −6 0 0 1 7 −3 0 2 1
SFA1 8 −3 −5 1 14 −7 −6 −6 −2 0 2 1 −5 1 7 1
SNX3 11 1 −1 4 12 0 6 1 1 −1 −1 −1 1 1 0 −1
ARG3 10 0 −2 0 10 1 0 2 0 −1 3 1 −1 1 −1 3
GTR1 14 0 −2 −1 13 −1 8 0 −2 0 1 6 2 1 −6 6
SUC2 14 −4 −3 0 10 −5 6 1 −1 5 0 1 3 −1 −7 −3
UBP5 16 0 −1 0 15 3 1 2 −3 0 1 0 −8 3 4 0
POL1 8 5 1 3 13 −3 −1 7 −2 −1 0 0 −11 4 4 4
NCA3 13 0 −1 3 12 −19 10 3 −3 0 4 5 −6 8 0 4
BCK1 19 −4 −2 1 16 −18 7 3 −4 0 1 9 −8 7 0 2
MRL1 10 −1 0 3 12 −4 8 3 −4 1 1 5 −8 4 −1 3
YJL057C 9 −2 2 4 9 −11 9 5 −5 −1 5 4 −13 9 0 4
BAP2 19 −5 −5 −1 12 −21 8 3 −3 2 2 2 3 0 −9 −4
YPD.K.Shift
HL.K.INTERACTION
CSH.K.INTERACTION
HL.AS.K.INTERACTION
YPEG.K.Shift
HLEG.K.INTERACTION
CSHEG.K.INTERACTION
HLEG.AS.K.INTERACTION
YPD.L.SHIFT
HL.L.INTERACTION
CSH.L.INTERACTION
HL.AS.L.INTERACTION
YPEG.L.SHIFT
HLEG.L.INTERACTION
CSHEG.L.INTERACTION
HLEG.AS.L.INTERACTION
Gene Name
Type of Media

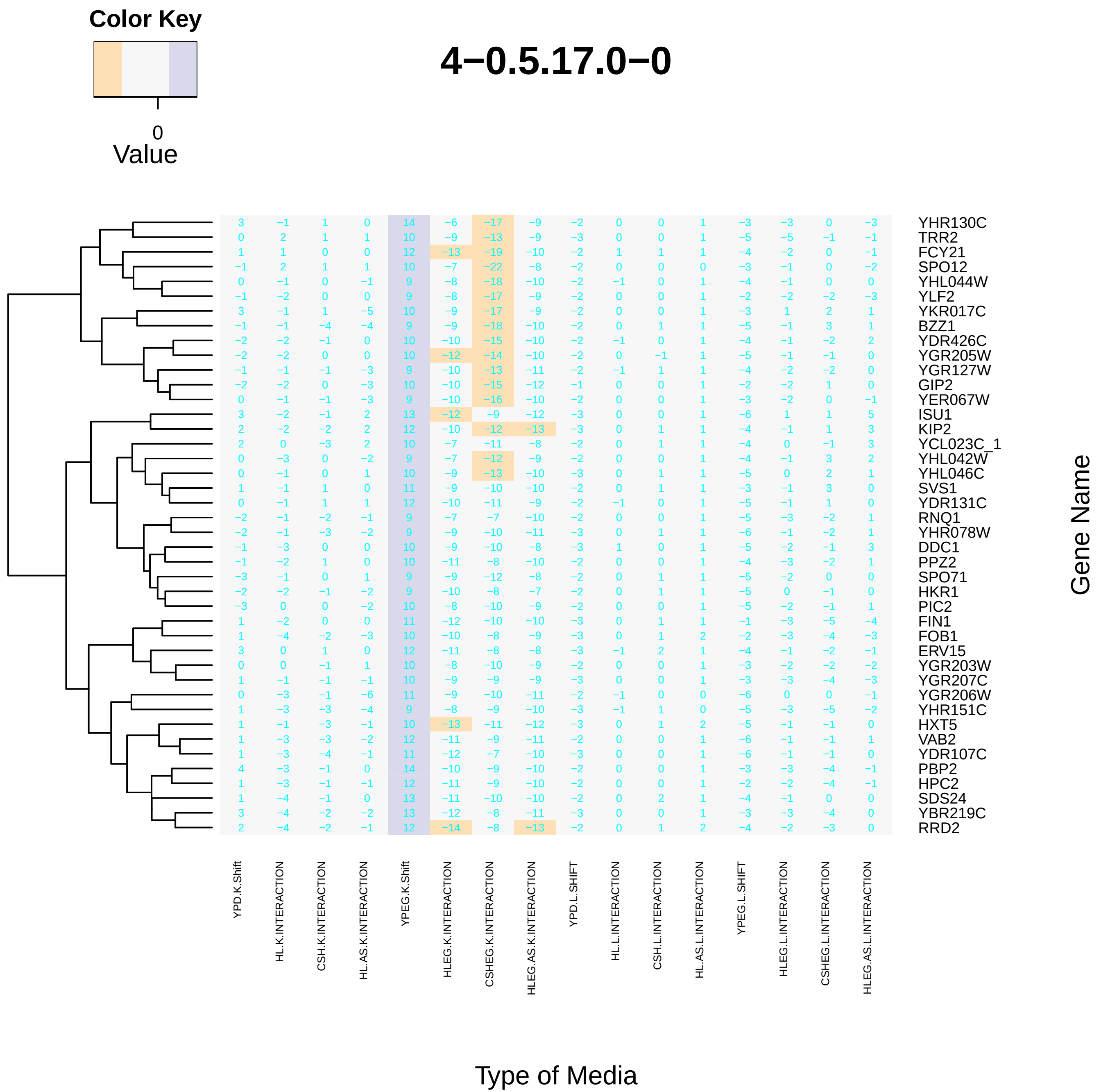
4−0.5.17.0−0
Color Key
0
Value
Gene Name
Type of Media
YPD.K.Shift
HL.K.INTERACTION
CSH.K.INTERACTION
HL.AS.K.INTERACTION
YPEG.K.Shift
HLEG.K.INTERACTION
CSHEG.K.INTERACTION
HLEG.AS.K.INTERACTION
YPD.L.SHIFT
HL.L.INTERACTION
CSH.L.INTERACTION
HL.AS.L.INTERACTION
YPEG.L.SHIFT
HLEG.L.INTERACTION
CSHEG.L.INTERACTION
HLEG.AS.L.INTERACTION
YHR130C
TRR2
FCY21
SPO12
YHL044W
YLF2
YKR017C
BZZ1
YDR426C
YGR205W
YGR127W
GIP2
YER067W
ISU1
KIP2
YCL023C_1
YHL042W
YHL046C
SVS1
YDR131C
RNQ1
YHR078W
DDC1
PPZ2
SPO71
HKR1
PIC2
FIN1
FOB1
ERV15
YGR203W
YGR207C
YGR206W
YHR151C
HXT5
VAB2
YDR107C
PBP2
HPC2
SDS24
YBR219C
RRD2

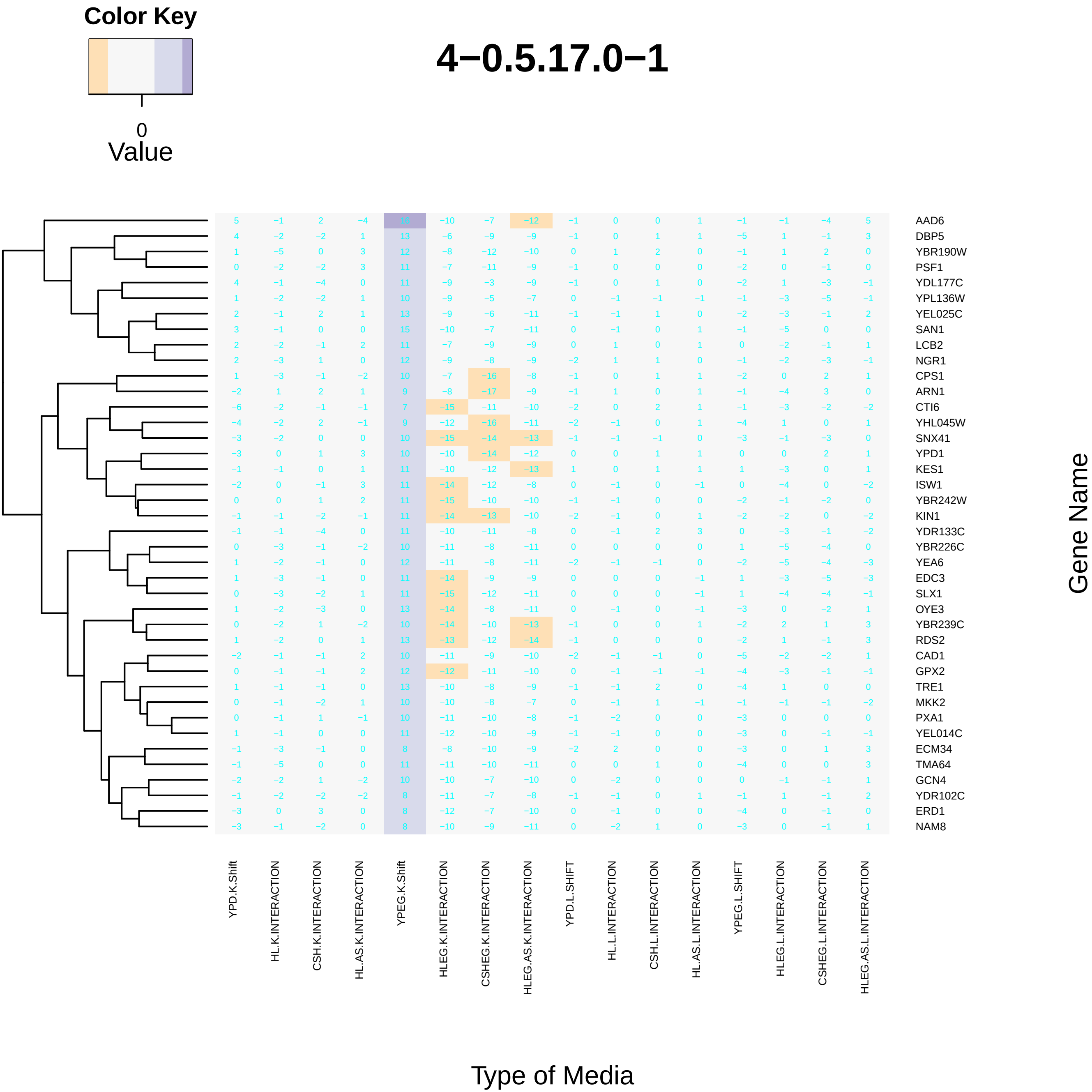
Color Key
0
Value
4−0.5.17.0−1
AAD6
DBP5
YBR190W
PSF1
YDL177C
YPL136W
YEL025C
SAN1
LCB2
NGR1
CPS1
ARN1
CTI6
YHL045W
SNX41
YPD1
KES1
ISW1
YBR242W
KIN1
YDR133C
YBR226C
YEA6
EDC3
SLX1
OYE3
YBR239C
RDS2
CAD1
GPX2
TRE1
MKK2
PXA1
YEL014C
ECM34
TMA64
GCN4
YDR102C
ERD1
NAM8
Gene Name
YPD.K.Shift
HL.K.INTERACTION
CSH.K.INTERACTION
HL.AS.K.INTERACTION
YPEG.K.Shift
HLEG.K.INTERACTION
CSHEG.K.INTERACTION
HLEG.AS.K.INTERACTION
YPD.L.SHIFT
HL.L.INTERACTION
CSH.L.INTERACTION
HL.AS.L.INTERACTION
YPEG.L.SHIFT
HLEG.L.INTERACTION
CSHEG.L.INTERACTION
HLEG.AS.L.INTERACTION
Type of Media

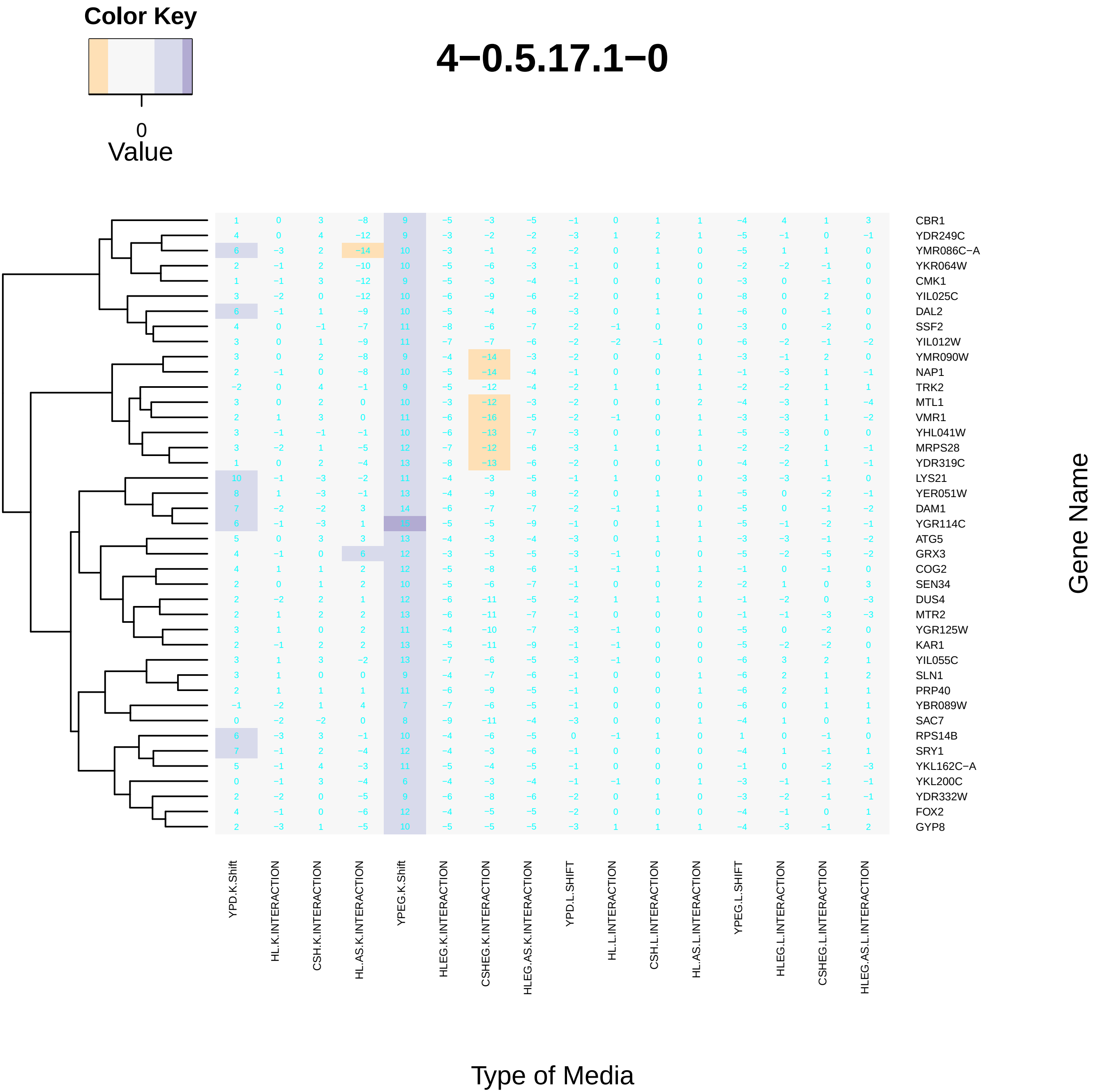

Color Key
0
Value
4−0.5.17.1−0
CBR1
YDR249C
YMR086C−A
YKR064W
CMK1
YIL025C
DAL2
SSF2
YIL012W
YMR090W
NAP1
TRK2
MTL1
VMR1
YHL041W
MRPS28
YDR319C
LYS21
YER051W
DAM1
YGR114C
ATG5
GRX3
COG2
SEN34
DUS4
MTR2
YGR125W
KAR1
YIL055C
SLN1
PRP40
YBR089W
SAC7
RPS14B
SRY1
YKL162C−A
YKL200C
YDR332W
FOX2
GYP8
Gene Name
YPD.K.Shift
HL.K.INTERACTION
CSH.K.INTERACTION
HL.AS.K.INTERACTION
YPEG.K.Shift
HLEG.K.INTERACTION
CSHEG.K.INTERACTION
HLEG.AS.K.INTERACTION
YPD.L.SHIFT
HL.L.INTERACTION
CSH.L.INTERACTION
HL.AS.L.INTERACTION
YPEG.L.SHIFT
HLEG.L.INTERACTION
CSHEG.L.INTERACTION
HLEG.AS.L.INTERACTION
Type of Media

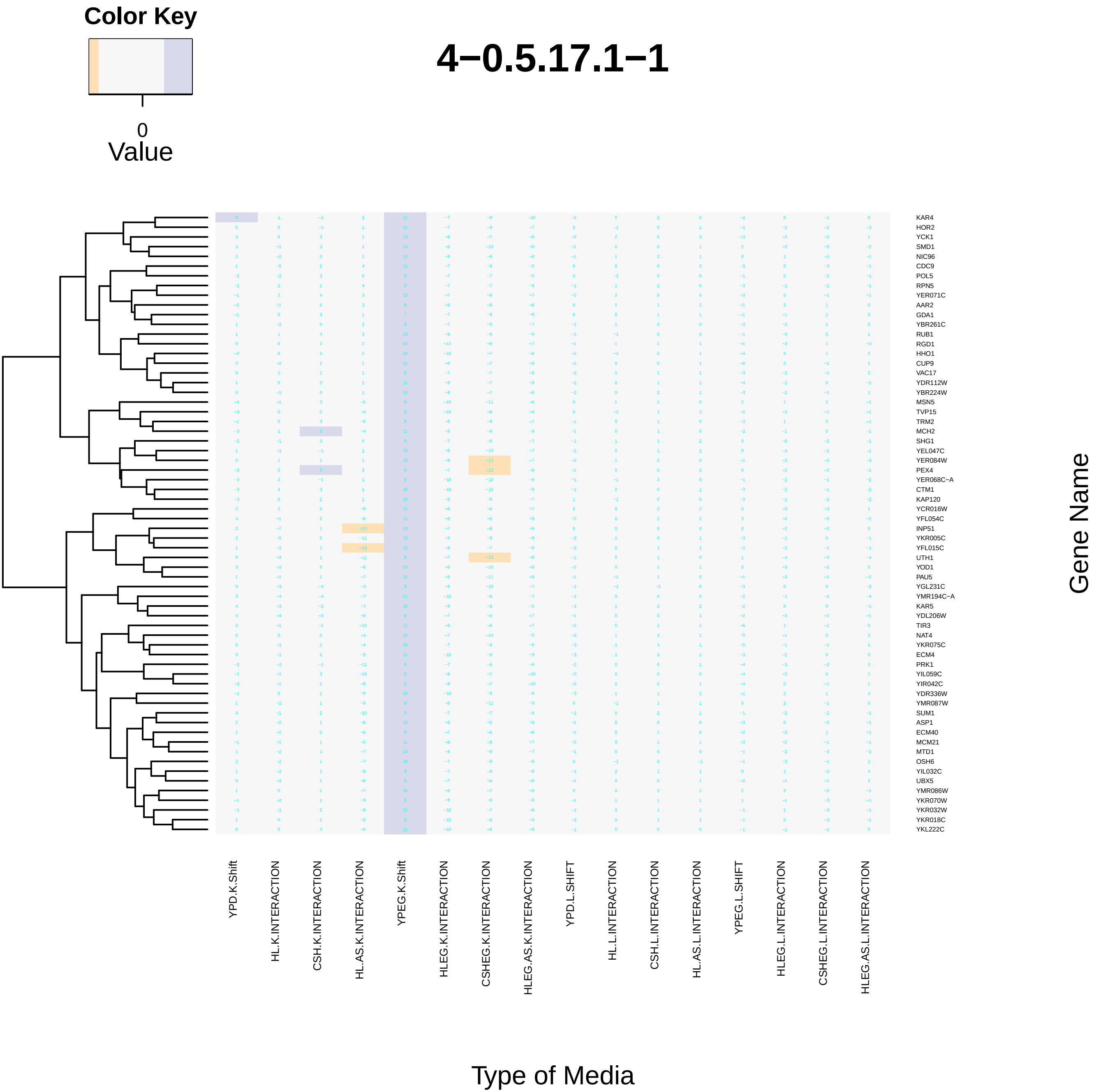
Color Key
0
Value
4−0.5.17.1−1
KAR4
HOR2
YCK1
SMD1
NIC96
CDC9
POL5
RPN5
YER071C
AAR2
GDA1
YBR261C
RUB1
RGD1
HHO1
CUP9
VAC17
YDR112W
YBR224W
MSN5
TVP15
TRM2
MCH2
SHG1
YEL047C
YER084W
PEX4
YER068C−A
CTM1
KAP120
YCR016W
YFL054C
INP51
YKR005C
YFL015C
UTH1
YOD1
PAU5
YGL231C
YMR194C−A
KAR5
YDL206W
TIR3
NAT4
YKR075C
ECM4
PRK1
YIL059C
YIR042C
YDR336W
YMR087W
SUM1
ASP1
ECM40
MCM21
MTD1
OSH6
YIL032C
UBX5
YMR086W
YKR070W
YKR032W
YKR018C
YKL222C
Gene Name
YPD.K.Shift
HL.K.INTERACTION
CSH.K.INTERACTION
HL.AS.K.INTERACTION
YPEG.K.Shift
HLEG.K.INTERACTION
CSHEG.K.INTERACTION
HLEG.AS.K.INTERACTION
YPD.L.SHIFT
HL.L.INTERACTION
CSH.L.INTERACTION
HL.AS.L.INTERACTION
YPEG.L.SHIFT
HLEG.L.INTERACTION
CSHEG.L.INTERACTION
HLEG.AS.L.INTERACTION
Type of Media

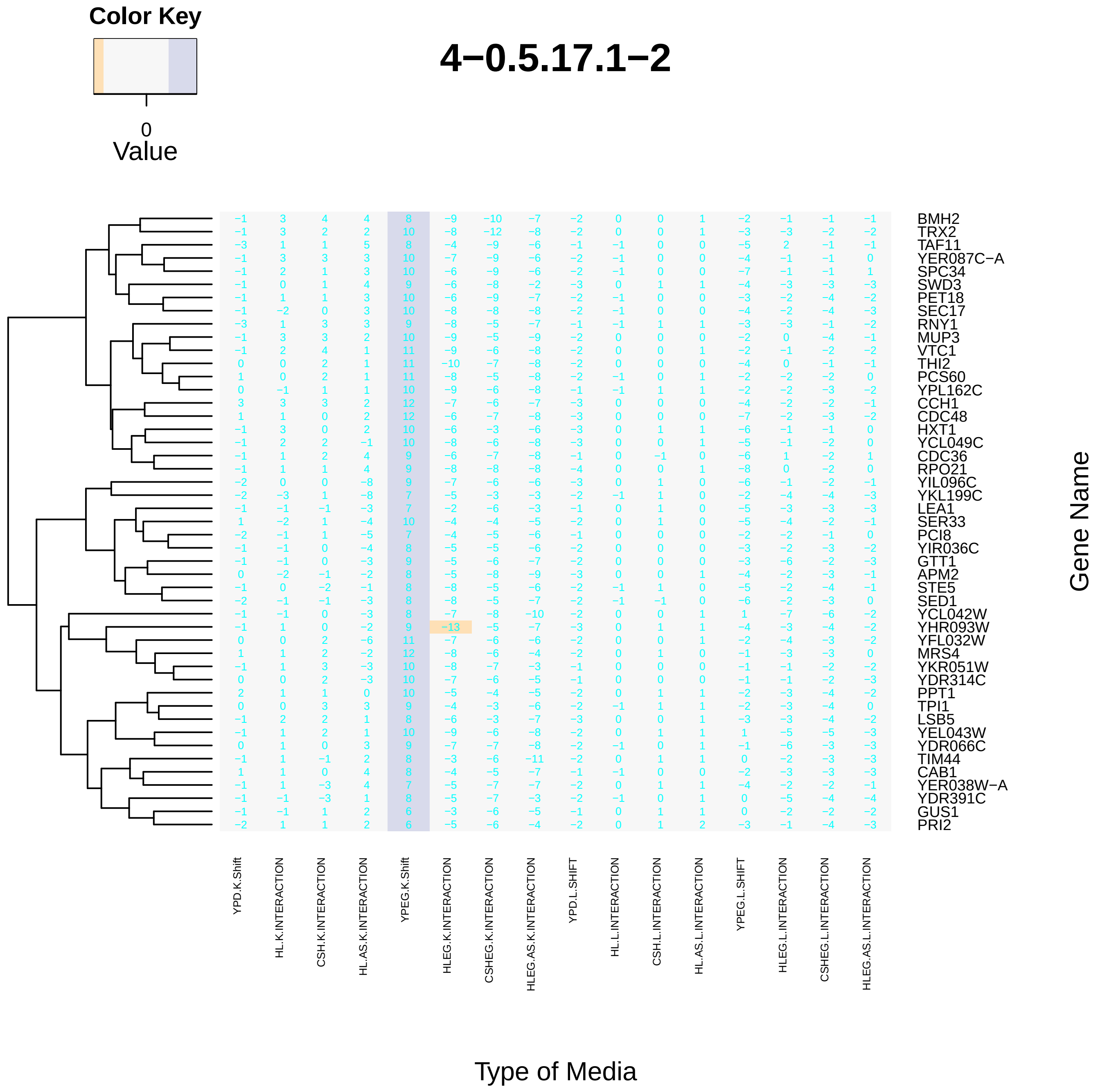
Color Key
0
Value
4−0.5.17.1−2
YPD.K.Shift HL.K.INTERACTION CSH.K.INTERACTION HL.AS.K.INTERACTION YPEG.K.Shift HLEG.K.INTERACTION CSHEG.K.INTERACTION HLEG.AS.K.INTERACTION YPD.L.SHIFT HL.L.INTERACTION CSH.L.INTERACTION HL.AS.L.INTERACTION YPEG.L.SHIFT HLEG.L.INTERACTION CSHEG.L.INTERACTION HLEG.AS.L.INTERACTION
BMH2 −1 3 4 4 8 −9 −10 −7 −2 0 0 1 −2 −1 −1 −1
TRX2 −1 3 2 2 10 −8 −12 −8 −2 0 0 1 −3 −3 −2 −2
TAF11 −3 1 1 5 8 −4 −9 −6 −1 −1 0 0 −5 2 −1 −1
YER087C−A −1 3 3 3 10 −7 −9 −6 −2 −1 0 0 −4 −1 −1 0
SPC34 −1 2 1 3 10 −6 −9 −6 −2 −1 0 0 −7 −1 −1 1
SWD3 −1 0 1 4 9 −6 −8 −2 −3 0 1 1 −4 −3 −3 −3
PET18 −1 1 1 3 10 −6 −9 −7 −2 −1 0 0 −3 −2 −4 −2
SEC17 −1 −2 0 3 10 −8 −8 −8 −2 −1 0 0 −4 −2 −4 −3
RNY1 −3 1 3 3 9 −8 −5 −7 −1 −1 1 1 −3 −3 −1 −2
MUP3 −1 3 3 2 10 −9 −5 −9 −2 0 0 0 −2 0 −4 −1
VTC1 −1 2 4 1 11 −9 −6 −8 −2 0 0 1 −2 −1 −2 −2
THI2 0 0 2 1 11 −10 −7 −8 −2 0 0 0 −4 0 −1 −1
PCS60 1 0 2 1 11 −8 −5 −8 −2 −1 0 1 −2 −2 −2 0
YPL162C 0 −1 1 1 10 −9 −6 −8 −1 −1 1 1 −2 −2 −3 −2
CCH1 3 3 3 2 12 −7 −6 −7 −3 0 0 0 −4 −2 −2 −1
CDC48 1 1 0 2 12 −6 −7 −8 −3 0 0 0 −7 −2 −3 −2
HXT1 −1 3 0 2 10 −6 −3 −6 −3 0 1 1 −6 −1 −1 0
YCL049C −1 2 2 −1 10 −8 −6 −8 −3 0 0 1 −5 −1 −2 0
CDC36 −1 1 2 4 9 −6 −7 −8 −1 0 −1 0 −6 1 −2 1
RPO21 −1 1 1 4 9 −8 −8 −8 −4 0 0 1 −8 0 −2 0
YIL096C −2 0 0 −8 9 −7 −6 −6 −3 0 1 0 −6 −1 −2 −1
YKL199C −2 −3 1 −8 7 −5 −3 −3 −2 −1 1 0 −2 −4 −4 −3
LEA1 −1 −1 −1 −3 7 −2 −6 −3 −1 0 1 0 −5 −3 −3 −3
SER33 1 −2 1 −4 10 −4 −4 −5 −2 0 1 0 −5 −4 −2 −1
PCI8 −2 −1 1 −5 7 −4 −5 −6 −1 0 0 0 −2 −2 −1 0
YIR036C −1 −1 0 −4 8 −5 −5 −6 −2 0 0 0 −3 −2 −3 −2
GTT1 −1 −1 0 −3 9 −5 −6 −7 −2 0 0 0 −3 −6 −2 −3
APM2 0 −2 −1 −2 8 −5 −8 −9 −3 0 0 1 −4 −2 −3 −1
STE5 −1 0 −2 −1 8 −8 −5 −6 −2 −1 1 0 −5 −2 −4 −1
SED1 −2 −1 −1 −3 8 −8 −5 −7 −2 −1 −1 0 −6 −2 −3 0
YCL042W −1 −1 0 −3 8 −7 −8 −10 −2 0 0 1 1 −7 −6 −2
YHR093W −1 1 0 −2 9 −13 −5 −7 −3 0 1 1 −4 −3 −4 −2
YFL032W 0 0 2 −6 11 −7 −6 −6 −2 0 0 1 −2 −4 −3 −2
MRS4 1 1 2 −2 12 −8 −6 −4 −2 0 1 0 −1 −3 −3 0
YKR051W −1 1 3 −3 10 −8 −7 −3 −1 0 0 0 −1 −1 −2 −2
YDR314C 0 0 2 −3 10 −7 −6 −5 −1 0 0 0 −1 −1 −2 −3
PPT1 2 1 1 0 10 −5 −4 −5 −2 0 1 1 −2 −3 −4 −2
TPI1 0 0 3 3 9 −4 −3 −6 −2 −1 1 1 −2 −3 −4 0
LSB5 −1 2 2 1 8 −6 −3 −7 −3 0 0 1 −3 −3 −4 −2
YEL043W −1 1 2 1 10 −9 −6 −8 −2 0 1 1 1 −5 −5 −3
YDR066C 0 1 0 3 9 −7 −7 −8 −2 −1 0 1 −1 −6 −3 −3
TIM44 −1 1 −1 2 8 −3 −6 −11 −2 0 1 1 0 −2 −3 −3
CAB1 1 1 0 4 8 −4 −5 −7 −1 −1 0 0 −2 −3 −3 −3
YER038W−A −1 1 −3 4 7 −5 −7 −7 −2 0 1 1 −4 −2 −2 −1
YDR391C −1 −1 −3 1 8 −5 −7 −3 −2 −1 0 1 0 −5 −4 −4
GUS1 −1 −1 1 2 6 −3 −6 −5 −1 0 1 1 0 −2 −2 −2
PRI2 −2 1 1 2 6 −5 −6 −4 −2 0 1 2 −3 −1 −4 −3
Gene Name
Type of Media

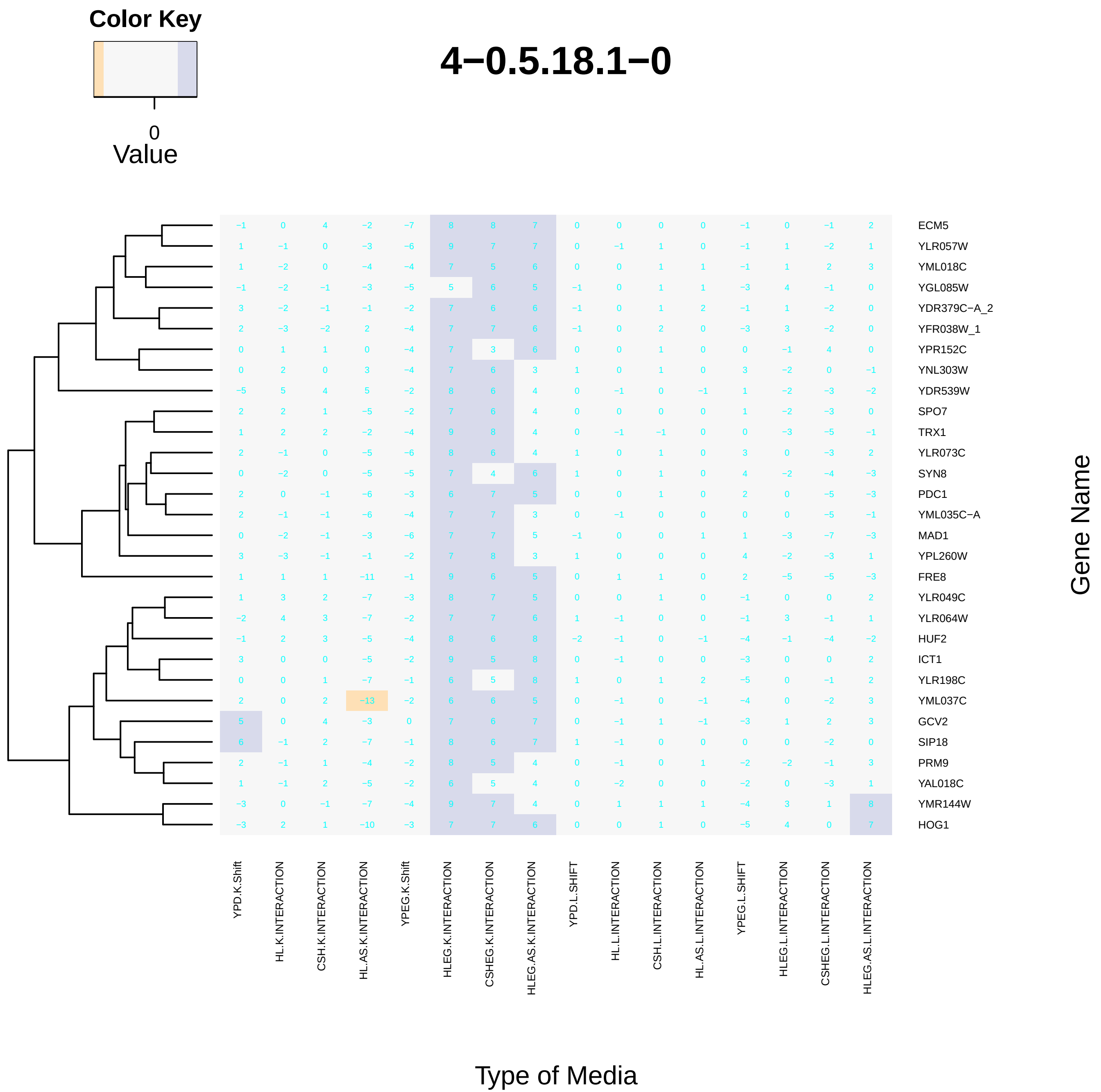
Color Key
0
Value
4−0.5.18.1−0
ECM5
YLR057W
YML018C
YGL085W
YDR379C−A_2
YFR038W_1
YPR152C
YNL303W
YDR539W
SPO7
TRX1
YLR073C
SYN8
PDC1
YML035C−A
MAD1
YPL260W
FRE8
YLR049C
YLR064W
HUF2
ICT1
YLR198C
YML037C
GCV2
SIP18
PRM9
YAL018C
YMR144W
HOG1
Gene Name
YPD.K.Shift
HL.K.INTERACTION
CSH.K.INTERACTION
HL.AS.K.INTERACTION
YPEG.K.Shift
HLEG.K.INTERACTION
CSHEG.K.INTERACTION
HLEG.AS.K.INTERACTION
YPD.L.SHIFT
HL.L.INTERACTION
CSH.L.INTERACTION
HL.AS.L.INTERACTION
YPEG.L.SHIFT
HLEG.L.INTERACTION
CSHEG.L.INTERACTION
HLEG.AS.L.INTERACTION
Type of Media

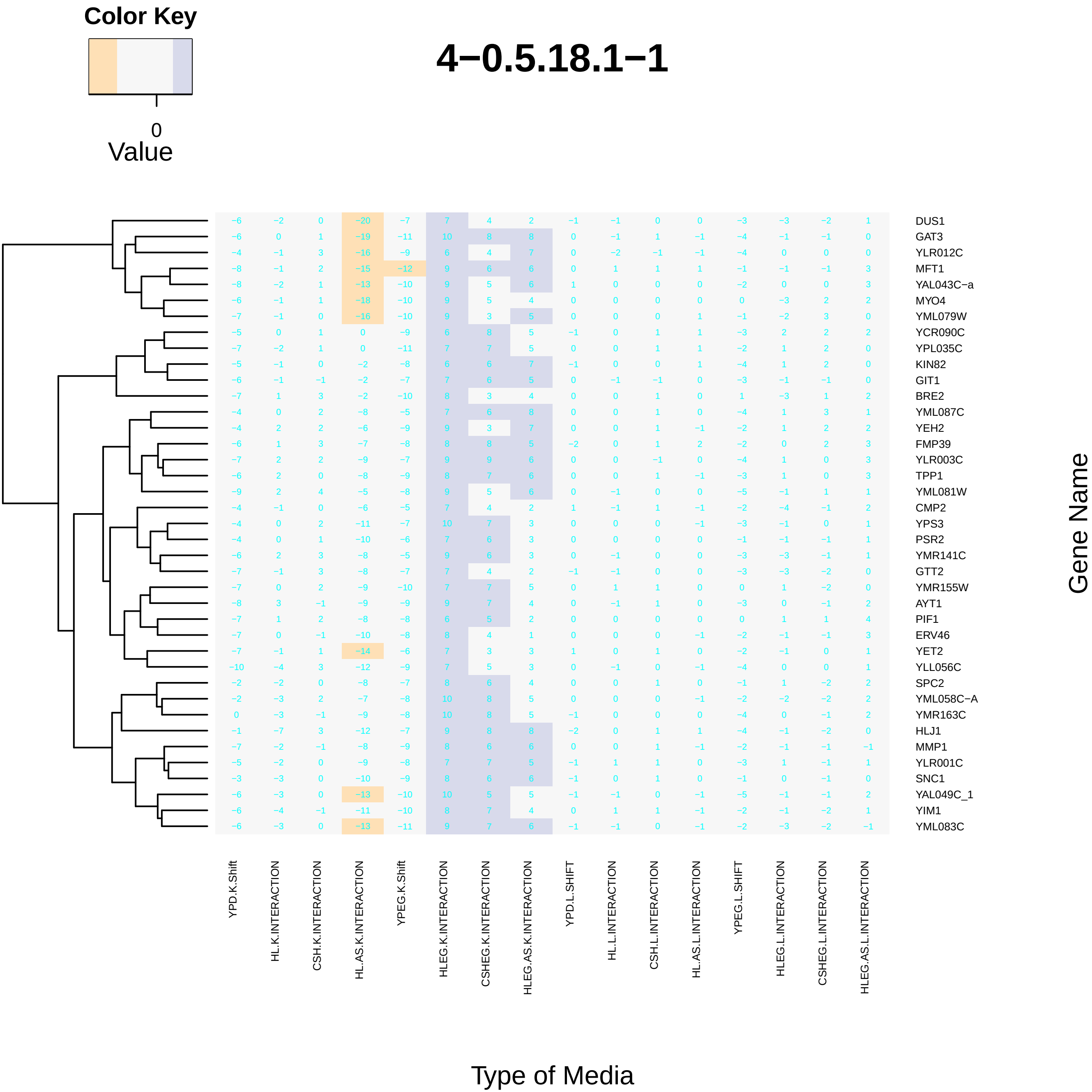
4−0.5.18.1−1
Color Key
0
Value
Type of Media
Gene Name
YPD.K.Shift
HL.K.INTERACTION
CSH.K.INTERACTION
HL.AS.K.INTERACTION
YPEG.K.Shift
HLEG.K.INTERACTION
CSHEG.K.INTERACTION
HLEG.AS.K.INTERACTION
YPD.L.SHIFT
HL.L.INTERACTION
CSH.L.INTERACTION
HL.AS.L.INTERACTION
YPEG.L.SHIFT
HLEG.L.INTERACTION
CSHEG.L.INTERACTION
HLEG.AS.L.INTERACTION
DUS1 −6 −2 0 −20 −7 7 4 2 −1 −1 0 0 −3 −3 −2 1
GAT3 −6 0 1 −19 −11 10 8 8 0 −1 1 −1 −4 −1 −1 0
YLR012C −4 −1 3 −16 −9 6 4 7 0 −2 −1 −1 −4 0 0 0
MFT1 −8 −1 2 −15 −12 9 6 6 0 1 1 1 −1 −1 −1 3
YAL043C−a −8 −2 1 −13 −10 9 5 6 1 0 0 0 −2 0 0 3
MYO4 −6 −1 1 −18 −10 9 5 4 0 0 0 0 0 −3 2 2
YML079W −7 −1 0 −16 −10 9 3 5 0 0 0 1 −1 −2 3 0
YCR090C −5 0 1 0 −9 6 8 5 −1 0 1 1 −3 2 2 2
YPL035C −7 −2 1 0 −11 7 7 5 0 0 1 1 −2 1 2 0
KIN82 −5 −1 0 −2 −8 6 6 7 −1 0 0 1 −4 1 2 0
GIT1 −6 −1 −1 −2 −7 7 6 5 0 −1 −1 0 −3 −1 −1 0
BRE2 −7 1 3 −2 −10 8 3 4 0 0 1 0 1 −3 1 2
YML087C −4 0 2 −8 −5 7 6 8 0 0 1 0 −4 1 3 1
YEH2 −4 2 2 −6 −9 9 3 7 0 0 1 −1 −2 1 2 2
FMP39 −6 1 3 −7 −8 8 8 5 −2 0 1 2 −2 0 2 3
YLR003C −7 2 2 −9 −7 9 9 6 0 0 −1 0 −4 1 0 3
TPP1 −6 2 0 −8 −9 8 7 6 0 0 1 −1 −3 1 0 3
YML081W −9 2 4 −5 −8 9 5 6 0 −1 0 0 −5 −1 1 1
CMP2 −4 −1 0 −6 −5 7 4 2 1 −1 1 −1 −2 −4 −1 2
YPS3 −4 0 2 −11 −7 10 7 3 0 0 0 −1 −3 −1 0 1
PSR2 −4 0 1 −10 −6 7 6 3 0 0 0 0 −1 −1 −1 1
YMR141C −6 2 3 −8 −5 9 6 3 0 −1 0 0 −3 −3 −1 1
GTT2 −7 −1 3 −8 −7 7 4 2 −1 −1 0 0 −3 −3 −2 0
YMR155W −7 0 2 −9 −10 7 7 5 0 1 1 0 0 1 −2 0
AYT1 −8 3 −1 −9 −9 9 7 4 0 −1 1 0 −3 0 −1 2
PIF1 −7 1 2 −8 −8 6 5 2 0 0 0 0 0 1 1 4
ERV46 −7 0 −1 −10 −8 8 4 1 0 0 0 −1 −2 −1 −1 3
YET2 −7 −1 1 −14 −6 7 3 3 1 0 1 0 −2 −1 0 1
YLL056C −10 −4 3 −12 −9 7 5 3 0 −1 0 −1 −4 0 0 1
SPC2 −2 −2 0 −8 −7 8 6 4 0 0 1 0 −1 1 −2 2
YML058C−A −2 −3 2 −7 −8 10 8 5 0 0 0 −1 −2 −2 −2 2
YMR163C 0 −3 −1 −9 −8 10 8 5 −1 0 0 0 −4 0 −1 2
HLJ1 −1 −7 3 −12 −7 9 8 8 −2 0 1 1 −4 −1 −2 0
MMP1 −7 −2 −1 −8 −9 8 6 6 0 0 1 −1 −2 −1 −1 −1
YLR001C −5 −2 0 −9 −8 7 7 5 −1 1 1 0 −3 1 −1 1
SNC1 −3 −3 0 −10 −9 8 6 6 −1 0 1 0 −1 0 −1 0
YAL049C_1 −6 −3 0 −13 −10 10 5 5 −1 −1 0 −1 −5 −1 −1 2
YIM1 −6 −4 −1 −11 −10 8 7 4 0 1 1 −1 −2 −1 −2 1
YML083C −6 −3 0 −13 −11 9 7 6 −1 −1 0 −1 −2 −3 −2 −1

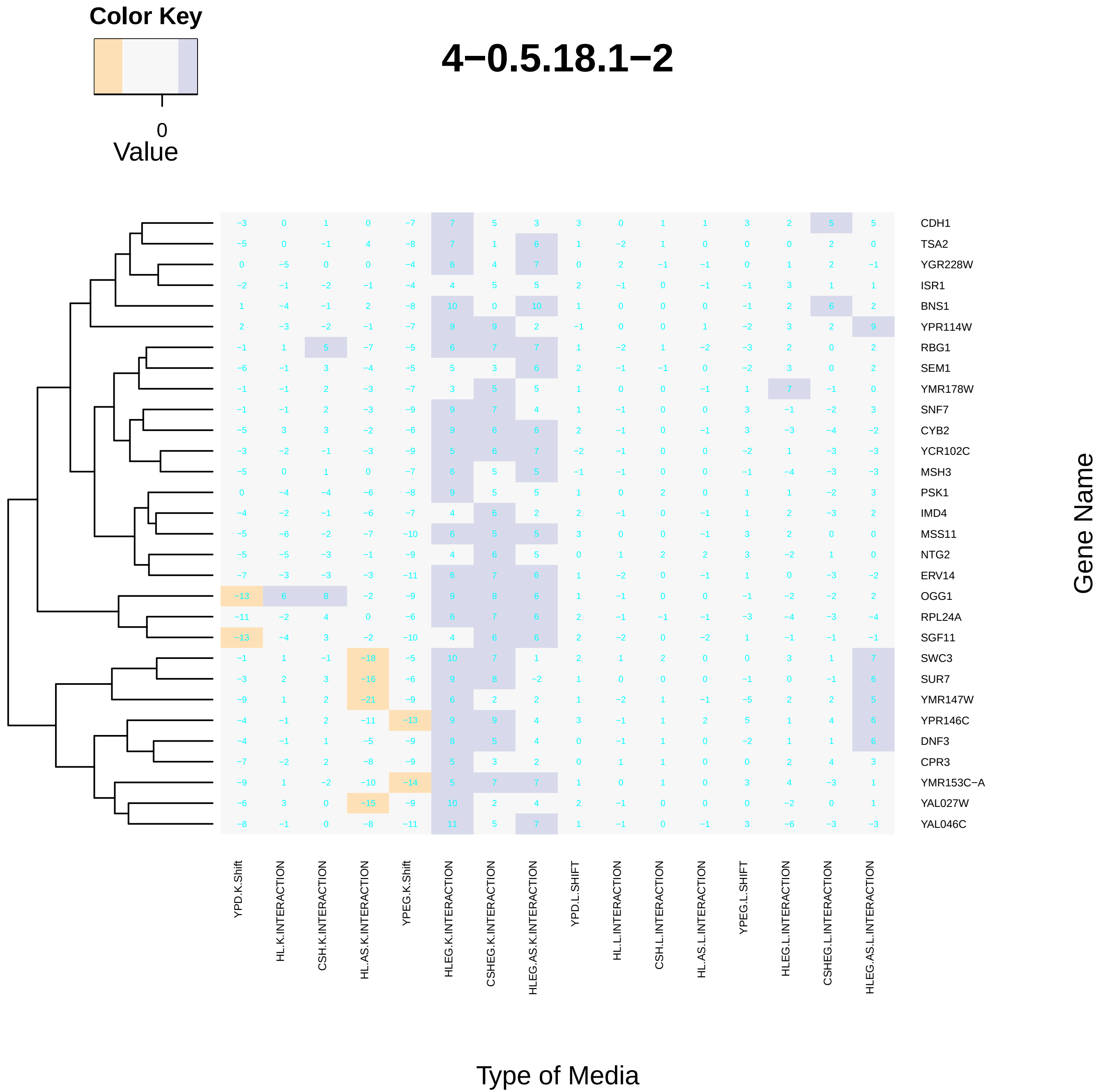
Color Key
0
Value
4−0.5.18.1−2
YPD.K.Shift HL.K.INTERACTION CSH.K.INTERACTION HL.AS.K.INTERACTION YPEG.K.Shift HLEG.K.INTERACTION CSHEG.K.INTERACTION HLEG.AS.K.INTERACTION YPD.L.SHIFT HL.L.INTERACTION CSH.L.INTERACTION HL.AS.L.INTERACTION YPEG.L.SHIFT HLEG.L.INTERACTION CSHEG.L.INTERACTION HLEG.AS.L.INTERACTION
CDH1 −3 0 1 0 −7 7 5 3 3 0 1 1 3 2 5 5
TSA2 −5 0 −1 4 −8 7 1 6 1 −2 1 0 0 0 2 0
YGR228W 0 −5 0 0 −4 6 4 7 0 2 −1 −1 0 1 2 −1
ISR1 −2 −1 −2 −1 −4 4 5 5 2 −1 0 −1 −1 3 1 1
BNS1 1 −4 −1 2 −8 10 0 10 1 0 0 0 −1 2 6 2
YPR114W 2 −3 −2 −1 −7 9 9 2 −1 0 0 1 −2 3 2 9
RBG1 −1 1 5 −7 −5 6 7 7 1 −2 1 −2 −3 2 0 2
SEM1 −6 −1 3 −4 −5 5 3 6 2 −1 −1 0 −2 3 0 2
YMR178W −1 −1 2 −3 −7 3 5 5 1 0 0 −1 1 7 −1 0
SNF7 −1 −1 2 −3 −9 9 7 4 1 −1 0 0 3 −1 −2 3
CYB2 −5 3 3 −2 −6 9 6 6 2 −1 0 −1 3 −3 −4 −2
YCR102C −3 −2 −1 −3 −9 5 6 7 −2 −1 0 0 −2 1 −3 −3
MSH3 −5 0 1 0 −7 6 5 5 −1 −1 0 0 −1 −4 −3 −3
PSK1 0 −4 −4 −6 −8 9 5 5 1 0 2 0 1 1 −2 3
IMD4 −4 −2 −1 −6 −7 4 6 2 2 −1 0 −1 1 2 −3 2
MSS11 −5 −6 −2 −7 −10 6 5 5 3 0 0 −1 3 2 0 0
NTG2 −5 −5 −3 −1 −9 4 6 5 0 1 2 2 3 −2 1 0
ERV14 −7 −3 −3 −3 −11 6 7 6 1 −2 0 −1 1 0 −3 −2
OGG1 −13 6 8 −2 −9 9 8 6 1 −1 0 0 −1 −2 −2 2
RPL24A −11 −2 4 0 −6 6 7 6 2 −1 −1 −1 −3 −4 −3 −4
SGF11 −13 −4 3 −2 −10 4 6 6 2 −2 0 −2 1 −1 −1 −1
SWC3 −1 1 −1 −18 −5 10 7 1 2 1 2 0 0 3 1 7
SUR7 −3 2 3 −16 −6 9 8 −2 1 0 0 0 −1 0 −1 6
YMR147W −9 1 2 −21 −9 6 2 2 1 −2 1 −1 −5 2 2 5
YPR146C −4 −1 2 −11 −13 9 9 4 3 −1 1 2 5 1 4 6
DNF3 −4 −1 1 −5 −9 8 5 4 0 −1 1 0 −2 1 1 6
CPR3 −7 −2 2 −8 −9 5 3 2 0 1 1 0 0 2 4 3
YMR153C−A −9 1 −2 −10 −14 5 7 7 1 0 1 0 3 4 −3 1
YAL027W −6 3 0 −15 −9 10 2 4 2 −1 0 0 0 −2 0 1
YAL046C −8 −1 0 −8 −11 11 5 7 1 −1 0 −1 3 −6 −3 −3
Gene Name
Type of Media

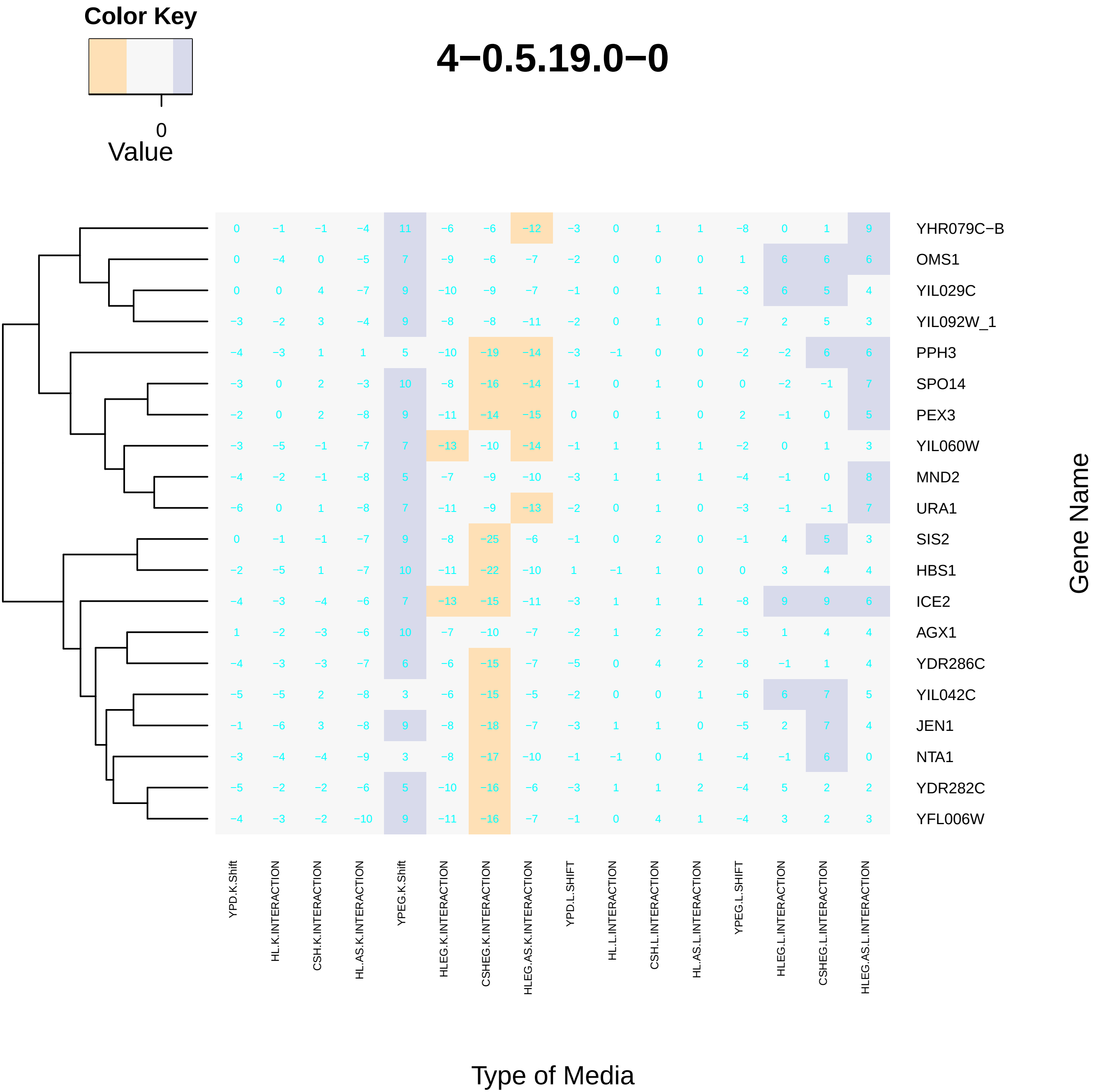
Color Key
0
Value
4−0.5.19.0−0
YHR079C−B: 0 −1 −1 −4 11 −6 −6 −12 −3 0 1 1 −8 0 1 9
OMS1: 0 −4 0 −5 7 −9 −6 −7 −2 0 0 0 1 6 6 6
YIL029C: 0 0 4 −7 9 −10 −9 −7 −1 0 1 1 −3 6 5 4
YIL092W_1: −3 −2 3 −4 9 −8 −8 −11 −2 0 1 0 −7 2 5 3
PPH3: −4 −3 1 1 5 −10 −19 −14 −3 −1 0 0 −2 −2 6 6
SPO14: −3 0 2 −3 10 −8 −16 −14 −1 0 1 0 0 −2 −1 7
PEX3: −2 0 2 −8 9 −11 −14 −15 0 0 1 0 2 −1 0 5
YIL060W: −3 −5 −1 −7 7 −13 −10 −14 −1 1 1 1 −2 0 1 3
MND2: −4 −2 −1 −8 5 −7 −9 −10 −3 1 1 1 −4 −1 0 8
URA1: −6 0 1 −8 7 −11 −9 −13 −2 0 1 0 −3 −1 −1 7
SIS2: 0 −1 −1 −7 9 −8 −25 −6 −1 0 2 0 −1 4 5 3
HBS1: −2 −5 1 −7 10 −11 −22 −10 1 −1 1 0 0 3 4 4
ICE2: −4 −3 −4 −6 7 −13 −15 −11 −3 1 1 1 −8 9 9 6
AGX1: 1 −2 −3 −6 10 −7 −10 −7 −2 1 2 2 −5 1 4 4
YDR286C: −4 −3 −3 −7 6 −6 −15 −7 −5 0 4 2 −8 −1 1 4
YIL042C: −5 −5 2 −8 3 −6 −15 −5 −2 0 0 1 −6 6 7 5
JEN1: −1 −6 3 −8 9 −8 −18 −7 −3 1 1 0 −5 2 7 4
NTA1: −3 −4 −4 −9 3 −8 −17 −10 −1 −1 0 1 −4 −1 6 0
YDR282C: −5 −2 −2 −6 5 −10 −16 −6 −3 1 1 2 −4 5 2 2
YFL006W: −4 −3 −2 −10 9 −11 −16 −7 −1 0 4 1 −4 3 2 3
YPD.K.Shift
HL.K.INTERACTION
CSH.K.INTERACTION
HL.AS.K.INTERACTION
YPEG.K.Shift
HLEG.K.INTERACTION
CSHEG.K.INTERACTION
HLEG.AS.K.INTERACTION
YPD.L.SHIFT
HL.L.INTERACTION
CSH.L.INTERACTION
HL.AS.L.INTERACTION
YPEG.L.SHIFT
HLEG.L.INTERACTION
CSHEG.L.INTERACTION
HLEG.AS.L.INTERACTION
Gene Name
Type of Media

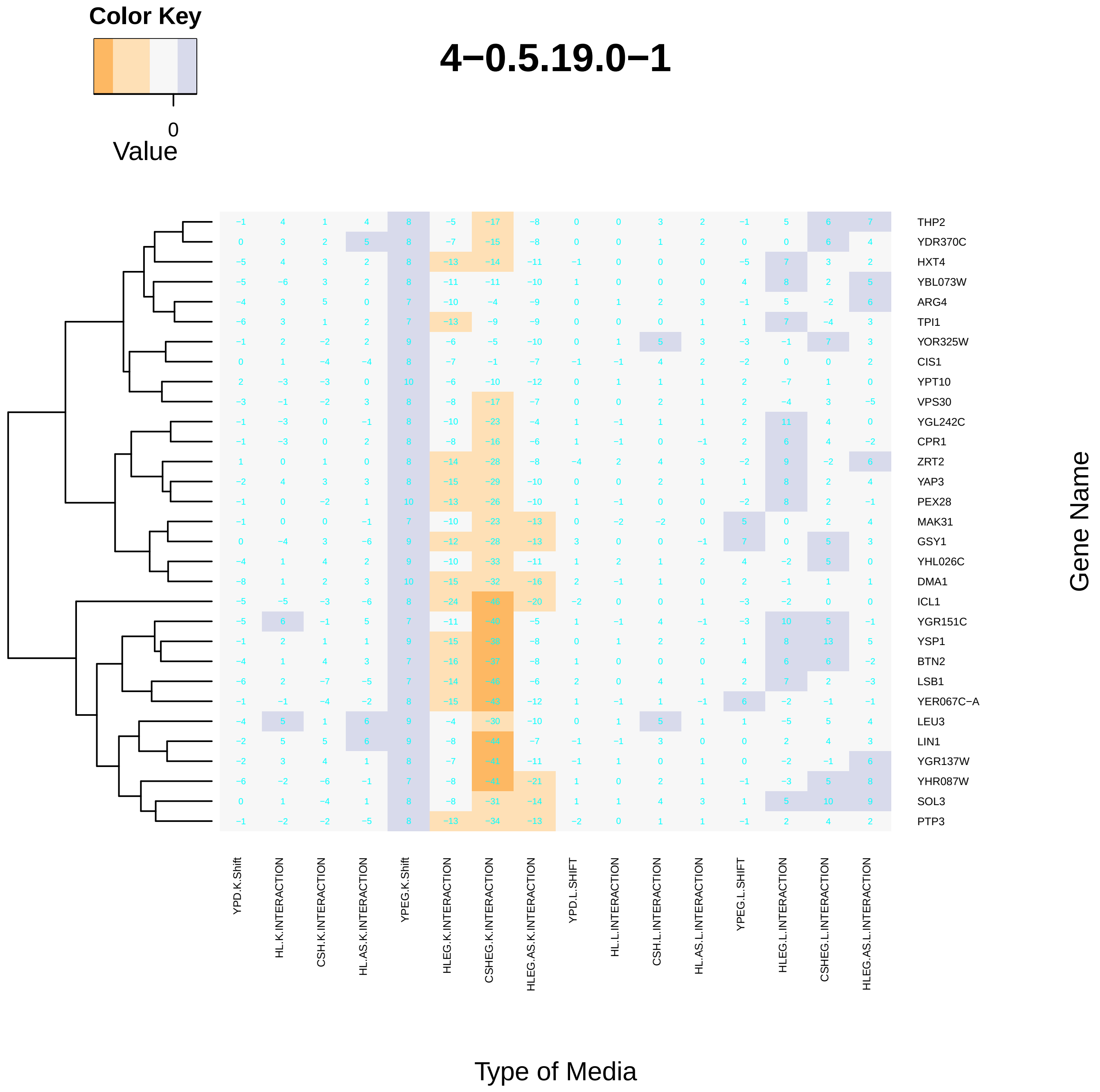
Color Key
0
Value
4−0.5.19.0−1
YPD.K.Shift HL.K.INTERACTION CSH.K.INTERACTION HL.AS.K.INTERACTION YPEG.K.Shift HLEG.K.INTERACTION CSHEG.K.INTERACTION HLEG.AS.K.INTERACTION YPD.L.SHIFT HL.L.INTERACTION CSH.L.INTERACTION HL.AS.L.INTERACTION YPEG.L.SHIFT HLEG.L.INTERACTION CSHEG.L.INTERACTION HLEG.AS.L.INTERACTION
THP2 −1 4 1 4 8 −5 −17 −8 0 0 3 2 −1 5 6 7
YDR370C 0 3 2 5 8 −7 −15 −8 0 0 1 2 0 0 6 4
HXT4 −5 4 3 2 8 −13 −14 −11 −1 0 0 0 −5 7 3 2
YBL073W −5 −6 3 2 8 −11 −11 −10 1 0 0 0 4 8 2 5
ARG4 −4 3 5 0 7 −10 −4 −9 0 1 2 3 −1 5 −2 6
TPI1 −6 3 1 2 7 −13 −9 −9 0 0 0 1 1 7 −4 3
YOR325W −1 2 −2 2 9 −6 −5 −10 0 1 5 3 −3 −1 7 3
CIS1 0 1 −4 −4 8 −7 −1 −7 −1 −1 4 2 −2 0 0 2
YPT10 2 −3 −3 0 10 −6 −10 −12 0 1 1 1 2 −7 1 0
VPS30 −3 −1 −2 3 8 −8 −17 −7 0 0 2 1 2 −4 3 −5
YGL242C −1 −3 0 −1 8 −10 −23 −4 1 −1 1 1 2 11 4 0
CPR1 −1 −3 0 2 8 −8 −16 −6 1 −1 0 −1 2 6 4 −2
ZRT2 1 0 1 0 8 −14 −28 −8 −4 2 4 3 −2 9 −2 6
YAP3 −2 4 3 3 8 −15 −29 −10 0 0 2 1 1 8 2 4
PEX28 −1 0 −2 1 10 −13 −26 −10 1 −1 0 0 −2 8 2 −1
MAK31 −1 0 0 −1 7 −10 −23 −13 0 −2 −2 0 5 0 2 4
GSY1 0 −4 3 −6 9 −12 −28 −13 3 0 0 −1 7 0 5 3
YHL026C −4 1 4 2 9 −10 −33 −11 1 2 1 2 4 −2 5 0
DMA1 −8 1 2 3 10 −15 −32 −16 2 −1 1 0 2 −1 1 1
ICL1 −5 −5 −3 −6 8 −24 −46 −20 −2 0 0 1 −3 −2 0 0
YGR151C −5 6 −1 5 7 −11 −40 −5 1 −1 4 −1 −3 10 5 −1
YSP1 −1 2 1 1 9 −15 −38 −8 0 1 2 2 1 8 13 5
BTN2 −4 1 4 3 7 −16 −37 −8 1 0 0 0 4 6 6 −2
LSB1 −6 2 −7 −5 7 −14 −46 −6 2 0 4 1 2 7 2 −3
YER067C−A −1 −1 −4 −2 8 −15 −43 −12 1 −1 1 −1 6 −2 −1 −1
LEU3 −4 5 1 6 9 −4 −30 −10 0 1 5 1 1 −5 5 4
LIN1 −2 5 5 6 9 −8 −44 −7 −1 −1 3 0 0 2 4 3
YGR137W −2 3 4 1 8 −7 −41 −11 −1 1 0 1 0 −2 −1 6
YHR087W −6 −2 −6 −1 7 −8 −41 −21 1 0 2 1 −1 −3 5 8
SOL3 0 1 −4 1 8 −8 −31 −14 1 1 4 3 1 5 10 9
PTP3 −1 −2 −2 −5 8 −13 −34 −13 −2 0 1 1 −1 2 4 2
Gene Name
Type of Media

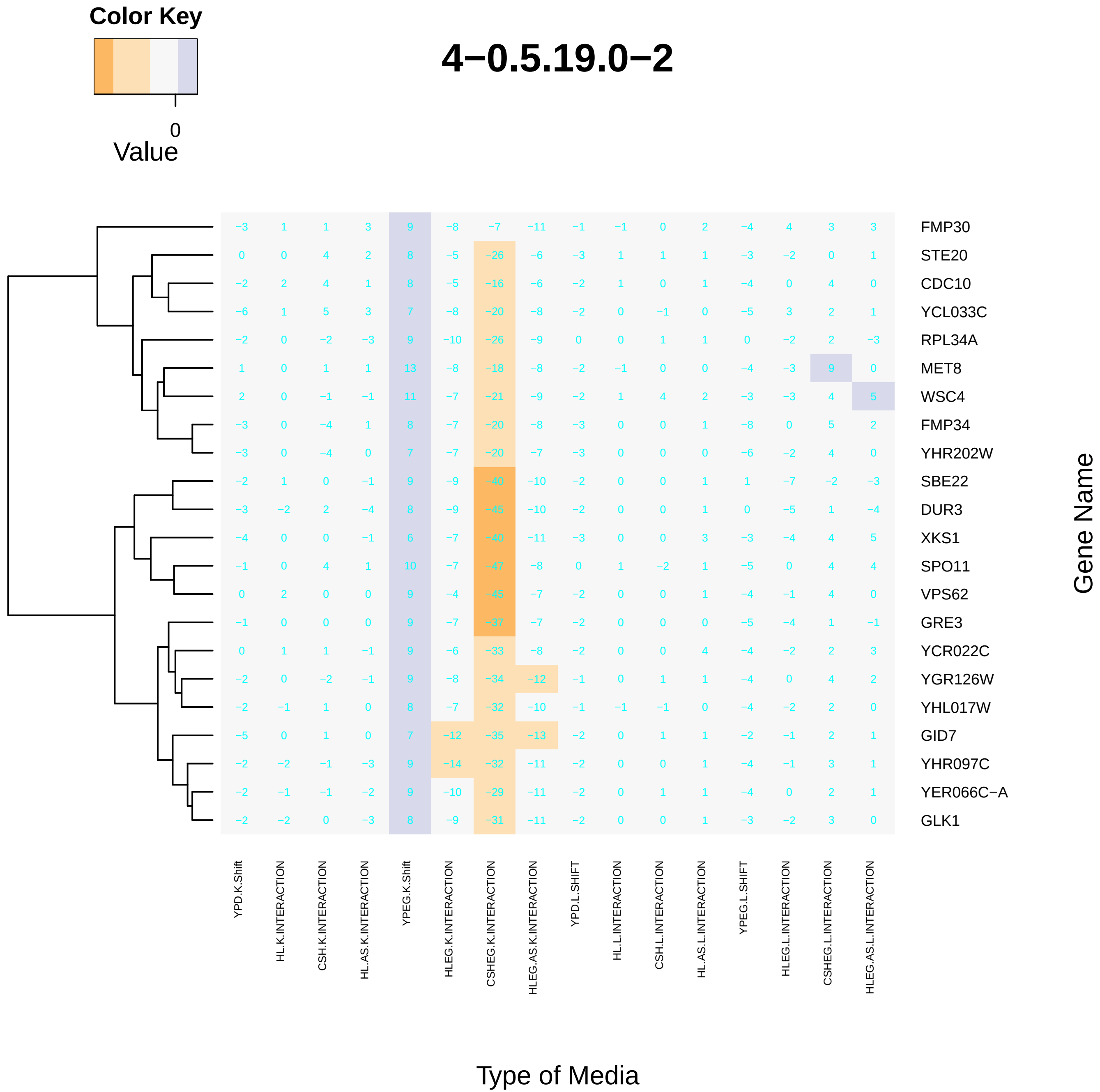
Color Key
0
Value
4−0.5.19.0−2
FMP30 −3 1 1 3 9 −8 −7 −11 −1 −1 0 2 −4 4 3 3
STE20 0 0 4 2 8 −5 −26 −6 −3 1 1 1 −3 −2 0 1
CDC10 −2 2 4 1 8 −5 −16 −6 −2 1 0 1 −4 0 4 0
YCL033C −6 1 5 3 7 −8 −20 −8 −2 0 −1 0 −5 3 2 1
RPL34A −2 0 −2 −3 9 −10 −26 −9 0 0 1 1 0 −2 2 −3
MET8 1 0 1 1 13 −8 −18 −8 −2 −1 0 0 −4 −3 9 0
WSC4 2 0 −1 −1 11 −7 −21 −9 −2 1 4 2 −3 −3 4 5
FMP34 −3 0 −4 1 8 −7 −20 −8 −3 0 0 1 −8 0 5 2
YHR202W −3 0 −4 0 7 −7 −20 −7 −3 0 0 0 −6 −2 4 0
SBE22 −2 1 0 −1 9 −9 −40 −10 −2 0 0 1 1 −7 −2 −3
DUR3 −3 −2 2 −4 8 −9 −45 −10 −2 0 0 1 0 −5 1 −4
XKS1 −4 0 0 −1 6 −7 −40 −11 −3 0 0 3 −3 −4 4 5
SPO11 −1 0 4 1 10 −7 −47 −8 0 1 −2 1 −5 0 4 4
VPS62 0 2 0 0 9 −4 −45 −7 −2 0 0 1 −4 −1 4 0
GRE3 −1 0 0 0 9 −7 −37 −7 −2 0 0 0 −5 −4 1 −1
YCR022C 0 1 1 −1 9 −6 −33 −8 −2 0 0 4 −4 −2 2 3
YGR126W −2 0 −2 −1 9 −8 −34 −12 −1 0 1 1 −4 0 4 2
YHL017W −2 −1 1 0 8 −7 −32 −10 −1 −1 −1 0 −4 −2 2 0
GID7 −5 0 1 0 7 −12 −35 −13 −2 0 1 1 −2 −1 2 1
YHR097C −2 −2 −1 −3 9 −14 −32 −11 −2 0 0 1 −4 −1 3 1
YER066C−A −2 −1 −1 −2 9 −10 −29 −11 −2 0 1 1 −4 0 2 1
GLK1 −2 −2 0 −3 8 −9 −31 −11 −2 0 0 1 −3 −2 3 0
YPD.K.Shift
HL.K.INTERACTION
CSH.K.INTERACTION
HL.AS.K.INTERACTION
YPEG.K.Shift
HLEG.K.INTERACTION
CSHEG.K.INTERACTION
HLEG.AS.K.INTERACTION
YPD.L.SHIFT
HL.L.INTERACTION
CSH.L.INTERACTION
HL.AS.L.INTERACTION
YPEG.L.SHIFT
HLEG.L.INTERACTION
CSHEG.L.INTERACTION
HLEG.AS.L.INTERACTION
Gene Name
Type of Media

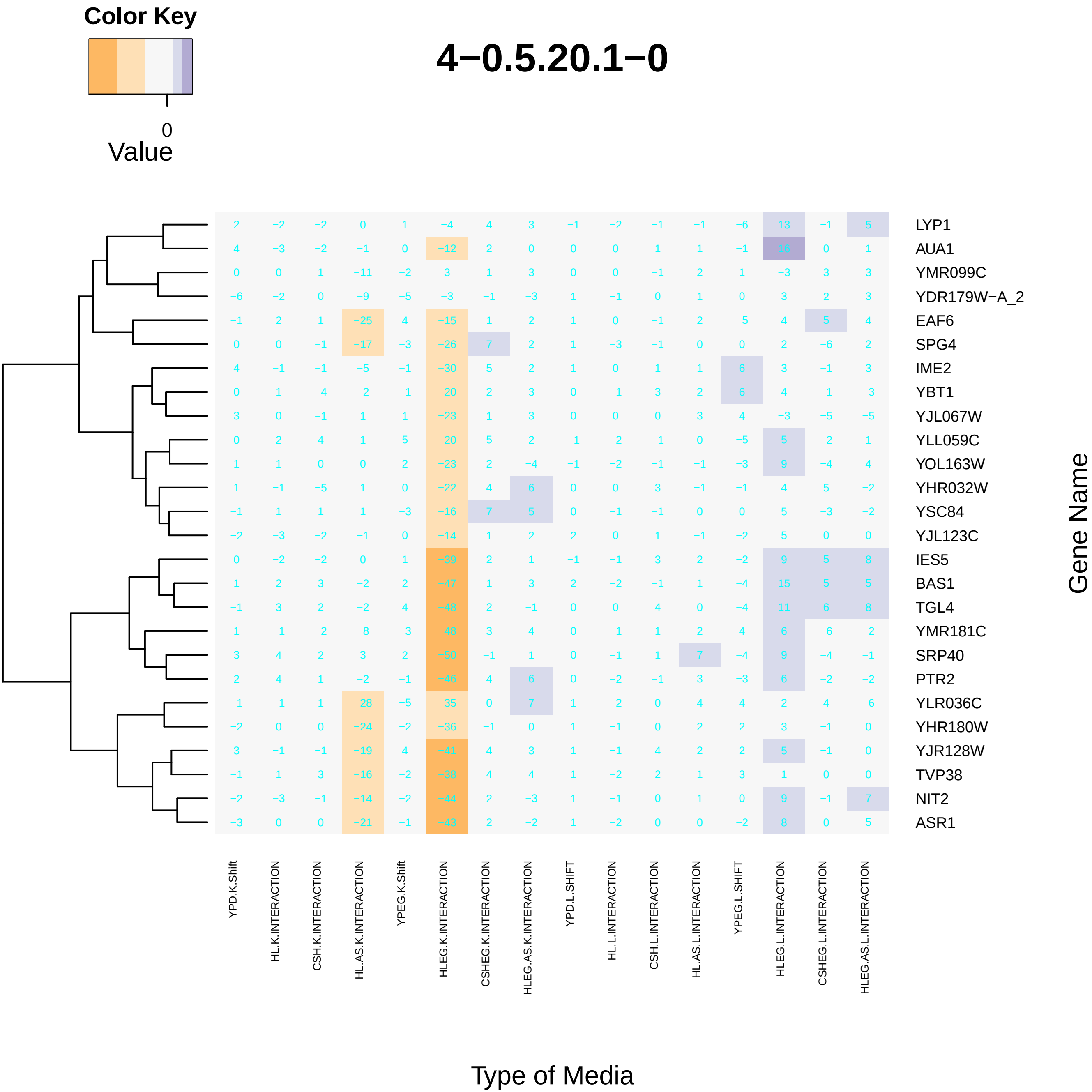
Color Key
0
Value
4−0.5.20.1−0
LYP1 2 −2 −2 0 1 −4 4 3 −1 −2 −1 −1 −6 13 −1 5
AUA1 4 −3 −2 −1 0 −12 2 0 0 0 1 1 −1 16 0 1
YMR099C 0 0 1 −11 −2 3 1 3 0 0 −1 2 1 −3 3 3
YDR179W−A_2 −6 −2 0 −9 −5 −3 −1 −3 1 −1 0 1 0 3 2 3
EAF6 −1 2 1 −25 4 −15 1 2 1 0 −1 2 −5 4 5 4
SPG4 0 0 −1 −17 −3 −26 7 2 1 −3 −1 0 0 2 −6 2
IME2 4 −1 −1 −5 −1 −30 5 2 1 0 1 1 6 3 −1 3
YBT1 0 1 −4 −2 −1 −20 2 3 0 −1 3 2 6 4 −1 −3
YJL067W 3 0 −1 1 1 −23 1 3 0 0 0 3 4 −3 −5 −5
YLL059C 0 2 4 1 5 −20 5 2 −1 −2 −1 0 −5 5 −2 1
YOL163W 1 1 0 0 2 −23 2 −4 −1 −2 −1 −1 −3 9 −4 4
YHR032W 1 −1 −5 1 0 −22 4 6 0 0 3 −1 −1 4 5 −2
YSC84 −1 1 1 1 −3 −16 7 5 0 −1 −1 0 0 5 −3 −2
YJL123C −2 −3 −2 −1 0 −14 1 2 2 0 1 −1 −2 5 0 0
IES5 0 −2 −2 0 1 −39 2 1 −1 −1 3 2 −2 9 5 8
BAS1 1 2 3 −2 2 −47 1 3 2 −2 −1 1 −4 15 5 5
TGL4 −1 3 2 −2 4 −48 2 −1 0 0 4 0 −4 11 6 8
YMR181C 1 −1 −2 −8 −3 −48 3 4 0 −1 1 2 4 6 −6 −2
SRP40 3 4 2 3 2 −50 −1 1 0 −1 1 7 −4 9 −4 −1
PTR2 2 4 1 −2 −1 −46 4 6 0 −2 −1 3 −3 6 −2 −2
YLR036C −1 −1 1 −28 −5 −35 0 7 1 −2 0 4 4 2 4 −6
YHR180W −2 0 0 −24 −2 −36 −1 0 1 −1 0 2 2 3 −1 0
YJR128W 3 −1 −1 −19 4 −41 4 3 1 −1 4 2 2 5 −1 0
TVP38 −1 1 3 −16 −2 −38 4 4 1 −2 2 1 3 1 0 0
NIT2 −2 −3 −1 −14 −2 −44 2 −3 1 −1 0 1 0 9 −1 7
ASR1 −3 0 0 −21 −1 −43 2 −2 1 −2 0 0 −2 8 0 5
Gene Name
YPD.K.Shift
HL.K.INTERACTION
CSH.K.INTERACTION
HL.AS.K.INTERACTION
YPEG.K.Shift
HLEG.K.INTERACTION
CSHEG.K.INTERACTION
HLEG.AS.K.INTERACTION
YPD.L.SHIFT
HL.L.INTERACTION
CSH.L.INTERACTION
HL.AS.L.INTERACTION
YPEG.L.SHIFT
HLEG.L.INTERACTION
CSHEG.L.INTERACTION
HLEG.AS.L.INTERACTION
Type of Media

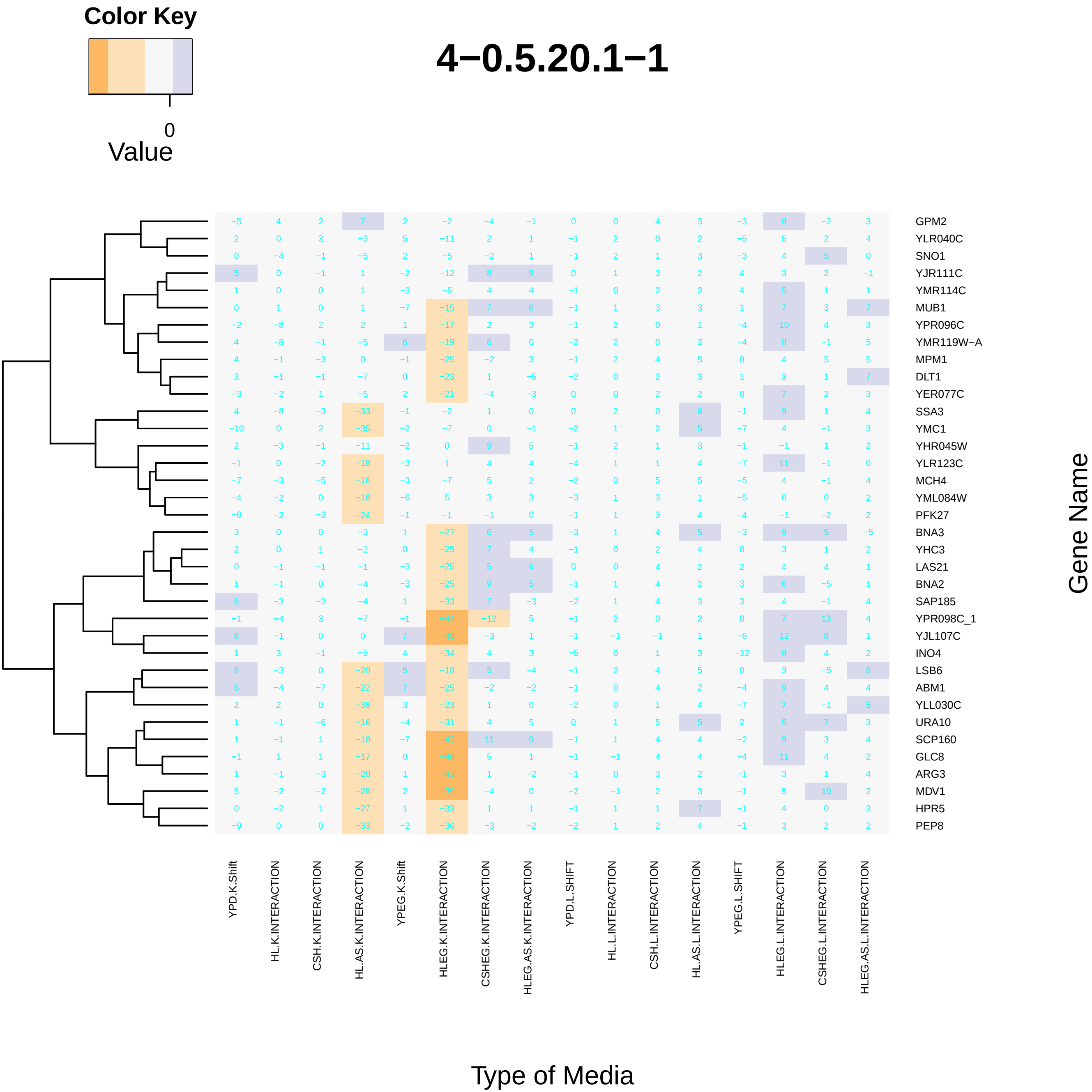
Color Key
0
Value
4−0.5.20.1−1
GPM2
YLR040C
SNO1
YJR111C
YMR114C
MUB1
YPR096C
YMR119W−A
MPM1
DLT1
YER077C
SSA3
YMC1
YHR045W
YLR123C
MCH4
YML084W
PFK27
BNA3
YHC3
LAS21
BNA2
SAP185
YPR098C_1
YJL107C
INO4
LSB6
ABM1
YLL030C
URA10
SCP160
GLC8
ARG3
MDV1
HPR5
PEP8
Gene Name
YPD.K.Shift
HL.K.INTERACTION
CSH.K.INTERACTION
HL.AS.K.INTERACTION
YPEG.K.Shift
HLEG.K.INTERACTION
CSHEG.K.INTERACTION
HLEG.AS.K.INTERACTION
YPD.L.SHIFT
HL.L.INTERACTION
CSH.L.INTERACTION
HL.AS.L.INTERACTION
YPEG.L.SHIFT
HLEG.L.INTERACTION
CSHEG.L.INTERACTION
HLEG.AS.L.INTERACTION
Type of Media

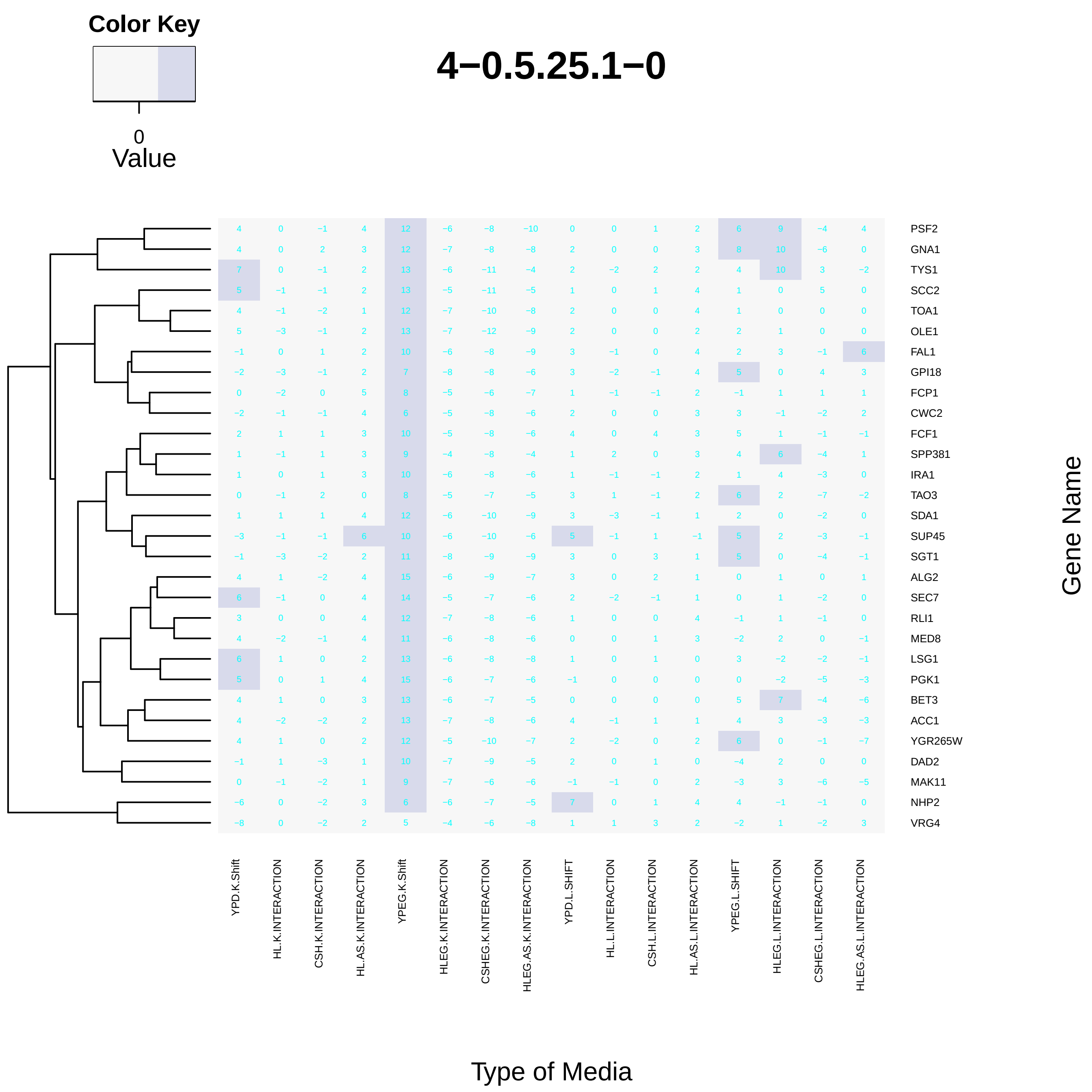
Color Key
0
Value
4−0.5.25.1−0
PSF2 4 0 −1 4 12 −6 −8 −10 0 0 1 2 6 9 −4 4
GNA1 4 0 2 3 12 −7 −8 −8 2 0 0 3 8 10 −6 0
TYS1 7 0 −1 2 13 −6 −11 −4 2 −2 2 2 4 10 3 −2
SCC2 5 −1 −1 2 13 −5 −11 −5 1 0 1 4 1 0 5 0
TOA1 4 −1 −2 1 12 −7 −10 −8 2 0 0 4 1 0 0 0
OLE1 5 −3 −1 2 13 −7 −12 −9 2 0 0 2 2 1 0 0
FAL1 −1 0 1 2 10 −6 −8 −9 3 −1 0 4 2 3 −1 6
GPI18 −2 −3 −1 2 7 −8 −8 −6 3 −2 −1 4 5 0 4 3
FCP1 0 −2 0 5 8 −5 −6 −7 1 −1 −1 2 −1 1 1 1
CWC2 −2 −1 −1 4 6 −5 −8 −6 2 0 0 3 3 −1 −2 2
FCF1 2 1 1 3 10 −5 −8 −6 4 0 4 3 5 1 −1 −1
SPP381 1 −1 1 3 9 −4 −8 −4 1 2 0 3 4 6 −4 1
IRA1 1 0 1 3 10 −6 −8 −6 1 −1 −1 2 1 4 −3 0
TAO3 0 −1 2 0 8 −5 −7 −5 3 1 −1 2 6 2 −7 −2
SDA1 1 1 1 4 12 −6 −10 −9 3 −3 −1 1 2 0 −2 0
SUP45 −3 −1 −1 6 10 −6 −10 −6 5 −1 1 −1 5 2 −3 −1
SGT1 −1 −3 −2 2 11 −8 −9 −9 3 0 3 1 5 0 −4 −1
ALG2 4 1 −2 4 15 −6 −9 −7 3 0 2 1 0 1 0 1
SEC7 6 −1 0 4 14 −5 −7 −6 2 −2 −1 1 0 1 −2 0
RLI1 3 0 0 4 12 −7 −8 −6 1 0 0 4 −1 1 −1 0
MED8 4 −2 −1 4 11 −6 −8 −6 0 0 1 3 −2 2 0 −1
LSG1 6 1 0 2 13 −6 −8 −8 1 0 1 0 3 −2 −2 −1
PGK1 5 0 1 4 15 −6 −7 −6 −1 0 0 0 0 −2 −5 −3
BET3 4 1 0 3 13 −6 −7 −5 0 0 0 0 5 7 −4 −6
ACC1 4 −2 −2 2 13 −7 −8 −6 4 −1 1 1 4 3 −3 −3
YGR265W 4 1 0 2 12 −5 −10 −7 2 −2 0 2 6 0 −1 −7
DAD2 −1 1 −3 1 10 −7 −9 −5 2 0 1 0 −4 2 0 0
MAK11 0 −1 −2 1 9 −7 −6 −6 −1 −1 0 2 −3 3 −6 −5
NHP2 −6 0 −2 3 6 −6 −7 −5 7 0 1 4 4 −1 −1 0
VRG4 −8 0 −2 2 5 −4 −6 −8 1 1 3 2 −2 1 −2 3
YPD.K.Shift
HL.K.INTERACTION
CSH.K.INTERACTION
HL.AS.K.INTERACTION
YPEG.K.Shift
HLEG.K.INTERACTION
CSHEG.K.INTERACTION
HLEG.AS.K.INTERACTION
YPD.L.SHIFT
HL.L.INTERACTION
CSH.L.INTERACTION
HL.AS.L.INTERACTION
YPEG.L.SHIFT
HLEG.L.INTERACTION
CSHEG.L.INTERACTION
HLEG.AS.L.INTERACTION
Gene Name
Type of Media

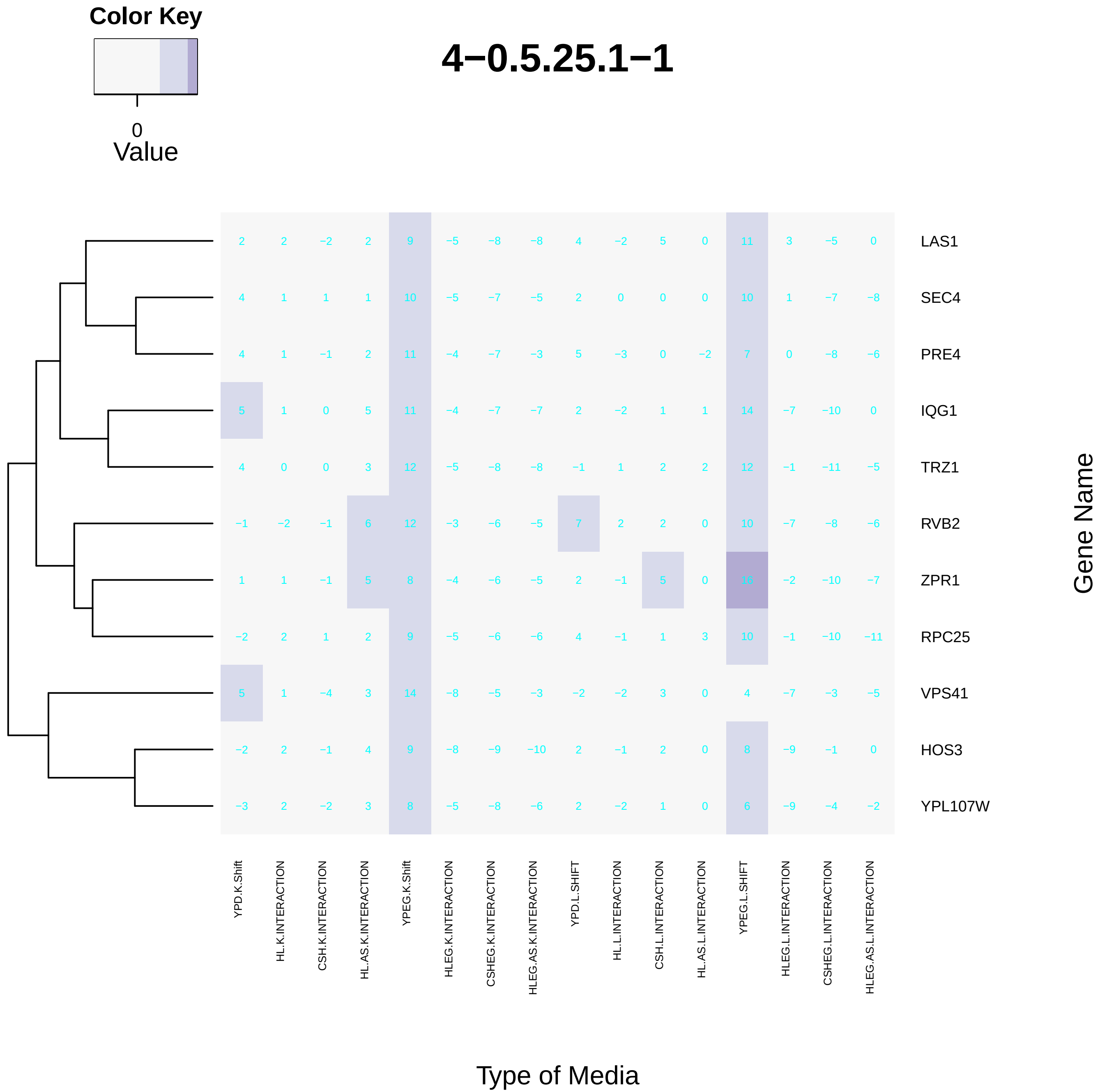

Color Key
0
Value
4−0.5.25.1−1
LAS1 2 2 −2 2 9 −5 −8 −8 4 −2 5 0 11 3 −5 0
SEC4 4 1 1 1 10 −5 −7 −5 2 0 0 0 10 1 −7 −8
PRE4 4 1 −1 2 11 −4 −7 −3 5 −3 0 −2 7 0 −8 −6
IQG1 5 1 0 5 11 −4 −7 −7 2 −2 1 1 14 −7 −10 0
TRZ1 4 0 0 3 12 −5 −8 −8 −1 1 2 2 12 −1 −11 −5
RVB2 −1 −2 −1 6 12 −3 −6 −5 7 2 2 0 10 −7 −8 −6
ZPR1 1 1 −1 5 8 −4 −6 −5 2 −1 5 0 16 −2 −10 −7
RPC25 −2 2 1 2 9 −5 −6 −6 4 −1 1 3 10 −1 −10 −11
VPS41 5 1 −4 3 14 −8 −5 −3 −2 −2 3 0 4 −7 −3 −5
HOS3 −2 2 −1 4 9 −8 −9 −10 2 −1 2 0 8 −9 −1 0
YPL107W −3 2 −2 3 8 −5 −8 −6 2 −2 1 0 6 −9 −4 −2
YPD.K.Shift
HL.K.INTERACTION
CSH.K.INTERACTION
HL.AS.K.INTERACTION
YPEG.K.Shift
HLEG.K.INTERACTION
CSHEG.K.INTERACTION
HLEG.AS.K.INTERACTION
YPD.L.SHIFT
HL.L.INTERACTION
CSH.L.INTERACTION
HL.AS.L.INTERACTION
YPEG.L.SHIFT
HLEG.L.INTERACTION
CSHEG.L.INTERACTION
HLEG.AS.L.INTERACTION
Gene Name
Type of Media

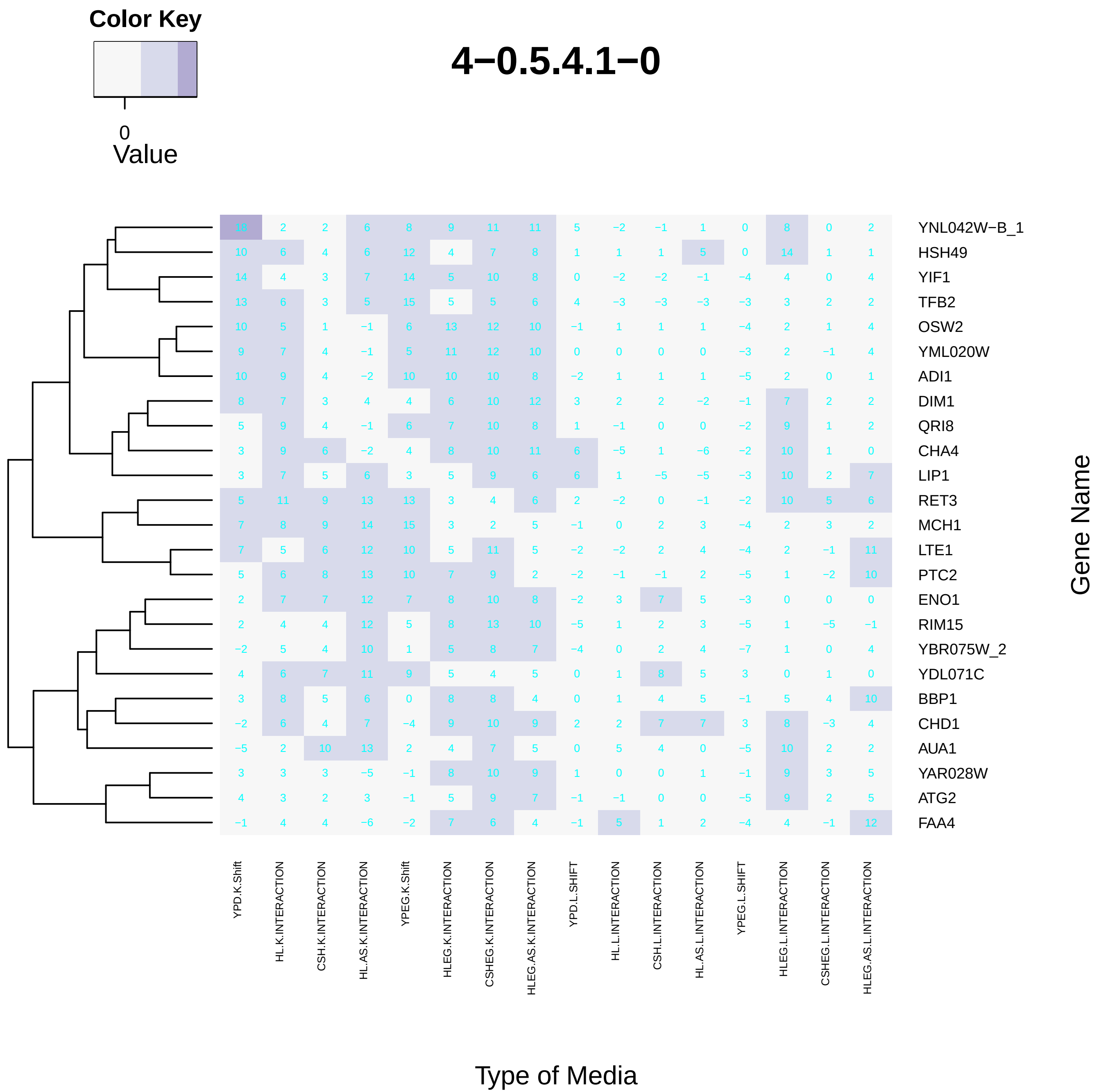
Color Key
0
Value
4−0.5.4.1−0
YNL042W−B_1
HSH49
YIF1
TFB2
OSW2
YML020W
ADI1
DIM1
QRI8
CHA4
LIP1
RET3
MCH1
LTE1
PTC2
ENO1
RIM15
YBR075W_2
YDL071C
BBP1
CHD1
AUA1
YAR028W
ATG2
FAA4
Gene Name
YPD.K.Shift
HL.K.INTERACTION
CSH.K.INTERACTION
HL.AS.K.INTERACTION
YPEG.K.Shift
HLEG.K.INTERACTION
CSHEG.K.INTERACTION
HLEG.AS.K.INTERACTION
YPD.L.SHIFT
HL.L.INTERACTION
CSH.L.INTERACTION
HL.AS.L.INTERACTION
YPEG.L.SHIFT
HLEG.L.INTERACTION
CSHEG.L.INTERACTION
HLEG.AS.L.INTERACTION
Type of Media

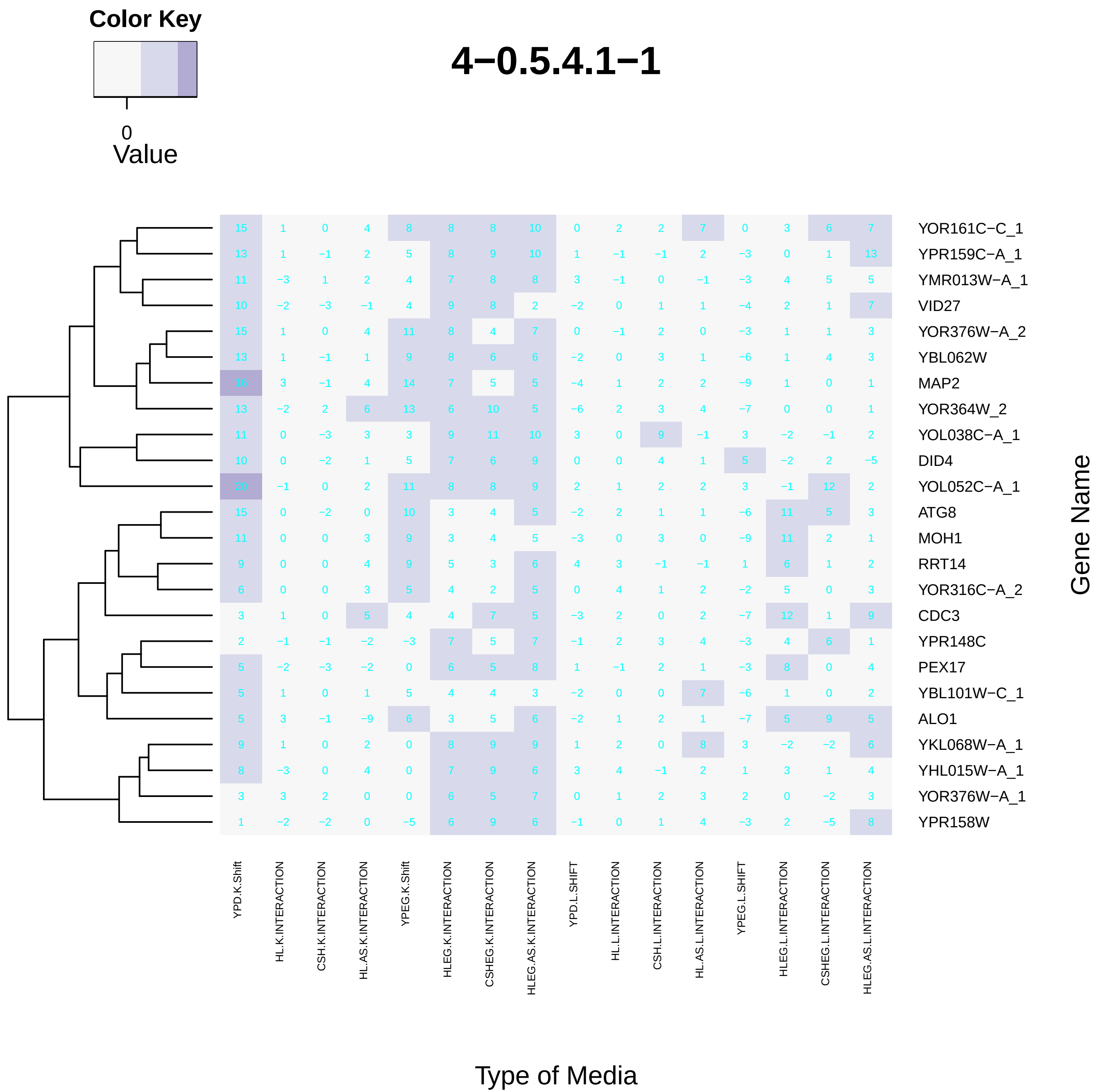

Color Key
0
Value
4−0.5.4.1−1
YOR161C−C_1
YPR159C−A_1
YMR013W−A_1
VID27
YOR376W−A_2
YBL062W
MAP2
YOR364W_2
YOL038C−A_1
DID4
YOL052C−A_1
ATG8
MOH1
RRT14
YOR316C−A_2
CDC3
YPR148C
PEX17
YBL101W−C_1
ALO1
YKL068W−A_1
YHL015W−A_1
YOR376W−A_1
YPR158W
Gene Name
YPD.K.Shift
HL.K.INTERACTION
CSH.K.INTERACTION
HL.AS.K.INTERACTION
YPEG.K.Shift
HLEG.K.INTERACTION
CSHEG.K.INTERACTION
HLEG.AS.K.INTERACTION
YPD.L.SHIFT
HL.L.INTERACTION
CSH.L.INTERACTION
HL.AS.L.INTERACTION
YPEG.L.SHIFT
HLEG.L.INTERACTION
CSHEG.L.INTERACTION
HLEG.AS.L.INTERACTION
Type of Media

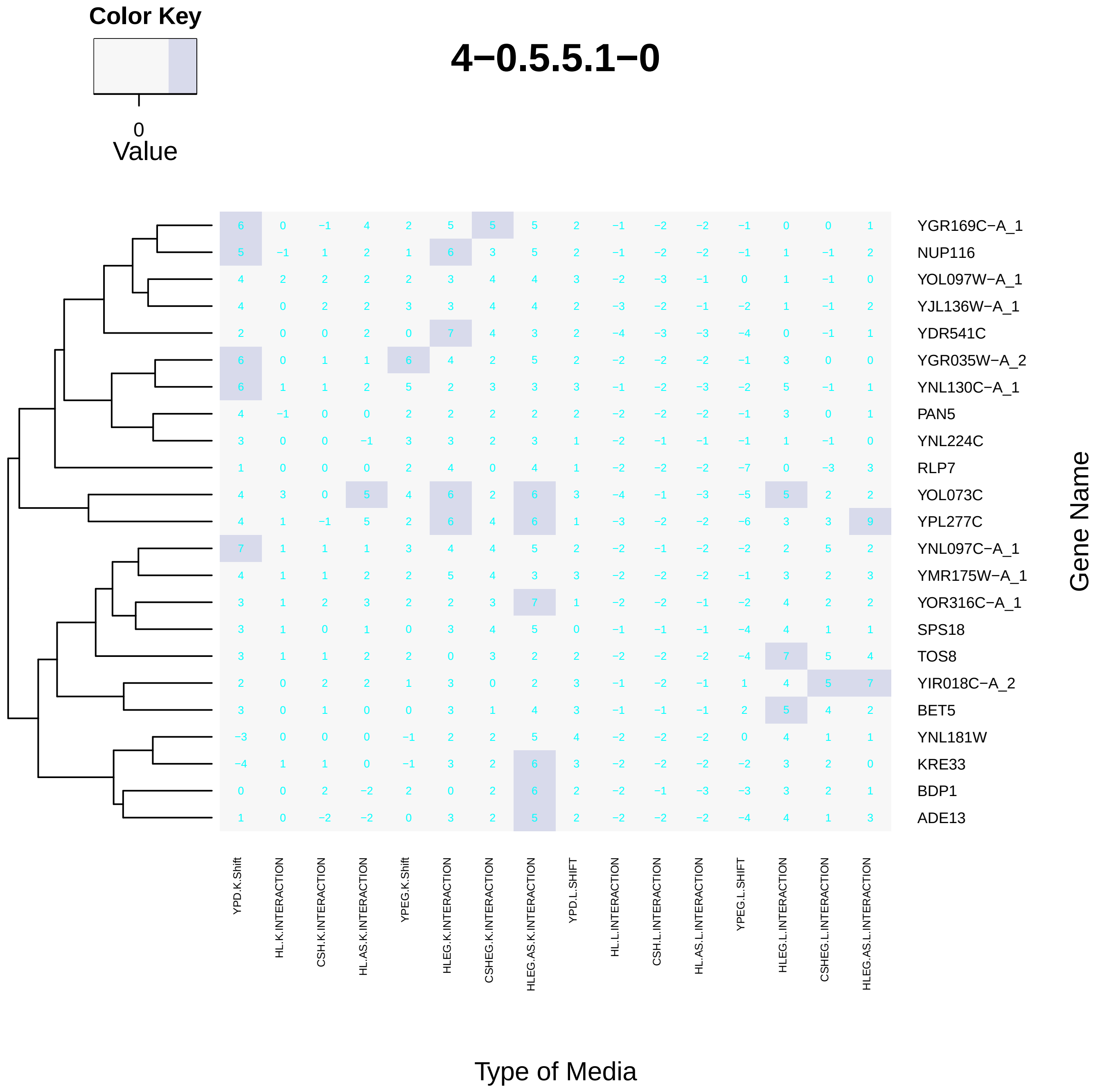
Color Key
0
Value
4−0.5.5.1−0
YGR169C−A_1
NUP116
YOL097W−A_1
YJL136W−A_1
YDR541C
YGR035W−A_2
YNL130C−A_1
PAN5
YNL224C
RLP7
YOL073C
YPL277C
YNL097C−A_1
YMR175W−A_1
YOR316C−A_1
SPS18
TOS8
YIR018C−A_2
BET5
YNL181W
KRE33
BDP1
ADE13
YPD.K.Shift
HL.K.INTERACTION
CSH.K.INTERACTION
HL.AS.K.INTERACTION
YPEG.K.Shift
HLEG.K.INTERACTION
CSHEG.K.INTERACTION
HLEG.AS.K.INTERACTION
YPD.L.SHIFT
HL.L.INTERACTION
CSH.L.INTERACTION
HL.AS.L.INTERACTION
YPEG.L.SHIFT
HLEG.L.INTERACTION
CSHEG.L.INTERACTION
HLEG.AS.L.INTERACTION
Gene Name
Type of Media

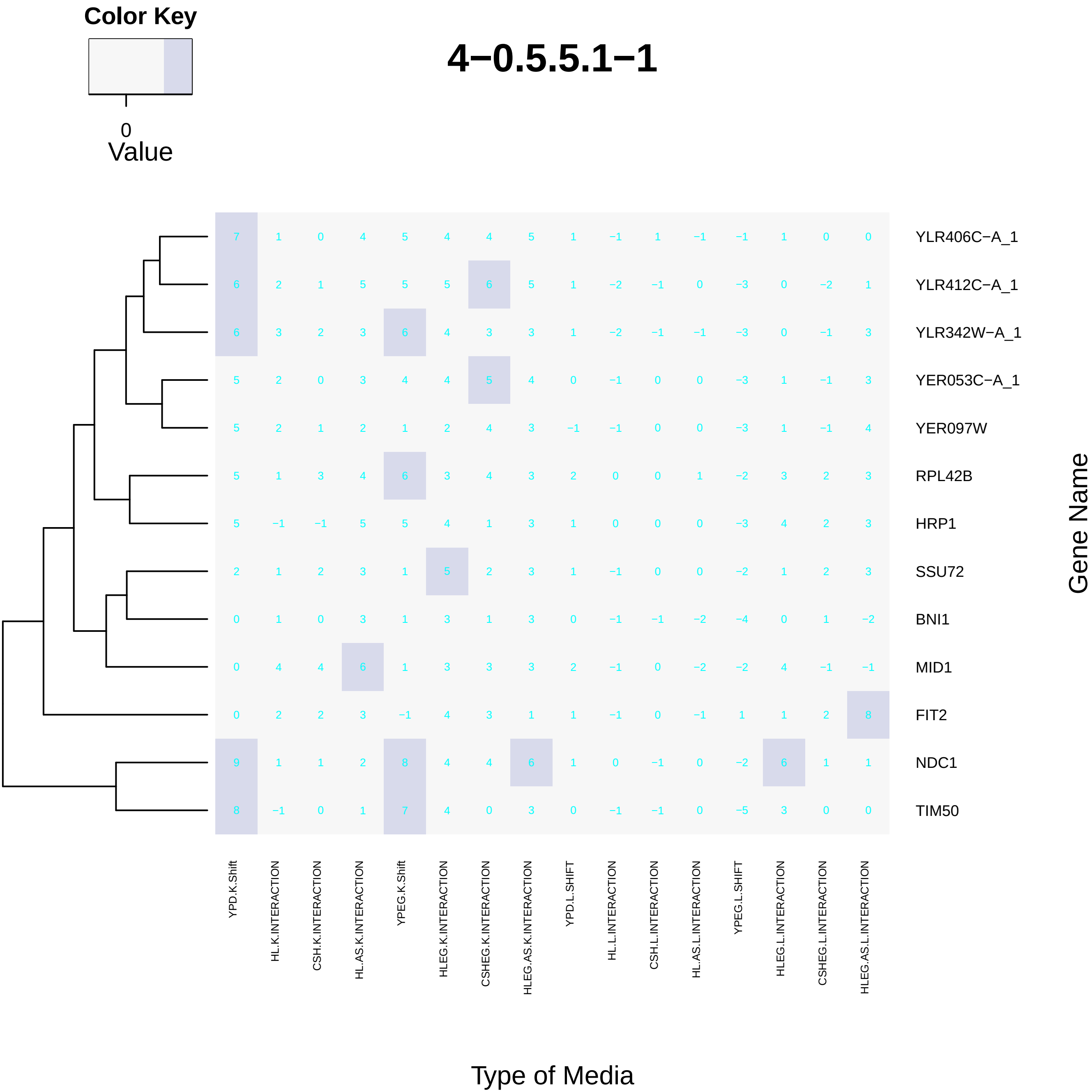

Color Key
0
Value
4−0.5.5.1−1
YLR406C−A_1
YLR412C−A_1
YLR342W−A_1
YER053C−A_1
YER097W
RPL42B
HRP1
SSU72
BNI1
MID1
FIT2
NDC1
TIM50
7 1 0 4 5 4 4 5 1 −1 1 −1 −1 1 0 0
6 2 1 5 5 5 6 5 1 −2 −1 0 −3 0 −2 1
6 3 2 3 6 4 3 3 1 −2 −1 −1 −3 0 −1 3
5 2 0 3 4 4 5 4 0 −1 0 0 −3 1 −1 3
5 2 1 2 1 2 4 3 −1 −1 0 0 −3 1 −1 4
5 1 3 4 6 3 4 3 2 0 0 1 −2 3 2 3
5 −1 −1 5 5 4 1 3 1 0 0 0 −3 4 2 3
2 1 2 3 1 5 2 3 1 −1 0 0 −2 1 2 3
0 1 0 3 1 3 1 3 0 −1 −1 −2 −4 0 1 −2
0 4 4 6 1 3 3 3 2 −1 0 −2 −2 4 −1 −1
0 2 2 3 −1 4 3 1 1 −1 0 −1 1 1 2 8
9 1 1 2 8 4 4 6 1 0 −1 0 −2 6 1 1
8 −1 0 1 7 4 0 3 0 −1 −1 0 −5 3 0 0
YPD.K.Shift
HL.K.INTERACTION
CSH.K.INTERACTION
HL.AS.K.INTERACTION
YPEG.K.Shift
HLEG.K.INTERACTION
CSHEG.K.INTERACTION
HLEG.AS.K.INTERACTION
YPD.L.SHIFT
HL.L.INTERACTION
CSH.L.INTERACTION
HL.AS.L.INTERACTION
YPEG.L.SHIFT
HLEG.L.INTERACTION
CSHEG.L.INTERACTION
HLEG.AS.L.INTERACTION
Gene Name
Type of Media

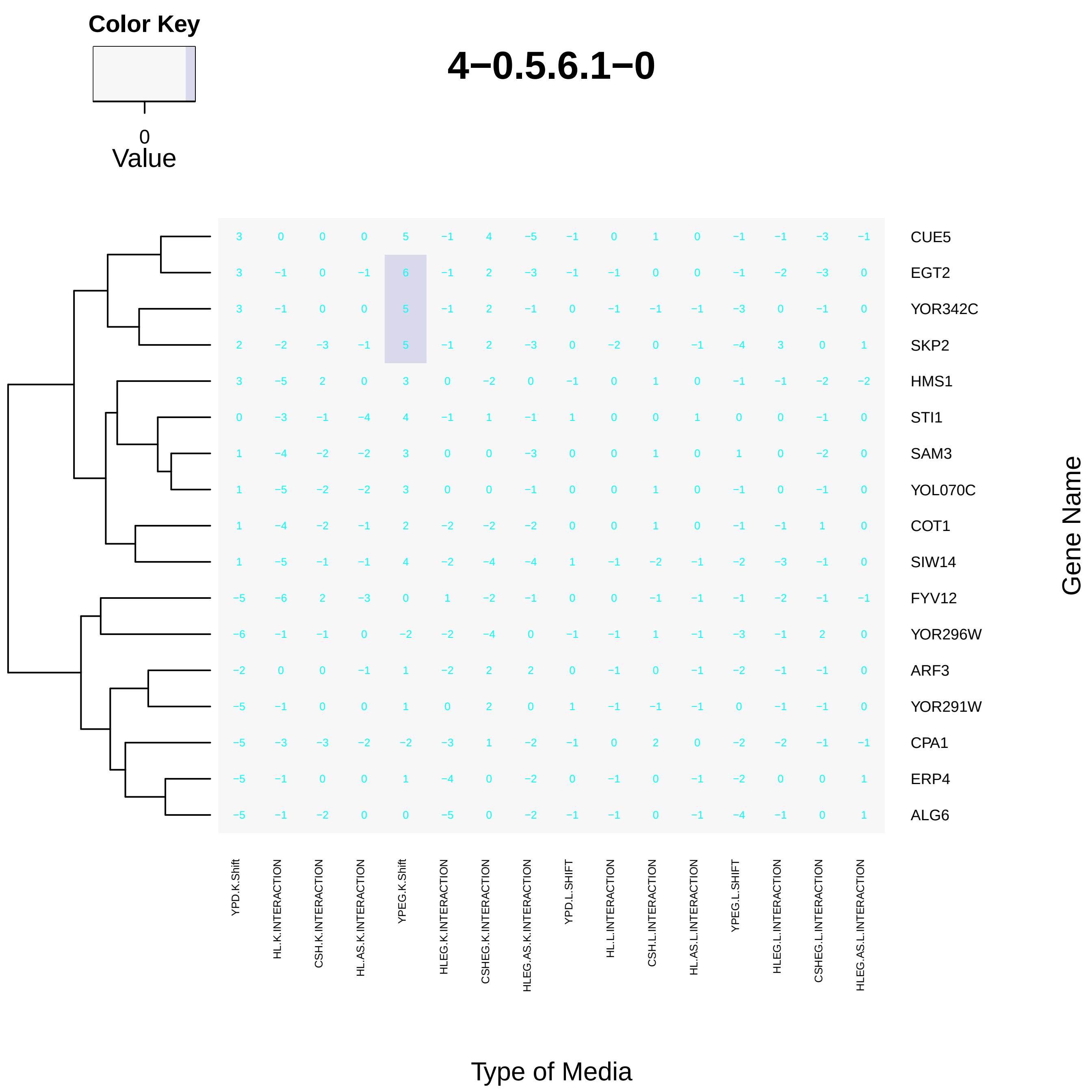
Color Key
0
Value
4−0.5.6.1−0
YPD.K.Shift, HL.K.INTERACTION, CSH.K.INTERACTION, HL.AS.K.INTERACTION, YPEG.K.Shift, HLEG.K.INTERACTION, CSHEG.K.INTERACTION, HLEG.AS.K.INTERACTION, YPD.L.SHIFT, HL.L.INTERACTION, CSH.L.INTERACTION, HL.AS.L.INTERACTION, YPEG.L.SHIFT, HLEG.L.INTERACTION, CSHEG.L.INTERACTION, HLEG.AS.L.INTERACTION
CUE5 3 0 0 0 5 −1 4 −5 −1 0 1 0 −1 −1 −3 −1
EGT2 3 −1 0 −1 6 −1 2 −3 −1 −1 0 0 −1 −2 −3 0
YOR342C 3 −1 0 0 5 −1 2 −1 0 −1 −1 −1 −3 0 −1 0
SKP2 2 −2 −3 −1 5 −1 2 −3 0 −2 0 −1 −4 3 0 1
HMS1 3 −5 2 0 3 0 −2 0 −1 0 1 0 −1 −1 −2 −2
STI1 0 −3 −1 −4 4 −1 1 −1 1 0 0 1 0 0 −1 0
SAM3 1 −4 −2 −2 3 0 0 −3 0 0 1 0 1 0 −2 0
YOL070C 1 −5 −2 −2 3 0 0 −1 0 0 1 0 −1 0 −1 0
COT1 1 −4 −2 −1 2 −2 −2 −2 0 0 1 0 −1 −1 1 0
SIW14 1 −5 −1 −1 4 −2 −4 −4 1 −1 −2 −1 −2 −3 −1 0
FYV12 −5 −6 2 −3 0 1 −2 −1 0 0 −1 −1 −1 −2 −1 −1
YOR296W −6 −1 −1 0 −2 −2 −4 0 −1 −1 1 −1 −3 −1 2 0
ARF3 −2 0 0 −1 1 −2 2 2 0 −1 0 −1 −2 −1 −1 0
YOR291W −5 −1 0 0 1 0 2 0 1 −1 −1 −1 0 −1 −1 0
CPA1 −5 −3 −3 −2 −2 −3 1 −2 −1 0 2 0 −2 −2 −1 −1
ERP4 −5 −1 0 0 1 −4 0 −2 0 −1 0 −1 −2 0 0 1
ALG6 −5 −1 −2 0 0 −5 0 −2 −1 −1 0 −1 −4 −1 0 1
Gene Name
Type of Media

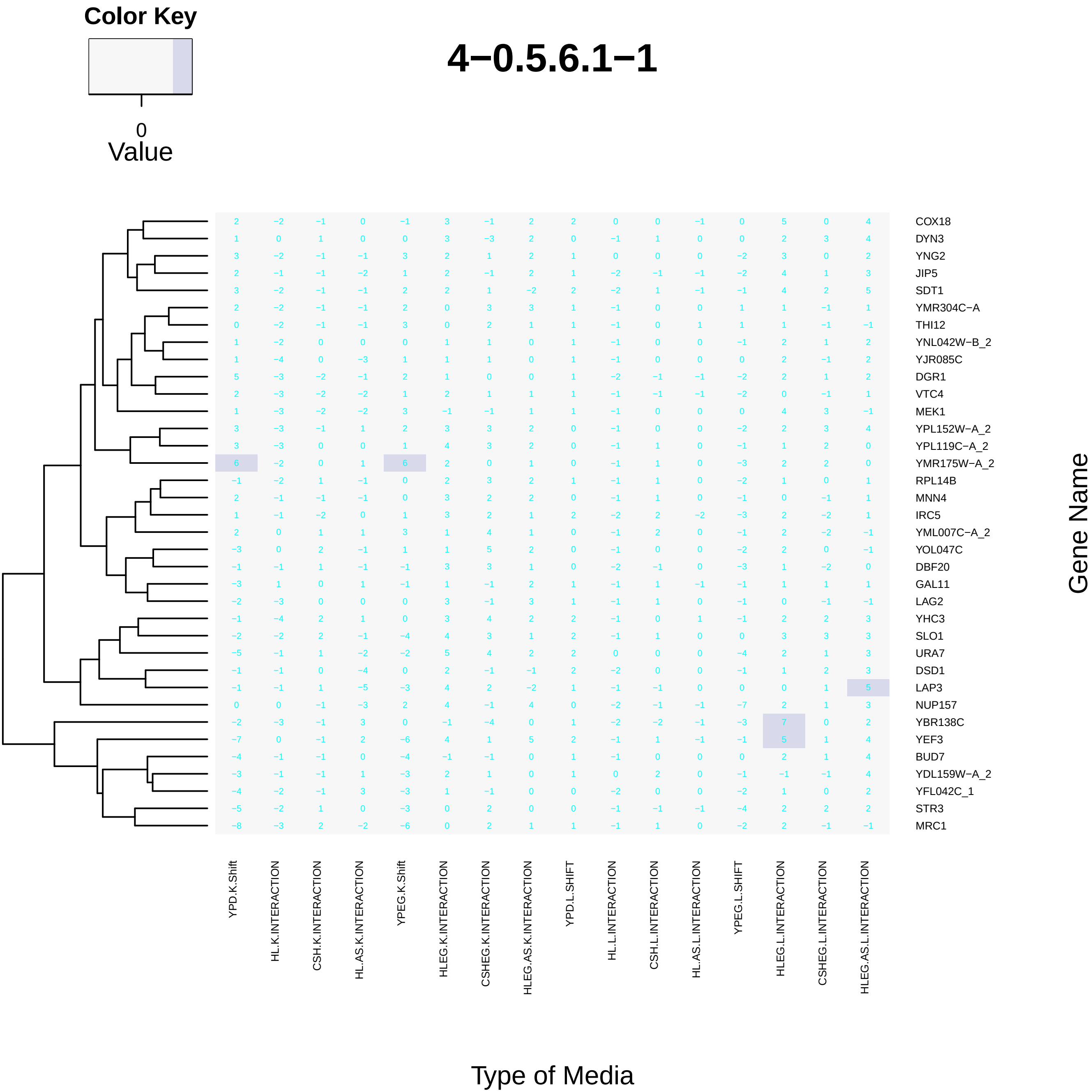
Color Key
0
Value
4−0.5.6.1−1
COX18
DYN3
YNG2
JIP5
SDT1
YMR304C−A
THI12
YNL042W−B_2
YJR085C
DGR1
VTC4
MEK1
YPL152W−A_2
YPL119C−A_2
YMR175W−A_2
RPL14B
MNN4
IRC5
YML007C−A_2
YOL047C
DBF20
GAL11
LAG2
YHC3
SLO1
URA7
DSD1
LAP3
NUP157
YBR138C
YEF3
BUD7
YDL159W−A_2
YFL042C_1
STR3
MRC1
Gene Name
YPD.K.Shift
HL.K.INTERACTION
CSH.K.INTERACTION
HL.AS.K.INTERACTION
YPEG.K.Shift
HLEG.K.INTERACTION
CSHEG.K.INTERACTION
HLEG.AS.K.INTERACTION
YPD.L.SHIFT
HL.L.INTERACTION
CSH.L.INTERACTION
HL.AS.L.INTERACTION
YPEG.L.SHIFT
HLEG.L.INTERACTION
CSHEG.L.INTERACTION
HLEG.AS.L.INTERACTION
Type of Media

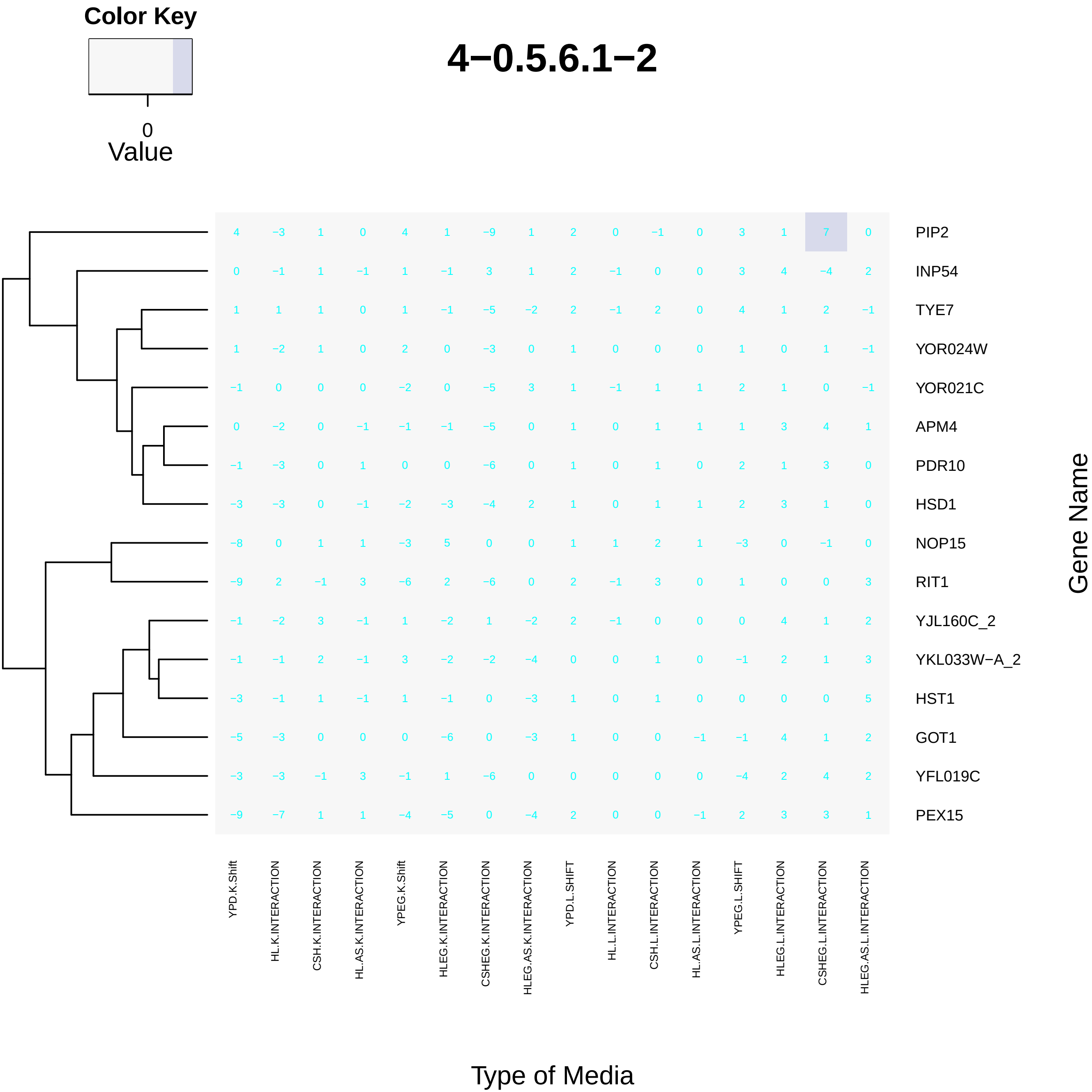
Color Key
0
Value
4−0.5.6.1−2
PIP2
INP54
TYE7
YOR024W
YOR021C
APM4
PDR10
HSD1
NOP15
RIT1
YJL160C_2
YKL033W−A_2
HST1
GOT1
YFL019C
PEX15
Gene Name
YPD.K.Shift
HL.K.INTERACTION
CSH.K.INTERACTION
HL.AS.K.INTERACTION
YPEG.K.Shift
HLEG.K.INTERACTION
CSHEG.K.INTERACTION
HLEG.AS.K.INTERACTION
YPD.L.SHIFT
HL.L.INTERACTION
CSH.L.INTERACTION
HL.AS.L.INTERACTION
YPEG.L.SHIFT
HLEG.L.INTERACTION
CSHEG.L.INTERACTION
HLEG.AS.L.INTERACTION
Type of Media
4 −3 1 0 4 1 −9 1 2 0 −1 0 3 1 7 0
0 −1 1 −1 1 −1 3 1 2 −1 0 0 3 4 −4 2
1 1 1 0 1 −1 −5 −2 2 −1 2 0 4 1 2 −1
1 −2 1 0 2 0 −3 0 1 0 0 0 1 0 1 −1
−1 0 0 0 −2 0 −5 3 1 −1 1 1 2 1 0 −1
0 −2 0 −1 −1 −1 −5 0 1 0 1 1 1 3 4 1
−1 −3 0 1 0 0 −6 0 1 0 1 0 2 1 3 0
−3 −3 0 −1 −2 −3 −4 2 1 0 1 1 2 3 1 0
−8 0 1 1 −3 5 0 0 1 1 2 1 −3 0 −1 0
−9 2 −1 3 −6 2 −6 0 2 −1 3 0 1 0 0 3
−1 −2 3 −1 1 −2 1 −2 2 −1 0 0 0 4 1 2
−1 −1 2 −1 3 −2 −2 −4 0 0 1 0 −1 2 1 3
−3 −1 1 −1 1 −1 0 −3 1 0 1 0 0 0 0 5
−5 −3 0 0 0 −6 0 −3 1 0 0 −1 −1 4 1 2
−3 −3 −1 3 −1 1 −6 0 0 0 0 0 −4 2 4 2
−9 −7 1 1 −4 −5 0 −4 2 0 0 −1 2 3 3 1

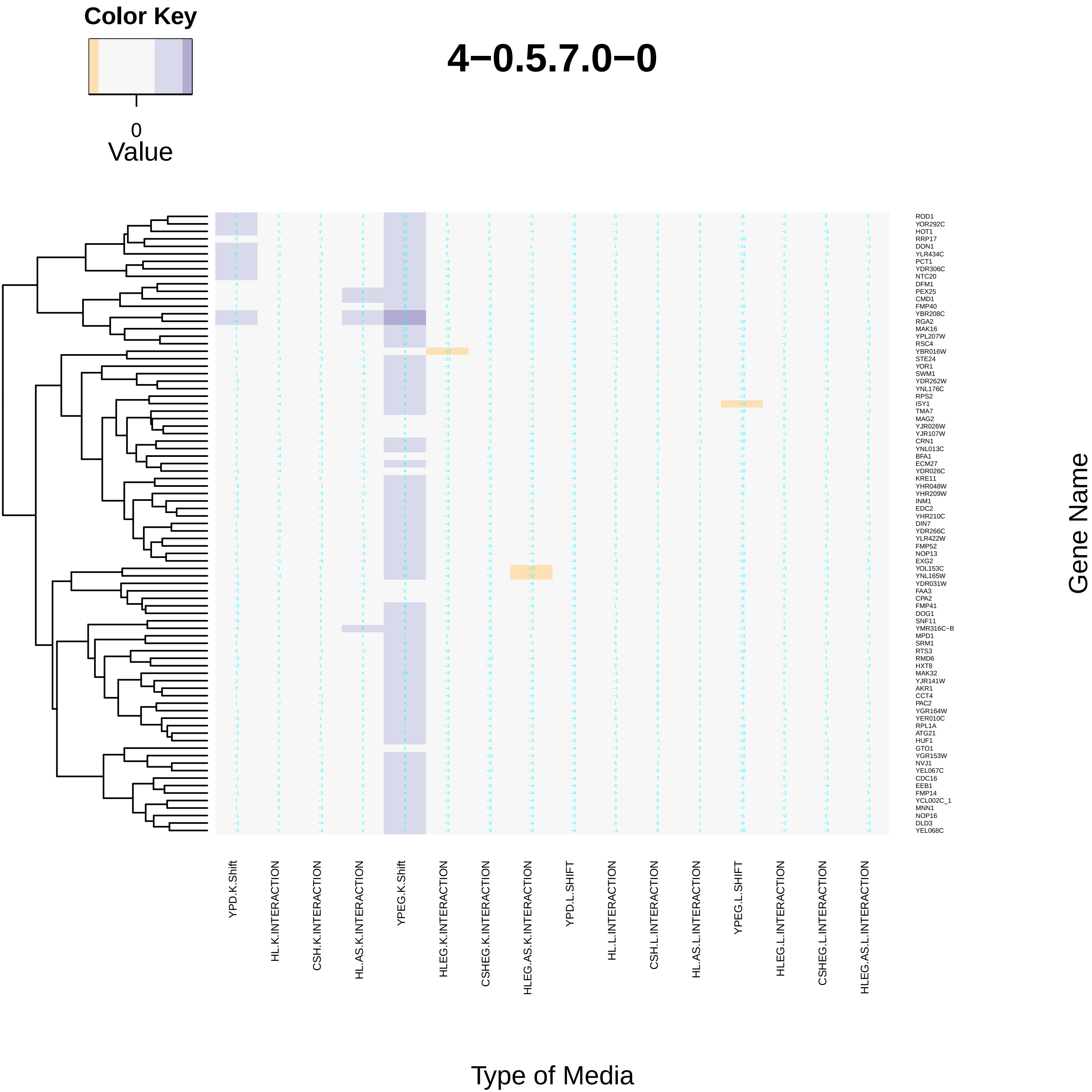
Color Key
0
Value
4−0.5.7.0−0
YPD.K.Shift
HL.K.INTERACTION
CSH.K.INTERACTION
HL.AS.K.INTERACTION
YPEG.K.Shift
HLEG.K.INTERACTION
CSHEG.K.INTERACTION
HLEG.AS.K.INTERACTION
YPD.L.SHIFT
HL.L.INTERACTION
CSH.L.INTERACTION
HL.AS.L.INTERACTION
YPEG.L.SHIFT
HLEG.L.INTERACTION
CSHEG.L.INTERACTION
HLEG.AS.L.INTERACTION
Type of Media
Gene Name
ROD1
YOR292C
HOT1
RRP17
DON1
YLR434C
PCT1
YDR306C
NTC20
DFM1
PEX25
CMD1
FMP40
YBR208C
RGA2
MAK16
YPL207W
RSC4
YBR016W
STE24
YOR1
SWM1
YDR262W
YNL176C
RPS2
ISY1
TMA7
MAG2
YJR026W
YJR107W
CRN1
YNL013C
BFA1
ECM27
YDR026C
KRE11
YHR048W
YHR209W
INM1
EDC2
YHR210C
DIN7
YDR266C
YLR422W
FMP52
NOP13
EXG2
YOL153C
YNL165W
YDR031W
FAA3
CPA2
FMP41
DOG1
SNF11
YMR316C−B
MPD1
SRM1
RTS3
RMD6
HXT8
MAK32
YJR141W
AKR1
CCT4
PAC2
YGR164W
YER010C
RPL1A
ATG21
HUF1
GTO1
YGR153W
NVJ1
YEL067C
CDC16
EEB1
FMP14
YCL002C_1
MNN1
NOP16
DLD3
YEL068C

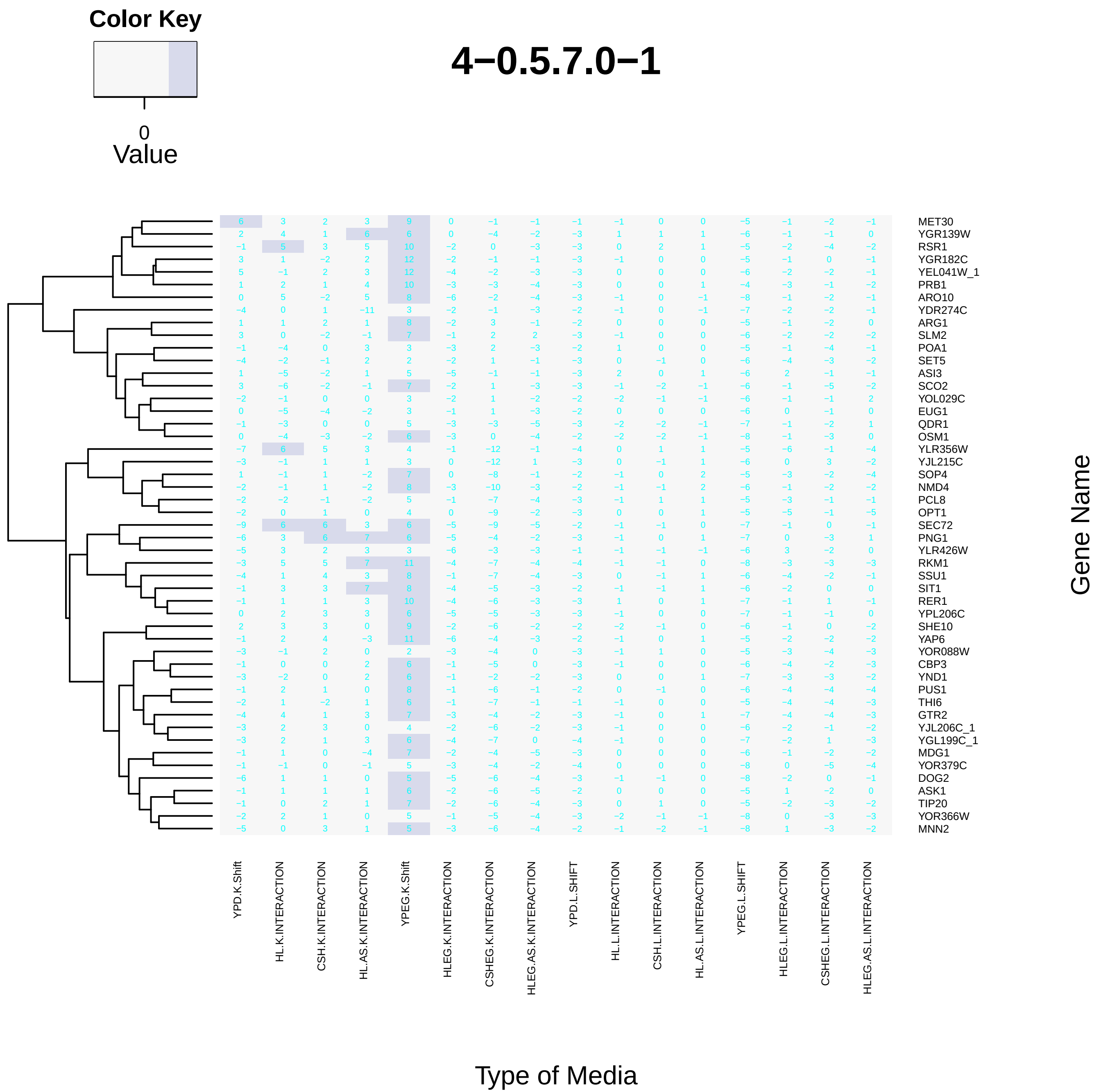
Color Key
0
Value
4−0.5.7.0−1
MET30
YGR139W
RSR1
YGR182C
YEL041W_1
PRB1
ARO10
YDR274C
ARG1
SLM2
POA1
SET5
ASI3
SCO2
YOL029C
EUG1
QDR1
OSM1
YLR356W
YJL215C
SOP4
NMD4
PCL8
OPT1
SEC72
PNG1
YLR426W
RKM1
SSU1
SIT1
RER1
YPL206C
SHE10
YAP6
YOR088W
CBP3
YND1
PUS1
THI6
GTR2
YJL206C_1
YGL199C_1
MDG1
YOR379C
DOG2
ASK1
TIP20
YOR366W
MNN2
Gene Name
YPD.K.Shift
HL.K.INTERACTION
CSH.K.INTERACTION
HL.AS.K.INTERACTION
YPEG.K.Shift
HLEG.K.INTERACTION
CSHEG.K.INTERACTION
HLEG.AS.K.INTERACTION
YPD.L.SHIFT
HL.L.INTERACTION
CSH.L.INTERACTION
HL.AS.L.INTERACTION
YPEG.L.SHIFT
HLEG.L.INTERACTION
CSHEG.L.INTERACTION
HLEG.AS.L.INTERACTION
Type of Media

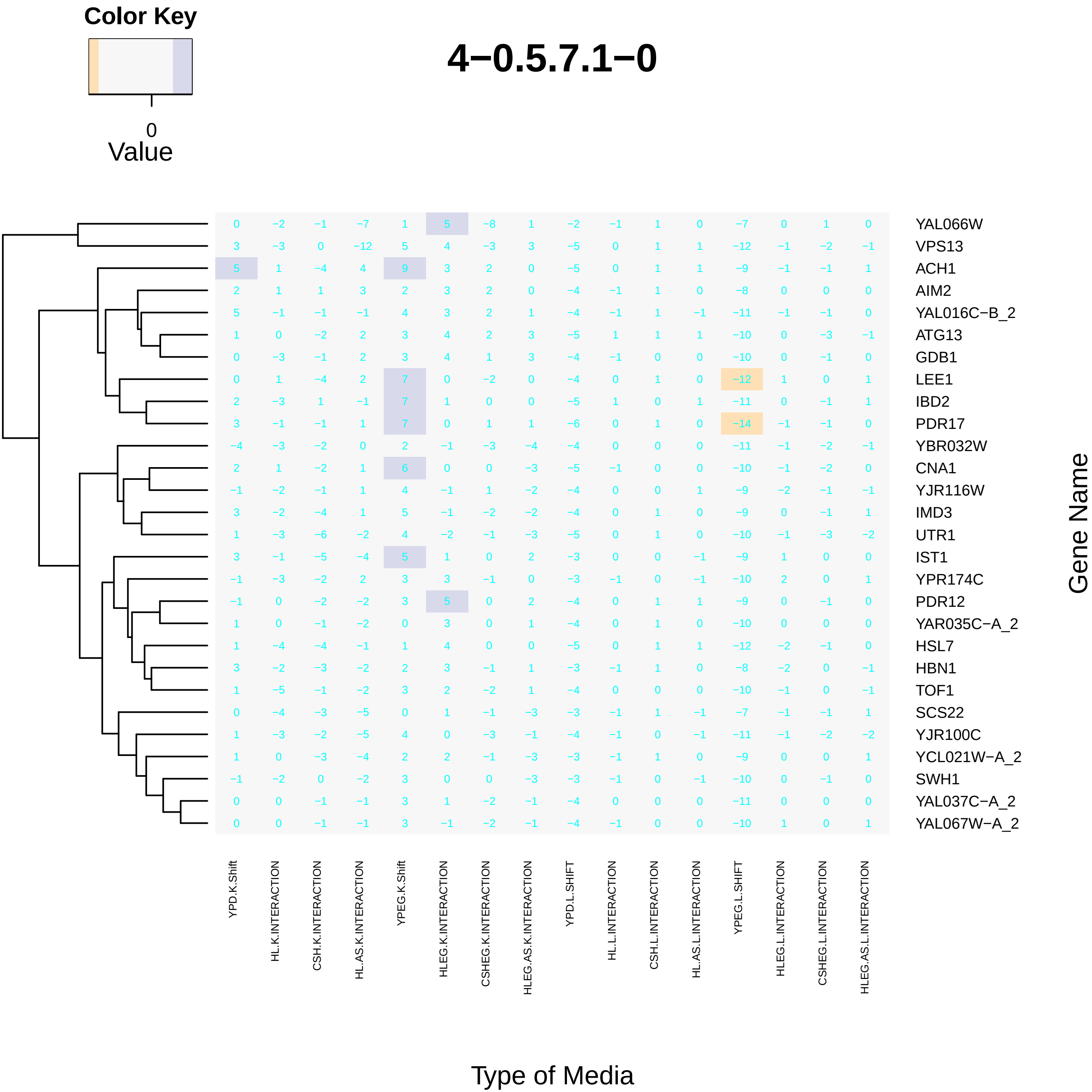
4−0.5.7.1−0
Color Key
0
Value
YPD.K.Shift HL.K.INTERACTION CSH.K.INTERACTION HL.AS.K.INTERACTION YPEG.K.Shift HLEG.K.INTERACTION CSHEG.K.INTERACTION HLEG.AS.K.INTERACTION YPD.L.SHIFT HL.L.INTERACTION CSH.L.INTERACTION HL.AS.L.INTERACTION YPEG.L.SHIFT HLEG.L.INTERACTION CSHEG.L.INTERACTION HLEG.AS.L.INTERACTION
YAL066W 0 −2 −1 −7 1 5 −8 1 −2 −1 1 0 −7 0 1 0
VPS13 3 −3 0 −12 5 4 −3 3 −5 0 1 1 −12 −1 −2 −1
ACH1 5 1 −4 4 9 3 2 0 −5 0 1 1 −9 −1 −1 1
AIM2 2 1 1 3 2 3 2 0 −4 −1 1 0 −8 0 0 0
YAL016C−B_2 5 −1 −1 −1 4 3 2 1 −4 −1 1 −1 −11 −1 −1 0
ATG13 1 0 −2 2 3 4 2 3 −5 1 1 1 −10 0 −3 −1
GDB1 0 −3 −1 2 3 4 1 3 −4 −1 0 0 −10 0 −1 0
LEE1 0 1 −4 2 7 0 −2 0 −4 0 1 0 −12 1 0 1
IBD2 2 −3 1 −1 7 1 0 0 −5 1 0 1 −11 0 −1 1
PDR17 3 −1 −1 1 7 0 1 1 −6 0 1 0 −14 −1 −1 0
YBR032W −4 −3 −2 0 2 −1 −3 −4 −4 0 0 0 −11 −1 −2 −1
CNA1 2 1 −2 1 6 0 0 −3 −5 −1 0 0 −10 −1 −2 0
YJR116W −1 −2 −1 1 4 −1 1 −2 −4 0 0 1 −9 −2 −1 −1
IMD3 3 −2 −4 1 5 −1 −2 −2 −4 0 1 0 −9 0 −1 1
UTR1 1 −3 −6 −2 4 −2 −1 −3 −5 0 1 0 −10 −1 −3 −2
IST1 3 −1 −5 −4 5 1 0 2 −3 0 0 −1 −9 1 0 0
YPR174C −1 −3 −2 2 3 3 −1 0 −3 −1 0 −1 −10 2 0 1
PDR12 −1 0 −2 −2 3 5 0 2 −4 0 1 1 −9 0 −1 0
YAR035C−A_2 1 0 −1 −2 0 3 0 1 −4 0 1 0 −10 0 0 0
HSL7 1 −4 −4 −1 1 4 0 0 −5 0 1 1 −12 −2 −1 0
HBN1 3 −2 −3 −2 2 3 −1 1 −3 −1 1 0 −8 −2 0 −1
TOF1 1 −5 −1 −2 3 2 −2 1 −4 0 0 0 −10 −1 0 −1
SCS22 0 −4 −3 −5 0 1 −1 −3 −3 −1 1 −1 −7 −1 −1 1
YJR100C 1 −3 −2 −5 4 0 −3 −1 −4 −1 0 −1 −11 −1 −2 −2
YCL021W−A_2 1 0 −3 −4 2 2 −1 −3 −3 −1 1 0 −9 0 0 1
SWH1 −1 −2 0 −2 3 0 0 −3 −3 −1 0 −1 −10 0 −1 0
YAL037C−A_2 0 0 −1 −1 3 1 −2 −1 −4 0 0 0 −11 0 0 0
YAL067W−A_2 0 0 −1 −1 3 −1 −2 −1 −4 −1 0 0 −10 1 0 1
Gene Name
Type of Media

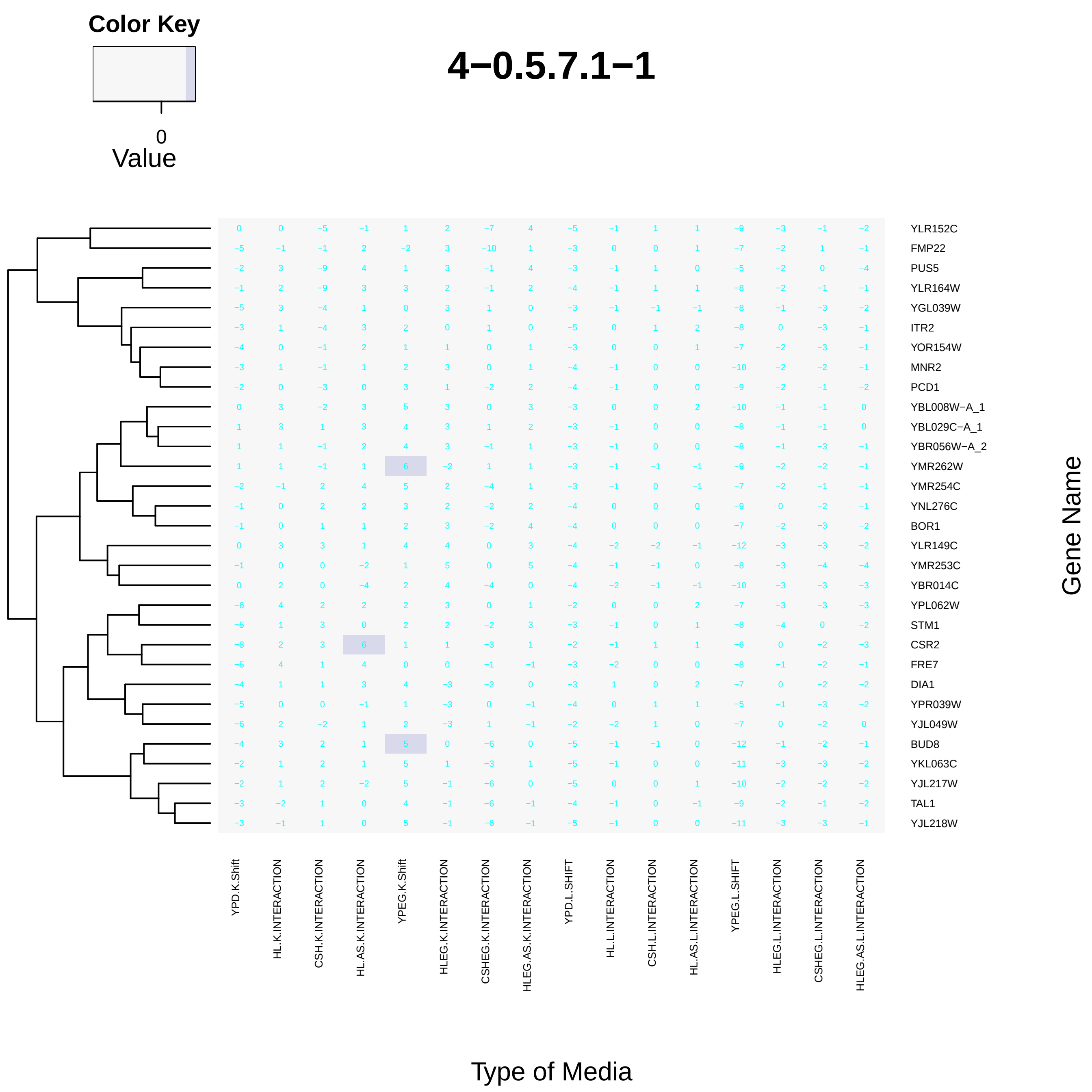
Color Key
0
Value
4−0.5.7.1−1
YPD.K.Shift HL.K.INTERACTION CSH.K.INTERACTION HL.AS.K.INTERACTION YPEG.K.Shift HLEG.K.INTERACTION CSHEG.K.INTERACTION HLEG.AS.K.INTERACTION YPD.L.SHIFT HL.L.INTERACTION CSH.L.INTERACTION HL.AS.L.INTERACTION YPEG.L.SHIFT HLEG.L.INTERACTION CSHEG.L.INTERACTION HLEG.AS.L.INTERACTION
0 0 −5 −1 1 2 −7 4 −5 −1 1 1 −9 −3 −1 −2 YLR152C
−5 −1 −1 2 −2 3 −10 1 −3 0 0 1 −7 −2 1 −1 FMP22
−2 3 −9 4 1 3 −1 4 −3 −1 1 0 −5 −2 0 −4 PUS5
−1 2 −9 3 3 2 −1 2 −4 −1 1 1 −8 −2 −1 −1 YLR164W
−5 3 −4 1 0 3 1 0 −3 −1 −1 −1 −8 −1 −3 −2 YGL039W
−3 1 −4 3 2 0 1 0 −5 0 1 2 −8 0 −3 −1 ITR2
−4 0 −1 2 1 1 0 1 −3 0 0 1 −7 −2 −3 −1 YOR154W
−3 1 −1 1 2 3 0 1 −4 −1 0 0 −10 −2 −2 −1 MNR2
−2 0 −3 0 3 1 −2 2 −4 −1 0 0 −9 −2 −1 −2 PCD1
0 3 −2 3 5 3 0 3 −3 0 0 2 −10 −1 −1 0 YBL008W−A_1
1 3 1 3 4 3 1 2 −3 −1 0 0 −8 −1 −1 0 YBL029C−A_1
1 1 −1 2 4 3 −1 1 −3 −1 0 0 −8 −1 −3 −1 YBR056W−A_2
1 1 −1 1 6 −2 1 1 −3 −1 −1 −1 −9 −2 −2 −1 YMR262W
−2 −1 2 4 5 2 −4 1 −3 −1 0 −1 −7 −2 −1 −1 YMR254C
−1 0 2 2 3 2 −2 2 −4 0 0 0 −9 0 −2 −1 YNL276C
−1 0 1 1 2 3 −2 4 −4 0 0 0 −7 −2 −3 −2 BOR1
0 3 3 1 4 4 0 3 −4 −2 −2 −1 −12 −3 −3 −2 YLR149C
−1 0 0 −2 1 5 0 5 −4 −1 −1 0 −8 −3 −4 −4 YMR253C
0 2 0 −4 2 4 −4 0 −4 −2 −1 −1 −10 −3 −3 −3 YBR014C
−6 4 2 2 2 3 0 1 −2 0 0 2 −7 −3 −3 −3 YPL062W
−5 1 3 0 2 2 −2 3 −3 −1 0 1 −8 −4 0 −2 STM1
−8 2 3 6 1 1 −3 1 −2 −1 1 1 −6 0 −2 −3 CSR2
−5 4 1 4 0 0 −1 −1 −3 −2 0 0 −8 −1 −2 −1 FRE7
−4 1 1 3 4 −3 −2 0 −3 1 0 2 −7 0 −2 −2 DIA1
−5 0 0 −1 1 −3 0 −1 −4 0 1 1 −5 −1 −3 −2 YPR039W
−6 2 −2 1 2 −3 1 −1 −2 −2 1 0 −7 0 −2 0 YJL049W
−4 3 2 1 5 0 −6 0 −5 −1 −1 0 −12 −1 −2 −1 BUD8
−2 1 2 1 5 1 −3 1 −5 −1 0 0 −11 −3 −3 −2 YKL063C
−2 1 2 −2 5 −1 −6 0 −5 0 0 1 −10 −2 −2 −2 YJL217W
−3 −2 1 0 4 −1 −6 −1 −4 −1 0 −1 −9 −2 −1 −2 TAL1
−3 −1 1 0 5 −1 −6 −1 −5 −1 0 0 −11 −3 −3 −1 YJL218W
Gene Name
Type of Media

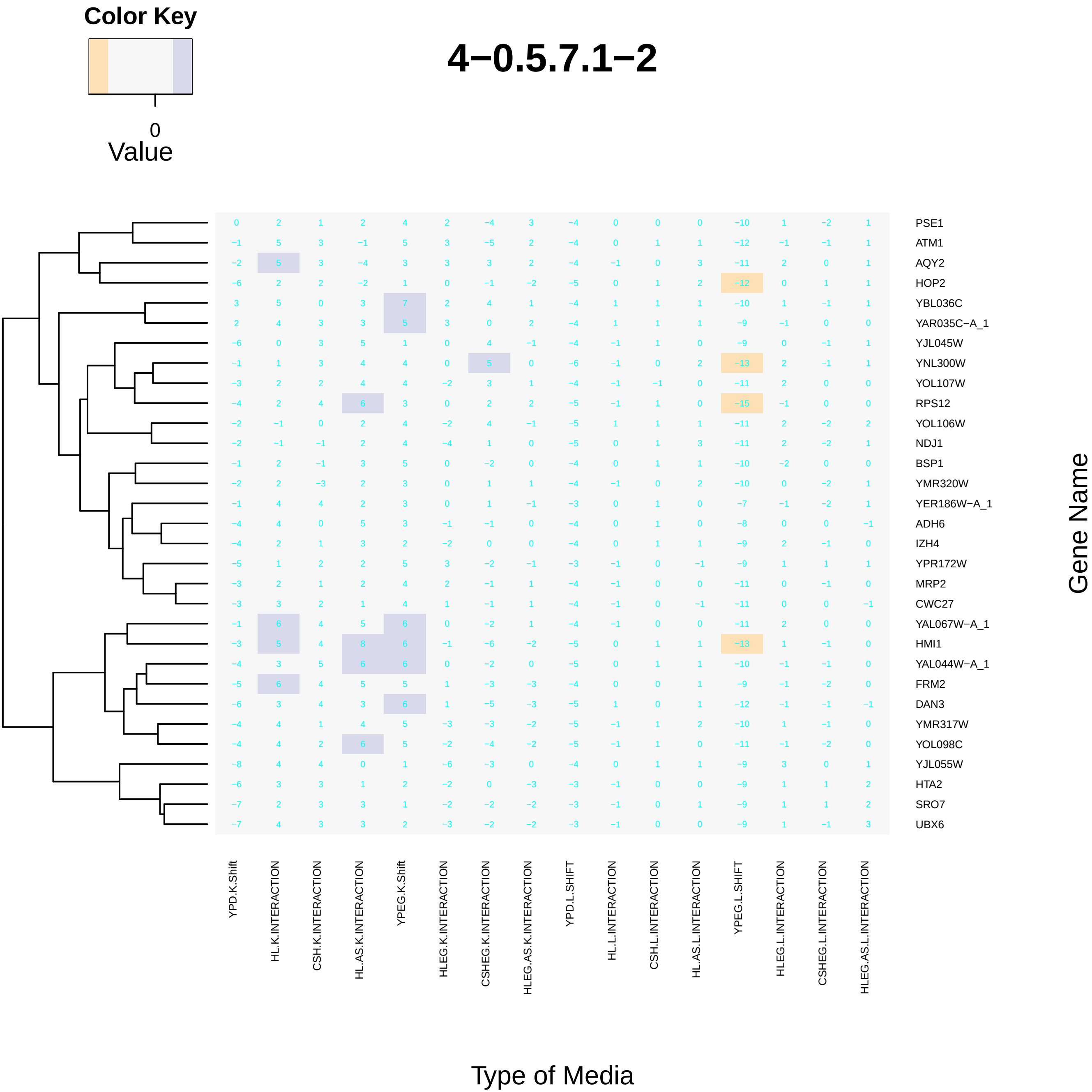
Color Key
0
Value
4−0.5.7.1−2
YPD.K.Shift
HL.K.INTERACTION
CSH.K.INTERACTION
HL.AS.K.INTERACTION
YPEG.K.Shift
HLEG.K.INTERACTION
CSHEG.K.INTERACTION
HLEG.AS.K.INTERACTION
YPD.L.SHIFT
HL.L.INTERACTION
CSH.L.INTERACTION
HL.AS.L.INTERACTION
YPEG.L.SHIFT
HLEG.L.INTERACTION
CSHEG.L.INTERACTION
HLEG.AS.L.INTERACTION
PSE1
ATM1
AQY2
HOP2
YBL036C
YAR035C−A_1
YJL045W
YNL300W
YOL107W
RPS12
YOL106W
NDJ1
BSP1
YMR320W
YER186W−A_1
ADH6
IZH4
YPR172W
MRP2
CWC27
YAL067W−A_1
HMI1
YAL044W−A_1
FRM2
DAN3
YMR317W
YOL098C
YJL055W
HTA2
SRO7
UBX6
Gene Name
Type of Media

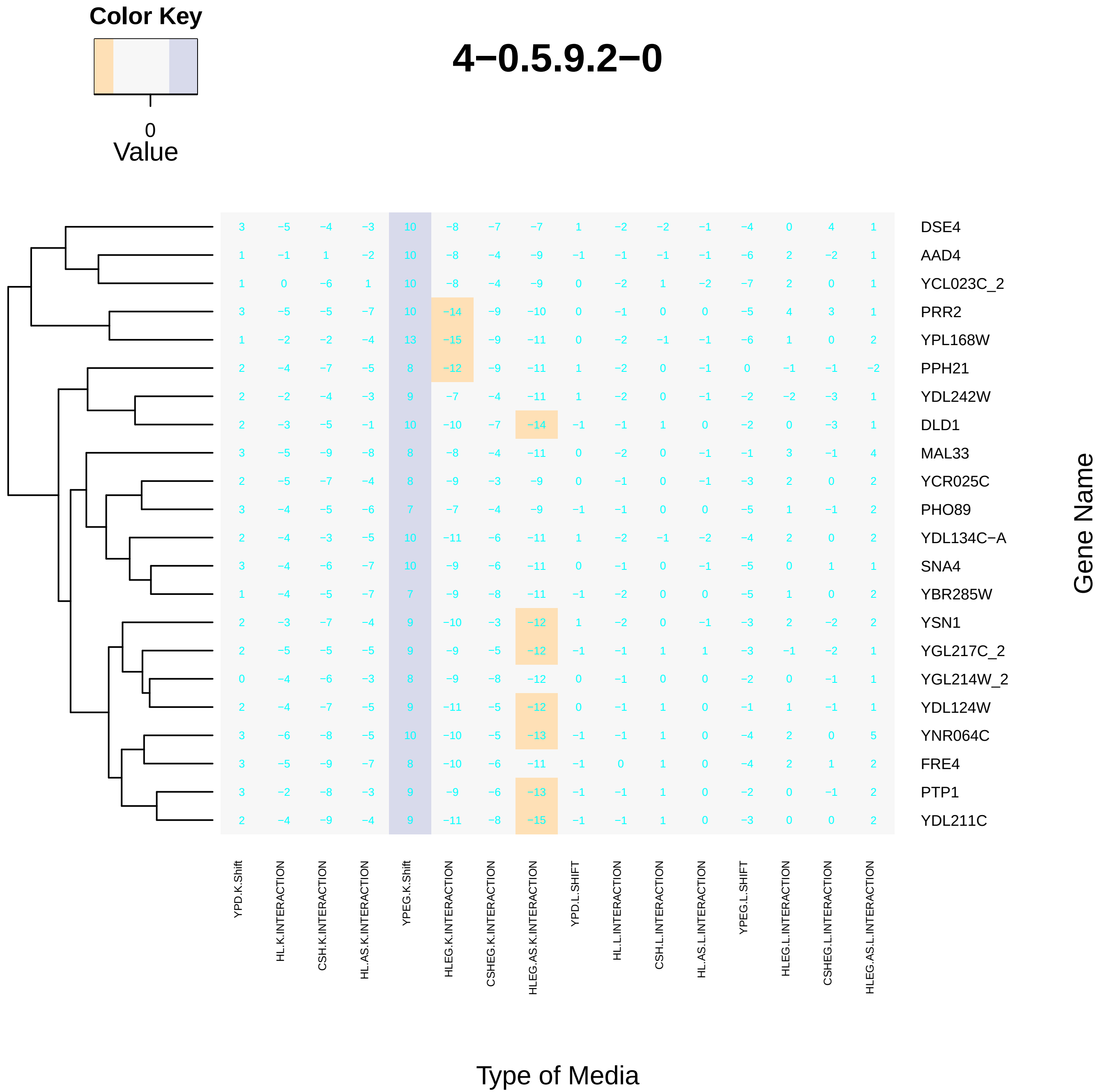
Color Key
0
Value
4−0.5.9.2−0
3 −5 −4 −3 10 −8 −7 −7 1 −2 −2 −1 −4 0 4 1 DSE4
1 −1 1 −2 10 −8 −4 −9 −1 −1 −1 −1 −6 2 −2 1 AAD4
1 0 −6 1 10 −8 −4 −9 0 −2 1 −2 −7 2 0 1 YCL023C_2
3 −5 −5 −7 10 −14 −9 −10 0 −1 0 0 −5 4 3 1 PRR2
1 −2 −2 −4 13 −15 −9 −11 0 −2 −1 −1 −6 1 0 2 YPL168W
2 −4 −7 −5 8 −12 −9 −11 1 −2 0 −1 0 −1 −1 −2 PPH21
2 −2 −4 −3 9 −7 −4 −11 1 −2 0 −1 −2 −2 −3 1 YDL242W
2 −3 −5 −1 10 −10 −7 −14 −1 −1 1 0 −2 0 −3 1 DLD1
3 −5 −9 −8 8 −8 −4 −11 0 −2 0 −1 −1 3 −1 4 MAL33
2 −5 −7 −4 8 −9 −3 −9 0 −1 0 −1 −3 2 0 2 YCR025C
3 −4 −5 −6 7 −7 −4 −9 −1 −1 0 0 −5 1 −1 2 PHO89
2 −4 −3 −5 10 −11 −6 −11 1 −2 −1 −2 −4 2 0 2 YDL134C−A
3 −4 −6 −7 10 −9 −6 −11 0 −1 0 −1 −5 0 1 1 SNA4
1 −4 −5 −7 7 −9 −8 −11 −1 −2 0 0 −5 1 0 2 YBR285W
2 −3 −7 −4 9 −10 −3 −12 1 −2 0 −1 −3 2 −2 2 YSN1
2 −5 −5 −5 9 −9 −5 −12 −1 −1 1 1 −3 −1 −2 1 YGL217C_2
0 −4 −6 −3 8 −9 −8 −12 0 −1 0 0 −2 0 −1 1 YGL214W_2
2 −4 −7 −5 9 −11 −5 −12 0 −1 1 0 −1 1 −1 1 YDL124W
3 −6 −8 −5 10 −10 −5 −13 −1 −1 1 0 −4 2 0 5 YNR064C
3 −5 −9 −7 8 −10 −6 −11 −1 0 1 0 −4 2 1 2 FRE4
3 −2 −8 −3 9 −9 −6 −13 −1 −1 1 0 −2 0 −1 2 PTP1
2 −4 −9 −4 9 −11 −8 −15 −1 −1 1 0 −3 0 0 2 YDL211C
Gene Name
YPD.K.Shift
HL.K.INTERACTION
CSH.K.INTERACTION
HL.AS.K.INTERACTION
YPEG.K.Shift
HLEG.K.INTERACTION
CSHEG.K.INTERACTION
HLEG.AS.K.INTERACTION
YPD.L.SHIFT
HL.L.INTERACTION
CSH.L.INTERACTION
HL.AS.L.INTERACTION
YPEG.L.SHIFT
HLEG.L.INTERACTION
CSHEG.L.INTERACTION
HLEG.AS.L.INTERACTION
Type of Media

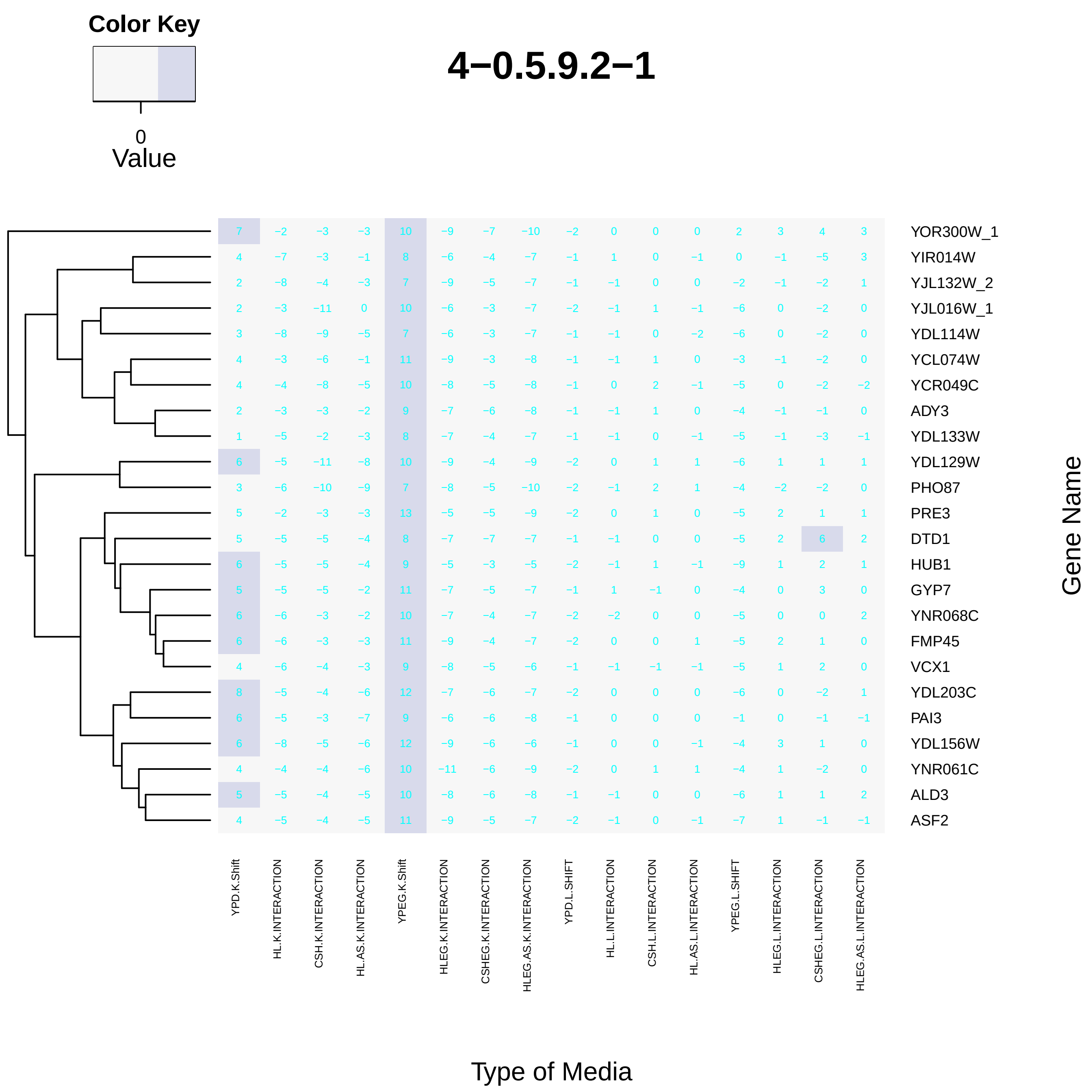
Color Key
0
Value
4−0.5.9.2−1
YPD.K.Shift
HL.K.INTERACTION
CSH.K.INTERACTION
HL.AS.K.INTERACTION
YPEG.K.Shift
HLEG.K.INTERACTION
CSHEG.K.INTERACTION
HLEG.AS.K.INTERACTION
YPD.L.SHIFT
HL.L.INTERACTION
CSH.L.INTERACTION
HL.AS.L.INTERACTION
YPEG.L.SHIFT
HLEG.L.INTERACTION
CSHEG.L.INTERACTION
HLEG.AS.L.INTERACTION
7 −2 −3 −3 10 −9 −7 −10 −2 0 0 0 2 3 4 3 YOR300W_1
4 −7 −3 −1 8 −6 −4 −7 −1 1 0 −1 0 −1 −5 3 YIR014W
2 −8 −4 −3 7 −9 −5 −7 −1 −1 0 0 −2 −1 −2 1 YJL132W_2
2 −3 −11 0 10 −6 −3 −7 −2 −1 1 −1 −6 0 −2 0 YJL016W_1
3 −8 −9 −5 7 −6 −3 −7 −1 −1 0 −2 −6 0 −2 0 YDL114W
4 −3 −6 −1 11 −9 −3 −8 −1 −1 1 0 −3 −1 −2 0 YCL074W
4 −4 −8 −5 10 −8 −5 −8 −1 0 2 −1 −5 0 −2 −2 YCR049C
2 −3 −3 −2 9 −7 −6 −8 −1 −1 1 0 −4 −1 −1 0 ADY3
1 −5 −2 −3 8 −7 −4 −7 −1 −1 0 −1 −5 −1 −3 −1 YDL133W
6 −5 −11 −8 10 −9 −4 −9 −2 0 1 1 −6 1 1 1 YDL129W
3 −6 −10 −9 7 −8 −5 −10 −2 −1 2 1 −4 −2 −2 0 PHO87
5 −2 −3 −3 13 −5 −5 −9 −2 0 1 0 −5 2 1 1 PRE3
5 −5 −5 −4 8 −7 −7 −7 −1 −1 0 0 −5 2 6 2 DTD1
6 −5 −5 −4 9 −5 −3 −5 −2 −1 1 −1 −9 1 2 1 HUB1
5 −5 −5 −2 11 −7 −5 −7 −1 1 −1 0 −4 0 3 0 GYP7
6 −6 −3 −2 10 −7 −4 −7 −2 −2 0 0 −5 0 0 2 YNR068C
6 −6 −3 −3 11 −9 −4 −7 −2 0 0 1 −5 2 1 0 FMP45
4 −6 −4 −3 9 −8 −5 −6 −1 −1 −1 −1 −5 1 2 0 VCX1
8 −5 −4 −6 12 −7 −6 −7 −2 0 0 0 −6 0 −2 1 YDL203C
6 −5 −3 −7 9 −6 −6 −8 −1 0 0 0 −1 0 −1 −1 PAI3
6 −8 −5 −6 12 −9 −6 −6 −1 0 0 −1 −4 3 1 0 YDL156W
4 −4 −4 −6 10 −11 −6 −9 −2 0 1 1 −4 1 −2 0 YNR061C
5 −5 −4 −5 10 −8 −6 −8 −1 −1 0 0 −6 1 1 2 ALD3
4 −5 −4 −5 11 −9 −5 −7 −2 −1 0 −1 −7 1 −1 −1 ASF2
Gene Name
Type of Media

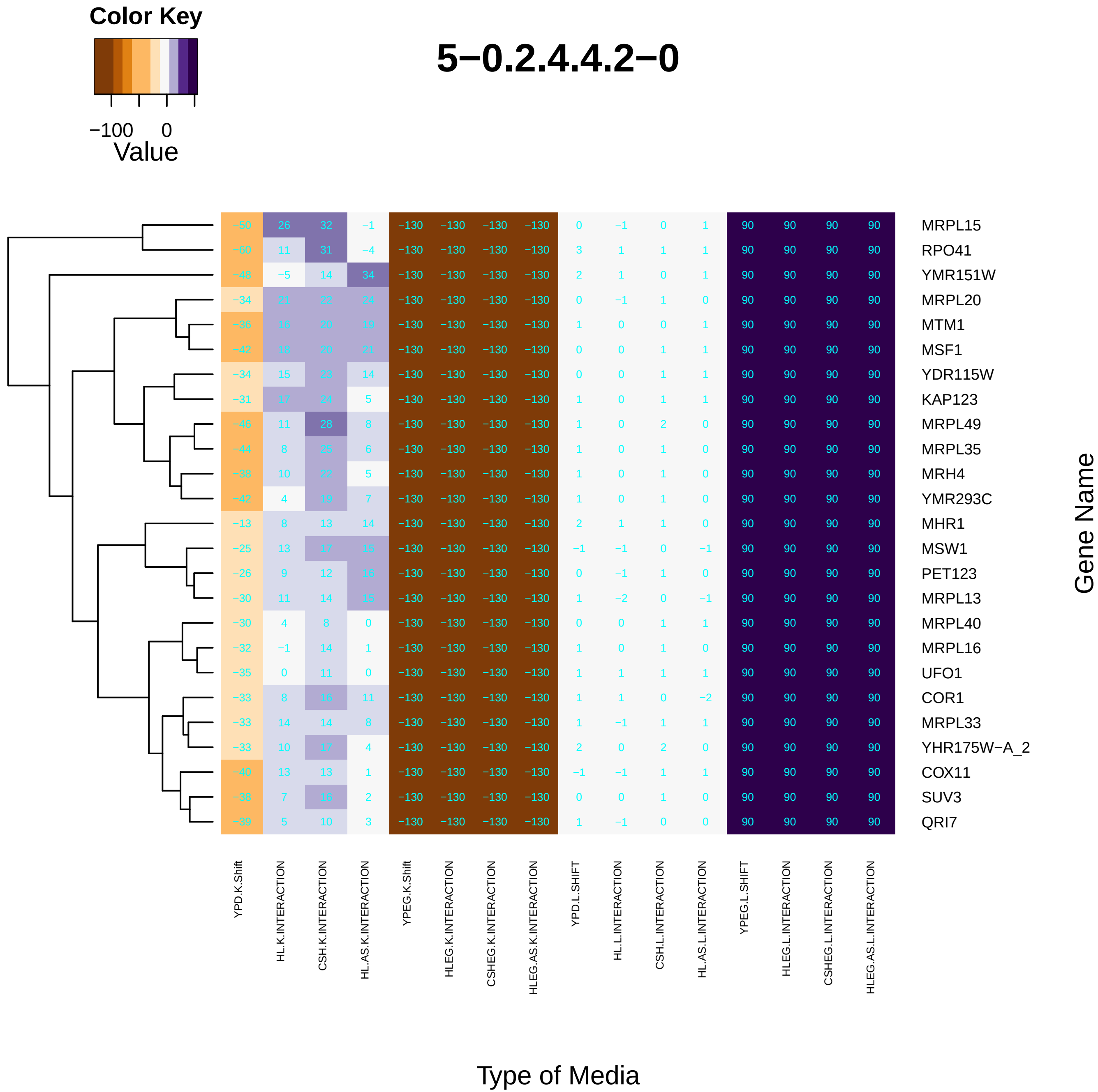

Color Key
−100
0
Value
5−0.2.4.4.2−0
Gene Name
Type of Media
YPD.K.Shift
HL.K.INTERACTION
CSH.K.INTERACTION
HL.AS.K.INTERACTION
YPEG.K.Shift
HLEG.K.INTERACTION
CSHEG.K.INTERACTION
HLEG.AS.K.INTERACTION
YPD.L.SHIFT
HL.L.INTERACTION
CSH.L.INTERACTION
HL.AS.L.INTERACTION
YPEG.L.SHIFT
HLEG.L.INTERACTION
CSHEG.L.INTERACTION
HLEG.AS.L.INTERACTION
MRPL15
RPO41
YMR151W
MRPL20
MTM1
MSF1
YDR115W
KAP123
MRPL49
MRPL35
MRH4
YMR293C
MHR1
MSW1
PET123
MRPL13
MRPL40
MRPL16
UFO1
COR1
MRPL33
YHR175W−A_2
COX11
SUV3
QRI7

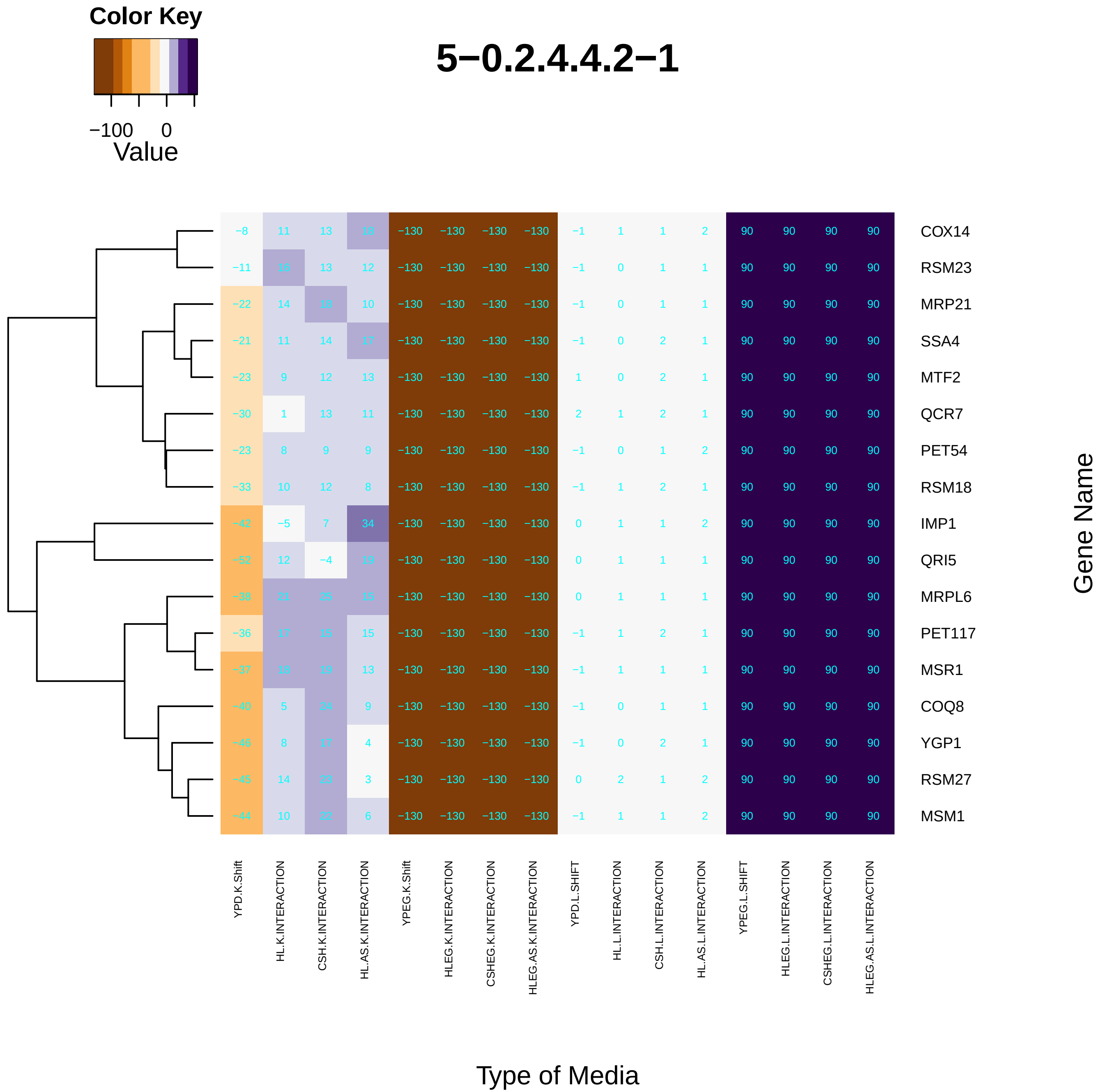
Color Key
−100 0
Value
5−0.2.4.4.2−1
−8 11 13 18 −130 −130 −130 −130 −1 1 1 2 90 90 90 90 COX14
−11 16 13 12 −130 −130 −130 −130 −1 0 1 1 90 90 90 90 RSM23
−22 14 28 10 −130 −130 −130 −130 −1 0 1 1 90 90 90 90 MRP21
−21 11 14 17 −130 −130 −130 −130 −1 0 2 1 90 90 90 90 SSA4
−23 9 12 13 −130 −130 −130 −130 1 0 2 1 90 90 90 90 MTF2
−30 1 13 11 −130 −130 −130 −130 2 1 2 1 90 90 90 90 QCR7
−23 8 9 9 −130 −130 −130 −130 −1 0 1 2 90 90 90 90 PET54
−33 10 12 8 −130 −130 −130 −130 −1 1 2 1 90 90 90 90 RSM18
−42 −5 7 34 −130 −130 −130 −130 0 1 1 2 90 90 90 90 IMP1
−52 12 −4 19 −130 −130 −130 −130 0 1 1 1 90 90 90 90 QRI5
−38 21 25 15 −130 −130 −130 −130 0 1 1 1 90 90 90 90 MRPL6
−36 17 35 15 −130 −130 −130 −130 −1 1 2 1 90 90 90 90 PET117
−37 18 19 13 −130 −130 −130 −130 −1 1 1 1 90 90 90 90 MSR1
−40 5 24 9 −130 −130 −130 −130 −1 0 1 1 90 90 90 90 COQ8
−46 8 17 4 −130 −130 −130 −130 −1 0 2 1 90 90 90 90 YGP1
−45 14 23 3 −130 −130 −130 −130 0 2 1 2 90 90 90 90 RSM27
−44 10 22 6 −130 −130 −130 −130 −1 1 1 2 90 90 90 90 MSM1
Gene Name
YPD.K.Shift
HL.K.INTERACTION
CSH.K.INTERACTION
HL.AS.K.INTERACTION
YPEG.K.Shift
HLEG.K.INTERACTION
CSHEG.K.INTERACTION
HLEG.AS.K.INTERACTION
YPD.L.SHIFT
HL.L.INTERACTION
CSH.L.INTERACTION
HL.AS.L.INTERACTION
YPEG.L.SHIFT
HLEG.L.INTERACTION
CSHEG.L.INTERACTION
HLEG.AS.L.INTERACTION
Type of Media

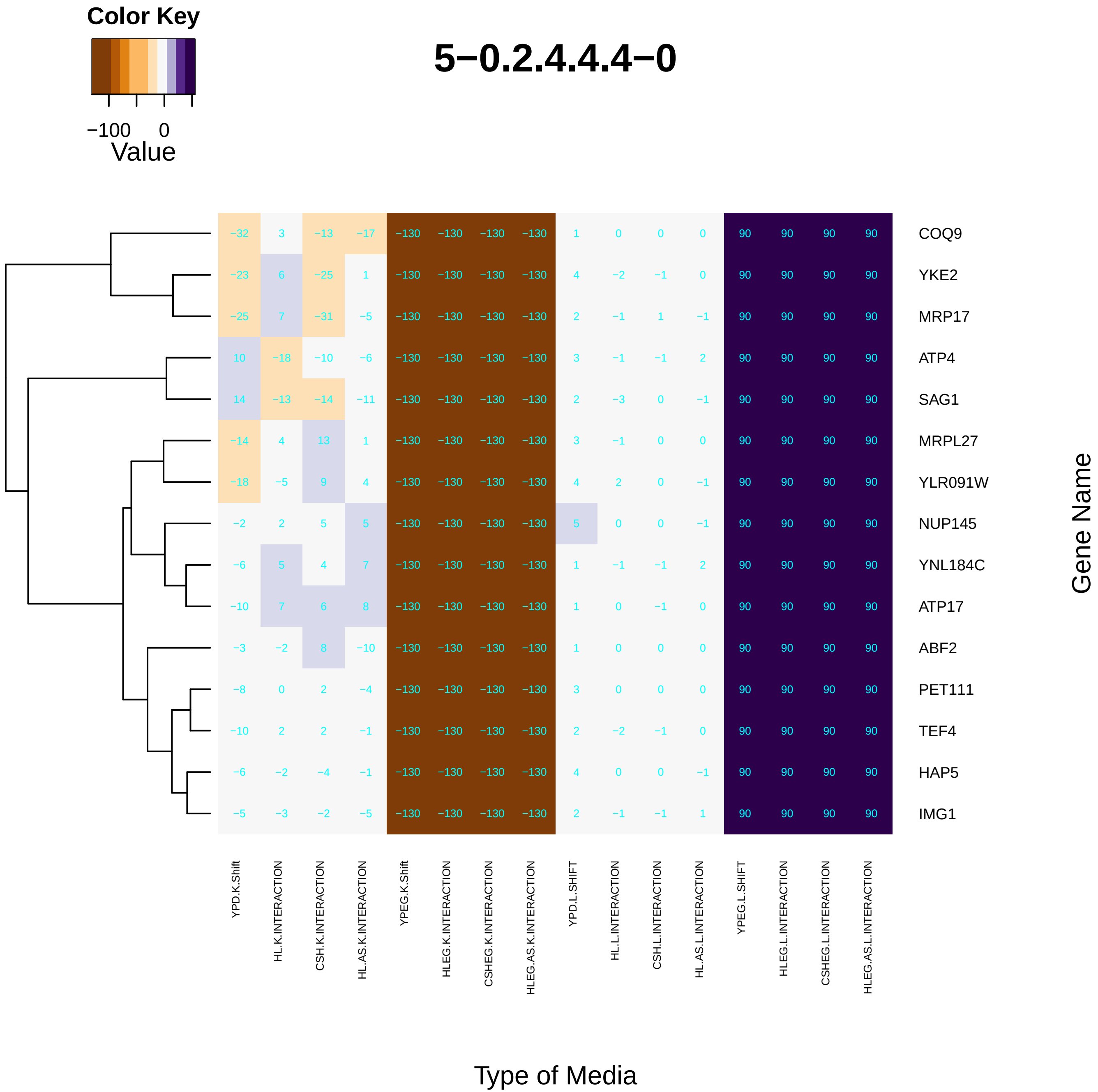
Color Key
−100 0
Value
5−0.2.4.4.4−0
YPD.K.Shift
HL.K.INTERACTION
CSH.K.INTERACTION
HL.AS.K.INTERACTION
YPEG.K.Shift
HLEG.K.INTERACTION
CSHEG.K.INTERACTION
HLEG.AS.K.INTERACTION
YPD.L.SHIFT
HL.L.INTERACTION
CSH.L.INTERACTION
HL.AS.L.INTERACTION
YPEG.L.SHIFT
HLEG.L.INTERACTION
CSHEG.L.INTERACTION
HLEG.AS.L.INTERACTION
COQ9 −32 3 −13 −17 −130 −130 −130 −130 1 0 0 0 90 90 90 90
YKE2 −23 6 −25 1 −130 −130 −130 −130 4 −2 −1 0 90 90 90 90
MRP17 −25 7 −31 −5 −130 −130 −130 −130 2 −1 1 −1 90 90 90 90
ATP4 10 −18 −10 −6 −130 −130 −130 −130 3 −1 −1 2 90 90 90 90
SAG1 14 −13 −14 −11 −130 −130 −130 −130 2 −3 0 −1 90 90 90 90
MRPL27 −14 4 13 1 −130 −130 −130 −130 3 −1 0 0 90 90 90 90
YLR091W −18 −5 9 4 −130 −130 −130 −130 4 2 0 −1 90 90 90 90
NUP145 −2 2 5 5 −130 −130 −130 −130 5 0 0 −1 90 90 90 90
YNL184C −6 5 4 7 −130 −130 −130 −130 1 −1 −1 2 90 90 90 90
ATP17 −10 7 6 8 −130 −130 −130 −130 1 0 −1 0 90 90 90 90
ABF2 −3 −2 8 −10 −130 −130 −130 −130 1 0 0 0 90 90 90 90
PET111 −8 0 2 −4 −130 −130 −130 −130 3 0 0 0 90 90 90 90
TEF4 −10 2 2 −1 −130 −130 −130 −130 2 −2 −1 0 90 90 90 90
HAP5 −6 −2 −4 −1 −130 −130 −130 −130 4 0 0 −1 90 90 90 90
IMG1 −5 −3 −2 −5 −130 −130 −130 −130 2 −1 −1 1 90 90 90 90
Gene Name
Type of Media

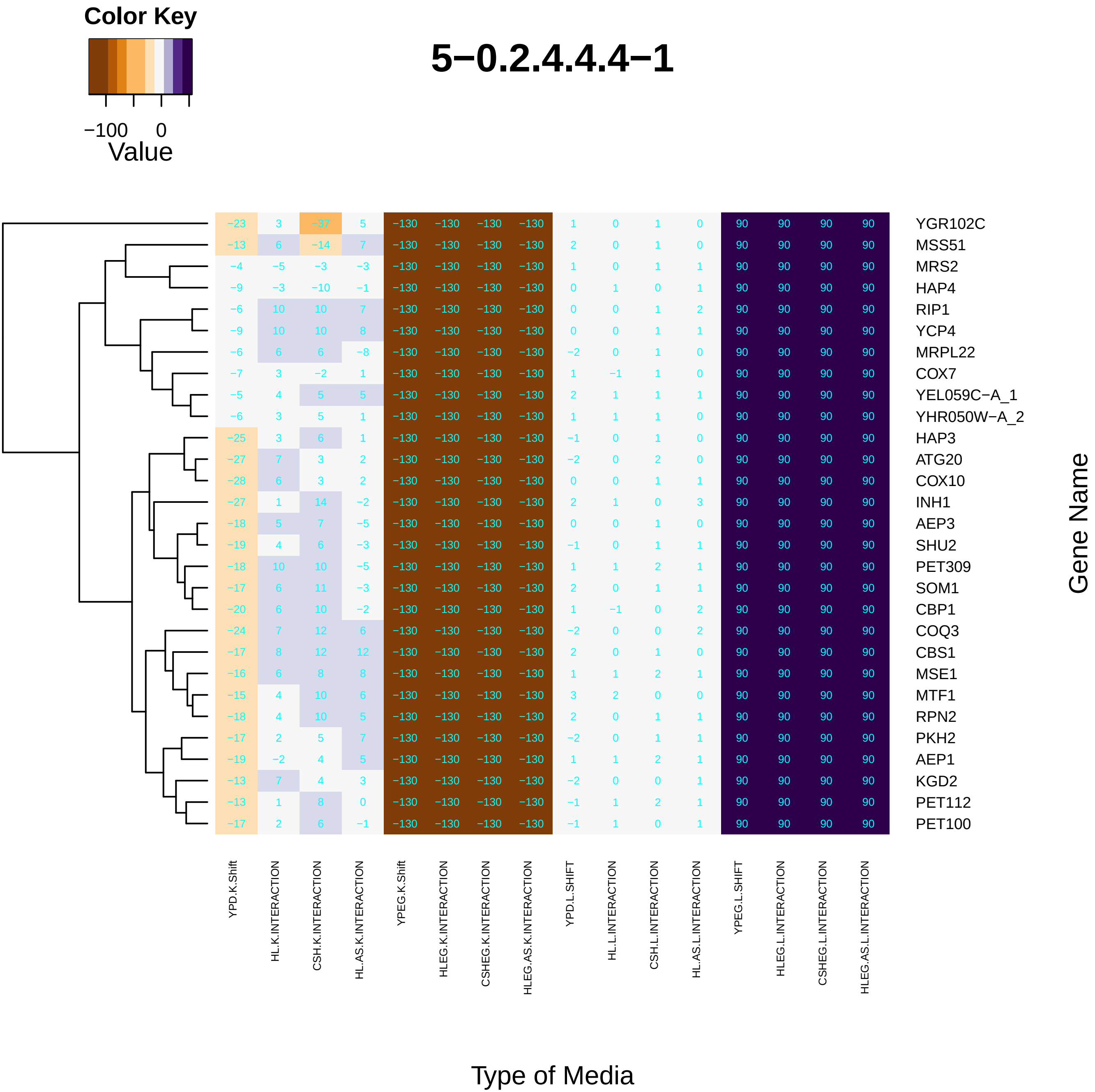
Color Key
−100
0
Value
5−0.2.4.4.4−1
YPD.K.Shift
HL.K.INTERACTION
CSH.K.INTERACTION
HL.AS.K.INTERACTION
YPEG.K.Shift
HLEG.K.INTERACTION
CSHEG.K.INTERACTION
HLEG.AS.K.INTERACTION
YPD.L.SHIFT
HL.L.INTERACTION
CSH.L.INTERACTION
HL.AS.L.INTERACTION
YPEG.L.SHIFT
HLEG.L.INTERACTION
CSHEG.L.INTERACTION
HLEG.AS.L.INTERACTION
YGR102C −23 3 −37 5 −130 −130 −130 −130 1 0 1 0 90 90 90 90
MSS51 −13 6 −14 7 −130 −130 −130 −130 2 0 1 0 90 90 90 90
MRS2 −4 −5 −3 −3 −130 −130 −130 −130 1 0 1 1 90 90 90 90
HAP4 −9 −3 −10 −1 −130 −130 −130 −130 0 1 0 1 90 90 90 90
RIP1 −6 10 10 7 −130 −130 −130 −130 0 0 1 2 90 90 90 90
YCP4 −9 10 10 8 −130 −130 −130 −130 0 0 1 1 90 90 90 90
MRPL22 −6 6 6 −8 −130 −130 −130 −130 −2 0 1 0 90 90 90 90
COX7 −7 3 −2 1 −130 −130 −130 −130 1 −1 1 0 90 90 90 90
YEL059C−A_1 −5 4 5 5 −130 −130 −130 −130 2 1 1 1 90 90 90 90
YHR050W−A_2 −6 3 5 1 −130 −130 −130 −130 1 1 1 0 90 90 90 90
HAP3 −25 3 6 1 −130 −130 −130 −130 −1 0 1 0 90 90 90 90
ATG20 −27 7 3 2 −130 −130 −130 −130 −2 0 2 0 90 90 90 90
COX10 −28 6 3 2 −130 −130 −130 −130 0 0 1 1 90 90 90 90
INH1 −27 1 14 −2 −130 −130 −130 −130 2 1 0 3 90 90 90 90
AEP3 −18 5 7 −5 −130 −130 −130 −130 0 0 1 0 90 90 90 90
SHU2 −19 4 6 −3 −130 −130 −130 −130 −1 0 1 1 90 90 90 90
PET309 −18 10 10 −5 −130 −130 −130 −130 1 1 2 1 90 90 90 90
SOM1 −17 6 11 −3 −130 −130 −130 −130 2 0 1 1 90 90 90 90
CBP1 −20 6 10 −2 −130 −130 −130 −130 1 −1 0 2 90 90 90 90
COQ3 −24 7 12 6 −130 −130 −130 −130 −2 0 0 2 90 90 90 90
CBS1 −17 8 12 12 −130 −130 −130 −130 2 0 1 0 90 90 90 90
MSE1 −16 6 8 8 −130 −130 −130 −130 1 1 2 1 90 90 90 90
MTF1 −15 4 10 6 −130 −130 −130 −130 3 2 0 0 90 90 90 90
RPN2 −18 4 10 5 −130 −130 −130 −130 2 0 1 1 90 90 90 90
PKH2 −17 2 5 7 −130 −130 −130 −130 −2 0 1 1 90 90 90 90
AEP1 −19 −2 4 5 −130 −130 −130 −130 1 1 2 1 90 90 90 90
KGD2 −13 7 4 3 −130 −130 −130 −130 −2 0 0 1 90 90 90 90
PET112 −13 1 8 0 −130 −130 −130 −130 −1 1 2 1 90 90 90 90
PET100 −17 2 6 −1 −130 −130 −130 −130 −1 1 0 1 90 90 90 90
Gene Name
Type of Media

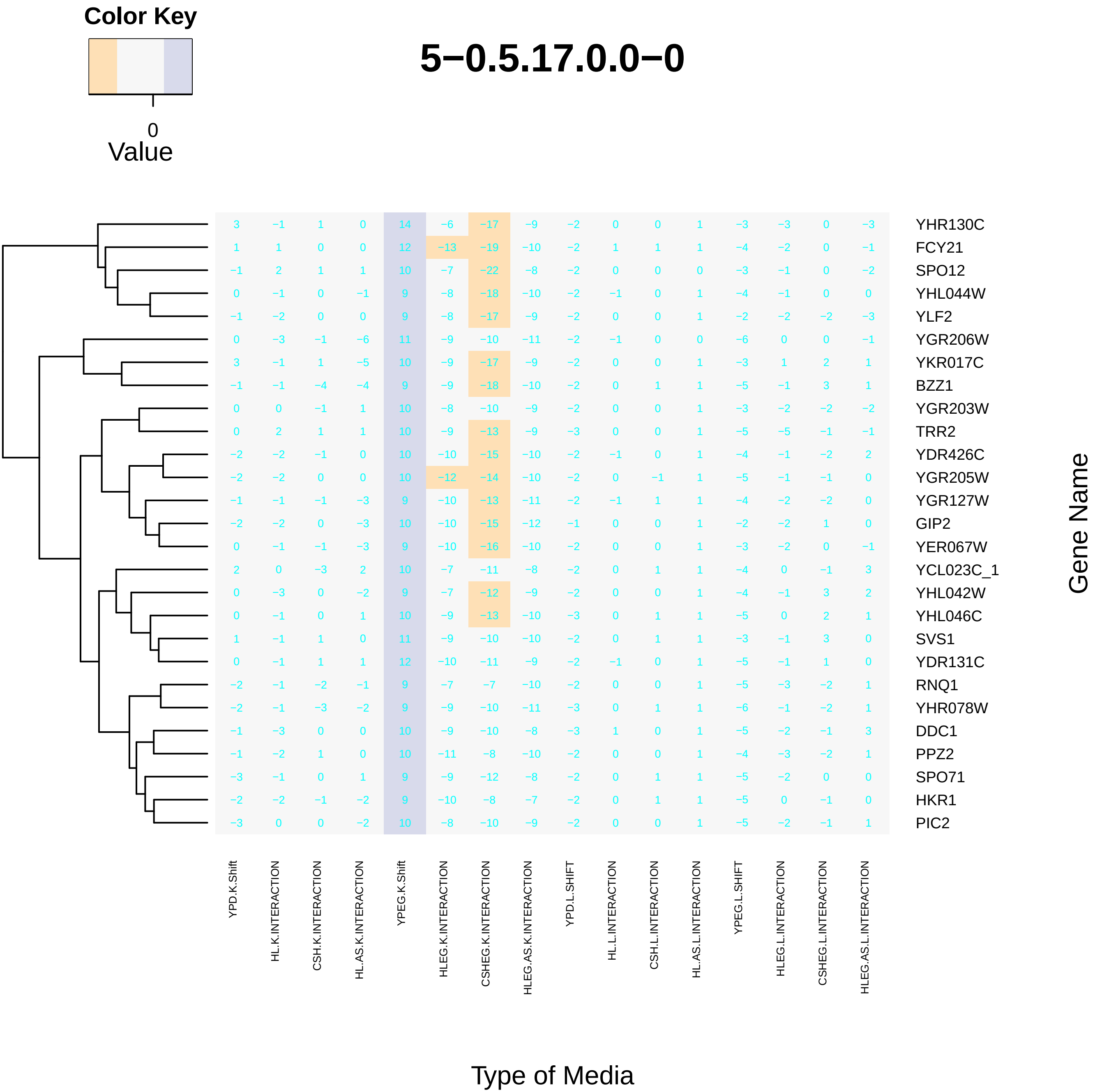
Color Key
0
Value
5−0.5.17.0.0−0
YHR130C 3 −1 1 0 14 −6 −17 −9 −2 0 0 1 −3 −3 0 −3
FCY21 1 1 0 0 12 −13 −19 −10 −2 1 1 1 −4 −2 0 −1
SPO12 −1 2 1 1 10 −7 −22 −8 −2 0 0 0 −3 −1 0 −2
YHL044W 0 −1 0 −1 9 −8 −18 −10 −2 −1 0 1 −4 −1 0 0
YLF2 −1 −2 0 0 9 −8 −17 −9 −2 0 0 1 −2 −2 −2 −3
YGR206W 0 −3 −1 −6 11 −9 −10 −11 −2 −1 0 0 −6 0 0 −1
YKR017C 3 −1 1 −5 10 −9 −17 −9 −2 0 0 1 −3 1 2 1
BZZ1 −1 −1 −4 −4 9 −9 −18 −10 −2 0 1 1 −5 −1 3 1
YGR203W 0 0 −1 1 10 −8 −10 −9 −2 0 0 1 −3 −2 −2 −2
TRR2 0 2 1 1 10 −9 −13 −9 −3 0 0 1 −5 −5 −1 −1
YDR426C −2 −2 −1 0 10 −10 −15 −10 −2 −1 0 1 −4 −1 −2 2
YGR205W −2 −2 0 0 10 −12 −14 −10 −2 0 −1 1 −5 −1 −1 0
YGR127W −1 −1 −1 −3 9 −10 −13 −11 −2 −1 1 1 −4 −2 −2 0
GIP2 −2 −2 0 −3 10 −10 −15 −12 −1 0 0 1 −2 −2 1 0
YER067W 0 −1 −1 −3 9 −10 −16 −10 −2 0 0 1 −3 −2 0 −1
YCL023C_1 2 0 −3 2 10 −7 −11 −8 −2 0 1 1 −4 0 −1 3
YHL042W 0 −3 0 −2 9 −7 −12 −9 −2 0 0 1 −4 −1 3 2
YHL046C 0 −1 0 1 10 −9 −13 −10 −3 0 1 1 −5 0 2 1
SVS1 1 −1 1 0 11 −9 −10 −10 −2 0 1 1 −3 −1 3 0
YDR131C 0 −1 1 1 12 −10 −11 −9 −2 −1 0 1 −5 −1 1 0
RNQ1 −2 −1 −2 −1 9 −7 −7 −10 −2 0 0 1 −5 −3 −2 1
YHR078W −2 −1 −3 −2 9 −9 −10 −11 −3 0 1 1 −6 −1 −2 1
DDC1 −1 −3 0 0 10 −9 −10 −8 −3 1 0 1 −5 −2 −1 3
PPZ2 −1 −2 1 0 10 −11 −8 −10 −2 0 0 1 −4 −3 −2 1
SPO71 −3 −1 0 1 9 −9 −12 −8 −2 0 1 1 −5 −2 0 0
HKR1 −2 −2 −1 −2 9 −10 −8 −7 −2 0 1 1 −5 0 −1 0
PIC2 −3 0 0 −2 10 −8 −10 −9 −2 0 0 1 −5 −2 −1 1
YPD.K.Shift
HL.K.INTERACTION
CSH.K.INTERACTION
HL.AS.K.INTERACTION
YPEG.K.Shift
HLEG.K.INTERACTION
CSHEG.K.INTERACTION
HLEG.AS.K.INTERACTION
YPD.L.SHIFT
HL.L.INTERACTION
CSH.L.INTERACTION
HL.AS.L.INTERACTION
YPEG.L.SHIFT
HLEG.L.INTERACTION
CSHEG.L.INTERACTION
HLEG.AS.L.INTERACTION
Gene Name
Type of Media

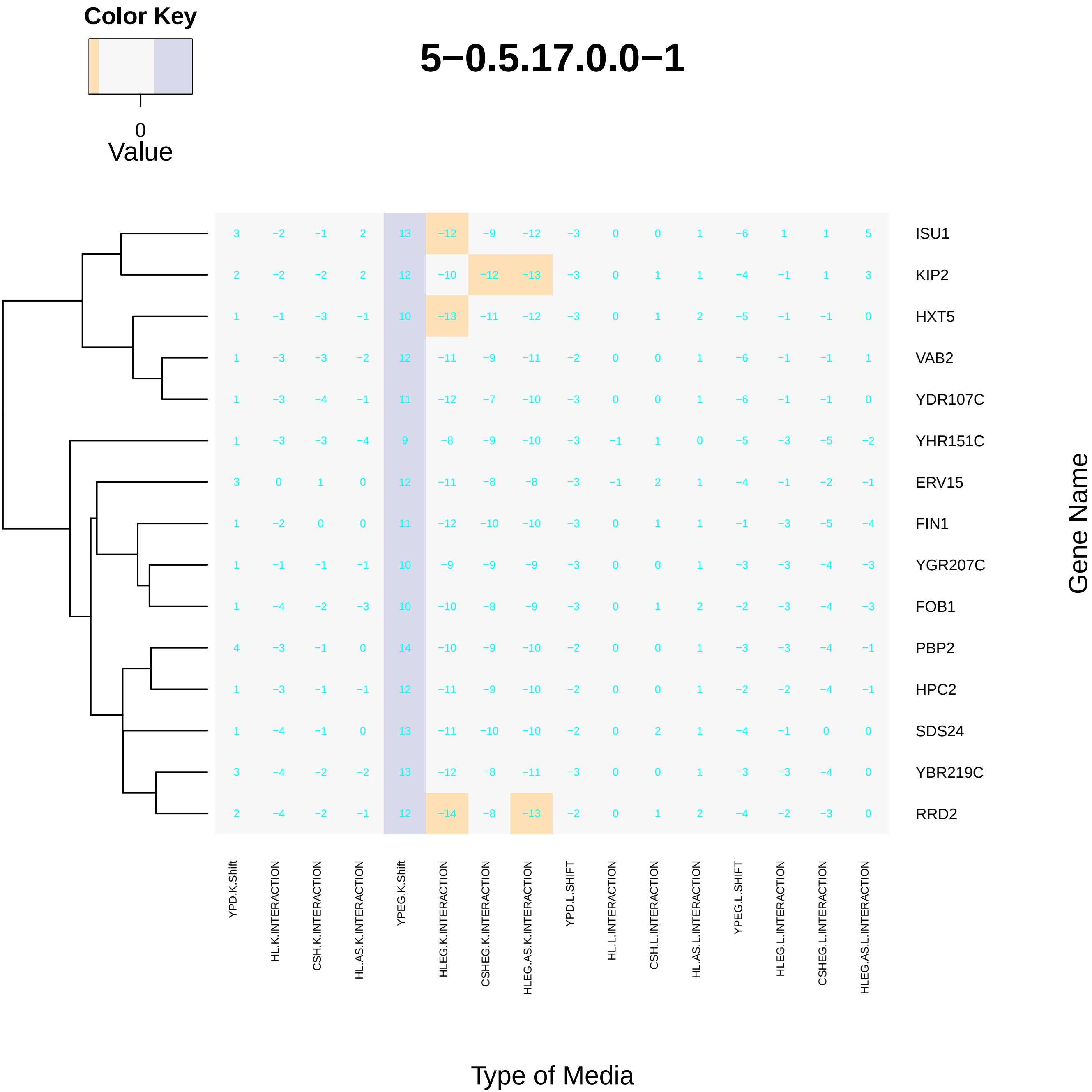
Color Key
0
Value
5−0.5.17.0.0−1
YPD.K.Shift HL.K.INTERACTION CSH.K.INTERACTION HL.AS.K.INTERACTION YPEG.K.Shift HLEG.K.INTERACTION CSHEG.K.INTERACTION HLEG.AS.K.INTERACTION YPD.L.SHIFT HL.L.INTERACTION CSH.L.INTERACTION HL.AS.L.INTERACTION YPEG.L.SHIFT HLEG.L.INTERACTION CSHEG.L.INTERACTION HLEG.AS.L.INTERACTION
ISU1 3 −2 −1 2 13 −12 −9 −12 −3 0 0 1 −6 1 1 5
KIP2 2 −2 −2 2 12 −10 −12 −13 −3 0 1 1 −4 −1 1 3
HXT5 1 −1 −3 −1 10 −13 −11 −12 −3 0 1 2 −5 −1 −1 0
VAB2 1 −3 −3 −2 12 −11 −9 −11 −2 0 0 1 −6 −1 −1 1
YDR107C 1 −3 −4 −1 11 −12 −7 −10 −3 0 0 1 −6 −1 −1 0
YHR151C 1 −3 −3 −4 9 −8 −9 −10 −3 −1 1 0 −5 −3 −5 −2
ERV15 3 0 1 0 12 −11 −8 −8 −3 −1 2 1 −4 −1 −2 −1
FIN1 1 −2 0 0 11 −12 −10 −10 −3 0 1 1 −1 −3 −5 −4
YGR207C 1 −1 −1 −1 10 −9 −9 −9 −3 0 0 1 −3 −3 −4 −3
FOB1 1 −4 −2 −3 10 −10 −8 −9 −3 0 1 2 −2 −3 −4 −3
PBP2 4 −3 −1 0 14 −10 −9 −10 −2 0 0 1 −3 −3 −4 −1
HPC2 1 −3 −1 −1 12 −11 −9 −10 −2 0 0 1 −2 −2 −4 −1
SDS24 1 −4 −1 0 13 −11 −10 −10 −2 0 2 1 −4 −1 0 0
YBR219C 3 −4 −2 −2 13 −12 −8 −11 −3 0 0 1 −3 −3 −4 0
RRD2 2 −4 −2 −1 12 −14 −8 −13 −2 0 1 2 −4 −2 −3 0
Gene Name
Type of Media

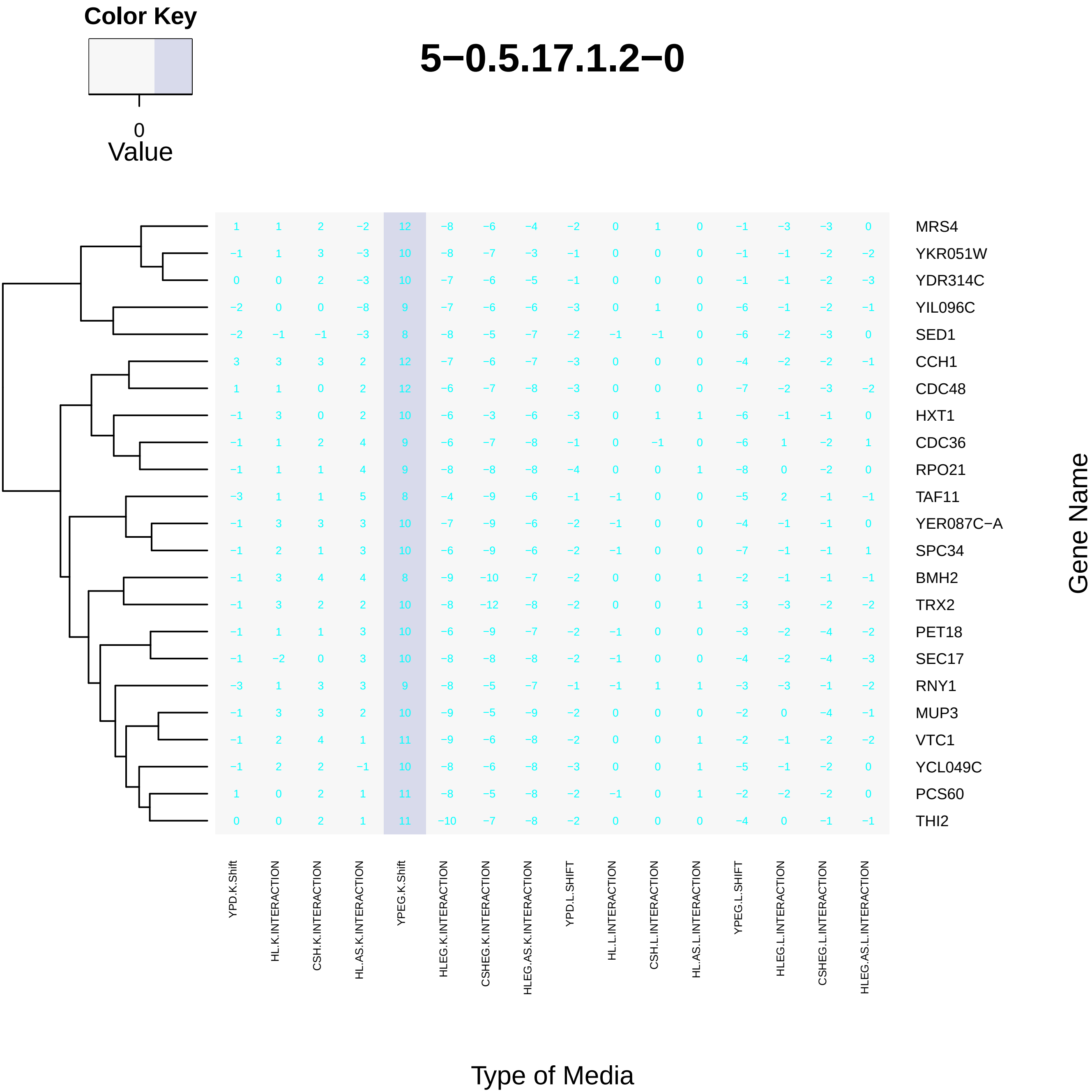
Color Key
0
Value
5−0.5.17.1.2−0
YPD.K.Shift HL.K.INTERACTION CSH.K.INTERACTION HL.AS.K.INTERACTION YPEG.K.Shift HLEG.K.INTERACTION CSHEG.K.INTERACTION HLEG.AS.K.INTERACTION YPD.L.SHIFT HL.L.INTERACTION CSH.L.INTERACTION HL.AS.L.INTERACTION YPEG.L.SHIFT HLEG.L.INTERACTION CSHEG.L.INTERACTION HLEG.AS.L.INTERACTION
MRS4 1 1 2 −2 12 −8 −6 −4 −2 0 1 0 −1 −3 −3 0
YKR051W −1 1 3 −3 10 −8 −7 −3 −1 0 0 0 −1 −1 −2 −2
YDR314C 0 0 2 −3 10 −7 −6 −5 −1 0 0 0 −1 −1 −2 −3
YIL096C −2 0 0 −8 9 −7 −6 −6 −3 0 1 0 −6 −1 −2 −1
SED1 −2 −1 −1 −3 8 −8 −5 −7 −2 −1 −1 0 −6 −2 −3 0
CCH1 3 3 3 2 12 −7 −6 −7 −3 0 0 0 −4 −2 −2 −1
CDC48 1 1 0 2 12 −6 −7 −8 −3 0 0 0 −7 −2 −3 −2
HXT1 −1 3 0 2 10 −6 −3 −6 −3 0 1 1 −6 −1 −1 0
CDC36 −1 1 2 4 9 −6 −7 −8 −1 0 −1 0 −6 1 −2 1
RPO21 −1 1 1 4 9 −8 −8 −8 −4 0 0 1 −8 0 −2 0
TAF11 −3 1 1 5 8 −4 −9 −6 −1 −1 0 0 −5 2 −1 −1
YER087C−A −1 3 3 3 10 −7 −9 −6 −2 −1 0 0 −4 −1 −1 0
SPC34 −1 2 1 3 10 −6 −9 −6 −2 −1 0 0 −7 −1 −1 1
BMH2 −1 3 4 4 8 −9 −10 −7 −2 0 0 1 −2 −1 −1 −1
TRX2 −1 3 2 2 10 −8 −12 −8 −2 0 0 1 −3 −3 −2 −2
PET18 −1 1 1 3 10 −6 −9 −7 −2 −1 0 0 −3 −2 −4 −2
SEC17 −1 −2 0 3 10 −8 −8 −8 −2 −1 0 0 −4 −2 −4 −3
RNY1 −3 1 3 3 9 −8 −5 −7 −1 −1 1 1 −3 −3 −1 −2
MUP3 −1 3 3 2 10 −9 −5 −9 −2 0 0 0 −2 0 −4 −1
VTC1 −1 2 4 1 11 −9 −6 −8 −2 0 0 1 −2 −1 −2 −2
YCL049C −1 2 2 −1 10 −8 −6 −8 −3 0 0 1 −5 −1 −2 0
PCS60 1 0 2 1 11 −8 −5 −8 −2 −1 0 1 −2 −2 −2 0
THI2 0 0 2 1 11 −10 −7 −8 −2 0 0 0 −4 0 −1 −1
Gene Name
Type of Media

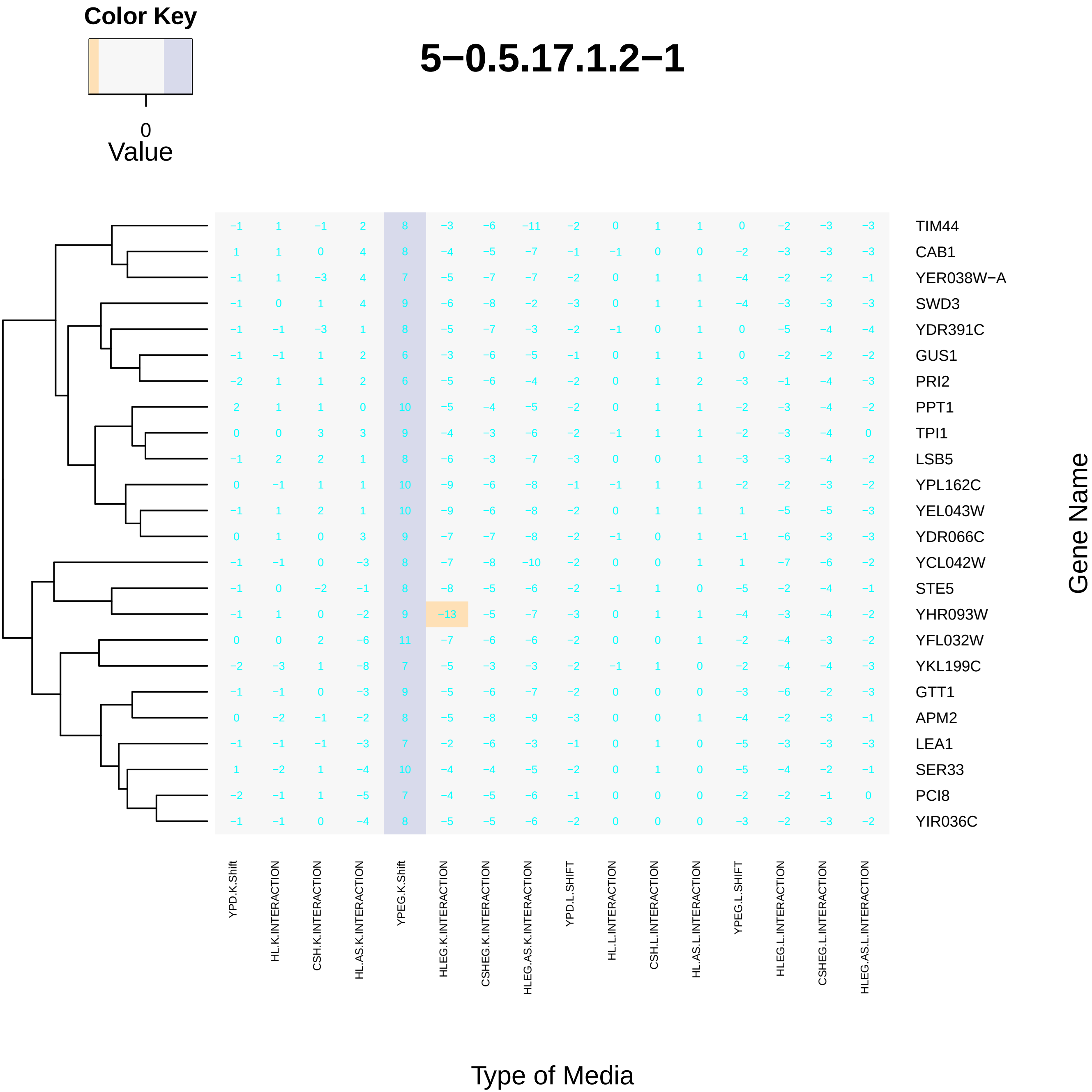
Color Key
0
Value
5−0.5.17.1.2−1
YPD.K.Shift HL.K.INTERACTION CSH.K.INTERACTION HL.AS.K.INTERACTION YPEG.K.Shift HLEG.K.INTERACTION CSHEG.K.INTERACTION HLEG.AS.K.INTERACTION YPD.L.SHIFT HL.L.INTERACTION CSH.L.INTERACTION HL.AS.L.INTERACTION YPEG.L.SHIFT HLEG.L.INTERACTION CSHEG.L.INTERACTION HLEG.AS.L.INTERACTION
TIM44 −1 1 −1 2 8 −3 −6 −11 −2 0 1 1 0 −2 −3 −3
CAB1 1 1 0 4 8 −4 −5 −7 −1 −1 0 0 −2 −3 −3 −3
YER038W−A −1 1 −3 4 7 −5 −7 −7 −2 0 1 1 −4 −2 −2 −1
SWD3 −1 0 1 4 9 −6 −8 −2 −3 0 1 1 −4 −3 −3 −3
YDR391C −1 −1 −3 1 8 −5 −7 −3 −2 −1 0 1 0 −5 −4 −4
GUS1 −1 −1 1 2 6 −3 −6 −5 −1 0 1 1 0 −2 −2 −2
PRI2 −2 1 1 2 6 −5 −6 −4 −2 0 1 2 −3 −1 −4 −3
PPT1 2 1 1 0 10 −5 −4 −5 −2 0 1 1 −2 −3 −4 −2
TPI1 0 0 3 3 9 −4 −3 −6 −2 −1 1 1 −2 −3 −4 0
LSB5 −1 2 2 1 8 −6 −3 −7 −3 0 0 1 −3 −3 −4 −2
YPL162C 0 −1 1 1 10 −9 −6 −8 −1 −1 1 1 −2 −2 −3 −2
YEL043W −1 1 2 1 10 −9 −6 −8 −2 0 1 1 1 −5 −5 −3
YDR066C 0 1 0 3 9 −7 −7 −8 −2 −1 0 1 −1 −6 −3 −3
YCL042W −1 −1 0 −3 8 −7 −8 −10 −2 0 0 1 1 −7 −6 −2
STE5 −1 0 −2 −1 8 −8 −5 −6 −2 −1 1 0 −5 −2 −4 −1
YHR093W −1 1 0 −2 9 −13 −5 −7 −3 0 1 1 −4 −3 −4 −2
YFL032W 0 0 2 −6 11 −7 −6 −6 −2 0 0 1 −2 −4 −3 −2
YKL199C −2 −3 1 −8 7 −5 −3 −3 −2 −1 1 0 −2 −4 −4 −3
GTT1 −1 −1 0 −3 9 −5 −6 −7 −2 0 0 0 −3 −6 −2 −3
APM2 0 −2 −1 −2 8 −5 −8 −9 −3 0 0 1 −4 −2 −3 −1
LEA1 −1 −1 −1 −3 7 −2 −6 −3 −1 0 1 0 −5 −3 −3 −3
SER33 1 −2 1 −4 10 −4 −4 −5 −2 0 1 0 −5 −4 −2 −1
PCI8 −2 −1 1 −5 7 −4 −5 −6 −1 0 0 0 −2 −2 −1 0
YIR036C −1 −1 0 −4 8 −5 −5 −6 −2 0 0 0 −3 −2 −3 −2
Gene Name
Type of Media

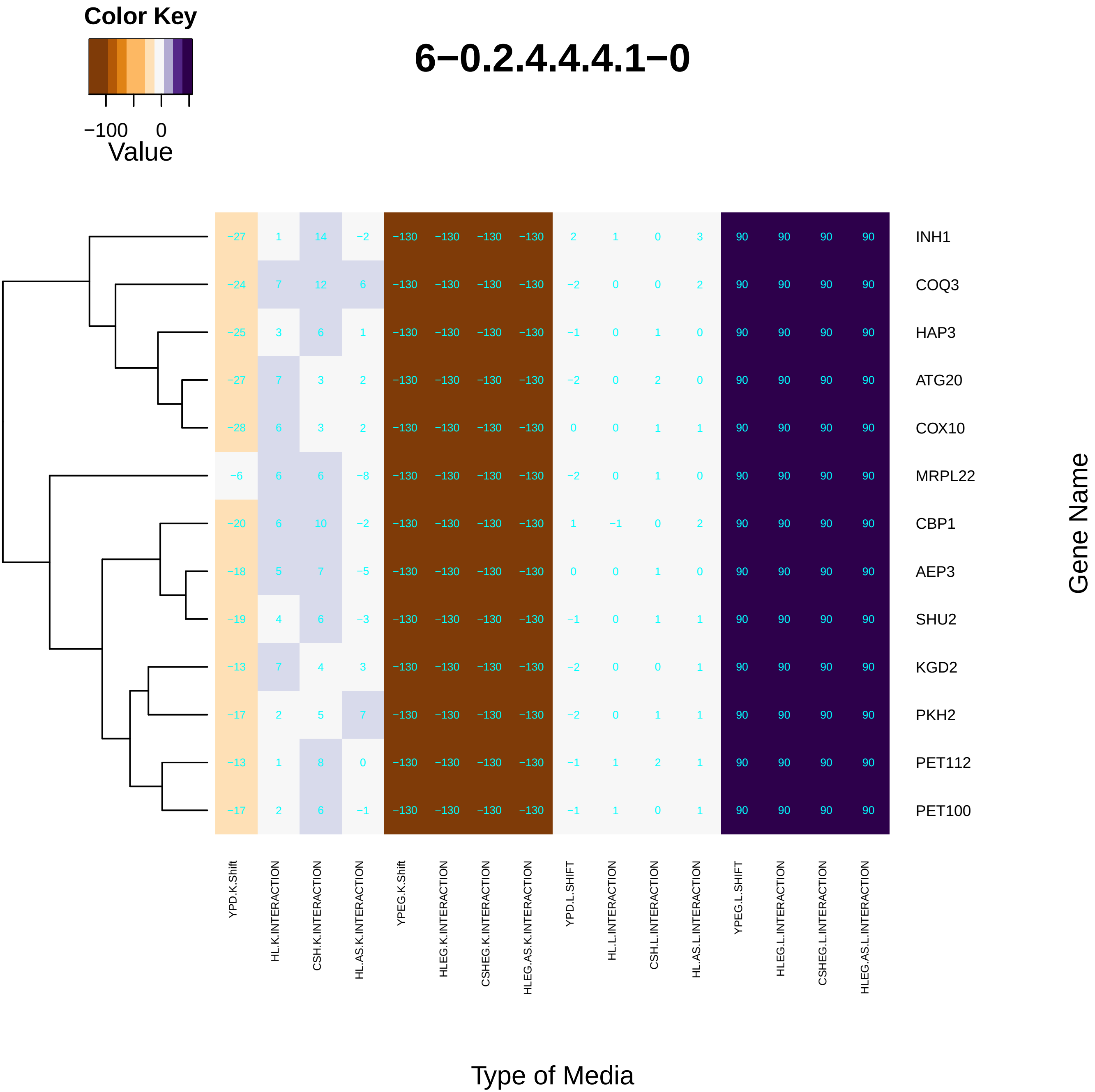
Color Key
−100 0
Value
6−0.2.4.4.4.1−0
INH1 −27 1 14 −2 −130 −130 −130 −130 2 1 0 3 90 90 90 90
COQ3 −24 7 12 6 −130 −130 −130 −130 −2 0 0 2 90 90 90 90
HAP3 −25 3 6 1 −130 −130 −130 −130 −1 0 1 0 90 90 90 90
ATG20 −27 7 3 2 −130 −130 −130 −130 −2 0 2 0 90 90 90 90
COX10 −28 6 3 2 −130 −130 −130 −130 0 0 1 1 90 90 90 90
MRPL22 −6 6 6 −8 −130 −130 −130 −130 −2 0 1 0 90 90 90 90
CBP1 −20 6 10 −2 −130 −130 −130 −130 1 −1 0 2 90 90 90 90
AEP3 −18 5 7 −5 −130 −130 −130 −130 0 0 1 0 90 90 90 90
SHU2 −19 4 6 −3 −130 −130 −130 −130 −1 0 1 1 90 90 90 90
KGD2 −13 7 4 3 −130 −130 −130 −130 −2 0 0 1 90 90 90 90
PKH2 −17 2 5 7 −130 −130 −130 −130 −2 0 1 1 90 90 90 90
PET112 −13 1 8 0 −130 −130 −130 −130 −1 1 2 1 90 90 90 90
PET100 −17 2 6 −1 −130 −130 −130 −130 −1 1 0 1 90 90 90 90
YPD.K.Shift
HL.K.INTERACTION
CSH.K.INTERACTION
HL.AS.K.INTERACTION
YPEG.K.Shift
HLEG.K.INTERACTION
CSHEG.K.INTERACTION
HLEG.AS.K.INTERACTION
YPD.L.SHIFT
HL.L.INTERACTION
CSH.L.INTERACTION
HL.AS.L.INTERACTION
YPEG.L.SHIFT
HLEG.L.INTERACTION
CSHEG.L.INTERACTION
HLEG.AS.L.INTERACTION
Gene Name
Type of Media

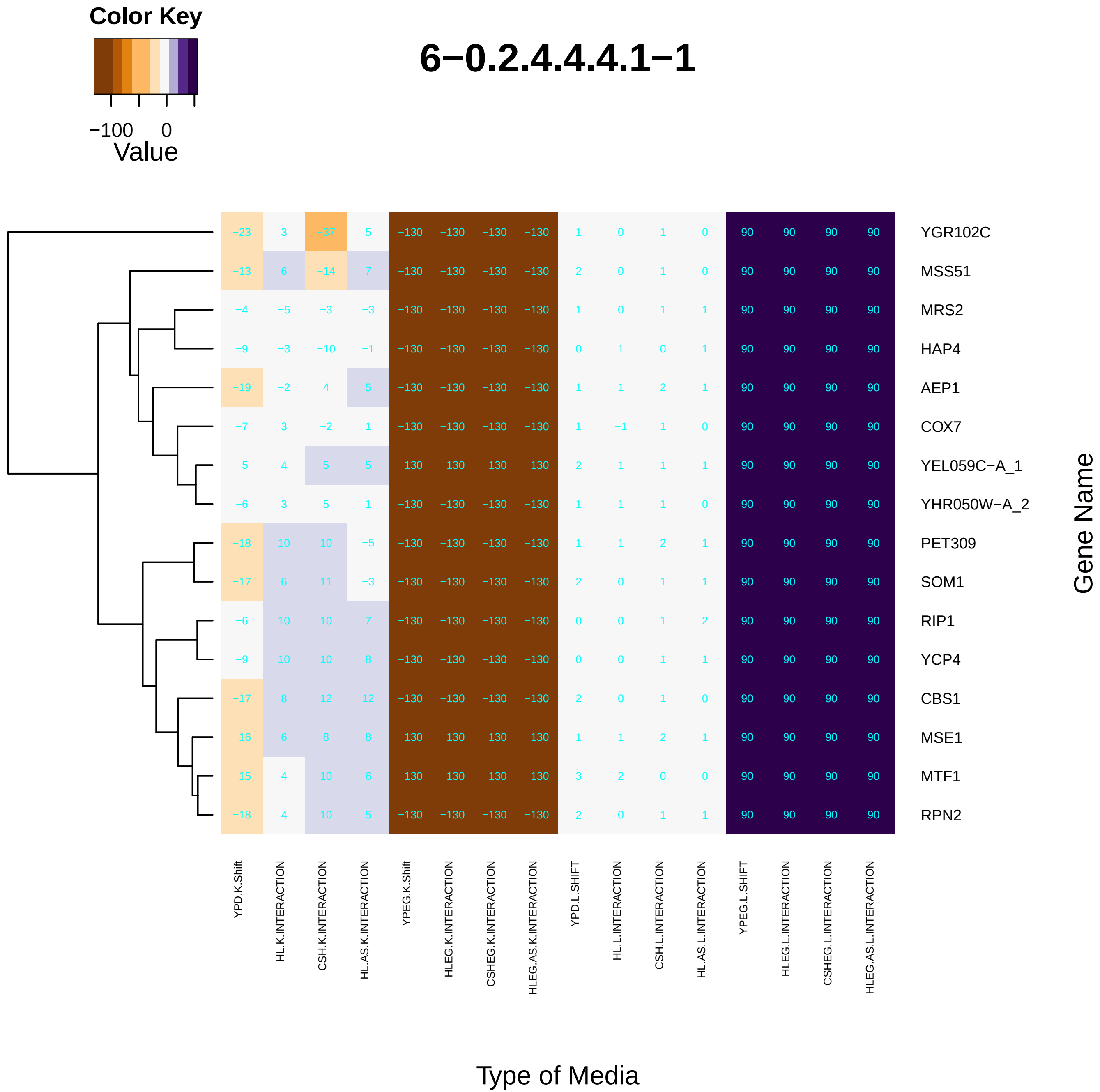
Color Key
−100 0
Value
6−0.2.4.4.4.1−1
YGR102C −23 3 −37 5 −130 −130 −130 −130 1 0 1 0 90 90 90 90
MSS51 −13 6 −14 7 −130 −130 −130 −130 2 0 1 0 90 90 90 90
MRS2 −4 −5 −3 −3 −130 −130 −130 −130 1 0 1 1 90 90 90 90
HAP4 −9 −3 −10 −1 −130 −130 −130 −130 0 1 0 1 90 90 90 90
AEP1 −19 −2 4 5 −130 −130 −130 −130 1 1 2 1 90 90 90 90
COX7 −7 3 −2 1 −130 −130 −130 −130 1 −1 1 0 90 90 90 90
YEL059C−A_1 −5 4 5 5 −130 −130 −130 −130 2 1 1 1 90 90 90 90
YHR050W−A_2 −6 3 5 1 −130 −130 −130 −130 1 1 1 0 90 90 90 90
PET309 −18 10 10 −5 −130 −130 −130 −130 1 1 2 1 90 90 90 90
SOM1 −17 6 11 −3 −130 −130 −130 −130 2 0 1 1 90 90 90 90
RIP1 −6 10 10 7 −130 −130 −130 −130 0 0 1 2 90 90 90 90
YCP4 −9 10 10 8 −130 −130 −130 −130 0 0 1 1 90 90 90 90
CBS1 −17 8 12 12 −130 −130 −130 −130 2 0 1 0 90 90 90 90
MSE1 −16 6 8 8 −130 −130 −130 −130 1 1 2 1 90 90 90 90
MTF1 −15 4 10 6 −130 −130 −130 −130 3 2 0 0 90 90 90 90
RPN2 −18 4 10 5 −130 −130 −130 −130 2 0 1 1 90 90 90 90
Gene Name
YPD.K.Shift
HL.K.INTERACTION
CSH.K.INTERACTION
HL.AS.K.INTERACTION
YPEG.K.Shift
HLEG.K.INTERACTION
CSHEG.K.INTERACTION
HLEG.AS.K.INTERACTION
YPD.L.SHIFT
HL.L.INTERACTION
CSH.L.INTERACTION
HL.AS.L.INTERACTION
YPEG.L.SHIFT
HLEG.L.INTERACTION
CSHEG.L.INTERACTION
HLEG.AS.L.INTERACTION
Type of Media
